# High Expression of ACOT2 Predicts Worse Overall Survival and Abnormal Lipid Metabolism: A Potential Target for Acute Myeloid Leukemia

**DOI:** 10.1155/2022/2669114

**Published:** 2022-09-23

**Authors:** Xuewei Yin, Chunyi Lyu, Zonghong Li, Qian Wang, Yi Ding, Yan Wang, Yan Qiu, Siyuan Cui, Dadong Guo, Ruirong Xu

**Affiliations:** ^1^Shandong University of Traditional Chinese Medicine, Jinan 250000, China; ^2^The Second Affiliated Hospital of Shandong University of Traditional Chinese Medicine, Jinan 250002, China; ^3^Shandong University of Traditional Chinese Medicine Affiliated Hospital, Key Laboratory of Integrated Traditional Chinese and Western Medicine Hematology, Shandong Provincial Department of Health, Institute of Hematology, Shandong University of Traditional Chinese Medicine, Jinan 250000, China; ^4^Shandong Provincial Key Laboratory of Integrated Traditional Chinese and Western Medicine for Prevention and Therapy of Ocular Diseases, Shandong Academy of Eye Disease Prevention and Therapy, Medical College of Optometry and Ophthalmology, Shandong University of Traditional Chinese Medicine, Jinan 250000, China

## Abstract

Acyl-CoA thioesterase (ACOT) plays a considerable role in lipid metabolism, which is closely related to the occurrence and development of cancer, nevertheless, its role has not been fully elucidated in acute myeloid leukemia (AML). To explore the role of ACOT2 in AML and to provide a potential therapeutic target for AML, the expression pattern of ACOT was investigated based on the TNMplot, Gene Expression Profiling Interactive Analysis (GEPIA), and Cancer Cell Line Encyclopedia (CCLE) database, and diagnostic value, prognostic value, and clinical phenotype of ACOT were explored based on data from The Cancer Genome Atlas (TCGA). Functional annotation and enrichment analysis of the common targets between ACOT2 coexpressed and AML-related genes were further performed by Gene Ontology (GO), Kyoto Encyclopedia of Genes and Genomes (KEGG), and Gene Set Enrichment Analysis (GSEA) analyses. The protein-protein interaction (PPI) network of ACOT2 coexpressed genes and functional ACOT2-related metabolites association network were constructed based on GeneMANIA and Human Metabolome Database. Among ACOTs, ACOT2 was highly expressed in AML compared to normal control subjects according to TNMplot, GEPIA, and CCLE database, which was significantly associated with poor overall survival (OS) in AML (*P*=0.003). Moreover, ACOT2 exhibited excellent diagnostic efficiency for AML (AUC: 1.000) and related to French-American-British (FAB) classification and cytogenetics. GO, KEGG, and GSEA analyses of 71 common targets between ACOT2 coexpressed and AML-related genes revealed that ACOT2 is closely related to ACOT1, ACOT4, enoyl-acyl carrier protein reductase, mitochondrial (MECR), puromycin-sensitive aminopeptidase (NPEPPS), SWI/SNF-related matrix-associated actin-dependent regulator of chromatin subfamily B member 1 (SMARCB1), and long-chain fatty acid-CoA ligase 1 (ACSL1) in PPI network, and plays a significant role in lipid metabolism, that is, involved in fatty acid elongation and biosynthesis of unsaturated fatty acids. Collectively, the increase of ACOT2 may be an important characteristic of worse OS and abnormal lipid metabolism, suggesting that ACOT2 may become a potential therapeutic target for AML.

## 1. Introduction

Acute myeloid leukemia (AML) is a neoplastic disease originating from myeloid progenitor cells characterized by malignant proliferation and differentiation, which is often accompanied by proliferative diseases such as severe leukocytosis [1–3]. Although various new treatments such as allogeneic hematopoietic stem cell transplantation (AHSCT) and chimeric antigen receptor (CAR)-T cells have recently been recommended, the low cure rate of AML is still a serious problem. Metabolic abnormalities are the main signs of cancer. Oncogenic signaling pathway can mediate the expression of metabolic genes and improve the activity of metabolic enzymes, and abnormal cancer metabolism plays a significant role in tumorigenesis, metastasis, cancer stem cells, and drug resistance [4]. In recent years, an increasing number of researchers are interested in the study of tumor cell metabolism. Studies have shown that hematological malignancies can reshape the pathway of glucose and lipid metabolism into aerobic glycolysis so as to support and promote tumor formation [5, 6]. Xu et al. [7] found that vitamin D (1,25VD3)-induced overexpression of fructose-1,6-bisphosphatase 1 (FBP1) regulates different metabolic processes in AML, which may serve as an attractive approach to block energy production in AML. Thus, targeted intervention in cancer metabolism has emerged as a potential strategy to ameliorate treatment in AML [8].

Hunt et al. [9, 10] identified acyl-CoA thioesterases (ACOTs) for the first time in 1950. ACOTs are a superfamily of enzymes that catalyze the hydrolysis of fatty acyl-CoA to form free fatty acids (FFA) and coenzyme A (CoA). These enzymes play a vital role in lipid metabolism by maintaining the appropriate levels of FFA, fatty acyl-CoA, and CoA in cells [11]. These acyl-CoA thioesterases were revised and classified in 2005, and the enzymes in humans were designated as ACOTs represented by uppercase letters, while those in mice and rats were represented by lowercase letters as ACOTs [12]. So far, 12 kinds of human ACOTs (ACOT1, 2, 4, 6, 7–9, 11–15) [13, 14] and 15 kinds of mouse ACOTs (Acot115) [15] have been identified, and the acyl-CoA thioesterases family was divided into two different subgroups according to the diversity in sequence and structure, type I acyl-CoA thioesterases (ACOT1, 2, 4, 6; Acot1–6) and type II acyl-CoA thioesterases (ACOT7–9, 11–15; Acot7–15) [16]. The expression level of ACOT8 was significantly upregulated in clinical samples of hepatocellular carcinoma (HCC), resulting in lower cumulative survival. Thus, silencing the expression of ACOT8 can inhibit the growth of HCC cells in vitro [11]. Also, ACOT7 was identified as a biomarker that influences the prognosis in AML patients, and AHSCT could not overcome the unfavorable effect of ACOT7 in AML patients [17]. ACOTs represent a relatively understudied lipid metabolism family in leukemia, and comprehensive analyses remain insufficient. To improve the understanding of ACOTs, in this study, we analyzed the expression, prognostic value, and diagnostic value of ACOT in AML, and mined the clinical significance and function of ACOT2 whose high expression is predictive of a poor prognosis. In addition, Gene Ontology (GO) biological process enrichment analysis, Kyoto Encyclopedia of Genes and Genomes (KEGG), and Gene Set Enrichment Analysis (GSEA) of ACOT2 were also performed, moreover, the significance of ACOT2 and lipid metabolism was further analyzed by constructing protein-protein interaction (PPI) network and ACOT2-metabolite network. Our study may provide us with potential treatment targets to guide and support clinical strategy.

## 2. Materials and Methods

### 2.1. TNMplot Datebase

The expression level of the type I ACOT family including ACOT1, ACOT2, ACOT4, and ACOT6 were evaluated using the TNMplot platform (https://tnmplot.com/) [18], which is a web tool for the comparison of gene expression in normal, tumor, and metastatic tissues. It can supply data generated by either gene arrays or RNA-seq. Gene array data were manually selected from NCBI-GEO. RNA sequencing data were downloaded from The Cancer Genome Atlas (TCGA), Therapeutically Applicable Research to Generate Effective Treatments (TARGET), and Genotype-Tissue Expression (GTEx) repositories. TCGA and TARGET contain predominantly tumor and metastatic samples from adult and pediatric patients, while GTEx samples are from healthy tissues. Statistical significance was computed using Mann–Whitney or Kruskall–Wallis tests.

### 2.2. GEPIA Dataset

Gene expression profiling interactive analysis (GEPIA, https://gepia.cancer-pku.cn/) [19] is a newly developed interactive web server for analyzing the RNA sequencing expression data from the TCGA and GTEx projects based on a standard processing pipeline. GEPIA provides customizable functions such as tumor or normal differential expression analysis, profiling according to cancer types or pathological stages, patient survival analysis, similar gene detection, correlation analysis, and dimensionality reduction analysis.

### 2.3. CCLE Dataset

The expression of ACOT1, ACOT2, ACOT4, and ACOT6 originated from type I ACOT family in AML cell lines was verified by the Cancer Cell Line Encyclopedia (CCLE, https://www.broadinstitute.org/ccle) [20], which gathers extensive mRNA expression and mutation information from human cancer cell lines [21]. The CCLE project is a collaboration between the Broad Institute and the Novartis Institutes for Biomedical Research and its Genomics Institute of the Novartis Research Foundation to conduct a detailed genetic and pharmacologic characterization of a large panel of human cancer models, to develop integrated computational analyses that link distinct pharmacologic vulnerabilities to genomic patterns, and to translate cell line integrative genomics into cancer patient stratification. The CCLE database provides public access to genomic data, analysis, and visualization for about 1,000 cell lines.

### 2.4. Diagnosis and Prognosis Value of ACOT Family in AML

The diagnostic and prognostic values of ACOT1, ACOT2, ACOT4, and ACOT6 expression were evaluated in patients with AML based on bone marrow RNA-seq dataset data acquired from TCGA and GTEx databases. The samples were divided into high and low expression groups according to the median expression, and the effects of high and low expression on AML prognosis were analyzed. The overall survival (OS) curve was drawn by a Kaplan–Meier method. The “survival” package (version 3.2-10) was applied for statistical analysis of survival data, and “survminer” package (version 0.4.9) was used for visualization. The log-rank test was applied to evaluate the statistical significance of differences in survival duration (*P* < 0.05). The receiver operating characteristic (ROC) curves were generated to evaluate the diagnostic value. The area under the ROC curve (AUC) was used to summarize diagnostic accuracy, with 1.0 representing perfect discrimination and 0.5 representing chance discrimination.

### 2.5. Correlation between ACOT2 Expression and Clinical Characteristics of AML

To further evaluate the clinical significance of ACOT2 in AML, the association between ACOT2 expression and clinical parameters including patients' gender, age, bone marrow (BM) blasts, peripheral blood (PB) blasts, white blood cell (WBC) counts, cytogenetic risks, French-American-British (FAB) classifications, cytogenetics, FLT3 mutation, IDH1 R132 mutation, IDH1 R140 mutation, IDH1 R172 mutation, RAS mutation, and NPM1 mutation was analyzed based on logistic regression models. This analysis was conducted by taking ACOT2 as independent variables, and relevant clinical characteristics were set as dependent variables.

### 2.6. LinkedOmics Dataset

LinkedOmics (https://www.linkedomics.orglogin.php) [22] is a new and unique tool in the software ecosystem for disseminating data from large-scale cancer omics projects. It uses preprocessed and normalized data from the Broad TCGA Firehose and Clinical Proteomic Tumor Analysis (CPTAC) data portal to reduce redundant efforts, and focuses on the discovery and interpretation of attribute associations, and thus complements existing cancer data portals. The function module of LinkedOmics database was applied to explore the coexpressed genes of ACOT2. The Comparative Toxicogenomics Database (CTD, https://ctdbase.org/) [23] was used to obtain AML-related disease targets with “AML” as the search term. The coexpressed genes of ACOT2 dataset and the AML-related genes dataset were intersected to obtain the common targets between ACOT2 coexpressed and AML-related genes by using Venny 2.1 (https://bioinfogp.cnb.csic.es/tools/venny/index.html).

### 2.7. GO Annotation, KEGG Pathway Enrichment and GSEA Analysis

The GO resource provides a platform for functional annotation and enrichment analysis of genes. KEGG is a comprehensive database of biological information designed to assist in the interpretation of large-scale molecular datasets. In the present study, the top 30 core targets of functional protein association network were screened, and GO biological function enrichment analysis and KEGG metabolic process analysis were performed based on g:Profiler (https://biit.cs.ut.ee/gprofiler/gost) [24]. *P* < 0.05 was considered statistically significant for GO annotation enrichment analysis and KEGG pathway enrichment analysis.

In order to further understand the functional implications of ACOT2, the gene expression files of the 151 cases downloaded from TCGA were separated into high-express groups and low-express groups based on “DESeq2” package (version 1.26.0). In accordance with the differential analysis of high and low expression groups, we sorted the differentially expressed genes (DEG) according to log fold change, then constructed the ordered gene set and enriched it with GSEA based on “clusterProfiler” package (version 3.14.3). *P* < 0.05 was considered to be significantly enriched. The Hallmark list from the h.all.v7.2.symbols.GMT was used as the gene set reference.

### 2.8. Protein–Protein Interaction Network Construction

GeneMANIA (https://genemania.org) [25] is a flexible user-friendly website for generating hypotheses about gene function, analyzing gene lists, and prioritizing genes for functional assays. The PPI of common targets between ACOT2 coexpressed and AML-related genes were predicted based on GeneMANIA. The Cytoscape software (version 3.8.1) was employed for visualization.

### 2.9. Metabolite Interaction Network Construction

The Human Metabolome Database (https://hmdb.ca/) [26] was used to obtain ACOT2-related metabolites with “ACOT2” as the search term. The ACOT2-related metabolites interaction network was predicted based on Cytoscape software (https://www.cytoscape.org) (version 3.8.1). ACOT2-related metabolites were input into Cytoscape software to construct a functional ACOT2-related metabolites association network.

## 3. Results

### 3.1. Comparison of the Expression of Type I ACOT Family

**Figure 1:**
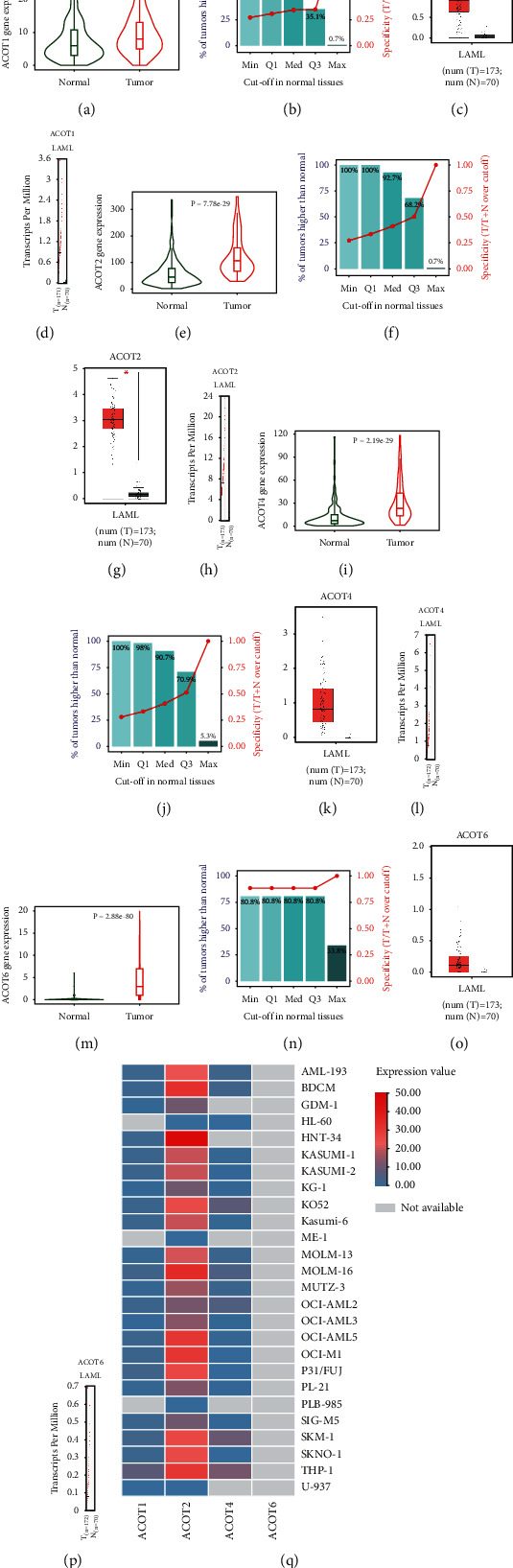
The expression level of type I ACOT family (i.e., ACOT1, ACOT2, ACOT4, and ACOT6) in normal, and AML gene array data. (a, e, i, m) the violin plots by TNMplot. (b, f, j, n) the bar charts by TNMplot. The bars represent the proportions of AML samples that showed high expression of the selected gene compared to normal samples at each of the quantile cutoff values (minimum, 1st quartile, median, 3rd quartile, maximum). Specificity was calculated by dividing the number of AML samples by the sum of tumor and normal samples below each given cutoff. In cases, where the fold change was over 1, those “over” were used instead of those “below.” (c, g, k, o) the box plot was analyzed by GEPIA database. (d, h, i, p) the scatter plot was analyzed by GEPIA database. (q) The expression of the type I ACOT family in 26 AML cell lines by CCLE database. ^*∗*^, *P* < 0.05.

The elevated expression of the type I ACOT (i.e., ACOT1, ACOT2, ACOT4, and ACOT6) in AML subjects was observed in the TNMplot database (all *P* < 0.05). The approach directly compared AML and normal samples by grouping all specimens of the same category, and the results were visualized by violin plots. The normal AML comparison was shown in Figures 1(a), 1(e), 1(i), and 1(m). We have also implemented a graphical representation of sensitivity and specificity. The diagram provided the percentage of AML samples that showed higher expression of the selected gene than that of the normal samples at each major cutoff value (Figures 1(b), 1(f), 1(j), and 1(n)). In GEPIA, ACOT2 was significantly higher upregulated in AML compared with that of normal samples (Figures 1(g) and 1(h)), yet there was no significant difference in ACOT1 (Figures 1(c) and 1(d)), ACOT4 (Figures 1(k) and 1(l)), and ACOT6 (Figures 1(o) and 1(p)) between AML and normal samples. The expressions of the type I ACOT (ACOT1, ACOT2, ACOT4, and ACOT6) family in 26 AML cell lines were obtained from the CCLE database. Moreover, the results also showed that ACOT2 was highly expressed in most 26 AML cell lines, especially in HNT-34, MOLM-16, BDCM, and THP-1 cell lines, whereas ACOT1 and ACOT4 were not expressed in almost any of the AML cell lines, and the expression of ACOT6 in 26 AML cell lines was not available (Figure 1(q)). However, the ACOT1, ACOT2, and ACOT4 were well expressed in THP-1 cells, and the high expression of ACOT2 was more significant.

### 3.2. Prognostic and Diagnostic Values of Type I ACOT in AML

**Figure 2:**
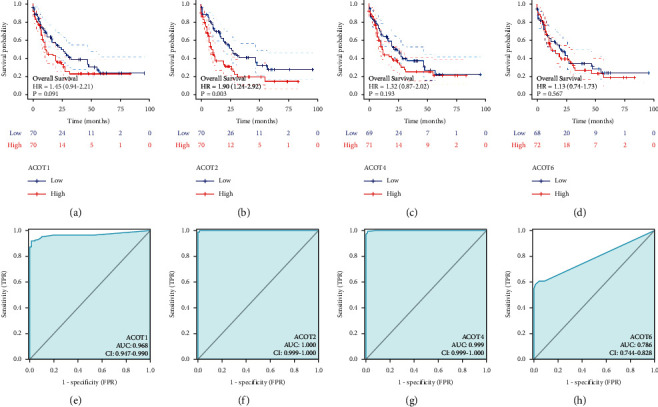
The effect of type I ACOT family (i.e., ACOT1, ACOT2, ACOT4, and ACOT6) on overall survival and ROC curve analysis in AML. (a, b, c, d) GEPIA analysis of ACOT1, ACOT2, ACOT4, and ACOT6; (e, f, g, h) ROC curve analysis of ACOT1, ACOT2, ACOT4, and ACOT6.

We investigated the prognostic values of type I ACOT (ACOT1, ACOT2, ACOT4, and ACOT6) in AML. One hundred and fifty-one AML samples and corresponding RNA-seq dataset were obtained from TCGA database, however, the information on survival time for 11 samples was missing. Consequently, 140 samples were included in the analysis. The cutoff value of median ACOT1, ACOT2, ACOT4, and ACOT6 expressions were 0.885577317, 2.972574801, 1.491169926, and 0.558179678, respectively. Patients were split into high- and low-expression groups. Results indicated that high expression of ACOT2 was correlated with a significantly poorer prognosis compared with those exhibiting low ACOT2 expression (hazard ratio (HR) = 1.90; *P*=0.003) (Figure 2(b)), whereas ACOT1 (hazard ratio (HR) = 1.45; *P*=0.091) (Figure 2(a)), ACOT4 (hazard ratio (HR) = 1.32; *P*=0.193) (Figure 2(c)), and ACOT6 (hazard ratio (HR) = 1.13; *P*=0.567) (Figure 2(d)) was not significantly associated with poor OS in AML. Moreover, receiver conducting feature curve analysis indicated that ACOT1 (AUC: 0.968) (Figure 2(e)), ACOT2 (AUC: 1.000) (Figure 2(f)), and ACOT4 (AUC: 0.999) (Figure 2(g)) exhibited excellent diagnostic efficiency for AML, and the diagnostic efficiency of ACOT6 (AUC: 0.786) (Figure 2(h)) on AML was general. Hence, high expressed ACOT2 is a valuable prognostic factor for AML.

### 3.3. Correlation between ACOT2 Expression and Clinical Characteristics of AML

**Figure 3:**
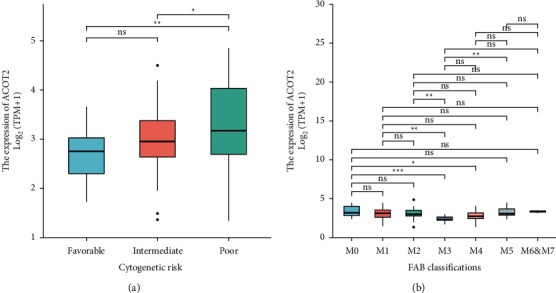
The expression level of ACOT2 in FAB classification and cytogenetic risk groups. (a) The expression level of ACOT2 in different cytogenetic risks. (b) The expression level of ACOT2 in different FAB classifications. Ns, *P* ≥ 0.05; ^*∗*^*P* < 0.05; ^∗∗^*P* < 0.01; ^∗∗∗^*P* < 0.001.

The correlation between ACOT2 expression levels and clinical characteristics, including age, gender, patients' race, WBC counts, BM blasts, PB blasts, cytogenetic risks, and FAB classification were analyzed, and the results suggested that ACOT2 did not differ significantly with most of these clinical characteristics in AML except for FAB classification and cytogenetics (Table 1). Compared to the favorable cytogenetic risk group, ACOT2 was higher expressed in intermediate and poor cytogenetic risk groups (Figure 3(a)). Also, ACOT2 expression was relatively low in M3 compared to that of M0, M1, M2, and M5 (Figure 3(b)). Additionally, we examined the association between ACOT2 expression and mutated genes in AML with FLT3, IDH1 R132, IDH1 R140, IDH1 R172, RAS, and NPM1 (Table 2), and there were no significant correlations between them.

### 3.4. Common Targets between ACOT2 Coexpressed and AML Related Genes

**Figure 4:**
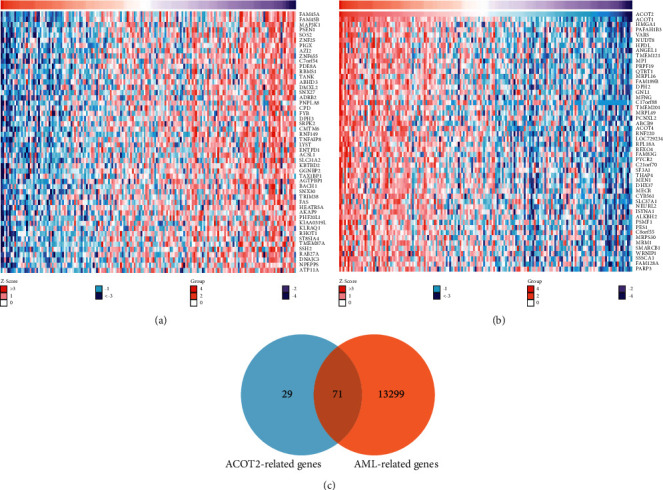
The top 50 significant genes positively or negatively correlated with ACOT2 and common targets between ACOT2 coexpressed and AML-related genes. (a) The top 50 significant genes were negatively correlated with ACOT2. (b) The top 50 significant genes were positively correlated with ACOT2. (c) Common genes between ACOT2 and AML expressed related genes. GO, KEGG, and GSEA enrichment analysis.

Next, the function module of LinkedOmics database was applied to explore the coexpressed genes of ACOT2 in AML. The top 50 significant genes either positively or negatively correlated with ACOT2 were screened out in the heatmap (Figures 4(a) and 4(b)). As shown in the heatmap, DENN domain-containing protein 10 (FAM45A), DENN domain-containing protein 10 B (FAM45 B), mitogen-activated protein kinase 1 (MAP3K1), presenilin-1 (PSEN1), son of sevenless homolog 2 (SOS2) were identified to be closely negatively correlated with ACOT2, and ACOT1, high mobility group protein HMG-I/HMG-Y (HMGA1), platelet-activating factor acetylhydrolase IB subunit alpha1 (PAFAH1B3), valine-tRNA ligase (VARS), mitochondrial coenzyme a diphosphatase NUDT8 (NUDT8) were identified to be closely positively correlated with ACOT2. This result indicated a widespread effect of ACOT2 on the transcriptome of AML. We retrieved 13299 AML-related genes from CTD, which have been listed in Supplement Table 1. In addition, there were 71 common genes between ACOT2 coexpressed related genes and AML-related genes (Figure 4(c)).

**Figure 5:**
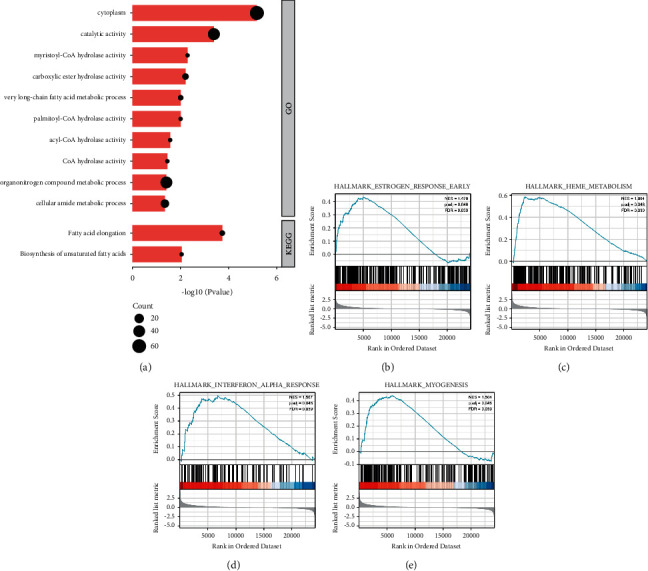
GO, KEGG, and GSEA enrichment analyses of common targets between ACOT2 coexpressed and AML-related genes. (a) GO and KEGG enrichment analysis and (b) GSEA enrichment analysis.

Functional enrichment analysis using Gene Ontology (GO) biological process enrichment analysis of 71 common targets between ACOT2 coexpressed and AML-related genes indicated that the processes were involved in the catalytic activity, myristoyl-CoA hydrolase activity, carboxylic ester hydrolase activity, palmitoyl-CoA hydrolase activity, acyl-CoA hydrolase activity, and CoA hydrolase activity at the MF levels, cytoplasm at the CC levels, and very long-chain fatty acid metabolic process, organonitrogen compound metabolic process, cellular amide metabolic process at the BP levels in AML (Figure 5(a)). The KEGG pathway enrichment analysis of 71 common targets screened by the Venn diagram (Figure 4(c)) showed that the most enriched pathways involved in fatty acid elongation and biosynthesis of unsaturated fatty acids (Figure 5(a)). To gain further biological insights into the underlying mechanisms of ACOT2 overexpression in AML, GSEA analysis revealed that the gene sets were significantly enriched in estrogen response early (NES = 1.479, *P*=0.046) (Figure 5(b)), heme metabolism (NES = 1.994, *P*=0.046) (Figure 5(c)), interferon alpha response (NES = 1.567, *P*=0.039) (Figure 5(d)), myogenesis (NES = 1.504, *P*=0.046) (Figure 5(e)). These findings revealed that high ACOT2 expression was related to the deterioration of OS in patients with AML, which is mainly related to abnormal lipid metabolism, suggesting that ACOT2 may be a potential target for the treatment of AML.

### 3.5. PPI Network

**Figure 6:**
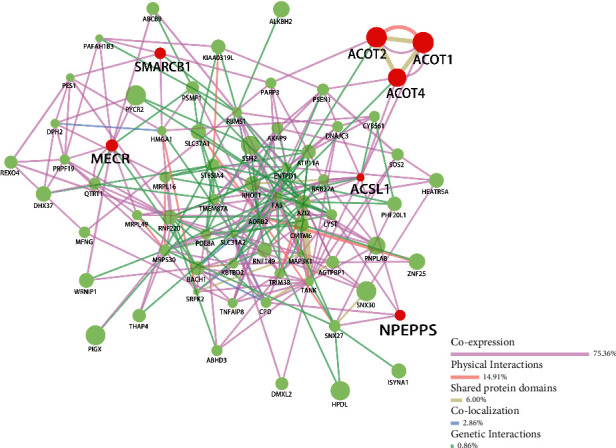
The PPI annotation of coexpressed genes related to ACOT2 in AML analyzed by GeneMANIA.

The 71 common targets between ACOT2 coexpressed and AML-related genes were uploaded to the GeneMANIA for the protein-protein interactions of common genes analysis (Figure 6). Among them, coexpression accounted for 75.36%, physical interactions accounted for 14.91%, shared protein domains accounted for 6.00%, colocalization accounted for 2.86%, and genetic interactions accounted for 0.86%. The results showed that ACOT2, ACOT1, ACOT4, enoyl-acyl carrier protein reductase, mitochondrial (MECR), puromycin-sensitive aminopeptidase (NPEPPS), SWI/SNF-related matrix-associated actin-dependent regulator of chromatin subfamily B member 1 (SMARCB1), and long-chain fatty acid-CoA ligase 1 (ACSL1) played a considerable role in the PPI network, which were related to lipid metabolism. Among them, NPEPPS was intersection of the catalytic activity, organonitrogen compound metabolic process, and cellular amide metabolic process, MECR was intersection of the catalytic activity, cytoplasm, and fatty acid elongation, SMARCB1 was intersection of organonitrogen compound metabolic process, ACSL1 was intersection of the catalytic activity, very long-chain fatty acid metabolic process, organonitrogen compound metabolic process, and cellular amide metabolic process, and ACOT2, ACOT1, and ACOT4 were involved in all cellular component, biological process, molecular function, and pathways.

### 3.6. ACOT2 and Its Metabolites Interaction Network

**Figure 7:**
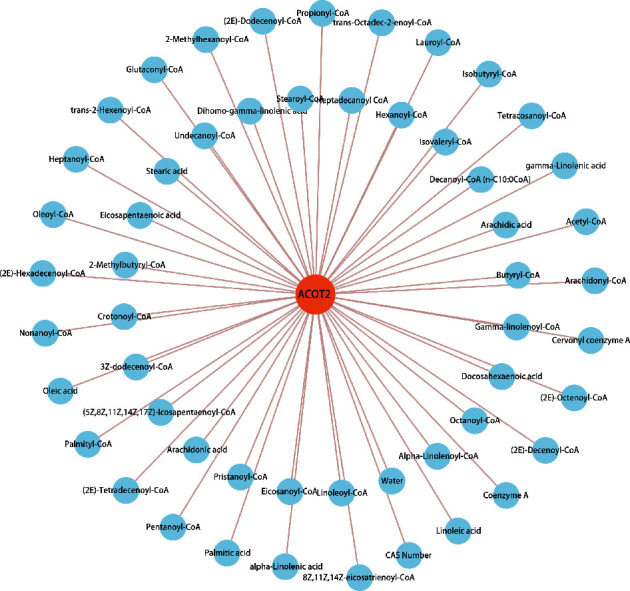
Construction of ACOT2-related metabolites interaction network by Cytoscape software.

In the present study, we obtained 53 ACOT2-related metabolites based on Human Metabolome Database (Supplement Table 2). Acyl-CoA thioesterases are a group of enzymes that catalyze the hydrolysis of acyl-CoAs to the free fatty acid and coenzyme A (CoASH), providing the potential to regulate intracellular levels of acyl-CoAs, free fatty acids, and CoASH. The results also revealed high levels of activity on medium and long-chain acyl CoAs. The general function of ACOT2 was involved in thiol ester hydrolase activity. The 53 ACOT2-related metabolites were input into the Cytoscape software for the protein-metabolite interaction network analysis (Figure 7). Among them, ACOT2-related metabolites were related to metabolites such as oleic acid, palmitic acid, linoleic acid, stearic acid, and arachidonic acid, and also were closely related to coenzyme A such as linoleoyl-CoA, octanoyl-CoA, butyryl-CoA, isovaleryl-CoA, stearoyl-CoA, acetyl-CoA, isobutyryl-CoA, propionyl-CoA, glutaconyl-CoA, and oleoyl-CoA. These metabolites were closely related to lipid metabolism, suggesting that ACOT2 plays a significant role in lipid metabolism, such as fatty acid elongation and biosynthesis of unsaturated fatty acids.

## 4. Discussion

AML is acute leukemia characterized by rapid proliferation and loss of differentiation of myeloid stem or progenitor cells, which is highly genetically heterogeneous and is most common in adults, accounting for about 25∼35% of all leukemia [27–31]. The harm of AML to patients is devastating, and the latest progress in treatment is still limited. Identifying the genomic heterogeneity of AML and highlighting the preprocessing characteristics of diagnosed AML are particularly pivotal for selecting the appropriate targeted and combined treatment methods to achieve a more satisfactory curative effect [32]. Metabolic reprogramming can accommodate the biosynthetic needs of tumor cells for rapid proliferation [33]. Tumor-related metabolic reprogramming can promote changes in intracellular and extracellular metabolites, thereby affecting the expression of related genes, the level of cell differentiation, and tumor microenvironment, so as to maintain the growth state of tumor [34]. Hanahan et al. [33–35] found that tumor cells can maximize the efficiency of metabolic enzyme activity by changing their metabolic pathway to adapt to poor angiogenesis in the tumor microenvironment, thereby maintaining the ability of tumor cells to proliferate indefinitely.

Acetyl-CoA is the product of various metabolic reactions in the metabolic process. It is also a lipid biosynthetic precursor that supports cell growth and proliferation, and thus regulates various cellular processes including epigenetics [36–40]. The level of lipid biosynthesis is significantly increased in many types of cancers such as prostate cancer, lung cancer, and gastric cancer [41, 42]. Oxidative phosphorylation (OXPHOS) inhibitors can further inhibit cell metabolism by inhibiting the fatty acid synthesis and other metabolic pathways to exert their anticancer roles, so, inhibiting these metabolic pathways can synergistically inhibit cancer growth [43–45]. Abnormal lipid metabolism is closely associated with tumor cell proliferation, which has prognostic significance in cancer progression. As a regulator of lipid metabolism, TPD52 can regulate the formation of lipid droplets and fatty acid storage. Abnormal sphingolipid metabolism and fatty acid oxidation in AML indicated that TPD52 may be a potential tumor marker with prognostic value in AML [46, 47]. The occurrence of metabolic reprogramming in the clonal evolution of AML can promote cell survival and drug resistance. Recent studies have shown that lipid metabolism and other metabolic abnormalities occur in AML mice, and the expression of endothelial lipase (LIPG) was increased in leukemia cells of AML mice, LIPG might be a potential target for the abnormal lipid metabolism in AML [48].

Humans contain abundant ACOT enzymes [12, 16, 49–51], and ACOTs are also known as acyl-CoA thioesterase, acyl-CoA thiolase, and deacylase [13]. A series of enzymes composed of ACOTs can exhibit a significant function in lipid metabolism by maintaining cell level and an appropriate proportion of free and activated fatty acids and CoASH [52, 53]. *β*-Oxidation is a process in which fatty acids are degraded in mitochondria and peroxisomes to provide energy for the physiological activities of the cells. ACOTs are one of the pivotal enzymes of the *β*-oxidation system, which can scavenge short-chain products that are detrimental to the organism from *β*-oxidation. In addition, Adams et al. [54] showed that ACOTs may also act as ligands for some transcription factors and participate in cellular systems and functions related to signal transduction, intracellular transport, vesicle budding, and endocytosis. The activity of ACOTs mainly comes from a broader class of thioester hydrolases [9]. Thioester hydrolases can cleave thioester bonds between sulfur atoms and carbonyl groups, while most ACOTs act exclusively on molecular substrates containing CoA [55]. There are two different subgroups of type I ACOT and type II ACOT in the ACOT family. Among them, type I ACOTs in humans include ACOT1, ACOT2, ACOT4, and ACOT6. Both ACOT1 and ACOT2 can effectively hydrolyze a variety of 2-APA-CoA substrates. ACOT1 can hydrolyze 2-APA-CoA esters outputted from mitochondria and peroxisomes, thereby suppressing cyclo-oxygenase (COX) -1 and -2 in the endoplasmic reticulum, while ACOT2 can regulate 2-APA toxicity and mitochondrial free CoA levels [56]. St Clair et al. [57] found that ACOT, as a rheostat controlling activated fatty acid, has a regulatory effect on the replication of dengue virus serotype 2 (DENV2) and the release of infectious particles. The single knockout of ACOT2 significantly reduced DENV2 genome replication, protein translation, and infectious virus release. In the process of biosynthesis of the unsaturated fatty acid signaling pathway, ACOT2 can hydrolyze LACoA into free fatty acids and CoA, and elevated SCD1 can cause the excessive synthesis of fatty acids. Excess fatty acids and the inhibition of ACOT2 aggravate the accumulation of LACoA, which leads to the accumulation of metabolic wastes [58–60]. The level of miR-27b in nonalcoholic fatty liver disease (NAFLD) is significantly increased, and increased miR-27b-3p can promote adipocyte differentiation by inducing ACOT2 expression, and knockdown of ACOT2 expression can suppress lipid accumulation and adipocyte differentiation [61].

So far, metabolic abnormalities have been observed in many tumor diseases, including AML. Nevertheless, there are few cases of specific metabolic enzyme-related gene changes that have been investigated, and the expression and clinical significance of ACOT2 in AML have not been revealed. Here, our research reported that the upregulated expression of ACOT2 and abnormal lipid metabolism were closely related to the clinical prognosis of AML, suggesting that ACOT2-dependent abnormal lipid metabolism may become a prospective target to evaluate the clinical significance of AML diseases. According to the data of TNMplot and GEPIA database, type I ACOT (i.e., ACOT1, ACOT2, ACOT4, and ACOT6) was highly expressed in AML (all *P* < 0.05). Our further research showed that the expression of ACOT2 was significantly upregulated in most 26 AML cell lines, especially in HNT-34, MOLM-16, BDCM, and THP-1 cell lines. However, there was no significant increase in ACOT1 and ACOT4 in almost any of the AML cell lines, and the expression of ACOT6 in 26 AML cell lines was not available. Survival analysis indicated that the increased level of ACOT2 (*P*=0.003) was significantly associated with poor OS in AML, whereas ACOT1 (*P*=0.091), ACOT4 (*P*=0.193), and ACOT6 (*P*=0.567) were not significantly correlated. Moreover, the receiver conducting feature curve analysis indicated that ACOT2 (AUC: 1.000) was a prediction of AML and exhibited excellent diagnostic efficiency for AML. Hence, highly expressed ACOT2 is a valuable prognostic factor for AML, indicating that inhibiting the expression of ACOT2 may be a potential approach to prevent the development of AML.

Then, we further studied the correlation between ACOT2 expression and clinical characteristics of AML, and the results suggested that ACOT2 did not differ significantly with most of these clinical characteristics in AML, except for FAB classification and cytogenetics. Compared to the favorable cytogenetic risk group, ACOT2 was highly expressed in intermediate and poor cytogenetic risk groups. Additionally, we examined the association between ACOT2 expression and mutated genes in AML with FLT3, IDH1 R132, IDH1 R140, IDH1 R172, RAS, and NPM1, but no significant correlations were found.

Next, the function module of LinkedOmics database was applied to explore the coexpressed genes of ACOT2 in AML, and the top 50 significant genes positively or negatively correlated with ACOT2 were screened out. GO, KEGG, and GSEA analysis of 71 common targets between ACOT2 coexpressed and AML-related genes revealed that the involved processes were the catalytic activity, palmitoyl-CoA hydrolase activity, acyl-CoA hydrolase activity, and CoA hydrolase activity, and very long-chain fatty acid metabolic process, organonitrogen compound metabolic process, cellular amide metabolic process, the most enriched pathways included fatty acid elongation and biosynthesis of unsaturated fatty acids, and the gene sets were significantly enriched in estrogen response early, heme metabolism, interferon alpha response, myogenesis. The PPI network results showed that ACOT2, ACOT1, ACOT4, MECR, NPEPPS, SMARCB1, and ACSL1 played a considerable role in the PPI network, which is related to lipid metabolism. Besides, the ACOT2 and its metabolites interaction network indicated that these metabolites are closely related to lipid metabolism, suggesting that ACOT2 plays significant roles in lipid metabolism involved in fatty acid elongation and biosynthesis of unsaturated fatty acids. Although the metabolites interaction network has greatly expanded the interaction relationships of multiple metabolites, there are still many members whose functions are relatively unexplored and deserve further study.

## 5. Conclusions

In this study, we explored the mechanism by which high expression of ACOT2 predicts decreased overall survival and abnormal lipid metabolism during AML disease progression. The expression of ACOT2 was significantly upregulated in AML cell lines, which had good diagnostic performance and was significantly associated with poor OS in AML. Besides, ACOT2 plays a significant role in lipid metabolism such as fatty acid elongation and biosynthesis of unsaturated fatty acids. In conclusion, the high expression of ACOT2 may be an important characteristic of decreased overall survival and abnormal lipid metabolism in AML, and the increase of ACOT2 in AML can be used as a potential biomarker for auxiliary diagnosis and prognosis, and targeted ACOT2 may be a promising approach to inhibit AML progression.

## Figures and Tables

**Table 1:** Correlation between ACOT2 expression levels in AML and clinical characteristics.

Characteristic	Low expression of ACOT2	High expression of ACOT2	*P*
*n*	75	76	
Gender, *n* (%)			0.122
Female	39 (25.8%)	29 (19.2%)	
Male	36 (23.8%)	47 (31.1%)	
Race, *n* (%)			1.000

Asian	1 (0.7%)	0 (0%)	
Black or African American	6 (4%)	7 (4.7%)	
White	67 (45%)	68 (45.6%)	
Age, *n* (%)			0.554

≤60	46 (30.5%)	42 (27.8%)	
>60	29 (19.2%)	34 (22.5%)	

WBC count (10^9/L), *n* (%)			1.000
≤20	38 (25.3%)	39 (26%)	
>20	36 (24%)	37 (24.7%)	

BM blasts (%), *n* (%)			0.058
≤20	36 (23.8%)	24 (15.9%)	
>20	39 (25.8%)	52 (34.4%)	

PB blasts (%), *n* (%)			0.932
≤70	35 (23.2%)	37 (24.5%)	
>70	40 (26.5%)	39 (25.8%)	
Cytogenetic risk, *n* (%)			0.036

Favorable	20 (13.4%)	11 (7.4%)	
Intermediate	42 (28.2%)	40 (26.8%)	
Poor	12 (8.1%)	24 (16.1%)	

FAB classifications, *n* (%)			<0.001
M0	4 (2.7%)	11 (7.3%)	
M1	14 (9.3%)	21 (14%)	
M2	18 (12%)	20 (13.3%)	
M3	15 (10%)	0 (0%)	
M4	18 (12%)	11 (7.3%)	
M5	6 (4%)	9 (6%)	
M6	0 (0%)	2 (1.3%)	
M7	0 (0%)	1 (0.7%)	

**Table 2:** Correlation between ACOT2 expression levels in AML and mutated genes.

Characteristics	Low expression of ACOT2	High expression of ACOT2	*P*
FLT3 mutation, *n* (%)			0.259
Negative	47 (32%)	55 (37.4%)	
Positive	26 (17.7%)	19 (12.9%)	

IDH1 R132 mutation, *n* (%)			0.077
Negative	64 (43%)	72 (48.3%)	
Positive	10 (6.7%)	3 (2%)	

IDH1 R140 mutation, *n* (%)			0.782
Negative	68 (45.6%)	69 (46.3%)	
Positive	7 (4.7%)	5 (3.4%)	

IDH1 R172 mutation, *n* (%)			0.497
Negative	73 (49%)	74 (49.7%)	
Positive	2 (1.3%)	0 (0%)	

RAS mutation, *n* (%)			0.276
Negative	69 (46%)	73 (48.7%)	
Positive	6 (4%)	2 (1.3%)	

NPM1 mutation, *n* (%)			0.115
Negative	54 (36%)	63 (42%)	
Positive	21 (14%)	12 (8%)	

## Data Availability

All the data are included in the main text.

## Supplementary Materials

Supplementary Materials Supplement Table 1. The 13299 AML-related genes from CTD. Supplement Table 2. The 53 ACOT2-related metabolites are based on Human Metabolome Database.
