# Supplementary material for: High Expression of ACOT2 Predicts Worse Overall Survival and Abnormal Lipid Metabolism: A Potential Target for Acute Myeloid Leukemia
Source: J Healthc Eng. 2022 Sep 23;2022:2669114. doi: 10.1155/2022/2669114 (PMC9525752; doi:10.1155/2022/2669114)
Supplement: Supplementary Materials — Supplement Table 1. The 13299 AML-related genes from CTD. Supplement Table 2. The 53 ACOT2-related metabolites are based on Human Metabolome Database. [file 2669114.f1.zip › Supplement Table1.pdf]

Gene Sym	Gene ID	Disease N	Disease ID	Direct Evic	Inference	Inference	Reference Count
CDKN1A	1026	Leukemia,	MESH:D015470		15-deoxy-	106.51	166
PARP1	142	Leukemia,	MESH:D015470		15-deoxy-	104.18	165
CASP3	836	Leukemia,	MESH:D015470		15-deoxy-	99.67	173
H2AX	3014	Leukemia,	MESH:D015470		ABT-737|a	96.99	150
CCNB1	891	Leukemia,	MESH:D015470		15-deoxy-	93.82	86
CASP8	841	Leukemia,	MESH:D015470		15-deoxy-	93.02	153
TP53	7157	Leukemia,	MESH:D015470		Air Polluta	92.63	163
AKT1	207	Leukemia,	MESH:D015470		15-deoxy-	91.95	113
JUN	3725	Leukemia,	MESH:D015470		15-deoxy-	91.33	140
MAPK3	5595	Leukemia,	MESH:D015470		15-deoxy-	88.3	96
MAPK1	5594	Leukemia,	MESH:D015470		15-deoxy-	85.5	96
RB1	5925	Leukemia,	MESH:D015470		15-deoxy-	85	101
XIAP	331	Leukemia,	MESH:D015470		15-deoxy-	82.64	115
TNFRSF10	8795	Leukemia,	MESH:D015470		Alitretinoi	81.91	126
MCL1	4170	Leukemia,	MESH:D015470		2-(2-chlor	81.9	107
BCL2L1	598	Leukemia,	MESH:D015470		ABT-737|a	78.88	136
CDKN1B	1027	Leukemia,	MESH:D015470		15-deoxy-	78.6	100
TOP2A	7153	Leukemia,	MESH:D015470		Amsacrine	77.91	101
FAS	355	Leukemia,	MESH:D01	marker/m	15-deoxy-	77.74	132
CCNA2	890	Leukemia,	MESH:D015470		15-deoxy-	76.57	59
MKI67	4288	Leukemia,	MESH:D015470		2-(2-chlor	76.57	70
BAX	581	Leukemia,	MESH:D015470		ABT-737|A	75.67	158
CCNE1	898	Leukemia,	MESH:D015470		15-deoxy-	75.34	83
ABCB1	5243	Leukemia,	MESH:D015470		Arsenic Tr	74.52	145
BCL2	596	Leukemia,	MESH:D01	marker/m	ABT-737|A	74.47	159
CASP7	840	Leukemia,	MESH:D01	marker/m	ABT-737|A	73.34	74
KIT	3815	Leukemia,	MESH:D01	marker/m	alvocidib|A	72.81	71
TNFSF10	8743	Leukemia,	MESH:D01	therapeuti	Air Polluta	72.39	124
IFNG	3458	Leukemia,	MESH:D015470		15-deoxy-	72.09	77
CASP9	842	Leukemia,	MESH:D015470		15-deoxy-	71.89	116
IL6	3569	Leukemia,	MESH:D015470		15-deoxy-	71.57	155
CDK2	1017	Leukemia,	MESH:D015470		15-deoxy-	71.55	77
VEGFA	7422	Leukemia,	MESH:D015470		Air Polluta	70.91	106
BIRC5	332	Leukemia,	MESH:D015470		alvocidib|A	69.24	86
CASP8	841	Leukemia,	MESH:D015473		Aclarubici	68.66	280
BIRC3	330	Leukemia,	MESH:D015470		15-deoxy-	68.65	75
SERPINE1	5054	Leukemia,	MESH:D015470		15-deoxy-	68.59	87
MMP9	4318	Leukemia,	MESH:D015470		15-deoxy-	68.36	75
TNF	7124	Leukemia,	MESH:D015470		15-deoxy-	68.28	159
BAX	581	Leukemia,	MESH:D015473		4'-methox	67.94	282
PTGS2	5743	Leukemia,	MESH:D015470		15-deoxy-	67.83	157
GADD45A	1647	Leukemia,	MESH:D015470		Air Polluta	67.71	137
VDR	7421	Leukemia,	MESH:D015470		Alitretinoi	66.9	137
MYC	4609	Leukemia,	MESH:D01	marker/m	Amsacrine	66.66	100
TXNIP	10628	Leukemia,	MESH:D015470		Allopurino	66.64	75
CCND1	595	Leukemia,	MESH:D015470		alvocidib|A	66.04	84
MDM2	4193	Leukemia,	MESH:D015470		alvocidib|A	65.44	85
CXCL8	3576	Leukemia,	MESH:D015470		15-deoxy-	65.43	106
PRC1	9055	Leukemia,	MESH:D015470		Air Polluta	65.43	118
TYMS	7298	Leukemia,	MESH:D015470		Arsenic Tr	65.21	123
CCND3	896	Leukemia,	MESH:D015470		15-deoxy-	64.88	126
RPS6KB1	6198	Leukemia,	MESH:D015470		15-deoxy-	64.84	75
NFKBIA	4792	Leukemia,	MESH:D015470		15-deoxy-	64.81	80
SFN	2810	Leukemia,	MESH:D015470		Arsenic Tr	64.74	119
GADD45B	4616	Leukemia,	MESH:D015470		Air Polluta	64.55	61
EGFR	1956	Leukemia,	MESH:D015470		2-(2-chlor	64.54	74
RELA	5970	Leukemia,	MESH:D015470		15-deoxy-	64.18	151

CCND2	894	Leukemia, MESH:D01	marker/m	Arsenic Tr	63.82	50
CASP3	836	Leukemia, MESH:D015473		2-(2-chlor	63.82	284
F3	2152	Leukemia, MESH:D015470		Air Polluta	63.72	85
GPX3	2878	Leukemia, MESH:D015470		Androgen	63.55	63
TNFRSF10	8797	Leukemia, MESH:D015470		Alitretinoi	63.05	70
ERBB2	2064	Leukemia, MESH:D015470		Bortezomi	63.02	53
INSIG1	3638	Leukemia, MESH:D015470		Air Polluta	62.93	67
PTEN	5728	Leukemia, MESH:D015470		15-deoxy-	62.92	139
BCL2L11	10018	Leukemia, MESH:D015470		ABT-737|A	62.76	46
CDKN1A	1026	Leukemia, MESH:D015473		alpha-Toc	62.69	283
BCL2	596	Leukemia, MESH:D015473		4'-methox	62.42	285
CDK4	1019	Leukemia, MESH:D015470		Air Polluta	61.94	46
RAD51	5888	Leukemia, MESH:D015470		Amsacrine	61.72	126
CDK1	983	Leukemia, MESH:D015470		alvocidib|A	61.57	66
PARP1	142	Leukemia, MESH:D015473		4'-methox	61.47	280
TGFB1	7040	Leukemia, MESH:D015470		15-deoxy-	61.43	94
COL1A1	1277	Leukemia, MESH:D015470		15-deoxy-	61.21	142
CFLAR	8837	Leukemia, MESH:D015470		15-deoxy-	60.61	56
CASP9	842	Leukemia, MESH:D015473		2-(2-chlor	60.41	284
IFITM1	8519	Leukemia, MESH:D015470		Air Polluta	59.99	68
CD274	29126	Leukemia, MESH:D015470		Arsenic Tr	59.85	45
DDIT3	1649	Leukemia, MESH:D015470		2-(2-chlor	59.71	72
GDF15	9518	Leukemia, MESH:D015470		Alitretinoi	59.7	85
ABCA1	19	Leukemia, MESH:D015470		Alitretinoi	59.47	57
CDKN2A	1029	Leukemia, MESH:D015470		Air Polluta	59.4	93
MAPK14	1432	Leukemia, MESH:D015470		Air Polluta	59.36	56
FAS	355	Leukemia, MESH:D015473		4'-methox	59.31	280
PMAIP1	5366	Leukemia, MESH:D015470		ABT-737|A	59.29	69
GSTM1	2944	Leukemia, MESH:D015470		15-deoxy-	59.23	134
STAT1	6772	Leukemia, MESH:D015470		15-deoxy-	59.09	75
CENPF	1063	Leukemia, MESH:D015470		Azacitidin	58.98	49
NFKB1	4790	Leukemia, MESH:D015470		15-deoxy-	58.9	70
IGFBP3	3486	Leukemia, MESH:D015470		Alitretinoi	58.89	125
HIF1A	3091	Leukemia, MESH:D015470		15-deoxy-	58.86	141
CD36	948	Leukemia, MESH:D015470		Alitretinoi	58.83	63
RRM2	6241	Leukemia, MESH:D015470		Alitretinoi	58.83	105
ABCG2	9429	Leukemia, MESH:D015470		Allopurino	58.68	87
FOXO1	2308	Leukemia, MESH:D01	marker/m	6,7-dimet	58.65	56
MGMT	4255	Leukemia, MESH:D015470		Air Polluta	58.56	52
CDK6	1021	Leukemia, MESH:D01	marker/m	Arsenic Tr	58.47	59
IL1B	3553	Leukemia, MESH:D015470		15-deoxy-	58.1	86
CYP3A4	1576	Leukemia, MESH:D015470		2-(2-chlor	58.06	58
STAT3	6774	Leukemia, MESH:D01	marker/m	15-deoxy-	57.98	53
IL4	3565	Leukemia, MESH:D015470		15-deoxy-	57.56	68
BID	637	Leukemia, MESH:D015470		ABT-737|a	57.48	121
SERPINB2	5055	Leukemia, MESH:D015470		Air Polluta	57.33	55
NQO1	1728	Leukemia, MESH:D015470		2-(2-chlor	57.27	105
CXCR4	7852	Leukemia, MESH:D015470		Arsenic Tr	56.76	133
CDH1	999	Leukemia, MESH:D015470		2-(2-chlor	56.56	52
NAMPT	10135	Leukemia, MESH:D015470		Air Polluta	56.45	61
ABCC1	4363	Leukemia, MESH:D015470		15-deoxy-	56.44	130
IGFBP5	3488	Leukemia, MESH:D015470		Androgen	56.35	108
AR	367	Leukemia, MESH:D015470		Amsacrine	56.15	116
TFAP2A	7020	Leukemia, MESH:D015470		Calcitriol|C	56.02	105
BECN1	8678	Leukemia, MESH:D015470		ABT-737|A	55.79	46
CDKN2B	1030	Leukemia, MESH:D015470		Arsenic Tr	55.78	81
KDR	3791	Leukemia, MESH:D015470		Arsenic Tr	55.64	38
TP53	7157	Leukemia, MESH:D015473		alpha-Toc	55.29	280

CCN2	1490	Leukemia, MESH:D015470		15-deoxy-	55.25	58
PMP22	5376	Leukemia, MESH:D015470		Cytarabine	55.23	102
XRCC5	7520	Leukemia, MESH:D015470		Arsenic Tr	55.18	62
ICAM1	3383	Leukemia, MESH:D015470		15-deoxy-	54.94	79
ABCC3	8714	Leukemia, MESH:D015470		15-deoxy-	54.88	53
VCAN	1462	Leukemia, MESH:D015470		Benzene|B	54.72	64
PLAU	5328	Leukemia, MESH:D015470		Arsenic Tr	54.36	54
BBC3	27113	Leukemia, MESH:D015470		Arsenic Tr	54.31	107
BCL2L1	598	Leukemia, MESH:D015473		alpha-Toc	54.31	277
MCM5	4174	Leukemia, MESH:D015470		Arsenic Tr	54.24	41
ATF3	467	Leukemia, MESH:D015470		Arsenic Tr	54.11	124
BTG2	7832	Leukemia, MESH:D015470		alvocidib|A	54.07	118
VCAM1	7412	Leukemia, MESH:D015470		Arsenic Tr	53.9	137
RELA	5970	Leukemia, MESH:D015473		4'-methox	53.87	283
MAPK14	1432	Leukemia, MESH:D015473		alpha-Toc	53.82	274
BAK1	578	Leukemia, MESH:D015470		Arsenic Tr	53.67	56
SGK1	6446	Leukemia, MESH:D01	marker/m	Arsenic Tr	53.51	46
CDC25B	994	Leukemia, MESH:D015470		Arsenic Tr	53.51	43
PPARG	5468	Leukemia, MESH:D015470		15-deoxy-	53.41	149
TNFRSF10	8795	Leukemia, MESH:D015473		alpha-Toc	53.33	275
THBS1	7057	Leukemia, MESH:D015470		Air Polluta	53.31	107
IL2	3558	Leukemia, MESH:D015470		Alitretinoi	53.27	68
IL12B	3593	Leukemia, MESH:D015470		Alitretinoi	53.21	53
ABL1	25	Leukemia, MESH:D015470		alvocidib|A	53.17	57
PCNA	5111	Leukemia, MESH:D015470		alvocidib|A	53.02	70
ABCC2	1244	Leukemia, MESH:D015470		Alkylating	52.7	83
SP1	6667	Leukemia, MESH:D015470		6,7-dimet	52.62	64
ATM	472	Leukemia, MESH:D015470		Arsenic Tr	52.44	84
EFEMP1	2202	Leukemia, MESH:D015470		Anthracyc	52.38	39
SNAI2	6591	Leukemia, MESH:D015470		Arsenic Tr	52.33	119
STAT5A	6776	Leukemia, MESH:D015470		Arsenic Tr	52.25	36
E2F1	1869	Leukemia, MESH:D015470		alvocidib|A	52.2	53
IL10	3586	Leukemia, MESH:D015470		Air Polluta	52.11	67
DCN	1634	Leukemia, MESH:D015470		Arsenic Tr	52.04	110
ACTA2	59	Leukemia, MESH:D015470		Alitretinoi	51.87	62
PIM1	5292	Leukemia, MESH:D015470		Arsenic Tr	51.83	56
LUM	4060	Leukemia, MESH:D015470		Calcitriol|C	51.75	96
HMOX1	3162	Leukemia, MESH:D015470		15-deoxy-	51.71	106
ABCC1	4363	Leukemia, MESH:D015473		Antimony	51.66	194
IGF1R	3480	Leukemia, MESH:D015470		Air Polluta	51.49	54
IRF7	3665	Leukemia, MESH:D015470		Arsenic Tr	51.2	62
BIRC5	332	Leukemia, MESH:D01	marker/m	alvocidib|A	51.19	277
MMP2	4313	Leukemia, MESH:D015470		Androgen	51.14	68
NR4A2	4929	Leukemia, MESH:D015470		Air Polluta	51.13	59
BAD	572	Leukemia, MESH:D015470		3-(2-(3-(1	51.04	52
CCNG1	900	Leukemia, MESH:D015470		Arsenic Tr	50.78	117
PDGFRB	5159	Leukemia, MESH:D015470		6,7-dimet	50.69	104
PPP1R15A	23645	Leukemia, MESH:D015470		Arsenic Tr	50.68	74
PECAM1	5175	Leukemia, MESH:D015470		2-(2-chlor	50.66	39
MCM7	4176	Leukemia, MESH:D015470		Arsenic Tr	50.54	46
PTTG1	9232	Leukemia, MESH:D015470		Arsenic Tr	50.49	57
UBE2C	11065	Leukemia, MESH:D015470		Amsacrine	50.49	106
IFI6	2537	Leukemia, MESH:D015470		Arsenic Tr	50.3	51
NFE2L2	4780	Leukemia, MESH:D015470		15-deoxy-	50.28	64
KRT8	3856	Leukemia, MESH:D015470		Alitretinoi	50.26	118
CLU	1191	Leukemia, MESH:D015470		Arsenic Tr	50.24	65
FN1	2335	Leukemia, MESH:D015470		Alitretinoi	50.24	45
EGR1	1958	Leukemia, MESH:D015470		15-deoxy-	50.14	71

TPI1	7167	Leukemia, MESH:D015473		Antimony	50.08	269
BIK	638	Leukemia, MESH:D015470		Arsenic Tr	50.05	48
CASP1	834	Leukemia, MESH:D015470		Aclarubicin	49.85	53
TNFSF10	8743	Leukemia, MESH:D015473		alvocidib|/	49.84	279
PDK1	5163	Leukemia, MESH:D015470		Arsenic Tr	49.81	45
CDH2	1000	Leukemia, MESH:D015470		2-(2-chlor	49.77	41
DNMT1	1786	Leukemia, MESH:D015470		Alitretinoi	49.77	54
SOD2	6648	Leukemia, MESH:D015470		Arsenic Tr	49.73	85
IKBKB	3551	Leukemia, MESH:D015470		Allopurinc	49.7	63
FASLG	356	Leukemia, MESH:D015470		Arsenic Tr	49.65	74
CCNG2	901	Leukemia, MESH:D015470		Arsenic Tr	49.62	62
VDR	7421	Leukemia, MESH:D015473		arsenite|Ca	49.5	167
BUB1B	701	Leukemia, MESH:D015470		Arsenic Tr	49.42	48
PTCH1	5727	Leukemia, MESH:D015470		Androgen	49.4	35
F13A1	2162	Leukemia, MESH:D015470		Arsenic Tr	49.35	48
CDKN1B	1027	Leukemia, MESH:D015473		alvocidib|/	49.34	273
MMP1	4312	Leukemia, MESH:D015470		15-deoxy-	49.32	53
DIABLO	56616	Leukemia, MESH:D015470		alvocidib|/	49.31	38
FKBP5	2289	Leukemia, MESH:D015470		Arsenic Tr	49.18	53
CTNNB1	1499	Leukemia, MESH:D015470		2-(2-chlor	49.16	73
CTH	1491	Leukemia, MESH:D015470		Arsenic Tr	49.1	65
KRT19	3880	Leukemia, MESH:D015470		Alitretinoi	49.06	111
BNIP3	664	Leukemia, MESH:D015470		Arsenic Tr	49.02	58
VEGFC	7424	Leukemia, MESH:D015470		Alitretinoi	48.99	99
VIM	7431	Leukemia, MESH:D015470		15-deoxy-	48.94	74
CHI3L1	1116	Leukemia, MESH:D015470		Air Polluta	48.89	37
CASP2	835	Leukemia, MESH:D015470		Amsacrine	48.88	73
CYCS	54205	Leukemia, MESH:D015470		alvocidib|/	48.79	82
FOS	2353	Leukemia, MESH:D015470		15-deoxy-	48.79	74
UGT1A1	54658	Leukemia, MESH:D015470		Alitretinoi	48.73	48
SPARC	6678	Leukemia, MESH:D01	marker/m	Air Polluta	48.7	112
DDIT4	54541	Leukemia, MESH:D015470		Arsenic Tr	48.7	53
FYN	2534	Leukemia, MESH:D015470		Azacitidine	48.68	100
DUSP10	11221	Leukemia, MESH:D015470		Air Polluta	48.65	41
SPP1	6696	Leukemia, MESH:D015470		15-deoxy-	48.6	61
AURKA	6790	Leukemia, MESH:D015470		Arsenic Tr	48.43	66
FASN	2194	Leukemia, MESH:D015470		Alitretinoi	48.38	70
CXCL10	3627	Leukemia, MESH:D015470		Air Polluta	48.29	48
GSTP1	2950	Leukemia, MESH:D015470		15-deoxy-	48.26	72
THBD	7056	Leukemia, MESH:D015470		Arsenic Tr	48.17	43
TGFA	7039	Leukemia, MESH:D015470		Androgen	48.08	49
GATA3	2625	Leukemia, MESH:D015470		Air Polluta	48.07	36
GPNMB	10457	Leukemia, MESH:D015470		Arsenic Tr	48.07	44
NOS2	4843	Leukemia, MESH:D015470		15-deoxy-	48.05	77
BTG2	7832	Leukemia, MESH:D015473		alvocidib|/	48.01	276
CCL2	6347	Leukemia, MESH:D015470		15-deoxy-	47.93	86
DUSP4	1846	Leukemia, MESH:D015470		Arsenic Tr	47.93	123
IFIT3	3437	Leukemia, MESH:D015470		Arsenic Tr	47.93	47
DNAJB1	3337	Leukemia, MESH:D015470		Air Polluta	47.91	50
BST2	684	Leukemia, MESH:D015470		Air Polluta	47.88	104
TEK	7010	Leukemia, MESH:D015470		Arsenic Tr	47.88	97
COL1A2	1278	Leukemia, MESH:D015470		Alitretinoi	47.82	109
OCLN	1.01E+08	Leukemia, MESH:D015470		Arsenic Tr	47.78	35
LMNB1	4001	Leukemia, MESH:D015470		Benzene|C	47.77	57
APAF1	317	Leukemia, MESH:D015470		Air Polluta	47.64	63
NR1H4	9971	Leukemia, MESH:D015473		alvocidib|a	47.59	170
OGG1	4968	Leukemia, MESH:D015470		Air Polluta	47.57	77
ABCC4	10257	Leukemia, MESH:D015470		Arsenic Tr	47.49	42

MCL1	4170	Leukemia, MESH:D015473		2-(2-chlor	47.49	273
BIRC2	329	Leukemia, MESH:D015470		15-deoxy-	47.48	62
CXCL1	2919	Leukemia, MESH:D015470		Air Polluta	47.46	49
CSF2	1437	Leukemia, MESH:D01	marker/me	15-deoxy-	47.43	55
RRAS	6237	Leukemia, MESH:D015470		Arsenic Tr	47.31	100
SREBF1	6720	Leukemia, MESH:D015470		Alitretinoi	47.27	60
MYBL2	4605	Leukemia, MESH:D015470		Calcitriol|D	47.2	30
ISG15	9636	Leukemia, MESH:D015470		alvocidib|A	47.19	70
RARA	5914	Leukemia, MESH:D01	marker/me	Antimony	47	282
CD14	929	Leukemia, MESH:D015470		Air Polluta	46.98	45
GCLC	2729	Leukemia, MESH:D015470		15-deoxy-	46.96	74
RRM2B	50484	Leukemia, MESH:D015470		Azacitidine	46.81	43
TIMP1	7076	Leukemia, MESH:D015470		Arsenic Tr	46.8	121
PLAT	5327	Leukemia, MESH:D015470		Alitretinoi	46.72	112
ITGAM	3684	Leukemia, MESH:D015470		2-(2-chlor	46.7	38
ZMAT3	64393	Leukemia, MESH:D015470		Benzene|D	46.68	81
SQSTM1	8878	Leukemia, MESH:D015470		2-(2-chlor	46.66	49
TRP53	22059	Leukemia, MESH:D01	marker/me	ABT-737|A	46.6	129
CYP1A1	1543	Leukemia, MESH:D015470		Anthracyc	46.56	106
JUN	3725	Leukemia, MESH:D015473		2-(2-chlor	46.52	276
CAV1	857	Leukemia, MESH:D015470		2-(2-chlor	46.35	42
TNFSF9	8744	Leukemia, MESH:D015470		Arsenic Tr	46.3	63
FADS2	9415	Leukemia, MESH:D015470		Azacitidine	46.26	25
ANTXR1	84168	Leukemia, MESH:D015470		Arsenic Tr	46.21	32
ARHGEF2	9181	Leukemia, MESH:D015470		Air Polluta	46.21	28
XRCC6	2547	Leukemia, MESH:D015470		Arsenic Tr	46.21	71
CEACAM1	634	Leukemia, MESH:D015470		Calcitriol|D	46.17	61
PLK1	5347	Leukemia, MESH:D015470		Arsenic Tr	46.17	42
PLK2	10769	Leukemia, MESH:D015470		Benzene|D	46.17	54
GPX1	2876	Leukemia, MESH:D015470		Air Polluta	46.16	81
IFIT1	3434	Leukemia, MESH:D015470		alvocidib|E	46.16	105
XRCC1	7515	Leukemia, MESH:D015470		Benzene|C	46.16	120
STAT1	6772	Leukemia, MESH:D015473		Antimony	46.12	272
IRF1	3659	Leukemia, MESH:D015470		15-deoxy-	46.08	50
LITAF	9516	Leukemia, MESH:D015470		Arsenic Tr	46.06	43
CAT	847	Leukemia, MESH:D015470		15-deoxy-	46.01	100
MAPK8	5599	Leukemia, MESH:D015470		6,7-dimetl	45.94	49
CEBPB	1051	Leukemia, MESH:D015470		Alitretinoi	45.92	45
NPM1	4869	Leukemia, MESH:D01	marker/me	Alitretinoi	45.85	58
NR3C1	2908	Leukemia, MESH:D015470		15-deoxy-	45.85	91
TGFBR3	7049	Leukemia, MESH:D015470		Arsenic Tr	45.76	116
GSTM1	2944	Leukemia, MESH:D015473		alpha-Toc	45.69	279
RARB	5915	Leukemia, MESH:D015470		15-deoxy-	45.47	49
ABCC3	8714	Leukemia, MESH:D015473		Antimony	45.43	268
CCNB2	9133	Leukemia, MESH:D015470		Arsenic Tr	45.43	37
CRYAB	1410	Leukemia, MESH:D015470		Arsenic Tr	45.43	97
HBEGF	1839	Leukemia, MESH:D015470		Alitretinoi	45.35	43
ID1	3397	Leukemia, MESH:D015470		Air Polluta	45.35	41
ATF4	468	Leukemia, MESH:D015470		2-(2-chlor	45.33	55
CDC25A	993	Leukemia, MESH:D015470		Arsenic Tr	45.25	71
CDK1	983	Leukemia, MESH:D015473		alvocidib|A	45.22	271
MAP2K1	5604	Leukemia, MESH:D015470		2-(2-chlor	45.21	47
MBP	4155	Leukemia, MESH:D015470		Air Polluta	45.21	54
SRXN1	140809	Leukemia, MESH:D015470		Arsenic Tr	45.21	113
HSPA1B	3304	Leukemia, MESH:D015470		Arsenic Tr	45.2	49
IER3	8870	Leukemia, MESH:D015470		Calcitriol|C	45.2	37
MYB	4602	Leukemia, MESH:D015470		Air Polluta	45.2	35
NPPB	4879	Leukemia, MESH:D015470		Azacitidine	45.2	112

ANGPT2	285	Leukemia, MESH:D015470		Arsenic Tr	45.18	58
XBP1	7494	Leukemia, MESH:D015470		Air Polluta	45.16	50
EDN1	1906	Leukemia, MESH:D015470		15-deoxy-	45.15	121
KLK3	354	Leukemia, MESH:D015470		Androgen	45.15	42
PCK1	5105	Leukemia, MESH:D015470		15-deoxy-	45.08	32
MFGE8	4240	Leukemia, MESH:D015470		Azacitidine	45.04	41
ASNS	440	Leukemia, MESH:D015470		Arsenic Tr	45.01	46
SOD1	6647	Leukemia, MESH:D015470		Aclarubici	45.01	59
EGFL6	25975	Leukemia, MESH:D015470		Arsenic Tr	44.99	94
ISG20	3669	Leukemia, MESH:D015473		arsenite|C	44.96	165
CSF1R	1436	Leukemia, MESH:D01	marker/m	Air Polluta	44.95	49
LIF	3976	Leukemia, MESH:D015470		15-deoxy-	44.88	51
H2AX	3014	Leukemia, MESH:D015473		alpha-Toc	44.87	278
KIF14	9928	Leukemia, MESH:D015470		Calcitriol|D	44.83	25
CD44	960	Leukemia, MESH:D01	marker/m	Arsenic Tr	44.82	123
EGF	1950	Leukemia, MESH:D015470		2-(2-chlor	44.81	71
FASLG	356	Leukemia, MESH:D015473		alpha-Toc	44.78	272
CASP7	840	Leukemia, MESH:D015473		alpha-Toc	44.73	269
STAT5A	6776	Leukemia, MESH:D015473		4'-methox	44.68	268
TLR4	7099	Leukemia, MESH:D015470		Air Polluta	44.67	70
STAT5B	6777	Leukemia, MESH:D015470		Arsenic Tr	44.66	30
AKT1	207	Leukemia, MESH:D01	marker/m	alpha-Toc	44.65	277
EDNRB	1910	Leukemia, MESH:D015470		Arsenic Tr	44.63	60
FZD2	2535	Leukemia, MESH:D015470		Air Polluta	44.63	41
PCSK9	255738	Leukemia, MESH:D015470		Arsenic Tr	44.63	31
TNFAIP3	7128	Leukemia, MESH:D015470		Arsenic Tr	44.62	114
DUSP1	1843	Leukemia, MESH:D015470		Androgen	44.59	47
HMGB2	3148	Leukemia, MESH:D015470		Arsenic Tr	44.57	53
DEPDC1	55635	Leukemia, MESH:D015470		Arsenic Tr	44.49	43
GINS2	51659	Leukemia, MESH:D015470		Calcitriol|D	44.49	26
ST3GAL5	8869	Leukemia, MESH:D015470		Benzene|C	44.49	107
LMNA	4000	Leukemia, MESH:D015470		Air Polluta	44.47	36
TYMS	7298	Leukemia, MESH:D015473		Antimony	44.47	271
TRIB3	57761	Leukemia, MESH:D015470		Arsenic Tr	44.44	50
KLF2	10365	Leukemia, MESH:D015470		Arsenic Tr	44.41	53
NDC80	10403	Leukemia, MESH:D015470		Arsenic Tr	44.41	97
HK2	3099	Leukemia, MESH:D015470		Arsenic Tr	44.39	33
KLF4	9314	Leukemia, MESH:D015470		Air Polluta	44.39	53
KAT2B	8850	Leukemia, MESH:D015470		Arsenic Tr	44.36	38
RFC3	5983	Leukemia, MESH:D015470		Azacitidine	44.36	29
FGR	2268	Leukemia, MESH:D015470		Air Polluta	44.33	27
ITPR1	3708	Leukemia, MESH:D015473		Arsenic|Ar	44.33	268
CXCL12	6387	Leukemia, MESH:D015470		Arsenic Tr	44.32	61
DLGAP5	9787	Leukemia, MESH:D015470		Bortezomi	44.3	34
TMPO	7112	Leukemia, MESH:D015470		Arsenic Tr	44.3	30
FDFT1	2222	Leukemia, MESH:D015470		Alitretinoi	44.23	66
H4C3	8364	Leukemia, MESH:D015470		Arsenic Tr	44.16	94
GSN	2934	Leukemia, MESH:D015470		Arsenic Tr	44.1	40
MAD2L1	4085	Leukemia, MESH:D015470		Arsenic Tr	44.08	30
TAP1	6890	Leukemia, MESH:D015470		Arsenic Tr	44.08	75
CDKN3	1033	Leukemia, MESH:D015470		Arsenic Tr	44.01	49
MPO	4353	Leukemia, MESH:D015470		Allopurinc	43.96	71
MATN2	4147	Leukemia, MESH:D015470		Arsenic Tr	43.84	96
AGRN	375790	Leukemia, MESH:D01	marker/m	Bortezomi	43.83	56
CCL20	6364	Leukemia, MESH:D015470		Air Polluta	43.83	56
CD69	969	Leukemia, MESH:D015470		Air Polluta	43.83	42
CDKN1C	1028	Leukemia, MESH:D015470		Alitretinoi	43.83	113
HSPA8	3312	Leukemia, MESH:D015470		Air Polluta	43.77	55

MMP10	4319	Leukemia, MESH:D015470		Alitretinoi	43.76	34
IL1RN	3557	Leukemia, MESH:D015473		Antimony	43.64	268
ITGAM	3684	Leukemia, MESH:D01	marker/m	2-(2-chlor	43.59	273
CXCR4	7852	Leukemia, MESH:D015473		Arsenic|Ar	43.59	277
CYP3A4	1576	Leukemia, MESH:D015473		2-(2-chlor	43.58	270
KIF2C	11004	Leukemia, MESH:D015470		Azacitidin	43.54	30
TOP2A	7153	Leukemia, MESH:D015473		Arsenic|Ar	43.54	277
ANXA2	302	Leukemia, MESH:D01	marker/m	Alitretinoi	43.52	117
CRKL	1399	Leukemia, MESH:D015470		alvocidib|/	43.52	25
CCNB1	891	Leukemia, MESH:D015473		alvocidib|/	43.47	271
TNFRSF1A	7132	Leukemia, MESH:D015470		Air Polluta	43.47	61
CCL3	6348	Leukemia, MESH:D015470		Air Polluta	43.45	61
PLK4	10733	Leukemia, MESH:D015470		Arsenic Tr	43.45	31
TPD52L1	7164	Leukemia, MESH:D015470		Arsenic Tr	43.45	34
CDC25C	995	Leukemia, MESH:D015470		Arsenic Tr	43.37	47
TFPI	7035	Leukemia, MESH:D015470		Benzene|B	43.34	109
CRISPLD2	83716	Leukemia, MESH:D015470		Air Polluta	43.32	89
EIF5A	1984	Leukemia, MESH:D015470		Arsenic Tr	43.32	39
HNRNPD	3184	Leukemia, MESH:D015470		Air Polluta	43.32	53
ASS1	445	Leukemia, MESH:D015470		Arsenic Tr	43.29	48
SAT1	6303	Leukemia, MESH:D015470		Arsenic Tr	43.29	41
SELENOP	6414	Leukemia, MESH:D015470		Androgen	43.29	39
TRIB1	10221	Leukemia, MESH:D015470		Azacitidin	43.23	37
HSD11B1	3290	Leukemia, MESH:D015470		Alitretinoi	43.22	29
RHOA	387	Leukemia, MESH:D015470		Alitretinoi	43.22	54
SLC22A18	5002	Leukemia, MESH:D015470		Arsenic Tr	43.22	42
COL6A3	1293	Leukemia, MESH:D015470		Arsenic Tr	43.2	96
ESR1	2099	Leukemia, MESH:D015470		Alitretinoi	43.15	102
CEBPD	1052	Leukemia, MESH:D01	marker/m	Arsenic Tr	43.14	103
DKK1	22943	Leukemia, MESH:D015470		Arsenic Tr	43.13	51
F2R	2149	Leukemia, MESH:D015470		Arsenic Tr	43.12	46
RHOB	388	Leukemia, MESH:D015470		Alitretinoi	43.12	43
KMT2A	4297	Leukemia, MESH:D01	marker/m	Arsenic Tr	43.08	99
CLGN	1047	Leukemia, MESH:D015470		Arsenic Tr	43.07	30
ERBB4	2066	Leukemia, MESH:D015470		Arsenic Tr	43.07	35
DES	1674	Leukemia, MESH:D015470		Azacitidin	43.03	58
IGFBP4	3487	Leukemia, MESH:D015470		Arsenic Tr	42.93	34
CCL4	6351	Leukemia, MESH:D015473		Arsenic|Ar	42.86	275
APOD	347	Leukemia, MESH:D015470		Chloroqui	42.84	34
NFAT5	10725	Leukemia, MESH:D015470		Allopurinc	42.84	42
EZH2	2146	Leukemia, MESH:D015470		Arsenic Tr	42.83	33
SDC4	6385	Leukemia, MESH:D015470		Air Polluta	42.83	95
DIAPH3	81624	Leukemia, MESH:D015470		Arsenic Tr	42.77	30
ZMYND8	23613	Leukemia, MESH:D015470		Arsenic Tr	42.77	30
IGFBP3	3486	Leukemia, MESH:D015473		Arsenic|Ar	42.76	276
BIRC2	329	Leukemia, MESH:D015473		Arsenic Tr	42.75	268
DAPK1	1612	Leukemia, MESH:D01	marker/m	Air Polluta	42.73	50
ALDH1A1	216	Leukemia, MESH:D015470		Allopurinc	42.72	67
STAT3	6774	Leukemia, MESH:D015473		2-(2-chlor	42.66	271
BRCA1	672	Leukemia, MESH:D015470		Arsenic Tr	42.63	52
NUSAP1	51203	Leukemia, MESH:D015470		Arsenic Tr	42.63	32
PPARD	5467	Leukemia, MESH:D015470		15-deoxy-	42.63	70
TP53I3	9540	Leukemia, MESH:D015470		Amsacrine	42.63	66
P4HA1	5033	Leukemia, MESH:D015470		Arsenic Tr	42.6	41
BNC2	54796	Leukemia, MESH:D015470		Arsenic Tr	42.57	97
PDGFRB	5159	Leukemia, MESH:D015473		alpha-Toc	42.57	270
CYP1B1	1545	Leukemia, MESH:D015470		Air Polluta	42.51	91
NCOA3	8202	Leukemia, MESH:D015470		Benzoates	42.48	23

POSTN	10631	Leukemia,	MESH:D015470	Arsenic Tr	42.48	97
TK1	7083	Leukemia,	MESH:D015470	Arsenic Tr	42.36	105
CGB3	1082	Leukemia,	MESH:D015470	15-deoxy-	42.34	17
KCNJ2	3759	Leukemia,	MESH:D015470	Air Polluta	42.34	76
LDHA	3939	Leukemia,	MESH:D015470	Arsenic Tr	42.34	29
SLC20A1	6574	Leukemia,	MESH:D015470	Air Polluta	42.34	34
STK17B	9262	Leukemia,	MESH:D015470	Arsenic Tr	42.34	34
TNFSF11	8600	Leukemia,	MESH:D015470	Amsacrine	42.34	47
KRT18	3875	Leukemia,	MESH:D015470	ABT-737|A	42.27	32
CENPA	1058	Leukemia,	MESH:D015470	Azacitidine	42.25	24
FANCD2	2177	Leukemia,	MESH:D015470	Arsenic Tr	42.25	31
ITGB5	3693	Leukemia,	MESH:D015470	Benzene|C	42.25	109
TMEM97	27346	Leukemia,	MESH:D015470	Air Polluta	42.25	39
MTHFD2	10797	Leukemia,	MESH:D015470	Arsenic Tr	42.24	49
CCNE1	898	Leukemia,	MESH:D015473	alvocidib|A	42.23	270
AGT	183	Leukemia,	MESH:D015470	15-deoxy-	42.22	37
FABP4	2167	Leukemia,	MESH:D015470	15-deoxy-	42.22	43
NR1H4	9971	Leukemia,	MESH:D015470	alvocidib|E	42.22	80
EPO	2056	Leukemia,	MESH:D015470	Benzene|B	42.2	58
PPP1CB	5500	Leukemia,	MESH:D015470	Arsenic Tr	42.2	41
RB1	5925	Leukemia,	MESH:D015473	alvocidib|A	42.18	270
WEE1	7465	Leukemia,	MESH:D015470	Air Polluta	42.14	47
HAS2	3037	Leukemia,	MESH:D015470	Arsenic Tr	42.13	108
TFPI2	7980	Leukemia,	MESH:D015470	Decitabine	42.13	41
HMGB1	3146	Leukemia,	MESH:D015470	Arsenic Tr	42.1	124
UHRF1	29128	Leukemia,	MESH:D015470	Arsenic Tr	42.05	28
GBP1	2633	Leukemia,	MESH:D015470	Air Polluta	41.92	26
PKP2	5318	Leukemia,	MESH:D015470	Calcitriol|C	41.92	90
TPD52	7163	Leukemia,	MESH:D015470	Arsenic Tr	41.92	40
LIG1	3978	Leukemia,	MESH:D015470	Calcitriol|D	41.91	32
VEGFA	7422	Leukemia,	MESH:D015473	alvocidib|A	41.91	277
CDKN2B	1030	Leukemia,	MESH:D015473	Antimony	41.9	271
SMAD2	4087	Leukemia,	MESH:D015470	15-deoxy-	41.87	58
BUB1	699	Leukemia,	MESH:D015470	Calcitriol|D	41.86	41
NT5E	4907	Leukemia,	MESH:D015470	Arsenic Tr	41.86	39
HSPB1	3315	Leukemia,	MESH:D01 marker/m	15-deoxy-	41.84	45
ATF5	22809	Leukemia,	MESH:D015470	Benzene|B	41.79	42
CREBBP	1387	Leukemia,	MESH:D015470	Androgen	41.79	40
NANOG	79923	Leukemia,	MESH:D015470	ABT-737|A	41.79	31
IL12A	3592	Leukemia,	MESH:D015470	Alitretinoi	41.78	42
SAMHD1	25939	Leukemia,	MESH:D015470	Air Polluta	41.78	24
PPARGC1A	10891	Leukemia,	MESH:D015470	Alitretinoi	41.75	50
HIF1A	3091	Leukemia,	MESH:D015473	alpinumiso	41.71	275
SPINT2	10653	Leukemia,	MESH:D015470	Arsenic Tr	41.69	31
CTSH	1512	Leukemia,	MESH:D01 marker/m	Arsenic Tr	41.68	103
IGF1	3479	Leukemia,	MESH:D015470	Alitretinoi	41.68	65
MAP1LC3I	81631	Leukemia,	MESH:D015470	ABT-737|A	41.67	43
TMPRSS2	7113	Leukemia,	MESH:D015470	Allopurino	41.67	34
CA12	771	Leukemia,	MESH:D015470	Calcitriol|C	41.64	94
COX11	1353	Leukemia,	MESH:D015470	Air Polluta	41.64	21
HSF1	3297	Leukemia,	MESH:D015470	15-deoxy-	41.64	45
RGS1	5996	Leukemia,	MESH:D015470	Air Polluta	41.64	103
SP110	3431	Leukemia,	MESH:D015470	Arsenic Tr	41.64	59
FLT3	2322	Leukemia,	MESH:D01 marker/m	Arsenic Tr	41.52	31
H2BC5	3017	Leukemia,	MESH:D015470	Arsenic Tr	41.52	36
CCN1	3491	Leukemia,	MESH:D015470	Arsenic Tr	41.51	34
KEAP1	9817	Leukemia,	MESH:D015470	Arsenic Tr	41.5	60
FGFR3	2261	Leukemia,	MESH:D015470	Calcitriol|D	41.49	25

BID	637	Leukemia, MESH:D015473		4'-methox	41.48	274
PHGDH	26227	Leukemia, MESH:D015470		Air Polluta	41.48	49
ZWINT	11130	Leukemia, MESH:D015470		Arsenic Tr	41.46	29
FSCN1	6624	Leukemia, MESH:D015470		Arsenic Tr	41.38	37
SRGN	5552	Leukemia, MESH:D015470		Arsenic Tr	41.38	114
CCN4	8840	Leukemia, MESH:D015470		Arsenic Tr	41.35	36
BHLHE40	8553	Leukemia, MESH:D015470		Arsenic Tr	41.32	39
CDKN3	1033	Leukemia, MESH:D015473		Antimony	41.32	264
GCLC	2729	Leukemia, MESH:D015473		alpha-Toc	41.32	271
HAS2	3037	Leukemia, MESH:D015473		Arsenic|Ar	41.29	273
CD38	952	Leukemia, MESH:D015470		2-(2-chlor	41.25	30
ZBTB16	7704	Leukemia, MESH:D015470		Busulfan|D	41.25	29
CEBPA	1050	Leukemia, MESH:D01	marker/m	15-deoxy-	41.2	54
HMGCS1	3157	Leukemia, MESH:D015470		Air Polluta	41.14	37
BGLAP	632	Leukemia, MESH:D015470		15-deoxy-	41.09	33
CITED2	10370	Leukemia, MESH:D015470		Air Polluta	41.04	107
FBLN1	2192	Leukemia, MESH:D015470		Arsenic Tr	41.03	98
ALDOA	226	Leukemia, MESH:D015470		Arsenic Tr	41.02	33
TGFBR2	7048	Leukemia, MESH:D015470		Arsenic Tr	41.02	40
CARD6	84674	Leukemia, MESH:D015470		Air Polluta	40.99	25
SULF1	23213	Leukemia, MESH:D015470		Arsenic Tr	40.99	32
TJP1	7082	Leukemia, MESH:D015470		Arsenic Tr	40.97	41
ERBB3	2065	Leukemia, MESH:D015470		Androgen	40.95	62
RARA	5914	Leukemia, MESH:D015470		Alitretinoi	40.94	45
IL4	3565	Leukemia, MESH:D015473		Antimony	40.93	269
LYN	4067	Leukemia, MESH:D015470		alvocidib|E	40.93	100
MVP	9961	Leukemia, MESH:D015470		Arsenic Tr	40.93	33
RCAN1	1827	Leukemia, MESH:D015470		Calcitriol|D	40.93	40
TLR3	7098	Leukemia, MESH:D015470		Arsenic Tr	40.93	44
GLS	2744	Leukemia, MESH:D015470		Air Polluta	40.86	94
SLC7A5	8140	Leukemia, MESH:D015470		Air Polluta	40.86	60
EGR3	1960	Leukemia, MESH:D015473		Antimony	40.85	272
RGCC	28984	Leukemia, MESH:D015470		Air Polluta	40.82	42
SLC5A5	6528	Leukemia, MESH:D015470		Alitretinoi	40.82	33
SULF2	55959	Leukemia, MESH:D015470		Arsenic Tr	40.82	36
TTK	7272	Leukemia, MESH:D015470		Azacitidine	40.82	32
LPL	4023	Leukemia, MESH:D015470		15-deoxy-	40.79	47
CHEK1	1111	Leukemia, MESH:D015470		Arsenic Tr	40.78	53
IRX4	50805	Leukemia, MESH:D015470		Calcitriol|D	40.78	65
NDRG1	10397	Leukemia, MESH:D015470		Arsenic Tr	40.78	100
VCAM1	7412	Leukemia, MESH:D015473		alpha-Toc	40.77	274
CCND1	595	Leukemia, MESH:D015473		alpha-Toc	40.75	274
GAPDH	2597	Leukemia, MESH:D015470		Alitretinoi	40.74	109
DSP	1832	Leukemia, MESH:D015470		Arsenic Tr	40.73	32
GREM1	26585	Leukemia, MESH:D015470		Arsenic Tr	40.73	35
HDAC4	9759	Leukemia, MESH:D015470		Arsenic Tr	40.73	62
NRP1	8829	Leukemia, MESH:D015470		Calcitriol|C	40.69	99
EDN1	1906	Leukemia, MESH:D015473		2-(2-chlor	40.68	275
C3	718	Leukemia, MESH:D015470		Androgen	40.65	35
KIT	3815	Leukemia, MESH:D01	marker/m	alvocidib|A	40.64	271
TNFAIP3	7128	Leukemia, MESH:D015473		Antimony	40.64	273
ABCB1	5243	Leukemia, MESH:D015473		alpha-Toc	40.63	274
FEN1	2237	Leukemia, MESH:D015470		Azacitidine	40.62	37
SESN1	27244	Leukemia, MESH:D015470		Arsenic Tr	40.62	47
B2M	567	Leukemia, MESH:D015470		Air Polluta	40.6	96
CD38	952	Leukemia, MESH:D015473		2-(2-chlor	40.58	268
CDC20	991	Leukemia, MESH:D015470		Arsenic Tr	40.57	32
INPP4B	8821	Leukemia, MESH:D01	marker/m	Air Polluta	40.54	63

SOX2	6657	Leukemia, MESH:D015470		Arsenic Tr	40.52	40
THY1	7070	Leukemia, MESH:D015470		Bortezomi	40.51	96
XIAP	331	Leukemia, MESH:D015473		Aclarubici	40.44	271
ADAMTS1	9510	Leukemia, MESH:D015470		Arsenic Tr	40.43	97
IFNB1	3456	Leukemia, MESH:D015470		Allopurinc	40.41	47
AKR1C3	8644	Leukemia, MESH:D015470		15-deoxy-	40.39	59
SUV39H1	6839	Leukemia, MESH:D015470		Bortezomi	40.39	19
CASP10	843	Leukemia, MESH:D015470		Air Polluta	40.36	31
DUSP2	1844	Leukemia, MESH:D015470		Arsenic Tr	40.36	53
MYOF	26509	Leukemia, MESH:D015470		Air Polluta	40.36	93
IL1RN	3557	Leukemia, MESH:D015470		15-deoxy-	40.33	48
CDK2	1017	Leukemia, MESH:D015473		alvocidib|/	40.3	267
RPS6KB1	6198	Leukemia, MESH:D015473		2-(2-chlor	40.27	275
IFITM1	8519	Leukemia, MESH:D015473		Arsenic|ars	40.24	163
ERN1	2081	Leukemia, MESH:D015470		Allopurinc	40.2	36
PRKCD	5580	Leukemia, MESH:D015470		Arsenic Tr	40.2	72
SCUBE2	57758	Leukemia, MESH:D015470		Arsenic Tr	40.18	25
NPHS1	4868	Leukemia, MESH:D015470		Bezafibrat	40.11	50
TRAF1	7185	Leukemia, MESH:D015470		Arsenic Tr	40.11	32
FGFR1	2260	Leukemia, MESH:D015470		Arsenic Tr	40.09	37
G0S2	50486	Leukemia, MESH:D015470		Arsenic Tr	40.09	33
KPNA2	3838	Leukemia, MESH:D015473		Arsenic|Ar	40.08	271
NOS3	4846	Leukemia, MESH:D015470		15-deoxy-	40.08	47
RXRA	6256	Leukemia, MESH:D015470		15-deoxy-	40.08	52
NFKBIA	4792	Leukemia, MESH:D015473		alpha-Toc	40.07	272
IL1A	3552	Leukemia, MESH:D015470		15-deoxy-	40.01	51
PRDX1	5052	Leukemia, MESH:D015473		Antimony	40.01	268
ZEB1	6935	Leukemia, MESH:D015470		Arsenic Tr	40.01	37
DUSP4	1846	Leukemia, MESH:D015473		Arsenic Tr	40	270
IFIT3	3437	Leukemia, MESH:D015473		Arsenic Tr	40	267
MTHFD1L	25902	Leukemia, MESH:D015470		Arsenic Tr	39.99	35
SH2D4A	63898	Leukemia, MESH:D015470		Calcitriol|D	39.99	15
PRDM2	7799	Leukemia, MESH:D015470		Bortezomi	39.94	31
RFTN1	23180	Leukemia, MESH:D015470		Dexameth	39.94	26
CPT2	1376	Leukemia, MESH:D015470		Allopurinc	39.93	32
IGFBP5	3488	Leukemia, MESH:D015473		alpha-Toc	39.93	271
MYLK	4638	Leukemia, MESH:D015470		Arsenic Tr	39.93	36
FOXA1	3169	Leukemia, MESH:D015470		Alitretinoi	39.88	29
NABP1	64859	Leukemia, MESH:D015470		Arsenic Tr	39.88	31
LCN2	3934	Leukemia, MESH:D015470		Alitretinoi	39.83	43
PRKDC	5591	Leukemia, MESH:D015470		Benzene|B	39.82	55
RGS4	5999	Leukemia, MESH:D015470		Cytarabine	39.82	92
SKP2	6502	Leukemia, MESH:D015470		Arsenic Tr	39.82	33
CYP19A1	1588	Leukemia, MESH:D015470		Alitretinoi	39.81	37
MAP1A	4130	Leukemia, MESH:D015470		Azacitidine	39.8	21
NOTCH1	4851	Leukemia, MESH:D015470		Arsenic Tr	39.78	50
ENC1	8507	Leukemia, MESH:D015470		Arsenic Tr	39.76	64
MAPK3	5595	Leukemia, MESH:D015473		2-(2-chlor	39.76	277
PRIM1	5557	Leukemia, MESH:D015470		Air Polluta	39.76	23
RASSF1	11186	Leukemia, MESH:D015470		ABT-737|/	39.76	44
EPHX1	2052	Leukemia, MESH:D01	marker/m	Arsenic Tr	39.75	66
SOX4	6659	Leukemia, MESH:D015470		Arsenic Tr	39.72	35
SLC2A4	6517	Leukemia, MESH:D015470		Alitretinoi	39.71	26
SMAD2	4087	Leukemia, MESH:D015473		Arsenic|Ar	39.68	273
MT1X	4501	Leukemia, MESH:D015473		Antimony	39.67	271
MAPK1	5594	Leukemia, MESH:D015473		2-(2-chlor	39.65	277
TERT	7015	Leukemia, MESH:D01	marker/m	Alitretinoi	39.64	53
AMIGO2	347902	Leukemia, MESH:D015470		Dasatinib|I	39.64	23

ATP2B4	493	Leukemia, MESH:D015470		Decitabine	39.64	32
ACACA	31	Leukemia, MESH:D015470		Arsenic Tr	39.58	55
GJA1	2697	Leukemia, MESH:D015470		Arsenic Tr	39.53	63
SMAD3	4088	Leukemia, MESH:D015470		Alitretinoi	39.53	50
AURKA	6790	Leukemia, MESH:D015473		Arsenic|Ar	39.44	272
ETS1	2113	Leukemia, MESH:D015470		Air Polluta	39.43	43
PLPP3	8613	Leukemia, MESH:D015470		Arsenic Tr	39.43	47
IL18	3606	Leukemia, MESH:D015470		Allopurinc	39.42	37
CCL4	6351	Leukemia, MESH:D015470		Air Polluta	39.41	73
IL1R1	3554	Leukemia, MESH:D015470		Benzene|B	39.36	43
LAMA2	3908	Leukemia, MESH:D015470		Alitretinoi	39.36	33
PLIN2	123	Leukemia, MESH:D015470		Arsenic Tr	39.36	40
AIFM1	9131	Leukemia, MESH:D015470		2-(2-chlor	39.35	34
ITGA5	3678	Leukemia, MESH:D015470		Benzene|C	39.34	47
S100A4	6275	Leukemia, MESH:D015470		Arsenic Tr	39.28	103
CFD	1675	Leukemia, MESH:D015470		Arsenic Tr	39.25	35
EMP1	2012	Leukemia, MESH:D015470		Air Polluta	39.25	30
RUNX2	860	Leukemia, MESH:D015470		15-deoxy-	39.23	44
DCBLD2	131566	Leukemia, MESH:D015470		Arsenic Tr	39.22	31
SPHK1	8877	Leukemia, MESH:D015470		Bortezomi	39.15	58
BDNF	627	Leukemia, MESH:D015470		Androgen	39.09	48
PELI1	57162	Leukemia, MESH:D015470		Air Polluta	39.08	112
H1-0	3005	Leukemia, MESH:D01	marker/m	Arsenic Tr	39.06	32
BRCA2	675	Leukemia, MESH:D015470		Arsenic Tr	39.06	106
FPR1	2357	Leukemia, MESH:D015470		Air Polluta	39.06	43
MCM2	4171	Leukemia, MESH:D015470		Arsenic Tr	39.05	39
APOBEC3(	60489	Leukemia, MESH:D015470		Air Polluta	39.04	14
HSPA5	3309	Leukemia, MESH:D015470		Allopurinc	39.01	56
LY6E	4061	Leukemia, MESH:D015473		Arsenic Tr	38.97	265
MCM6	4175	Leukemia, MESH:D015470		Arsenic Tr	38.97	40
MALAT1	378938	Leukemia, MESH:D01	marker/m	Arsenic Tr	38.96	28
TNF	7124	Leukemia, MESH:D015473		alpha-Toc	38.94	282
NFE2L2	4780	Leukemia, MESH:D015473		2-(2-chlor	38.92	274
RELB	5971	Leukemia, MESH:D015473		Arsenic|Ar	38.9	167
CHCHD3	54927	Leukemia, MESH:D015470		Decitabine	38.88	26
SERTAD1	29950	Leukemia, MESH:D015470		Benzene|C	38.85	107
PRC1	9055	Leukemia, MESH:D015473		Arsenic Tr	38.83	270
CYP26A1	1592	Leukemia, MESH:D015470		Alitretinoi	38.82	42
NGF	4803	Leukemia, MESH:D015470		Calcitriol|C	38.82	36
UCP2	7351	Leukemia, MESH:D015470		Arsenic Tr	38.82	45
ATG5	9474	Leukemia, MESH:D015470		Arsenic Tr	38.74	37
PLPP2	8612	Leukemia, MESH:D015470		Arsenic Tr	38.71	35
SERPINE2	5270	Leukemia, MESH:D015470		Calcitriol|D	38.7	33
INS	3630	Leukemia, MESH:D015470		15-deoxy-	38.68	46
HMGA2	8091	Leukemia, MESH:D015470		Arsenic Tr	38.63	39
LEF1	51176	Leukemia, MESH:D015470		Air Polluta	38.61	31
COL3A1	1281	Leukemia, MESH:D015470		Benzene|C	38.59	115
TNC	3371	Leukemia, MESH:D015470		Azacitidine	38.59	46
MAFF	23764	Leukemia, MESH:D015470		Arsenic Tr	38.57	56
ERMP1	79956	Leukemia, MESH:D015470		Calcitriol|D	38.54	25
GSK3B	2932	Leukemia, MESH:D015470		Arsenic Tr	38.54	67
IGFBP7	3490	Leukemia, MESH:D015470		Arsenic Tr	38.53	51
CDKN2C	1031	Leukemia, MESH:D015470		Arsenic Tr	38.52	32
HLA-DRA	3122	Leukemia, MESH:D015470		Arsenic Tr	38.52	40
PER1	5187	Leukemia, MESH:D015470		Air Polluta	38.52	25
AURKB	9212	Leukemia, MESH:D015470		Calcitriol|C	38.51	28
EEF1A1	1915	Leukemia, MESH:D015473		Arsenic Tr	38.47	269
S100A9	6280	Leukemia, MESH:D015473		Antimony	38.45	270

ANGPT1	284	Leukemia, MESH:D015470		Arsenic Tr	38.42	54
CDCA8	55143	Leukemia, MESH:D015470		Calcitriol|C	38.42	90
NCL	4691	Leukemia, MESH:D015470		Amsacrine	38.42	35
S100P	6286	Leukemia, MESH:D015470		Azacitidine	38.42	99
TNNI3	7137	Leukemia, MESH:D015470		Azacitidine	38.42	55
MLKL	197259	Leukemia, MESH:D015470		Decitabine	38.4	23
TENT5C	54855	Leukemia, MESH:D015470		Air Polluta	38.4	94
CTSL	1514	Leukemia, MESH:D015470		Arsenic Tr	38.36	102
ERRFI1	54206	Leukemia, MESH:D015470		Arsenic Tr	38.34	35
CD24	1E+08	Leukemia, MESH:D015470		Arsenic Tr	38.31	31
FHL1	2273	Leukemia, MESH:D015470		Air Polluta	38.31	50
PPIA	5478	Leukemia, MESH:D015470		Air Polluta	38.31	35
S100A8	6279	Leukemia, MESH:D015473		Antimony	38.28	270
IRF6	3664	Leukemia, MESH:D015470		Arsenic Tr	38.27	34
NBN	4683	Leukemia, MESH:D015470		Arsenic Tr	38.27	52
STOM	2040	Leukemia, MESH:D015470		Benzene|C	38.27	105
KIF20A	10112	Leukemia, MESH:D015470		Benzene|C	38.26	49
IFIT2	3433	Leukemia, MESH:D015470		Arsenic Tr	38.21	43
PHLDA2	7262	Leukemia, MESH:D015470		Arsenic Tr	38.21	36
HSPD1	3329	Leukemia, MESH:D015470		Arsenic Tr	38.15	36
RAD23B	5887	Leukemia, MESH:D015470		Arsenic Tr	38.14	32
PDK4	5166	Leukemia, MESH:D015470		Air Polluta	38.12	38
CDC25A	993	Leukemia, MESH:D015473		Arsenic|Ar	38.11	275
EFNA1	1942	Leukemia, MESH:D015470		Arsenic Tr	38.08	104
HSPA2	3306	Leukemia, MESH:D015470		Arsenic Tr	38.08	47
CASP4	837	Leukemia, MESH:D015470		Arsenic Tr	38.07	44
ID2	3398	Leukemia, MESH:D01	marker/m	Arsenic Tr	38.06	54
CLCF1	23529	Leukemia, MESH:D015470		Calcitriol|D	38.01	29
ATP2A2	488	Leukemia, MESH:D015470		Arsenic Tr	38	44
CD83	9308	Leukemia, MESH:D015470		Air Polluta	38	21
MYC	4609	Leukemia, MESH:D015473		alpha-Toc	38	274
HGF	3082	Leukemia, MESH:D01	marker/m	ABT-737|A	37.93	30
KLF6	1316	Leukemia, MESH:D015470		15-deoxy-	37.93	54
KLF5	688	Leukemia, MESH:D015473		Antimony	37.91	271
UCP1	7350	Leukemia, MESH:D015470		Alitretinoi	37.91	40
CYCS	54205	Leukemia, MESH:D015473		4'-methox	37.9	272
LY96	23643	Leukemia, MESH:D015470		Arsenic Tr	37.9	47
BNC1	646	Leukemia, MESH:D015470		Cytarabine	37.86	91
GSR	2936	Leukemia, MESH:D015470		Allopurino	37.86	66
RGS2	5997	Leukemia, MESH:D01	marker/m	Arsenic Tr	37.85	61
EIF5A	1984	Leukemia, MESH:D015473		Arsenic|Ar	37.82	269
POR	5447	Leukemia, MESH:D015470		Benzene|B	37.82	44
SCD	6319	Leukemia, MESH:D015470		Alitretinoi	37.81	37
XPC	7508	Leukemia, MESH:D015470		Arsenic Tr	37.8	67
CPEB2	132864	Leukemia, MESH:D015470		Calcitriol|D	37.76	26
CSK	1445	Leukemia, MESH:D015470		Calcitriol|C	37.76	20
RASSF2	9770	Leukemia, MESH:D015470		Benzene|C	37.72	102
SVEP1	79987	Leukemia, MESH:D015470		Calcitriol|C	37.72	93
SESN2	83667	Leukemia, MESH:D015470		Arsenic Tr	37.71	50
HSPA4L	22824	Leukemia, MESH:D015470		Arsenic Tr	37.7	28
MUC1	4582	Leukemia, MESH:D015470		Air Polluta	37.7	27
CXCL8	3576	Leukemia, MESH:D015473		alpha-Toc	37.67	274
BCL6	604	Leukemia, MESH:D015470		Arsenic Tr	37.66	47
NFKB2	4791	Leukemia, MESH:D015470		Arsenic Tr	37.66	38
MKI67	4288	Leukemia, MESH:D015473		2-(2-chlor	37.65	270
CLDN1	9076	Leukemia, MESH:D015470		Air Polluta	37.64	32
DHRS9	10170	Leukemia, MESH:D015470		Alitretinoi	37.64	49
E2F3	1871	Leukemia, MESH:D015470		Arsenic Tr	37.64	35

HES1	3280	Leukemia, MESH:D015470	Arsenic Tr	37.64	32
LFNG	3955	Leukemia, MESH:D015470	Air Polluta	37.64	26
HMGB2	3148	Leukemia, MESH:D015473	Arsenic|Ar	37.63	267
PFAS	5198	Leukemia, MESH:D015473	Arsenic|Ar	37.61	267
F2RL1	2150	Leukemia, MESH:D015470	Air Polluta	37.6	34
MAP3K8	1326	Leukemia, MESH:D015470	Benzene|B	37.6	45
ERBB3	2065	Leukemia, MESH:D015473	Arsenic|ars	37.57	167
PLAT	5327	Leukemia, MESH:D015473	Arsenic Tr	37.57	272
EXOSC2	23404	Leukemia, MESH:D015470	Arsenic Tr	37.56	27
JCHAIN	3512	Leukemia, MESH:D015470	Air Polluta	37.56	30
PHF19	26147	Leukemia, MESH:D015470	Air Polluta	37.56	17
RAF1	5894	Leukemia, MESH:D015470	15-deoxy-	37.56	31
IL3	3562	Leukemia, MESH:D015470	Benzene|B	37.52	33
PPARA	5465	Leukemia, MESH:D015470	15-deoxy-	37.48	63
CENPF	1063	Leukemia, MESH:D015473	Arsenic|ars	37.44	162
DIO2	1734	Leukemia, MESH:D015470	Azacitidine	37.43	29
ALDOC	230	Leukemia, MESH:D015470	Arsenic Tr	37.41	45
CCL5	6352	Leukemia, MESH:D015470	15-deoxy-	37.41	47
MFAP2	4237	Leukemia, MESH:D015470	Alitretinoi	37.41	26
ITGA2	3673	Leukemia, MESH:D015470	Arsenic Tr	37.4	29
NME1	4830	Leukemia, MESH:D015470	Arsenic Tr	37.4	27
SOCS1	8651	Leukemia, MESH:D015470	15-deoxy-	37.4	43
LYZ	4069	Leukemia, MESH:D015470	Air Polluta	37.39	93
IRS1	3667	Leukemia, MESH:D015470	Allopurino	37.38	30
TGFB2	7042	Leukemia, MESH:D015470	Benzene|C	37.38	51
DHRS2	10202	Leukemia, MESH:D015473	Arsenic Tr	37.36	267
EPCAM	4072	Leukemia, MESH:D015470	Arsenic Tr	37.33	35
DHRS3	9249	Leukemia, MESH:D015470	Alitretinoi	37.3	22
PTHLH	5744	Leukemia, MESH:D015470	Alitretinoi	37.3	33
CXCL2	2920	Leukemia, MESH:D015470	Air Polluta	37.29	37
CARHSP1	23589	Leukemia, MESH:D015470	Calcitriol|D	37.27	31
MELK	9833	Leukemia, MESH:D015470	Calcitriol|C	37.27	24
MDC1	9656	Leukemia, MESH:D015470	Air Polluta	37.25	22
NCOA2	10499	Leukemia, MESH:D015473	Arsenic Tr	37.24	269
CCNF	899	Leukemia, MESH:D015470	Arsenic Tr	37.21	45
PML	5371	Leukemia, MESH:D015470	Arsenic Tr	37.21	38
SLC1A5	6510	Leukemia, MESH:D015470	Benzoates	37.21	28
MMP1	4312	Leukemia, MESH:D015473	2-(2-chlor	37.2	267
VLDLR	7436	Leukemia, MESH:D015470	Arsenic Tr	37.17	31
TAGLN	6876	Leukemia, MESH:D015470	Arsenic Tr	37.15	105
TPX2	22974	Leukemia, MESH:D015470	Calcitriol|D	37.11	23
BORA	79866	Leukemia, MESH:D015470	Cytarabine	37.1	89
CASP1	834	Leukemia, MESH:D015473	Aclarubici	37.1	268
EIF4G2	1982	Leukemia, MESH:D015470	Arsenic Tr	37.1	39
CREM	1390	Leukemia, MESH:D015470	Arsenic Tr	37.09	54
TIMP3	7078	Leukemia, MESH:D015470	Arsenic Tr	37.09	104
IL2	3558	Leukemia, MESH:D015473	Arsenic|Ar	37.08	269
LBH	81606	Leukemia, MESH:D015470	Calcitriol|C	37.04	87
METTL7A	25840	Leukemia, MESH:D015470	Air Polluta	37.04	27
PHF5A	84844	Leukemia, MESH:D015470	Arsenic Tr	37.04	21
SLC7A11	23657	Leukemia, MESH:D015470	Arsenic Tr	37.03	58
AXIN2	8313	Leukemia, MESH:D015470	Air Polluta	37.01	36
BMP6	654	Leukemia, MESH:D015470	Air Polluta	37.01	38
KPNA2	3838	Leukemia, MESH:D015470	Arsenic Tr	37.01	98
SRI	6717	Leukemia, MESH:D015473	Arsenic|Ar	37.01	270
ISG15	9636	Leukemia, MESH:D015473	alvocidib|A	36.99	267
HMCN1	83872	Leukemia, MESH:D015470	Calcitriol|D	36.95	23
PRRC2C	23215	Leukemia, MESH:D015470	Arsenic Tr	36.95	91

FST	10468	Leukemia, MESH:D015470		Arsenic Tr	36.93	37
KLF9	687	Leukemia, MESH:D015470		Arsenic Tr	36.93	35
MTUS1	57509	Leukemia, MESH:D015470		Arsenic Tr	36.92	95
RBL1	5933	Leukemia, MESH:D015470		alvocidib|/	36.92	37
ACOX1	51	Leukemia, MESH:D015470		Air Polluta	36.91	33
CHUK	1147	Leukemia, MESH:D015470		15-deoxy-	36.88	37
NRG1	3084	Leukemia, MESH:D015470		Arsenic Tr	36.88	40
ARMC9	80210	Leukemia, MESH:D015470		Arsenic Tr	36.84	23
TRIM38	10475	Leukemia, MESH:D015470		Calcitriol|D	36.84	27
ZC2HC1A	51101	Leukemia, MESH:D015470		Doxorubic	36.84	22
MX1	4599	Leukemia, MESH:D01	marker/me	alvocidib|/	36.82	50
S100A8	6279	Leukemia, MESH:D01	marker/me	Alitretinoi	36.82	40
BMP2	650	Leukemia, MESH:D015470		Arsenic Tr	36.82	41
CDC42	998	Leukemia, MESH:D015470		Arsenic Tr	36.82	38
PRKCB	5579	Leukemia, MESH:D015470		Arsenic Tr	36.82	39
EGFR	1956	Leukemia, MESH:D015473		2-(2-chlor	36.81	269
FASL	14103	Leukemia, MESH:D015470		Arsenic Tr	36.8	34
ITGA1	3672	Leukemia, MESH:D015470		Decitabine	36.8	32
LMCD1	29995	Leukemia, MESH:D015470		Calcitriol|C	36.8	87
UBE2C	11065	Leukemia, MESH:D015473		Antimony	36.8	268
GPT	2875	Leukemia, MESH:D015470		Arsenic Tr	36.75	55
ABCC2	1244	Leukemia, MESH:D015473		Antimony	36.74	273
OCLN	1.01E+08	Leukemia, MESH:D015473		Arsenic|Ar	36.73	271
LGALS1	3956	Leukemia, MESH:D015470		Arsenic Tr	36.7	48
ALAS2	212	Leukemia, MESH:D015470		Arsenic Tr	36.69	46
H2BC21	8349	Leukemia, MESH:D015470		Benzene|D	36.66	38
PRSS12	8492	Leukemia, MESH:D015470		Azacitidine	36.66	21
SH3BGRL	6451	Leukemia, MESH:D015470		Arsenic Tr	36.66	25
ZC3HAV1	56829	Leukemia, MESH:D015470		Benzene|C	36.66	99
MOK	5891	Leukemia, MESH:D015470		Calcitriol|D	36.65	18
BCL2A1	597	Leukemia, MESH:D015470		Arsenic Tr	36.64	48
EIF4EBP1	1978	Leukemia, MESH:D01	marker/me	2-(2-chlor	36.62	58
ABL1	25	Leukemia, MESH:D015473		alvocidib|a	36.62	268
FABP5	2171	Leukemia, MESH:D015470		Alitretinoi	36.62	33
MAP3K5	4217	Leukemia, MESH:D015470		Benzene|C	36.62	69
IL6	3569	Leukemia, MESH:D015473		2-(2-chlor	36.6	282
RPS6	6194	Leukemia, MESH:D015473		alpha-Toc	36.59	159
RUNX1	861	Leukemia, MESH:D01	marker/me	Air Polluta	36.55	49
MT1X	4501	Leukemia, MESH:D015470		Arsenic Tr	36.55	112
NCF2	4688	Leukemia, MESH:D015470		Air Polluta	36.55	65
HDAC1	3065	Leukemia, MESH:D015470		15-deoxy-	36.54	38
PGK1	5230	Leukemia, MESH:D015470		Alitretinoi	36.54	42
SLC40A1	30061	Leukemia, MESH:D015470		Arsenic Tr	36.54	94
ARL4C	10123	Leukemia, MESH:D015470		Arsenic Tr	36.52	24
SQSTM1	8878	Leukemia, MESH:D015473		2-(2-chlor	36.52	270
XAF1	54739	Leukemia, MESH:D015470		Arsenic Tr	36.52	38
HIPK1	204851	Leukemia, MESH:D015473		Arsenic|ars	36.48	271
ARHGDIA	396	Leukemia, MESH:D015470		Bortezomi	36.46	25
CYB5R2	51700	Leukemia, MESH:D015470		Arsenic Tr	36.46	94
DLG1	1739	Leukemia, MESH:D015470		Arsenic Tr	36.46	27
RAN	5901	Leukemia, MESH:D015470		Decitabine	36.45	31
FAM234B	57613	Leukemia, MESH:D015470		Air Polluta	36.39	17
SRI	6717	Leukemia, MESH:D015470		Arsenic Tr	36.38	36
TMEM158	25907	Leukemia, MESH:D015470		Arsenic Tr	36.38	25
AKAP12	9590	Leukemia, MESH:D015470		Arsenic Tr	36.36	44
ELF3	1999	Leukemia, MESH:D015470		Arsenic Tr	36.35	35
MAP2	4133	Leukemia, MESH:D015470		Alitretinoi	36.31	96
N4BP1	9683	Leukemia, MESH:D015470		Arsenic Tr	36.28	25

ACP5	54	Leukemia, MESH:D015470		Calcitriol|D	36.27	28
LEP	3952	Leukemia, MESH:D015470		15-deoxy-	36.27	42
DHFR	1719	Leukemia, MESH:D015473		Antineopl	36.25	163
GPM6B	2824	Leukemia, MESH:D015470		Arsenic Tr	36.24	38
GSPT1	2935	Leukemia, MESH:D015470		Arsenic Tr	36.24	29
PDGFRA	5156	Leukemia, MESH:D015470		6,7-dimetl	36.24	29
UPP1	7378	Leukemia, MESH:D015470		Arsenic Tr	36.18	29
ZNF692	55657	Leukemia, MESH:D015470		Dexameth	36.18	18
RFC4	5984	Leukemia, MESH:D015470		Calcitriol|D	36.13	27
WIPF1	7456	Leukemia, MESH:D015470		Arsenic Tr	36.1	87
ASPM	259266	Leukemia, MESH:D015470		Arsenic Tr	36.09	32
MICB	4277	Leukemia, MESH:D015470		Arsenic Tr	36.09	32
NOS2	4843	Leukemia, MESH:D015473		alpha-Toc	36.06	273
PER2	8864	Leukemia, MESH:D015470		Arsenic Tr	36.02	31
CST3	1471	Leukemia, MESH:D01	marker/m	Arsenic Tr	36	50
PML	5371	Leukemia, MESH:D01	marker/m	Antimony	36	275
CLGN	1047	Leukemia, MESH:D015473		Antimony	36	262
SLC7A8	23428	Leukemia, MESH:D015473		Arsenic|Ar	36	266
TFAP2A	7020	Leukemia, MESH:D015473		arsenite|C	36	162
HSPA1A	3303	Leukemia, MESH:D015470		Air Polluta	35.98	46
ACSL1	2180	Leukemia, MESH:D015470		Arsenic Tr	35.97	43
HTRA2	27429	Leukemia, MESH:D015470		alvocidib|	35.96	27
LTBR	4055	Leukemia, MESH:D015470		Arsenic Tr	35.96	45
MT2A	4502	Leukemia, MESH:D015470		Arsenic Tr	35.95	107
NQO1	1728	Leukemia, MESH:D015473		2-(2-chlor	35.95	274
NDC80	10403	Leukemia, MESH:D015473		Arsenic|Ar	35.92	268
BUB3	9184	Leukemia, MESH:D015470		Cyclophos	35.91	23
FOXP3	50943	Leukemia, MESH:D015470		Arsenic Tr	35.91	44
NOX4	50507	Leukemia, MESH:D015470		Arsenic Tr	35.91	107
ZFP36L1	677	Leukemia, MESH:D015470		Arsenic Tr	35.91	42
KRT19	3880	Leukemia, MESH:D015473		Arsenic Tr	35.9	270
RXRA	6256	Leukemia, MESH:D015473		alpha-Toc	35.89	269
ZFP36	7538	Leukemia, MESH:D015470		Arsenic Tr	35.87	44
HBB	3043	Leukemia, MESH:D015470		Arsenic Tr	35.83	53
NES	10763	Leukemia, MESH:D015470		15-deoxy-	35.83	99
RUNX3	864	Leukemia, MESH:D01	marker/m	Azacitidin	35.82	47
HDAC9	9734	Leukemia, MESH:D015470		Decitabine	35.82	29
KIF4A	24137	Leukemia, MESH:D015470		Calcitriol|D	35.82	22
RAD54L	8438	Leukemia, MESH:D015470		Calcitriol|C	35.82	33
WDHD1	11169	Leukemia, MESH:D015470		Calcitriol|D	35.82	19
PMEPA1	56937	Leukemia, MESH:D015470		Benzene|C	35.81	42
CHEK2	11200	Leukemia, MESH:D015470		Arsenic Tr	35.79	45
TXN	7295	Leukemia, MESH:D015470		Arsenic Tr	35.79	44
ADA	100	Leukemia, MESH:D015470		Benzene|D	35.74	43
PLEKHG3	26030	Leukemia, MESH:D015470		Arsenic Tr	35.74	92
UNG	7374	Leukemia, MESH:D015470		Calcitriol|C	35.74	43
ANXA1	301	Leukemia, MESH:D015470		Alitretinoi	35.72	39
APEX1	328	Leukemia, MESH:D015470		Benzene|B	35.72	69
EP300	2033	Leukemia, MESH:D015470		Arsenic Tr	35.72	43
TXNIP	10628	Leukemia, MESH:D015473		Arsenic|ar	35.71	267
PTEN	5728	Leukemia, MESH:D015473		Arsenic|Ar	35.7	272
SORT1	6272	Leukemia, MESH:D015470		Chloroqui	35.7	31
BMI1	648	Leukemia, MESH:D015470		Arsenic Tr	35.69	30
TAP1	6890	Leukemia, MESH:D015473		Arsenic|Ar	35.69	276
CAPN2	824	Leukemia, MESH:D01	marker/m	Alitretinoi	35.66	29
ERBB2	2064	Leukemia, MESH:D015473		Antineopl	35.66	162
RELB	5971	Leukemia, MESH:D015470		Air Polluta	35.66	46
SMAD7	4092	Leukemia, MESH:D015470		Air Polluta	35.66	31

BMP4	652	Leukemia, MESH:D015470	Busulfan|C	35.65	99
SMCO4	56935	Leukemia, MESH:D015470	Air Polluta	35.64	24
IFITM3	10410	Leukemia, MESH:D015470	Arsenic Tr	35.6	31
IFNGR1	3459	Leukemia, MESH:D015470	Alitretinoi	35.6	36
IL17A	3605	Leukemia, MESH:D015470	Arsenic Tr	35.6	53
NCF1	653361	Leukemia, MESH:D015470	Arsenic Tr	35.6	56
CASP6	839	Leukemia, MESH:D015470	Arsenic Tr	35.58	49
EIF4E	1977	Leukemia, MESH:D015470	Arsenic Tr	35.57	28
IL6R	3570	Leukemia, MESH:D015470	Air Polluta	35.57	69
KIF23	9493	Leukemia, MESH:D015470	Calcitriol|D	35.57	34
IFI35	3430	Leukemia, MESH:D015470	Air Polluta	35.56	21
LOXL4	84171	Leukemia, MESH:D015470	Calcitriol|C	35.56	91
LRATD2	157638	Leukemia, MESH:D015470	Calcitriol|D	35.56	19
PTPRE	5791	Leukemia, MESH:D015470	Arsenic Tr	35.56	44
TCP11L2	255394	Leukemia, MESH:D015470	Dasatinib|I	35.56	23
YPEL5	51646	Leukemia, MESH:D015470	Arsenic Tr	35.56	31
ATM	472	Leukemia, MESH:D015473	Arsenic|Ar	35.49	273
ETHE1	23474	Leukemia, MESH:D015470	Arsenic Tr	35.43	28
KIAA0319I	79932	Leukemia, MESH:D015470	Arsenic Tr	35.43	20
H4-16	121504	Leukemia, MESH:D015470	Alitretinoi	35.41	27
SPOCD1	90853	Leukemia, MESH:D015470	Benzene|D	35.41	35
TGFB1	7040	Leukemia, MESH:D015473	alpha-Toc	35.4	277
ZEB1	6935	Leukemia, MESH:D015473	Arsenic|Ar	35.4	266
CPE	1363	Leukemia, MESH:D015470	Arsenic Tr	35.39	30
MDFIC	29969	Leukemia, MESH:D015470	Calcitriol|C	35.39	27
NFIX	4784	Leukemia, MESH:D015470	Decitabine	35.39	31
SP4	6671	Leukemia, MESH:D015470	Air Polluta	35.39	27
GLUL	2752	Leukemia, MESH:D015470	Alitretinoi	35.36	52
ANXA4	307	Leukemia, MESH:D01 marker/m	Air Polluta	35.32	60
IL11	3589	Leukemia, MESH:D015470	Arsenic Tr	35.32	51
PFKP	5214	Leukemia, MESH:D015470	Arsenic Tr	35.32	91
AREG	374	Leukemia, MESH:D015470	Calcitriol|D	35.3	40
CSTA	1475	Leukemia, MESH:D015470	Arsenic Tr	35.3	29
TNFRSF12	51330	Leukemia, MESH:D015470	Arsenic Tr	35.3	37
UCK2	7371	Leukemia, MESH:D015470	Benzoates	35.3	21
CDC45	8318	Leukemia, MESH:D015470	Arsenic Tr	35.29	36
COL4A2	1284	Leukemia, MESH:D015470	Alitretinoi	35.29	21
DFFA	1676	Leukemia, MESH:D015473	Aclarubici	35.29	271
EDNRB	1910	Leukemia, MESH:D015473	Arsenic|Ar	35.29	269
SLC25A4	291	Leukemia, MESH:D015470	Arsenic Tr	35.29	52
TGFA	7039	Leukemia, MESH:D015473	alpha-Toc	35.29	268
TWIST1	7291	Leukemia, MESH:D015470	Calcitriol|C	35.29	22
DNAJB9	4189	Leukemia, MESH:D015470	Arsenic Tr	35.23	45
WDR76	79968	Leukemia, MESH:D015470	Arsenic Tr	35.23	29
CLDN7	1366	Leukemia, MESH:D015473	Arsenic|Ar	35.19	266
HIPK2	28996	Leukemia, MESH:D015470	Calcitriol|D	35.19	30
MAPKAPK	9261	Leukemia, MESH:D015470	Arsenic Tr	35.19	50
MYD88	4615	Leukemia, MESH:D015470	Arsenic Tr	35.18	45
BAG2	9532	Leukemia, MESH:D015470	Calcitriol|D	35.17	30
KLF11	8462	Leukemia, MESH:D015470	Arsenic Tr	35.17	27
NOL11	25926	Leukemia, MESH:D015470	Dexameth	35.17	18
PRDM16	63976	Leukemia, MESH:D015470	Daunorub	35.17	58
RNF19B	127544	Leukemia, MESH:D015470	Air Polluta	35.17	24
SLC7A8	23428	Leukemia, MESH:D015470	Arsenic Tr	35.17	23
EEF1A1	1915	Leukemia, MESH:D015470	Arsenic Tr	35.15	31
PINK1	65018	Leukemia, MESH:D015470	Arsenic Tr	35.15	35
CYP1A1	1543	Leukemia, MESH:D015473	alpha-Toc	35.14	275
KRAS	3845	Leukemia, MESH:D01 marker/m	Benzene|C	35.12	45

ALDOA	226	Leukemia, MESH:D015473	Antimony	35.12	267
TGFBR2	7048	Leukemia, MESH:D015473	Arsenic|ars	35.12	268
TRIB1	10221	Leukemia, MESH:D015473	Arsenic|ars	35.12	165
SLC2A3	6515	Leukemia, MESH:D015470	Arsenic Tr	35.09	31
RERG	85004	Leukemia, MESH:D015470	Arsenic Tr	35.06	93
IL4R	3566	Leukemia, MESH:D01 marker/m	Arsenic Tr	35.05	49
CCN2	1490	Leukemia, MESH:D015473	Arsenic|Ar	35.05	268
DLK1	8788	Leukemia, MESH:D015470	Arsenic Tr	35.05	31
MAP2K1	5604	Leukemia, MESH:D015473	2-(2-chlor	35.05	265
SET	6418	Leukemia, MESH:D015470	Arsenic Tr	35.05	44
GADD45A	1647	Leukemia, MESH:D015473	alpha-Toc	35.04	273
MAPK9	5601	Leukemia, MESH:D015470	6,7-dimet	35.04	54
TNFRSF21	27242	Leukemia, MESH:D015470	Arsenic Tr	34.99	29
BIRC3	330	Leukemia, MESH:D015473	alvocidib|	34.97	267
OGG1	4968	Leukemia, MESH:D015473	Arsenic|Ar	34.96	271
PRDX2	7001	Leukemia, MESH:D015470	Arsenic Tr	34.95	46
PTGS1	5742	Leukemia, MESH:D015470	15-deoxy-	34.95	37
IGF1R	3480	Leukemia, MESH:D015473	Antineopl	34.93	268
DFFB	1677	Leukemia, MESH:D015470	Arsenic Tr	34.92	33
LXN	56925	Leukemia, MESH:D015470	Calcitriol|D	34.92	38
NUDT7	283927	Leukemia, MESH:D015470	Air Polluta	34.92	30
CYBA	1535	Leukemia, MESH:D015473	Arsenic|Ar	34.91	277
PBK	55872	Leukemia, MESH:D015470	Calcitriol|D	34.91	33
RAD51	5888	Leukemia, MESH:D015473	Arsenic|ars	34.91	165
RFFL	117584	Leukemia, MESH:D015470	Bortezomi	34.9	22
ITPR3	3710	Leukemia, MESH:D015470	Arsenic Tr	34.89	34
RAC2	5880	Leukemia, MESH:D015470	Arsenic Tr	34.89	48
NTRK2	4915	Leukemia, MESH:D015470	Arsenic Tr	34.88	33
TLR2	7097	Leukemia, MESH:D015470	15-deoxy-	34.88	47
ABCC5	10057	Leukemia, MESH:D015470	Arsenic Tr	34.82	40
CCNG1	900	Leukemia, MESH:D015473	Arsenic|Ar	34.81	273
NET1	10276	Leukemia, MESH:D015470	Calcitriol|D	34.8	20
SERPINB5	5268	Leukemia, MESH:D015470	Arsenic Tr	34.8	31
MYL7	58498	Leukemia, MESH:D015470	Calcitriol|C	34.74	93
SLC2A2	6514	Leukemia, MESH:D015470	Alitretinoi	34.74	28
STK4	6789	Leukemia, MESH:D015470	Bortezomi	34.74	22
AIP	9049	Leukemia, MESH:D015470	Alitretinoi	34.73	19
MXRA5	25878	Leukemia, MESH:D015470	Calcitriol|D	34.73	20
SASH3	54440	Leukemia, MESH:D015470	Air Polluta	34.73	22
ALB	213	Leukemia, MESH:D015470	Allopurino	34.71	66
ME1	4199	Leukemia, MESH:D015470	Arsenic Tr	34.71	43
BRCA2	675	Leukemia, MESH:D015473	Arsenic Tr	34.7	271
KIF23	9493	Leukemia, MESH:D015473	Arsenic|ars	34.7	160
CENPU	79682	Leukemia, MESH:D015470	Calcitriol|D	34.68	26
COL1A2	1278	Leukemia, MESH:D015473	Arsenic|Ar	34.68	271
DACT1	51339	Leukemia, MESH:D015470	Arsenic Tr	34.68	31
SPRY1	10252	Leukemia, MESH:D015470	Dexameth	34.68	24
ACHE	43	Leukemia, MESH:D015473	alpha-Toc	34.64	273
AHR	196	Leukemia, MESH:D015470	2-(2-chlor	34.64	55
CALR	811	Leukemia, MESH:D015473	Antimony	34.6	266
EXO1	9156	Leukemia, MESH:D015470	Calcitriol|D	34.59	42
MAP2K3	5606	Leukemia, MESH:D015470	Arsenic Tr	34.59	60
SHC1	6464	Leukemia, MESH:D015470	Arsenic Tr	34.59	46
DNAJA1	3301	Leukemia, MESH:D015470	Alitretinoi	34.58	30
HPGD	3248	Leukemia, MESH:D015470	Arsenic Tr	34.58	27
LIMS1	3987	Leukemia, MESH:D015470	Azacitidin	34.58	25
NR3C1	2908	Leukemia, MESH:D015473	Aclarubici	34.58	271
CDC25B	994	Leukemia, MESH:D015473	Arsenic|Ar	34.57	267

SERPINB2	5055	Leukemia, MESH:D015473	Arsenic Tr	34.57	267
NID2	22795	Leukemia, MESH:D015470	Calcitriol|D	34.56	35
ANXA2	302	Leukemia, MESH:D015473	Arsenic|Ar	34.55	276
TMSB4X	7114	Leukemia, MESH:D015470	Decitabine	34.55	36
MMP3	4314	Leukemia, MESH:D015470	Benzene|C	34.54	43
CYP1A2	1544	Leukemia, MESH:D015470	Anthracyc	34.53	89
IHH	3549	Leukemia, MESH:D015473	Arsenic|Ar	34.53	266
CAPRIN2	65981	Leukemia, MESH:D015470	Air Polluta	34.51	13
CTSK	1513	Leukemia, MESH:D015470	Calcitriol|D	34.51	25
FOXRED2	80020	Leukemia, MESH:D015470	Arsenic Tr	34.51	25
KLF5	688	Leukemia, MESH:D015470	Arsenic Tr	34.51	41
SLC22A5	6584	Leukemia, MESH:D015470	Allopurinc	34.51	32
PPL	5493	Leukemia, MESH:D015470	Calcitriol|D	34.5	22
GADD45G	10912	Leukemia, MESH:D015470	Bortezomi	34.48	43
CREBRF	153222	Leukemia, MESH:D015470	Decitabine	34.44	30
DKK3	27122	Leukemia, MESH:D015470	Arsenic Tr	34.44	38
HMBS	3145	Leukemia, MESH:D015470	Aclarubicii	34.44	26
ECH1	1891	Leukemia, MESH:D015470	Arsenic Tr	34.43	31
HIC1	3090	Leukemia, MESH:D015470	Decitabine	34.42	36
PAMR1	25891	Leukemia, MESH:D015470	Calcitriol|C	34.42	92
SH3D19	152503	Leukemia, MESH:D015470	Arsenic Tr	34.42	26
DMD	1756	Leukemia, MESH:D015470	Daunorub	34.4	59
OLR1	4973	Leukemia, MESH:D015470	Alitretinoi	34.4	99
SERPINF1	5176	Leukemia, MESH:D015470	Calcitriol|D	34.4	22
ODC1	4953	Leukemia, MESH:D015470	Alitretinoi	34.39	32
TIMP2	7077	Leukemia, MESH:D015470	Arsenic Tr	34.37	45
ABCG1	9619	Leukemia, MESH:D015470	Alitretinoi	34.35	27
CDC6	990	Leukemia, MESH:D015470	Arsenic Tr	34.35	39
MTUS1	57509	Leukemia, MESH:D015473	Arsenic|Ar	34.35	268
TGFBI	7045	Leukemia, MESH:D015470	Decitabine	34.35	32
DDIT3	1649	Leukemia, MESH:D015473	2-(2-chlor	34.32	270
ETV4	2118	Leukemia, MESH:D015470	Androgen	34.32	27
FRZB	2487	Leukemia, MESH:D015470	Androgen	34.32	92
FUBP1	8880	Leukemia, MESH:D015470	Arsenic Tr	34.32	28
IL10RA	3587	Leukemia, MESH:D015470	Alitretinoi	34.32	33
CDCA3	83461	Leukemia, MESH:D015470	Calcitriol|D	34.31	32
ROCK1	6093	Leukemia, MESH:D015470	Arsenic Tr	34.31	61
CCNE2	9134	Leukemia, MESH:D015470	Calcitriol|C	34.28	89
GINS2	51659	Leukemia, MESH:D015473	Arsenic|ars	34.27	161
HP	3240	Leukemia, MESH:D015470	Benzene|C	34.27	37
CCNA2	890	Leukemia, MESH:D015473	alvocidib|/	34.25	268
CYBA	1535	Leukemia, MESH:D015470	Arsenic Tr	34.22	106
GEM	2669	Leukemia, MESH:D015470	Calcitriol|C	34.22	22
GPX4	2879	Leukemia, MESH:D015470	Arsenic Tr	34.22	29
CDH3	1001	Leukemia, MESH:D015470	Calcitriol|C	34.2	92
DLC1	10395	Leukemia, MESH:D015470	Arsenic Tr	34.2	33
MTR	4548	Leukemia, MESH:D015470	Arsenic Tr	34.2	28
RFC3	5983	Leukemia, MESH:D015473	arsenite|Ca	34.18	158
ID3	3399	Leukemia, MESH:D015470	Azacitidine	34.15	37
ATP7A	538	Leukemia, MESH:D015470	Carboplati	34.12	42
H2BC12	85236	Leukemia, MESH:D015470	Benzene|D	34.12	36
HELLS	3070	Leukemia, MESH:D015470	Calcitriol|D	34.12	21
HOXA5	3202	Leukemia, MESH:D015470	Alitretinoi	34.12	93
KDM4B	23030	Leukemia, MESH:D015470	Bortezomi	34.12	21
NCOA1	8648	Leukemia, MESH:D015470	Alitretinoi	34.12	22
RAB11FIP1	80223	Leukemia, MESH:D015470	Air Polluta	34.12	27
TMC6	11322	Leukemia, MESH:D015470	Calcitriol|D	34.12	29
CEP41	95681	Leukemia, MESH:D015470	Air Polluta	34.1	19

ATAD2	29028	Leukemia, MESH:D015473	Arsenic|Ar	34.09	267
FLT1	2321	Leukemia, MESH:D015470	Arsenic Tr	34.09	33
GSTP1	2950	Leukemia, MESH:D015473	Aclarubici	34.09	269
PDGFB	5155	Leukemia, MESH:D015470	Alitretinoi	34.09	34
SQLE	6713	Leukemia, MESH:D015470	Air Polluta	34.09	41
BLM	641	Leukemia, MESH:D015470	Air Polluta	34.08	32
COL8A1	1295	Leukemia, MESH:D015470	Calcitriol|D	34.08	22
MAPK13	5603	Leukemia, MESH:D015470	Arsenic Tr	34.08	26
TP73	7161	Leukemia, MESH:D015470	2-(2-chlo	34.08	41
HSPB8	26353	Leukemia, MESH:D015470	Arsenic Tr	34.04	36
TPI1	7167	Leukemia, MESH:D015470	Arsenic Tr	34.04	40
BNIP3L	665	Leukemia, MESH:D015470	Arsenic Tr	34.03	36
GPX3	2878	Leukemia, MESH:D015473	Arsenic Tr	34	265
AHCYL1	10768	Leukemia, MESH:D015470	Air Polluta	33.97	35
BTG3	10950	Leukemia, MESH:D015470	Air Polluta	33.97	33
ENPP2	5168	Leukemia, MESH:D015470	Arsenic Tr	33.97	42
MAP2K6	5608	Leukemia, MESH:D015470	Arsenic Tr	33.97	44
RARRES1	5918	Leukemia, MESH:D015470	Alitretinoi	33.97	24
SLC43A3	29015	Leukemia, MESH:D015470	Bortezomi	33.97	93
ST3GAL1	6482	Leukemia, MESH:D015470	Benzene|C	33.97	104
BRCA1	672	Leukemia, MESH:D015473	Arsenic|Ar	33.95	271
GBP2	2634	Leukemia, MESH:D015470	Azacitidin	33.94	27
NRIP1	8204	Leukemia, MESH:D015470	Azacitidin	33.94	37
TOP1	7150	Leukemia, MESH:D015470	Arsenic Tr	33.94	30
BST2	684	Leukemia, MESH:D015473	Arsenic Tr	33.92	272
DNMT3B	1789	Leukemia, MESH:D015473	Arsenic|ar	33.92	268
FYN	2534	Leukemia, MESH:D015473	alpha-Toc	33.92	168
GRB2	2885	Leukemia, MESH:D015473	Arsenic Tr	33.92	264
MYH10	4628	Leukemia, MESH:D015473	Arsenic|Ar	33.92	270
NPHS1	4868	Leukemia, MESH:D015473	Calcitriol|C	33.92	161
GPX2	2877	Leukemia, MESH:D015473	Antimony	33.9	161
NR1I2	8856	Leukemia, MESH:D015470	Alitretinoi	33.9	47
PLAAT4	5920	Leukemia, MESH:D015470	Alitretinoi	33.9	24
WDR19	57728	Leukemia, MESH:D015470	Air Polluta	33.9	22
HNF4A	3172	Leukemia, MESH:D015470	Alitretinoi	33.89	35
NR1D1	9572	Leukemia, MESH:D015470	Dexameth	33.89	27
FKBP5	2289	Leukemia, MESH:D015473	Arsenic|Ar	33.86	269
H2AC6	8334	Leukemia, MESH:D015470	Arsenic Tr	33.86	28
HSP90B1	7184	Leukemia, MESH:D015473	Arsenic|Ar	33.86	270
RRBP1	6238	Leukemia, MESH:D015470	Arsenic Tr	33.86	26
NEK2	4751	Leukemia, MESH:D015470	Calcitriol|D	33.85	32
PLTP	5360	Leukemia, MESH:D015470	Arsenic Tr	33.85	91
ABCA1	19	Leukemia, MESH:D01 marker/m	Arsenic|Ar	33.84	268
ACTG2	72	Leukemia, MESH:D015473	Arsenic Tr	33.83	263
S100A9	6280	Leukemia, MESH:D015470	Alitretinoi	33.83	40
ATF2	1386	Leukemia, MESH:D015470	Arsenic Tr	33.82	38
MBD2	8932	Leukemia, MESH:D015470	Arsenic Tr	33.82	30
RASGRP2	10235	Leukemia, MESH:D015470	Decitabin	33.82	29
TFRC	7037	Leukemia, MESH:D015470	Arsenic Tr	33.77	42
EIF2AK3	9451	Leukemia, MESH:D015470	Arsenic Tr	33.76	34
MYH6	4624	Leukemia, MESH:D015470	Arsenic Tr	33.76	60
TNFSF11	8600	Leukemia, MESH:D015473	Arsenic|Ar	33.76	267
DPP4	1803	Leukemia, MESH:D015470	Arsenic Tr	33.74	50
FTL	2512	Leukemia, MESH:D015473	Antimony	33.74	269
GAS1	2619	Leukemia, MESH:D015470	Arsenic Tr	33.74	22
ORC6	23594	Leukemia, MESH:D015470	Calcitriol|D	33.74	21
PABPC1L	80336	Leukemia, MESH:D015470	Dexameth	33.7	15
PLCXD1	55344	Leukemia, MESH:D015470	Arsenic Tr	33.7	26

WRN	7486	Leukemia, MESH:D015470		Amsacrine	33.7	45
BCR	613	Leukemia, MESH:D015470		Arsenic Tr	33.68	20
HLA-A	3105	Leukemia, MESH:D015470		Arsenic Tr	33.68	33
POLD3	10714	Leukemia, MESH:D015470		Arsenic Tr	33.68	28
ACSS2	55902	Leukemia, MESH:D015470		Arsenic Tr	33.67	29
MAT2A	4144	Leukemia, MESH:D015470		Air Polluta	33.67	37
PTGES	9536	Leukemia, MESH:D015470		Air Polluta	33.67	34
CDCA7	83879	Leukemia, MESH:D015470		Calcitriol|D	33.63	20
HCK	3055	Leukemia, MESH:D015470		Air Polluta	33.63	24
ORC1	4998	Leukemia, MESH:D015470		Benzene|C	33.63	32
PALLD	23022	Leukemia, MESH:D015470		Bortezomi	33.63	28
RARG	5916	Leukemia, MESH:D015470		Alitretinoi	33.63	32
STARD4	134429	Leukemia, MESH:D015470		Arsenic Tr	33.63	32
STAT4	6775	Leukemia, MESH:D015470		Arsenic Tr	33.63	43
THBD	7056	Leukemia, MESH:D01	marker/m	Arsenic|Ar	33.61	273
AOX1	316	Leukemia, MESH:D015470		Dasatinib|	33.59	24
CSF3	1440	Leukemia, MESH:D01	therapeuti	Air Polluta	33.58	45
ACKR3	57007	Leukemia, MESH:D015473		Arsenic|ars	33.58	160
HNRNPA1	3178	Leukemia, MESH:D015470		Arsenic Tr	33.58	28
PLK4	10733	Leukemia, MESH:D015473		Arsenic Tr	33.58	266
SLC9A3R1	9368	Leukemia, MESH:D015470		Air Polluta	33.58	112
MET	4233	Leukemia, MESH:D01	marker/m	ABT-737|A	33.54	37
MN1	4330	Leukemia, MESH:D01	marker/m	Calcitriol|D	33.54	26
CDCA7L	55536	Leukemia, MESH:D015470		Calcitriol|D	33.54	19
CHRDL1	91851	Leukemia, MESH:D015470		Arsenic Tr	33.54	30
LRRN3	54674	Leukemia, MESH:D015470		Air Polluta	33.54	87
WNT10B	7480	Leukemia, MESH:D015470		15-deoxy-	33.54	19
C1R	715	Leukemia, MESH:D015470		Arsenic Tr	33.52	40
SMPD1	6609	Leukemia, MESH:D015470		Arsenic Tr	33.52	53
TNFRSF11	4982	Leukemia, MESH:D015470		Amsacrine	33.52	43
STXBP4	252983	Leukemia, MESH:D015470		Dexameth	33.5	17
TRIM8	81603	Leukemia, MESH:D015470		Azacitidine	33.5	24
TSPAN31	6302	Leukemia, MESH:D015470		Arsenic Tr	33.5	26
SIGMAR1	10280	Leukemia, MESH:D015470		Air Polluta	33.49	24
AKR1B1	231	Leukemia, MESH:D015470		Arsenic Tr	33.46	54
ZNF738	148203	Leukemia, MESH:D015470		Air Polluta	33.46	15
CAT	847	Leukemia, MESH:D015473		alpha-Toc	33.45	275
CCL2	6347	Leukemia, MESH:D015473		alpha-Toc	33.45	274
RRM2	6241	Leukemia, MESH:D015473		Arsenic Tr	33.43	270
NMU	10874	Leukemia, MESH:D015473		Arsenic|ars	33.42	164
CRABP2	1382	Leukemia, MESH:D015470		Alitretinoi	33.41	30
LY6E	4061	Leukemia, MESH:D015470		Arsenic Tr	33.41	36
RIPK2	8767	Leukemia, MESH:D015470		Benzene|C	33.41	47
SMO	6608	Leukemia, MESH:D015470		Androgen	33.41	29
TP53INP1	94241	Leukemia, MESH:D015470		Arsenic Tr	33.41	31
CNBP	7555	Leukemia, MESH:D015470		Arsenic Tr	33.4	24
PLD3	23646	Leukemia, MESH:D015470		Arsenic Tr	33.4	91
ALPL	249	Leukemia, MESH:D015470		Allopurinc	33.39	47
GFAP	2670	Leukemia, MESH:D015473		alpha-Toc	33.39	167
MAFF	23764	Leukemia, MESH:D015473		Antimony	33.39	270
AHNAK	79026	Leukemia, MESH:D015470		Calcitriol|C	33.32	94
CIZ1	25792	Leukemia, MESH:D015470		Dexameth	33.31	20
GSN	2934	Leukemia, MESH:D015473		arsenic dis	33.31	267
LDLRAP1	26119	Leukemia, MESH:D015470		Calcitriol|D	33.31	20
PDIA3	2923	Leukemia, MESH:D015473		Arsenic|Ar	33.31	268
CXCL5	6374	Leukemia, MESH:D015470		Arsenic Tr	33.3	49
EPB41L3	23136	Leukemia, MESH:D015470		Calcitriol|D	33.3	33
NCAPD2	9918	Leukemia, MESH:D015470		Air Polluta	33.3	19

OLFML3	56944	Leukemia, MESH:D015470		Arsenic Tr	33.3	91
PPM1D	8493	Leukemia, MESH:D015470		Arsenic Tr	33.3	35
PSMB10	5699	Leukemia, MESH:D015470		Calcitriol|D	33.3	20
CHD9	80205	Leukemia, MESH:D015470		Calcitriol|D	33.26	19
IRF7	3665	Leukemia, MESH:D015473		Arsenic|Ar	33.26	270
LHX2	9355	Leukemia, MESH:D015470		Cyclophos	33.26	29
MEIS1	4211	Leukemia, MESH:D015470		Alitretinoi	33.26	99
POLQ	10721	Leukemia, MESH:D015470		Calcitriol|D	33.26	29
CXCL14	9547	Leukemia, MESH:D015470		Alitretinoi	33.23	30
MSH2	4436	Leukemia, MESH:D015470		Air Polluta	33.23	37
PLK3	1263	Leukemia, MESH:D015470		Air Polluta	33.23	52
PRKCE	5581	Leukemia, MESH:D015470		Arsenic Tr	33.23	37
PRNP	5621	Leukemia, MESH:D015470		Arsenic Tr	33.23	110
STC1	6781	Leukemia, MESH:D015470		Arsenic Tr	33.23	35
HSPA9	3313	Leukemia, MESH:D015470		Air Polluta	33.21	35
FLNC	2318	Leukemia, MESH:D015470		Calcitriol|C	33.2	88
CCL5	6352	Leukemia, MESH:D015473		Arsenic|ar	33.18	166
SOD2	6648	Leukemia, MESH:D015473		alpha-Toc	33.18	274
CTNNB1	1499	Leukemia, MESH:D015473		2-(2-chlor	33.16	269
RBM17	84991	Leukemia, MESH:D015470		Arsenic Tr	33.13	44
CYP2C9	1559	Leukemia, MESH:D015470		Bortezomi	33.12	30
FANCG	2189	Leukemia, MESH:D015470		Calcitriol|D	33.12	26
GALM	130589	Leukemia, MESH:D015470		Arsenic Tr	33.12	23
IL10RB	3588	Leukemia, MESH:D015470		Air Polluta	33.12	37
NUP153	9972	Leukemia, MESH:D015470		Arsenic Tr	33.12	28
PPIC	5480	Leukemia, MESH:D015470		Calcitriol|C	33.12	91
CDCA5	113130	Leukemia, MESH:D015470		Calcitriol|D	33.09	21
COL4A1	1282	Leukemia, MESH:D015470		Busulfan|D	33.09	30
FBLN5	10516	Leukemia, MESH:D015470		Arsenic Tr	33.09	31
IRS2	8660	Leukemia, MESH:D015470		Benzene|D	33.09	52
MECP2	4204	Leukemia, MESH:D015470		Arsenic Tr	33.09	47
SLC7A7	9056	Leukemia, MESH:D015470		Calcitriol|C	33.09	85
MAPT	4137	Leukemia, MESH:D015470		Azacitidine	33.07	53
MPO	4353	Leukemia, MESH:D015473		alpha-Toc	33.07	269
PHLDA3	23612	Leukemia, MESH:D015470		Arsenic Tr	33.06	46
SLC16A3	9123	Leukemia, MESH:D015470		Arsenic Tr	33.06	111
ENO2	2026	Leukemia, MESH:D01	marker/m	Azacitidine	33.02	33
CTSD	1509	Leukemia, MESH:D015470		Arsenic Tr	33.02	31
ELOVL6	79071	Leukemia, MESH:D015470		Benzene|B	33.02	33
NOTCH2	4853	Leukemia, MESH:D015473		Arsenic|Ar	33.02	266
P4HA1	5033	Leukemia, MESH:D015473		Arsenic|Ar	33.02	269
TP63	8626	Leukemia, MESH:D015473		Antimony	33.02	264
ABLIM1	3983	Leukemia, MESH:D015470		Arsenic Tr	32.99	39
CNN1	1264	Leukemia, MESH:D015470		Alitretinoi	32.99	25
LAMC1	3915	Leukemia, MESH:D015470		Arsenic Tr	32.99	36
RYR2	6262	Leukemia, MESH:D015470		Arsenic Tr	32.99	62
TNFAIP8	25816	Leukemia, MESH:D015470		Arsenic Tr	32.99	26
CFL2	1073	Leukemia, MESH:D015470		Air Polluta	32.98	21
CPT1A	1374	Leukemia, MESH:D015470		Arsenic Tr	32.98	35
MT1A	4489	Leukemia, MESH:D015470		Arsenic Tr	32.98	128
OASL	8638	Leukemia, MESH:D015470		Calcitriol|D	32.98	21
RAD54B	25788	Leukemia, MESH:D015470		Arsenic Tr	32.98	32
SCG2	7857	Leukemia, MESH:D015470		Arsenic Tr	32.98	30
SLC2A1	6513	Leukemia, MESH:D015470		Arsenic Tr	32.98	98
G0S2	50486	Leukemia, MESH:D015473		Arsenic|Ar	32.97	267
LEPR	3953	Leukemia, MESH:D015470		Allopurinc	32.97	94
PRKCA	5578	Leukemia, MESH:D015470		Arsenic Tr	32.97	47
ACADM	34	Leukemia, MESH:D015470		Air Polluta	32.95	34

ALAS1	211	Leukemia, MESH:D015470		Arsenic Tr	32.95	59
CHP1	11261	Leukemia, MESH:D015470		Benzene|D	32.95	40
TNFSF9	8744	Leukemia, MESH:D015473		Arsenic|Ar	32.94	268
GUSB	2990	Leukemia, MESH:D015470		Benzene|C	32.89	42
LAMC2	3918	Leukemia, MESH:D015470		Arsenic Tr	32.89	25
CD59	966	Leukemia, MESH:D015470		Benzene|C	32.88	41
NRP2	8828	Leukemia, MESH:D015470		Calcitriol|C	32.88	19
HMGA2	8091	Leukemia, MESH:D015473		Arsenic|Ar	32.87	265
HSP90AA1	3320	Leukemia, MESH:D015470		Air Polluta	32.87	44
ADM	133	Leukemia, MESH:D015470		Arsenic Tr	32.86	38
PRNP	5621	Leukemia, MESH:D015473		Arsenic Tr	32.85	271
PTTG1	9232	Leukemia, MESH:D015473		Antimony	32.85	264
HMMR	3161	Leukemia, MESH:D015470		Calcitriol|D	32.81	23
GCLM	2730	Leukemia, MESH:D015470		Air Polluta	32.8	55
GLI1	2735	Leukemia, MESH:D01	marker/m	Arsenic|Ar	32.79	268
EIF4A1	1973	Leukemia, MESH:D015470		Arsenic Tr	32.78	30
SLC27A1	376497	Leukemia, MESH:D015470		Alitretinoi	32.78	27
RTN2	6253	Leukemia, MESH:D015470		Calcitriol|D	32.77	14
EGR2	1959	Leukemia, MESH:D015470		Arsenic Tr	32.74	42
C1S	716	Leukemia, MESH:D015470		Benzene|C	32.72	43
MPZL1	9019	Leukemia, MESH:D015470		Cytarabine	32.72	89
NELL2	4753	Leukemia, MESH:D015470		Calcitriol|C	32.72	93
RGS16	6004	Leukemia, MESH:D015470		Benzoates	32.72	24
RORA	6095	Leukemia, MESH:D015470		Arsenic Tr	32.72	36
RRM1	6240	Leukemia, MESH:D015470		Arsenic Tr	32.72	27
S100P	6286	Leukemia, MESH:D015473		Arsenic|ar	32.72	163
CKB	1152	Leukemia, MESH:D015470		Arsenic Tr	32.68	91
CSF1	1435	Leukemia, MESH:D015470		Azacitidine	32.68	37
MMP13	4322	Leukemia, MESH:D015470		Androgen	32.68	50
MYH10	4628	Leukemia, MESH:D015470		Arsenic Tr	32.68	26
PKM	5315	Leukemia, MESH:D015470		Alitretinoi	32.68	35
ZFP36L2	678	Leukemia, MESH:D015470		Dexameth	32.68	19
S100A10	6281	Leukemia, MESH:D01	marker/m	Arsenic Tr	32.64	111
FADS1	3992	Leukemia, MESH:D015470		Benzene|B	32.64	41
IFNG	3458	Leukemia, MESH:D01	marker/m	alpha-Toc	32.63	273
ALAS1	211	Leukemia, MESH:D015473		alpha-Toc	32.63	269
ZNF787	126208	Leukemia, MESH:D015470		Bortezomi	32.63	15
SLC3A2	6520	Leukemia, MESH:D015470		Arsenic Tr	32.62	36
ITPR1	3708	Leukemia, MESH:D015470		Arsenic Tr	32.61	35
NOS3	4846	Leukemia, MESH:D015473		alpha-Toc	32.61	269
OAT	4942	Leukemia, MESH:D015470		Benzene|D	32.61	46
BCL7A	605	Leukemia, MESH:D015470		Arsenic Tr	32.59	23
IGF2BP3	10643	Leukemia, MESH:D015470		Cyclophos	32.59	28
ISLR	3671	Leukemia, MESH:D015470		Dexameth	32.59	24
LSAMP	4045	Leukemia, MESH:D015470		Arsenic Tr	32.59	51
NOC2L	26155	Leukemia, MESH:D015470		Dexameth	32.59	20
PMS1	5378	Leukemia, MESH:D015470		Cyclophos	32.59	23
TAP2	6891	Leukemia, MESH:D015470		Air Polluta	32.59	46
TROAP	10024	Leukemia, MESH:D015470		Calcitriol|D	32.59	19
ACTG2	72	Leukemia, MESH:D015470		Alitretinoi	32.57	31
FSTL1	11167	Leukemia, MESH:D015470		Decitabine	32.57	29
HBP1	26959	Leukemia, MESH:D015470		Air Polluta	32.57	32
MAP2K3	5606	Leukemia, MESH:D015473		Arsenic Tr	32.57	267
SERPINE1	5054	Leukemia, MESH:D015473		Antimony	32.57	273
TXNRD1	7296	Leukemia, MESH:D015470		Arsenic Tr	32.57	31
PROM1	8842	Leukemia, MESH:D015470		Arsenic Tr	32.56	56
BAK1	578	Leukemia, MESH:D015473		Arsenic Tr	32.54	268
CD14	929	Leukemia, MESH:D015473		Arsenic|Ar	32.54	268

CDX1	1044	Leukemia, MESH:D015470		Arsenic Tr	32.54	24
GYG2	8908	Leukemia, MESH:D015470		Cytarabine	32.54	83
IRS1	3667	Leukemia, MESH:D015473		Arsenic|Ar	32.54	267
TGFB2	7042	Leukemia, MESH:D015473		arsenite|ca	32.54	164
INPP4B	8821	Leukemia, MESH:D015473		Arsenic|Ca	32.53	165
FOXO3	2309	Leukemia, MESH:D015470		Arsenic Tr	32.51	37
ITGA3	3675	Leukemia, MESH:D015470		Calcitriol|D	32.47	30
PPARG	5468	Leukemia, MESH:D01	marker/m	alpha-Toc	32.46	274
ARF1	375	Leukemia, MESH:D015470		Arsenic Tr	32.46	23
CDC34	997	Leukemia, MESH:D015470		Arsenic Tr	32.46	23
SLC44A1	23446	Leukemia, MESH:D015470		Arsenic Tr	32.46	23
TCF12	6938	Leukemia, MESH:D015470		Calcitriol|C	32.46	87
XRCC2	7516	Leukemia, MESH:D015470		Calcitriol|D	32.46	31
ENO1	2023	Leukemia, MESH:D015470		Decitabine	32.45	40
TCF12	6938	Leukemia, MESH:D015473		Arsenic|ars	32.43	164
DMD	1756	Leukemia, MESH:D015473		arsenite|D	32.42	163
MED13	9969	Leukemia, MESH:D015470		Calcitriol|D	32.42	19
SKA3	221150	Leukemia, MESH:D015470		Calcitriol|D	32.42	16
ZHX3	23051	Leukemia, MESH:D015470		Arsenic Tr	32.42	25
CCND2	894	Leukemia, MESH:D015473		alpha-Toc	32.41	268
PIK3R1	5295	Leukemia, MESH:D015473		Antimony	32.41	270
TPM4	7171	Leukemia, MESH:D015470		Arsenic Tr	32.4	35
CDCA2	157313	Leukemia, MESH:D015470		Benzene|C	32.37	40
ZBTB20	26137	Leukemia, MESH:D015470		Daunorub	32.37	46
GOT1	2805	Leukemia, MESH:D015470		Air Polluta	32.34	29
MTOR	2475	Leukemia, MESH:D015473		Arsenic|ars	32.34	175
PRKCD	5580	Leukemia, MESH:D015473		Arsenic Tr	32.33	272
SON	6651	Leukemia, MESH:D015470		Arsenic Tr	32.33	42
FGR	2268	Leukemia, MESH:D015473		Arsenic Tr	32.32	266
GDF15	9518	Leukemia, MESH:D015473		Arsenic|Ar	32.3	273
HLA-DRA	3122	Leukemia, MESH:D015473		Arsenic|Ar	32.3	267
ZSCAN31	64288	Leukemia, MESH:D015470		Arsenic Tr	32.3	86
ACKR3	57007	Leukemia, MESH:D015470		Calcitriol|C	32.28	20
SLC7A2	6542	Leukemia, MESH:D015470		Calcitriol|C	32.28	85
ABCB1B	18669	Leukemia, MESH:D015470		Dexameth	32.27	27
SDC1	6382	Leukemia, MESH:D015473		Arsenic|Ar	32.27	268
ZWINT	11130	Leukemia, MESH:D015473		Antimony	32.27	264
IL12B	3593	Leukemia, MESH:D015473		Arsenic|Ar	32.26	267
GJB2	2706	Leukemia, MESH:D015470		Calcitriol|C	32.24	17
H4C3	8364	Leukemia, MESH:D015473		Antimony	32.22	269
ANXA11	311	Leukemia, MESH:D015470		Arsenic Tr	32.21	23
ARHGAP5	394	Leukemia, MESH:D015470		Arsenic Tr	32.21	29
BCOR	54880	Leukemia, MESH:D015470		Decitabine	32.21	29
SCARA3	51435	Leukemia, MESH:D015470		Arsenic Tr	32.21	27
SRPX	8406	Leukemia, MESH:D015470		Arsenic Tr	32.21	27
IKBKB	3551	Leukemia, MESH:D015473		alpha-Toc	32.2	269
TK1	7083	Leukemia, MESH:D015473		Arsenic Tr	32.19	267
GTSE1	51512	Leukemia, MESH:D015470		Calcitriol|D	32.18	21
LAMB3	3914	Leukemia, MESH:D015470		Arsenic Tr	32.18	25
ACAA2	10449	Leukemia, MESH:D015470		15-deoxy-	32.16	29
CAV1	857	Leukemia, MESH:D015473		2-(2-chlor	32.16	266
PCNA	5111	Leukemia, MESH:D015473		alpha-Toc	32.16	273
TH	7054	Leukemia, MESH:D015470		15-deoxy-	32.15	102
TH	7054	Leukemia, MESH:D015473		alpha-Toc	32.13	271
ATP2A3	489	Leukemia, MESH:D015473		Arsenic|Ar	32.12	267
CD9	928	Leukemia, MESH:D01	marker/m	Arsenic Tr	32.08	46
CAV2	858	Leukemia, MESH:D015470		Arsenic Tr	32.08	27
CD47	961	Leukemia, MESH:D015470		Benzene|D	32.08	36

DCTD	1635	Leukemia, MESH:D015470		Arsenic Tr	32.08	23
DEPP1	11067	Leukemia, MESH:D015470		Calcitriol|D	32.08	23
DUSP5	1847	Leukemia, MESH:D015470		Arsenic Tr	32.08	27
FOXM1	2305	Leukemia, MESH:D015470		Arsenic Tr	32.08	29
HSD11B2	3291	Leukemia, MESH:D015470		Calcitriol|D	32.08	28
LCN2	3934	Leukemia, MESH:D015473		Arsenic Tr	32.08	262
LDB3	11155	Leukemia, MESH:D015470		Air Polluta	32.08	53
NPPA	4878	Leukemia, MESH:D015470		2-(2-chlor	32.08	54
NR1H3	10062	Leukemia, MESH:D015470		Alitretinoi	32.08	43
NUCB2	4925	Leukemia, MESH:D015470		Arsenic Tr	32.08	26
PCYOX1	51449	Leukemia, MESH:D015470		Air Polluta	32.08	33
PKNOX2	63876	Leukemia, MESH:D015470		Arsenic Tr	32.08	27
POLE2	5427	Leukemia, MESH:D015470		Calcitriol|D	32.08	19
TAPBP	6892	Leukemia, MESH:D015470		Air Polluta	32.08	19
WNT5A	7474	Leukemia, MESH:D015470		Azacitidine	32.08	32
DUSP10	11221	Leukemia, MESH:D015473		Arsenic|Ar	32.06	272
NHLRC3	387921	Leukemia, MESH:D015470		Decitabine	32.06	24
CD68	968	Leukemia, MESH:D015473		Arsenic|Bu	32.03	164
KIF4A	24137	Leukemia, MESH:D015473		Arsenic|ars	32.02	159
MATN2	4147	Leukemia, MESH:D015473		Arsenic|Ar	32.02	267
DNMT3A	1788	Leukemia, MESH:D01	marker/m	Alitretinoi	32.01	52
EIF4EBP1	1978	Leukemia, MESH:D015473		2-(2-chlor	32	272
MARCKS	4082	Leukemia, MESH:D015470		Air Polluta	32	30
WRN	7486	Leukemia, MESH:D015473		Arsenic|Ar	31.99	266
TUBB	203068	Leukemia, MESH:D015470		Dexameth	31.98	20
MOCOS	55034	Leukemia, MESH:D015470		Arsenic Tr	31.96	41
MSX2	4488	Leukemia, MESH:D015470		Antifungal	31.96	27
NAV1	89796	Leukemia, MESH:D015470		Calcitriol|D	31.96	25
PTPRG	5793	Leukemia, MESH:D015470		Decitabine	31.96	29
CYP2C8	1558	Leukemia, MESH:D015470		Bezafibrat	31.95	24
INHBA	3624	Leukemia, MESH:D015470		Arsenic Tr	31.95	36
COL1A1	1277	Leukemia, MESH:D015473		alpha-Toc	31.93	269
IL6ST	3572	Leukemia, MESH:D015470		Arsenic Tr	31.93	33
AGR2	10551	Leukemia, MESH:D015473		Arsenic|Ar	31.92	267
DHRS3	9249	Leukemia, MESH:D015473		Arsenic|ars	31.92	165
LYN	4067	Leukemia, MESH:D015473		alpha-Toc	31.92	165
MYH6	4624	Leukemia, MESH:D015473		Arsenic Tr	31.92	266
RCAN1	1827	Leukemia, MESH:D015473		Arsenic|ars	31.92	161
RHOQ	23433	Leukemia, MESH:D015473		Arsenic|Ar	31.92	269
SERPINB1	1992	Leukemia, MESH:D015473		Antimony	31.92	266
TRMT1	55621	Leukemia, MESH:D015470		Arsenic Tr	31.92	22
ERO1A	30001	Leukemia, MESH:D015470		Calcitriol|D	31.89	21
HDAC2	3066	Leukemia, MESH:D015470		15-deoxy-	31.89	31
IKBKG	8517	Leukemia, MESH:D015470		Arsenic Tr	31.89	34
NPC1	4864	Leukemia, MESH:D015470		Arsenic Tr	31.89	67
SNAI2	6591	Leukemia, MESH:D015473		Arsenic|Ar	31.88	270
ZNF282	8427	Leukemia, MESH:D015470		Cytarabine	31.88	84
CD36	948	Leukemia, MESH:D015473		alpha-Toc	31.86	270
ALDH1A3	220	Leukemia, MESH:D015470		Arsenic Tr	31.85	28
CYP7A1	1581	Leukemia, MESH:D015470		Alitretinoi	31.84	24
NFE2L3	9603	Leukemia, MESH:D015470		Arsenic Tr	31.84	41
OIP5	11339	Leukemia, MESH:D015470		Calcitriol|D	31.84	21
RFC5	5985	Leukemia, MESH:D015470		Dasatinib|	31.84	21
SERPINB1	1992	Leukemia, MESH:D015470		Arsenic Tr	31.84	26
SIPA1L2	57568	Leukemia, MESH:D015470		Calcitriol|C	31.84	88
COL21A1	81578	Leukemia, MESH:D015470		Arsenic Tr	31.83	22
FAM149B1	317662	Leukemia, MESH:D015470		Decitabine	31.83	23
INHBA	3624	Leukemia, MESH:D015473		Arsenic|Ar	31.83	267

MBP	4155	Leukemia, MESH:D015473	alpha-Toc	31.83	270
TNFRSF10	8794	Leukemia, MESH:D015470	Arsenic Tr	31.83	24
CDK4	1019	Leukemia, MESH:D015473	alvocidib|/	31.8	266
CREB1	1385	Leukemia, MESH:D015473	alpha-Toc	31.79	271
FABP7	2173	Leukemia, MESH:D015470	Arsenic Tr	31.79	92
HNRNPK	3190	Leukemia, MESH:D015470	Arsenic Tr	31.79	26
PDLIM1	9124	Leukemia, MESH:D015470	Decitabine	31.79	28
WARS1	7453	Leukemia, MESH:D015470	Arsenic Tr	31.79	34
AK5	26289	Leukemia, MESH:D015470	Azacitidine	31.76	14
GCLM	2730	Leukemia, MESH:D015473	alpha-Toc	31.76	268
HAND2	9464	Leukemia, MESH:D015470	Cytarabine	31.76	97
VPS13B	157680	Leukemia, MESH:D015470	Dexameth	31.76	20
VPS13D	55187	Leukemia, MESH:D015470	Dexameth	31.76	16
ZCCHC2	54877	Leukemia, MESH:D015470	Benzene|B	31.76	35
PLA2G4A	5321	Leukemia, MESH:D015470	Arsenic Tr	31.75	45
HHIP	64399	Leukemia, MESH:D015470	Calcitriol|D	31.72	25
SLC25A1	6576	Leukemia, MESH:D015470	Decitabine	31.72	28
CD55	1604	Leukemia, MESH:D015470	Arsenic Tr	31.7	47
FBXO5	26271	Leukemia, MESH:D015470	Calcitriol|D	31.7	23
HSPA1L	3305	Leukemia, MESH:D015470	Arsenic Tr	31.7	26
ABCB1A	18671	Leukemia, MESH:D015470	Bortezomi	31.68	48
RUNX2	860	Leukemia, MESH:D015473	Arsenic|Ar	31.68	271
PRDX2	7001	Leukemia, MESH:D015473	Arsenic|Ar	31.67	269
LUM	4060	Leukemia, MESH:D015473	Calcitriol|C	31.65	160
DDIT4	54541	Leukemia, MESH:D015473	Arsenic Tr	31.64	265
RRAS	6237	Leukemia, MESH:D015473	Arsenic|Ar	31.63	267
APC	324	Leukemia, MESH:D015470	Air Polluta	31.62	22
E2F8	79733	Leukemia, MESH:D015470	Arsenic Tr	31.62	95
P4HB	5034	Leukemia, MESH:D015470	Allopurinc	31.62	30
PAWR	5074	Leukemia, MESH:D015470	alvocidib|/	31.61	21
PCLAF	9768	Leukemia, MESH:D015470	Calcitriol|D	31.61	17
SFRP2	6423	Leukemia, MESH:D015470	Arsenic Tr	31.61	97
SRSF7	6432	Leukemia, MESH:D015470	Arsenic Tr	31.61	27
ADAM19	8728	Leukemia, MESH:D015470	Calcitriol|C	31.6	91
BCL2L12	83596	Leukemia, MESH:D015470	Arsenic Tr	31.6	26
CDC14B	8555	Leukemia, MESH:D015470	Air Polluta	31.6	21
CMIP	80790	Leukemia, MESH:D015470	Arsenic Tr	31.6	49
GCNT2	2651	Leukemia, MESH:D015470	Azacitidine	31.6	86
VGLL4	9686	Leukemia, MESH:D015470	Arsenic Tr	31.6	89
GPX1	2876	Leukemia, MESH:D015473	Arsenic|Ar	31.57	275
CFB	629	Leukemia, MESH:D015470	Arsenic Tr	31.55	40
PLD1	5337	Leukemia, MESH:D015470	Benzene|D	31.55	42
ZNF397	84307	Leukemia, MESH:D015470	Dexameth	31.53	16
ATP1A2	477	Leukemia, MESH:D015470	Calcitriol|D	31.52	62
COTL1	23406	Leukemia, MESH:D015470	Arsenic Tr	31.52	25
HMGB1	3146	Leukemia, MESH:D015473	Arsenic Tr	31.52	169
SLC2A2	6514	Leukemia, MESH:D015473	Arsenic Tr	31.52	264
SOCS3	9021	Leukemia, MESH:D015470	15-deoxy-	31.52	50
VIM	7431	Leukemia, MESH:D015473	Arsenic|Ar	31.52	275
ALDH1A1	216	Leukemia, MESH:D015473	Arsenic|Ar	31.51	268
HSD17B2	3294	Leukemia, MESH:D015473	Antimony	31.51	266
EMP3	2014	Leukemia, MESH:D015470	Arsenic Tr	31.48	38
LPXN	9404	Leukemia, MESH:D015470	Air Polluta	31.48	33
NAV3	89795	Leukemia, MESH:D015470	Arsenic Tr	31.48	23
PERP	64065	Leukemia, MESH:D015470	alvocidib|D	31.48	29
RANBP1	5902	Leukemia, MESH:D015470	Doxorubic	31.48	21
RBBP8	5932	Leukemia, MESH:D015470	Calcitriol|D	31.48	32
SRXN1	140809	Leukemia, MESH:D015473	Antimony	31.48	268

CD74	972	Leukemia, MESH:D015470		Arsenic Tr	31.47	41
STC2	8614	Leukemia, MESH:D015470		Arsenic Tr	31.47	33
BCL2A1	597	Leukemia, MESH:D015473		Arsenic Tr	31.45	267
TCEA2	6919	Leukemia, MESH:D01	marker/m	Calcitriol|D	31.44	28
DMXL2	23312	Leukemia, MESH:D015470		Air Polluta	31.44	20
GEMIN4	50628	Leukemia, MESH:D015470		Calcitriol|D	31.44	19
LGALS3	3958	Leukemia, MESH:D015470		Arsenic Tr	31.44	41
MTFR2	113115	Leukemia, MESH:D015470		Calcitriol|D	31.44	17
TMEM106	79022	Leukemia, MESH:D015470		Dexameth	31.44	21
LCK	3932	Leukemia, MESH:D015470		Crizotinib|	31.42	16
NR2F2	7026	Leukemia, MESH:D015470		Arsenic Tr	31.42	25
KITLG	4254	Leukemia, MESH:D015470		Arsenic Tr	31.4	67
MTTP	4547	Leukemia, MESH:D015470		Alitretinoi	31.4	17
NGF	4803	Leukemia, MESH:D015473		Arsenic|ars	31.4	163
NGFR	4804	Leukemia, MESH:D015470		Calcitriol|C	31.4	29
PCK1	5105	Leukemia, MESH:D015473		alpha-Toc	31.4	265
SERPINA1	5265	Leukemia, MESH:D015470		Arsenic Tr	31.4	98
BIVM	54841	Leukemia, MESH:D015470		Dexameth	31.38	18
DACT3	147906	Leukemia, MESH:D015470		Calcitriol|D	31.38	22
ISG20L2	81875	Leukemia, MESH:D015470		Doxorubic	31.38	24
TBX6	6911	Leukemia, MESH:D015470		Arsenic Tr	31.38	20
YDJC	150223	Leukemia, MESH:D015470		Dexameth	31.38	18
AFF4	27125	Leukemia, MESH:D015470		Arsenic Tr	31.36	26
DDR1	780	Leukemia, MESH:D015470		Calcitriol|D	31.36	26
MR1	3140	Leukemia, MESH:D015470		Arsenic Tr	31.36	34
PCOLCE	5118	Leukemia, MESH:D015470		Arsenic Tr	31.36	26
PPT1	5538	Leukemia, MESH:D015470		Chloroqui	31.36	19
PRDX1	5052	Leukemia, MESH:D015470		Air Polluta	31.36	39
SCPEP1	59342	Leukemia, MESH:D015470		Arsenic Tr	31.36	30
GADD45G	10912	Leukemia, MESH:D015473		Calcitriol|C	31.33	158
IL13RA1	3597	Leukemia, MESH:D015470		Benzene|C	31.33	41
LAMA3	3909	Leukemia, MESH:D015470		Alitretinoi	31.33	24
CPT1B	1375	Leukemia, MESH:D015470		Arsenic Tr	31.32	30
HSD17B4	3295	Leukemia, MESH:D015470		Air Polluta	31.32	32
MCM4	4173	Leukemia, MESH:D015470		Arsenic Tr	31.32	36
PRLR	5618	Leukemia, MESH:D015470		Benzene|C	31.32	37
ABCB4	5244	Leukemia, MESH:D015470		Arsenic Tr	31.31	33
NR1I2	8856	Leukemia, MESH:D015473		alpha-Toc	31.3	161
GFI1	2672	Leukemia, MESH:D01	marker/m	Benzene|C	31.29	39
PAK1IP1	55003	Leukemia, MESH:D015470		Calcitriol|D	31.29	17
SOX9	6662	Leukemia, MESH:D015470		Azacitidin	31.26	31
COL3A1	1281	Leukemia, MESH:D015473		arsenite|Ca	31.25	162
CSRP3	8048	Leukemia, MESH:D015470		Arsenic Tr	31.25	27
FOSL2	2355	Leukemia, MESH:D015470		Androgen	31.25	44
HSPE1	3336	Leukemia, MESH:D015470		Calcitriol|C	31.25	30
KIF2C	11004	Leukemia, MESH:D015473		arsenite|Ca	31.25	158
LBR	3930	Leukemia, MESH:D015470		Air Polluta	31.25	19
PAPPA	5069	Leukemia, MESH:D015470		Arsenic Tr	31.25	25
SIRPA	140885	Leukemia, MESH:D015470		Arsenic Tr	31.25	35
SPC24	147841	Leukemia, MESH:D015470		Calcitriol|C	31.25	18
TP53I3	9540	Leukemia, MESH:D015473		Calcitriol|D	31.25	161
WNT3A	89780	Leukemia, MESH:D015470		Decitabine	31.25	30
E2F2	1870	Leukemia, MESH:D015470		Arsenic Tr	31.24	29
RHOC	389	Leukemia, MESH:D015470		Arsenic Tr	31.24	98
TYMP	1890	Leukemia, MESH:D015470		Arsenic Tr	31.24	28
CD86	942	Leukemia, MESH:D015473		Arsenic|Ar	31.21	268
SCARB1	949	Leukemia, MESH:D015470		Alitretinoi	31.2	20
ATP1B1	481	Leukemia, MESH:D01	marker/m	Alitretinoi	31.18	100

IL24	11009	Leukemia,	MESH:D015470	Alitretinoi	31.18	34
MGP	4256	Leukemia,	MESH:D015470	Arsenic Tr	31.18	92
SKP2	6502	Leukemia,	MESH:D015473	Arsenic|Ar	31.18	272
UCHL1	7345	Leukemia,	MESH:D015470	Arsenic Tr	31.18	43
AR	367	Leukemia,	MESH:D015473	Antimony	31.15	269
C1QA	712	Leukemia,	MESH:D015470	Cyclophos	31.15	24
FGF7	2252	Leukemia,	MESH:D015470	Androgen	31.15	30
HNRNPA2	3181	Leukemia,	MESH:D015470	Alitretinoi	31.15	34
KRT5	3852	Leukemia,	MESH:D015470	Alitretinoi	31.15	23
OGDH	4967	Leukemia,	MESH:D015470	Air Polluta	31.15	24
RACGAP1	29127	Leukemia,	MESH:D015470	Arsenic Tr	31.15	26
TNFRSF19	55504	Leukemia,	MESH:D015470	Calcitriol|C	31.15	84
ANKH	56172	Leukemia,	MESH:D015470	Benzene|C	31.14	33
APLP2	334	Leukemia,	MESH:D015470	Air Polluta	31.14	29
ARHGAP1	93663	Leukemia,	MESH:D015470	Arsenic Tr	31.14	40
BCL2L2	599	Leukemia,	MESH:D015470	ABT-737|A	31.14	30
CPA4	51200	Leukemia,	MESH:D015470	Calcitriol|D	31.14	13
EIF5A2	56648	Leukemia,	MESH:D015470	Arsenic Tr	31.14	22
GPC6	10082	Leukemia,	MESH:D015470	Arsenic Tr	31.14	29
NANOS1	340719	Leukemia,	MESH:D015470	Cyclophos	31.14	14
NCK1	4690	Leukemia,	MESH:D015470	Decitabine	31.14	21
PPP1R13L	10848	Leukemia,	MESH:D015470	Daunorub	31.14	48
PRKAR2B	5577	Leukemia,	MESH:D015470	Dexameth	31.14	19
ZMYM2	7750	Leukemia,	MESH:D015470	Arsenic Tr	31.14	23
ADIPOQ	9370	Leukemia,	MESH:D015470	15-deoxy-	31.13	112
LSAMP	4045	Leukemia,	MESH:D015473	Arsenic|Ar	31.12	268
ORM1	5004	Leukemia,	MESH:D015470	Calcitriol|D	31.1	28
CCNA1	8900	Leukemia,	MESH:D015470	Arsenic Tr	31.07	32
KCNJ2	3759	Leukemia,	MESH:D015473	Arsenic|Ar	31.07	270
NID2	22795	Leukemia,	MESH:D015473	arsenite|Ca	31.07	13
PODXL	5420	Leukemia,	MESH:D015470	Arsenic Tr	31.07	91
SLC19A1	6573	Leukemia,	MESH:D015470	Decitabine	31.07	28
SIRT1	23411	Leukemia,	MESH:D015470	Arsenic Tr	31.05	34
BTG1	694	Leukemia,	MESH:D015470	Arsenic Tr	31.03	43
FBXO32	114907	Leukemia,	MESH:D015470	Arsenic Tr	31.03	28
CBX5	23468	Leukemia,	MESH:D015470	Doxorubic	31.02	23
CKAP4	10970	Leukemia,	MESH:D015470	Arsenic Tr	31.02	26
LAMA1	284217	Leukemia,	MESH:D015470	Azacitidine	31.02	28
LRP8	7804	Leukemia,	MESH:D015470	Dexameth	31.02	22
NDRG4	65009	Leukemia,	MESH:D015470	Arsenic Tr	31.02	30
NFE2L1	4779	Leukemia,	MESH:D015470	Arsenic Tr	31.02	35
SDCBP	6386	Leukemia,	MESH:D015470	Arsenic Tr	31.02	23
SEMA6D	80031	Leukemia,	MESH:D015470	Arsenic Tr	31.02	24
SULT1C2	6819	Leukemia,	MESH:D015470	Arsenic Tr	31.02	23
TPR	7175	Leukemia,	MESH:D015470	Dexameth	31.02	20
STMN1	3925	Leukemia,	MESH:D015470	Androgen	31.01	37
DHX15	1665	Leukemia,	MESH:D01 marker/m	Air Polluta	30.99	23
CST3	1471	Leukemia,	MESH:D015473	Arsenic|Ar	30.99	268
EVA1A	84141	Leukemia,	MESH:D015470	Calcitriol|D	30.99	17
NETO2	81831	Leukemia,	MESH:D015470	Arsenic Tr	30.99	22
ANLN	54443	Leukemia,	MESH:D015470	Benzene|C	30.98	36
EGF	1950	Leukemia,	MESH:D015473	2-(2-chlor	30.98	269
STOM	2040	Leukemia,	MESH:D015473	Arsenic|ars	30.98	163
XRCC6	2547	Leukemia,	MESH:D015473	alpha-Toc	30.98	175
H3-3B	3021	Leukemia,	MESH:D015470	Doxorubic	30.96	18
ZNF148	7707	Leukemia,	MESH:D015470	Dexameth	30.96	18
CEBPA	1050	Leukemia,	MESH:D01 marker/m	Antimony	30.95	268
HSPD1	3329	Leukemia,	MESH:D015473	Arsenic|Ar	30.95	269

MTOR	2475	Leukemia,	MESH:D015470	15-deoxy-	30.95	59
TAGLN	6876	Leukemia,	MESH:D015473	Arsenic|Ar	30.94	273
GFAP	2670	Leukemia,	MESH:D015470	15-deoxy-	30.92	38
H1-2	3006	Leukemia,	MESH:D01 marker/m	Arsenic Tr	30.91	96
ITGAX	3687	Leukemia,	MESH:D015470	Alitretinoi	30.91	27
TYROBP	7305	Leukemia,	MESH:D015470	Calcitriol|C	30.91	25
BCAT1	586	Leukemia,	MESH:D015470	Azacitidine	30.89	33
CCND3	896	Leukemia,	MESH:D015473	Arsenic|Ar	30.89	272
IL15	3600	Leukemia,	MESH:D015470	Arsenic Tr	30.89	22
LIFR	3977	Leukemia,	MESH:D015470	Calcitriol|D	30.89	21
PTPRF	5792	Leukemia,	MESH:D015470	Benzene|C	30.89	35
SOCS2	8835	Leukemia,	MESH:D015470	Alitretinoi	30.89	27
TIMP3	7078	Leukemia,	MESH:D015473	Arsenic Tr	30.89	265
C1ORF43	25912	Leukemia,	MESH:D015470	Bortezomi	30.88	15
LINC0031	29931	Leukemia,	MESH:D015470	Cytarabine	30.88	77
ZNF580	51157	Leukemia,	MESH:D015470	Bortezomi	30.88	17
CLCN5	1184	Leukemia,	MESH:D015470	Dexameth	30.84	18
MLF1	4291	Leukemia,	MESH:D015470	Air Polluta	30.84	18
MYO5C	55930	Leukemia,	MESH:D015470	Arsenic Tr	30.84	17
NDUFS8	4728	Leukemia,	MESH:D015470	Arsenic Tr	30.84	32
PTK2	5747	Leukemia,	MESH:D015470	Benzene|D	30.84	35
STS	412	Leukemia,	MESH:D015470	Alitretinoi	30.82	26
EZH2	2146	Leukemia,	MESH:D015473	Arsenic|Ar	30.81	265
GBP1	2633	Leukemia,	MESH:D015473	Arsenic|Ca	30.81	158
NFKB1	4790	Leukemia,	MESH:D015473	alpha-Toc	30.81	267
PKP2	5318	Leukemia,	MESH:D015473	arsenite|Ca	30.81	160
GABARAP	23710	Leukemia,	MESH:D015470	Air Polluta	30.8	106
NTRK1	4914	Leukemia,	MESH:D015470	Arsenic Tr	30.8	20
PPFIBP1	8496	Leukemia,	MESH:D015470	Calcitriol|D	30.8	20
RND1	27289	Leukemia,	MESH:D015470	Calcitriol|C	30.8	29
TSC22D3	1831	Leukemia,	MESH:D015470	Arsenic Tr	30.78	35
F3	2152	Leukemia,	MESH:D01 marker/m	Arsenic|Ar	30.76	274
ACBD7	414149	Leukemia,	MESH:D015470	Calcitriol|D	30.75	11
APOBEC3	27350	Leukemia,	MESH:D015470	Doxorubic	30.75	33
COX10	1352	Leukemia,	MESH:D015470	Arsenic Tr	30.75	22
KRT8	3856	Leukemia,	MESH:D015473	Arsenic|Ar	30.75	271
PTPDC1	138639	Leukemia,	MESH:D015470	Arsenic Tr	30.75	27
DAPK1	1612	Leukemia,	MESH:D015473	Arsenic|ar	30.74	163
VWF	7450	Leukemia,	MESH:D015473	Arsenic|Ar	30.74	163
CALR	811	Leukemia,	MESH:D015470	Arsenic Tr	30.72	38
HOPX	84525	Leukemia,	MESH:D015473	Antimony	30.72	266
MACF1	23499	Leukemia,	MESH:D015473	Arsenic|Ar	30.72	270
MSMO1	6307	Leukemia,	MESH:D015470	Arsenic Tr	30.72	36
SDC1	6382	Leukemia,	MESH:D015470	Air Polluta	30.72	57
TP73	7161	Leukemia,	MESH:D015473	2-(2-chlor	30.72	267
CDC25C	995	Leukemia,	MESH:D015473	Arsenic|Ar	30.71	267
CBX1	10951	Leukemia,	MESH:D015470	Dexameth	30.7	19
EXOSC5	56915	Leukemia,	MESH:D015470	Arsenic Tr	30.7	24
MAPK7	5598	Leukemia,	MESH:D015470	Arsenic Tr	30.7	26
STK38	11329	Leukemia,	MESH:D015470	Air Polluta	30.7	21
KCTD12	115207	Leukemia,	MESH:D015470	Arsenic Tr	30.69	90
KRT23	25984	Leukemia,	MESH:D015470	Calcitriol|D	30.69	22
NR4A1	3164	Leukemia,	MESH:D015470	Arsenic Tr	30.69	44
EEF2	1938	Leukemia,	MESH:D015470	Arsenic Tr	30.68	107
MMP7	4316	Leukemia,	MESH:D015473	Arsenic Tr	30.68	264
GAPDH	2597	Leukemia,	MESH:D015473	Antimony	30.65	266
IMPDH2	3615	Leukemia,	MESH:D015473	Arsenic Tr	30.64	261
CPEB2	132864	Leukemia,	MESH:D015473	arsenite|Ca	30.63	159

RGS1	5996	Leukemia, MESH:D015473		Arsenic|ar:	30.63	161
CFLAR	8837	Leukemia, MESH:D015473		alvocidib|/	30.62	263
IFIT1	3434	Leukemia, MESH:D015473		alvocidib|a	30.62	160
TNFRSF11	4982	Leukemia, MESH:D015473		Arsenic Tr	30.62	264
XRCC1	7515	Leukemia, MESH:D015473		Arsenic|ar:	30.62	164
TGM2	7052	Leukemia, MESH:D015470		Alitretinoi	30.6	26
FTH1	2495	Leukemia, MESH:D015470		Arsenic Tr	30.59	46
LIMCH1	22998	Leukemia, MESH:D015470		Calcitriol|(	30.59	86
MAOA	4128	Leukemia, MESH:D015470		Arsenic Tr	30.59	37
PCSK5	5125	Leukemia, MESH:D015470		Arsenic Tr	30.59	102
DNA2	1763	Leukemia, MESH:D015473		Arsenic|ar:	30.58	161
ZNF383	163087	Leukemia, MESH:D015470		Arsenic Tr	30.58	86
PIM2	11040	Leukemia, MESH:D01	marker/m	Dexameth	30.56	21
NMB	4828	Leukemia, MESH:D015470		Decitabine	30.56	27
SKI	6497	Leukemia, MESH:D015470		Decitabine	30.56	26
TMED3	23423	Leukemia, MESH:D015470		Azacitidine	30.56	26
IGF1	3479	Leukemia, MESH:D015473		Arsenic|Ar	30.55	272
ISG20	3669	Leukemia, MESH:D015470		Calcitriol|D	30.55	46
PTGDS	5730	Leukemia, MESH:D015470		Calcitriol|D	30.55	26
TBC1D17	79735	Leukemia, MESH:D015470		Decitabine	30.55	22
TMEM41B	440026	Leukemia, MESH:D015470		Bortezomi	30.55	14
UGCG	7357	Leukemia, MESH:D015470		Dexameth	30.55	25
VIT	5212	Leukemia, MESH:D015470		Arsenic Tr	30.55	26
CLDN7	1366	Leukemia, MESH:D015470		Arsenic Tr	30.48	26
GAS7	8522	Leukemia, MESH:D015470		Arsenic Tr	30.48	27
PDE4B	5142	Leukemia, MESH:D01	marker/m	Arsenic Tr	30.47	103
PIK3CA	5290	Leukemia, MESH:D015470		Arsenic Tr	30.47	27
VAV3	10451	Leukemia, MESH:D015473		Arsenic|Ar	30.47	269
ALDH1A3	220	Leukemia, MESH:D015473		Antineopl	30.44	267
AP2B1	163	Leukemia, MESH:D015470		Arsenic Tr	30.42	19
GYPC	2995	Leukemia, MESH:D015470		Decitabine	30.42	24
MECR	51102	Leukemia, MESH:D015470		Decitabine	30.42	27
RRAGC	64121	Leukemia, MESH:D015470		Benzene|D	30.42	30
SETD5	55209	Leukemia, MESH:D015470		Benzene|D	30.42	31
SLFN5	162394	Leukemia, MESH:D015470		Arsenic Tr	30.42	22
TOR1A	1861	Leukemia, MESH:D015470		Air Polluta	30.42	27
GLRX	2745	Leukemia, MESH:D015470		Air Polluta	30.4	22
MYH7	4625	Leukemia, MESH:D015470		Azacitidine	30.4	57
XDH	7498	Leukemia, MESH:D015470		Allopurinc	30.4	29
AHCY	191	Leukemia, MESH:D015470		Arsenic Tr	30.38	41
BUB1B	701	Leukemia, MESH:D015473		Arsenic Tr	30.38	264
FGL2	10875	Leukemia, MESH:D015470		Air Polluta	30.38	23
GZMB	3002	Leukemia, MESH:D015470		Azacitidine	30.38	20
HCK	3055	Leukemia, MESH:D015473		arsenite|Ca	30.38	162
IGFBP6	3489	Leukemia, MESH:D015470		Decitabine	30.38	28
RAMP1	10267	Leukemia, MESH:D015470		Calcitriol|(	30.38	85
USP7	7874	Leukemia, MESH:D015470		ABT-737|(	30.38	86
FCER1G	2207	Leukemia, MESH:D015470		Arsenic Tr	30.37	30
TXNRD1	7296	Leukemia, MESH:D015473		Antimony	30.36	267
PALM2AK	445815	Leukemia, MESH:D015473		Arsenic|Ar	30.33	270
CDKN2C	1031	Leukemia, MESH:D015473		Arsenic Tr	30.32	265
CEBPD	1052	Leukemia, MESH:D007948		2-(2-amin	30.32	4
DIABLO	56616	Leukemia, MESH:D015473		alpha-Toc	30.32	264
FGF2	2247	Leukemia, MESH:D015470		Arsenic Tr	30.32	37
UHRF1	29128	Leukemia, MESH:D015473		Arsenic Tr	30.32	264
TRP53	22059	Leukemia, MESH:D015473		Arsenic|Ar	30.31	269
CARHSP1	23589	Leukemia, MESH:D015473		arsenite|Ca	30.3	159
MFGE8	4240	Leukemia, MESH:D015473		Arsenic|Da	30.3	162

SMPD1	6609	Leukemia, MESH:D015473		Arsenic Tr	30.3	269
LHFPL2	10184	Leukemia, MESH:D015470		Benzene|C	30.28	26
PLPP1	8611	Leukemia, MESH:D015470		Arsenic Tr	30.28	20
SLFN11	91607	Leukemia, MESH:D015470		Benzene|C	30.28	94
ARID5B	84159	Leukemia, MESH:D015470		Arsenic Tr	30.27	25
E2F8	79733	Leukemia, MESH:D015473		Arsenic|Ar	30.27	271
FANCI	55215	Leukemia, MESH:D015470		Calcitriol|D	30.27	19
IMPDH2	3615	Leukemia, MESH:D015470		Arsenic Tr	30.27	30
MERTK	10461	Leukemia, MESH:D015470		Arsenic Tr	30.27	95
CD40	958	Leukemia, MESH:D015470		Arsenic Tr	30.26	37
ABCG2	9429	Leukemia, MESH:D015473		Arsenic Tr	30.25	267
COL6A1	1291	Leukemia, MESH:D015470		Alitretinoi	30.22	22
LSP1	4046	Leukemia, MESH:D015473		Arsenic|Ar	30.22	266
RBP4	5950	Leukemia, MESH:D015470		Decitabine	30.22	26
TP53INP1	94241	Leukemia, MESH:D015473		Arsenic|Ar	30.22	265
ATF6	22926	Leukemia, MESH:D015470		Allopurinc	30.2	31
MAPK9	5601	Leukemia, MESH:D015473		alpha-Toc	30.19	269
HLA-B	3106	Leukemia, MESH:D015470		Allopurinc	30.17	31
MX2	4600	Leukemia, MESH:D015470		Air Polluta	30.17	33
NFKBIE	4794	Leukemia, MESH:D015470		Arsenic Tr	30.17	25
POGLUT1	56983	Leukemia, MESH:D015470		Air Polluta	30.17	17
POLD1	5424	Leukemia, MESH:D015470		Arsenic Tr	30.17	25
RPS6KA4	8986	Leukemia, MESH:D015470		Arsenic Tr	30.17	23
ITGB1	3688	Leukemia, MESH:D015470		Arsenic Tr	30.15	31
KIF20A	10112	Leukemia, MESH:D015473		arsenite|Ca	30.15	158
VEGFC	7424	Leukemia, MESH:D015473		Arsenic|Ar	30.15	268
ALPK2	115701	Leukemia, MESH:D015470		Calcitriol|C	30.14	85
B3GNT2	10678	Leukemia, MESH:D015470		Bortezomi	30.14	18
EPB41L3	23136	Leukemia, MESH:D015473		Arsenic|ars	30.14	160
HIF3A	64344	Leukemia, MESH:D015470		Clioquinol	30.14	18
KCNA5	3741	Leukemia, MESH:D015470		Decitabine	30.14	28
LBH	81606	Leukemia, MESH:D015473		arsenite|Ca	30.14	161
LTBP3	4054	Leukemia, MESH:D015470		Calcitriol|D	30.14	24
ANPEP	290	Leukemia, MESH:D015470		Air Polluta	30.13	42
BMF	90427	Leukemia, MESH:D015470		Arsenic Tr	30.13	28
COL5A2	1290	Leukemia, MESH:D015470		Cytarabine	30.13	96
GLB1	2720	Leukemia, MESH:D015470		Air Polluta	30.13	27
STAT6	6778	Leukemia, MESH:D015470		Calcitriol|C	30.13	23
CTH	1491	Leukemia, MESH:D015473		Arsenic|Ar	30.12	268
HRAS	3265	Leukemia, MESH:D015470		Arsenic Tr	30.1	52
ACTA1	58	Leukemia, MESH:D015473		Arsenic|ars	30.09	32
GSTO1	9446	Leukemia, MESH:D015473		Antimony	30.09	270
ANXA3	306	Leukemia, MESH:D015470		Arsenic Tr	30.07	37
ARPC1B	10095	Leukemia, MESH:D015470		Alitretinoi	30.07	20
GGT1	2678	Leukemia, MESH:D015470		Arsenic Tr	30.07	33
PSRC1	84722	Leukemia, MESH:D015470		Benzene|C	30.07	94
VDAC1	7416	Leukemia, MESH:D015470		Arsenic Tr	30.07	45
RASGRP1	10125	Leukemia, MESH:D01	marker/m	Benzene|D	30.06	38
GART	2618	Leukemia, MESH:D015473		Arsenic|Ar	30.06	267
HDAC4	9759	Leukemia, MESH:D015473		Arsenic|Ar	30.06	266
HSPA2	3306	Leukemia, MESH:D015473		Arsenic|Ar	30.03	168
APOBEC3I	9582	Leukemia, MESH:D015470		Calcitriol|C	30.01	87
CDC42BP/	8476	Leukemia, MESH:D015470		Arsenic Tr	30.01	25
IDS	3423	Leukemia, MESH:D015470		Arsenic Tr	30.01	23
SGO2	151246	Leukemia, MESH:D015470		Calcitriol|D	30.01	21
IGFBP1	3484	Leukemia, MESH:D015473		Arsenic|Ar	29.99	267
MOSMO	730094	Leukemia, MESH:D015470		Calcitriol|D	29.99	15
CDCA5	113130	Leukemia, MESH:D015473		Arsenic|ars	29.98	161

ENOSF1	55556	Leukemia, MESH:D015470	Calcitriol|D	29.98	18
HPGD	3248	Leukemia, MESH:D015473	Arsenic|Ar	29.98	269
PLXNA2	5362	Leukemia, MESH:D015473	Arsenic|ars	29.98	161
REPS1	85021	Leukemia, MESH:D015470	Dexameth	29.98	21
TRIP10	9322	Leukemia, MESH:D015470	Dexameth	29.98	16
SLC22A7	10864	Leukemia, MESH:D015470	Allopurinc	29.97	21
COL7A1	1294	Leukemia, MESH:D015470	Arsenic Tr	29.96	20
CCNB2	9133	Leukemia, MESH:D015473	Antimony	29.95	265
CXCL9	4283	Leukemia, MESH:D015470	Arsenic Tr	29.94	36
SLC16A1	6566	Leukemia, MESH:D015470	Alitretinoi	29.94	41
TSC22D1	8848	Leukemia, MESH:D015470	Arsenic Tr	29.94	45
KAT2B	8850	Leukemia, MESH:D015473	Arsenic Tr	29.9	264
LAMC1	3915	Leukemia, MESH:D015473	Arsenic Tr	29.9	267
TOP2B	7155	Leukemia, MESH:D015473	Arsenic|Ar	29.9	268
ENO3	2027	Leukemia, MESH:D015470	Cytarabine	29.89	93
ADGRE5	976	Leukemia, MESH:D015470	Arsenic Tr	29.88	22
CSTF3	1479	Leukemia, MESH:D015470	Air Polluta	29.88	13
MT1M	4499	Leukemia, MESH:D015470	Arsenic Tr	29.88	25
OSBPL6	114880	Leukemia, MESH:D015470	Arsenic Tr	29.88	29
APOB	338	Leukemia, MESH:D015470	Allopurinc	29.87	63
HSP90B1	7184	Leukemia, MESH:D015470	Arsenic Tr	29.87	37
ORM1	5004	Leukemia, MESH:D015473	alpha-Toc	29.87	158
CXCL2	2920	Leukemia, MESH:D015473	alpha-Toc	29.86	165
ELOVL2	54898	Leukemia, MESH:D015470	Arsenic Tr	29.86	31
LRRFIP1	9208	Leukemia, MESH:D015470	Arsenic Tr	29.86	27
TIA1	7072	Leukemia, MESH:D015470	Decitabine	29.86	33
DUSP2	1844	Leukemia, MESH:D015473	Antimony	29.82	261
ENO1	2023	Leukemia, MESH:D015473	Arsenic|ars	29.82	162
BMP7	655	Leukemia, MESH:D015470	Calcitriol|D	29.81	35
BTG1	694	Leukemia, MESH:D015473	arsenic dis	29.81	266
DCN	1634	Leukemia, MESH:D015473	Arsenic Tr	29.81	266
ITGB4	3691	Leukemia, MESH:D015470	Arsenic Tr	29.81	27
CCDC28B	79140	Leukemia, MESH:D015470	Arsenic Tr	29.8	28
CSRNP2	81566	Leukemia, MESH:D015470	Benzene|C	29.8	40
PHF2	5253	Leukemia, MESH:D015470	Air Polluta	29.8	17
MAPK8	5599	Leukemia, MESH:D015473	alpha-Toc	29.77	270
SPP1	6696	Leukemia, MESH:D015473	Arsenic Tr	29.77	270
AK3	50808	Leukemia, MESH:D015470	Dexameth	29.76	23
BDKRB1	623	Leukemia, MESH:D015470	Calcitriol|C	29.76	23
LSP1	4046	Leukemia, MESH:D015470	Arsenic Tr	29.76	28
MAL	4118	Leukemia, MESH:D015470	Decitabine	29.76	24
SPON1	10418	Leukemia, MESH:D015470	Arsenic Tr	29.76	92
ARID2	196528	Leukemia, MESH:D015470	Benzene|D	29.75	34
NFKB2	4791	Leukemia, MESH:D015473	Antimony	29.75	261
PELO	53918	Leukemia, MESH:D015470	Benzene|D	29.75	39
PRELP	5549	Leukemia, MESH:D015470	Arsenic Tr	29.75	27
EFNA5	1946	Leukemia, MESH:D015470	Calcitriol|D	29.74	24
HACD4	401494	Leukemia, MESH:D015470	Arsenic Tr	29.74	22
MIR21	406991	Leukemia, MESH:D015470	Air Polluta	29.74	54
NMU	10874	Leukemia, MESH:D015470	Calcitriol|C	29.74	81
PARPBP	55010	Leukemia, MESH:D015470	Calcitriol|D	29.74	16
PLEKHA2	59339	Leukemia, MESH:D015470	Calcitriol|D	29.74	26
RPL6	6128	Leukemia, MESH:D015473	Arsenic Tr	29.74	264
SPTAN1	6709	Leukemia, MESH:D015470	Arsenic Tr	29.74	34
VCL	7414	Leukemia, MESH:D015470	Dexameth	29.74	23
FPR1	2357	Leukemia, MESH:D015473	Arsenic|Ar	29.73	264
ZNF189	7743	Leukemia, MESH:D015470	Azacitidin	29.72	20
PIM1	5292	Leukemia, MESH:D015473	arsenic dis	29.7	266

CD86	942	Leukemia, MESH:D015470	Alitretinoi	29.68	54
CHAC1	79094	Leukemia, MESH:D015470	Arsenic Tr	29.68	29
ATG12	9140	Leukemia, MESH:D015470	Arsenic Tr	29.67	28
CBLB	868	Leukemia, MESH:D015470	Benzene|C	29.67	33
PFKFB4	5210	Leukemia, MESH:D015470	Arsenic Tr	29.67	33
PYGM	5837	Leukemia, MESH:D015473	alvocidib|a	29.67	24
TRAF1	7185	Leukemia, MESH:D015473	Arsenic Tr	29.67	263
AREG	374	Leukemia, MESH:D015473	Arsenic|ars	29.65	161
GATA3	2625	Leukemia, MESH:D015473	Arsenic|Ar	29.64	267
CASZ1	54897	Leukemia, MESH:D015470	Calcitriol|D	29.62	25
EIF4EBP2	1979	Leukemia, MESH:D015470	Alitretinoi	29.62	17
ELF1	1997	Leukemia, MESH:D015470	Arsenic Tr	29.62	20
FBXW7	55294	Leukemia, MESH:D015470	Arsenic Tr	29.62	20
FMNL2	114793	Leukemia, MESH:D015470	Dexameth	29.62	19
HOOK1	51361	Leukemia, MESH:D015470	Calcitriol|D	29.62	19
LZTS2	84445	Leukemia, MESH:D015470	Dexameth	29.62	14
OSBPL10	114884	Leukemia, MESH:D015470	Cytarabine	29.62	89
PCDH20	64881	Leukemia, MESH:D015470	Arsenic Tr	29.62	88
PRPH	5630	Leukemia, MESH:D015470	Cytarabine	29.62	92
TFAP4	7023	Leukemia, MESH:D015470	Calcitriol|D	29.62	14
CDK6	1021	Leukemia, MESH:D015473	Arsenic|Ar	29.61	266
MEGF6	1953	Leukemia, MESH:D015473	Arsenic|Ar	29.61	266
CD69	969	Leukemia, MESH:D015473	Arsenic|ars	29.59	165
PYGL	5836	Leukemia, MESH:D015470	alvocidib|A	29.58	27
TNFRSF9	3604	Leukemia, MESH:D015470	Arsenic Tr	29.58	31
ZEB2	9839	Leukemia, MESH:D015470	Air Polluta	29.58	25
BARD1	580	Leukemia, MESH:D015470	Bortezomi	29.57	48
GART	2618	Leukemia, MESH:D015470	Air Polluta	29.57	22
KNL1	57082	Leukemia, MESH:D015470	Calcitriol|D	29.57	22
MAP3K6	9064	Leukemia, MESH:D015470	Decitabine	29.57	26
PLEC	5339	Leukemia, MESH:D015470	Decitabine	29.57	27
XPO1	7514	Leukemia, MESH:D015470	Air Polluta	29.57	24
GSTM3	2947	Leukemia, MESH:D015470	Allopurino	29.54	35
JUNB	3726	Leukemia, MESH:D015470	Arsenic Tr	29.54	35
ENPP2	5168	Leukemia, MESH:D015473	Arsenic|Ar	29.53	265
AK4	205	Leukemia, MESH:D015470	Benzene|D	29.5	40
SPTBN1	6711	Leukemia, MESH:D015470	Arsenic Tr	29.5	46
POLR1B	84172	Leukemia, MESH:D015470	Air Polluta	29.49	21
RBM25	58517	Leukemia, MESH:D015470	Dexameth	29.49	20
TLE1	7088	Leukemia, MESH:D015470	Decitabine	29.49	26
LMNB1	4001	Leukemia, MESH:D015473	arsenite|Ca	29.48	163
DSC2	1824	Leukemia, MESH:D015470	Arsenic Tr	29.47	91
PLXNA2	5362	Leukemia, MESH:D015470	Calcitriol|D	29.47	22
SPON2	10417	Leukemia, MESH:D015470	Air Polluta	29.47	29
ATP5IF1	93974	Leukemia, MESH:D015470	Arsenic Tr	29.46	28
ESR1	2099	Leukemia, MESH:D015473	alpha-Toc	29.46	278
NBPF1	55672	Leukemia, MESH:D015470	Air Polluta	29.46	12
AP3S1	1176	Leukemia, MESH:D015470	Arsenic Tr	29.44	19
FAM214B	80256	Leukemia, MESH:D015470	Calcitriol|D	29.44	16
GOLGA8A	23015	Leukemia, MESH:D015470	Dexameth	29.44	19
H3-4	8290	Leukemia, MESH:D015470	Alitretinoi	29.44	27
SLC5A12	159963	Leukemia, MESH:D015470	Arsenic Tr	29.44	87
SMC4	10051	Leukemia, MESH:D015473	Arsenic Tr	29.44	266
TMEM62	80021	Leukemia, MESH:D015470	Cytarabine	29.44	79
ATF2	1386	Leukemia, MESH:D015473	alpha-Toc	29.43	265
BAD	572	Leukemia, MESH:D015473	alpha-Toc	29.42	265
DHFR	1719	Leukemia, MESH:D015470	Cytarabine	29.42	90
CPT1A	1374	Leukemia, MESH:D015473	alpha-Toc	29.39	267

PLAUR	5329	Leukemia, MESH:D015470		Arsenic Tr	29.39	43
ASPH	444	Leukemia, MESH:D015470		Arsenic Tr	29.38	24
PRDM1	639	Leukemia, MESH:D015470		Decitabine	29.38	22
SORBS1	10580	Leukemia, MESH:D015470		Decitabine	29.38	41
TOP2B	7155	Leukemia, MESH:D015470		Amsacrine	29.38	36
KIF15	56992	Leukemia, MESH:D015473		Arsenic|ars	29.37	163
SLC7A2	6542	Leukemia, MESH:D015473		Arsenic|ars	29.37	164
CD3D	915	Leukemia, MESH:D015470		Benzene|C	29.36	28
FOXC2	2303	Leukemia, MESH:D015470		Decitabine	29.36	24
MAP7	9053	Leukemia, MESH:D015470		Arsenic Tr	29.36	34
MMP16	4325	Leukemia, MESH:D015470		Arsenic Tr	29.36	27
MPZL2	10205	Leukemia, MESH:D015470		Decitabine	29.36	20
PITPNC1	26207	Leukemia, MESH:D015470		Arsenic Tr	29.36	25
SLC43A1	8501	Leukemia, MESH:D015470		Arsenic Tr	29.36	22
SLCO4C1	353189	Leukemia, MESH:D015470		Air Polluta	29.36	21
PDGFA	5154	Leukemia, MESH:D015470		Arsenic Tr	29.3	48
BBC3	27113	Leukemia, MESH:D015473		Arsenic|Ar	29.29	269
CRISPLD2	83716	Leukemia, MESH:D015473		Arsenic|Ca	29.29	162
MYBL2	4605	Leukemia, MESH:D015473		Calcitriol|D	29.29	156
BGN	633	Leukemia, MESH:D015470		Dasatinib|I	29.28	25
BSG	682	Leukemia, MESH:D015470		Arsenic Tr	29.28	40
CD40LG	959	Leukemia, MESH:D015470		Arsenic Tr	29.28	33
RFC2	5982	Leukemia, MESH:D015473		Antimony	29.28	265
SLCO2A1	6578	Leukemia, MESH:D015470		Decitabine	29.28	24
TRIM2	23321	Leukemia, MESH:D015470		Air Polluta	29.28	36
ABCA7	10347	Leukemia, MESH:D015470		Docetaxel|	29.27	15
ABI2	10152	Leukemia, MESH:D015470		Arsenic Tr	29.27	17
AXL	558	Leukemia, MESH:D015470		Cyclophos	29.27	95
FOXF2	2295	Leukemia, MESH:D015470		Calcitriol|D	29.27	27
GDF11	10220	Leukemia, MESH:D015470		Decitabine	29.27	22
RAP2A	5911	Leukemia, MESH:D015470		Androgen	29.27	19
TAOK2	9344	Leukemia, MESH:D015470		Air Polluta	29.27	17
SCD	6319	Leukemia, MESH:D015473		Antimony	29.25	162
BDNF	627	Leukemia, MESH:D015473		alpha-Toc	29.24	272
PFAS	5198	Leukemia, MESH:D015470		Arsenic Tr	29.24	33
SLC1A2	6506	Leukemia, MESH:D015470		Arsenic Tr	29.24	32
TIPIN	54962	Leukemia, MESH:D015470		Dasatinib|I	29.24	22
COL6A3	1293	Leukemia, MESH:D015473		Arsenic|Ar	29.22	269
PTPRC	5788	Leukemia, MESH:D015473		Arsenic Tr	29.22	267
TLR4	7099	Leukemia, MESH:D015473		Arsenic|Ar	29.21	271
BCL3	602	Leukemia, MESH:D015470		Air Polluta	29.2	27
ANXA6	309	Leukemia, MESH:D01	marker/m	Cytarabine	29.19	91
LEP	3952	Leukemia, MESH:D01	marker/m	Arsenic|Ar	29.19	267
EIF4G1	1981	Leukemia, MESH:D015470		Arsenic Tr	29.19	31
IFIH1	64135	Leukemia, MESH:D015470		Arsenic Tr	29.19	28
NCAPG	64151	Leukemia, MESH:D015470		Calcitriol|D	29.19	20
FOSB	2354	Leukemia, MESH:D015470		Air Polluta	29.18	35
ALPK2	115701	Leukemia, MESH:D015473		Calcitriol|C	29.17	158
GNAL	2774	Leukemia, MESH:D015473		Arsenic|Ar	29.17	265
HOXA5	3202	Leukemia, MESH:D015473		Arsenic Tr	29.17	266
INS1	16333	Leukemia, MESH:D015470		15-deoxy-	29.17	29
PPP1R15A	23645	Leukemia, MESH:D015473		Arsenic Tr	29.17	268
LHB	3972	Leukemia, MESH:D015470		2-(2-chlor	29.14	33
CYP3A5	1577	Leukemia, MESH:D015470		2-(2-chlor	29.13	33
TNNT2	7139	Leukemia, MESH:D015470		Arsenic Tr	29.13	105
RGS10	6001	Leukemia, MESH:D015470		Decitabine	29.12	23
ZFAND5	7763	Leukemia, MESH:D015470		Arsenic Tr	29.12	37
AAMDC	28971	Leukemia, MESH:D015470		Dexameth	29.1	19

AMFR	267	Leukemia, MESH:D015470	Arsenic Tr	29.1	22
IFT74	80173	Leukemia, MESH:D015470	Decitabine	29.1	22
TOR1AIP1	26092	Leukemia, MESH:D015470	Bortezomi	29.1	17
DDB2	1643	Leukemia, MESH:D015470	Arsenic Tr	29.09	27
EIF1	10209	Leukemia, MESH:D015470	Benzene|B	29.09	35
MT1G	4495	Leukemia, MESH:D015470	Arsenic Tr	29.09	42
NASP	4678	Leukemia, MESH:D015470	Arsenic Tr	29.09	29
SHMT1	6470	Leukemia, MESH:D015470	Arsenic Tr	29.09	23
EIF2A	83939	Leukemia, MESH:D015473	2-(2-chlor	29.08	271
APOBEC3I	9582	Leukemia, MESH:D015473	arsenite|Ca	29.06	160
GGT1	2678	Leukemia, MESH:D015473	Antimony	29.06	270
HOXB9	3219	Leukemia, MESH:D015473	Arsenic|Ar	29.06	264
PLEKHA5	54477	Leukemia, MESH:D015473	Arsenic|ars	29.06	160
SOD3	6649	Leukemia, MESH:D015473	Arsenic Tr	29.06	268
XPOT	11260	Leukemia, MESH:D015473	Arsenic Tr	29.06	267
ITGB2	3689	Leukemia, MESH:D015470	Alitretinoi	29.05	39
PHLDA1	22822	Leukemia, MESH:D015470	Air Polluta	29.05	31
PSAT1	29968	Leukemia, MESH:D015470	Arsenic Tr	29.05	26
CAPG	822	Leukemia, MESH:D015473	Arsenic|Ar	29.01	269
HDAC1	3065	Leukemia, MESH:D015473	arsenic dis	29.01	264
PGK1	5230	Leukemia, MESH:D015473	alpha-Toc	29.01	268
TGM1	7051	Leukemia, MESH:D015473	Antimony	29.01	165
WARS1	7453	Leukemia, MESH:D015473	Arsenic Tr	29.01	269
ALDH1L2	160428	Leukemia, MESH:D015470	Dasatinib|I	29	19
CDK5R1	8851	Leukemia, MESH:D015470	Arsenic Tr	29	90
COL13A1	1305	Leukemia, MESH:D015470	Calcitriol|C	29	89
DHRS2	10202	Leukemia, MESH:D015470	Arsenic Tr	29	32
EFNB1	1947	Leukemia, MESH:D015470	Dexameth	29	19
EML4	27436	Leukemia, MESH:D015470	Arsenic Tr	29	36
EPHA4	2043	Leukemia, MESH:D015470	Air Polluta	29	19
GPC4	2239	Leukemia, MESH:D015470	Arsenic Tr	29	29
GPSM2	29899	Leukemia, MESH:D015470	Calcitriol|D	29	27
NOLC1	9221	Leukemia, MESH:D015470	Air Polluta	29	26
PLSCR1	5359	Leukemia, MESH:D015470	Air Polluta	29	26
RAD18	56852	Leukemia, MESH:D015470	Calcitriol|D	29	18
SEMA3A	10371	Leukemia, MESH:D015470	Arsenic Tr	29	27
VTN	7448	Leukemia, MESH:D015470	Decitabine	29	25
PDIA4	9601	Leukemia, MESH:D015470	Carboplati	28.98	22
SLC27A2	11001	Leukemia, MESH:D015470	Arsenic Tr	28.98	25
ABCB4	5244	Leukemia, MESH:D015473	Arsenic Tr	28.97	264
ASS1	445	Leukemia, MESH:D015473	Arsenic Tr	28.96	266
WNT16	51384	Leukemia, MESH:D015470	Arsenic Tr	28.95	24
CDC45	8318	Leukemia, MESH:D015473	Arsenic Tr	28.94	264
DOK4	55715	Leukemia, MESH:D015470	Calcitriol|C	28.94	91
GTF2A1	2957	Leukemia, MESH:D015470	Arsenic Tr	28.94	17
HIPK1	204851	Leukemia, MESH:D015470	Arsenic Tr	28.94	22
SETD1B	23067	Leukemia, MESH:D015470	Bortezomi	28.94	16
VSIR	64115	Leukemia, MESH:D015470	Decitabine	28.94	23
FGF1	2246	Leukemia, MESH:D015470	Alitretinoi	28.93	28
JUND	3727	Leukemia, MESH:D015470	Air Polluta	28.93	28
CDH13	1012	Leukemia, MESH:D015470	Androgen	28.91	50
ENC1	8507	Leukemia, MESH:D015473	Arsenic Tr	28.91	265
ICAM1	3383	Leukemia, MESH:D015473	alpha-Toc	28.91	273
IRAK2	3656	Leukemia, MESH:D015470	Air Polluta	28.91	24
S100A11	6282	Leukemia, MESH:D015470	Air Polluta	28.91	32
STIP1	10963	Leukemia, MESH:D015470	Air Polluta	28.91	36
VWF	7450	Leukemia, MESH:D015470	Arsenic Tr	28.91	50
ACTB	60	Leukemia, MESH:D015473	Arsenic|Ar	28.89	268

AKR1C3	8644	Leukemia, MESH:D015473	Antimony	28.89	270
ANGPTL2	23452	Leukemia, MESH:D015470	Cytarabine	28.88	82
CRABP1	1381	Leukemia, MESH:D015470	Alitretinoi	28.88	90
FAM107A	11170	Leukemia, MESH:D015470	Decitabine	28.88	24
GDF10	2662	Leukemia, MESH:D015470	Decitabine	28.88	22
NUDT1	4521	Leukemia, MESH:D015470	Benzene|C	28.88	29
PTPN13	5783	Leukemia, MESH:D015470	Doxorubic	28.88	20
SERINC2	347735	Leukemia, MESH:D015470	Calcitriol|D	28.88	13
EIF2S1	1965	Leukemia, MESH:D015470	Arsenic Tr	28.87	38
BCR	613	Leukemia, MESH:D015473	arsenic dis	28.86	262
PARPBP	55010	Leukemia, MESH:D015473	arsenite|Ca	28.85	158
PTPN21	11099	Leukemia, MESH:D015473	Arsenic Tr	28.85	158
FHL2	2274	Leukemia, MESH:D01 marker/me	Arsenic Tr	28.83	41
MMP7	4316	Leukemia, MESH:D015470	Arsenic Tr	28.83	28
SLC30A1	7779	Leukemia, MESH:D015470	Arsenic Tr	28.83	30
SULT1A1	6817	Leukemia, MESH:D015470	Arsenic Tr	28.83	45
CKS1B	1163	Leukemia, MESH:D015470	Calcitriol|D	28.82	27
MAP4K4	9448	Leukemia, MESH:D015470	Bortezomi	28.82	25
NIBAN1	116496	Leukemia, MESH:D015470	Arsenic Tr	28.82	33
PROCR	10544	Leukemia, MESH:D015470	Cyclophos	28.82	36
SMC4	10051	Leukemia, MESH:D015470	Arsenic Tr	28.82	41
GRB10	2887	Leukemia, MESH:D015473	Arsenic|Ar	28.8	267
LTF	4057	Leukemia, MESH:D015473	Arsenic|ars	28.8	159
MCM10	55388	Leukemia, MESH:D015473	arsenite|Ca	28.8	157
POSTN	10631	Leukemia, MESH:D015473	Arsenic Tr	28.8	264
TIMP1	7076	Leukemia, MESH:D015473	Arsenic Tr	28.8	273
TRIP13	9319	Leukemia, MESH:D015473	Arsenic Tr	28.8	264
BCL2L12	83596	Leukemia, MESH:D015473	Arsenic|Ar	28.79	161
FYB1	2533	Leukemia, MESH:D015473	Arsenic|Ar	28.79	267
NEMP1	23306	Leukemia, MESH:D015473	arsenite|Ca	28.79	161
NOS2A	404036	Leukemia, MESH:D015473	Arsenic Tr	28.79	263
RPS6	6194	Leukemia, MESH:D015470	Arsenic Tr	28.77	31
SPATA7	55812	Leukemia, MESH:D015470	Cytarabine	28.77	90
UBE2W	55284	Leukemia, MESH:D015470	Dexameth	28.77	19
ALDH3A1	218	Leukemia, MESH:D015470	Arsenic Tr	28.76	42
ALOX12	239	Leukemia, MESH:D015470	Benzene|B	28.76	33
CD4	920	Leukemia, MESH:D015470	Benzene|C	28.76	38
CKS2	1164	Leukemia, MESH:D015470	Calcitriol|C	28.76	83
CTBP2	1488	Leukemia, MESH:D015470	Air Polluta	28.76	18
FERMT1	55612	Leukemia, MESH:D015470	Arsenic Tr	28.76	28
RAB8B	51762	Leukemia, MESH:D015470	Benzene|B	28.76	36
RASD1	51655	Leukemia, MESH:D015470	Calcitriol|D	28.76	21
BTF3	689	Leukemia, MESH:D015473	Antimony	28.75	267
CEACAM1	634	Leukemia, MESH:D015473	Calcitriol|D	28.73	160
KIF15	56992	Leukemia, MESH:D015470	Calcitriol|C	28.73	89
THBS2	7058	Leukemia, MESH:D015470	Calcitriol|D	28.73	18
SENP3	26168	Leukemia, MESH:D015470	Azacitidine	28.71	16
ZNF22	7570	Leukemia, MESH:D015470	Decitabine	28.71	21
CDH2	1000	Leukemia, MESH:D015473	2-(2-chlor	28.7	268
BRAF	673	Leukemia, MESH:D015470	ABT-737|A	28.69	23
NEDD9	4739	Leukemia, MESH:D015470	Alitretinoi	28.69	41
CYBB	1536	Leukemia, MESH:D015473	Arsenic|Ar	28.66	273
ITPR3	3710	Leukemia, MESH:D015473	Arsenic Tr	28.66	267
PDK1	5163	Leukemia, MESH:D015473	Arsenic Tr	28.66	264
TMEM97	27346	Leukemia, MESH:D015473	Arsenic Tr	28.66	261
FGF8	2253	Leukemia, MESH:D015470	Androgen	28.65	17
H2BC5	3017	Leukemia, MESH:D015473	Arsenic Tr	28.65	263
KIF1A	547	Leukemia, MESH:D015470	Daunorub	28.65	49

NKX3-1	4824	Leukemia, MESH:D015470		Alitretinoi	28.65	25
PARP9	83666	Leukemia, MESH:D015470		Air Polluta	28.65	20
TJP3	27134	Leukemia, MESH:D015470		Calcitriol|D	28.65	21
TNNT1	7138	Leukemia, MESH:D015473		arsenite|ca	28.65	161
ASF1B	55723	Leukemia, MESH:D015470		Calcitriol|D	28.64	22
CCL3	6348	Leukemia, MESH:D015473		Arsenic|ars	28.64	266
LTBP1	4052	Leukemia, MESH:D015470		Benzene|D	28.64	39
SAMD4A	23034	Leukemia, MESH:D015470		Dexameth	28.64	20
SH3PXD2A	9644	Leukemia, MESH:D015470		Calcitriol|D	28.64	18
TPM1	7168	Leukemia, MESH:D015470		Bortezomi	28.64	31
CD80	941	Leukemia, MESH:D015470		Alitretinoi	28.62	31
CLDN4	1364	Leukemia, MESH:D015470		Arsenic Tr	28.62	27
EHBP1L1	254102	Leukemia, MESH:D015470		Calcitriol|D	28.62	21
FRK	2444	Leukemia, MESH:D015470		Arsenic Tr	28.62	22
PTX3	5806	Leukemia, MESH:D015470		Arsenic Tr	28.62	39
TOR1B	27348	Leukemia, MESH:D015470		Air Polluta	28.62	26
CLPP	8192	Leukemia, MESH:D015470		Dexameth	28.61	16
GLOD4	51031	Leukemia, MESH:D015470		Cyclophos	28.61	16
HNRNPLL	92906	Leukemia, MESH:D015470		Benzene|B	28.61	28
IK	3550	Leukemia, MESH:D015470		Arsenic Tr	28.61	23
KSR1	8844	Leukemia, MESH:D015470		Benzene|C	28.61	33
PLK1	5347	Leukemia, MESH:D015473		Arsenic Tr	28.61	264
S100A4	6275	Leukemia, MESH:D015473		Arsenic Tr	28.61	270
SALL2	6297	Leukemia, MESH:D015470		Dexameth	28.61	25
AQP9	366	Leukemia, MESH:D01	marker/m	Arsenic|Ar	28.59	267
REL	5966	Leukemia, MESH:D015473		Arsenic|Ar	28.59	265
MDM2	4193	Leukemia, MESH:D015473		alvocidib|A	28.58	170
IRF1	3659	Leukemia, MESH:D015473		Arsenic|Ar	28.56	270
ACAT1	38	Leukemia, MESH:D015470		Arsenic Tr	28.55	27
FGF12	2257	Leukemia, MESH:D015473		Arsenic|Ar	28.55	265
FKBP1A	2280	Leukemia, MESH:D015470		Benzene|B	28.55	33
IL13	3596	Leukemia, MESH:D015473		Arsenic|Ar	28.55	265
MMP16	4325	Leukemia, MESH:D015473		Arsenic|Ar	28.55	263
PAICS	10606	Leukemia, MESH:D015470		Air Polluta	28.55	25
PTPRC	5788	Leukemia, MESH:D015470		Arsenic Tr	28.55	36
COL5A3	50509	Leukemia, MESH:D015470		Dexameth	28.53	22
FAM20C	56975	Leukemia, MESH:D015470		Arsenic Tr	28.53	30
HNRNPL	3191	Leukemia, MESH:D015470		Arsenic Tr	28.53	25
LGALS8	3964	Leukemia, MESH:D015470		Arsenic Tr	28.53	36
MEOX1	4222	Leukemia, MESH:D015470		Air Polluta	28.53	22
NR5A2	2494	Leukemia, MESH:D015473		Arsenic|ars	28.52	159
PPIA	5478	Leukemia, MESH:D015473		alpha-Toc	28.52	264
RACGAP1	29127	Leukemia, MESH:D015473		Arsenic Tr	28.52	264
NAF1	92345	Leukemia, MESH:D015470		Bortezomi	28.48	12
UBXN6	80700	Leukemia, MESH:D015470		Decitabine	28.48	21
WDR59	79726	Leukemia, MESH:D015470		Dexameth	28.48	12
CXCL11	6373	Leukemia, MESH:D015470		Calcitriol|C	28.47	17
FCGR2B	2213	Leukemia, MESH:D015470		Dexameth	28.47	23
GLO1	2739	Leukemia, MESH:D015470		Arsenic Tr	28.47	25
KCNMA1	3778	Leukemia, MESH:D015470		Arsenic Tr	28.47	20
AFTPH	54812	Leukemia, MESH:D015470		Arsenic Tr	28.46	22
AHI1	54806	Leukemia, MESH:D015470		Cytarabine	28.46	86
ANKFY1	51479	Leukemia, MESH:D015470		Benzene|D	28.46	30
APBB1	322	Leukemia, MESH:D015470		Arsenic Tr	28.46	24
BCAP31	10134	Leukemia, MESH:D015470		Dexameth	28.46	17
DENND1A	57706	Leukemia, MESH:D015470		Cytarabine	28.46	82
DONSON	29980	Leukemia, MESH:D015470		Calcitriol|D	28.46	19
EP300	2033	Leukemia, MESH:D015473		Arsenic Tr	28.46	265

FAM117A	81558	Leukemia, MESH:D015470		Air Polluta	28.46	26
LIG1	3978	Leukemia, MESH:D015473		Arsenic|ar:	28.46	161
PATL1	219988	Leukemia, MESH:D015470		Dexameth	28.46	20
TFF1	7031	Leukemia, MESH:D015470		Arsenic Tr	28.46	33
ZNF395	55893	Leukemia, MESH:D015470		Dexameth	28.46	14
ZNF652	22834	Leukemia, MESH:D015470		Dexameth	28.46	20
CCL1	6346	Leukemia, MESH:D015473		Arsenic|Ar	28.45	265
SNHG32	50854	Leukemia, MESH:D015473		Arsenic|Ar	28.45	161
FTH1	2495	Leukemia, MESH:D015473		Antimony	28.44	266
ACTN2	88	Leukemia, MESH:D015470		Air Polluta	28.42	18
ANGPTL4	51129	Leukemia, MESH:D015470		Bezafibrat	28.42	30
ARHGAP2	83478	Leukemia, MESH:D015470		Dexameth	28.42	15
AZGP1	563	Leukemia, MESH:D015470		Arsenic Tr	28.42	30
CD93	22918	Leukemia, MESH:D015470		Air Polluta	28.42	20
COL15A1	1306	Leukemia, MESH:D015470		Dexameth	28.42	29
EMP2	2013	Leukemia, MESH:D015470		Arsenic Tr	28.42	30
FGD4	121512	Leukemia, MESH:D015470		Air Polluta	28.42	16
IL17RB	55540	Leukemia, MESH:D015470		Calcitriol|(	28.42	19
MEF2D	4209	Leukemia, MESH:D015470		Azacitidin	28.42	33
SATB1	6304	Leukemia, MESH:D015470		Arsenic Tr	28.42	46
SMIM14	201895	Leukemia, MESH:D015470		Calcitriol|D	28.42	22
SQOR	58472	Leukemia, MESH:D015470		Air Polluta	28.42	24
ZFHX4	79776	Leukemia, MESH:D015470		Calcitriol|C	28.42	86
BMP4	652	Leukemia, MESH:D015473		Arsenic|ar:	28.41	164
MCM6	4175	Leukemia, MESH:D015473		Arsenic Tr	28.41	264
FOSL1	8061	Leukemia, MESH:D015473		alpha-Toc	28.4	266
ARHGDIB	397	Leukemia, MESH:D015473		Arsenic|Ar	28.39	268
PTCH1	5727	Leukemia, MESH:D015473		Arsenic|Ar	28.39	266
ALOX5AP	241	Leukemia, MESH:D015470		Arsenic Tr	28.38	37
FAH	2184	Leukemia, MESH:D015470		Benzoates	28.38	16
TRADD	8717	Leukemia, MESH:D015470		Dexameth	28.38	15
JAK2	3717	Leukemia, MESH:D01	marker/m	15-deoxy-	28.37	67
IL1B	3553	Leukemia, MESH:D015473		alpha-Toc	28.37	274
CASP6	839	Leukemia, MESH:D015473		Arsenic|Ar	28.36	268
NAMPT	10135	Leukemia, MESH:D015473		Arsenic Tr	28.36	267
SMAD3	4088	Leukemia, MESH:D015473		Arsenic|Ar	28.36	266
HMCN1	83872	Leukemia, MESH:D015473		Arsenic|Ca	28.35	156
OASL	8638	Leukemia, MESH:D015473		Arsenic|Ca	28.35	163
CEBPB	1051	Leukemia, MESH:D015473		Arsenic|Ar	28.34	272
ANXA2P3	305	Leukemia, MESH:D015470		Arsenic Tr	28.31	16
FBP2	8789	Leukemia, MESH:D015470		Benzene|D	28.31	33
KDM1A	23028	Leukemia, MESH:D015470		Arsenic Tr	28.31	22
KLF13	51621	Leukemia, MESH:D015470		Arsenic Tr	28.31	39
PIP5K1B	8395	Leukemia, MESH:D015470		Doxorubic	28.31	24
UBE2L6	9246	Leukemia, MESH:D015470		Arsenic Tr	28.31	29
CD27	939	Leukemia, MESH:D015470		Arsenic Tr	28.3	20
GIT2	9815	Leukemia, MESH:D015470		Air Polluta	28.3	24
MGAT4A	11320	Leukemia, MESH:D015470		Doxorubic	28.3	20
PHLDB2	90102	Leukemia, MESH:D015470		Arsenic Tr	28.3	21
PLEKHF2	79666	Leukemia, MESH:D015470		Dexameth	28.3	14
RAB2A	5862	Leukemia, MESH:D015470		Arsenic Tr	28.3	19
RAP1GAP2	23108	Leukemia, MESH:D015470		Calcitriol|D	28.3	16
SMARCB1	6598	Leukemia, MESH:D015470		Arsenic Tr	28.3	24
CAPG	822	Leukemia, MESH:D01	marker/m	Arsenic Tr	28.29	25
AGO2	27161	Leukemia, MESH:D015470		Arsenic Tr	28.29	24
CLIC4	25932	Leukemia, MESH:D015470		Air Polluta	28.29	18
ECE1	1889	Leukemia, MESH:D015470		Benzene|D	28.29	35
LGMN	5641	Leukemia, MESH:D015470		Arsenic Tr	28.29	23

ATG5	9474	Leukemia, MESH:D015473		Arsenic Tr	28.27	265
PRKN	5071	Leukemia, MESH:D015470		Arsenic Tr	28.27	26
CDCA3	83461	Leukemia, MESH:D015473		Arsenic|ars	28.26	161
LY96	23643	Leukemia, MESH:D015473		Arsenic|Ar	28.26	164
MXD1	4084	Leukemia, MESH:D015473		Arsenic|Ar	28.26	267
PAK2	5062	Leukemia, MESH:D015473		Arsenic|Ar	28.26	268
C1ORF21	81563	Leukemia, MESH:D015470		Calcitriol|D	28.25	15
DFFA	1676	Leukemia, MESH:D007948		2-(2-amin	28.23	3
PIK3R1	5295	Leukemia, MESH:D015470		Alitretinoi	28.23	55
ABCC6	368	Leukemia, MESH:D015470		Alitretinoi	28.21	21
BAMBI	25805	Leukemia, MESH:D015470		Arsenic Tr	28.21	34
RETSAT	54884	Leukemia, MESH:D015470		Dexameth	28.21	27
TCF7L2	6934	Leukemia, MESH:D015470		Arsenic Tr	28.21	24
APP	351	Leukemia, MESH:D015470		Alitretinoi	28.2	63
CX3CL1	6376	Leukemia, MESH:D015470		Air Polluta	28.2	25
ITGA4	3676	Leukemia, MESH:D015473		Arsenic Tr	28.2	267
MYCN	4613	Leukemia, MESH:D015473		Antimony	28.2	264
CD34	947	Leukemia, MESH:D015470		Arsenic Tr	28.19	47
CSE1L	1434	Leukemia, MESH:D015470		Calcitriol|C	28.19	24
TPMT	7172	Leukemia, MESH:D015470		Arsenic Tr	28.19	27
INSR	3643	Leukemia, MESH:D015470		Alitretinoi	28.18	23
TNFRSF12	51330	Leukemia, MESH:D015473		Arsenic|Ar	28.17	266
DENND5A	23258	Leukemia, MESH:D015470		Air Polluta	28.15	32
FAM111B	374393	Leukemia, MESH:D015470		Calcitriol|D	28.15	21
FGF4	2249	Leukemia, MESH:D015470		Decitabine	28.15	19
TRPC1	7220	Leukemia, MESH:D015470		Arsenic Tr	28.15	21
DNMT3A	1788	Leukemia, MESH:D01	marker/m	Arsenic|ars	28.13	268
ACTA1	58	Leukemia, MESH:D015470		Calcitriol|C	28.13	105
ARNTL	406	Leukemia, MESH:D015470		Air Polluta	28.13	26
GSTO1	9446	Leukemia, MESH:D015470		Arsenic Tr	28.13	31
HADH	3033	Leukemia, MESH:D015470		Air Polluta	28.13	26
JAK1	3716	Leukemia, MESH:D015470		Arsenic Tr	28.13	25
IGF2	3481	Leukemia, MESH:D015470		Arsenic Tr	28.12	32
NOTCH2	4853	Leukemia, MESH:D015470		Arsenic Tr	28.12	24
FASN	2194	Leukemia, MESH:D015473		Arsenic|Ar	28.11	269
CCL8	6355	Leukemia, MESH:D015470		Bortezomi	28.09	20
MIR155	406947	Leukemia, MESH:D015470		Air Polluta	28.09	25
PRF1	5551	Leukemia, MESH:D015470		Air Polluta	28.09	23
STAR	6770	Leukemia, MESH:D015470		Arsenic Tr	28.09	32
ABCA5	23461	Leukemia, MESH:D015470		Docetaxel|	28.08	19
RHOU	58480	Leukemia, MESH:D015470		Decitabine	28.08	26
ST3GAL6	10402	Leukemia, MESH:D015470		Azacitidine	28.08	91
UBE2H	7328	Leukemia, MESH:D015470		Air Polluta	28.08	20
XIAP	331	Leukemia, MESH:D007948		2-(2-amin	28.08	4
COL5A1	1289	Leukemia, MESH:D015470		Cytarabine	28.07	88
PLXNC1	10154	Leukemia, MESH:D015473		Arsenic|Ar	28.07	266
ADGRG1	9289	Leukemia, MESH:D015470		Air Polluta	28.04	26
DDX17	10521	Leukemia, MESH:D015470		Dexameth	28.04	16
JUP	3728	Leukemia, MESH:D015470		Calcitriol|D	28.04	26
KIFC1	3833	Leukemia, MESH:D015470		Benzene|B	28.04	35
MCM10	55388	Leukemia, MESH:D015470		Calcitriol|D	28.04	29
PRKCA	5578	Leukemia, MESH:D015473		Arsenic|Ar	28.04	266
SDC2	6383	Leukemia, MESH:D015470		Arsenic Tr	28.04	35
SMOX	54498	Leukemia, MESH:D015470		Air Polluta	28.04	19
TRIP13	9319	Leukemia, MESH:D015470		Arsenic Tr	28.04	23
H2-K1	14972	Leukemia, MESH:D015470		Azacitidine	28.03	14
SSH3	54961	Leukemia, MESH:D015470		Decitabine	28.03	22
ACVR1C	130399	Leukemia, MESH:D015470		Benzene|C	28.01	31

KLK6	5653	Leukemia, MESH:D015470	Calcitriol|C	28.01	88
NCOA6	23054	Leukemia, MESH:D015470	Dexameth	28.01	16
NRAP	4892	Leukemia, MESH:D015470	Cyclophos	28.01	52
PLEKHH1	57475	Leukemia, MESH:D015470	Dexameth	28.01	11
PRKAR1B	5575	Leukemia, MESH:D015470	Arsenic Tr	28.01	24
SIRT5	23408	Leukemia, MESH:D015470	Arsenic Tr	28.01	18
SNN	8303	Leukemia, MESH:D015470	Alitretinoi	28.01	14
UNC93B1	81622	Leukemia, MESH:D015470	Air Polluta	28.01	12
ZFPM2	23414	Leukemia, MESH:D015470	Arsenic Tr	28.01	87
CENPE	1062	Leukemia, MESH:D015473	Arsenic|ars	28	161
ESYT1	23344	Leukemia, MESH:D015470	Air Polluta	28	23
GAP43	2596	Leukemia, MESH:D015470	Arsenic Tr	28	91
GBP2	2634	Leukemia, MESH:D015473	Arsenic|ars	28	160
GPD1L	23171	Leukemia, MESH:D015470	Dexameth	28	17
LRP10	26020	Leukemia, MESH:D015470	Calcitriol|D	28	11
USP53	54532	Leukemia, MESH:D015470	Bortezomi	28	12
MIR29A	407021	Leukemia, MESH:D015473	Arsenic|Ar	27.99	163
AMD1	262	Leukemia, MESH:D015470	Arsenic Tr	27.98	23
DTYMK	1841	Leukemia, MESH:D015470	Calcitriol|D	27.98	20
FXYD5	53827	Leukemia, MESH:D015470	Decitabine	27.98	24
HDAC3	8841	Leukemia, MESH:D015470	15-deoxy-	27.98	18
LGR4	55366	Leukemia, MESH:D015470	Arsenic Tr	27.98	32
NAB2	4665	Leukemia, MESH:D015470	Arsenic Tr	27.98	37
OAS1	4938	Leukemia, MESH:D015470	Air Polluta	27.98	20
PDGFRL	5157	Leukemia, MESH:D015470	Calcitriol|D	27.98	24
POLA2	23649	Leukemia, MESH:D015470	Calcitriol|D	27.98	20
PPAT	5471	Leukemia, MESH:D015473	Arsenic|Ar	27.98	266
ARF1	375	Leukemia, MESH:D015473	Arsenic|Ar	27.97	265
DEPDC1	55635	Leukemia, MESH:D015473	Arsenic Tr	27.97	262
CDT1	81620	Leukemia, MESH:D015470	Bortezomi	27.96	17
ENPP1	5167	Leukemia, MESH:D015470	Allopurino	27.96	19
PTGS2	5743	Leukemia, MESH:D015473	alpha-Toc	27.96	275
SLC39A8	64116	Leukemia, MESH:D015470	Benzoates	27.96	21
SPAG5	10615	Leukemia, MESH:D015470	Azacitidine	27.96	32
ALOX5	240	Leukemia, MESH:D015470	Air Polluta	27.95	38
IFI27	3429	Leukemia, MESH:D015470	Calcitriol|D	27.95	28
MCL1	4170	Leukemia, MESH:D007948	2-(2-amin	27.95	4
USP18	11274	Leukemia, MESH:D015470	Benzene|D	27.95	35
FBLN1	2192	Leukemia, MESH:D015473	Arsenic Tr	27.94	264
PCDHA11	56138	Leukemia, MESH:D015470	Azacitidine	27.94	10
PLTP	5360	Leukemia, MESH:D015473	alpha-Toc	27.94	268
GATA4	2626	Leukemia, MESH:D015470	Arsenic Tr	27.93	25
CYP17A1	1586	Leukemia, MESH:D015470	Androgen	27.91	43
LOX	4015	Leukemia, MESH:D015470	Calcitriol|C	27.9	95
CD53	963	Leukemia, MESH:D015473	Arsenic Tr	27.88	267
GPM6B	2824	Leukemia, MESH:D015473	Arsenic Tr	27.88	259
IVL	3713	Leukemia, MESH:D015473	Antimony	27.88	267
MVP	9961	Leukemia, MESH:D015473	Antineopla	27.88	266
ARRDC2	27106	Leukemia, MESH:D015470	Benzene|D	27.87	33
BDH2	56898	Leukemia, MESH:D015470	Arsenic Tr	27.87	22
CEMIP2	23670	Leukemia, MESH:D015470	Dexameth	27.87	17
DST	667	Leukemia, MESH:D015470	Arsenic Tr	27.87	18
NQO2	4835	Leukemia, MESH:D015470	Arsenic Tr	27.87	25
OGN	4969	Leukemia, MESH:D015470	Arsenic Tr	27.87	26
PRIM2	5558	Leukemia, MESH:D015470	Benzene|D	27.87	28
RALA	5898	Leukemia, MESH:D015470	Androgen	27.87	22
TACSTD2	4070	Leukemia, MESH:D015470	Alitretinoi	27.87	19
TCF7	6932	Leukemia, MESH:D015470	Arsenic Tr	27.87	27

ZBTB10	65986	Leukemia, MESH:D015470		Air Polluta	27.87	21
MLLT10	8028	Leukemia, MESH:D01	marker/m	Arsenic Tr	27.86	24
AGPS	8540	Leukemia, MESH:D015470		Arsenic Tr	27.86	18
CNNM2	54805	Leukemia, MESH:D015470		Arsenic Tr	27.86	22
DNA2	1763	Leukemia, MESH:D015470		Calcitriol|D	27.86	16
H3C4	8351	Leukemia, MESH:D015470		Calcitriol|D	27.86	12
IPO9	55705	Leukemia, MESH:D015470		Calcitriol|D	27.86	17
PUS7	54517	Leukemia, MESH:D015470		Busulfan|D	27.86	12
RGS20	8601	Leukemia, MESH:D015470		Cytarabine	27.86	86
RNF144A	9781	Leukemia, MESH:D015470		Arsenic Tr	27.86	40
STK38L	23012	Leukemia, MESH:D015470		Air Polluta	27.86	18
TMEM38B	55151	Leukemia, MESH:D015470		Dexameth	27.86	17
TNKS	8658	Leukemia, MESH:D015470		Bortezomi	27.86	33
IL13	3596	Leukemia, MESH:D015470		Air Polluta	27.85	25
MXRA5	25878	Leukemia, MESH:D015473		Arsenic|Ca	27.84	157
SOD1	6647	Leukemia, MESH:D015473		Aclarubici	27.84	268
CDH1	999	Leukemia, MESH:D015473		2-(2-chlo	27.83	269
EXOG	9941	Leukemia, MESH:D015470		Arsenic Tr	27.82	88
H4C11	8363	Leukemia, MESH:D015470		Arsenic Tr	27.82	22
PCYT1B	9468	Leukemia, MESH:D015470		Doxorubic	27.82	18
SULF2	55959	Leukemia, MESH:D015473		Arsenic|Ar	27.82	265
BHLHE40	8553	Leukemia, MESH:D015473		Arsenic|Ar	27.81	270
FTL	2512	Leukemia, MESH:D015470		Arsenic Tr	27.8	56
LPIN1	23175	Leukemia, MESH:D015470		Air Polluta	27.8	19
CD44	960	Leukemia, MESH:D01	marker/m	Arsenic|Ar	27.79	275
ELK1	2002	Leukemia, MESH:D015470		Arsenic Tr	27.79	36
ESRRA	2101	Leukemia, MESH:D015470		Bortezomi	27.79	32
LDLR	3949	Leukemia, MESH:D015470		Air Polluta	27.79	24
PDE4D	5144	Leukemia, MESH:D015470		Air Polluta	27.79	42
CD274	29126	Leukemia, MESH:D015473		Arsenic Tr	27.76	262
CRY2	1408	Leukemia, MESH:D015470		Arsenic Tr	27.76	23
FAM111A	63901	Leukemia, MESH:D015470		Cytarabine	27.76	89
FOXC1	2296	Leukemia, MESH:D015470		Calcitriol|D	27.76	25
ILF3	3609	Leukemia, MESH:D015470		Cyclophos	27.76	19
KDELR3	11015	Leukemia, MESH:D015470		Calcitriol|C	27.76	90
SLC9A3R1	9368	Leukemia, MESH:D015473		Arsenic|Ar	27.76	270
ST14	6768	Leukemia, MESH:D015470		Calcitriol|C	27.76	18
SYNJ2	8871	Leukemia, MESH:D015470		Arsenic Tr	27.76	19
TAX1BP3	30851	Leukemia, MESH:D015470		Daunorub	27.76	49
TRIM24	8805	Leukemia, MESH:D015470		Arsenic Tr	27.76	24
CREB1	1385	Leukemia, MESH:D015470		Alitretinoi	27.75	44
CCL11	6356	Leukemia, MESH:D015470		Chloroqui	27.73	21
POU5F1	5460	Leukemia, MESH:D015470		Arsenic Tr	27.73	35
ARHGEF16	27237	Leukemia, MESH:D015470		Calcitriol|D	27.72	17
CIAPIN1	57019	Leukemia, MESH:D015470		Doxorubic	27.72	27
DNMBP	23268	Leukemia, MESH:D015470		Arsenic Tr	27.72	22
FCGR3A	2214	Leukemia, MESH:D015470		Air Polluta	27.72	32
GPR37	2861	Leukemia, MESH:D015470		Arsenic Tr	27.72	29
ISCA1	81689	Leukemia, MESH:D015470		Azacitidine	27.72	17
NKD2	85409	Leukemia, MESH:D015470		Arsenic Tr	27.72	25
RAC1	5879	Leukemia, MESH:D015473		Arsenic|Ar	27.72	266
RELL1	768211	Leukemia, MESH:D015470		Dexameth	27.72	17
SKA2	348235	Leukemia, MESH:D015470		Calcitriol|D	27.72	19
STX2	2054	Leukemia, MESH:D015470		Carboplati	27.72	17
TRAM2	9697	Leukemia, MESH:D015470		Calcitriol|D	27.72	17
USP3	9960	Leukemia, MESH:D015470		Arsenic Tr	27.72	33
ABCD3	5825	Leukemia, MESH:D015470		Air Polluta	27.71	18
IL7R	3575	Leukemia, MESH:D015470		Bortezomi	27.71	26

EPHA1	2041	Leukemia, MESH:D015473	Antimony	27.7	160
FEN1	2237	Leukemia, MESH:D015473	arsenite|Ca	27.7	159
LTBR	4055	Leukemia, MESH:D015473	Arsenic Tr	27.7	261
PRDM2	7799	Leukemia, MESH:D015473	Arsenic|ars	27.7	163
TMPO	7112	Leukemia, MESH:D015473	Arsenic Tr	27.7	268
PLAU	5328	Leukemia, MESH:D015473	Arsenic|Ar	27.69	264
SRC	6714	Leukemia, MESH:D015470	Arsenic Tr	27.67	38
ADAMTS9	56999	Leukemia, MESH:D015470	Calcitriol|C	27.66	84
ERCC4	2072	Leukemia, MESH:D015470	Arsenic Tr	27.66	23
MYO5A	4644	Leukemia, MESH:D015470	Bortezomi	27.66	15
PTGIS	5740	Leukemia, MESH:D015470	Calcitriol|D	27.66	19
RABGGTB	5876	Leukemia, MESH:D015470	Benzene|C	27.66	96
JUNB	3726	Leukemia, MESH:D015473	alpha-Toc	27.65	268
KLF4	9314	Leukemia, MESH:D015473	Arsenic|Ar	27.63	265
NDUFS1	4719	Leukemia, MESH:D015470	Arsenic Tr	27.63	39
NFIA	4774	Leukemia, MESH:D015470	Arsenic Tr	27.63	24
PTK2B	2185	Leukemia, MESH:D015473	Arsenic|Ar	27.63	271
THY1	7070	Leukemia, MESH:D015473	Arsenic|ars	27.63	164
CSE1L	1434	Leukemia, MESH:D015473	Arsenic|ars	27.62	162
IL5	3567	Leukemia, MESH:D015470	Air Polluta	27.62	19
MSX2	4488	Leukemia, MESH:D015473	Arsenic Tr	27.62	261
BCL2A1A	12044	Leukemia, MESH:D015470	Alitretinoi	27.61	15
CENPW	387103	Leukemia, MESH:D015473	arsenite|Ca	27.61	158
CHRAC1	54108	Leukemia, MESH:D015470	Decitabine	27.61	21
FAM32A	26017	Leukemia, MESH:D015470	Dexameth	27.61	15
KCNQ1OT	10984	Leukemia, MESH:D015470	Doxorubic	27.61	17
LRIG1	26018	Leukemia, MESH:D015473	Antimony	27.61	162
MAPKBP1	23005	Leukemia, MESH:D015470	Dexameth	27.61	17
PTPRG	5793	Leukemia, MESH:D015473	Arsenic|ars	27.61	160
RAD54L	8438	Leukemia, MESH:D015473	Arsenic|ars	27.61	161
SYTL2	54843	Leukemia, MESH:D015473	Arsenic|Cy	27.61	163
TAFA5	25817	Leukemia, MESH:D015470	Arsenic Tr	27.61	18
WDHD1	11169	Leukemia, MESH:D015473	arsenite|Ca	27.61	159
ZNF462	58499	Leukemia, MESH:D015470	Calcitriol|D	27.61	15
ANXA8L1	728113	Leukemia, MESH:D015470	Cytarabine	27.6	77
DNAJB4	11080	Leukemia, MESH:D015470	Arsenic Tr	27.6	41
H2BC12L	54145	Leukemia, MESH:D015470	Doxorubic	27.6	18
SLPI	6590	Leukemia, MESH:D015470	Benzene|C	27.6	34
PTEN	5728	Leukemia, MESH:D007948	2-(2-amin	27.59	4
CP	1356	Leukemia, MESH:D015470	Arsenic Tr	27.58	30
HES1	3280	Leukemia, MESH:D015473	Arsenic|Ar	27.58	267
THRA	7067	Leukemia, MESH:D015470	Amsacrine	27.58	33
MACROH2	9555	Leukemia, MESH:D015470	Dexameth	27.57	16
MAD2L1	4085	Leukemia, MESH:D015473	Arsenic|Ar	27.57	267
MCOLN3	55283	Leukemia, MESH:D015470	Arsenic Tr	27.57	87
PALM2AK	445815	Leukemia, MESH:D015470	Arsenic Tr	27.57	19
PTPN18	26469	Leukemia, MESH:D015470	Dexameth	27.57	18
SCAF11	9169	Leukemia, MESH:D015470	Dronabinc	27.57	17
WNT3	7473	Leukemia, MESH:D015470	Decitabine	27.57	22
ARNT2	9915	Leukemia, MESH:D015470	Arsenic Tr	27.56	24
CDC7	8317	Leukemia, MESH:D015470	Arsenic Tr	27.56	24
EGR1	1958	Leukemia, MESH:D015473	Arsenic|Ar	27.56	270
FILIP1L	11259	Leukemia, MESH:D015470	Azacitidine	27.56	41
FZD1	8321	Leukemia, MESH:D015470	Arsenic Tr	27.56	32
GNG11	2791	Leukemia, MESH:D015470	Benzene|D	27.56	33
GSDME	1687	Leukemia, MESH:D015470	Benzene|D	27.56	33
NSD2	7468	Leukemia, MESH:D015470	Arsenic Tr	27.56	23
PLIN3	10226	Leukemia, MESH:D015470	Chloroqui	27.56	85

RABGAP1l	9910	Leukemia, MESH:D015470	Benzoates	27.56	18
RGL1	23179	Leukemia, MESH:D015470	Arsenic Tr	27.56	97
RPA2	6118	Leukemia, MESH:D015470	Air Polluta	27.56	15
SNX10	29887	Leukemia, MESH:D015470	Cyclophos	27.56	25
TOB1	10140	Leukemia, MESH:D015470	Dexameth	27.56	20
SLC8A1	6546	Leukemia, MESH:D015470	Air Polluta	27.55	33
TGIF1	7050	Leukemia, MESH:D015470	Alitretinoi	27.55	34
ULK1	8408	Leukemia, MESH:D015470	Arsenic Tr	27.55	19
FDFT1	2222	Leukemia, MESH:D015473	Antimony	27.54	160
TNFRSF1B	7133	Leukemia, MESH:D015470	Air Polluta	27.54	33
CCL8	6355	Leukemia, MESH:D015473	Arsenic|Ca	27.53	17
CASP12	1.01E+08	Leukemia, MESH:D015470	Arsenic Tr	27.52	27
TH	7054	Leukemia, MESH:D007948	2-(2-amin	27.52	4
ABCC4	10257	Leukemia, MESH:D015473	Arsenic|Ar	27.51	268
CDK5R1	8851	Leukemia, MESH:D007948	Arsenic Tr	27.51	4
CXCL14	9547	Leukemia, MESH:D015473	Arsenic Tr	27.51	264
DPYD	1806	Leukemia, MESH:D015470	Air Polluta	27.48	30
COX4I1	1327	Leukemia, MESH:D015470	Arsenic Tr	27.47	52
G6PD	2539	Leukemia, MESH:D015470	Alitretinoi	27.47	31
SHH	6469	Leukemia, MESH:D015470	Androgen	27.47	20
AMACR	23600	Leukemia, MESH:D015470	Arsenic Tr	27.45	19
CHST15	51363	Leukemia, MESH:D015470	Air Polluta	27.45	20
COL14A1	7373	Leukemia, MESH:D015470	Arsenic Tr	27.45	27
DIO2	1734	Leukemia, MESH:D015473	Arsenic|Bu	27.45	16
MMD	23531	Leukemia, MESH:D015470	Arsenic Tr	27.45	34
MT1H	4496	Leukemia, MESH:D015470	Arsenic Tr	27.45	31
PIGA	5277	Leukemia, MESH:D015470	Air Polluta	27.45	25
SMAD6	4091	Leukemia, MESH:D015470	Arsenic Tr	27.45	101
CEBPZ	10153	Leukemia, MESH:D015470	Arsenic Tr	27.44	48
IMMT	10989	Leukemia, MESH:D015470	Dexameth	27.44	25
MAPRE3	22924	Leukemia, MESH:D015470	Decitabine	27.44	25
OAS1	4938	Leukemia, MESH:D015473	Arsenic|ars	27.44	160
PTPRE	5791	Leukemia, MESH:D015473	Arsenic|Ar	27.44	269
RCOR3	55758	Leukemia, MESH:D015470	Dexameth	27.44	11
RIMS3	9783	Leukemia, MESH:D015470	Benzene|D	27.44	36
SECISBP2L	9728	Leukemia, MESH:D015470	Decitabine	27.44	22
SOX13	9580	Leukemia, MESH:D015470	Arsenic Tr	27.44	19
SURF4	6836	Leukemia, MESH:D015470	Benzene|C	27.44	97
TM6SF1	53346	Leukemia, MESH:D015470	Arsenic Tr	27.44	20
YPEL5	51646	Leukemia, MESH:D015473	Arsenic Tr	27.44	264
BRPF1	7862	Leukemia, MESH:D015470	Arsenic Tr	27.41	22
CBR1	873	Leukemia, MESH:D015470	Androgen	27.41	55
MOB3A	126308	Leukemia, MESH:D015470	Bortezomi	27.41	16
SRP54	6729	Leukemia, MESH:D015470	Benzene|D	27.41	28
STMN1	3925	Leukemia, MESH:D015473	Arsenic Tr	27.41	265
TUBA4A	7277	Leukemia, MESH:D015473	Arsenic|De	27.4	163
EGR3	1960	Leukemia, MESH:D015470	Arsenic Tr	27.39	37
MXD1	4084	Leukemia, MESH:D015470	Arsenic Tr	27.39	37
SFRP1	6422	Leukemia, MESH:D015470	Arsenic Tr	27.39	98
FLT1	2321	Leukemia, MESH:D015473	Arsenic Tr	27.37	263
FNBP1	23048	Leukemia, MESH:D015470	Dexameth	27.35	21
LPCAT1	79888	Leukemia, MESH:D015470	Arsenic Tr	27.35	22
PLEKHF1	79156	Leukemia, MESH:D015470	Decitabine	27.35	25
SOS1	6654	Leukemia, MESH:D015470	Air Polluta	27.35	21
ZEB2	9839	Leukemia, MESH:D015473	Arsenic|ars	27.34	160
CCL20	6364	Leukemia, MESH:D015473	Arsenic|Ar	27.32	266
INSIG2	51141	Leukemia, MESH:D015470	Calcitriol|D	27.31	22
ITGA4	3676	Leukemia, MESH:D015470	Alitretinoi	27.31	27

MYCN	4613	Leukemia, MESH:D015470	Arsenic Tr	27.31	35
AFDN	4301	Leukemia, MESH:D015470	Arsenic Tr	27.3	17
ANP32B	10541	Leukemia, MESH:D015470	Arsenic Tr	27.3	26
BSCL2	26580	Leukemia, MESH:D015470	Cyclophos	27.3	85
GREM2	64388	Leukemia, MESH:D015470	Dasatinib|	27.3	18
MAX	4149	Leukemia, MESH:D015470	Arsenic Tr	27.3	25
MSH3	4437	Leukemia, MESH:D015470	Cyclophos	27.3	16
NDC1	55706	Leukemia, MESH:D015470	Dasatinib|	27.3	18
OCIAD2	132299	Leukemia, MESH:D015470	Decitabine	27.3	18
RAB29	8934	Leukemia, MESH:D015470	Dexameth	27.3	19
SIDT2	51092	Leukemia, MESH:D015470	Decitabine	27.3	25
STK17A	9263	Leukemia, MESH:D015470	Arsenic Tr	27.3	30
TPST1	8460	Leukemia, MESH:D015470	Calcitriol|C	27.3	80
BAG3	9531	Leukemia, MESH:D015470	ABT-737|A	27.29	98
CCR2	729230	Leukemia, MESH:D015470	Air Polluta	27.29	28
NCOA2	10499	Leukemia, MESH:D015470	Arsenic Tr	27.29	55
TNFAIP6	7130	Leukemia, MESH:D015470	Arsenic Tr	27.29	43
AKAP12	9590	Leukemia, MESH:D015473	Arsenic Tr	27.28	263
ABCA12	26154	Leukemia, MESH:D015473	Arsenic|ars	27.27	159
CSTA	1475	Leukemia, MESH:D015473	Arsenic Tr	27.27	262
PLAAT4	5920	Leukemia, MESH:D015473	arsenite|Ca	27.27	160
SYNJ2	8871	Leukemia, MESH:D015473	Arsenic|Ar	27.27	264
TFF3	7033	Leukemia, MESH:D015473	arsenite|Cl	27.27	21
TRIO	7204	Leukemia, MESH:D01 marker/m	Bortezomi	27.25	19
ATP6V1A	523	Leukemia, MESH:D015470	Air Polluta	27.25	30
GPX7	2882	Leukemia, MESH:D015470	Arsenic Tr	27.25	24
MLLT11	10962	Leukemia, MESH:D015470	Arsenic Tr	27.25	30
SYNM	23336	Leukemia, MESH:D015470	Calcitriol|D	27.25	15
GSK3B	2932	Leukemia, MESH:D015473	Arsenic|Ar	27.24	270
EPHA2	1969	Leukemia, MESH:D015470	Dasatinib|	27.23	27
GATM	2628	Leukemia, MESH:D015470	Dexameth	27.23	22
MEF2C	4208	Leukemia, MESH:D015470	Decitabine	27.23	27
VASP	7408	Leukemia, MESH:D015470	Bortezomi	27.23	23
ATP1A1	476	Leukemia, MESH:D015470	Calcitriol|C	27.22	32
PRKDC	5591	Leukemia, MESH:D015473	Arsenic|ars	27.22	165
RGS4	5999	Leukemia, MESH:D015473	arsenite|Cy	27.22	160
ABCC10	89845	Leukemia, MESH:D015470	Docetaxel|	27.21	16
CA8	767	Leukemia, MESH:D015470	Dexameth	27.21	12
HSPA8	3312	Leukemia, MESH:D015473	Antimony	27.21	266
RCCD1	91433	Leukemia, MESH:D015470	Calcitriol|D	27.21	21
SCAF1	58506	Leukemia, MESH:D015470	Arsenic Tr	27.21	22
STX4	6810	Leukemia, MESH:D015470	Dexameth	27.21	13
TRMT5	57570	Leukemia, MESH:D015470	Arsenic Tr	27.21	27
WIPF2	147179	Leukemia, MESH:D015470	Dexameth	27.21	16
MSMO1	6307	Leukemia, MESH:D015473	Antineopla	27.19	262
PRDM16	63976	Leukemia, MESH:D015473	Arsenic|ars	27.19	166
RABGGTB	5876	Leukemia, MESH:D015473	Cytarabine	27.19	162
ANO3	63982	Leukemia, MESH:D015470	Arsenic Tr	27.16	42
COL8A2	1296	Leukemia, MESH:D015470	Alitretinoi	27.16	23
CYP26B1	56603	Leukemia, MESH:D015470	Alitretinoi	27.16	25
ETS2	2114	Leukemia, MESH:D015470	Arsenic Tr	27.16	23
LMO2	4005	Leukemia, MESH:D015470	Arsenic Tr	27.16	22
MAN1A1	4121	Leukemia, MESH:D015470	Air Polluta	27.16	21
NLE1	54475	Leukemia, MESH:D015470	Dexameth	27.16	16
POLR1H	30834	Leukemia, MESH:D015470	Doxorubic	27.16	28
SIN3A	25942	Leukemia, MESH:D015470	Arsenic Tr	27.16	29
SLC31A1	1317	Leukemia, MESH:D015470	Bortezomi	27.16	23
SOX4	6659	Leukemia, MESH:D015473	Arsenic Tr	27.16	262

SP100	6672	Leukemia, MESH:D015470	Dexameth	27.16	20
SPINT1	6692	Leukemia, MESH:D015470	Arsenic Tr	27.16	21
TFPI	7035	Leukemia, MESH:D015473	Arsenic|Cy	27.16	162
TSPO	706	Leukemia, MESH:D015470	Cytarabine	27.16	93
WDR26	80232	Leukemia, MESH:D015470	Dexameth	27.16	18
CDKN2D	1032	Leukemia, MESH:D015470	Arsenic Tr	27.15	24
CLIC1	1192	Leukemia, MESH:D015470	Decitabine	27.15	24
CLMN	79789	Leukemia, MESH:D015470	Calcitriol|D	27.15	18
DBF4	10926	Leukemia, MESH:D015470	Calcitriol|E	27.15	22
DEPDC1B	55789	Leukemia, MESH:D015470	Calcitriol|D	27.15	19
EPOR	2057	Leukemia, MESH:D015470	Arsenic Tr	27.15	41
FAM43A	131583	Leukemia, MESH:D015470	Bortezomi	27.15	21
GPC3	2719	Leukemia, MESH:D015470	Arsenic Tr	27.15	26
MXI1	4601	Leukemia, MESH:D015470	Cyclophos	27.15	34
ASPM	259266	Leukemia, MESH:D015473	Arsenic Tr	27.11	263
CHUK	1147	Leukemia, MESH:D015473	4'-methox	27.11	160
PECAM1	5175	Leukemia, MESH:D015473	2-(2-chlor	27.11	267
CYP3A11	13112	Leukemia, MESH:D015470	Alitretinoi	27.1	25
FZD1	8321	Leukemia, MESH:D015473	Arsenic Tr	27.1	263
IL4R	3566	Leukemia, MESH:D015473	Arsenic|Ar	27.1	264
MYH11	4629	Leukemia, MESH:D015473	Arsenic|Da	27.1	163
NSD2	7468	Leukemia, MESH:D015473	Arsenic|Ar	27.1	265
PAPPA	5069	Leukemia, MESH:D015473	Arsenic Tr	27.1	264
ACSL3	2181	Leukemia, MESH:D015470	Calcitriol|D	27.08	42
CENPE	1062	Leukemia, MESH:D015470	Calcitriol|D	27.08	20
P2RX7	5027	Leukemia, MESH:D015470	Air Polluta	27.08	45
BMP2	650	Leukemia, MESH:D015473	Arsenic|Ar	27.07	266
PRKCB	5579	Leukemia, MESH:D015473	Arsenic|Ar	27.07	270
ADAMTS5	11096	Leukemia, MESH:D015470	Arsenic Tr	27.06	24
DLAT	1737	Leukemia, MESH:D015470	Decitabine	27.06	27
PDE4A	5141	Leukemia, MESH:D015470	Arsenic Tr	27.06	36
TLR6	10333	Leukemia, MESH:D015470	Air Polluta	27.06	31
YWHAH	7533	Leukemia, MESH:D015470	Arsenic Tr	27.06	23
TNFRSF1A	7132	Leukemia, MESH:D015473	Arsenic|Ar	27.05	266
TFAM	7019	Leukemia, MESH:D015470	Calcitriol|C	27.04	31
ATP6V0B	533	Leukemia, MESH:D015470	Arsenic Tr	27.03	16
AXIN1	8312	Leukemia, MESH:D015470	Benzene|D	27.03	36
BCAS3	54828	Leukemia, MESH:D015470	Dexameth	27.03	17
CELSR1	9620	Leukemia, MESH:D015470	Arsenic Tr	27.03	23
COL17A1	1308	Leukemia, MESH:D015470	Alitretinoi	27.03	18
JMY	133746	Leukemia, MESH:D015470	Air Polluta	27.03	17
PRKX	5613	Leukemia, MESH:D015470	Benzene|D	27.03	67
PRPF8	10594	Leukemia, MESH:D015470	Arsenic Tr	27.03	32
RASGRF1	5923	Leukemia, MESH:D015470	Arsenic Tr	27.03	18
RFLNB	359845	Leukemia, MESH:D015470	Calcitriol|D	27.03	19
RIT1	6016	Leukemia, MESH:D015470	Benzene|B	27.03	26
TBL1X	6907	Leukemia, MESH:D015470	Arsenic Tr	27.03	29
CENPO	79172	Leukemia, MESH:D015470	Azacitidine	27.02	18
DFFB	1677	Leukemia, MESH:D015473	Arsenic|Ar	27.02	264
DIAPH3	81624	Leukemia, MESH:D015473	Arsenic Tr	27.02	262
FBXO41	150726	Leukemia, MESH:D015470	Decitabine	27.02	18
LONRF2	164832	Leukemia, MESH:D015470	Cyclophos	27.02	15
LXN	56925	Leukemia, MESH:D015473	Calcitriol|D	27.02	156
MED7	9443	Leukemia, MESH:D015470	Dexameth	27.02	14
NUDT7	283927	Leukemia, MESH:D015473	Arsenic|Ar	27.02	266
ODC1	4953	Leukemia, MESH:D015473	Arsenic|Ar	27.02	271
PAPOLG	64895	Leukemia, MESH:D015470	Air Polluta	27.02	14
QSOX2	169714	Leukemia, MESH:D015470	Air Polluta	27.02	17

TBP	6908	Leukemia, MESH:D015470	Arsenic Tr	27.02	29
ZNF532	55205	Leukemia, MESH:D015470	Dexameth	27.02	14
C5	727	Leukemia, MESH:D015470	Arsenic Tr	27.01	43
PITX1	5307	Leukemia, MESH:D015473	Antimony	27.01	158
ACTA2	59	Leukemia, MESH:D015473	Arsenic|Ar	27	271
MRPL24	79590	Leukemia, MESH:D015473	Arsenic|Ar	27	266
ETS1	2113	Leukemia, MESH:D015473	Arsenic|Ar	26.99	266
PLPP3	8613	Leukemia, MESH:D015473	Arsenic|Ar	26.99	264
ELK1	2002	Leukemia, MESH:D007948	2-(2-amin	26.97	3
MAP1LC3(	440738	Leukemia, MESH:D015470	Decitabine	26.97	14
CALB2	794	Leukemia, MESH:D015470	Arsenic Tr	26.96	89
DFFA	1676	Leukemia, MESH:D015470	Aclarubici	26.96	70
G3BP1	10146	Leukemia, MESH:D015470	Cyclophos	26.96	18
P4HA2	8974	Leukemia, MESH:D015470	Clioquinol	26.96	86
PRKCH	5583	Leukemia, MESH:D015470	Air Polluta	26.96	35
RRAGD	58528	Leukemia, MESH:D015470	Arsenic Tr	26.96	38
CDKN2A	1029	Leukemia, MESH:D015473	Arsenic|Ar	26.95	271
CEBPD	1052	Leukemia, MESH:D015473	Arsenic Tr	26.94	269
IGFBP4	3487	Leukemia, MESH:D015473	arsenic dis	26.94	264
NES	10763	Leukemia, MESH:D015473	2-(2-chlor	26.94	270
NFE2L1	4779	Leukemia, MESH:D015473	Arsenic|Ar	26.94	163
SERPINB5	5268	Leukemia, MESH:D015473	Arsenic|Ar	26.94	162
FGF7	2252	Leukemia, MESH:D007948	2-(2-amin	26.93	3
MAP3K1	4214	Leukemia, MESH:D015470	Calcitriol|C	26.93	23
TUBB6	84617	Leukemia, MESH:D015470	Calcitriol|D	26.93	21
PCK2	5106	Leukemia, MESH:D015470	Arsenic Tr	26.92	37
GSTA3	2940	Leukemia, MESH:D015470	15-deoxy-	26.91	44
NPPB	4879	Leukemia, MESH:D004915	Cytarabine	26.91	4
ASB2	51676	Leukemia, MESH:D015470	Azacitidine	26.9	18
CLEC2D	29121	Leukemia, MESH:D015470	Arsenic Tr	26.9	20
HERC6	55008	Leukemia, MESH:D015470	Decitabine	26.9	26
HNF1B	6928	Leukemia, MESH:D015470	Dexameth	26.9	14
ITPK1	3705	Leukemia, MESH:D015470	Dexameth	26.9	16
KIF3A	11127	Leukemia, MESH:D015470	Arsenic Tr	26.9	22
MXRA7	439921	Leukemia, MESH:D015470	Arsenic Tr	26.9	87
MYO6	4646	Leukemia, MESH:D015470	Arsenic Tr	26.9	23
NPHS2	7827	Leukemia, MESH:D015470	Arsenic Tr	26.9	23
PAIP1	10605	Leukemia, MESH:D015470	Cyclophos	26.9	19
PILRA	29992	Leukemia, MESH:D015470	Arsenic Tr	26.9	21
THBS1	7057	Leukemia, MESH:D015473	arsenite|Cy	26.9	161
TMEM47	83604	Leukemia, MESH:D015470	Decitabine	26.9	39
UNG	7374	Leukemia, MESH:D015473	arsenite|Ca	26.88	159
BRINP1	1620	Leukemia, MESH:D015473	Arsenic Tr	26.87	263
ACSL5	51703	Leukemia, MESH:D015470	Bezafibrat	26.86	20
AQP3	360	Leukemia, MESH:D015470	Arsenic Tr	26.86	29
CENPU	79682	Leukemia, MESH:D015473	Calcitriol|D	26.86	157
CXCL3	2921	Leukemia, MESH:D015470	Calcitriol|C	26.86	21
DLL1	28514	Leukemia, MESH:D015470	Benzene|D	26.86	33
ENG	2022	Leukemia, MESH:D015470	Arsenic Tr	26.86	56
EREG	2069	Leukemia, MESH:D015470	Air Polluta	26.86	30
FANCA	2175	Leukemia, MESH:D015473	Arsenic|Ar	26.86	267
KCNJ8	3764	Leukemia, MESH:D015470	Decitabine	26.86	24
RSAD2	91543	Leukemia, MESH:D015470	Calcitriol|D	26.86	22
SLC25A20	788	Leukemia, MESH:D015470	Air Polluta	26.86	19
TYROBP	7305	Leukemia, MESH:D015473	Calcitriol|D	26.86	160
BECN1	8678	Leukemia, MESH:D015473	Arsenic|Ar	26.83	269
SLC7A11	23657	Leukemia, MESH:D015473	alpha-Toc	26.83	267
SMAD7	4092	Leukemia, MESH:D015473	Arsenic|Ca	26.83	159

SPHK1	8877	Leukemia, MESH:D015473		Arsenic|ars	26.83	165
SYTL3	94120	Leukemia, MESH:D015470		Calcitriol|D	26.83	19
TMEM267	64417	Leukemia, MESH:D015470		Arsenic Tr	26.83	23
ZNF703	80139	Leukemia, MESH:D015470		Doxorubic	26.83	17
ATF6	22926	Leukemia, MESH:D015473		Arsenic|Ar	26.82	268
HMOX1	3162	Leukemia, MESH:D015473		alpha-Toc	26.79	273
FAM43A	131583	Leukemia, MESH:D015473		Arsenic|ars	26.78	160
KYNU	8942	Leukemia, MESH:D015470		Arsenic Tr	26.78	92
SIK1	150094	Leukemia, MESH:D015470		Air Polluta	26.78	20
WFDC2	10406	Leukemia, MESH:D015473		Arsenic|Ca	26.78	159
IFI30	10437	Leukemia, MESH:D01	marker/m	Decitabine	26.77	21
AGPAT4	56895	Leukemia, MESH:D015470		Arsenic Tr	26.77	22
AKAP9	10142	Leukemia, MESH:D015470		Arsenic Tr	26.77	21
ASL	435	Leukemia, MESH:D015470		Arsenic Tr	26.77	19
ECM1	1893	Leukemia, MESH:D015470		Arsenic Tr	26.77	25
EIF4E	1977	Leukemia, MESH:D015473		Arsenic Tr	26.77	264
FBXL20	84961	Leukemia, MESH:D015470		Decitabine	26.77	25
FHL2	2274	Leukemia, MESH:D015473		Arsenic Tr	26.77	264
GDNF	2668	Leukemia, MESH:D015473		Arsenic|Ar	26.77	266
H4C8	8365	Leukemia, MESH:D015470		Arsenic Tr	26.77	22
IL32	9235	Leukemia, MESH:D015470		Calcitriol|D	26.77	24
IL6R	3570	Leukemia, MESH:D015473		Arsenic|ars	26.77	164
KIF21B	23046	Leukemia, MESH:D015470		Arsenic Tr	26.77	21
LRPAP1	4043	Leukemia, MESH:D015470		Arsenic Tr	26.77	22
NDUFS5	4725	Leukemia, MESH:D015470		Doxorubic	26.77	28
OXTR	5021	Leukemia, MESH:D015470		Azacitidine	26.77	28
PRKAR1A	5573	Leukemia, MESH:D015470		Androgen	26.77	23
PTH1R	5745	Leukemia, MESH:D015470		Benzene|C	26.77	95
SMYD3	64754	Leukemia, MESH:D015470		Arsenic Tr	26.77	28
SNRPF	6636	Leukemia, MESH:D015470		Arsenic Tr	26.77	16
TOB2	10766	Leukemia, MESH:D015470		Dexameth	26.77	17
DOK4	55715	Leukemia, MESH:D015473		Calcitriol|C	26.75	157
IGFBP2	3485	Leukemia, MESH:D015470		Alitretinoi	26.74	32
CKS2	1164	Leukemia, MESH:D015473		Antimony	26.72	163
TYMS	7298	Leukemia, MESH:D004915		Cytarabine	26.72	3
ATRX	546	Leukemia, MESH:D015470		Benzene|D	26.71	49
KLHL24	54800	Leukemia, MESH:D015470		Arsenic Tr	26.71	31
ANTXR1	84168	Leukemia, MESH:D015473		Arsenic|Ar	26.7	262
IFI6	2537	Leukemia, MESH:D015473		Arsenic Tr	26.7	262
IRF6	3664	Leukemia, MESH:D015473		Arsenic Tr	26.7	264
KCTD12	115207	Leukemia, MESH:D015473		Arsenic Tr	26.7	266
NGEF	25791	Leukemia, MESH:D015473		Arsenic|ars	26.7	25
TGFB3	7043	Leukemia, MESH:D015470		Calcitriol|C	26.68	18
GSR	2936	Leukemia, MESH:D015473		alpha-Toc	26.67	269
MFAP4	4239	Leukemia, MESH:D015470		Calcitriol|C	26.67	85
RET	5979	Leukemia, MESH:D015473		Arsenic|ca	26.67	159
ROBO1	6091	Leukemia, MESH:D015470		Arsenic Tr	26.67	36
SMOC1	64093	Leukemia, MESH:D015470		Dexameth	26.67	13
ZNF600	162966	Leukemia, MESH:D015470		Arsenic Tr	26.67	20
ZNF660	285349	Leukemia, MESH:D015470		Dexameth	26.67	14
LITAF	9516	Leukemia, MESH:D015473		Arsenic|Ar	26.66	266
ADAM22	53616	Leukemia, MESH:D015470		Calcitriol|D	26.65	18
APOL1	8542	Leukemia, MESH:D015470		Bortezomi	26.65	11
BCO1	53630	Leukemia, MESH:D015470		Alitretinoi	26.65	16
FXYD3	5349	Leukemia, MESH:D015470		Calcitriol|D	26.65	12
GATA1	2623	Leukemia, MESH:D015470		Aclarubici	26.65	28
IPO13	9670	Leukemia, MESH:D015470		Calcitriol|D	26.65	12
PMEL	6490	Leukemia, MESH:D015470		Cytarabine	26.65	86

PPM1M	132160	Leukemia, MESH:D015470		Calcitriol|C	26.65	78
SLC35F2	54733	Leukemia, MESH:D015470		Dexameth	26.65	24
SLC9A9	285195	Leukemia, MESH:D015470		Dexameth	26.65	20
TEX10	54881	Leukemia, MESH:D015470		Dexameth	26.65	14
TEX261	113419	Leukemia, MESH:D015470		Arsenic Tr	26.65	24
CORIN	10699	Leukemia, MESH:D015473		Arsenic|Ar	26.63	268
CEACAM6	4680	Leukemia, MESH:D015473		Arsenic|Ca	26.62	160
DHCR24	1718	Leukemia, MESH:D015470		Androgen	26.62	103
IFRD1	3475	Leukemia, MESH:D015470		Arsenic Tr	26.62	39
LIMCH1	22998	Leukemia, MESH:D015473		Arsenic|Ca	26.62	159
MT1A	4489	Leukemia, MESH:D015473		Arsenic|Ar	26.62	179
RP2	6102	Leukemia, MESH:D015473		Arsenic|Ar	26.62	266
ATXN1	6310	Leukemia, MESH:D015470		Daunorub	26.58	44
CAVIN2	8436	Leukemia, MESH:D015470		Air Polluta	26.58	24
HIGD1A	25994	Leukemia, MESH:D015470		Arsenic Tr	26.58	26
MAP3K14	9020	Leukemia, MESH:D015470		Calcitriol|D	26.58	16
MTHFD1	4522	Leukemia, MESH:D015470		Arsenic Tr	26.58	31
NCOA7	135112	Leukemia, MESH:D015470		Arsenic Tr	26.58	26
RECK	8434	Leukemia, MESH:D015470		Dasatinib|I	26.58	20
TLR3	7098	Leukemia, MESH:D007948		2-(2-amin	26.57	3
NPM1	4869	Leukemia, MESH:D01	marker/m	Arsenic|Ar	26.56	267
IL1A	3552	Leukemia, MESH:D015473		alpha-Toc	26.56	268
DBN1	1627	Leukemia, MESH:D015473		Arsenic Tr	26.55	264
GAS7	8522	Leukemia, MESH:D015473		Arsenic|Ar	26.55	266
MTR	4548	Leukemia, MESH:D015473		Arsenic|Ar	26.55	265
TF	7018	Leukemia, MESH:D015470		Busulfan|C	26.55	23
ADM	133	Leukemia, MESH:D015473		Antimony	26.54	266
AFF3	3899	Leukemia, MESH:D015470		Calcitriol|D	26.52	22
CTTNBP2N	55917	Leukemia, MESH:D015470		Arsenic Tr	26.52	23
CYP2U1	113612	Leukemia, MESH:D015470		Bortezomi	26.52	18
KBTBD11	9920	Leukemia, MESH:D015470		Arsenic Tr	26.52	16
KDM6A	7403	Leukemia, MESH:D015470		Doxorubic	26.52	19
NOP2	4839	Leukemia, MESH:D015470		Benzoates	26.52	15
PKDCC	91461	Leukemia, MESH:D015470		Decitabine	26.52	22
RNASE1	6035	Leukemia, MESH:D015470		Air Polluta	26.52	18
RNF128	79589	Leukemia, MESH:D015470		Arsenic Tr	26.52	22
SUB1	10923	Leukemia, MESH:D015470		Arsenic Tr	26.52	22
TNFSF13B	10673	Leukemia, MESH:D015470		Air Polluta	26.52	21
TRIM6	117854	Leukemia, MESH:D015470		Calcitriol|C	26.52	78
WDFY3	23001	Leukemia, MESH:D015470		Dexameth	26.52	19
SFN	2810	Leukemia, MESH:D015473		Arsenic|Ar	26.51	269
LDHA	3939	Leukemia, MESH:D015473		alpha-Toc	26.5	268
NOX4	50507	Leukemia, MESH:D015473		Arsenic|Ar	26.5	271
CDA	978	Leukemia, MESH:D015470		Calcitriol|C	26.49	19
GPD1	2819	Leukemia, MESH:D015470		Arsenic Tr	26.49	22
IL1R2	7850	Leukemia, MESH:D015470		Arsenic Tr	26.49	43
KCNQ1	3784	Leukemia, MESH:D015470		Arsenic Tr	26.49	54
LAMP2	3920	Leukemia, MESH:D015470		Chloroqui	26.49	20
OXCT1	5019	Leukemia, MESH:D015470		Air Polluta	26.49	29
PSMB4	5692	Leukemia, MESH:D015470		Air Polluta	26.49	25
UBE2I	7329	Leukemia, MESH:D015470		Arsenic Tr	26.49	26
WSB1	26118	Leukemia, MESH:D015470		Calcitriol|D	26.49	21
AKAP9	10142	Leukemia, MESH:D015473		Arsenic|Ar	26.47	268
BLM	641	Leukemia, MESH:D015473		Arsenic|ars	26.47	161
CLDN11	5010	Leukemia, MESH:D015473		Arsenic|Ar	26.47	266
COL8A1	1295	Leukemia, MESH:D015473		Arsenic|ars	26.47	159
CYP1B1	1545	Leukemia, MESH:D015473		Antimony	26.47	271
FCER1G	2207	Leukemia, MESH:D015473		Arsenic Tr	26.47	266

MIR142	406934	Leukemia, MESH:D015470		Air Polluta	26.47	34
RASL10B	91608	Leukemia, MESH:D015470		Dexameth	26.47	15
SPINK5	11005	Leukemia, MESH:D015470		(+)-JQ1 cc	26.47	13
SYNE3	161176	Leukemia, MESH:D015470		Dexameth	26.47	16
TGFBR3	7049	Leukemia, MESH:D015473		Arsenic Tr	26.47	265
CD55	1604	Leukemia, MESH:D015473		Arsenic Tr	26.45	265
MGLL	11343	Leukemia, MESH:D015470		Bezafibrat	26.44	22
GJB1	2705	Leukemia, MESH:D015470		Benzene|C	26.42	35
LIPG	9388	Leukemia, MESH:D015470		Bezafibrat	26.42	28
SLC1A1	6505	Leukemia, MESH:D015470		Calcitriol|D	26.42	19
ADCY7	113	Leukemia, MESH:D01	marker/m	Calcitriol|D	26.4	19
ANAPC1	64682	Leukemia, MESH:D015470		Dexameth	26.4	19
CALB1	793	Leukemia, MESH:D015470		Calcitriol|C	26.4	80
DAPK2	23604	Leukemia, MESH:D015470		Arsenic Tr	26.4	27
DDR2	4921	Leukemia, MESH:D015470		Arsenic Tr	26.4	89
ECHDC2	55268	Leukemia, MESH:D015470		Decitabine	26.4	24
EIF4A2	1974	Leukemia, MESH:D015470		Benzene|C	26.4	35
FANCC	2176	Leukemia, MESH:D015470		Arsenic Tr	26.4	25
GHR	2690	Leukemia, MESH:D015473		arsenite|ca	26.4	161
HNRNPA3	220988	Leukemia, MESH:D015470		Cytarabine	26.4	83
KIF1B	23095	Leukemia, MESH:D015470		Benzene|C	26.4	31
LACTB2	51110	Leukemia, MESH:D015470		Air Polluta	26.4	21
LARP7	51574	Leukemia, MESH:D015470		Decitabine	26.4	23
NDST1	3340	Leukemia, MESH:D015470		Arsenic Tr	26.4	18
OLFML2A	169611	Leukemia, MESH:D015470		Arsenic Tr	26.4	21
POLR3E	55718	Leukemia, MESH:D015470		Air Polluta	26.4	20
PPIF	10105	Leukemia, MESH:D015470		Arsenic Tr	26.4	25
PPP3CB	5532	Leukemia, MESH:D015470		Arsenic Tr	26.4	36
ST3GAL4	6484	Leukemia, MESH:D015470		Benzoates	26.4	16
TLE2	7089	Leukemia, MESH:D015470		Arsenic Tr	26.4	21
TMEFF2	23671	Leukemia, MESH:D015470		Air Polluta	26.4	16
WWC1	23286	Leukemia, MESH:D015470		Arsenic Tr	26.4	22
C9ORF85	138241	Leukemia, MESH:D015470		Bortezomi	26.39	14
FANCI	55215	Leukemia, MESH:D015473		arsenite|Ca	26.39	158
MERTK	10461	Leukemia, MESH:D015473		arsenic dis	26.39	267
MFAP4	4239	Leukemia, MESH:D015473		Calcitriol|C	26.39	157
RARRES1	5918	Leukemia, MESH:D015473		Arsenic|Ca	26.39	159
ZNF438	220929	Leukemia, MESH:D015470		Dexameth	26.39	15
ZPLD1	131368	Leukemia, MESH:D015470		Dexameth	26.39	14
ANXA1	301	Leukemia, MESH:D015473		Arsenic|Ar	26.38	268
C3ORF38	285237	Leukemia, MESH:D015470		Dexameth	26.38	14
CRYAB	1410	Leukemia, MESH:D015473		Arsenic|Ar	26.38	270
FAM171A1	221061	Leukemia, MESH:D015473		Arsenic|ars	26.38	157
NOC2L	26155	Leukemia, MESH:D015473		Arsenic|De	26.38	158
NR0B2	8431	Leukemia, MESH:D015470		2-(2-chlor	26.36	20
BUB1	699	Leukemia, MESH:D015473		arsenite|Ca	26.35	157
HBEGF	1839	Leukemia, MESH:D015473		Arsenic Tr	26.35	263
ID1	3397	Leukemia, MESH:D015473		Antimony	26.35	266
PLA2G2A	5320	Leukemia, MESH:D015470		Decitabine	26.35	23
SLC29A1	2030	Leukemia, MESH:D015470		Arsenic Tr	26.35	91
SLC6A9	6536	Leukemia, MESH:D015470		Arsenic Tr	26.35	26
MIR21	406991	Leukemia, MESH:D007948		2-(2-amin	26.34	3
MAP3K14	9020	Leukemia, MESH:D015473		arsenite|Ca	26.32	158
POLD1	5424	Leukemia, MESH:D015473		Arsenic|Ar	26.32	265
IL17A	3605	Leukemia, MESH:D01	marker/m	Arsenic|Ar	26.31	165
ABHD2	11057	Leukemia, MESH:D015470		Bortezomi	26.31	20
DUSP6	1848	Leukemia, MESH:D015470		Azacitidine	26.31	28
XPA	7507	Leukemia, MESH:D015470		Arsenic Tr	26.31	39

CD74	972	Leukemia, MESH:D015473	Arsenic Tr	26.3	267
DHX33	56919	Leukemia, MESH:D015470	Dexameth	26.29	17
DUS2	54920	Leukemia, MESH:D015470	Arsenic Tr	26.29	21
FBXW5	54461	Leukemia, MESH:D015470	Dexameth	26.29	10
KDM5C	8242	Leukemia, MESH:D015470	Bortezomi	26.29	22
MAP3K10	4294	Leukemia, MESH:D015470	Calcitriol|D	26.29	14
RBM7	10179	Leukemia, MESH:D015470	Dexameth	26.29	15
ANXA8	653145	Leukemia, MESH:D015470	Cytarabine	26.28	79
IRX3	79191	Leukemia, MESH:D015470	Calcitriol|D	26.28	23
ITGB3BP	23421	Leukemia, MESH:D015470	Arsenic Tr	26.28	19
PPP3CA	5530	Leukemia, MESH:D015470	Arsenic Tr	26.28	26
PRR5L	79899	Leukemia, MESH:D015470	Air Polluta	26.28	18
RIPOR2	9750	Leukemia, MESH:D015470	Air Polluta	26.28	18
SLC27A3	11000	Leukemia, MESH:D015470	Dexameth	26.28	24
TMCC3	57458	Leukemia, MESH:D015470	Calcitriol|D	26.28	15
VRK1	7443	Leukemia, MESH:D015470	Calcitriol|D	26.28	14
WNT5B	81029	Leukemia, MESH:D015470	Alitretinoi	26.28	13
IER3	8870	Leukemia, MESH:D015473	Arsenic|ars	26.27	166
NOX1	27035	Leukemia, MESH:D007948	2-(2-amin	26.27	3
VCPIP1	80124	Leukemia, MESH:D015473	Arsenic|Ar	26.26	263
CDK5	1020	Leukemia, MESH:D015473	alvocidib|A	26.25	267
KCNQ1	3784	Leukemia, MESH:D015473	Arsenic|Ar	26.25	270
UBE2I	7329	Leukemia, MESH:D015473	Arsenic|Ar	26.25	262
JAK1	3716	Leukemia, MESH:D015473	Arsenic|Ar	26.24	271
PLAUR	5329	Leukemia, MESH:D015473	Arsenic|Ar	26.23	268
ADD3	120	Leukemia, MESH:D015470	Dexameth	26.22	18
DEPTOR	64798	Leukemia, MESH:D015470	Cytarabine	26.22	82
TPP1	1200	Leukemia, MESH:D015470	Arsenic Tr	26.22	43
UQCRC1	7384	Leukemia, MESH:D015470	Air Polluta	26.22	18
GRIA1	2890	Leukemia, MESH:D015470	Arsenic Tr	26.21	24
HPRT1	3251	Leukemia, MESH:D015470	1-(2-hydr	26.21	21
MAP2K2	5605	Leukemia, MESH:D015470	2-(2-chlor	26.21	31
PARP1	142	Leukemia, MESH:D004915	Cytarabine	26.21	4
SERPINH1	871	Leukemia, MESH:D015470	Arsenic Tr	26.21	92
DGAT2	84649	Leukemia, MESH:D015473	Buthionine	26.19	155
EFNA1	1942	Leukemia, MESH:D015473	Arsenic|Ar	26.19	268
APOE	348	Leukemia, MESH:D015473	alpha-Toc	26.18	267
EIF4A2	1974	Leukemia, MESH:D015473	Arsenic|Ca	26.17	164
STAT4	6775	Leukemia, MESH:D015473	Arsenic|Ar	26.17	161
HOXB9	3219	Leukemia, MESH:D015470	Arsenic Tr	26.16	20
KMT5B	51111	Leukemia, MESH:D015470	Air Polluta	26.16	19
MADD	8567	Leukemia, MESH:D015470	Dexameth	26.16	17
MNS1	55329	Leukemia, MESH:D015470	Calcitriol|D	26.16	14
PKIA	5569	Leukemia, MESH:D015470	Bortezomi	26.16	83
PLEKHA5	54477	Leukemia, MESH:D015470	Dexameth	26.16	19
POLH	5429	Leukemia, MESH:D015470	Air Polluta	26.16	13
S100A1	6271	Leukemia, MESH:D015470	Alitretinoi	26.16	17
SLA	6503	Leukemia, MESH:D015470	Arsenic Tr	26.16	92
SLC17A5	26503	Leukemia, MESH:D015470	Benzene|C	26.16	26
STAMBPL1	57559	Leukemia, MESH:D015470	Air Polluta	26.16	20
TMTC1	83857	Leukemia, MESH:D015470	Arsenic Tr	26.16	21
TNFRSF14	8764	Leukemia, MESH:D015470	Arsenic Tr	26.16	29
CETP	1071	Leukemia, MESH:D015473	Arsenic Tr	26.15	261
FAM111B	374393	Leukemia, MESH:D015473	Calcitriol|D	26.15	156
FOXO1	2308	Leukemia, MESH:D015473	Arsenic Tr	26.15	265
IRX4	50805	Leukemia, MESH:D015473	Calcitriol|D	26.15	157
NR0B2	8431	Leukemia, MESH:D015473	2-(2-chlor	26.15	158
SCAMP5	192683	Leukemia, MESH:D015473	Arsenic|Ar	26.15	171

ACSL4	2182	Leukemia, MESH:D015470	Bezafibrat	26.14	23
CYB5R2	51700	Leukemia, MESH:D015473	Arsenic Tr	26.14	265
FGFR3	2261	Leukemia, MESH:D015473	Antimony	26.14	161
HSPA6	3310	Leukemia, MESH:D015470	Arsenic Tr	26.14	93
IGF2R	3482	Leukemia, MESH:D015470	Arsenic Tr	26.14	25
LDHB	3945	Leukemia, MESH:D015470	Arsenic Tr	26.14	26
PLG	5340	Leukemia, MESH:D015470	Arsenic Tr	26.14	104
PRSS23	11098	Leukemia, MESH:D015470	Calcitriol|[	26.14	19
ESD	2098	Leukemia, MESH:D015470	Arsenic Tr	26.13	25
NID1	4811	Leukemia, MESH:D015470	Cyclophos	26.13	20
AKAP10	11216	Leukemia, MESH:D015470	Dexameth	26.12	18
ATP6V0E1	8992	Leukemia, MESH:D015470	Bortezomi	26.12	19
GPR157	80045	Leukemia, MESH:D015470	Bortezomi	26.12	16
MCMBP	79892	Leukemia, MESH:D015470	Air Polluta	26.12	13
MSI1	4440	Leukemia, MESH:D015470	Arsenic Tr	26.12	28
RC3H1	149041	Leukemia, MESH:D015470	Cyclophos	26.12	16
TMBIM4	51643	Leukemia, MESH:D015470	Arsenic Tr	26.12	28
ZC3H7B	23264	Leukemia, MESH:D015470	Arsenic Tr	26.12	21
BTN3A3	10384	Leukemia, MESH:D015470	Arsenic Tr	26.11	19
PRIMPOL	201973	Leukemia, MESH:D015470	Calcitriol|[	26.11	9
MELK	9833	Leukemia, MESH:D015473	arsenite|C	26.1	157
CSF1R	1436	Leukemia, MESH:D015473	Calcitriol|[	26.09	156
CTSK	1513	Leukemia, MESH:D015473	Arsenic|ca	26.09	159
G6PD	2539	Leukemia, MESH:D015473	Antimony	26.09	267
MGP	4256	Leukemia, MESH:D015473	Arsenic Tr	26.09	265
UCHL1	7345	Leukemia, MESH:D015473	Arsenic|Ar	26.09	268
ALDH2	217	Leukemia, MESH:D015470	Benzene|C	26.07	51
CS	1431	Leukemia, MESH:D015470	Chloroqui	26.07	28
CXCL1	2919	Leukemia, MESH:D015473	alpha-Toc	26.07	163
DAB2	1601	Leukemia, MESH:D015470	Arsenic Tr	26.07	93
GRN	2896	Leukemia, MESH:D015470	Arsenic Tr	26.07	26
MAP1LC3I	81631	Leukemia, MESH:D015473	Arsenic|Ar	26.06	268
LPAR1	1902	Leukemia, MESH:D01 marker/m	Calcitriol|[	26.04	26
B3GNT5	84002	Leukemia, MESH:D015470	Dexameth	26.04	20
CLSTN1	22883	Leukemia, MESH:D015470	Arsenic Tr	26.04	18
CYP27B1	1594	Leukemia, MESH:D015470	Calcitriol|[	26.04	25
ECH1	1891	Leukemia, MESH:D015473	Arsenic Tr	26.04	261
FGF19	9965	Leukemia, MESH:D015473	Arsenic|Ch	26.04	154
GARS1	2617	Leukemia, MESH:D015470	Arsenic Tr	26.04	17
HDAC5	10014	Leukemia, MESH:D015470	Decitabine	26.04	25
HPSE	10855	Leukemia, MESH:D015470	Air Polluta	26.04	25
ITGB6	3694	Leukemia, MESH:D015470	Bortezomi	26.04	17
MBD1	4152	Leukemia, MESH:D015470	Bortezomi	26.04	22
MDN1	23195	Leukemia, MESH:D015470	Dexameth	26.04	19
MICAL1	64780	Leukemia, MESH:D015470	Calcitriol|[	26.04	11
MYD88	4615	Leukemia, MESH:D015473	Arsenic|Ar	26.04	270
STAT2	6773	Leukemia, MESH:D015470	Air Polluta	26.04	17
SYNE1	23345	Leukemia, MESH:D015470	Dexameth	26.04	15
TCOF1	6949	Leukemia, MESH:D015470	Calcitriol|[	26.04	21
TIGAR	57103	Leukemia, MESH:D015470	Arsenic Tr	26.04	27
CASQ2	845	Leukemia, MESH:D015473	Arsenic|ars	26.03	158
CHI3L1	1116	Leukemia, MESH:D015473	Arsenic Tr	26.03	262
EGFL6	25975	Leukemia, MESH:D015473	Arsenic Tr	26.03	264
ESYT1	23344	Leukemia, MESH:D015473	Arsenic Tr	26.03	262
NCOA6	23054	Leukemia, MESH:D015473	arsenite|D	26.03	159
PKNOX2	63876	Leukemia, MESH:D015473	Arsenic|Ar	26.03	262
RIPK2	8767	Leukemia, MESH:D015473	Calcitriol|C	26.03	153
SPON1	10418	Leukemia, MESH:D015473	Arsenic Tr	26.03	266

SULF1	23213	Leukemia,	MESH:D015473	Arsenic|Ar	26.03	264
DUSP1	1843	Leukemia,	MESH:D015473	Antimony	26.01	162
GPAM	57678	Leukemia,	MESH:D015470	Air Polluta	26	23
RORC	6097	Leukemia,	MESH:D015470	Bortezomi	26	27
ZFAND2A	90637	Leukemia,	MESH:D015470	Bortezomi	26	21
AHR	196	Leukemia,	MESH:D015473	2-(2-chlor	25.99	267
CDC6	990	Leukemia,	MESH:D015473	Arsenic Tr	25.99	265
CYP24A1	1591	Leukemia,	MESH:D015473	Arsenic Tr	25.99	267
FGFR2	2263	Leukemia,	MESH:D015470	Arsenic Tr	25.99	46
FN1	2335	Leukemia,	MESH:D015473	alpha-Toc	25.99	267
HSPA5	3309	Leukemia,	MESH:D015473	Arsenic|ars	25.99	269
TGFBI	7045	Leukemia,	MESH:D015473	Arsenic|ars	25.99	161
SREBF1	6720	Leukemia,	MESH:D015473	Arsenic|Ar	25.97	266
CTNNA1	1495	Leukemia,	MESH:D01 marker/m	Arsenic Tr	25.95	33
ACTR1A	10121	Leukemia,	MESH:D015470	Dexameth	25.95	17
ADAT2	134637	Leukemia,	MESH:D015470	Air Polluta	25.95	13
AEN	64782	Leukemia,	MESH:D015470	Arsenic Tr	25.95	22
BRIX1	55299	Leukemia,	MESH:D015470	Calcitriol|D	25.95	10
DENND2A	27147	Leukemia,	MESH:D015470	Dexameth	25.95	14
DSC3	1825	Leukemia,	MESH:D015470	Calcitriol|C	25.95	87
IKZF3	22806	Leukemia,	MESH:D015470	Decitabine	25.95	16
INAVA	55765	Leukemia,	MESH:D015470	Calcitriol|D	25.95	16
IQCK	124152	Leukemia,	MESH:D015470	Dronabinc	25.95	12
LOXL2	4017	Leukemia,	MESH:D015470	Decitabine	25.95	25
MTSS1	9788	Leukemia,	MESH:D015470	Calcitriol|D	25.95	24
NOL4L	140688	Leukemia,	MESH:D015470	Calcitriol|D	25.95	18
PATZ1	23598	Leukemia,	MESH:D015470	Bortezomi	25.95	17
PDZD8	118987	Leukemia,	MESH:D015470	Dexameth	25.95	15
PLCG2	5336	Leukemia,	MESH:D015470	Arsenic Tr	25.95	29
PNMA2	10687	Leukemia,	MESH:D015470	Decitabine	25.95	23
PRR16	51334	Leukemia,	MESH:D015470	Arsenic Tr	25.95	20
RPS6KA1	6195	Leukemia,	MESH:D015470	15-deoxy-	25.95	18
TIMP1	7076	Leukemia,	MESH:D007948	2-(2-amin	25.95	4
ZYG11B	79699	Leukemia,	MESH:D015470	Dexameth	25.95	15
NUMA1	4926	Leukemia,	MESH:D015470	Arsenic Tr	25.93	26
RELN	5649	Leukemia,	MESH:D015470	Bezafibrat	25.93	87
ABCA3	21	Leukemia,	MESH:D015470	Arsenic Tr	25.92	25
ACIN1	22985	Leukemia,	MESH:D015470	Air Polluta	25.92	18
C1QTNF6	114904	Leukemia,	MESH:D015470	Air Polluta	25.92	21
EPB41L2	2037	Leukemia,	MESH:D015470	Arsenic Tr	25.92	31
INPP1	3628	Leukemia,	MESH:D015470	Arsenic Tr	25.92	39
MAMDC2	256691	Leukemia,	MESH:D015470	Arsenic Tr	25.92	23
RNF144A	9781	Leukemia,	MESH:D015473	Arsenic|Ar	25.92	164
RNF213	57674	Leukemia,	MESH:D015470	Doxorubic	25.92	19
SOX6	55553	Leukemia,	MESH:D015470	Dexameth	25.92	17
STK38L	23012	Leukemia,	MESH:D015473	Arsenic|Ar	25.92	266
TYK2	7297	Leukemia,	MESH:D015470	Arsenic Tr	25.92	25
USP36	57602	Leukemia,	MESH:D015470	Air Polluta	25.92	13
XRCC4	7518	Leukemia,	MESH:D015470	Arsenic Tr	25.92	30
RBL1	5933	Leukemia,	MESH:D015473	alvocidib|/	25.89	263
KNL1	57082	Leukemia,	MESH:D015473	arsenite|C	25.88	158
POLA1	5422	Leukemia,	MESH:D015470	Dasatinib|I	25.87	28
SRM	6723	Leukemia,	MESH:D015470	Calcitriol|C	25.87	21
EIF2S1	1965	Leukemia,	MESH:D015473	Arsenic|Ar	25.86	269
IL23A	51561	Leukemia,	MESH:D015470	Air Polluta	25.86	29
NRF1	4899	Leukemia,	MESH:D015470	Arsenic Tr	25.86	25
TGM5	9333	Leukemia,	MESH:D015473	Arsenic|Ge	25.86	161
RSPH3	83861	Leukemia,	MESH:D015470	Doxorubic	25.85	15

FSHB	2488	Leukemia, MESH:D015470		2-(2-chlor	25.84	27
GPNMB	10457	Leukemia, MESH:D015473		Arsenic|Ar	25.84	264
TNFRSF10	8797	Leukemia, MESH:D015473		alvocidib|A	25.84	267
FBLN5	10516	Leukemia, MESH:D015473		Arsenic Tr	25.82	261
BUB3	9184	Leukemia, MESH:D015473		arsenite|D	25.81	158
CARD10	29775	Leukemia, MESH:D015470		Calcitriol|D	25.81	19
COL4A6	1288	Leukemia, MESH:D015470		Decitabine	25.81	22
DNMBP	23268	Leukemia, MESH:D015473		Arsenic|Ar	25.81	162
FAM210B	116151	Leukemia, MESH:D015470		Dexameth	25.81	11
RECQL4	9401	Leukemia, MESH:D015470		Bortezomi	25.81	17
SLC25A19	60386	Leukemia, MESH:D015473		Arsenic|Ca	25.81	159
SLC7A7	9056	Leukemia, MESH:D015473		Arsenic|Ca	25.81	162
ST3GAL5	8869	Leukemia, MESH:D015473		Arsenic|ars	25.81	160
TENM2	57451	Leukemia, MESH:D015470		Arsenic Tr	25.81	25
TRIM63	84676	Leukemia, MESH:D015470		Arsenic Tr	25.81	24
ALDOC	230	Leukemia, MESH:D015473		Antimony	25.79	262
ANKRD6	22881	Leukemia, MESH:D015470		Arsenic Tr	25.79	17
ARMC8	25852	Leukemia, MESH:D015470		Arsenic Tr	25.79	24
ATP5A1	11946	Leukemia, MESH:D015470		Arsenic Tr	25.79	30
ERLIN1	10613	Leukemia, MESH:D015470		Air Polluta	25.79	16
GLS	2744	Leukemia, MESH:D015473		Arsenic|Ar	25.79	266
GPATCH2	55105	Leukemia, MESH:D015470		Air Polluta	25.79	17
HSPB8	26353	Leukemia, MESH:D015473		Antimony	25.79	265
LIX1L	128077	Leukemia, MESH:D015470		Arsenic Tr	25.79	28
MTREX	23517	Leukemia, MESH:D015470		Arsenic Tr	25.79	23
SH2D3C	10044	Leukemia, MESH:D015470		Air Polluta	25.79	23
STYX	6815	Leukemia, MESH:D015470		Arsenic Tr	25.79	17
ZNF827	152485	Leukemia, MESH:D015470		Decitabine	25.79	24
ATAD2	29028	Leukemia, MESH:D015470		Arsenic Tr	25.78	26
NAP1L1	4673	Leukemia, MESH:D015470		Arsenic Tr	25.78	30
TSKU	25987	Leukemia, MESH:D015470		Arsenic Tr	25.78	20
HMGCS2	3158	Leukemia, MESH:D015470		Bezafibrat	25.77	90
ITGB3	3690	Leukemia, MESH:D007948		2-(2-amin	25.75	3
RYR2	6262	Leukemia, MESH:D015473		Arsenic|Ar	25.75	173
TNFAIP8	25816	Leukemia, MESH:D015473		Arsenic|ars	25.75	263
ASPH	444	Leukemia, MESH:D015473		Arsenic|Ar	25.74	265
MAP2K6	5608	Leukemia, MESH:D015473		Arsenic|Ar	25.74	266
NFKBIB	4793	Leukemia, MESH:D015470		Arsenic Tr	25.73	39
PLCB1	23236	Leukemia, MESH:D015470		Arsenic Tr	25.73	24
ANXA5	308	Leukemia, MESH:D01	marker/m	Alitretinoi	25.71	28
C1QC	714	Leukemia, MESH:D015470		Calcitriol|D	25.7	19
CBR1	873	Leukemia, MESH:D015473		Anthracyc	25.7	168
CCT4	10575	Leukemia, MESH:D015470		Air Polluta	25.7	17
DDX60	55601	Leukemia, MESH:D015470		Air Polluta	25.7	19
DNAJC2	27000	Leukemia, MESH:D015470		Arsenic Tr	25.7	17
ELK3	2004	Leukemia, MESH:D015470		Dexameth	25.7	19
EPHB4	2050	Leukemia, MESH:D015473		Arsenic|Da	25.7	155
FZD5	7855	Leukemia, MESH:D015470		Arsenic Tr	25.7	29
GPT2	84706	Leukemia, MESH:D015470		Arsenic Tr	25.7	24
LZTFL1	54585	Leukemia, MESH:D015473		Arsenic|Ar	25.7	268
MDK	4192	Leukemia, MESH:D015470		Alitretinoi	25.7	84
NRP1	8829	Leukemia, MESH:D015473		Arsenic|ars	25.7	162
PGAM2	5224	Leukemia, MESH:D015470		Alitretinoi	25.7	30
RERE	473	Leukemia, MESH:D015470		Arsenic Tr	25.7	20
RPF2	84154	Leukemia, MESH:D015470		Arsenic Tr	25.7	18
RPL21	6144	Leukemia, MESH:D015470		Arsenic Tr	25.7	32
SHCBP1	79801	Leukemia, MESH:D015470		Calcitriol|D	25.7	16
SMAD1	4086	Leukemia, MESH:D015470		Arsenic Tr	25.7	26

SMC1A	8243	Leukemia, MESH:D015470	Bortezomi	25.7	26
SOX17	64321	Leukemia, MESH:D015470	Dasatinib|I	25.7	24
CD40LG	959	Leukemia, MESH:D015473	Arsenic|Ar	25.68	265
NAP1L1	4673	Leukemia, MESH:D015473	Arsenic Tr	25.68	267
TNFRSF17	608	Leukemia, MESH:D015473	Arsenic|Ar	25.68	162
CASP10	843	Leukemia, MESH:D015473	Arsenic|Ar	25.67	264
GADD45B	4616	Leukemia, MESH:D015473	Arsenic|Ar	25.67	266
DHCR7	1717	Leukemia, MESH:D015470	Benzene|B	25.66	31
TCF4	6925	Leukemia, MESH:D015470	Arsenic Tr	25.66	27
AQP1	358	Leukemia, MESH:D015473	Arsenic|Ar	25.65	171
VLDLR	7436	Leukemia, MESH:D015473	Arsenic Tr	25.65	262
ARPC5	10092	Leukemia, MESH:D015470	Dexameth	25.63	12
CARS2	79587	Leukemia, MESH:D015470	Dexameth	25.63	16
DENND4C	55667	Leukemia, MESH:D015470	Bortezomi	25.63	11
DPH2	1802	Leukemia, MESH:D015470	Arsenic Tr	25.63	15
ERH	2079	Leukemia, MESH:D015470	Arsenic Tr	25.63	22
HAVCR2	84868	Leukemia, MESH:D015470	Air Polluta	25.63	19
MCHR1	2847	Leukemia, MESH:D015470	Doxorubic	25.63	16
MS4A6A	64231	Leukemia, MESH:D015470	Arsenic Tr	25.63	19
MTURN	222166	Leukemia, MESH:D015470	Air Polluta	25.63	15
MYPN	84665	Leukemia, MESH:D015470	Dasatinib|I	25.63	14
RBM34	23029	Leukemia, MESH:D015470	Cytarabine	25.63	88
RNF150	57484	Leukemia, MESH:D015470	Dexameth	25.63	12
SYTL1	84958	Leukemia, MESH:D015470	Arsenic Tr	25.63	28
TMEM254	80195	Leukemia, MESH:D015470	Air Polluta	25.63	11
EIF4A1	1973	Leukemia, MESH:D015473	Arsenic Tr	25.61	263
FTMT	94033	Leukemia, MESH:D015470	Arsenic Tr	25.61	20
GRB2	2885	Leukemia, MESH:D015470	Arsenic Tr	25.61	20
IFIH1	64135	Leukemia, MESH:D015473	Arsenic Tr	25.61	263
IL7	3574	Leukemia, MESH:D015470	Dexameth	25.61	19
PTPRD	5789	Leukemia, MESH:D015470	Calcitriol|D	25.61	17
PYGM	5837	Leukemia, MESH:D015470	alvocidib|D	25.61	52
RAB31	11031	Leukemia, MESH:D015470	Arsenic Tr	25.61	20
RSRP1	57035	Leukemia, MESH:D015470	Arsenic Tr	25.61	90
SHCBP1	79801	Leukemia, MESH:D015473	arsenite|Ca	25.61	158
SLC9A1	6548	Leukemia, MESH:D015470	Arsenic Tr	25.61	24
ALKBH4	54784	Leukemia, MESH:D015470	Arsenic Tr	25.6	20
BAG3	9531	Leukemia, MESH:D015473	Arsenic|Ar	25.6	269
COL18A1	80781	Leukemia, MESH:D015470	Cytarabine	25.6	87
KCNH2	3757	Leukemia, MESH:D015473	alpha-Toc	25.6	271
PDE4B	5142	Leukemia, MESH:D015473	Arsenic|Ar	25.6	268
SOX2	6657	Leukemia, MESH:D015473	Arsenic|Ar	25.6	266
TMEM179	374395	Leukemia, MESH:D015470	Dexameth	25.6	14
TPM3	7170	Leukemia, MESH:D015470	Arsenic Tr	25.6	30
CLSPN	63967	Leukemia, MESH:D015470	Arsenic Tr	25.59	26
DNAJB2	3300	Leukemia, MESH:D015470	Arsenic Tr	25.59	37
MTFR2	113115	Leukemia, MESH:D015473	arsenite|Ca	25.59	158
NME4	4833	Leukemia, MESH:D015470	Doxorubic	25.59	18
PRR11	55771	Leukemia, MESH:D015470	Calcitriol|D	25.59	18
TMEM106	79022	Leukemia, MESH:D015473	Arsenic|ars	25.59	15
ARL6IP5	10550	Leukemia, MESH:D015470	Arsenic Tr	25.58	96
CRTAP	10491	Leukemia, MESH:D015470	Cytarabine	25.58	85
CTHRC1	115908	Leukemia, MESH:D015470	Calcitriol|D	25.58	23
DTX4	23220	Leukemia, MESH:D015470	Calcitriol|D	25.58	17
ERAP1	51752	Leukemia, MESH:D015470	Cyclophos	25.58	16
GJA4	2701	Leukemia, MESH:D015470	Arsenic Tr	25.58	25
HSPA12A	259217	Leukemia, MESH:D015470	Decitabine	25.58	22
RTN4RL1	146760	Leukemia, MESH:D015470	Dexameth	25.58	19

TUBG1	7283	Leukemia, MESH:D015470		Arsenic Tr	25.58	32
TYRO3	7301	Leukemia, MESH:D015470		Dexameth	25.58	18
MYH7	4625	Leukemia, MESH:D015473		Calcitriol|D	25.55	23
VCAM1	7412	Leukemia, MESH:D007948		2-(2-amin	25.55	4
ELF3	1999	Leukemia, MESH:D015473		Antimony	25.54	264
NASP	4678	Leukemia, MESH:D015473		Arsenic Tr	25.54	263
ALDH3A2	224	Leukemia, MESH:D015470		Arsenic Tr	25.53	26
CAPN1	823	Leukemia, MESH:D015470		Alitretinoi	25.53	84
FBP1	2203	Leukemia, MESH:D015470		Arsenic Tr	25.53	27
LRP1	4035	Leukemia, MESH:D015470		Arsenic Tr	25.53	25
LTA4H	4048	Leukemia, MESH:D015470		Air Polluta	25.53	28
MLH1	4292	Leukemia, MESH:D015470		Air Polluta	25.53	23
PPP1R3B	79660	Leukemia, MESH:D015470		Calcitriol|D	25.53	14
PSMA5	5686	Leukemia, MESH:D015470		Air Polluta	25.53	24
SLC4A4	8671	Leukemia, MESH:D015470		Dexameth	25.53	18
EDN1	1906	Leukemia, MESH:D007948		2-(2-amin	25.52	4
FST	10468	Leukemia, MESH:D015473		Arsenic Tr	25.51	264
JAK2	3717	Leukemia, MESH:D015473		Arsenic|Ar	25.5	269
SP4	6671	Leukemia, MESH:D015473		Arsenic Tr	25.49	265
AFDN	4301	Leukemia, MESH:D015473		Arsenic|Ar	25.48	266
CTSB	1508	Leukemia, MESH:D015470		Arsenic Tr	25.48	27
TOPBP1	11073	Leukemia, MESH:D015470		Calcitriol|D	25.48	35
ASXL2	55252	Leukemia, MESH:D01	marker/m	Air Polluta	25.47	18
ABCA12	26154	Leukemia, MESH:D015470		Calcitriol|D	25.47	13
ALS2CL	259173	Leukemia, MESH:D015470		Calcitriol|D	25.47	14
AP1S2	8905	Leukemia, MESH:D015470		Decitabine	25.47	20
CHD7	55636	Leukemia, MESH:D015470		Air Polluta	25.47	23
COL9A1	1297	Leukemia, MESH:D015470		Cytarabine	25.47	82
COLGALT2	23127	Leukemia, MESH:D015470		Dexameth	25.47	11
DRAP1	10589	Leukemia, MESH:D015470		Dexameth	25.47	12
EYA1	2138	Leukemia, MESH:D015470		Azacitidine	25.47	17
GPATCH4	54865	Leukemia, MESH:D015470		Dexameth	25.47	11
HBP1	26959	Leukemia, MESH:D015473		Arsenic Tr	25.47	263
MKRN1	23608	Leukemia, MESH:D015470		Dexameth	25.47	16
MUC13	56667	Leukemia, MESH:D015470		Cyclophos	25.47	17
NBPF1	55672	Leukemia, MESH:D015473		Arsenic|ars	25.47	157
ODF3B	440836	Leukemia, MESH:D015470		Air Polluta	25.47	15
PCBP3	54039	Leukemia, MESH:D015470		Dexameth	25.47	15
PITRM1	10531	Leukemia, MESH:D015470		Decitabine	25.47	23
PKIG	11142	Leukemia, MESH:D015470		Benzene|D	25.47	39
POLR1E	64425	Leukemia, MESH:D015470		Dexameth	25.47	17
REST	5978	Leukemia, MESH:D015470		Alitretinoi	25.47	22
RET	5979	Leukemia, MESH:D015470		Azacitidine	25.47	18
RFNG	5986	Leukemia, MESH:D015470		Dexameth	25.47	14
RUVBL1	8607	Leukemia, MESH:D015470		Arsenic Tr	25.47	28
SEMA7A	8482	Leukemia, MESH:D015470		Arsenic Tr	25.47	24
SUFU	51684	Leukemia, MESH:D015470		Doxorubic	25.47	14
TMEM54	113452	Leukemia, MESH:D015470		Decitabine	25.47	23
TSPAN4	7106	Leukemia, MESH:D015470		Arsenic Tr	25.47	88
CYB5A	1528	Leukemia, MESH:D015473		Arsenic|ars	25.46	25
ARRB1	408	Leukemia, MESH:D015470		Bortezomi	25.45	25
COL6A2	1292	Leukemia, MESH:D015470		Cytarabine	25.45	86
PTGER2	5732	Leukemia, MESH:D015470		Arsenic Tr	25.45	31
SLC22A4	6583	Leukemia, MESH:D015470		Allopurinc	25.45	24
TNFRSF25	8718	Leukemia, MESH:D015470		Arsenic Tr	25.45	32
ACTB	60	Leukemia, MESH:D015470		Arsenic Tr	25.43	42
IRAK2	3656	Leukemia, MESH:D015473		arsenite|Ca	25.41	162
ITGA3	3675	Leukemia, MESH:D015473		arsenite|Ca	25.41	160

RPS6KA3	6197	Leukemia, MESH:D015473	Arsenic Tr	25.41	267
ACHE	43	Leukemia, MESH:D015470	Arsenic Tr	25.4	61
EYA4	2070	Leukemia, MESH:D015473	Arsenic|Ar	25.38	265
GPC6	10082	Leukemia, MESH:D015473	Arsenic|Ar	25.38	265
PAK3	5063	Leukemia, MESH:D015473	Arsenic Tr	25.38	263
PDCD5	9141	Leukemia, MESH:D015473	Arsenic Tr	25.38	264
WDR76	79968	Leukemia, MESH:D015473	Arsenic Tr	25.38	262
ADIPOR2	79602	Leukemia, MESH:D015470	Allopurinc	25.37	18
CARS1	833	Leukemia, MESH:D015470	Arsenic Tr	25.37	19
COX2	4513	Leukemia, MESH:D015470	Chlorampl	25.37	22
DPT	1805	Leukemia, MESH:D015470	Air Polluta	25.37	16
EFNB2	1948	Leukemia, MESH:D015470	Calcitriol|D	25.37	21
GTPBP4	23560	Leukemia, MESH:D015470	Dronabinc	25.37	13
HJURP	55355	Leukemia, MESH:D015470	Calcitriol|D	25.37	17
MND1	84057	Leukemia, MESH:D015470	Arsenic Tr	25.37	19
NRXN3	9369	Leukemia, MESH:D015470	Arsenic Tr	25.37	21
PIK3C2B	5287	Leukemia, MESH:D015470	Dexameth	25.37	18
PPP1R1A	5502	Leukemia, MESH:D015470	Dexameth	25.37	12
PRKCI	5584	Leukemia, MESH:D015470	Dexameth	25.37	17
PTPN12	5782	Leukemia, MESH:D015470	Doxorubic	25.37	19
RHBDF1	64285	Leukemia, MESH:D015470	Dexameth	25.37	17
SEMA3C	10512	Leukemia, MESH:D015470	Calcitriol|D	25.37	13
SEPTIN4	5414	Leukemia, MESH:D015470	Arsenic Tr	25.37	27
SLC2A6	11182	Leukemia, MESH:D015470	Benzene|C	25.37	37
SLC6A8	6535	Leukemia, MESH:D015470	Arsenic Tr	25.37	28
SMC3	9126	Leukemia, MESH:D015470	Bortezomi	25.37	20
SPATA13	221178	Leukemia, MESH:D015470	Arsenic Tr	25.37	22
UBA7	7318	Leukemia, MESH:D015470	Dexameth	25.37	17
DPYSL3	1809	Leukemia, MESH:D015470	Arsenic Tr	25.36	24
KIAA0040	9674	Leukemia, MESH:D015470	Air Polluta	25.36	14
PCNX4	64430	Leukemia, MESH:D015470	Air Polluta	25.36	14
BCDIN3D	144233	Leukemia, MESH:D015470	Dexameth	25.35	14
NBPF10	1E+08	Leukemia, MESH:D015470	Calcitriol|D	25.35	14
ATF2	1386	Leukemia, MESH:D007948	2-(2-amir	25.34	3
CORO1A	11151	Leukemia, MESH:D015473	Arsenic|Ar	25.34	269
PRIM1	5557	Leukemia, MESH:D015473	Calcitriol|D	25.34	156
AQP1	358	Leukemia, MESH:D007948	2-(2-amir	25.33	4
CD99	4267	Leukemia, MESH:D015470	Dexameth	25.32	17
COL9A3	1299	Leukemia, MESH:D015470	Azacitidin	25.32	19
EIF3M	10480	Leukemia, MESH:D015470	Arsenic Tr	25.32	18
FERMT3	83706	Leukemia, MESH:D015470	Arsenic Tr	25.32	16
H2BC9	8345	Leukemia, MESH:D015470	Arsenic Tr	25.32	23
HOMER3	9454	Leukemia, MESH:D015470	Arsenic Tr	25.32	21
HYLS1	219844	Leukemia, MESH:D015470	Cyclophos	25.32	15
KDM2A	22992	Leukemia, MESH:D015470	Arsenic Tr	25.32	35
MARCHF7	64844	Leukemia, MESH:D015470	Cyclophos	25.32	13
RAD52	5893	Leukemia, MESH:D015470	Arsenic Tr	25.32	27
SPATA18	132671	Leukemia, MESH:D015470	Decitabine	25.32	30
SPIN4	139886	Leukemia, MESH:D015470	Calcitriol|C	25.32	85
UBR3	130507	Leukemia, MESH:D015470	Bortezomi	25.32	16
ZNF503	84858	Leukemia, MESH:D015470	Dexameth	25.32	19
KCNH2	3757	Leukemia, MESH:D015470	Air Polluta	25.3	81
SNCA	6622	Leukemia, MESH:D015470	Arsenic Tr	25.3	97
GUCY1A1	2982	Leukemia, MESH:D015470	Calcitriol|C	25.28	84
ISYNA1	51477	Leukemia, MESH:D015470	Decitabine	25.28	25
LIMA1	51474	Leukemia, MESH:D015470	Bortezomi	25.28	22
LY6D	8581	Leukemia, MESH:D015473	Arsenic|De	25.28	158
PRPF8	10594	Leukemia, MESH:D015473	Arsenic|Ar	25.28	264

PTPRR	5801	Leukemia, MESH:D015473		arsenite|D	25.28	158
TPD52L1	7164	Leukemia, MESH:D015473		Arsenic Tr	25.28	263
UBE2T	29089	Leukemia, MESH:D015470		Calcitriol|D	25.28	18
AQP3	360	Leukemia, MESH:D015473		Arsenic|Ar	25.27	266
FBN1	2200	Leukemia, MESH:D015470		Dexameth	25.27	20
GCH1	2643	Leukemia, MESH:D015473		Arsenic|ars	25.27	162
MCM3	4172	Leukemia, MESH:D015470		Air Polluta	25.27	16
MYLK	4638	Leukemia, MESH:D015473		Arsenic Tr	25.27	262
PPP1R3C	5507	Leukemia, MESH:D015470		Dasatinib|I	25.27	23
BIN1	274	Leukemia, MESH:D015470		Bortezomi	25.26	25
CAPNS1	826	Leukemia, MESH:D015470		Arsenic Tr	25.26	23
FUCA1	2517	Leukemia, MESH:D015470		Air Polluta	25.26	29
GASK1B	51313	Leukemia, MESH:D015470		Air Polluta	25.26	16
GNG2	54331	Leukemia, MESH:D015470		Dexameth	25.26	20
PEA15	8682	Leukemia, MESH:D015470		Dexameth	25.26	20
PPM1A	5494	Leukemia, MESH:D015470		Arsenic Tr	25.26	25
RBMS1	5937	Leukemia, MESH:D015470		Benzene|D	25.26	33
ROR1	4919	Leukemia, MESH:D015470		Calcitriol|D	25.26	16
VASN	114990	Leukemia, MESH:D015470		Cytarabine	25.26	82
DNMT1	1786	Leukemia, MESH:D015473		Arsenic|ars	25.25	266
CXCL9	4283	Leukemia, MESH:D015473		Arsenic|Ar	25.23	269
THRB	7068	Leukemia, MESH:D015470		Arsenic Tr	25.22	28
LAMB3	3914	Leukemia, MESH:D015473		Arsenic Tr	25.21	157
XRCC5	7520	Leukemia, MESH:D015473		Arsenic|Ar	25.21	264
GPD2	2820	Leukemia, MESH:D015470		Arsenic Tr	25.2	29
MCM8	84515	Leukemia, MESH:D015473		Arsenic|Ca	25.18	158
NUP155	9631	Leukemia, MESH:D015473		arsenite|Ca	25.18	158
SLC39A11	201266	Leukemia, MESH:D015473		Arsenic|ars	25.18	158
ZBTB7A	51341	Leukemia, MESH:D01	marker/m	Dexameth	25.17	17
CASD1	64921	Leukemia, MESH:D015470		Dexameth	25.17	17
CYBB	1536	Leukemia, MESH:D015470		Arsenic Tr	25.17	52
D2HGDH	728294	Leukemia, MESH:D015470		Dexameth	25.17	14
FANCL	55120	Leukemia, MESH:D015470		Dexameth	25.17	16
ING5	84289	Leukemia, MESH:D015470		Arsenic Tr	25.17	29
METTL21A	151194	Leukemia, MESH:D015470		Bortezomi	25.17	11
NUDCD1	84955	Leukemia, MESH:D015470		Air Polluta	25.17	14
RIC8A	60626	Leukemia, MESH:D015470		Benzene|D	25.17	23
STX8	9482	Leukemia, MESH:D015470		Dexameth	25.17	11
VTCN1	79679	Leukemia, MESH:D015470		Calcitriol|D	25.17	12
ZKSCAN1	7586	Leukemia, MESH:D015470		Air Polluta	25.17	13
ZXDC	79364	Leukemia, MESH:D015470		Dexameth	25.17	11
MECOM	2122	Leukemia, MESH:D01	marker/m	Arsenic Tr	25.15	22
AHNAK2	113146	Leukemia, MESH:D015470		Decitabine	25.15	21
DSG2	1829	Leukemia, MESH:D015470		Arsenic Tr	25.15	19
FJX1	24147	Leukemia, MESH:D015470		Dexameth	25.15	12
FOXO4	4303	Leukemia, MESH:D015470		Arsenic Tr	25.15	29
GHR	2690	Leukemia, MESH:D015470		Bortezomi	25.15	86
GPR183	1880	Leukemia, MESH:D015470		Arsenic Tr	25.15	25
GPRC5B	51704	Leukemia, MESH:D015470		Calcitriol|D	25.15	22
LATS2	26524	Leukemia, MESH:D015470		Arsenic Tr	25.15	29
PKMYT1	9088	Leukemia, MESH:D015470		Arsenic Tr	25.15	17
UBE3A	7337	Leukemia, MESH:D015470		Arsenic Tr	25.15	23
WIF1	11197	Leukemia, MESH:D015470		Arsenic Tr	25.15	36
XRCC3	7517	Leukemia, MESH:D015470		Arsenic Tr	25.15	30
DCLK1	9201	Leukemia, MESH:D015470		Arsenic Tr	25.12	31
GFRA1	2674	Leukemia, MESH:D015470		Cyclophos	25.12	19
H19	283120	Leukemia, MESH:D015470		Decitabine	25.12	25
IFI44	10561	Leukemia, MESH:D015470		Bortezomi	25.12	23

PWWP2A	114825	Leukemia, MESH:D015470		Dexameth	25.12	15
TTI1	9675	Leukemia, MESH:D015470		Dexameth	25.12	14
RXRG	6258	Leukemia, MESH:D015473		Dexameth	25.09	159
CAMP	820	Leukemia, MESH:D015473		Arsenic|Ar	25.08	263
FABP3	2170	Leukemia, MESH:D015470		Bezafibrat	25.08	36
IER2	9592	Leukemia, MESH:D015473		Antimony	25.08	266
KMT2A	4297	Leukemia, MESH:D015473		Arsenic Tr	25.08	265
MAPK7	5598	Leukemia, MESH:D015473		Arsenic|Ar	25.08	162
CTSZ	1522	Leukemia, MESH:D01	marker/m	Benzoates	25.05	23
ACAD11	84129	Leukemia, MESH:D015470		Decitabine	25.05	17
ATP1B2	482	Leukemia, MESH:D015470		Decitabine	25.05	19
FLNB	2317	Leukemia, MESH:D015470		Arsenic Tr	25.05	28
FXN	2395	Leukemia, MESH:D015470		Dexameth	25.05	17
GPR137B	7107	Leukemia, MESH:D015470		Benzene|C	25.05	33
HADHB	3032	Leukemia, MESH:D015470		Bezafibrat	25.05	30
NEIL3	55247	Leukemia, MESH:D015470		Calcitriol|D	25.05	23
NUDT4	11163	Leukemia, MESH:D015470		Arsenic Tr	25.05	21
PDZD2	23037	Leukemia, MESH:D015470		Arsenic Tr	25.05	22
RAB20	55647	Leukemia, MESH:D015470		Dexameth	25.05	25
RPA3	6119	Leukemia, MESH:D015470		Arsenic Tr	25.05	23
SATB2	23314	Leukemia, MESH:D015470		Arsenic Tr	25.05	31
STRA6	64220	Leukemia, MESH:D015470		Arsenic Tr	25.05	16
TCF19	6941	Leukemia, MESH:D015470		Arsenic Tr	25.05	23
AMOTL1	154810	Leukemia, MESH:D015470		Dexameth	25.02	26
CCDC117	150275	Leukemia, MESH:D015470		Dexameth	25.02	15
CHFR	55743	Leukemia, MESH:D015470		Bortezomi	25.02	18
DDX11	1663	Leukemia, MESH:D015470		Bortezomi	25.02	17
EXOC7	23265	Leukemia, MESH:D015470		Dexameth	25.02	16
GIMAP4	55303	Leukemia, MESH:D015470		Air Polluta	25.02	15
GPR18	2841	Leukemia, MESH:D015470		Arsenic Tr	25.02	16
HIF1AN	55662	Leukemia, MESH:D015470		Bortezomi	25.02	12
KIFAP3	22920	Leukemia, MESH:D015470		Calcitriol|D	25.02	23
MET	4233	Leukemia, MESH:D015473		Arsenic Tr	25.02	263
METTL3	56339	Leukemia, MESH:D015470		Air Polluta	25.02	23
MNT	4335	Leukemia, MESH:D015470		Arsenic Tr	25.02	23
NCSTN	23385	Leukemia, MESH:D015470		Dexameth	25.02	10
RAB7A	7879	Leukemia, MESH:D015470		Air Polluta	25.02	29
ROPN1L	83853	Leukemia, MESH:D015470		Indometha	25.02	11
SYP	6855	Leukemia, MESH:D015470		2-(2-chlor	25.02	15
TMEM109	79073	Leukemia, MESH:D015470		Dexameth	25.02	15
TMEM38A	79041	Leukemia, MESH:D015470		Dexameth	25.02	13
ZCCHC14	23174	Leukemia, MESH:D015470		Benzene|D	25.02	30
EPHX1	2052	Leukemia, MESH:D015473		Antimony	25.01	264
MAOA	4128	Leukemia, MESH:D015473		Antimony	25.01	265
GATA1	2623	Leukemia, MESH:D015473		Aclarubici	24.99	264
PLPP2	8612	Leukemia, MESH:D015473		Arsenic Tr	24.98	262
PMAIP1	5366	Leukemia, MESH:D015473		Arsenic Tr	24.98	264
CA9	768	Leukemia, MESH:D015470		Arsenic Tr	24.97	23
CLDN3	1365	Leukemia, MESH:D015470		Air Polluta	24.97	18
CYP3A7	1551	Leukemia, MESH:D015470		2-(2-chlor	24.97	13
EIF2A	83939	Leukemia, MESH:D015470		2-(2-chlor	24.97	23
NTRK3	4916	Leukemia, MESH:D01	marker/m	Arsenic Tr	24.94	23
BCAT2	587	Leukemia, MESH:D015470		Arsenic Tr	24.94	23
CDV3	55573	Leukemia, MESH:D015470		Arsenic Tr	24.94	22
DYRK2	8445	Leukemia, MESH:D015470		Arsenic Tr	24.94	26
G2E3	55632	Leukemia, MESH:D015470		Calcitriol|D	24.94	17
IFI44L	10964	Leukemia, MESH:D015470		Benzene|D	24.94	35
KCNB1	3745	Leukemia, MESH:D015470		Dexameth	24.94	18

NUAK1	9891	Leukemia, MESH:D015470	Arsenic Tr	24.94	17
PPAT	5471	Leukemia, MESH:D015470	Arsenic Tr	24.94	20
APOE	348	Leukemia, MESH:D015470	Arsenic Tr	24.93	36
HSPH1	10808	Leukemia, MESH:D015470	Alitretinoi	24.93	24
NUPR1	26471	Leukemia, MESH:D015470	Arsenic Tr	24.93	33
CSF1	1435	Leukemia, MESH:D015473	Arsenic|Ca	24.92	159
S100A4	6275	Leukemia, MESH:D007948	2-(2-amin	24.92	4
DGAT2	84649	Leukemia, MESH:D015470	Benzene|C	24.9	29
PIR	8544	Leukemia, MESH:D015470	Alitretinoi	24.9	23
CAMK2D	817	Leukemia, MESH:D015473	Arsenic|Ar	24.89	262
DDX58	23586	Leukemia, MESH:D015470	15-deoxy-	24.89	20
LAMP1	3916	Leukemia, MESH:D015473	Arsenic Tr	24.89	263
MT1E	4493	Leukemia, MESH:D015470	Arsenic Tr	24.89	89
PCDHB14	56122	Leukemia, MESH:D015470	Azacitidine	24.89	15
SHFL	55337	Leukemia, MESH:D015470	Decitabine	24.89	20
SLC25A4	291	Leukemia, MESH:D015473	Arsenic|Ar	24.89	269
SLC2A4RG	56731	Leukemia, MESH:D015470	Dexameth	24.89	15
TWIST1	7291	Leukemia, MESH:D015473	Arsenic|ar	24.89	161
YWHAE	7531	Leukemia, MESH:D015470	Air Polluta	24.89	27
EDN2	1907	Leukemia, MESH:D015473	Arsenic|De	24.88	162
KDR	3791	Leukemia, MESH:D015473	Arsenic Tr	24.88	264
TNFSF13B	10673	Leukemia, MESH:D015473	Arsenic|Ar	24.88	265
TRIM6	117854	Leukemia, MESH:D015473	Arsenic|Ca	24.88	160
ALG14	199857	Leukemia, MESH:D015470	Dexameth	24.87	14
CD58	965	Leukemia, MESH:D015470	Doxorubic	24.87	28
CORO1B	57175	Leukemia, MESH:D015470	Alitretinoi	24.87	12
FOSL1	8061	Leukemia, MESH:D015470	Arsenic Tr	24.87	41
HERC1	8925	Leukemia, MESH:D015470	Arsenic Tr	24.87	24
JAZF1	221895	Leukemia, MESH:D015470	Dexameth	24.87	15
KLHL7	55975	Leukemia, MESH:D015470	Decitabine	24.87	22
MICAL3	57553	Leukemia, MESH:D015470	Calcitriol|D	24.87	19
PPIL3	53938	Leukemia, MESH:D015470	Arsenic Tr	24.87	20
RAG1	5896	Leukemia, MESH:D015470	Benzene|C	24.87	29
RNFT2	84900	Leukemia, MESH:D015470	Dronabinc	24.87	11
SGCD	6444	Leukemia, MESH:D015470	Arsenic Tr	24.87	20
SOX3	6658	Leukemia, MESH:D015470	Cytarabine	24.87	80
TDRD3	81550	Leukemia, MESH:D015470	Decitabine	24.87	32
VEZF1	7716	Leukemia, MESH:D015470	Cytarabine	24.87	85
IL10	3586	Leukemia, MESH:D015473	alpha-Toc	24.86	271
CYP11A1	1583	Leukemia, MESH:D015470	Alitretinoi	24.85	29
AOC3	8639	Leukemia, MESH:D015470	Arsenic Tr	24.84	24
ASAP1	50807	Leukemia, MESH:D015470	Bortezomi	24.84	19
ATIC	471	Leukemia, MESH:D015470	Arsenic Tr	24.84	23
CSNK2A2	1459	Leukemia, MESH:D015470	Decitabine	24.84	24
EHBP1	23301	Leukemia, MESH:D015470	Calcitriol|D	24.84	17
FAS	355	Leukemia, MESH:D007948	2-(2-amin	24.84	4
FKBP1B	2281	Leukemia, MESH:D015470	Calcitriol|C	24.84	17
GCK	2645	Leukemia, MESH:D015470	Alitretinoi	24.84	34
JAG1	182	Leukemia, MESH:D015470	Dasatinib|I	24.84	20
KALRN	8997	Leukemia, MESH:D015470	Arsenic Tr	24.84	24
KCNN2	3781	Leukemia, MESH:D015470	Arsenic Tr	24.84	91
MATR3	9782	Leukemia, MESH:D015470	Doxorubic	24.84	20
MBNL1	4154	Leukemia, MESH:D015470	Bortezomi	24.84	12
MCTP1	79772	Leukemia, MESH:D015470	Arsenic Tr	24.84	16
PDLIM3	27295	Leukemia, MESH:D015470	Cytarabine	24.84	90
SEL1L	6400	Leukemia, MESH:D015470	Benzene|D	24.84	39
SHISA2	387914	Leukemia, MESH:D015470	Arsenic Tr	24.84	27
SPARCL1	8404	Leukemia, MESH:D015470	Calcitriol|D	24.84	17

HIPK2	28996	Leukemia, MESH:D015473		Arsenic|ars	24.83	161
MAPKAPK	9261	Leukemia, MESH:D015473		Arsenic Tr	24.83	266
PCLAF	9768	Leukemia, MESH:D015473		arsenite|Ca	24.83	11
SFRP2	6423	Leukemia, MESH:D015473		Arsenic|Ar	24.83	268
CXCL10	3627	Leukemia, MESH:D015473		Arsenic Tr	24.82	266
ELOVL5	60481	Leukemia, MESH:D015470		Arsenic Tr	24.81	43
MYH9	4627	Leukemia, MESH:D015470		Arsenic Tr	24.81	24
NPR3	4883	Leukemia, MESH:D015470		Dexameth	24.81	18
A2M	2	Leukemia, MESH:D015473		Antimony	24.79	260
ANAPC1	64682	Leukemia, MESH:D015473		arsenite|D	24.79	157
FANCC	2176	Leukemia, MESH:D015473		Arsenic|Ar	24.79	265
MAP2K2	5605	Leukemia, MESH:D015473		2-(2-chlor	24.79	264
RNF4	6047	Leukemia, MESH:D015473		Arsenic|Ar	24.79	264
VAV1	7409	Leukemia, MESH:D015473		Arsenic|Ar	24.79	266
MCM2	4171	Leukemia, MESH:D015473		Antimony	24.78	262
PODN	127435	Leukemia, MESH:D015473		Arsenic|Ca	24.77	10
FOS	2353	Leukemia, MESH:D015473		alpha-Toc	24.76	269
BDH1	622	Leukemia, MESH:D015470		Arsenic Tr	24.74	24
CAVIN3	112464	Leukemia, MESH:D015470		Arsenic Tr	24.74	87
CPS1	1373	Leukemia, MESH:D015470		Dexameth	24.74	28
CYB5R3	1727	Leukemia, MESH:D015470		Arsenic Tr	24.74	21
FH	2271	Leukemia, MESH:D015470		Arsenic Tr	24.74	21
PSMB9	5698	Leukemia, MESH:D015470		Calcitriol|D	24.74	19
RAMP2	10266	Leukemia, MESH:D015470		Arsenic Tr	24.74	30
SP3	6670	Leukemia, MESH:D015470		Arsenic Tr	24.74	71
TP63	8626	Leukemia, MESH:D015470		Arsenic Tr	24.74	20
CDK10	8558	Leukemia, MESH:D015470		Doxorubic	24.73	18
FRYL	285527	Leukemia, MESH:D015470		Doxorubic	24.73	16
KAT2A	2648	Leukemia, MESH:D015470		Arsenic Tr	24.73	21
MTMR6	9107	Leukemia, MESH:D015470		Bortezomi	24.73	18
RHOF	54509	Leukemia, MESH:D015470		Arsenic Tr	24.73	35
SNAI1	6615	Leukemia, MESH:D015470		Arsenic Tr	24.73	33
UQCR10	29796	Leukemia, MESH:D015470		Dexameth	24.73	16
ZNF367	195828	Leukemia, MESH:D015470		Calcitriol|(	24.73	13
IDH1	3417	Leukemia, MESH:D01	marker/me	Air Polluta	24.72	40
HIF1A	3091	Leukemia, MESH:D007948		2-(2-amin	24.72	4
TIPARP	25976	Leukemia, MESH:D015470		Air Polluta	24.72	20
ALDH1B1	219	Leukemia, MESH:D015473		Arsenic Tr	24.71	264
BIK	638	Leukemia, MESH:D015473		Arsenic|Ar	24.71	262
FGF1	2246	Leukemia, MESH:D007948		2-(2-amin	24.71	3
LCK	3932	Leukemia, MESH:D015473		alpha-Toc	24.71	157
NR2F2	7026	Leukemia, MESH:D015473		Arsenic Tr	24.71	264
SLC39A8	64116	Leukemia, MESH:D015473		Arsenic|Ca	24.71	159
SMPD3	55512	Leukemia, MESH:D015473		Arsenic|Ar	24.71	267
CYP2A4	13086	Leukemia, MESH:D015473		Arsenic|ars	24.7	159
FOSB	2354	Leukemia, MESH:D015473		alpha-Toc	24.7	270
CTSD	1509	Leukemia, MESH:D015473		alpha-Toc	24.69	271
AKR1B10	57016	Leukemia, MESH:D015470		Arsenic Tr	24.68	68
BOD1L1	259282	Leukemia, MESH:D015470		Arsenic Tr	24.68	16
FAM118B	79607	Leukemia, MESH:D015470		Air Polluta	24.68	15
SIRPB1	10326	Leukemia, MESH:D015470		Azacitidine	24.68	21
SNAI2	6591	Leukemia, MESH:D007948		Arsenic Tr	24.68	4
TMEM50A	23585	Leukemia, MESH:D015470		Dexameth	24.67	14
TSTD2	158427	Leukemia, MESH:D015470		Dexameth	24.67	14
ACADVL	37	Leukemia, MESH:D015470		Bezafibrat	24.66	37
CYP26A1	1592	Leukemia, MESH:D015473		Arsenic|Ar	24.66	265
GRB10	2887	Leukemia, MESH:D015470		Arsenic Tr	24.66	26
LAMB1	3912	Leukemia, MESH:D015470		Chloroqui	24.66	26

PPP2CA	5515	Leukemia, MESH:D015470		Arsenic Tr	24.66	25
PSMB8	5696	Leukemia, MESH:D015470		Arsenic Tr	24.66	25
RETN	56729	Leukemia, MESH:D01	marker/m	Arsenic Tr	24.65	261
ATP2B1	490	Leukemia, MESH:D015473		Antimony	24.65	270
CACNA1C	775	Leukemia, MESH:D015473		Arsenic|Ar	24.65	264
CENPA	1058	Leukemia, MESH:D015473		arsenite|C	24.65	158
FANCD2	2177	Leukemia, MESH:D015473		Arsenic Tr	24.65	264
IGFBP7	3490	Leukemia, MESH:D015473		Arsenic|Ar	24.65	265
MT2A	4502	Leukemia, MESH:D015473		Antimony	24.65	171
ADARB1	104	Leukemia, MESH:D015470		Dexameth	24.64	22
AK2	204	Leukemia, MESH:D015470		Decitabine	24.64	23
ARHGAP2	23092	Leukemia, MESH:D015470		Arsenic Tr	24.64	22
ATP2A3	489	Leukemia, MESH:D015470		Arsenic Tr	24.64	21
CAMK1D	57118	Leukemia, MESH:D015470		Calcitriol|D	24.64	17
CCR6	1235	Leukemia, MESH:D015470		Dexameth	24.64	16
EPHA1	2041	Leukemia, MESH:D015470		Bortezomi	24.64	20
FLRT3	23767	Leukemia, MESH:D015470		Arsenic Tr	24.64	25
FOXN3	1112	Leukemia, MESH:D015470		Benzene|D	24.64	34
INA	9118	Leukemia, MESH:D015470		Cytarabine	24.64	81
MEIS2	4212	Leukemia, MESH:D015470		Alitretinoi	24.64	16
RASA1	5921	Leukemia, MESH:D015470		Dexameth	24.64	25
SERPINB8	5271	Leukemia, MESH:D015470		Alitretinoi	24.64	20
TSC1	7248	Leukemia, MESH:D015470		Azacitidine	24.64	25
STAR	6770	Leukemia, MESH:D015473		alpha-Toc	24.62	263
AHCYL1	10768	Leukemia, MESH:D015473		Arsenic|Ar	24.61	263
GCA	25801	Leukemia, MESH:D015473		arsenite|C	24.61	161
PKIA	5569	Leukemia, MESH:D015473		Arsenic|ar	24.61	161
SLA	6503	Leukemia, MESH:D015473		Arsenic Tr	24.61	267
SLC43A3	29015	Leukemia, MESH:D015473		Arsenic|ar	24.61	160
NRN1	51299	Leukemia, MESH:D015473		Arsenic|ar	24.6	159
PTPRN2	5799	Leukemia, MESH:D015473		Arsenic|Ar	24.6	265
SGO2	151246	Leukemia, MESH:D015473		Arsenic|Ca	24.6	158
TMTC1	83857	Leukemia, MESH:D015473		Arsenic|Ar	24.6	265
WT1	7490	Leukemia, MESH:D01	marker/m	Arsenic Tr	24.59	22
ACADL	33	Leukemia, MESH:D015470		Bezafibrat	24.59	19
ALDH1B1	219	Leukemia, MESH:D015470		Arsenic Tr	24.59	22
ANGPT1	284	Leukemia, MESH:D015473		Arsenic|Ar	24.59	267
AP3M2	10947	Leukemia, MESH:D015470		Air Polluta	24.59	29
AQP4	361	Leukemia, MESH:D015470		Arsenic Tr	24.59	22
ATE1	11101	Leukemia, MESH:D015470		Bortezomi	24.59	17
C15ORF48	84419	Leukemia, MESH:D015470		Air Polluta	24.59	18
CDCA8	55143	Leukemia, MESH:D015473		arsenite|C	24.59	160
COX6B2	125965	Leukemia, MESH:D015470		Arsenic Tr	24.59	24
E2F2	1870	Leukemia, MESH:D015473		Arsenic|Ar	24.59	265
FAM241A	132720	Leukemia, MESH:D015470		Dexameth	24.59	15
KAZALD1	81621	Leukemia, MESH:D015470		Dexameth	24.59	16
MAP2K4	6416	Leukemia, MESH:D015470		alvocidib|A	24.59	26
PAPSS2	9060	Leukemia, MESH:D015470		Arsenic Tr	24.59	45
PBRM1	55193	Leukemia, MESH:D015470		Doxorubic	24.59	18
PPIH	10465	Leukemia, MESH:D015470		Arsenic Tr	24.59	24
RBM4	5936	Leukemia, MESH:D015470		Decitabine	24.59	21
RRP15	51018	Leukemia, MESH:D015470		Doxorubic	24.59	15
SEC16A	9919	Leukemia, MESH:D015470		Benzene|D	24.59	29
SHOX2	6474	Leukemia, MESH:D015470		Calcitriol|D	24.59	19
SMAD4	4089	Leukemia, MESH:D015473		alpha-Toc	24.59	164
STRIP2	57464	Leukemia, MESH:D015470		Dexameth	24.59	18
SUGP2	10147	Leukemia, MESH:D015470		Decitabine	24.59	23
TFPI2	7980	Leukemia, MESH:D015473		arsenite|D	24.59	157

TPD52L2	7165	Leukemia, MESH:D015470	Arsenic Tr	24.59	29
ZMYND11	10771	Leukemia, MESH:D015470	Bortezomi	24.59	13
CENPW	387103	Leukemia, MESH:D015470	Calcitriol|[illegible]	24.54	16
CITED4	163732	Leukemia, MESH:D015470	Calcitriol|[illegible]	24.54	16
CXXC5	51523	Leukemia, MESH:D015470	Arsenic Tr	24.54	16
DAXX	1616	Leukemia, MESH:D015470	Arsenic Tr	24.54	23
ENTPD1	953	Leukemia, MESH:D015470	Air Polluta	24.54	82
FUT8	2530	Leukemia, MESH:D015470	Calcitriol|[illegible]	24.54	24
LRIG1	26018	Leukemia, MESH:D015470	Calcitriol|[illegible]	24.54	81
MYL1	4632	Leukemia, MESH:D015470	Dexameth	24.54	19
PLA1A	51365	Leukemia, MESH:D015470	Dexameth	24.54	16
SYTL2	54843	Leukemia, MESH:D015470	Cytarabine	24.54	81
ZFR	51663	Leukemia, MESH:D015470	Cyclophos	24.54	14
ASNS	440	Leukemia, MESH:D015473	Arsenic Tr	24.53	263
CD24	1E+08	Leukemia, MESH:D015473	Arsenic Tr	24.53	264
COL11A1	1301	Leukemia, MESH:D015473	arsenite|Ca	24.53	156
FHL1	2273	Leukemia, MESH:D015473	Arsenic Tr	24.53	262
IL7R	3575	Leukemia, MESH:D015473	Arsenic|ars	24.53	19
KRT5	3852	Leukemia, MESH:D015473	Arsenic|ars	24.53	161
NR1H3	10062	Leukemia, MESH:D015473	Arsenic|Ar	24.53	264
OGDH	4967	Leukemia, MESH:D015473	alpha-Toc	24.53	161
ACAN	176	Leukemia, MESH:D015473	caffeic aci	24.52	159
AMPD3	272	Leukemia, MESH:D015470	Calcitriol|[illegible]	24.51	27
CACNA1C	775	Leukemia, MESH:D015470	Arsenic Tr	24.51	25
CTTN	2017	Leukemia, MESH:D015470	Air Polluta	24.51	36
EEF1E1	9521	Leukemia, MESH:D015473	Arsenic|Ar	24.51	268
GLI1	2735	Leukemia, MESH:D015470	Arsenic Tr	24.51	29
MBD2	8932	Leukemia, MESH:D015473	Arsenic|Ar	24.51	264
MYBL1	4603	Leukemia, MESH:D015470	Arsenic Tr	24.51	22
MYH14	79784	Leukemia, MESH:D015473	Arsenic|ars	24.51	14
HSP90AB1	3326	Leukemia, MESH:D015470	Arsenic Tr	24.48	26
PODXL	5420	Leukemia, MESH:D015473	Arsenic|Ar	24.47	269
RNF113A	7737	Leukemia, MESH:D015470	Dronabinc	24.47	19
SHC1	6464	Leukemia, MESH:D015473	Arsenic|Ar	24.47	266
SPC25	57405	Leukemia, MESH:D015473	Arsenic|Ar	24.47	267
ANKRD36	57730	Leukemia, MESH:D015470	Dexameth	24.46	9
ATP5O	28080	Leukemia, MESH:D015470	Dexameth	24.46	22
BGLAP	632	Leukemia, MESH:D007948	2-(2-amin	24.46	3
C1ORF116	79098	Leukemia, MESH:D015470	Calcitriol|[illegible]	24.46	12
CWF19L1	55280	Leukemia, MESH:D015470	Arsenic Tr	24.46	20
GTF2E1	2960	Leukemia, MESH:D015470	Air Polluta	24.46	8
MALL	7851	Leukemia, MESH:D015470	Arsenic Tr	24.46	15
SDHAF1	644096	Leukemia, MESH:D015470	Dexameth	24.46	16
CDC73	79577	Leukemia, MESH:D015470	Arsenic Tr	24.45	23
CREBBP	1387	Leukemia, MESH:D015473	alpha-Toc	24.45	269
DENND1B	163486	Leukemia, MESH:D015470	Calcitriol|[illegible]	24.45	15
FAM171A1	221061	Leukemia, MESH:D015470	Dexameth	24.45	25
KRT6A	3853	Leukemia, MESH:D015470	Alitretinoi	24.45	23
MYOM1	8736	Leukemia, MESH:D015470	Arsenic Tr	24.45	20
NAIP	4671	Leukemia, MESH:D015470	Air Polluta	24.45	16
RHOH	399	Leukemia, MESH:D015470	Air Polluta	24.45	15
SCD1	20249	Leukemia, MESH:D015470	Arsenic Tr	24.45	28
SPTY2D1	144108	Leukemia, MESH:D015470	Bortezomi	24.45	15
TMEM107	84314	Leukemia, MESH:D015470	Arsenic Tr	24.45	36
GNAI3	2773	Leukemia, MESH:D015470	Arsenic Tr	24.44	22
IL13RA2	3598	Leukemia, MESH:D015470	Dexameth	24.44	22
NEFM	4741	Leukemia, MESH:D015470	Alitretinoi	24.44	27
PADI2	11240	Leukemia, MESH:D015470	Dexameth	24.44	11

PLXDC2	84898	Leukemia, MESH:D015470		Calcitriol|D	24.44	16
PLXNB2	23654	Leukemia, MESH:D015470		Air Polluta	24.44	23
PPBP	5473	Leukemia, MESH:D015470		Arsenic Tr	24.44	38
RASL11B	65997	Leukemia, MESH:D015470		Calcitriol|D	24.44	22
RHOQ	23433	Leukemia, MESH:D015470		Arsenic Tr	24.44	21
SLC16A6	9120	Leukemia, MESH:D015470		Dasatinib|I	24.44	36
SMAD4	4089	Leukemia, MESH:D015470		Dexameth	24.44	22
SPARC	6678	Leukemia, MESH:D015473		Arsenic|ars	24.44	164
TET2	54790	Leukemia, MESH:D015470		Arsenic Tr	24.44	21
CTSG	1511	Leukemia, MESH:D015473		Arsenic|Ar	24.43	261
ADRB1	153	Leukemia, MESH:D015470		Azacitidine	24.42	30
CYP2C9	1559	Leukemia, MESH:D015473		Cholesterc	24.42	156
GH1	2688	Leukemia, MESH:D015470		Alitretinoi	24.42	24
HLA-A	3105	Leukemia, MESH:D015473		Arsenic Tr	24.42	261
MME	4311	Leukemia, MESH:D015470		Doxorubic	24.42	20
POLD3	10714	Leukemia, MESH:D015473		Arsenic Tr	24.42	264
SLC2A3	6515	Leukemia, MESH:D015473		Antimony	24.4	264
ADAM4	11498	Leukemia, MESH:D015470		Cyclophos	24.36	11
BCAT1	586	Leukemia, MESH:D015473		arsenite|D	24.36	162
COL11A1	1301	Leukemia, MESH:D015470		Calcitriol|D	24.36	30
COX4I1	1327	Leukemia, MESH:D015473		Arsenic Tr	24.36	267
CTSH	1512	Leukemia, MESH:D015473		Arsenic Tr	24.36	266
GNAS	2778	Leukemia, MESH:D015470		Benzene|D	24.36	33
LTB	4050	Leukemia, MESH:D015470		Arsenic Tr	24.36	36
OLR1	4973	Leukemia, MESH:D015473		Arsenic Tr	24.36	265
RAD51	5888	Leukemia, MESH:D007948		2-(2-amin	24.36	3
B4GALT1	2683	Leukemia, MESH:D015470		Calcitriol|D	24.35	18
CDON	50937	Leukemia, MESH:D015470		Dexameth	24.35	16
FZD4	8322	Leukemia, MESH:D015470		Decitabine	24.35	19
GFPT2	9945	Leukemia, MESH:D015470		Calcitriol|D	24.35	18
LIFR	3977	Leukemia, MESH:D015473		Arsenic|ars	24.35	161
PEG10	23089	Leukemia, MESH:D015470		Decitabine	24.35	23
PSMB2	5690	Leukemia, MESH:D015470		Carboplati	24.35	19
PSMD7	5713	Leukemia, MESH:D015470		Arsenic Tr	24.35	21
PTPRO	5800	Leukemia, MESH:D015470		Arsenic Tr	24.35	20
TP53BP1	7158	Leukemia, MESH:D015470		Bortezomi	24.35	26
TRIB2	28951	Leukemia, MESH:D015470		Arsenic Tr	24.35	29
C5ORF22	55322	Leukemia, MESH:D015470		Doxorubic	24.34	21
CHRDL1	91851	Leukemia, MESH:D015473		Arsenic Tr	24.34	264
KEAP1	9817	Leukemia, MESH:D015473		Arsenic|Ar	24.34	267
PRKCZ	5590	Leukemia, MESH:D004915		Cytarabine	24.34	2
PRPH	5630	Leukemia, MESH:D015473		Arsenic|ars	24.34	159
C11ORF71	54494	Leukemia, MESH:D015470		Dexameth	24.33	13
CARD10	29775	Leukemia, MESH:D015473		Arsenic|Ca	24.33	157
CDCA7L	55536	Leukemia, MESH:D015473		Arsenic|Ca	24.33	159
CRP	1401	Leukemia, MESH:D015470		Arsenic Tr	24.33	36
VOPP1	81552	Leukemia, MESH:D01	marker/m	Benzene|D	24.32	29
ANKRD22	118932	Leukemia, MESH:D015470		Calcitriol|(	24.32	12
COMMD1	51397	Leukemia, MESH:D015470		Decitabine	24.32	18
DHX37	57647	Leukemia, MESH:D015470		Dexameth	24.32	14
FITM2	128486	Leukemia, MESH:D015470		Dexameth	24.32	16
HRG	3273	Leukemia, MESH:D015470		Arsenic Tr	24.32	23
MBTD1	54799	Leukemia, MESH:D015470		Calcitriol|C	24.32	84
MYO9A	4649	Leukemia, MESH:D015470		Dexameth	24.32	15
NFS1	9054	Leukemia, MESH:D015470		Dexameth	24.32	17
PLA2G4A	5321	Leukemia, MESH:D015473		Arsenic Tr	24.32	263
PPP1R18	170954	Leukemia, MESH:D015470		Decitabine	24.32	23
RSU1	6251	Leukemia, MESH:D015470		Arsenic Tr	24.32	15

SIRT4	23409	Leukemia, MESH:D015470		Decitabine	24.32	17
STRAP	11171	Leukemia, MESH:D015470		Air Polluta	24.32	34
UBL3	5412	Leukemia, MESH:D015470		Cytarabine	24.32	80
ROCK1	6093	Leukemia, MESH:D01	marker/m	Arsenic|Ar	24.3	269
PRKAA1	5562	Leukemia, MESH:D015470		Arsenic Tr	24.3	26
RAD50	10111	Leukemia, MESH:D015470		Arsenic Tr	24.29	37
SPC25	57405	Leukemia, MESH:D015470		Arsenic Tr	24.29	21
LIF	3976	Leukemia, MESH:D015473		Arsenic Tr	24.28	262
PDGFRB	5159	Leukemia, MESH:D007948		2-(2-amin	24.28	4
PHGDH	26227	Leukemia, MESH:D015473		Arsenic Tr	24.28	264
FLT3	2322	Leukemia, MESH:D01	marker/m	Arsenic Tr	24.26	263
LPP	4026	Leukemia, MESH:D01	marker/m	Calcitriol|D	24.25	19
ANKRD11	29123	Leukemia, MESH:D015470		Arsenic Tr	24.25	18
AP1S1	1174	Leukemia, MESH:D015470		Arsenic Tr	24.25	24
AQP1	358	Leukemia, MESH:D015470		Arsenic Tr	24.25	92
C12ORF57	113246	Leukemia, MESH:D015470		Doxorubic	24.25	14
CH25H	9023	Leukemia, MESH:D015470		Dexameth	24.25	17
CNBP	7555	Leukemia, MESH:D015473		Antimony	24.25	263
COMTD1	118881	Leukemia, MESH:D015470		Dexameth	24.25	20
CYP27A1	1593	Leukemia, MESH:D015470		Alitretinoi	24.25	82
DENND10	404636	Leukemia, MESH:D015470		Air Polluta	24.25	13
FETUB	26998	Leukemia, MESH:D015470		Dexameth	24.25	16
KMO	8564	Leukemia, MESH:D015470		Air Polluta	24.25	81
KNTC1	9735	Leukemia, MESH:D015470		Calcitriol|D	24.25	14
MARCHF4	57574	Leukemia, MESH:D015470		Arsenic Tr	24.25	15
MSX1	4487	Leukemia, MESH:D015470		Cytarabine	24.25	90
PCBD2	84105	Leukemia, MESH:D015470		Doxorubic	24.25	14
PLD3	23646	Leukemia, MESH:D015473		Arsenic Tr	24.25	263
PSME4	23198	Leukemia, MESH:D015470		Arsenic Tr	24.25	35
SESN3	143686	Leukemia, MESH:D015470		Cytarabine	24.25	86
TUBB3	10381	Leukemia, MESH:D015470		2-(2-chlor	24.25	91
TRIB3	57761	Leukemia, MESH:D01	marker/m	Arsenic Tr	24.24	262
FZD5	7855	Leukemia, MESH:D015473		Arsenic Tr	24.24	261
IRF4	3662	Leukemia, MESH:D015473		Arsenic|Ar	24.24	161
TNFRSF10	8793	Leukemia, MESH:D015473		Arsenic|Ar	24.24	266
CCND3	896	Leukemia, MESH:D007948		2-(2-amin	24.23	4
ARHGDIB	397	Leukemia, MESH:D015470		Arsenic Tr	24.22	92
CALD1	800	Leukemia, MESH:D015470		Arsenic Tr	24.22	28
DDAH1	23576	Leukemia, MESH:D015470		Dexameth	24.22	17
DLG4	1742	Leukemia, MESH:D015470		Decitabine	24.22	25
MSH6	2956	Leukemia, MESH:D015470		Bortezomi	24.22	34
SNAI1	6615	Leukemia, MESH:D007948		2-(2-amin	24.22	3
TAGLN2	8407	Leukemia, MESH:D015470		Bortezomi	24.22	19
TNFAIP2	7127	Leukemia, MESH:D015470		Calcitriol|D	24.22	21
AIMP2	7965	Leukemia, MESH:D015470		Doxorubic	24.19	18
ATP10D	57205	Leukemia, MESH:D015470		Cytarabine	24.19	82
CBFA2T3	863	Leukemia, MESH:D015470		Dexameth	24.19	11
DESI2	51029	Leukemia, MESH:D015470		Arsenic Tr	24.19	29
GABRP	2568	Leukemia, MESH:D015470		Calcitriol|C	24.19	80
GBP3	2635	Leukemia, MESH:D015470		Air Polluta	24.19	15
GJB3	2707	Leukemia, MESH:D015470		Arsenic Tr	24.19	24
GKAP1	80318	Leukemia, MESH:D015470		Bortezomi	24.19	11
HLA-E	3133	Leukemia, MESH:D015470		Arsenic Tr	24.19	28
ITGA11	22801	Leukemia, MESH:D015470		Calcitriol|D	24.19	10
MGAT4B	11282	Leukemia, MESH:D015470		Arsenic Tr	24.19	16
MUC1	4582	Leukemia, MESH:D015473		Arsenic|ca	24.19	158
MYOM2	9172	Leukemia, MESH:D015470		Chloroqui	24.19	58
NDUFB9	4715	Leukemia, MESH:D015470		Dexameth	24.19	16

ORAI1	84876	Leukemia, MESH:D015470	Arsenic Tr	24.19	18
PLAGL2	5326	Leukemia, MESH:D015470	Arsenic Tr	24.19	15
SOX7	83595	Leukemia, MESH:D015470	Doxorubic	24.19	16
UNKL	64718	Leukemia, MESH:D015470	Bortezomi	24.19	10
ADGRD1	283383	Leukemia, MESH:D015470	Dexameth	24.18	15
CXCL12	6387	Leukemia, MESH:D015473	Arsenic Tr	24.18	261
AIFM2	84883	Leukemia, MESH:D015470	Bortezomi	24.16	19
CCN4	8840	Leukemia, MESH:D015473	Arsenic|Ar	24.16	263
CLSPN	63967	Leukemia, MESH:D015473	Arsenic Tr	24.16	262
DRAM1	55332	Leukemia, MESH:D015470	Decitabine	24.16	27
GJA4	2701	Leukemia, MESH:D015473	Arsenic|Ar	24.16	265
JADE1	79960	Leukemia, MESH:D015470	Dexameth	24.16	14
MYO10	4651	Leukemia, MESH:D015470	Doxorubic	24.16	18
TFF3	7033	Leukemia, MESH:D015470	Cytarabine	24.16	84
TRA2A	29896	Leukemia, MESH:D015470	Arsenic Tr	24.16	22
ARRDC3	57561	Leukemia, MESH:D015470	Arsenic Tr	24.15	24
CASP12	1.01E+08	Leukemia, MESH:D007948	2-(2-amin	24.15	3
CYP26B1	56603	Leukemia, MESH:D015473	Arsenic|ars	24.13	161
GREB1	9687	Leukemia, MESH:D015473	Arsenic|Ar	24.13	162
MAP3K8	1326	Leukemia, MESH:D015473	Arsenic|ars	24.13	159
TAFA5	25817	Leukemia, MESH:D015473	Arsenic|Ar	24.13	261
LGALS3	3958	Leukemia, MESH:D015473	Arsenic Tr	24.12	264
RGS2	5997	Leukemia, MESH:D015473	arsenic dis	24.12	265
MMP2	4313	Leukemia, MESH:D015473	Arsenic|Ar	24.11	267
TNFRSF11	4982	Leukemia, MESH:D007948	2-(2-amin	24.11	3
CISH	1154	Leukemia, MESH:D015470	Benzene|B	24.08	32
COL12A1	1303	Leukemia, MESH:D015470	Alitretinoi	24.08	18
DNAJB1	3337	Leukemia, MESH:D015473	Antimony	24.08	264
LMNA	4000	Leukemia, MESH:D015473	Arsenic|Ar	24.08	162
PRKAA2	5563	Leukemia, MESH:D015470	Dexameth	24.08	19
TES	26136	Leukemia, MESH:D015470	Arsenic Tr	24.08	25
TUBA1A	7846	Leukemia, MESH:D015470	Alitretinoi	24.08	20
CD82	3732	Leukemia, MESH:D015470	Bortezomi	24.07	17
DCT	1638	Leukemia, MESH:D015470	Carboplati	24.07	27
NRIP1	8204	Leukemia, MESH:D015473	arsenite|Ca	24.07	159
PBX1	5087	Leukemia, MESH:D015470	Calcitriol|D	24.07	33
PKIB	5570	Leukemia, MESH:D015473	Arsenic|Ar	24.07	162
RAD23A	5886	Leukemia, MESH:D015470	Arsenic Tr	24.07	85
SKA1	220134	Leukemia, MESH:D015473	Arsenic|ars	24.07	160
TBC1D4	9882	Leukemia, MESH:D015470	Alitretinoi	24.07	82
TERT	7015	Leukemia, MESH:D007948	1,3-benzo	24.07	3
TLN1	7094	Leukemia, MESH:D015470	Arsenic Tr	24.07	25
ACTR3	10096	Leukemia, MESH:D015470	Arsenic Tr	24.06	18
ARAP1	116985	Leukemia, MESH:D015470	Air Polluta	24.06	23
CASTOR1	652968	Leukemia, MESH:D015470	Calcitriol|D	24.06	14
DNAAF5	54919	Leukemia, MESH:D015470	Bortezomi	24.06	11
DNAJC12	56521	Leukemia, MESH:D015470	Decitabine	24.06	23
EPB41L1	2036	Leukemia, MESH:D015470	Arsenic Tr	24.06	35
FAM171B	165215	Leukemia, MESH:D015470	Dexameth	24.06	14
ITPRID2	6744	Leukemia, MESH:D015470	Arsenic Tr	24.06	19
LZTS1	11178	Leukemia, MESH:D015470	Calcitriol|D	24.06	17
S1PR3	1903	Leukemia, MESH:D015470	Decitabine	24.06	24
SLC14A2	8170	Leukemia, MESH:D015470	Arsenic Tr	24.06	21
STX12	23673	Leukemia, MESH:D015470	Bortezomi	24.06	14
THBS3	7059	Leukemia, MESH:D015470	Dasatinib|I	24.06	15
UBE2D4	51619	Leukemia, MESH:D015470	Dexameth	24.06	14
ZC3H11A	9877	Leukemia, MESH:D015470	Arsenic Tr	24.06	16
AMPH	273	Leukemia, MESH:D015470	Dexameth	24.05	10

C1ORF54	79630	Leukemia, MESH:D015470		Decitabine	24.05	17
CC2D1A	54862	Leukemia, MESH:D015470		Dexameth	24.05	14
DENND4B	9909	Leukemia, MESH:D015470		Bortezomi	24.05	15
GLCE	26035	Leukemia, MESH:D015470		Doxorubic	24.05	16
MDM1	56890	Leukemia, MESH:D015470		Calcitriol|D	24.05	15
PBX2	5089	Leukemia, MESH:D015470		Dexameth	24.05	17
PLPPR2	64748	Leukemia, MESH:D015470		Dexameth	24.05	14
PNMA1	9240	Leukemia, MESH:D015470		Air Polluta	24.05	19
SESN2	83667	Leukemia, MESH:D015473		Antineopla	24.04	157
AHCY	191	Leukemia, MESH:D015473		Arsenic|Ar	24.02	266
EFEMP1	2202	Leukemia, MESH:D015473		Anthracyc	24.02	153
DBI	1622	Leukemia, MESH:D015470		Bezafibrat	24.01	20
EPHX2	2053	Leukemia, MESH:D015470		Air Polluta	24.01	27
FMO5	2330	Leukemia, MESH:D015470		Calcitriol|D	24.01	19
NCOR2	9612	Leukemia, MESH:D015470		Calcitriol|D	24.01	13
PSEN1	5663	Leukemia, MESH:D015470		Air Polluta	24.01	16
ARNT	405	Leukemia, MESH:D015473		arsenite|Bu	24	156
CLDN1	9076	Leukemia, MESH:D015473		Arsenic|Ar	24	265
SOX9	6662	Leukemia, MESH:D015473		Arsenic|ars	24	161
MND1	84057	Leukemia, MESH:D015473		Arsenic Tr	23.99	264
GYPA	2993	Leukemia, MESH:D007948		Arsenic Tr	23.98	3
KIAA1958	158405	Leukemia, MESH:D015470		Dexameth	23.98	13
MIR17	406952	Leukemia, MESH:D015473		Arsenic Tr	23.98	155
MIR20B	574032	Leukemia, MESH:D015473		Arsenic Tr	23.98	260
MYH11	4629	Leukemia, MESH:D01	marker/m	Dasatinib|I	23.97	29
ADIRF	10974	Leukemia, MESH:D015473		Arsenic|De	23.97	156
ANK2	287	Leukemia, MESH:D015470		Air Polluta	23.97	48
COL21A1	81578	Leukemia, MESH:D015473		Arsenic|Ar	23.97	264
LAMP3	27074	Leukemia, MESH:D015470		Decitabine	23.97	25
PGR	5241	Leukemia, MESH:D015470		Amsacrine	23.97	40
PRICKLE1	144165	Leukemia, MESH:D015470		Cytarabine	23.97	83
MAP3K1	4214	Leukemia, MESH:D015473		Arsenic|Ca	23.96	160
SLC7A5	8140	Leukemia, MESH:D015473		Arsenic|Ch	23.96	160
BLVRB	645	Leukemia, MESH:D015470		Arsenic Tr	23.94	29
CBR3	874	Leukemia, MESH:D015470		alvocidib|E	23.94	45
CIDEA	1149	Leukemia, MESH:D015470		Arsenic Tr	23.94	25
IRAK1	3654	Leukemia, MESH:D015470		Arsenic Tr	23.94	26
ACTR2	10097	Leukemia, MESH:D015470		Dexameth	23.93	14
ADCY4	196883	Leukemia, MESH:D015470		Dexameth	23.93	16
AGPAT5	55326	Leukemia, MESH:D015470		Air Polluta	23.93	24
EIF4H	7458	Leukemia, MESH:D015470		Chloroqui	23.93	81
ELP4	26610	Leukemia, MESH:D015470		Calcitriol|D	23.93	17
F2RL2	2151	Leukemia, MESH:D015470		Calcitriol|C	23.93	15
FAM3C	10447	Leukemia, MESH:D015470		Alitretinoi	23.93	10
GABARAP	11337	Leukemia, MESH:D015470		Dexameth	23.93	16
GNA11	2767	Leukemia, MESH:D015470		Arsenic Tr	23.93	23
H1-10	8971	Leukemia, MESH:D015470		Doxorubic	23.93	17
HPDL	84842	Leukemia, MESH:D015470		Dexameth	23.93	13
ITSN2	50618	Leukemia, MESH:D015470		Air Polluta	23.93	10
MARCHF2	51257	Leukemia, MESH:D015470		Arsenic Tr	23.93	20
RRP1B	23076	Leukemia, MESH:D015470		Air Polluta	23.93	16
SERINC1	57515	Leukemia, MESH:D015470		Arsenic Tr	23.93	20
SLC4A11	83959	Leukemia, MESH:D015470		Calcitriol|D	23.93	24
SNRPA1	6627	Leukemia, MESH:D015470		Air Polluta	23.93	14
ST7	7982	Leukemia, MESH:D015470		Calcitriol|D	23.93	23
SUN2	25777	Leukemia, MESH:D015470		Decitabine	23.93	25
SWAP70	23075	Leukemia, MESH:D015470		Arsenic Tr	23.93	26
TMEM64	169200	Leukemia, MESH:D015470		Dexameth	23.93	16

CCNE2	9134	Leukemia, MESH:D015473		Arsenic|ars	23.92	164
VDR	7421	Leukemia, MESH:D007948		2-(2-amin	23.92	3
GPER1	2852	Leukemia, MESH:D015473		Arsenic Tr	23.91	266
ITGAV	3685	Leukemia, MESH:D015473		Arsenic Tr	23.91	263
RGCC	28984	Leukemia, MESH:D015473		Arsenic|Ar	23.91	263
RSAD2	91543	Leukemia, MESH:D015473		Arsenic|ars	23.91	160
SEC31A	22872	Leukemia, MESH:D015473		Arsenic|ars	23.91	165
RAD18	56852	Leukemia, MESH:D015473		Arsenic|ars	23.9	161
PXDN	7837	Leukemia, MESH:D01	marker/me	Arsenic Tr	23.88	32
ECI2	10455	Leukemia, MESH:D015470		Bezafibrat	23.88	19
ERCC2	2068	Leukemia, MESH:D015470		Air Polluta	23.88	35
MAP4	4134	Leukemia, MESH:D015470		Benzene|D	23.88	32
MRPS18B	28973	Leukemia, MESH:D015470		Dexameth	23.88	16
RAD51C	5889	Leukemia, MESH:D015470		Arsenic Tr	23.88	23
UGP2	7360	Leukemia, MESH:D015470		Air Polluta	23.88	20
COL2A1	1280	Leukemia, MESH:D015470		Decitabine	23.87	29
FKBP4	2288	Leukemia, MESH:D015470		Dexameth	23.87	20
GREB1	9687	Leukemia, MESH:D015470		Arsenic Tr	23.87	24
IDH3A	3419	Leukemia, MESH:D015470		Bortezomi	23.87	29
MGST2	4258	Leukemia, MESH:D015470		Busulfan|D	23.87	20
WNT4	54361	Leukemia, MESH:D015470		Androgen	23.87	17
ADGRB3	577	Leukemia, MESH:D015470		Benzene|C	23.86	90
BAIAP2L2	80115	Leukemia, MESH:D015470		Calcitriol|D	23.86	9
COQ8B	79934	Leukemia, MESH:D015470		Dexameth	23.86	13
DNAJC14	85406	Leukemia, MESH:D015470		Bortezomi	23.86	14
ELAPOR2	222223	Leukemia, MESH:D015470		Benzene|D	23.86	23
ERAP2	64167	Leukemia, MESH:D015470		Air Polluta	23.86	17
NOX4	50507	Leukemia, MESH:D007948		2-(2-amin	23.86	4
PLPP4	196051	Leukemia, MESH:D015470		Calcitriol|D	23.86	14
PPP1R3D	5509	Leukemia, MESH:D015470		Arsenic Tr	23.86	19
PRR3	80742	Leukemia, MESH:D015470		Arsenic Tr	23.86	24
RASL10A	10633	Leukemia, MESH:D015470		Arsenic Tr	23.86	21
TOR2A	27433	Leukemia, MESH:D015470		Dexameth	23.86	10
ANPEP	290	Leukemia, MESH:D015473		Arsenic|Ar	23.85	265
COL5A2	1290	Leukemia, MESH:D015473		Arsenic|ars	23.85	161
KYNU	8942	Leukemia, MESH:D015473		Arsenic Tr	23.85	265
SDHA	6389	Leukemia, MESH:D015473		Arsenic|Ar	23.85	266
ID3	3399	Leukemia, MESH:D015473		arsenite|D	23.84	158
NDUFA4L2	56901	Leukemia, MESH:D015473		Arsenic|ars	23.84	12
OLAH	55301	Leukemia, MESH:D015473		Arsenic|Ca	23.84	155
MIR16-1	406950	Leukemia, MESH:D01	therapeuti	Arsenic Tr	23.83	261
AXL	558	Leukemia, MESH:D004915		Cytarabine	23.83	3
BAX	581	Leukemia, MESH:D004915		Cytarabine	23.83	4
EDRF1	26098	Leukemia, MESH:D015473		Arsenic Tr	23.83	265
FABP1	2168	Leukemia, MESH:D015470		Bezafibrat	23.83	17
IGFBP1	3484	Leukemia, MESH:D015470		Arsenic Tr	23.83	28
MMP13	4322	Leukemia, MESH:D007948		2-(2-amin	23.83	3
ADAMTS4	9507	Leukemia, MESH:D015473		Arsenic Tr	23.82	263
CDC42EP3	10602	Leukemia, MESH:D015473		Arsenic Tr	23.82	264
CIT	11113	Leukemia, MESH:D015473		Arsenic|ars	23.82	14
COX6A2	1339	Leukemia, MESH:D015473		Arsenic|Ar	23.82	263
CRABP1	1381	Leukemia, MESH:D015473		Arsenic Tr	23.82	264
EOMES	8320	Leukemia, MESH:D015473		arsenite|Ca	23.82	157
KIF14	9928	Leukemia, MESH:D015473		arsenite|Ca	23.82	157
MMP9	4318	Leukemia, MESH:D015473		Arsenic|ars	23.82	269
NELL2	4753	Leukemia, MESH:D015473		Calcitriol|C	23.82	159
NUDT1	4521	Leukemia, MESH:D015473		Arsenic|ars	23.82	161
PCDH17	27253	Leukemia, MESH:D015473		Arsenic|Ar	23.82	265

WNK1	65125	Leukemia, MESH:D015473		Arsenic|ars	23.82	162
NUPR1	26471	Leukemia, MESH:D015473		Arsenic Tr	23.81	263
SQLE	6713	Leukemia, MESH:D015473		Antimony	23.81	262
ALCAM	214	Leukemia, MESH:D015470		Benzoates	23.8	14
CDC20	991	Leukemia, MESH:D015473		Arsenic|Ar	23.8	266
CDC42EP5	148170	Leukemia, MESH:D015470		Cytarabine	23.8	83
CDH8	1006	Leukemia, MESH:D015470		Arsenic Tr	23.8	29
CHD1	1105	Leukemia, MESH:D015470		Dexameth	23.8	15
CHST14	113189	Leukemia, MESH:D015470		Azacitidine	23.8	16
CILP	8483	Leukemia, MESH:D015470		Calcitriol|D	23.8	15
CRYBG1	202	Leukemia, MESH:D015470		Benzene|D	23.8	25
HERC4	26091	Leukemia, MESH:D015470		Air Polluta	23.8	14
HTRA1	5654	Leukemia, MESH:D015470		Cytarabine	23.8	85
LACC1	144811	Leukemia, MESH:D015470		Calcitriol|D	23.8	15
MSRB2	22921	Leukemia, MESH:D015470		Arsenic Tr	23.8	21
MYL12A	10627	Leukemia, MESH:D015470		Bortezomi	23.8	19
P2RX2	22953	Leukemia, MESH:D015470		Alitretinoi	23.8	17
RBM19	9904	Leukemia, MESH:D015470		Dexameth	23.8	17
RMI2	116028	Leukemia, MESH:D015470		Arsenic Tr	23.8	17
SCHIP1	29970	Leukemia, MESH:D015470		Bortezomi	23.8	25
SESN1	27244	Leukemia, MESH:D015473		Arsenic Tr	23.8	159
SETD2	29072	Leukemia, MESH:D015470		Deferoxan	23.8	14
SSBP3	23648	Leukemia, MESH:D015470		Arsenic Tr	23.8	21
THEMIS2	9473	Leukemia, MESH:D015470		Doxorubic	23.8	18
UGGT2	55757	Leukemia, MESH:D015470		Dexameth	23.8	18
YWHAZ	7534	Leukemia, MESH:D015470		Arsenic Tr	23.8	23
PSIP1	11168	Leukemia, MESH:D01	marker/m	Decitabine	23.79	25
ACOT7	11332	Leukemia, MESH:D015470		Bortezomi	23.79	15
CDH11	1009	Leukemia, MESH:D015470		Cytarabine	23.79	89
CNP	1267	Leukemia, MESH:D015470		Dexameth	23.79	16
CXADR	1525	Leukemia, MESH:D015470		Calcitriol|D	23.79	30
DHX9	1660	Leukemia, MESH:D015470		Arsenic Tr	23.79	36
EDEM1	9695	Leukemia, MESH:D015470		Air Polluta	23.79	21
IPO5	3843	Leukemia, MESH:D015470		Air Polluta	23.79	19
IRF8	3394	Leukemia, MESH:D015470		Benzoates	23.79	17
MCF2L	23263	Leukemia, MESH:D015470		Arsenic Tr	23.79	24
METRNL	284207	Leukemia, MESH:D015470		Calcitriol|D	23.79	13
NDUFS4	4724	Leukemia, MESH:D015470		Dexameth	23.79	16
PDHB	5162	Leukemia, MESH:D015470		Deferoxan	23.79	16
IL17A	3605	Leukemia, MESH:D007948		2-(2-amin	23.77	3
NCF1	653361	Leukemia, MESH:D007948		2-(2-amin	23.77	3
ANK1	286	Leukemia, MESH:D015473		Arsenic|Ca	23.74	157
COX6B1	1340	Leukemia, MESH:D015473		Arsenic|ars	23.74	160
CTBP2	1488	Leukemia, MESH:D015473		Arsenic|Ar	23.74	265
EEF1B2	1933	Leukemia, MESH:D015473		Arsenic|Ar	23.74	265
LBP	3929	Leukemia, MESH:D015473		arsenite|Ca	23.74	158
PDZD2	23037	Leukemia, MESH:D015473		Arsenic|Ar	23.74	267
TAP2	6891	Leukemia, MESH:D015473		Arsenic|Da	23.74	166
TROAP	10024	Leukemia, MESH:D015473		Arsenic|Ca	23.74	163
CIRBP	1153	Leukemia, MESH:D015470		Dexameth	23.73	21
PTK2	5747	Leukemia, MESH:D015473		Arsenic|ars	23.73	160
CHD2	1106	Leukemia, MESH:D015470		Arsenic Tr	23.7	18
IKBKE	9641	Leukemia, MESH:D015470		Benzene|C	23.7	55
QSOX1	5768	Leukemia, MESH:D015470		Arsenic Tr	23.7	17
AHNAK	79026	Leukemia, MESH:D015473		arsenite|Ca	23.69	163
CCDC93	54520	Leukemia, MESH:D015473		arsenite|Ca	23.69	159
CDC42	998	Leukemia, MESH:D015473		Arsenic|Ar	23.69	267
CENPO	79172	Leukemia, MESH:D015473		Calcitriol|D	23.69	156

DNM1L	10059	Leukemia, MESH:D015470	Arsenic Tr	23.69	24
HERPUD1	9709	Leukemia, MESH:D015470	Benzene|D	23.69	34
LIMD1	8994	Leukemia, MESH:D015473	Arsenic|Ar	23.69	161
MX1	4599	Leukemia, MESH:D015473	alvocidib|A	23.69	264
TSC22D3	1831	Leukemia, MESH:D015473	Antimony	23.69	265
CAPN3	825	Leukemia, MESH:D015470	Alitretinoi	23.68	22
CYP4A11	1579	Leukemia, MESH:D015470	15-deoxy-	23.68	13
FAM110C	642273	Leukemia, MESH:D015470	Calcitriol|D	23.68	12
IMPDH1	3614	Leukemia, MESH:D015470	Dexameth	23.68	17
LZTFL1	54585	Leukemia, MESH:D015470	Arsenic Tr	23.68	31
NAP1L5	266812	Leukemia, MESH:D015470	Calcitriol|D	23.68	10
NEUROG1	4762	Leukemia, MESH:D015473	Arsenic|ars	23.68	158
NLGN3	54413	Leukemia, MESH:D015470	Azacitidine	23.68	17
PCDH1	5097	Leukemia, MESH:D015470	Calcitriol|D	23.68	16
PDZK1IP1	10158	Leukemia, MESH:D015470	Benzene|C	23.68	26
PPP2R5A	5525	Leukemia, MESH:D015470	Arsenic Tr	23.68	21
PSTPIP2	9050	Leukemia, MESH:D015470	Cytarabine	23.68	80
RHOJ	57381	Leukemia, MESH:D015470	Dexameth	23.68	17
S100A16	140576	Leukemia, MESH:D015470	Doxorubic	23.68	19
SAC3D1	29901	Leukemia, MESH:D015470	Dexameth	23.68	15
SLC25A11	8402	Leukemia, MESH:D015470	Air Polluta	23.68	29
SRPX2	27286	Leukemia, MESH:D015470	Dexameth	23.68	16
STARD5	80765	Leukemia, MESH:D015470	Decitabine	23.68	20
STXBP2	6813	Leukemia, MESH:D015470	Dexameth	23.68	10
TTF2	8458	Leukemia, MESH:D015470	Dexameth	23.68	19
ABCB5	340273	Leukemia, MESH:D015470	Calcitriol|D	23.67	15
CTSG	1511	Leukemia, MESH:D015470	Arsenic Tr	23.67	22
EXOC6B	23233	Leukemia, MESH:D015470	Doxorubic	23.67	14
HSF1	3297	Leukemia, MESH:D007948	2-(2-amin	23.67	3
KIAA1549I	25758	Leukemia, MESH:D015470	Dexameth	23.67	13
MYRFL	196446	Leukemia, MESH:D015470	Decitabine	23.67	15
SMG6	23293	Leukemia, MESH:D015470	Dexameth	23.67	15
TMEM200	114801	Leukemia, MESH:D015470	Calcitriol|C	23.67	78
UBE2Q1	55585	Leukemia, MESH:D015470	Benzene|D	23.67	28
ECT2	1894	Leukemia, MESH:D015470	Benzene|C	23.66	32
FAM107B	83641	Leukemia, MESH:D015473	Arsenic|Ar	23.66	267
GNAI2	2771	Leukemia, MESH:D015470	Dexameth	23.66	19
PAX6	5080	Leukemia, MESH:D015470	Benzene|C	23.66	104
PRC1	9055	Leukemia, MESH:D004915	Cytarabine	23.66	2
RFX8	731220	Leukemia, MESH:D015470	Calcitriol|D	23.66	9
SAMD4A	23034	Leukemia, MESH:D015473	Arsenic|ars	23.66	160
SPTLC2	9517	Leukemia, MESH:D015473	Arsenic Tr	23.66	261
AKR1C2	1646	Leukemia, MESH:D015470	Arsenic Tr	23.65	52
C8ORF34	116328	Leukemia, MESH:D015470	Calcitriol|D	23.65	7
CYP2D6	1565	Leukemia, MESH:D015470	Arsenic Tr	23.65	37
DHRS4-AS	55449	Leukemia, MESH:D015470	Decitabine	23.65	19
SNAI1	6615	Leukemia, MESH:D015473	Arsenic|Ar	23.65	266
BMP7	655	Leukemia, MESH:D015473	arsenite|Ca	23.64	160
IL1R2	7850	Leukemia, MESH:D015473	Arsenic|Ar	23.64	162
MSH2	4436	Leukemia, MESH:D015473	Arsenic|Ar	23.64	264
AKR1B10	57016	Leukemia, MESH:D015473	Antimony	23.62	188
CBL	867	Leukemia, MESH:D015470	Arsenic Tr	23.61	23
FAM162A	26355	Leukemia, MESH:D015470	Dexameth	23.61	16
FAM83D	81610	Leukemia, MESH:D015470	Calcitriol|D	23.61	20
HNRNPAE	3182	Leukemia, MESH:D015470	Dexameth	23.61	15
NAV2	89797	Leukemia, MESH:D015470	Cytarabine	23.61	79
PCDH7	5099	Leukemia, MESH:D015470	Calcitriol|C	23.61	81
WFDC2	10406	Leukemia, MESH:D015470	Calcitriol|D	23.61	22

ZYX	7791	Leukemia, MESH:D015470	Calcitriol|D	23.61	18
ITGAV	3685	Leukemia, MESH:D015470	Arsenic Tr	23.59	20
MAP1B	4131	Leukemia, MESH:D015470	Arsenic Tr	23.59	49
MIR21	406991	Leukemia, MESH:D015473	Arsenic|Ar	23.59	270
PMP22	5376	Leukemia, MESH:D015473	Arsenic|Cy	23.59	162
RNASEH2A	10535	Leukemia, MESH:D015473	Antimony	23.59	262
RPS27A	6233	Leukemia, MESH:D015473	arsenic dis	23.59	264
SLC1A1	6505	Leukemia, MESH:D015473	arsenite|ca	23.59	162
ATIC	471	Leukemia, MESH:D015473	Arsenic Tr	23.58	262
COL5A3	50509	Leukemia, MESH:D015473	arsenite|Bu	23.58	155
FAM20C	56975	Leukemia, MESH:D015473	Arsenic|Ar	23.58	264
JAM2	58494	Leukemia, MESH:D015473	Arsenic|Ar	23.58	170
KALRN	8997	Leukemia, MESH:D015473	Arsenic|Ar	23.58	162
KCNN2	3781	Leukemia, MESH:D015473	Arsenic Tr	23.58	265
NRG1	3084	Leukemia, MESH:D015473	Arsenic|Ar	23.58	266
ZWILCH	55055	Leukemia, MESH:D015473	arsenite|Ca	23.58	158
AQP2	359	Leukemia, MESH:D015470	Chloroqui	23.56	16
FAM98A	25940	Leukemia, MESH:D015470	Benzene|D	23.56	28
HIRIP3	8479	Leukemia, MESH:D015470	Calcitriol|D	23.56	11
MBTPS1	8720	Leukemia, MESH:D015470	Dexameth	23.56	18
NOL6	65083	Leukemia, MESH:D015470	Arsenic Tr	23.56	18
DMKN	93099	Leukemia, MESH:D015470	Calcitriol|D	23.55	11
FUT11	170384	Leukemia, MESH:D015473	Arsenic Tr	23.55	262
HUWE1	10075	Leukemia, MESH:D015470	Arsenic Tr	23.55	22
MAN2B2	23324	Leukemia, MESH:D015470	Cyclophos	23.55	22
OSBPL8	114882	Leukemia, MESH:D015470	Arsenic Tr	23.55	17
SYTL5	94122	Leukemia, MESH:D015470	(+)-JQ1 cc	23.55	15
CASP8	841	Leukemia, MESH:D004915	Cytarabine	23.54	3
NCF2	4688	Leukemia, MESH:D015473	Arsenic Tr	23.54	268
SLC16A3	9123	Leukemia, MESH:D015473	Arsenic Tr	23.54	269
SLC29A1	2030	Leukemia, MESH:D015473	Arsenic|Ar	23.54	268
ACACB	32	Leukemia, MESH:D015473	Arsenic|ars	23.53	160
CCDC80	151887	Leukemia, MESH:D015470	Calcitriol|D	23.53	17
DAG1	1605	Leukemia, MESH:D015470	Arsenic Tr	23.53	22
EBF1	1879	Leukemia, MESH:D015470	Dexameth	23.53	20
EIF3A	8661	Leukemia, MESH:D015470	Dexameth	23.53	25
FAM13A	10144	Leukemia, MESH:D015470	Dexameth	23.53	18
HAL	3034	Leukemia, MESH:D015470	Air Polluta	23.53	21
HSDL2	84263	Leukemia, MESH:D015470	Decitabine	23.53	23
KPNB1	3837	Leukemia, MESH:D015470	Calcitriol|D	23.53	19
PAK1	5058	Leukemia, MESH:D015470	Air Polluta	23.53	18
TBL1XR1	79718	Leukemia, MESH:D015470	Dexameth	23.53	19
TXN2	25828	Leukemia, MESH:D015470	Air Polluta	23.53	32
WWOX	51741	Leukemia, MESH:D015470	Arsenic Tr	23.53	23
YWHAQ	10971	Leukemia, MESH:D015470	Arsenic Tr	23.53	37
ABCB11	8647	Leukemia, MESH:D015470	Allopurinc	23.52	17
BCL2L1	598	Leukemia, MESH:D007948	2-(2-amin	23.52	4
ARHGAP5	394	Leukemia, MESH:D015473	Arsenic Tr	23.5	157
BBC3	27113	Leukemia, MESH:D007948	Arsenic Tr	23.5	4
BCOR	54880	Leukemia, MESH:D015473	arsenite|D	23.5	158
CSF3	1440	Leukemia, MESH:D015473	Arsenic Tr	23.5	265
EMP2	2013	Leukemia, MESH:D015473	Arsenic Tr	23.5	262
FGD4	121512	Leukemia, MESH:D015473	Arsenic|Ca	23.5	159
MAGI1	9223	Leukemia, MESH:D015473	Arsenic|ars	23.5	161
RPS6	6194	Leukemia, MESH:D007948	2-(2-amin	23.5	3
DCLRE1A	9937	Leukemia, MESH:D015470	Dexameth	23.49	14
DCLRE1B	64858	Leukemia, MESH:D015470	Dexameth	23.49	15
EEFSEC	60678	Leukemia, MESH:D015470	Arsenic Tr	23.49	19

PIGH	5283	Leukemia, MESH:D015470		Arsenic Tr	23.49	85
TSPOAP1	9256	Leukemia, MESH:D015470		Arsenic Tr	23.49	20
TNFRSF9	3604	Leukemia, MESH:D015473		Arsenic Tr	23.48	262
HSP90AB1	3326	Leukemia, MESH:D015473		Arsenic Tr	23.47	266
SLC1A3	6507	Leukemia, MESH:D015470		Calcitriol|D	23.46	19
AGT	183	Leukemia, MESH:D015473		alpha-Toc	23.45	162
ABCF1	23	Leukemia, MESH:D015470		arsenic tris	23.44	17
CAB39L	81617	Leukemia, MESH:D015470		Arsenic Tr	23.44	83
CCDC6	8030	Leukemia, MESH:D015470		Arsenic Tr	23.44	28
CLEC11A	6320	Leukemia, MESH:D015470		Arsenic Tr	23.44	15
F11R	50848	Leukemia, MESH:D015470		Dexameth	23.44	25
GRK5	2869	Leukemia, MESH:D015470		Calcitriol|C	23.44	82
HACL1	26061	Leukemia, MESH:D015470		Arsenic Tr	23.44	41
IVNS1ABP	10625	Leukemia, MESH:D015470		Air Polluta	23.44	20
MKNK2	2872	Leukemia, MESH:D015470		Arsenic Tr	23.44	28
NDUFB5	4711	Leukemia, MESH:D015470		Dexameth	23.44	15
NEU1	4758	Leukemia, MESH:D015470		Dexameth	23.44	16
NFATC2	4773	Leukemia, MESH:D015470		Calcitriol|C	23.44	14
RAB23	51715	Leukemia, MESH:D015470		Calcitriol|D	23.44	23
TERF1	7013	Leukemia, MESH:D015470		Arsenic Tr	23.44	36
TLR1	7096	Leukemia, MESH:D015470		Air Polluta	23.44	32
TLR8	51311	Leukemia, MESH:D015470		Air Polluta	23.44	13
TPT1	7178	Leukemia, MESH:D015470		Arsenic Tr	23.44	37
XPO5	57510	Leukemia, MESH:D015470		Benzene|B	23.44	28
AKR1B1	231	Leukemia, MESH:D015473		alpha-Toc	23.43	266
FUT4	2526	Leukemia, MESH:D015470		Arsenic Tr	23.43	20
HTRA2	27429	Leukemia, MESH:D015473		alvocidib|A	23.43	263
PHF11	51131	Leukemia, MESH:D015470		Decitabine	23.43	24
RNF24	11237	Leukemia, MESH:D015470		Arsenic Tr	23.43	20
SERPINB8	5271	Leukemia, MESH:D015473		Arsenic|Ca	23.43	160
SERPINH1	871	Leukemia, MESH:D015473		Arsenic|Ar	23.43	267
SPTBN1	6711	Leukemia, MESH:D015473		Arsenic|Ar	23.43	266
UBC	7316	Leukemia, MESH:D015473		Arsenic|Ar	23.43	265
ZFYVE16	9765	Leukemia, MESH:D015470		Arsenic Tr	23.43	24
DSC1	1823	Leukemia, MESH:D015473		Antimony	23.42	157
EHHADH	1962	Leukemia, MESH:D015470		Bezafibrat	23.42	21
KLF13	51621	Leukemia, MESH:D015473		Arsenic|Ar	23.42	264
GATA2	2624	Leukemia, MESH:D01	marker/m	Arsenic Tr	23.39	32
ACTN1	87	Leukemia, MESH:D015470		Decitabine	23.39	26
LBP	3929	Leukemia, MESH:D015470		Calcitriol|D	23.39	20
IGF2R	3482	Leukemia, MESH:D015473		Arsenic|Ar	23.38	266
LDHB	3945	Leukemia, MESH:D015473		alpha-Toc	23.38	264
TKT	7086	Leukemia, MESH:D015473		Arsenic|Ar	23.38	266
ATF3	467	Leukemia, MESH:D015473		Arsenic|Ar	23.37	269
CD14	929	Leukemia, MESH:D007948		2-(2-amin	23.36	3
CD34	947	Leukemia, MESH:D015473		Arsenic Tr	23.36	266
MOCOS	55034	Leukemia, MESH:D015473		Arsenic Tr	23.36	160
POMZP3	22932	Leukemia, MESH:D015470		Doxorubic	23.36	29
AHSG	197	Leukemia, MESH:D015470		Alitretinoi	23.35	19
CD40	958	Leukemia, MESH:D015473		Arsenic|Ar	23.35	268
CHAF1A	10036	Leukemia, MESH:D015473		Arsenic|Ca	23.35	158
DBN1	1627	Leukemia, MESH:D015470		Arsenic Tr	23.35	88
ERCC6L	54821	Leukemia, MESH:D015473		arsenite|Ca	23.35	158
KCNQ2	3785	Leukemia, MESH:D015473		Arsenic|ars	23.35	156
NEAT1	283131	Leukemia, MESH:D015470		Arsenic Tr	23.35	21
PDHA1	5160	Leukemia, MESH:D015470		Arsenic Tr	23.35	32
PLA1A	51365	Leukemia, MESH:D015473		arsenite|D	23.35	9
RUNX3	864	Leukemia, MESH:D015473		Arsenic|ars	23.35	159

SLIT3	6586	Leukemia, MESH:D015470	Daunorub	23.35	43
TRIM25	7706	Leukemia, MESH:D015473	Arsenic Tr	23.35	266
VAT1	10493	Leukemia, MESH:D015470	Dexameth	23.35	13
CALCA	796	Leukemia, MESH:D015470	Azacitidine	23.34	25
ZNF384	171017	Leukemia, MESH:D015470	Dexameth	23.34	12
ZNF70	7621	Leukemia, MESH:D015470	Arsenic Tr	23.34	19
C1S	716	Leukemia, MESH:D015473	Arsenic|Ca	23.33	159
DAB2	1601	Leukemia, MESH:D015473	Arsenic|Ar	23.33	266
RGS16	6004	Leukemia, MESH:D015473	arsenite|Ca	23.33	161
RORA	6095	Leukemia, MESH:D015473	Arsenic|Ar	23.33	264
TAT	6898	Leukemia, MESH:D015470	Arsenic Tr	23.33	20
ACP3	55	Leukemia, MESH:D015470	Doxorubic	23.32	21
CDK8	1024	Leukemia, MESH:D015470	Decitabine	23.32	22
CLN3	1201	Leukemia, MESH:D015470	Air Polluta	23.32	24
CST7	8530	Leukemia, MESH:D015470	Benzoates	23.32	20
EPB41L4B	54566	Leukemia, MESH:D015470	Arsenic Tr	23.32	16
EYA4	2070	Leukemia, MESH:D015470	Arsenic Tr	23.32	20
FGFRL1	53834	Leukemia, MESH:D015470	Dexameth	23.32	14
FMO4	2329	Leukemia, MESH:D015470	Calcitriol|C	23.32	83
H2BC4	8347	Leukemia, MESH:D015470	Air Polluta	23.32	17
ITGAE	3682	Leukemia, MESH:D015470	Air Polluta	23.32	9
LLGL2	3993	Leukemia, MESH:D015470	Arsenic Tr	23.32	20
MBD4	8930	Leukemia, MESH:D015470	Arsenic Tr	23.32	24
NMI	9111	Leukemia, MESH:D015470	Azacitidine	23.32	11
OLFML1	283298	Leukemia, MESH:D015470	Calcitriol|D	23.32	14
PAK3	5063	Leukemia, MESH:D015470	Arsenic Tr	23.32	18
PDCD5	9141	Leukemia, MESH:D015470	Arsenic Tr	23.32	23
PEX19	5824	Leukemia, MESH:D015470	Alitretinoi	23.32	15
PSMD13	5719	Leukemia, MESH:D015470	Arsenic Tr	23.32	28
RNH1	6050	Leukemia, MESH:D015470	Arsenic Tr	23.32	22
RPS21	6227	Leukemia, MESH:D015470	Arsenic Tr	23.32	31
SLC16A14	151473	Leukemia, MESH:D015470	Calcitriol|C	23.32	13
SLC16A4	9122	Leukemia, MESH:D015470	Decitabine	23.32	21
SLC35G1	159371	Leukemia, MESH:D015470	Arsenic Tr	23.32	18
SPTB	6710	Leukemia, MESH:D015470	Arsenic Tr	23.32	20
TCIRG1	10312	Leukemia, MESH:D015470	Doxorubic	23.32	18
TERT	7015	Leukemia, MESH:D015473	alpha-Toc	23.32	267
TRERF1	55809	Leukemia, MESH:D015470	Arsenic Tr	23.32	24
UTP20	27340	Leukemia, MESH:D015470	Dexameth	23.32	10
ALG9	79796	Leukemia, MESH:D015470	Doxorubic	23.31	15
ARHGAP6	395	Leukemia, MESH:D015470	Dexameth	23.31	15
EDARADD	128178	Leukemia, MESH:D015470	Bortezomi	23.31	11
GGT7	2686	Leukemia, MESH:D015470	Dexameth	23.31	13
KBTBD7	84078	Leukemia, MESH:D015470	Decitabine	23.31	16
NUDT15	55270	Leukemia, MESH:D015470	Doxorubic	23.31	17
PAEP	5047	Leukemia, MESH:D015470	Azacitidine	23.31	15
RPRD2	23248	Leukemia, MESH:D015470	Doxorubic	23.31	15
SECTM1	6398	Leukemia, MESH:D015470	Air Polluta	23.31	20
SLC25A43	203427	Leukemia, MESH:D015470	Dexameth	23.31	14
SPRYD4	283377	Leukemia, MESH:D015470	Doxorubic	23.31	15
UBXN8	7993	Leukemia, MESH:D015470	Dronabinc	23.31	9
UNC50	25972	Leukemia, MESH:D015470	Dexameth	23.31	9
ITGAM	3684	Leukemia, MESH:D007948	2-(2-amin	23.29	3
AXL	558	Leukemia, MESH:D015473	Arsenic|ars	23.28	160
GPAM	57678	Leukemia, MESH:D015473	Cholestero	23.28	155
ITGB1	3688	Leukemia, MESH:D015473	Arsenic|ars	23.28	265
MTMR2	8898	Leukemia, MESH:D015473	Arsenic Tr	23.28	262
ABCA5	23461	Leukemia, MESH:D015473	Arsenic|ars	23.27	158

AMIGO2	347902	Leukemia, MESH:D015473	Dasatinib|I	23.27	153
ANK3	288	Leukemia, MESH:D015470	Arsenic Tr	23.27	21
CD81	975	Leukemia, MESH:D015470	Benzoates	23.27	18
CELF2	10659	Leukemia, MESH:D015470	Dexameth	23.27	25
CLDN11	5010	Leukemia, MESH:D015470	Arsenic Tr	23.27	16
DHRS7	51635	Leukemia, MESH:D015470	Arsenic Tr	23.27	26
HOPX	84525	Leukemia, MESH:D015470	Arsenic Tr	23.27	22
IL13RA2	3598	Leukemia, MESH:D015473	Arsenic|De	23.27	158
MACF1	23499	Leukemia, MESH:D015470	Arsenic Tr	23.27	32
NME2	4831	Leukemia, MESH:D015470	Alitretinoi	23.27	25
PDE2A	5138	Leukemia, MESH:D015470	Arsenic Tr	23.27	27
PDE4DIP	9659	Leukemia, MESH:D015470	Arsenic Tr	23.27	27
RFC5	5985	Leukemia, MESH:D015473	arsenite|D	23.27	161
SIAH2	6478	Leukemia, MESH:D015470	Doxorubic	23.27	21
SLC4A7	9497	Leukemia, MESH:D015470	Calcitriol|D	23.27	14
YBX3	8531	Leukemia, MESH:D015473	arsenite|ca	23.27	157
HSD17B2	3294	Leukemia, MESH:D015470	Arsenic Tr	23.26	21
MAFB	9935	Leukemia, MESH:D015470	Calcitriol|D	23.26	19
CYP3A11	13112	Leukemia, MESH:D015473	alpha-Toc	23.25	158
CHRM3	1131	Leukemia, MESH:D015473	Arsenic|Ar	23.23	267
CYP2E1	1571	Leukemia, MESH:D015470	Androgen	23.23	44
MCM7	4176	Leukemia, MESH:D015473	Arsenic Tr	23.23	262
BCL2	596	Leukemia, MESH:D004915	Cytarabine	23.22	4
A2M	2	Leukemia, MESH:D015470	Arsenic Tr	23.21	23
MIR106A	406899	Leukemia, MESH:D015473	Arsenic|De	23.21	158
ABCB9	23457	Leukemia, MESH:D015470	Dexameth	23.2	16
ALDH1A2	8854	Leukemia, MESH:D015470	Bezafibrat	23.2	19
AMD1	262	Leukemia, MESH:D015473	Arsenic|Ar	23.2	268
CPZ	8532	Leukemia, MESH:D015470	Calcitriol|C	23.2	78
ERP27	121506	Leukemia, MESH:D015470	Air Polluta	23.2	11
FBXO3	26273	Leukemia, MESH:D015470	Benzene|C	23.2	29
GALNT16	57452	Leukemia, MESH:D015470	Dexameth	23.2	15
IL27RA	9466	Leukemia, MESH:D015470	Arsenic Tr	23.2	27
LARP6	55323	Leukemia, MESH:D015470	Decitabine	23.2	26
LPCAT2	54947	Leukemia, MESH:D015470	Dexameth	23.2	15
MFAP3L	9848	Leukemia, MESH:D015470	Air Polluta	23.2	13
MPP7	143098	Leukemia, MESH:D015470	Calcitriol|D	23.2	14
ND4	4538	Leukemia, MESH:D015470	Arsenic Tr	23.2	29
NIFK	84365	Leukemia, MESH:D015470	Arsenic Tr	23.2	22
NOD2	64127	Leukemia, MESH:D015470	Calcitriol|C	23.2	11
NPTN	27020	Leukemia, MESH:D015470	Arsenic Tr	23.2	22
PHACTR2	9749	Leukemia, MESH:D015470	Azacitidine	23.2	82
PLLP	51090	Leukemia, MESH:D015470	Arsenic Tr	23.2	87
PTPN3	5774	Leukemia, MESH:D015473	arsenite|Ca	23.2	160
TANC1	85461	Leukemia, MESH:D015470	Arsenic Tr	23.2	21
TCP11L2	255394	Leukemia, MESH:D015473	arsenite|D	23.2	157
TMEM45A	55076	Leukemia, MESH:D015470	Cytarabine	23.2	82
TRIM13	10206	Leukemia, MESH:D015470	Calcitriol|D	23.2	15
TRIM27	5987	Leukemia, MESH:D015470	Air Polluta	23.2	22
ZBTB21	49854	Leukemia, MESH:D015470	Air Polluta	23.2	21
ABCC9	10060	Leukemia, MESH:D015470	Dexameth	23.19	17
CEP55	55165	Leukemia, MESH:D015470	Arsenic Tr	23.19	22
DTYMK	1841	Leukemia, MESH:D015473	arsenite|Ca	23.19	158
GABARAP	23766	Leukemia, MESH:D015470	Azacitidine	23.19	83
IFITM2	10581	Leukemia, MESH:D015470	Benzene|D	23.19	34
KDM3A	55818	Leukemia, MESH:D015470	Arsenic Tr	23.19	22
LRP5	4041	Leukemia, MESH:D015470	Androgen	23.19	17
RPL17	6139	Leukemia, MESH:D015470	Arsenic Tr	23.19	26

SCN5A	6331	Leukemia, MESH:D015470	Crizotinib|	23.19	55
DES	1674	Leukemia, MESH:D015473	Arsenic|Da	23.18	163
IL23A	51561	Leukemia, MESH:D015473	Arsenic|Ar	23.18	163
PPARD	5467	Leukemia, MESH:D015473	arsenite|Ca	23.18	162
TNNT2	7139	Leukemia, MESH:D015473	Arsenic|Ar	23.18	271
ATF4	468	Leukemia, MESH:D015473	2-(2-chlor	23.17	264
IL1R1	3554	Leukemia, MESH:D015473	Arsenic|Ca	23.17	160
TMSB15A	11013	Leukemia, MESH:D015473	Calcitriol|D	23.15	156
ACACB	32	Leukemia, MESH:D015470	Bezafibrat	23.14	18
CHMP1A	5119	Leukemia, MESH:D015470	Arsenic Tr	23.14	28
EMC8	10328	Leukemia, MESH:D015470	Decitabine	23.14	21
FABP4	2167	Leukemia, MESH:D015473	Arsenic|ars	23.14	161
MIR10B	406903	Leukemia, MESH:D015470	Air Polluta	23.14	19
NDUFAF3	25915	Leukemia, MESH:D015470	Alitretinoi	23.14	14
PHACTR3	116154	Leukemia, MESH:D015470	Dexameth	23.14	11
AASDH	132949	Leukemia, MESH:D015470	Dronabino	23.13	10
ADIRF	10974	Leukemia, MESH:D015470	Dexameth	23.13	16
BTN3A2	11118	Leukemia, MESH:D015470	Arsenic Tr	23.13	19
FAM216A	29902	Leukemia, MESH:D015470	Calcitriol|D	23.13	9
GPR135	64582	Leukemia, MESH:D015470	Dexameth	23.13	10
HCP5	10866	Leukemia, MESH:D015470	Decitabine	23.13	21
HES4	57801	Leukemia, MESH:D015470	Decitabine	23.13	19
MEAF6	64769	Leukemia, MESH:D015470	Dexameth	23.13	15
NUP43	348995	Leukemia, MESH:D015470	Air Polluta	23.13	14
PRDM4	11108	Leukemia, MESH:D015470	Dexameth	23.13	13
SEMA6C	10500	Leukemia, MESH:D015470	Azacitidine	23.13	15
ZFP42	132625	Leukemia, MESH:D015470	Arsenic Tr	23.13	14
COMTD1	118881	Leukemia, MESH:D015473	Arsenic|De	23.12	159
CYP2S1	29785	Leukemia, MESH:D015473	Calcitriol|D	23.12	154
ESM1	11082	Leukemia, MESH:D015473	Arsenic|Ch	23.12	157
KNTC1	9735	Leukemia, MESH:D015473	Arsenic|ars	23.12	160
LAMA2	3908	Leukemia, MESH:D015473	arsenite|Ca	23.12	157
PRIM2	5558	Leukemia, MESH:D015473	Buthionine	23.12	156
HILPDA	29923	Leukemia, MESH:D015470	Arsenic Tr	23.1	27
PTN	5764	Leukemia, MESH:D015470	Calcitriol|D	23.1	16
RBPMS	11030	Leukemia, MESH:D015470	Arsenic Tr	23.1	21
RPLP0	6175	Leukemia, MESH:D015470	Arsenic Tr	23.1	24
SP1	6667	Leukemia, MESH:D015473	Arsenic|Ar	23.1	265
TBXAS1	6916	Leukemia, MESH:D015470	Benzene|C	23.1	26
CUL1	8454	Leukemia, MESH:D015470	Arsenic Tr	23.09	21
GOLM1	51280	Leukemia, MESH:D015470	Calcitriol|D	23.09	11
HLA-DQB	3119	Leukemia, MESH:D015470	Air Polluta	23.09	15
IFI16	3428	Leukemia, MESH:D015470	Air Polluta	23.09	20
KCNS3	3790	Leukemia, MESH:D015470	Arsenic Tr	23.09	16
NADK2	133686	Leukemia, MESH:D015470	Bortezomi	23.09	17
NUP155	9631	Leukemia, MESH:D015470	Calcitriol|D	23.09	17
SALL4	57167	Leukemia, MESH:D015470	Azacitidine	23.09	13
SMS	6611	Leukemia, MESH:D015470	Arsenic Tr	23.09	19
STAT5	1E+08	Leukemia, MESH:D015470	Arsenic Tr	23.09	13
TCF7L1	83439	Leukemia, MESH:D015470	Arsenic Tr	23.09	19
TDG	6996	Leukemia, MESH:D015470	Bezafibrat	23.09	15
UBE2J1	51465	Leukemia, MESH:D015470	Dexameth	23.09	13
SLC27A2	11001	Leukemia, MESH:D015473	Arsenic Tr	23.08	262
MAFG	4097	Leukemia, MESH:D015470	Benzene|B	23.07	32
NFATC1	4772	Leukemia, MESH:D015470	Azacitidine	23.07	41
DCBLD2	131566	Leukemia, MESH:D015473	Arsenic|Ar	23.05	264
GCH2	64263	Leukemia, MESH:D015470	Dexameth	23.05	9
NCAPG2	54892	Leukemia, MESH:D015473	arsenite|Ca	23.05	157

TRIM24	8805	Leukemia, MESH:D015473	Arsenic|Ar	23.05	264
AQP1	358	Leukemia, MESH:D004915	Cytarabine	23.04	4
MCPT1	17224	Leukemia, MESH:D015473	Calcitriol|(	23.04	7
MSI1	4440	Leukemia, MESH:D015473	Arsenic Tr	23.04	264
ZNF780B	163131	Leukemia, MESH:D015470	Air Polluta	23.04	13
AIFM1	9131	Leukemia, MESH:D007948	2-(2-amin	23.03	3
SNRPB2	6629	Leukemia, MESH:D015473	arsenite|D	23.03	157
SRC	6714	Leukemia, MESH:D015473	Arsenic|Ar	23.03	160
TMBIM4	51643	Leukemia, MESH:D015473	Arsenic Tr	23.03	262
ANKRD12	23253	Leukemia, MESH:D015470	Arsenic Tr	23.02	17
ANKRD37	353322	Leukemia, MESH:D015470	Calcitriol|D	23.02	14
GALE	2582	Leukemia, MESH:D015470	Arsenic Tr	23.02	19
NR1H2	7376	Leukemia, MESH:D015470	Alitretinoi	23.02	25
TRAP1	10131	Leukemia, MESH:D015470	Arsenic Tr	23.02	22
VAV3	10451	Leukemia, MESH:D015470	Arsenic Tr	23.02	20
CA2	760	Leukemia, MESH:D015470	Alitretinoi	23.01	16
PYCARD	29108	Leukemia, MESH:D015470	Allopurinc	23.01	27
SLC7A1	6541	Leukemia, MESH:D015470	Androgen	23.01	22
UBC	7316	Leukemia, MESH:D015470	Arsenic Tr	23.01	26
RUNX2	860	Leukemia, MESH:D007948	2-(2-amin	23	3
COL18A1	80781	Leukemia, MESH:D015473	Arsenic|ca	22.99	161
BAG2	9532	Leukemia, MESH:D015473	arsenite|C	22.98	158
HSPA1A	3303	Leukemia, MESH:D015473	Antimony	22.98	267
PBX1	5087	Leukemia, MESH:D015473	Antimony	22.98	157
PTGIS	5740	Leukemia, MESH:D015473	Calcitriol|D	22.98	154
SUMO1	7341	Leukemia, MESH:D015473	Antimony	22.98	261
TPM3	7170	Leukemia, MESH:D015473	Arsenic|Ar	22.98	266
ARSG	22901	Leukemia, MESH:D015470	Dexameth	22.97	15
CD3G	917	Leukemia, MESH:D015470	Benzene|C	22.97	24
CLDN9	9080	Leukemia, MESH:D015470	Arsenic Tr	22.97	18
CLSTN2	64084	Leukemia, MESH:D015470	Dexameth	22.97	15
CSPP1	79848	Leukemia, MESH:D015470	Arsenic Tr	22.97	20
DDB1	1642	Leukemia, MESH:D015470	Arsenic Tr	22.97	20
EMG1	10436	Leukemia, MESH:D015470	Decitabine	22.97	17
EPHB6	2051	Leukemia, MESH:D015470	Calcitriol|D	22.97	23
ERBB4	2066	Leukemia, MESH:D015473	Arsenic|Ar	22.97	264
GSTCD	79807	Leukemia, MESH:D015470	Bortezomi	22.97	81
HCFC1	3054	Leukemia, MESH:D015470	Arsenic Tr	22.97	22
ITPKB	3707	Leukemia, MESH:D015470	Doxorubic	22.97	17
MEGF6	1953	Leukemia, MESH:D015470	Arsenic Tr	22.97	16
MLPH	79083	Leukemia, MESH:D015470	Dexameth	22.97	15
NBR1	4077	Leukemia, MESH:D015470	Air Polluta	22.97	18
OLAH	55301	Leukemia, MESH:D015470	Calcitriol|D	22.97	13
PLEKHO2	80301	Leukemia, MESH:D015470	Benzene|C	22.97	24
RGMA	56963	Leukemia, MESH:D015470	Dexameth	22.97	15
RHO	6010	Leukemia, MESH:D015470	Alitretinoi	22.97	13
SCO2	9997	Leukemia, MESH:D015470	Arsenic Tr	22.97	18
TMEM185	79134	Leukemia, MESH:D015470	Doxorubic	22.97	22
UBAP2L	9898	Leukemia, MESH:D015470	Arsenic Tr	22.97	21
UQCRQ	27089	Leukemia, MESH:D015470	Arsenic Tr	22.97	21
USP25	29761	Leukemia, MESH:D015470	Chloroqui	22.97	13
VPS25	84313	Leukemia, MESH:D015470	Dexameth	22.97	12
COMMD6	170622	Leukemia, MESH:D015470	Dexameth	22.96	14
DCAF4	26094	Leukemia, MESH:D015470	Doxorubic	22.96	23
INTS3	65123	Leukemia, MESH:D015470	Dexameth	22.96	14
LMF2	91289	Leukemia, MESH:D015470	Gasoline|(	22.96	10
LNPK	80856	Leukemia, MESH:D015470	Dexameth	22.96	11
PLXNA1	5361	Leukemia, MESH:D015470	Dexameth	22.96	14

TMEM199	147007	Leukemia, MESH:D015470	Dexameth	22.96	14
TXN	7295	Leukemia, MESH:D015473	Antimony	22.96	266
USP42	84132	Leukemia, MESH:D015470	Dexameth	22.96	13
CRYL1	51084	Leukemia, MESH:D015470	Benzene|[illegible]	22.94	27
CTNND1	1500	Leukemia, MESH:D015470	Arsenic Tr	22.94	28
ENDOG	2021	Leukemia, MESH:D015470	Dexameth	22.94	18
FOXM1	2305	Leukemia, MESH:D015473	Arsenic Tr	22.94	264
LRP1	4035	Leukemia, MESH:D015473	Arsenic|Ar	22.94	161
PPRC1	23082	Leukemia, MESH:D015470	Arsenic Tr	22.94	18
PSMD12	5718	Leukemia, MESH:D015470	Arsenic Tr	22.94	41
E2F1	1869	Leukemia, MESH:D015473	alvocidib|[illegible]	22.93	266
ALDH3B2	222	Leukemia, MESH:D015473	Antimony	22.92	14
CYP11A1	1583	Leukemia, MESH:D015473	Arsenic|Ar	22.92	265
HMGB1	3146	Leukemia, MESH:D007948	Arsenic Tr	22.92	4
ANK2	287	Leukemia, MESH:D015473	Arsenic|ar[illegible]	22.91	163
CEP135	9662	Leukemia, MESH:D015473	arsenite|D	22.91	159
CYP3A23-	25642	Leukemia, MESH:D015470	Benzene|[illegible]	22.91	30
ERCC8	1161	Leukemia, MESH:D015473	Arsenic Tr	22.91	264
H1-5	3009	Leukemia, MESH:D015473	Calcitriol|[illegible]	22.91	153
PRR16	51334	Leukemia, MESH:D015473	Arsenic|Ar	22.91	264
ALDH4A1	8659	Leukemia, MESH:D015473	Arsenic|Ar	22.9	265
CSRP3	8048	Leukemia, MESH:D015473	Arsenic Tr	22.9	261
FOXRED2	80020	Leukemia, MESH:D015473	Arsenic Tr	22.9	261
PIK3CB	5291	Leukemia, MESH:D015473	Arsenic Tr	22.9	267
SLC2A1	6513	Leukemia, MESH:D015473	Arsenic|Ar	22.9	270
TOMM34	10953	Leukemia, MESH:D015473	Arsenic|Ar	22.9	264
PTP4A1	7803	Leukemia, MESH:D015470	Calcitriol|[illegible]	22.88	17
S100B	6285	Leukemia, MESH:D015470	Arsenic Tr	22.88	22
SLC6A6	6533	Leukemia, MESH:D015470	Dasatinib|[illegible]	22.88	18
BOK	666	Leukemia, MESH:D015470	Azacitidin[illegible]	22.86	18
CD302	9936	Leukemia, MESH:D015470	Dasatinib|[illegible]	22.86	16
CKLF	51192	Leukemia, MESH:D015470	Calcitriol|[illegible]	22.86	19
CMTM3	123920	Leukemia, MESH:D015470	Calcitriol|[illegible]	22.86	10
COL11A2	1302	Leukemia, MESH:D015470	Dexameth	22.86	15
CYB561	1534	Leukemia, MESH:D015470	Dexameth	22.86	18
DUSP8	1850	Leukemia, MESH:D015470	Air Polluta	22.86	23
FARSA	2193	Leukemia, MESH:D015470	Bortezomi	22.86	15
HBA-A1	15122	Leukemia, MESH:D015470	Benzene|[illegible]	22.86	31
HMOX2	3163	Leukemia, MESH:D015470	Arsenic Tr	22.86	16
MYBPC3	4607	Leukemia, MESH:D015470	Dexameth	22.86	15
NCF1	653361	Leukemia, MESH:D015473	Arsenic|Ar	22.86	272
OLA1	29789	Leukemia, MESH:D015470	Decitabin[illegible]	22.86	21
OPTN	10133	Leukemia, MESH:D015470	Arsenic Tr	22.86	26
PDXK	8566	Leukemia, MESH:D015470	Air Polluta	22.86	29
PLA2G4C	8605	Leukemia, MESH:D015470	Dexameth	22.86	19
PLIN5	440503	Leukemia, MESH:D015470	Dexameth	22.86	11
PNRC1	10957	Leukemia, MESH:D015470	Arsenic Tr	22.86	22
RASSF4	83937	Leukemia, MESH:D015470	Dexameth	22.86	16
SCLY	51540	Leukemia, MESH:D015470	Arsenic Tr	22.86	26
SDHC	6391	Leukemia, MESH:D015470	Arsenic Tr	22.86	21
SLC15A3	51296	Leukemia, MESH:D015470	Benzene|[illegible]	22.86	30
SPOCK1	6695	Leukemia, MESH:D015470	Dexameth	22.86	15
SRGAP3	9901	Leukemia, MESH:D015470	Cytarabin[illegible]	22.86	79
TBC1D8	11138	Leukemia, MESH:D015470	Calcitriol|[illegible]	22.86	26
TRAF2	7186	Leukemia, MESH:D015470	Arsenic Tr	22.86	47
TTC28	23331	Leukemia, MESH:D015470	Arsenic Tr	22.86	22
CD80	941	Leukemia, MESH:D015473	Arsenic|Ar	22.84	266
CLDN4	1364	Leukemia, MESH:D015473	Arsenic|Ar	22.84	266

MAP3K5	4217	Leukemia, MESH:D004915		Daunorub	22.84	3
SLC20A1	6574	Leukemia, MESH:D015473		Arsenic Tr	22.84	261
KRT15	3866	Leukemia, MESH:D015473		Arsenic|ar	22.83	160
RAD51C	5889	Leukemia, MESH:D015473		Arsenic Tr	22.83	264
ZMYND8	23613	Leukemia, MESH:D015473		Arsenic Tr	22.83	263
HSPA1B	3304	Leukemia, MESH:D015473		alpha-Toc	22.82	163
NPPB	4879	Leukemia, MESH:D015473		Calcitriol|C	22.82	164
SRRT	51593	Leukemia, MESH:D007948		Arsenic Tr	22.82	3
ANKDD1A	348094	Leukemia, MESH:D015470		Benzene|D	22.8	32
ANKRD13	124930	Leukemia, MESH:D015470		Dexameth	22.8	14
AZIN2	113451	Leukemia, MESH:D015470		Decitabine	22.8	20
CCDC93	54520	Leukemia, MESH:D015470		Calcitriol|D	22.8	14
CSTF2T	23283	Leukemia, MESH:D015470		Doxorubic	22.8	17
ELFN1	392617	Leukemia, MESH:D015470		Dexameth	22.8	16
FANCE	2178	Leukemia, MESH:D015470		Calcitriol|D	22.8	8
GPATCH8	23131	Leukemia, MESH:D015470		Cyclophos	22.8	14
JKAMP	51528	Leukemia, MESH:D015470		Arsenic Tr	22.8	19
MAST3	23031	Leukemia, MESH:D015470		Dexameth	22.8	14
MICU3	286097	Leukemia, MESH:D015470		Arsenic Tr	22.8	20
MTHFD2	10797	Leukemia, MESH:D015473		Arsenic Tr	22.8	263
NECAP1	25977	Leukemia, MESH:D015470		Dexameth	22.8	9
NREP	9315	Leukemia, MESH:D015473		Arsenic|Ar	22.8	263
PHB	18673	Leukemia, MESH:D015470		Busulfan|D	22.8	10
PM20D2	135293	Leukemia, MESH:D015470		Bortezomi	22.8	18
RFESD	317671	Leukemia, MESH:D015470		Decitabine	22.8	15
RNF5	6048	Leukemia, MESH:D015470		Alitretinoi	22.8	14
SMCR8	140775	Leukemia, MESH:D015470		Dexameth	22.8	16
SNHG7	84973	Leukemia, MESH:D015470		Dexameth	22.8	13
ZNF165	7718	Leukemia, MESH:D015470		Bortezomi	22.8	10
PRDM8	56978	Leukemia, MESH:D015473		Arsenic Tr	22.79	265
ROBO3	64221	Leukemia, MESH:D015473		Arsenic|Cy	22.79	158
SERPINE2	5270	Leukemia, MESH:D015473		arsenite|C	22.79	156
CAD	790	Leukemia, MESH:D015470		Arsenic Tr	22.78	18
CARM1	10498	Leukemia, MESH:D015473		Arsenic Tr	22.78	262
CLDN2	9075	Leukemia, MESH:D015470		Chloroqui	22.78	23
EEF1A2	1917	Leukemia, MESH:D015470		Arsenic Tr	22.78	28
IRF3	3661	Leukemia, MESH:D015470		Arsenic Tr	22.78	18
KL	9365	Leukemia, MESH:D015470		Arsenic Tr	22.78	24
MANF	7873	Leukemia, MESH:D015470		Arsenic Tr	22.78	23
NNMT	4837	Leukemia, MESH:D015470		Dasatinib|	22.78	17
PLN	5350	Leukemia, MESH:D015470		Allopurino	22.78	19
SLC26A3	1811	Leukemia, MESH:D015473		Calcitriol|D	22.78	154
SPSB1	80176	Leukemia, MESH:D015470		Dexameth	22.78	16
NPIPB3	23117	Leukemia, MESH:D015470		Dexameth	22.77	12
PLAU	5328	Leukemia, MESH:D007948		2-(2-amin	22.77	3
CAMK2N1	55450	Leukemia, MESH:D015473		arsenite|Cy	22.76	161
LRP8	7804	Leukemia, MESH:D015473		Arsenic|De	22.76	159
MMP12	4321	Leukemia, MESH:D015470		Air Polluta	22.76	25
PLP1	5354	Leukemia, MESH:D015473		Arsenic|Ar	22.76	266
POTEF	728378	Leukemia, MESH:D015470		Dexameth	22.76	14
SDCBP	6386	Leukemia, MESH:D015473		Arsenic|Ar	22.76	265
ZNF317	57693	Leukemia, MESH:D015470		Dexameth	22.76	14
EHD3	30845	Leukemia, MESH:D01	marker/m	Decitabine	22.75	24
ABCA2	20	Leukemia, MESH:D015470		Bortezomi	22.75	18
ASIC1	41	Leukemia, MESH:D015470		Arsenic Tr	22.75	15
CDC42EP4	23580	Leukemia, MESH:D015470		Decitabine	22.75	21
EFEMP2	30008	Leukemia, MESH:D015470		Calcitriol|C	22.75	88
ITM2A	9452	Leukemia, MESH:D015470		Arsenic Tr	22.75	22

LEF1	51176	Leukemia, MESH:D015473	Arsenic|ars	22.75	161
MASTL	84930	Leukemia, MESH:D015470	Dasatinib|I	22.75	16
NDUFA3	4696	Leukemia, MESH:D015470	Dexameth	22.75	16
NPTX2	4885	Leukemia, MESH:D015470	Arsenic Tr	22.75	22
NSG1	27065	Leukemia, MESH:D015470	Benzene|D	22.75	31
PARM1	25849	Leukemia, MESH:D015470	Cytarabine	22.75	83
PBX3	5090	Leukemia, MESH:D015470	Bortezomi	22.75	16
PPP1R2	5504	Leukemia, MESH:D015470	Dexameth	22.75	23
SLC16A12	387700	Leukemia, MESH:D015470	Dexameth	22.75	15
USP10	9100	Leukemia, MESH:D015470	Dexameth	22.75	13
GLUL	2752	Leukemia, MESH:D015473	arsenic dis	22.72	265
CSF2	1437	Leukemia, MESH:D015473	Arsenic|Ar	22.71	266
CTF1	1489	Leukemia, MESH:D007948	Arsenic Tr	22.71	3
DYNC1H1	1778	Leukemia, MESH:D015470	Arsenic Tr	22.7	36
MSR1	4481	Leukemia, MESH:D015470	Arsenic Tr	22.7	25
PGM1	5236	Leukemia, MESH:D015470	Arsenic Tr	22.7	24
PRMT1	3276	Leukemia, MESH:D015470	Arsenic Tr	22.7	22
PSMA1	5682	Leukemia, MESH:D015470	Arsenic Tr	22.7	21
SH3KBP1	30011	Leukemia, MESH:D015470	Calcitriol|D	22.7	15
SLC19A2	10560	Leukemia, MESH:D015470	Dexameth	22.7	21
SMC2	10592	Leukemia, MESH:D015470	Benzene|C	22.7	32
ATP6V1A	523	Leukemia, MESH:D015473	Arsenic Tr	22.69	265
CEBPA	1050	Leukemia, MESH:D007948	2-(2-amin	22.69	3
MGMT	4255	Leukemia, MESH:D015473	Arsenic|ars	22.69	18
SYNM	23336	Leukemia, MESH:D015473	Arsenic|ars	22.69	158
TNFRSF10	8795	Leukemia, MESH:D007948	2-(2-amin	22.69	4
RBM34	23029	Leukemia, MESH:D015473	Cytarabine	22.67	158
ST6GALN/	81849	Leukemia, MESH:D015473	Arsenic|ars	22.67	157
TNS4	84951	Leukemia, MESH:D015473	Arsenic Tr	22.67	263
FGF10	2255	Leukemia, MESH:D007948	2-(2-amin	22.66	3
MIF	4282	Leukemia, MESH:D015470	Arsenic Tr	22.66	24
P4HB	5034	Leukemia, MESH:D015473	Arsenic|Ar	22.66	267
PTPN1	5770	Leukemia, MESH:D015473	Arsenic Tr	22.66	156
RNF150	57484	Leukemia, MESH:D015473	arsenite|D	22.66	155
CLPX	10845	Leukemia, MESH:D015470	Air Polluta	22.65	17
FLT4	2324	Leukemia, MESH:D015470	Antifungal	22.65	25
RGS17	26575	Leukemia, MESH:D015470	Calcitriol|D	22.65	13
STIL	6491	Leukemia, MESH:D015470	Calcitriol|D	22.65	14
AP1G1	164	Leukemia, MESH:D015470	Calcitriol|D	22.64	12
APCDD1	147495	Leukemia, MESH:D015470	Calcitriol|D	22.64	15
ATG7	10533	Leukemia, MESH:D015470	Air Polluta	22.64	23
AUTS2	26053	Leukemia, MESH:D015470	Arsenic Tr	22.64	35
CHTF18	63922	Leukemia, MESH:D015470	Arsenic Tr	22.64	19
COX7A2	1347	Leukemia, MESH:D015470	Cyclophos	22.64	16
DNASE1L1	1774	Leukemia, MESH:D015470	Arsenic Tr	22.64	19
ELP5	23587	Leukemia, MESH:D015470	Arsenic Tr	22.64	20
FUT11	170384	Leukemia, MESH:D015470	Arsenic Tr	22.64	22
GALC	2581	Leukemia, MESH:D015470	Cyclophos	22.64	16
GRAMD2E	65983	Leukemia, MESH:D015470	Arsenic Tr	22.64	23
HECTD4	283450	Leukemia, MESH:D015470	Dexameth	22.64	12
ITGA6	3655	Leukemia, MESH:D015470	Arsenic Tr	22.64	24
ITPRIP	85450	Leukemia, MESH:D015470	Dexameth	22.64	15
LHFPL6	10186	Leukemia, MESH:D015470	Arsenic Tr	22.64	87
MAP3K4	4216	Leukemia, MESH:D015470	Doxorubic	22.64	17
MIR22HG	84981	Leukemia, MESH:D015470	Benzene|C	22.64	92
RFC2	5982	Leukemia, MESH:D015470	Arsenic Tr	22.64	18
RNF167	26001	Leukemia, MESH:D015470	Arsenic Tr	22.64	15
RNF31	55072	Leukemia, MESH:D015470	Benzene|D	22.64	27

SLC39A13	91252	Leukemia, MESH:D015470	Arsenic Tr	22.64	19
SNRPA	6626	Leukemia, MESH:D015470	Arsenic Tr	22.64	22
SNTB1	6641	Leukemia, MESH:D015470	Calcitriol|D	22.64	15
STIM2	57620	Leukemia, MESH:D015470	Bortezomi	22.64	14
TDP1	55775	Leukemia, MESH:D015470	Arsenic Tr	22.64	17
TESK1	7016	Leukemia, MESH:D015470	Benzene|D	22.64	28
CDKN2D	1032	Leukemia, MESH:D015473	Arsenic|Ar	22.63	265
GLT8D2	83468	Leukemia, MESH:D015470	Calcitriol|D	22.63	14
IST1	9798	Leukemia, MESH:D015470	Dexameth	22.63	14
MXI1	4601	Leukemia, MESH:D015473	arsenite|ca	22.63	156
PITPNM2	57605	Leukemia, MESH:D015470	Doxorubic	22.63	16
TIGD2	166815	Leukemia, MESH:D015470	Arsenic Tr	22.63	20
ZNF385B	151126	Leukemia, MESH:D015470	Decitabine	22.63	17
CCN1	3491	Leukemia, MESH:D015473	Arsenic|Ar	22.62	264
CCN5	8839	Leukemia, MESH:D015473	Arsenic Tr	22.62	157
CYP3A5	1577	Leukemia, MESH:D015473	2-(2-chlor	22.62	16
EPS8	2059	Leukemia, MESH:D015470	Dexameth	22.62	17
FAM83D	81610	Leukemia, MESH:D015473	Arsenic|ars	22.62	159
PCDH7	5099	Leukemia, MESH:D015473	arsenite|Ca	22.62	158
PLAGL1	5325	Leukemia, MESH:D015470	Decitabine	22.62	28
PTAFR	5724	Leukemia, MESH:D015470	Dexameth	22.62	14
PTMA	5757	Leukemia, MESH:D015470	Arsenic Tr	22.62	24
SDHB	6390	Leukemia, MESH:D015470	Arsenic Tr	22.62	22
SP7	121340	Leukemia, MESH:D015473	Arsenic Tr	22.62	261
STK17B	9262	Leukemia, MESH:D015473	Arsenic|Ar	22.62	263
TENT5C	54855	Leukemia, MESH:D015473	Arsenic Tr	22.62	267
ALAD	210	Leukemia, MESH:D015470	Arsenic Tr	22.61	49
ERRFI1	54206	Leukemia, MESH:D015473	Arsenic|Ar	22.61	267
F3	2152	Leukemia, MESH:D007948	2-(2-amin	22.61	3
NT5E	4907	Leukemia, MESH:D015473	Arsenic|Ar	22.61	266
PBK	55872	Leukemia, MESH:D015473	Calcitriol|D	22.61	155
BIRC5	332	Leukemia, MESH:D004915	Daunorub	22.6	4
ERCC1	2067	Leukemia, MESH:D015470	Benzene|C	22.58	36
RBP1	5947	Leukemia, MESH:D015470	Alitretinoi	22.58	28
ABCC5	10057	Leukemia, MESH:D015473	Arsenic Tr	22.57	157
KRT23	25984	Leukemia, MESH:D015473	Calcitriol|C	22.56	154
PLCD1	5333	Leukemia, MESH:D007948	2-(2-amin	22.56	3
PPP1CB	5500	Leukemia, MESH:D015473	Arsenic Tr	22.56	266
VCAM1	7412	Leukemia, MESH:D004915	Cytarabine	22.56	3
ADAMTS5	11096	Leukemia, MESH:D015473	Arsenic|Ar	22.55	263
ATP6V1E1	529	Leukemia, MESH:D015473	Arsenic|Ar	22.55	265
GCSHB	436852	Leukemia, MESH:D015470	Dexameth	22.55	6
MRPS17	51373	Leukemia, MESH:D015473	Arsenic|ars	22.55	160
RAB37	326624	Leukemia, MESH:D015473	Arsenic|ars	22.55	160
VSNL1	7447	Leukemia, MESH:D015473	Arsenic|Ar	22.55	266
ACER2	340485	Leukemia, MESH:D015470	Doxorubic	22.54	23
ADI1	55256	Leukemia, MESH:D015470	Decitabine	22.54	21
AQP5	362	Leukemia, MESH:D015470	Azacitidine	22.54	10
CCAR1	55749	Leukemia, MESH:D015470	Bortezomi	22.54	19
CREB3L2	64764	Leukemia, MESH:D015470	Arsenic Tr	22.54	19
DYRK1A	1859	Leukemia, MESH:D015470	Air Polluta	22.54	19
EDN1	1906	Leukemia, MESH:D004915	Cytarabine	22.54	3
HR	55806	Leukemia, MESH:D015470	Calcitriol|D	22.54	16
ITGAL	3683	Leukemia, MESH:D015470	Bortezomi	22.54	16
LARP1	23367	Leukemia, MESH:D015470	Dexameth	22.54	15
PGPEP1	54858	Leukemia, MESH:D015470	Calcitriol|I	22.54	12
PHTF2	57157	Leukemia, MESH:D015470	Benzoates	22.54	18
PNPLA3	80339	Leukemia, MESH:D015470	Dexameth	22.54	14

SAMD9	54809	Leukemia, MESH:D015470		Air Polluta	22.54	16
TRAFD1	10906	Leukemia, MESH:D015470		Bortezomi	22.54	16
VEPH1	79674	Leukemia, MESH:D015470		Arsenic Tr	22.54	21
KITLG	4254	Leukemia, MESH:D015473		Arsenic Tr	22.52	263
NGFR	4804	Leukemia, MESH:D015473		caffeic aci	22.52	156
NR4A3	8013	Leukemia, MESH:D015473		alpha-Toc	22.52	158
RND3	390	Leukemia, MESH:D015470		Arsenic Tr	22.52	21
SERPINA1	5265	Leukemia, MESH:D015473		Arsenic Tr	22.52	265
PLAT	5327	Leukemia, MESH:D007948		Arsenic Tr	22.5	4
DHFR2	200895	Leukemia, MESH:D015470		Dexameth	22.49	7
FRZB	2487	Leukemia, MESH:D015473		Arsenic|Ca	22.49	160
FUBP1	8880	Leukemia, MESH:D015473		Arsenic|Ar	22.49	266
G3BP1	10146	Leukemia, MESH:D015473		arsenite|D	22.49	158
GPKOW	27238	Leukemia, MESH:D015470		Arsenic Tr	22.49	22
MKNK2	2872	Leukemia, MESH:D015473		Arsenic|Ar	22.49	161
MPL	4352	Leukemia, MESH:D015470		Arsenic Tr	22.49	35
ZNF516	9658	Leukemia, MESH:D015470		Arsenic Tr	22.49	19
ANKMY2	57037	Leukemia, MESH:D015470		Calcitriol|D	22.48	15
BAHCC1	57597	Leukemia, MESH:D015470		Dexameth	22.48	9
CEP19	84984	Leukemia, MESH:D015470		Dexameth	22.48	8
CHMP4B	128866	Leukemia, MESH:D015470		Bezafibrat	22.48	10
DAAM2	23500	Leukemia, MESH:D015470		Dexameth	22.48	9
HNRNPUL	221092	Leukemia, MESH:D015470		Dexameth	22.48	13
INTS2	57508	Leukemia, MESH:D015470		Doxorubic	22.48	15
MDFI	4188	Leukemia, MESH:D015470		Arsenic Tr	22.48	20
MIER3	166968	Leukemia, MESH:D015470		Doxorubic	22.48	15
ORAI2	80228	Leukemia, MESH:D015470		Benzene|B	22.48	29
POLR2J2	246721	Leukemia, MESH:D015473		Antimony	22.48	155
RTEL1	51750	Leukemia, MESH:D015470		Cyclophos	22.48	16
SOX1	6656	Leukemia, MESH:D015470		Arsenic Tr	22.48	16
SPRYD7	57213	Leukemia, MESH:D015470		Arsenic Tr	22.48	20
TAPBPL	55080	Leukemia, MESH:D015470		Dexameth	22.48	14
TFCP2	7024	Leukemia, MESH:D015470		Dexameth	22.48	15
TMEM59	9528	Leukemia, MESH:D015470		Dexameth	22.48	14
TNFAIP8L3	388121	Leukemia, MESH:D015470		Alitretinoi	22.48	15
TRAPPC10	7109	Leukemia, MESH:D015470		Dexameth	22.48	11
VPS72	6944	Leukemia, MESH:D015470		Air Polluta	22.48	16
BCLAF1	9774	Leukemia, MESH:D015470		Dexameth	22.47	20
BHLHE41	79365	Leukemia, MESH:D015470		Air Polluta	22.47	19
COL5A1	1289	Leukemia, MESH:D015473		Arsenic|ars	22.47	163
ETV5	2119	Leukemia, MESH:D015470		Dexameth	22.47	17
HHEX	3087	Leukemia, MESH:D015470		Air Polluta	22.47	27
KLK3	354	Leukemia, MESH:D015473		Antimony	22.47	162
LASP1	3927	Leukemia, MESH:D015470		Arsenic Tr	22.47	21
MCM4	4173	Leukemia, MESH:D015473		Arsenic Tr	22.47	265
MFN2	9927	Leukemia, MESH:D015473		Arsenic Tr	22.47	263
NRXN1	9378	Leukemia, MESH:D015470		Decitabine	22.47	25
PRLR	5618	Leukemia, MESH:D015473		arsenite|Ca	22.47	10
PSAP	5660	Leukemia, MESH:D015470		Bortezomi	22.47	19
SERPINB9	5272	Leukemia, MESH:D015470		Alitretinoi	22.47	81
SLC22A1	6580	Leukemia, MESH:D015470		Allopurinc	22.47	20
TUBB2A	7280	Leukemia, MESH:D01	marker/m	Bortezomi	22.46	26
SLC38A2	54407	Leukemia, MESH:D015470		Arsenic Tr	22.46	38
FASLG	356	Leukemia, MESH:D007948		2-(2-amin	22.45	3
MIR708	1E+08	Leukemia, MESH:D015473		arsenite|D	22.45	157
FCHO2	115548	Leukemia, MESH:D015473		Arsenic|ars	22.44	13
H1-3	3007	Leukemia, MESH:D015473		arsenite|G	22.44	158
PLCXD1	55344	Leukemia, MESH:D015473		Arsenic Tr	22.44	261

RAD52	5893	Leukemia, MESH:D015473		Arsenic Tr	22.44	158
SLBP	7884	Leukemia, MESH:D015470		Azacitidine	22.44	27
SPIN4	139886	Leukemia, MESH:D015473		Calcitriol|C	22.44	159
CD70	970	Leukemia, MESH:D015470		Arsenic Tr	22.43	27
DDX6	1656	Leukemia, MESH:D015470		Dexameth	22.43	16
FMN2	56776	Leukemia, MESH:D015473		Arsenic|Ar	22.43	265
HEY2	23493	Leukemia, MESH:D015470		Air Polluta	22.43	27
LPL	4023	Leukemia, MESH:D015473		Arsenic Tr	22.43	264
NRN1	51299	Leukemia, MESH:D015470		Azacitidine	22.43	14
NUMB	8650	Leukemia, MESH:D015470		Arsenic Tr	22.43	23
PLCB3	5331	Leukemia, MESH:D015470		Calcitriol|D	22.43	16
RPLP2	6181	Leukemia, MESH:D015470		Calcitriol|D	22.43	22
SCEL	8796	Leukemia, MESH:D015473		Arsenic|Ca	22.43	157
SELPLG	6404	Leukemia, MESH:D015470		Air Polluta	22.43	15
SEMA6A	57556	Leukemia, MESH:D015470		Doxorubic	22.43	15
SLC7A6	9057	Leukemia, MESH:D015470		Dexameth	22.43	17
UBR4	23352	Leukemia, MESH:D015470		Dexameth	22.43	13
XPOT	11260	Leukemia, MESH:D015470		Arsenic Tr	22.43	15
APAF1	317	Leukemia, MESH:D015473		Arsenic|Ar	22.42	264
DLC1	10395	Leukemia, MESH:D015473		Arsenic|Ar	22.42	263
HGF	3082	Leukemia, MESH:D015473		Arsenic|Ar	22.42	264
PDHA1	5160	Leukemia, MESH:D015473		Arsenic Tr	22.42	263
RAC1	5879	Leukemia, MESH:D015470		Arsenic Tr	22.42	26
SLIT3	6586	Leukemia, MESH:D015473		Arsenic|ars	22.42	163
KIF11	3832	Leukemia, MESH:D015470		Calcitriol|D	22.4	28
NRAS	4893	Leukemia, MESH:D01	marker/m	Carboplati	22.39	42
CAST	831	Leukemia, MESH:D015470		Doxorubic	22.39	25
COMT	1312	Leukemia, MESH:D015470		Calcitriol|C	22.39	24
HNMT	3176	Leukemia, MESH:D015470		Arsenic Tr	22.39	16
BECN1	8678	Leukemia, MESH:D007948		2-(2-amin	22.37	3
CYP2B6	1555	Leukemia, MESH:D015470		Alitretinoi	22.37	21
MMP1	4312	Leukemia, MESH:D007948		2-(2-amin	22.37	3
ME1	4199	Leukemia, MESH:D01	marker/m	Antimony	22.36	264
SLC4A7	9497	Leukemia, MESH:D015473		arsenite|Ca	22.36	160
TJP1	7082	Leukemia, MESH:D015473		Arsenic|Ar	22.36	266
ADRB2	154	Leukemia, MESH:D015470		Azacitidine	22.35	31
OXTR	5021	Leukemia, MESH:D015473		arsenite|Ca	22.35	158
PDE4DIP	9659	Leukemia, MESH:D015473		Arsenic|Ar	22.35	265
PPARA	5465	Leukemia, MESH:D015473		alpha-Toc	22.35	270
PTH1R	5745	Leukemia, MESH:D015473		Arsenic|Ca	22.35	160
USP7	7874	Leukemia, MESH:D015473		Arsenic|ars	22.35	23
FOXF2	2295	Leukemia, MESH:D015473		arsenite|Ca	22.34	12
PDCD4	27250	Leukemia, MESH:D015473		Arsenic|Ar	22.34	266
SC5D	6309	Leukemia, MESH:D015470		Benzene|D	22.34	27
BAIAP2L1	55971	Leukemia, MESH:D015470		Arsenic Tr	22.33	89
BCL2L13	23786	Leukemia, MESH:D015470		Azacitidine	22.33	12
CCDC86	79080	Leukemia, MESH:D015470		Arsenic Tr	22.33	21
CLYBL	171425	Leukemia, MESH:D015470		Daunorub	22.33	38
DCP2	167227	Leukemia, MESH:D015470		Arsenic Tr	22.33	18
EPSTI1	94240	Leukemia, MESH:D015470		Arsenic Tr	22.33	18
FAM83H	286077	Leukemia, MESH:D015470		Calcitriol|D	22.33	8
GBP4	115361	Leukemia, MESH:D015470		Air Polluta	22.33	82
GPATCH2	55668	Leukemia, MESH:D015470		Bortezomi	22.33	12
LAD1	3898	Leukemia, MESH:D015470		Decitabine	22.33	17
MFAP5	8076	Leukemia, MESH:D015470		Calcitriol|D	22.33	18
MYO1C	4641	Leukemia, MESH:D015470		Benzoates	22.33	14
NDUFB6	4712	Leukemia, MESH:D015470		Dexameth	22.33	15
PACSIN1	29993	Leukemia, MESH:D015473		Arsenic|Ca	22.33	156

RAB3D	9545	Leukemia, MESH:D015470	Dexameth	22.33	16
RPRD1A	55197	Leukemia, MESH:D015470	Doxorubic	22.33	18
SLC12A5	57468	Leukemia, MESH:D015470	Decitabine	22.33	16
SNX18	112574	Leukemia, MESH:D015470	Calcitriol|I	22.33	12
TBCD	6904	Leukemia, MESH:D015470	Bortezomi	22.33	16
TGDS	23483	Leukemia, MESH:D015470	Dronabinc	22.33	19
TINAGL1	64129	Leukemia, MESH:D015470	Calcitriol|D	22.33	11
AAMP	14	Leukemia, MESH:D015470	Air Polluta	22.32	20
AFG3L2	10939	Leukemia, MESH:D015470	Dexameth	22.32	14
CYP4V2	285440	Leukemia, MESH:D015470	Doxorubic	22.32	18
ENOX1	55068	Leukemia, MESH:D015470	Decitabine	22.32	22
LRP2	4036	Leukemia, MESH:D015470	Dronabinc	22.32	13
LY6K	54742	Leukemia, MESH:D015470	Dexameth	22.32	9
MIR143	406935	Leukemia, MESH:D015470	Air Polluta	22.32	20
MTMR2	8898	Leukemia, MESH:D015470	Arsenic Tr	22.32	21
N6AMT1	29104	Leukemia, MESH:D015470	Doxorubic	22.32	14
NDFIP2	54602	Leukemia, MESH:D015470	Decitabine	22.32	20
NLRP1	22861	Leukemia, MESH:D015470	Arsenic Tr	22.32	21
NUDT19	390916	Leukemia, MESH:D015470	Dexameth	22.32	14
PCDHGC3	5098	Leukemia, MESH:D015470	Doxorubic	22.32	16
RNF11	26994	Leukemia, MESH:D015470	Bortezomi	22.32	16
SLC44A5	204962	Leukemia, MESH:D015470	Calcitriol|D	22.32	17
SNAP91	9892	Leukemia, MESH:D015470	Cytarabine	22.32	87
TSPAN31	6302	Leukemia, MESH:D015473	Arsenic Tr	22.32	262
CACYBP	27101	Leukemia, MESH:D015470	Doxorubic	22.31	18
CCL22	6367	Leukemia, MESH:D015470	Allopurinc	22.31	22
CMBL	134147	Leukemia, MESH:D015470	Decitabine	22.31	21
ESPL1	9700	Leukemia, MESH:D015470	Calcitriol|D	22.31	13
RPL6	6128	Leukemia, MESH:D015470	Arsenic Tr	22.31	33
LIPC	3990	Leukemia, MESH:D015473	Arsenic|De	22.3	156
ABCC2	1244	Leukemia, MESH:D004915	Daunorub	22.29	4
ARID5B	84159	Leukemia, MESH:D015473	Arsenic|Ar	22.29	265
FBXO32	114907	Leukemia, MESH:D015473	Arsenic|Ar	22.29	160
RPL17	6139	Leukemia, MESH:D015473	arsenic dis	22.29	159
SLC11A2	4891	Leukemia, MESH:D015470	Air Polluta	22.28	16
JUN	3725	Leukemia, MESH:D007948	2-(2-amin	22.27	4
MMP3	4314	Leukemia, MESH:D015473	arsenite|ca	22.26	158
SOCS3	9021	Leukemia, MESH:D015473	2-(2-chlor	22.26	161
CCL11	6356	Leukemia, MESH:D015473	Buthionine	22.25	9
LUZP2	338645	Leukemia, MESH:D015473	Calcitriol|D	22.25	17
NPIPB3	23117	Leukemia, MESH:D015473	Dexameth	22.25	156
POU5F1	5460	Leukemia, MESH:D015473	Arsenic|Ar	22.25	264
ATP5MJ	9556	Leukemia, MESH:D015470	Doxorubic	22.24	13
FBN2	2201	Leukemia, MESH:D015470	Arsenic Tr	22.24	21
NR2F1	7025	Leukemia, MESH:D015470	Calcitriol|D	22.24	18
SLC4A1	6521	Leukemia, MESH:D015470	Dexameth	22.24	15
ACO1	48	Leukemia, MESH:D015470	Arsenic Tr	22.23	31
ACVRL1	94	Leukemia, MESH:D015470	Arsenic Tr	22.23	30
ALPI	248	Leukemia, MESH:D015470	mangiferin	22.23	15
ATXN1	6310	Leukemia, MESH:D015473	Arsenic|Bu	22.23	160
BMPR1B	658	Leukemia, MESH:D015470	Calcitriol|D	22.23	18
CBX7	23492	Leukemia, MESH:D015470	Arsenic Tr	22.23	21
CCDC92	80212	Leukemia, MESH:D015470	Arsenic Tr	22.23	20
CLCN4	1183	Leukemia, MESH:D015470	Air Polluta	22.23	15
CSF3R	1441	Leukemia, MESH:D015470	Calcitriol|D	22.23	17
DCX	1641	Leukemia, MESH:D015470	Arsenic Tr	22.23	19
DHRS9	10170	Leukemia, MESH:D015473	Antimony	22.23	265
DUBR	344595	Leukemia, MESH:D015470	Doxorubic	22.23	14

EEF1G	1937	Leukemia, MESH:D015473	Arsenic|Ar	22.23	266
F2	2147	Leukemia, MESH:D015473	arsenite|Cy	22.23	161
FIBIN	387758	Leukemia, MESH:D015470	Calcitriol|D	22.23	12
FMOD	2331	Leukemia, MESH:D015470	Arsenic Tr	22.23	23
FOXK2	3607	Leukemia, MESH:D015473	Arsenic|M	22.23	156
GTF2H1	2965	Leukemia, MESH:D015470	Air Polluta	22.23	20
KCNAB2	8514	Leukemia, MESH:D015470	Doxorubic	22.23	32
NFKBIZ	64332	Leukemia, MESH:D015470	Benzoates	22.23	21
NREP	9315	Leukemia, MESH:D015470	Arsenic Tr	22.23	26
PRUNE2	158471	Leukemia, MESH:D015470	Dexameth	22.23	17
RAB5A	5868	Leukemia, MESH:D015470	Arsenic Tr	22.23	15
RAD17	5884	Leukemia, MESH:D015470	Arsenic Tr	22.23	39
RDH10	157506	Leukemia, MESH:D015470	Dasatinib|	22.23	16
SEPTIN11	55752	Leukemia, MESH:D015470	Arsenic Tr	22.23	23
SOBP	55084	Leukemia, MESH:D015470	Dexameth	22.23	18
SYT12	91683	Leukemia, MESH:D015470	Calcitriol|D	22.23	14
TIMP4	7079	Leukemia, MESH:D015470	Decitabine	22.23	18
AMOTL1	154810	Leukemia, MESH:D015473	arsenite|D	22.22	159
H2AC6	8334	Leukemia, MESH:D015473	Arsenic|Ar	22.22	265
LDLRAP1	26119	Leukemia, MESH:D015473	Arsenic|Ca	22.22	156
LRRN1	57633	Leukemia, MESH:D015473	Arsenic|ars	22.22	157
MNT	4335	Leukemia, MESH:D015473	Arsenic Tr	22.22	262
RPLP0	6175	Leukemia, MESH:D015473	Arsenic|Ar	22.22	265
ARPC3	10094	Leukemia, MESH:D015473	Arsenic Tr	22.21	262
BCHE	590	Leukemia, MESH:D015473	alpha-Toc	22.21	172
WAS	7454	Leukemia, MESH:D015473	arsenite|D	22.21	157
C3	718	Leukemia, MESH:D015473	Arsenic Tr	22.2	263
ACE	1636	Leukemia, MESH:D015470	Air Polluta	22.19	22
DHX38	9785	Leukemia, MESH:D015470	Dexameth	22.18	14
GEN1	348654	Leukemia, MESH:D015470	Calcitriol|D	22.18	13
H2BC6	8344	Leukemia, MESH:D015470	Doxorubic	22.18	19
MIR34C	407042	Leukemia, MESH:D015470	Arsenic Tr	22.18	43
RPS6KB1	6198	Leukemia, MESH:D007948	2-(2-amin	22.18	3
ASB7	140460	Leukemia, MESH:D015470	Dexameth	22.17	15
ATP8B1	5205	Leukemia, MESH:D015470	Calcitriol|D	22.17	16
AVP	551	Leukemia, MESH:D015470	Chloroqui	22.17	21
CADM1	23705	Leukemia, MESH:D015470	Arsenic Tr	22.17	30
CHODL	140578	Leukemia, MESH:D015470	Arsenic Tr	22.17	21
CNPY3	10695	Leukemia, MESH:D015470	Dexameth	22.17	14
E2F6	1876	Leukemia, MESH:D015470	Arsenic Tr	22.17	35
EIF1AD	84285	Leukemia, MESH:D015470	Dexameth	22.17	16
EIF1B	10289	Leukemia, MESH:D015470	Air Polluta	22.17	14
EVC	2121	Leukemia, MESH:D015470	Calcitriol|D	22.17	17
HAUS6	54801	Leukemia, MESH:D015470	Arsenic Tr	22.17	20
ICAM3	3385	Leukemia, MESH:D015470	Arsenic Tr	22.17	14
ITFG2	55846	Leukemia, MESH:D015470	Doxorubic	22.17	14
KLHL12	59349	Leukemia, MESH:D015470	Benzene|D	22.17	34
KLHL14	57565	Leukemia, MESH:D015470	Arsenic Tr	22.17	17
MAP1LC3A	84557	Leukemia, MESH:D015470	Arsenic Tr	22.17	30
MT1F	4494	Leukemia, MESH:D015470	Arsenic Tr	22.17	26
PARN	5073	Leukemia, MESH:D015470	Arsenic Tr	22.17	16
PDPK1	5170	Leukemia, MESH:D015470	Arsenic Tr	22.17	24
PHLPP2	23035	Leukemia, MESH:D015470	Benzene|D	22.17	23
PRDX5	25824	Leukemia, MESH:D015470	Arsenic Tr	22.17	29
RAB11FIP2	22841	Leukemia, MESH:D015470	Doxorubic	22.17	16
SAMD8	142891	Leukemia, MESH:D015470	Air Polluta	22.17	20
SCRN2	90507	Leukemia, MESH:D015470	Dexameth	22.17	13
SLC4A8	9498	Leukemia, MESH:D015470	Cytarabine	22.17	81

THAP1	55145	Leukemia, MESH:D015470		Dexameth	22.17	13
TIMM22	29928	Leukemia, MESH:D015470		Dexameth	22.17	10
TMSB15A	11013	Leukemia, MESH:D015470		Calcitriol|[	22.17	11
TNFRSF18	8784	Leukemia, MESH:D015470		Arsenic Tr	22.17	21
VAT1L	57687	Leukemia, MESH:D015470		Decitabine	22.17	20
ZMYM6	9204	Leukemia, MESH:D015470		Decitabine	22.17	15
ARG2	384	Leukemia, MESH:D015473		Arsenic|ars	22.16	25
ARPC1B	10095	Leukemia, MESH:D015473		Arsenic Tr	22.16	262
F13A1	2162	Leukemia, MESH:D015473		Arsenic Tr	22.16	260
NCOA1	8648	Leukemia, MESH:D015473		arsenite|Ca	22.16	158
NR1H2	7376	Leukemia, MESH:D015473		Arsenic|Ar	22.16	263
ORC6	23594	Leukemia, MESH:D015473		arsenite|Ca	22.16	158
OXCT1	5019	Leukemia, MESH:D015473		Arsenic|De	22.16	160
RASGRP1	10125	Leukemia, MESH:D015473		arsenite|D	22.16	157
SRGN	5552	Leukemia, MESH:D015473		Arsenic Tr	22.16	266
WSB1	26118	Leukemia, MESH:D015473		Arsenic|ars	22.16	161
ATF7IP	55729	Leukemia, MESH:D015470		Doxorubic	22.13	17
CEP57	9702	Leukemia, MESH:D015470		Arsenic Tr	22.13	29
FRMD4B	23150	Leukemia, MESH:D015470		Doxorubic	22.13	16
HIVEP3	59269	Leukemia, MESH:D015470		Dexameth	22.13	14
HNRNPR	10236	Leukemia, MESH:D015470		Arsenic Tr	22.13	21
ICA1	3382	Leukemia, MESH:D015470		Cyclophos	22.13	15
MBNL3	55796	Leukemia, MESH:D015470		Decitabine	22.13	24
NDE1	54820	Leukemia, MESH:D015470		Calcitriol|[	22.13	18
NMT2	9397	Leukemia, MESH:D015470		Arsenic Tr	22.13	28
OGFRL1	79627	Leukemia, MESH:D015470		Azacitidine	22.13	17
RBM17	84991	Leukemia, MESH:D015473		Arsenic Tr	22.13	159
RBM24	221662	Leukemia, MESH:D015470		Calcitriol|I	22.13	12
SIX1	6495	Leukemia, MESH:D015470		Dexameth	22.13	14
CD58	965	Leukemia, MESH:D015473		Arsenic|Ge	22.12	156
SOX10	6663	Leukemia, MESH:D015473		caffeic aci	22.12	153
TCIM	56892	Leukemia, MESH:D015470		Arsenic Tr	22.12	20
TLE4	7091	Leukemia, MESH:D015470		Decitabine	22.12	17
VEZF1	7716	Leukemia, MESH:D015473		Cytarabine	22.12	160
FES	2242	Leukemia, MESH:D015473		Arsenic|De	22.11	162
SOX3	6658	Leukemia, MESH:D015473		arsenite|Cy	22.11	160
CALB1	793	Leukemia, MESH:D015473		arsenite|Ca	22.1	159
CASP8	841	Leukemia, MESH:D007948		2-(2-amin	22.1	4
STARD4	134429	Leukemia, MESH:D015473		Arsenic|Ar	22.1	265
VEZF1	7716	Leukemia, MESH:D004915		Cytarabine	22.1	3
ADCY7	113	Leukemia, MESH:D015473		arsenite|Ca	22.09	156
FOLR1	2348	Leukemia, MESH:D015470		Air Polluta	22.09	17
KLF15	28999	Leukemia, MESH:D015470		Dexameth	22.09	17
TPO	7173	Leukemia, MESH:D015470		Arsenic Tr	22.09	24
FGF2	2247	Leukemia, MESH:D007948		2-(2-amin	22.07	3
SCARB1	949	Leukemia, MESH:D015473		Arsenic|Ch	22.07	159
HSPB1	3315	Leukemia, MESH:D007948		2-(2-amin	22.06	3
ITGB1BP3	415448	Leukemia, MESH:D015470		Decitabine	22.06	21
BAD	572	Leukemia, MESH:D007948		2-(2-amin	22.05	3
CYBA	1535	Leukemia, MESH:D004915		Cytarabine	22.04	3
GC	2638	Leukemia, MESH:D015473		arsenite|Bu	22.04	156
ETV6	2120	Leukemia, MESH:D01	marker/m	Doxorubic	22.03	24
AKT2	208	Leukemia, MESH:D015473		Arsenic Tr	22.03	264
CCDC186	55088	Leukemia, MESH:D015470		Bortezomi	22.03	13
CDCP1	64866	Leukemia, MESH:D015470		Benzene|C	22.03	28
CENPM	79019	Leukemia, MESH:D015470		Calcitriol|[	22.03	16
COA7	65260	Leukemia, MESH:D015470		Etoposide	22.03	19
CST6	1474	Leukemia, MESH:D015470		Calcitriol|[	22.03	19

DNER	92737	Leukemia, MESH:D015470		Calcitriol|D	22.03	12
DTL	51514	Leukemia, MESH:D015473		Arsenic Tr	22.03	262
EVI2A	2123	Leukemia, MESH:D015470		Arsenic Tr	22.03	30
GPRC5C	55890	Leukemia, MESH:D015470		Cytarabine	22.03	79
IGSF3	3321	Leukemia, MESH:D015470		Calcitriol|D	22.03	23
IRF5	3663	Leukemia, MESH:D015470		Dexameth	22.03	12
LMO4	8543	Leukemia, MESH:D015470		Doxorubic	22.03	17
LRRFIP1	9208	Leukemia, MESH:D015473		Arsenic|Ar	22.03	267
MCPT1	17224	Leukemia, MESH:D015470		Air Polluta	22.03	12
METAP1	23173	Leukemia, MESH:D015470		Air Polluta	22.03	19
MMP11	4320	Leukemia, MESH:D015470		Cyclophos	22.03	15
MRPS12	6183	Leukemia, MESH:D015470		Arsenic Tr	22.03	16
N4BP2L1	90634	Leukemia, MESH:D015470		Arsenic Tr	22.03	22
NUDT14	256281	Leukemia, MESH:D015470		Decitabine	22.03	15
PIGW	284098	Leukemia, MESH:D015470		Calcitriol|D	22.03	15
PRRX1	5396	Leukemia, MESH:D015470		Dexameth	22.03	16
TAB3	257397	Leukemia, MESH:D015470		Doxorubic	22.03	14
THAP11	57215	Leukemia, MESH:D015470		Dexameth	22.03	16
TNNT1	7138	Leukemia, MESH:D015470		Calcitriol|D	22.03	53
TNPO2	30000	Leukemia, MESH:D015470		Calcitriol|D	22.03	12
ZFP64	55734	Leukemia, MESH:D015470		Azacitidine	22.03	15
SPRY4	81848	Leukemia, MESH:D01	marker/m	Dexameth	22.02	20
ARHGAP2	201176	Leukemia, MESH:D015470		Decitabine	22.02	19
BVES	11149	Leukemia, MESH:D015470		Arsenic Tr	22.02	19
CNNM4	26504	Leukemia, MESH:D015470		Arsenic Tr	22.02	22
COG3	83548	Leukemia, MESH:D015470		Dexameth	22.02	15
CPTP	80772	Leukemia, MESH:D015470		Arsenic Tr	22.02	20
CYGB	114757	Leukemia, MESH:D015470		Arsenic Tr	22.02	29
DEF8	54849	Leukemia, MESH:D015470		Calcitriol|D	22.02	16
EDEM2	55741	Leukemia, MESH:D015470		Dexameth	22.02	9
ISM1	140862	Leukemia, MESH:D015470		Calcitriol|D	22.02	9
NCAPH	23397	Leukemia, MESH:D015470		Arsenic Tr	22.02	24
NPY6R	4888	Leukemia, MESH:D015470		Arsenic Tr	22.02	18
RMI1	80010	Leukemia, MESH:D015470		Calcitriol|D	22.02	11
ROGDI	79641	Leukemia, MESH:D015470		Dexameth	22.02	14
SCML1	6322	Leukemia, MESH:D015470		Dexameth	22.02	14
SLAIN1	122060	Leukemia, MESH:D015470		Arsenic Tr	22.02	20
SLC41A3	54946	Leukemia, MESH:D015470		Arsenic Tr	22.02	16
SUPV3L1	6832	Leukemia, MESH:D015470		Dexameth	22.02	14
SYK	6850	Leukemia, MESH:D015470		Air Polluta	22.02	25
TMEM208	29100	Leukemia, MESH:D015470		Decitabine	22.02	20
TNFRSF10	8793	Leukemia, MESH:D015470		Arsenic Tr	22.02	21
CENPL	91687	Leukemia, MESH:D015473		Arsenic|ars	22.01	158
TPK1	27010	Leukemia, MESH:D015473		Arsenic|ars	22.01	21
H4F16	320332	Leukemia, MESH:D015470		Alitretinoi	22	20
KNG1	3827	Leukemia, MESH:D015470		Dexameth	22	19
UQCR10	29796	Leukemia, MESH:D015473		Arsenic|ars	22	160
B2M	567	Leukemia, MESH:D015473		Arsenic|Ca	21.99	162
ZNF574	64763	Leukemia, MESH:D015470		Dexameth	21.99	12
ANO2	57101	Leukemia, MESH:D015470		Dexameth	21.98	15
C9ORF40	55071	Leukemia, MESH:D015470		Calcitriol|F	21.98	10
SNCA	6622	Leukemia, MESH:D015473		alpha-Toc	21.98	270
CRABP2	1382	Leukemia, MESH:D015473		alpha-Toc	21.97	159
DEPTOR	64798	Leukemia, MESH:D015473		Cytarabine	21.97	158
EEF1A2	1917	Leukemia, MESH:D015473		Arsenic Tr	21.97	263
KNSTRN	90417	Leukemia, MESH:D015473		arsenite|Ca	21.97	157
PXN	5829	Leukemia, MESH:D015473		arsenite|D	21.97	156
INS1	16333	Leukemia, MESH:D015473		alpha-Toc	21.96	163

DDX5	1655	Leukemia, MESH:D015470		Arsenic Tr	21.95	19
GLI2	2736	Leukemia, MESH:D015470		Arsenic Tr	21.95	22
HEY1	23462	Leukemia, MESH:D015470		Arsenic Tr	21.95	87
PIK3CA	5290	Leukemia, MESH:D015473		Arsenic Tr	21.95	262
SLIT2	9353	Leukemia, MESH:D015470		Cytarabine	21.95	90
TNFAIP6	7130	Leukemia, MESH:D015473		Arsenic|Ar	21.95	265
CSF2	1437	Leukemia, MESH:D007948		2-(2-amin	21.94	3
UGT1A1	54658	Leukemia, MESH:D015473		alpha-Toc	21.94	160
ABCD2	225	Leukemia, MESH:D015470		Dexameth	21.93	12
AGPAT2	10555	Leukemia, MESH:D015470		Arsenic Tr	21.93	23
ATP8A1	10396	Leukemia, MESH:D015470		Arsenic Tr	21.93	24
CCND3	896	Leukemia, MESH:D004915		Cytarabine	21.93	3
CELF1	10658	Leukemia, MESH:D015470		Dexameth	21.93	19
CKAP2L	150468	Leukemia, MESH:D015470		Calcitriol|D	21.93	17
FGF12	2257	Leukemia, MESH:D015470		Arsenic Tr	21.93	22
GSDMD	79792	Leukemia, MESH:D015470		Paclitaxel|	21.93	13
HDAC7	51564	Leukemia, MESH:D015470		Decitabine	21.93	20
KANK4	163782	Leukemia, MESH:D015470		Arsenic Tr	21.93	88
KAT6B	23522	Leukemia, MESH:D015470		Arsenic Tr	21.93	20
PCYT2	5833	Leukemia, MESH:D015470		Arsenic Tr	21.93	28
PIMREG	54478	Leukemia, MESH:D015470		Calcitriol|D	21.93	15
PLAA	9373	Leukemia, MESH:D015470		Bortezomi	21.93	16
PRF1	5551	Leukemia, MESH:D007948		2-(2-amin	21.93	3
S1PR2	9294	Leukemia, MESH:D015470		Decitabine	21.93	18
SFRP4	6424	Leukemia, MESH:D015470		Calcitriol|D	21.93	29
SSBP1	6742	Leukemia, MESH:D015470		Arsenic Tr	21.93	23
SSBP2	23635	Leukemia, MESH:D015470		Arsenic Tr	21.93	26
TMTC2	160335	Leukemia, MESH:D015470		Arsenic Tr	21.93	22
USP5	8078	Leukemia, MESH:D015470		Bortezomi	21.93	11
IDH2	3418	Leukemia, MESH:D01	marker/m	Arsenic Tr	21.91	25
ATG12	9140	Leukemia, MESH:D015473		Arsenic Tr	21.91	263
BMP6	654	Leukemia, MESH:D015473		Antimony	21.91	158
BTBD3	22903	Leukemia, MESH:D015473		Arsenic|Ar	21.91	165
C15ORF48	84419	Leukemia, MESH:D015473		Arsenic|Ar	21.91	262
METTL7A	25840	Leukemia, MESH:D015473		Arsenic|Ca	21.91	159
NPC2	10577	Leukemia, MESH:D015473		arsenite|D	21.91	161
NPY	4852	Leukemia, MESH:D015470		Arsenic Tr	21.91	87
OLFML3	56944	Leukemia, MESH:D015473		Arsenic Tr	21.91	263
PRMT1	3276	Leukemia, MESH:D015473		Arsenic Tr	21.91	262
CHCHD2	51142	Leukemia, MESH:D015473		Arsenic|ars	21.9	159
LHX6	26468	Leukemia, MESH:D015473		Arsenic Tr	21.9	262
MYO9B	4650	Leukemia, MESH:D015473		Arsenic|Ar	21.9	265
NDUFB4	4710	Leukemia, MESH:D015473		arsenite|D	21.9	158
SHOX2	6474	Leukemia, MESH:D015473		arsenite|Ca	21.9	155
SRGAP1	57522	Leukemia, MESH:D015473		Arsenic|ars	21.9	158
TRIM38	10475	Leukemia, MESH:D004915		Doxorubic	21.9	3
COX1	4512	Leukemia, MESH:D015470		Arsenic Tr	21.89	26
NR4A3	8013	Leukemia, MESH:D015470		Air Polluta	21.89	34
ACAD8	27034	Leukemia, MESH:D015470		Dexameth	21.88	14
ALG8	79053	Leukemia, MESH:D015470		Doxorubic	21.88	15
CHST7	56548	Leukemia, MESH:D015470		Benzene|C	21.88	24
EYA3	2140	Leukemia, MESH:D015470		Dexameth	21.88	16
H1-5	3009	Leukemia, MESH:D015470		Calcitriol|D	21.88	17
HAPLN3	145864	Leukemia, MESH:D015470		Dasatinib|	21.88	14
LGI1	9211	Leukemia, MESH:D015470		Arsenic Tr	21.88	25
MT1	17748	Leukemia, MESH:D015473		Arsenic|ars	21.88	161
NBEAL2	23218	Leukemia, MESH:D015470		Dexameth	21.88	14
RASL12	51285	Leukemia, MESH:D015470		Decitabine	21.88	19

RILPL1	353116	Leukemia, MESH:D015470		Decitabine	21.88	15
SETMAR	6419	Leukemia, MESH:D015470		Arsenic Tr	21.88	20
SHROOM4	57477	Leukemia, MESH:D015470		Dexameth	21.88	16
SLC26A11	284129	Leukemia, MESH:D015470		Bortezomi	21.88	15
SNX13	23161	Leukemia, MESH:D015470		Decitabine	21.88	21
TMEM184	25829	Leukemia, MESH:D015470		Doxorubic	21.88	14
TOMM34	10953	Leukemia, MESH:D015470		Arsenic Tr	21.88	16
WDR44	54521	Leukemia, MESH:D015470		Bortezomi	21.88	13
CDKN1C	1028	Leukemia, MESH:D015473		Arsenic|ars	21.87	163
TM7SF2	7108	Leukemia, MESH:D015470		Chloroqui	21.87	11
MAOB	4129	Leukemia, MESH:D015470		Calcitriol|D	21.86	21
MGST1	4257	Leukemia, MESH:D015470		Arsenic Tr	21.86	25
BCAR1	9564	Leukemia, MESH:D015473		Arsenic|Ar	21.85	265
FLNC	2318	Leukemia, MESH:D015473		arsenite|Ca	21.85	158
GREM1	26585	Leukemia, MESH:D015473		Arsenic Tr	21.85	263
TFDP1	7027	Leukemia, MESH:D015473		Arsenic|Ar	21.85	268
PLEC	5339	Leukemia, MESH:D015473		Arsenic|ars	21.84	160
PTMA	5757	Leukemia, MESH:D015473		Arsenic Tr	21.84	261
SEPTIN9	10801	Leukemia, MESH:D01	marker/m	Decitabine	21.83	18
ADCY1	107	Leukemia, MESH:D015470		Arsenic Tr	21.83	20
AFAP1	60312	Leukemia, MESH:D015470		Arsenic Tr	21.83	16
CAP2	10486	Leukemia, MESH:D015470		Dexameth	21.83	18
CCL1	6346	Leukemia, MESH:D015470		Arsenic Tr	21.83	20
CDK9	1025	Leukemia, MESH:D015470		alvocidib|D	21.83	16
COL27A1	85301	Leukemia, MESH:D015470		Doxorubic	21.83	16
CYP7B1	9420	Leukemia, MESH:D015470		Arsenic Tr	21.83	38
EBI3	10148	Leukemia, MESH:D015470		Decitabine	21.83	22
EHD2	30846	Leukemia, MESH:D015470		Doxorubic	21.83	15
GLIPR2	152007	Leukemia, MESH:D015470		Calcitriol|D	21.83	12
IL12B	3593	Leukemia, MESH:D007948		2-(2-amin	21.83	3
KDM5B	10765	Leukemia, MESH:D015470		Benzene|D	21.83	31
LSR	51599	Leukemia, MESH:D015470		Arsenic Tr	21.83	29
ME3	10873	Leukemia, MESH:D015470		Dexameth	21.83	10
MFN2	9927	Leukemia, MESH:D015470		Arsenic Tr	21.83	23
MMP15	4324	Leukemia, MESH:D015470		Decitabine	21.83	25
MYO5B	4645	Leukemia, MESH:D015470		Arsenic Tr	21.83	20
OAS3	4940	Leukemia, MESH:D015470		Air Polluta	21.83	15
PKIB	5570	Leukemia, MESH:D015470		Arsenic Tr	21.83	23
PPIB	5479	Leukemia, MESH:D015470		Dexameth	21.83	15
SMIM3	85027	Leukemia, MESH:D015470		Calcitriol|D	21.83	11
TICAM1	148022	Leukemia, MESH:D015470		Dexameth	21.83	17
TNFSF12	8742	Leukemia, MESH:D015470		Arsenic Tr	21.83	21
TSHZ1	10194	Leukemia, MESH:D015470		Doxorubic	21.83	20
AHI1	54806	Leukemia, MESH:D015473		Arsenic|Cy	21.82	155
EIF2AK3	9451	Leukemia, MESH:D015473		Arsenic|Ar	21.82	267
APBB1	322	Leukemia, MESH:D015473		Arsenic|Ar	21.8	262
CLK1	1195	Leukemia, MESH:D015470		Air Polluta	21.8	23
KRT6A	3853	Leukemia, MESH:D015473		Arsenic|Ar	21.8	266
MALL	7851	Leukemia, MESH:D015473		Arsenic Tr	21.8	156
MSC	9242	Leukemia, MESH:D015473		Arsenic Tr	21.8	262
PIK3R3	8503	Leukemia, MESH:D015470		Alitretinoi	21.8	17
SYNCRIP	10492	Leukemia, MESH:D015470		Arsenic Tr	21.8	31
TMEM107	84314	Leukemia, MESH:D015473		Arsenic Tr	21.8	157
TRAF6	7189	Leukemia, MESH:D015470		Arsenic Tr	21.8	23
TRIM38	10475	Leukemia, MESH:D015473		arsenite|Ca	21.8	155
DSC2	1824	Leukemia, MESH:D015473		Arsenic|Ar	21.79	268
LMCD1	29995	Leukemia, MESH:D015473		Calcitriol|C	21.79	20
SPON2	10417	Leukemia, MESH:D015473		Arsenic Tr	21.79	263

SPRY2	10253	Leukemia, MESH:D015473		Arsenic|ars	21.79	161
DNMT3B	1789	Leukemia, MESH:D015470		Arsenic Tr	21.78	33
MAPK14	1432	Leukemia, MESH:D007948		2-(2-amin	21.78	3
AIF1	199	Leukemia, MESH:D015470		Arsenic Tr	21.77	38
NR4A1	3164	Leukemia, MESH:D015473		Arsenic|Ar	21.76	267
CKMT1A	548596	Leukemia, MESH:D015470		Dexameth	21.75	9
IL18	3606	Leukemia, MESH:D015473		Arsenic Tr	21.75	263
SIRT1	23411	Leukemia, MESH:D015473		arsenic dis	21.75	158
SLC22A16	85413	Leukemia, MESH:D015470		Daunorub	21.75	38
TRABD2A	129293	Leukemia, MESH:D015470		Dexameth	21.75	9
ADA2	51816	Leukemia, MESH:D015470		Doxorubic	21.74	13
APOL3	80833	Leukemia, MESH:D015470		Dexameth	21.74	14
ATN1	1822	Leukemia, MESH:D015470		Arsenic Tr	21.74	87
AZI2	64343	Leukemia, MESH:D015470		Decitabine	21.74	20
CARD16	114769	Leukemia, MESH:D015470		Arsenic Tr	21.74	19
CARM1	10498	Leukemia, MESH:D015470		Arsenic Tr	21.74	19
CECR2	27443	Leukemia, MESH:D015470		Doxorubic	21.74	14
CWC27	10283	Leukemia, MESH:D015470		Daunorub	21.74	35
DBF4B	80174	Leukemia, MESH:D015470		Calcitriol|D	21.74	12
DLX4	1748	Leukemia, MESH:D015470		Arsenic Tr	21.74	15
FGFR1	2260	Leukemia, MESH:D015473		Arsenic Tr	21.74	266
HIF1A	3091	Leukemia, MESH:D004915		Cytarabine	21.74	3
HMGCR	3156	Leukemia, MESH:D015470		Benzene|B	21.74	39
JOSD1	9929	Leukemia, MESH:D015470		Air Polluta	21.74	22
LGALS1	3956	Leukemia, MESH:D015473		Arsenic Tr	21.74	267
MAD1L1	8379	Leukemia, MESH:D015470		Azacitidine	21.74	16
MICALL2	79778	Leukemia, MESH:D015470		Dexameth	21.74	14
ND1	4535	Leukemia, MESH:D015470		Chloramph	21.74	18
NDFIP1	80762	Leukemia, MESH:D015470		Dexameth	21.74	9
PFDN4	5203	Leukemia, MESH:D015470		Bortezomi	21.74	15
PHIP	55023	Leukemia, MESH:D015470		Doxorubic	21.74	16
TOM1L2	146691	Leukemia, MESH:D015470		Dexameth	21.74	9
TTC14	151613	Leukemia, MESH:D015470		Dexameth	21.74	13
VIPR1	7433	Leukemia, MESH:D015470		Alitretinoi	21.74	12
WDR74	54663	Leukemia, MESH:D015470		Bortezomi	21.74	10
ZBTB2	57621	Leukemia, MESH:D015470		Doxorubic	21.74	15
ZNF493	284443	Leukemia, MESH:D015470		Dexameth	21.74	12
TRH	7200	Leukemia, MESH:D01	marker/m	Azacitidine	21.73	22
E2F7	144455	Leukemia, MESH:D015473		Arsenic|ars	21.73	14
EEPD1	80820	Leukemia, MESH:D015470		Calcitriol|D	21.73	10
EWSR1	2130	Leukemia, MESH:D015470		Arsenic Tr	21.73	23
GAMT	2593	Leukemia, MESH:D015470		Cyclophos	21.73	17
IL1RAP	3556	Leukemia, MESH:D015470		Benzene|C	21.73	31
INSIG1	3638	Leukemia, MESH:D015473		Arsenic Tr	21.73	262
LGALS3BP	3959	Leukemia, MESH:D015470		Arsenic Tr	21.73	21
RPL13	6137	Leukemia, MESH:D015470		Dexameth	21.73	20
STAT1	6772	Leukemia, MESH:D007948		2-(2-amin	21.73	3
TACC1	6867	Leukemia, MESH:D015470		Arsenic Tr	21.73	22
TG	7038	Leukemia, MESH:D015473		Arsenic|ca	21.73	158
TSLP	85480	Leukemia, MESH:D015473		Arsenic|ars	21.73	158
IL33	90865	Leukemia, MESH:D015470		Air Polluta	21.72	18
MIR125A	406910	Leukemia, MESH:D015473		Arsenic Tr	21.72	155
SLC39A10	57181	Leukemia, MESH:D015473		Arsenic|Ar	21.72	264
SRM	6723	Leukemia, MESH:D015473		Calcitriol|D	21.72	155
ANKRD22	118932	Leukemia, MESH:D015473		Arsenic|ars	21.7	157
ANXA3	306	Leukemia, MESH:D015473		Arsenic|Ar	21.7	265
ARPC5L	81873	Leukemia, MESH:D015473		Arsenic|Ar	21.7	264
MBTD1	54799	Leukemia, MESH:D015473		Calcitriol|C	21.7	157

PCK2	5106	Leukemia,	MESH:D015473		Arsenic Tr	21.7	263
RAB2A	5862	Leukemia,	MESH:D015473		Arsenic|Ar	21.7	262
SKA3	221150	Leukemia,	MESH:D015473		Calcitriol|D	21.7	155
VDAC1	7416	Leukemia,	MESH:D015473		Arsenic Tr	21.7	160
ZHX3	23051	Leukemia,	MESH:D015473		Arsenic|Ar	21.69	263
CD36	948	Leukemia,	MESH:D007948		2-(2-amin	21.68	3
NRAS	4893	Leukemia,	MESH:D01	marker/m	Arsenic|Da	21.67	25
CD59	966	Leukemia,	MESH:D015473		Arsenic|Bu	21.67	157
NRP2	8828	Leukemia,	MESH:D015473		Arsenic|ars	21.67	13
PDCD4	27250	Leukemia,	MESH:D015470		Arsenic Tr	21.67	25
SELENBP1	8991	Leukemia,	MESH:D015470		Androgen	21.67	19
CROT	54677	Leukemia,	MESH:D015470		Alitretinoi	21.66	13
EREG	2069	Leukemia,	MESH:D015473		Arsenic|ars	21.66	158
KRT7	3855	Leukemia,	MESH:D015470		Alitretinoi	21.66	19
L1CAM	3897	Leukemia,	MESH:D015470		Decitabine	21.66	19
MYOF	26509	Leukemia,	MESH:D015473		Arsenic Tr	21.66	265
PRKCE	5581	Leukemia,	MESH:D015473		Arsenic|Ar	21.66	262
SLCO2A1	6578	Leukemia,	MESH:D015473		Arsenic|De	21.66	158
AIG1	51390	Leukemia,	MESH:D015470		Calcitriol|D	21.64	16
BACE2	25825	Leukemia,	MESH:D015470		Doxorubic	21.64	33
BMP5	653	Leukemia,	MESH:D015470		Cytarabine	21.64	88
CD28	940	Leukemia,	MESH:D015470		Air Polluta	21.64	13
DDX39B	7919	Leukemia,	MESH:D015470		Arsenic Tr	21.64	21
DOCK4	9732	Leukemia,	MESH:D015470		Calcitriol|D	21.64	18
DPP7	29952	Leukemia,	MESH:D015470		Dronabinc	21.64	19
EXOSC8	11340	Leukemia,	MESH:D015470		Calcitriol|D	21.64	15
GMFB	2764	Leukemia,	MESH:D015470		Dexameth	21.64	17
ITGA2B	3674	Leukemia,	MESH:D007948		Arsenic Tr	21.64	3
LARP1B	55132	Leukemia,	MESH:D015470		Calcitriol|D	21.64	13
MOV10	4343	Leukemia,	MESH:D015470		(+)-JQ1 cc	21.64	11
PAK2	5062	Leukemia,	MESH:D015470		Arsenic Tr	21.64	23
PLP2	5355	Leukemia,	MESH:D015470		Decitabine	21.64	25
PSMC3IP	29893	Leukemia,	MESH:D015470		Bezafibrat	21.64	13
REV1	51455	Leukemia,	MESH:D015470		Cyclophos	21.64	15
SEC31A	22872	Leukemia,	MESH:D015470		Doxorubic	21.64	25
SETBP1	26040	Leukemia,	MESH:D015470		Dexameth	21.64	17
SFXN2	118980	Leukemia,	MESH:D015470		Doxorubic	21.64	16
SYVN1	84447	Leukemia,	MESH:D015470		Doxorubic	21.64	15
TLR5	7100	Leukemia,	MESH:D015470		Air Polluta	21.64	24
COL1A1	1277	Leukemia,	MESH:D004915		Cytarabine	21.63	4
CYP2C19	1557	Leukemia,	MESH:D015470		Bortezomi	21.63	22
HAND2	9464	Leukemia,	MESH:D004915		Cytarabine	21.62	2
MT1A	4489	Leukemia,	MESH:D004915		Cytarabine	21.62	3
MYOM2	9172	Leukemia,	MESH:D015473		Arsenic|Da	21.62	24
S100A12	6283	Leukemia,	MESH:D015473		Antimony	21.62	154
SAT1	6303	Leukemia,	MESH:D015473		Arsenic Tr	21.62	264
SELENOP	6414	Leukemia,	MESH:D015473		Arsenic Tr	21.62	262
ALPL	249	Leukemia,	MESH:D015473		Arsenic Tr	21.61	264
CYP24A1	1591	Leukemia,	MESH:D015470		Arsenic Tr	21.61	18
NCAPG	64151	Leukemia,	MESH:D015473		Calcitriol|D	21.61	156
RIPPLY3	53820	Leukemia,	MESH:D015470		Alitretinoi	21.61	23
SLC2A14	144195	Leukemia,	MESH:D015470		Calcitriol|D	21.61	16
SOX17	64321	Leukemia,	MESH:D015473		arsenite|D	21.61	155
SPATA5	166378	Leukemia,	MESH:D015470		Bortezomi	21.61	13
UGT1A5	54579	Leukemia,	MESH:D015470		Alitretinoi	21.61	8
VDR	7421	Leukemia,	MESH:D004915		Cytarabine	21.61	2
POU4F1	5457	Leukemia,	MESH:D01	marker/m	Arsenic Tr	21.6	16
BTRC	8945	Leukemia,	MESH:D015470		Decitabine	21.6	16

CCN2	1490	Leukemia, MESH:D007948	2-(2-amin	21.6	3
CGA	1081	Leukemia, MESH:D015470	2-(2-chlor	21.6	21
DDX50	79009	Leukemia, MESH:D015470	Decitabine	21.6	24
DOK5	55816	Leukemia, MESH:D015470	Arsenic Tr	21.6	15
EGFLAM	133584	Leukemia, MESH:D015470	Cytarabine	21.6	81
EIF4G1	1981	Leukemia, MESH:D015473	Arsenic Tr	21.6	263
FAM126A	84668	Leukemia, MESH:D015470	Calcitriol|D	21.6	8
FOXK1	221937	Leukemia, MESH:D015470	Calcitriol|D	21.6	16
GABRP	2568	Leukemia, MESH:D015473	Calcitriol|C	21.6	156
GLRB	2743	Leukemia, MESH:D015470	Cyclophos	21.6	14
GOT2	2806	Leukemia, MESH:D015473	Arsenic|Ar	21.6	265
GTF3C1	2975	Leukemia, MESH:D015470	Cyclophos	21.6	13
HAS3	3038	Leukemia, MESH:D015470	Arsenic Tr	21.6	14
MCAM	4162	Leukemia, MESH:D015470	Dasatinib|I	21.6	24
MEGF9	1955	Leukemia, MESH:D015470	Air Polluta	21.6	16
MYDGF	56005	Leukemia, MESH:D015470	Cytarabine	21.6	81
NDN	4692	Leukemia, MESH:D015470	Decitabine	21.6	20
NECAB2	54550	Leukemia, MESH:D015470	Arsenic Tr	21.6	20
NUDT21	11051	Leukemia, MESH:D015473	arsenite|D	21.6	158
NUP107	57122	Leukemia, MESH:D015470	Arsenic Tr	21.6	17
PAIP2B	400961	Leukemia, MESH:D015470	Dexameth	21.6	13
POLR2I	5438	Leukemia, MESH:D015470	Dexameth	21.6	14
PRRC1	133619	Leukemia, MESH:D015470	Arsenic Tr	21.6	14
SCUBE3	222663	Leukemia, MESH:D015470	Dexameth	21.6	12
SLITRK5	26050	Leukemia, MESH:D015470	Cytarabine	21.6	77
SMAD1	4086	Leukemia, MESH:D015473	Arsenic|Ar	21.6	265
TBC1D2B	23102	Leukemia, MESH:D015470	Air Polluta	21.6	22
TMEM154	201799	Leukemia, MESH:D015470	Air Polluta	21.6	19
TTYH1	57348	Leukemia, MESH:D015470	Calcitriol|C	21.6	77
UBALD2	283991	Leukemia, MESH:D015470	Dexameth	21.6	17
VWA5A	4013	Leukemia, MESH:D015473	Arsenic Tr	21.6	157
ADORA2B	136	Leukemia, MESH:D015470	Calcitriol|D	21.59	13
ANO2	57101	Leukemia, MESH:D015473	Dexameth	21.59	153
BCAR3	8412	Leukemia, MESH:D015470	Cytarabine	21.59	84
CEBPB	1051	Leukemia, MESH:D007948	2-(2-amin	21.59	3
CLDN5	7122	Leukemia, MESH:D015470	Arsenic Tr	21.59	23
LAMA5	3911	Leukemia, MESH:D015470	Calcitriol|C	21.59	23
MAPK10	5602	Leukemia, MESH:D015470	Bortezomi	21.59	19
SLC2A5	6518	Leukemia, MESH:D015470	Arsenic Tr	21.59	17
COL4A1	1282	Leukemia, MESH:D015473	Arsenic|ars	21.58	157
IGFBP2	3485	Leukemia, MESH:D015473	Arsenic Tr	21.58	267
AIFM1	9131	Leukemia, MESH:D015473	2-(2-chlor	21.56	263
RPSAP52	204010	Leukemia, MESH:D015470	Dasatinib|I	21.56	5
SP7	121340	Leukemia, MESH:D007948	2-(2-amin	21.56	3
ADAMTS4	9507	Leukemia, MESH:D015470	Arsenic Tr	21.55	21
ARAP2	116984	Leukemia, MESH:D015470	Calcitriol|D	21.55	19
CABLES1	91768	Leukemia, MESH:D015470	Decitabine	21.55	18
CDC42EP3	10602	Leukemia, MESH:D015470	Arsenic Tr	21.55	23
CHRNA5	1138	Leukemia, MESH:D015470	Dexameth	21.55	14
CIT	11113	Leukemia, MESH:D015470	Calcitriol|D	21.55	18
EOMES	8320	Leukemia, MESH:D015470	Air Polluta	21.55	13
EVL	51466	Leukemia, MESH:D015470	Arsenic Tr	21.55	22
FBN2	2201	Leukemia, MESH:D015473	Arsenic Tr	21.55	266
GAS2	2620	Leukemia, MESH:D015470	Arsenic Tr	21.55	15
HERC5	51191	Leukemia, MESH:D015470	Benzene|D	21.55	36
KLF3	51274	Leukemia, MESH:D015470	Air Polluta	21.55	16
PCDH17	27253	Leukemia, MESH:D015470	Arsenic Tr	21.55	21
RAD21	5885	Leukemia, MESH:D015470	Arsenic Tr	21.55	21

RPTOR	57521	Leukemia, MESH:D015470	Dexameth	21.55	11
RSRP1	57035	Leukemia, MESH:D015473	Arsenic|Ar	21.55	267
SHMT1	6470	Leukemia, MESH:D015473	Arsenic|Ar	21.55	264
SIVA1	10572	Leukemia, MESH:D015470	Air Polluta	21.55	13
SLC27A4	10999	Leukemia, MESH:D015470	Dronabinc	21.55	12
TRAF4	9618	Leukemia, MESH:D015470	Arsenic Tr	21.55	20
WNK1	65125	Leukemia, MESH:D015470	Dexameth	21.55	18
ENO2	2026	Leukemia, MESH:D015473	Arsenic|ars	21.54	160
F2R	2149	Leukemia, MESH:D015473	Arsenic|Ar	21.54	264
HAPLN1A	493635	Leukemia, MESH:D015470	Dexameth	21.54	6
MAP2	4133	Leukemia, MESH:D015473	Arsenic|Ar	21.54	267
TGFB3	7043	Leukemia, MESH:D015473	Arsenic|ars	21.54	161
DUT	1854	Leukemia, MESH:D015470	Calcitriol|D	21.53	13
FOXP1	27086	Leukemia, MESH:D015470	Dexameth	21.53	20
HNRNPH1	3187	Leukemia, MESH:D015470	Arsenic Tr	21.53	18
RACK1	10399	Leukemia, MESH:D015470	Decitabine	21.53	25
TLR1	7096	Leukemia, MESH:D007948	2-(2-amin	21.53	2
ZNF544	27300	Leukemia, MESH:D015470	Dexameth	21.53	20
CASP9	842	Leukemia, MESH:D004915	Daunorub	21.52	4
HOXC8	3224	Leukemia, MESH:D015470	Cytarabine	21.52	77
KANSL3	55683	Leukemia, MESH:D015470	Dexameth	21.52	11
NRAP	4892	Leukemia, MESH:D015473	Daunorub	21.52	20
NUDT17	200035	Leukemia, MESH:D015470	Doxorubic	21.52	12
PPARG	5468	Leukemia, MESH:D007948	2-(2-amin	21.52	4
ALMS1	7840	Leukemia, MESH:D015473	arsenite|Ca	21.51	158
CEP152	22995	Leukemia, MESH:D015473	arsenite|Ca	21.51	157
IGIP	492311	Leukemia, MESH:D015470	Dexameth	21.51	13
KLK6	5653	Leukemia, MESH:D015473	Arsenic|Ca	21.51	158
LHX1	3975	Leukemia, MESH:D015473	arsenite|Ca	21.51	153
RABL2A	11159	Leukemia, MESH:D015470	Dexameth	21.51	12
RFWD3	55159	Leukemia, MESH:D015473	arsenite|Ca	21.51	154
ACADM	34	Leukemia, MESH:D015473	Calcitriol|C	21.5	156
ASIC2	40	Leukemia, MESH:D015473	Arsenic|ars	21.5	157
CMSS1	84319	Leukemia, MESH:D015473	Arsenic|ars	21.5	159
FAM171B	165215	Leukemia, MESH:D015473	Arsenic|ars	21.5	15
ITPRID2	6744	Leukemia, MESH:D015473	Arsenic Tr	21.5	263
PDGFRA	5156	Leukemia, MESH:D015473	Arsenic|ars	21.5	158
PRKAR1B	5575	Leukemia, MESH:D015473	Arsenic|Ar	21.5	263
ALDH1L2	160428	Leukemia, MESH:D015473	Arsenic|ars	21.49	160
EPHA4	2043	Leukemia, MESH:D015473	Arsenic|ars	21.49	160
MTHFD1L	25902	Leukemia, MESH:D015473	Arsenic|Ar	21.49	264
TAC1	6863	Leukemia, MESH:D015470	Benzene|C	21.48	98
ABHD14A	25864	Leukemia, MESH:D015470	Air Polluta	21.47	13
FAXDC2	10826	Leukemia, MESH:D015470	Air Polluta	21.47	20
H2AC18	8337	Leukemia, MESH:D015470	Benzene|D	21.47	36
KMT2B	9757	Leukemia, MESH:D015470	Benzene|D	21.47	36
MELTF	4241	Leukemia, MESH:D015470	Benzene|C	21.47	28
MRPS17	51373	Leukemia, MESH:D015470	Dexameth	21.47	12
MRPS18A	55168	Leukemia, MESH:D015470	Decitabine	21.47	21
PDCL3	79031	Leukemia, MESH:D015470	Arsenic Tr	21.47	28
PLEKHM1	9842	Leukemia, MESH:D015470	Benzene|D	21.47	23
SASS6	163786	Leukemia, MESH:D015470	Calcitriol|I	21.47	9
TPPP	11076	Leukemia, MESH:D015470	Doxorubic	21.47	13
C1QTNF5	114902	Leukemia, MESH:D015470	Dexameth	21.46	14
CENPI	2491	Leukemia, MESH:D015470	Calcitriol|D	21.46	15
CHAC1	79094	Leukemia, MESH:D015473	Arsenic|Ar	21.46	162
CTSA	5476	Leukemia, MESH:D015470	Arsenic Tr	21.46	21
DPYSL2	1808	Leukemia, MESH:D015470	Arsenic Tr	21.46	30

EIF2AK2	5610	Leukemia, MESH:D015470	Arsenic Tr	21.46	29
GPAT3	84803	Leukemia, MESH:D015470	Air Polluta	21.46	13
GPX8	493869	Leukemia, MESH:D015470	Calcitriol|D	21.46	12
HES5	388585	Leukemia, MESH:D015470	2-(2-chlor	21.46	82
HS3ST1	9957	Leukemia, MESH:D015470	Cytarabine	21.46	88
KCNJ11	3767	Leukemia, MESH:D015470	Dexameth	21.46	15
MED14	9282	Leukemia, MESH:D015470	Calcitriol|D	21.46	14
NDUFB8	4714	Leukemia, MESH:D015470	Deferoxan	21.46	14
NRIP3	56675	Leukemia, MESH:D015470	Arsenic Tr	21.46	96
PHC2	1912	Leukemia, MESH:D015470	Dexameth	21.46	13
PLA2G15	23659	Leukemia, MESH:D015470	Dexameth	21.46	13
PPP2R5C	5527	Leukemia, MESH:D015470	Air Polluta	21.46	16
RDH11	51109	Leukemia, MESH:D015470	Bortezomi	21.46	16
SDS	10993	Leukemia, MESH:D015470	Benzene|C	21.46	28
THBS4	7060	Leukemia, MESH:D015470	Decitabine	21.46	32
TNFSF14	8740	Leukemia, MESH:D015470	Arsenic Tr	21.46	38
WHAMM	123720	Leukemia, MESH:D015470	Air Polluta	21.46	14
ADORA2A	135	Leukemia, MESH:D015470	Bezafibrat	21.45	23
ANK1	286	Leukemia, MESH:D015470	Calcitriol|D	21.45	23
ARG2	384	Leukemia, MESH:D015470	Cytarabine	21.45	87
ATP6V0D1	9114	Leukemia, MESH:D015470	Arsenic Tr	21.45	20
COX6B1	1340	Leukemia, MESH:D015470	Dexameth	21.45	18
CYP17A1	1586	Leukemia, MESH:D015473	Arsenic|Ar	21.45	265
EEF1B2	1933	Leukemia, MESH:D015470	Arsenic Tr	21.45	20
EFHD2	79180	Leukemia, MESH:D015470	Dexameth	21.45	17
FLI1	2313	Leukemia, MESH:D015470	Dexameth	21.45	15
FRMD4A	55691	Leukemia, MESH:D015470	Dexameth	21.45	15
HLA-DRB5	3127	Leukemia, MESH:D015473	Arsenic|Cy	21.45	24
MPDZ	8777	Leukemia, MESH:D015470	Dexameth	21.45	15
PLXNC1	10154	Leukemia, MESH:D015470	Arsenic Tr	21.45	16
BSCL2	26580	Leukemia, MESH:D004915	Cytarabine	21.44	3
POMC	5443	Leukemia, MESH:D015470	Bezafibrat	21.44	20
COL6A2	1292	Leukemia, MESH:D015473	arsenite|Cy	21.43	160
RFC4	5984	Leukemia, MESH:D015473	Calcitriol|D	21.43	156
TNFRSF25	8718	Leukemia, MESH:D015473	Arsenic|Ar	21.43	264
AGPS	8540	Leukemia, MESH:D015473	Arsenic Tr	21.42	263
MARCHF3	115123	Leukemia, MESH:D015473	Arsenic Tr	21.42	163
ABHD11	83451	Leukemia, MESH:D015473	Dexameth	21.41	156
BID	637	Leukemia, MESH:D007948	Arsenic Tr	21.41	4
INS2	16334	Leukemia, MESH:D015473	Arsenic|Ar	21.41	159
MICB	4277	Leukemia, MESH:D015473	Arsenic|Ar	21.41	264
SURF1	6834	Leukemia, MESH:D015473	arsenite|D	21.41	161
TMEM38B	55151	Leukemia, MESH:D015473	Arsenic|ars	21.41	158
TP73	7161	Leukemia, MESH:D007948	2-(2-amin	21.41	3
RPS29	6235	Leukemia, MESH:D015473	Arsenic|Ar	21.4	265
SERPINB4	6318	Leukemia, MESH:D015473	Arsenic|ars	21.4	156
LTF	4057	Leukemia, MESH:D015470	Calcitriol|C	21.39	15
CKS1B	1163	Leukemia, MESH:D015473	arsenite|Ca	21.38	156
NCAPH	23397	Leukemia, MESH:D015473	Arsenic Tr	21.38	262
SCD1	20249	Leukemia, MESH:D015473	Arsenic Tr	21.38	261
CDCA2	157313	Leukemia, MESH:D015473	arsenite|Ca	21.37	158
CIITA	4261	Leukemia, MESH:D015470	Alitretinoi	21.37	23
COX2	4513	Leukemia, MESH:D015473	Arsenic|ars	21.37	160
EFNB2	1948	Leukemia, MESH:D015473	Arsenic|ars	21.37	160
GRPEL1	80273	Leukemia, MESH:D015470	Doxorubic	21.37	22
NIBAN1	116496	Leukemia, MESH:D015473	Arsenic Tr	21.37	261
RRP12	23223	Leukemia, MESH:D015470	Decitabine	21.37	19
ANO1	55107	Leukemia, MESH:D015470	Dexameth	21.36	16

B4GALT5	9334	Leukemia, MESH:D015470	Arsenic Tr	21.36	20
DHX58	79132	Leukemia, MESH:D015470	Dexameth	21.36	15
DNM2	1785	Leukemia, MESH:D015470	Arsenic Tr	21.36	22
ENDOD1	23052	Leukemia, MESH:D015470	Dexameth	21.36	15
EPB41L5	57669	Leukemia, MESH:D015470	Ethylnitros	21.36	12
FAM107B	83641	Leukemia, MESH:D015470	Arsenic Tr	21.36	18
FRAS1	80144	Leukemia, MESH:D015470	Calcitriol|D	21.36	10
LPAR6	10161	Leukemia, MESH:D015470	Air Polluta	21.36	15
LPGAT1	9926	Leukemia, MESH:D015470	Calcitriol|D	21.36	12
MUC2	4583	Leukemia, MESH:D015470	Dronabinc	21.36	13
NDUFV2	4729	Leukemia, MESH:D015470	Benzene|D	21.36	29
NRGN	4900	Leukemia, MESH:D015470	Air Polluta	21.36	10
PAPOLA	10914	Leukemia, MESH:D015470	Bortezomi	21.36	13
PRXL2A	84293	Leukemia, MESH:D015470	Dexameth	21.36	15
SMOC2	64094	Leukemia, MESH:D015470	Cytarabine	21.36	85
SPTLC2	9517	Leukemia, MESH:D015470	Arsenic Tr	21.36	17
STEAP3	55240	Leukemia, MESH:D015470	Calcitriol|D	21.36	10
TFAP2C	7022	Leukemia, MESH:D015470	Arsenic Tr	21.36	24
VAMP8	8673	Leukemia, MESH:D015470	Benzene|D	21.36	30
APP	351	Leukemia, MESH:D015473	alpha-Toc	21.35	265
MIR150	406942	Leukemia, MESH:D015470	Air Polluta	21.34	32
NUDT12	83594	Leukemia, MESH:D015470	Arsenic Tr	21.34	17
STAR	6770	Leukemia, MESH:D007948	2-(2-amin	21.34	3
TDGF1	6997	Leukemia, MESH:D015470	Bortezomi	21.34	10
TLR21	402884	Leukemia, MESH:D007948	2-(2-amin	21.34	1
XPNPEP3	63929	Leukemia, MESH:D015470	Bortezomi	21.34	18
ANGPTL1	9068	Leukemia, MESH:D015470	Arsenic Tr	21.33	20
ARL8A	127829	Leukemia, MESH:D015470	Bortezomi	21.33	16
CNPY2	10330	Leukemia, MESH:D015470	Doxorubic	21.33	14
CSTF1	1477	Leukemia, MESH:D015470	Dexameth	21.33	17
EGF	1950	Leukemia, MESH:D007948	2-(2-amin	21.33	3
GNPTAB	79158	Leukemia, MESH:D015470	Dexameth	21.33	13
HAND2	9464	Leukemia, MESH:D015473	arsenite|C	21.33	159
HSPBP1	23640	Leukemia, MESH:D015470	Dexameth	21.33	13
KCNJ13	3769	Leukemia, MESH:D015470	Decitabine	21.33	20
KCTD10	83892	Leukemia, MESH:D015470	Bortezomi	21.33	16
MED20	9477	Leukemia, MESH:D015470	Air Polluta	21.33	13
MIB1	57534	Leukemia, MESH:D015470	Calcitriol|D	21.33	14
NADK	65220	Leukemia, MESH:D015470	Air Polluta	21.33	15
NDRG3	57446	Leukemia, MESH:D015470	Air Polluta	21.33	16
NSD3	54904	Leukemia, MESH:D015470	Arsenic Tr	21.33	16
PTPMT1	114971	Leukemia, MESH:D015470	Dexameth	21.33	15
RAI2	10742	Leukemia, MESH:D015470	Calcitriol|D	21.33	14
RNF114	55905	Leukemia, MESH:D015470	Dexameth	21.33	15
RTTN	25914	Leukemia, MESH:D015470	Calcitriol|D	21.33	14
SERTAD3	29946	Leukemia, MESH:D015470	Dexameth	21.33	12
TMED4	222068	Leukemia, MESH:D015470	Dexameth	21.33	14
TOMM7	54543	Leukemia, MESH:D015470	Arsenic Tr	21.33	21
TRIM26	7726	Leukemia, MESH:D015470	Arsenic Tr	21.33	16
ADRA2A	150	Leukemia, MESH:D015470	Arsenic Tr	21.32	22
ATP2B4	493	Leukemia, MESH:D015473	Arsenic|ar	21.32	158
FOXP3	50943	Leukemia, MESH:D015473	Arsenic|Ar	21.32	164
HSP90AA1	3320	Leukemia, MESH:D015473	Arsenic|Ar	21.32	163
KAT5	10524	Leukemia, MESH:D015473	Arsenic Tr	21.32	265
PNPLA2	57104	Leukemia, MESH:D015470	Dexameth	21.32	17
RAD51AP1	10635	Leukemia, MESH:D015470	Calcitriol|D	21.32	18
SCHIP1	29970	Leukemia, MESH:D015473	Arsenic|De	21.32	158
SMPD3	55512	Leukemia, MESH:D015470	Arsenic Tr	21.32	50

UBE2T	29089	Leukemia, MESH:D015473	Calcitriol|[illegible]	21.32	156
VPS13D	55187	Leukemia, MESH:D015473	Arsenic|ars	21.32	156
GPR37	2861	Leukemia, MESH:D015473	Arsenic Tr	21.31	262
HLA-C	3107	Leukemia, MESH:D015473	Arsenic|Ar	21.31	267
NKD2	85409	Leukemia, MESH:D015473	Arsenic|Ar	21.31	262
RMI2	116028	Leukemia, MESH:D015473	Arsenic Tr	21.31	262
SKA2	348235	Leukemia, MESH:D015473	arsenite|C	21.31	156
ADGRE2	30817	Leukemia, MESH:D015470	Air Polluta	21.3	10
CDK5	1020	Leukemia, MESH:D007948	2-(2-amin	21.3	3
NABP2	79035	Leukemia, MESH:D015470	Dexameth	21.3	15
SIMC1	375484	Leukemia, MESH:D015470	Doxorubic	21.3	14
UGT1A6	54578	Leukemia, MESH:D015470	Alitretinoi	21.3	36
ZNF598	90850	Leukemia, MESH:D015470	Dexameth	21.3	11
ZNF606	80095	Leukemia, MESH:D015470	Doxorubic	21.3	13
ZSWIM3	140831	Leukemia, MESH:D015470	Dexameth	21.3	12
ZSWIM8	23053	Leukemia, MESH:D015470	Dexameth	21.3	14
AKT1	207	Leukemia, MESH:D004915	Daunorub	21.29	4
CYP17A1	1586	Leukemia, MESH:D007948	2-(2-amin	21.29	3
FMO1	2326	Leukemia, MESH:D015470	Cytarabine	21.29	83
UCP2	7351	Leukemia, MESH:D015473	Arsenic Tr	21.29	263
ABL2	27	Leukemia, MESH:D015470	Dasatinib|	21.28	14
AMH	268	Leukemia, MESH:D015470	Decitabine	21.28	21
HTT	3064	Leukemia, MESH:D015470	Dexameth	21.28	9
IVL	3713	Leukemia, MESH:D015470	Alitretinoi	21.28	18
JAM2	58494	Leukemia, MESH:D015470	Arsenic Tr	21.28	90
NFATC3	4775	Leukemia, MESH:D015470	Arsenic Tr	21.28	38
PIDD1	55367	Leukemia, MESH:D015470	Calcitriol|H	21.28	14
ARHGEF3	50650	Leukemia, MESH:D015470	Air Polluta	21.27	82
CNN2	1265	Leukemia, MESH:D015470	Calcitriol|[illegible]	21.27	15
DPYSL4	10570	Leukemia, MESH:D015470	Cyclophos	21.27	22
IFNGR2	3460	Leukemia, MESH:D015470	Arsenic Tr	21.27	34
NEXN	91624	Leukemia, MESH:D015470	Calcitriol|[illegible]	21.27	16
PIM3	415116	Leukemia, MESH:D015470	Air Polluta	21.27	16
RNASEH2	10535	Leukemia, MESH:D015470	Arsenic Tr	21.27	18
TFDP2	7029	Leukemia, MESH:D015470	Air Polluta	21.27	15
TYRP1	7306	Leukemia, MESH:D015470	Doxorubic	21.27	24
ZWILCH	55055	Leukemia, MESH:D015470	Calcitriol|[illegible]	21.27	13
ASF1B	55723	Leukemia, MESH:D015473	arsenite|C	21.26	157
ATP2B1	490	Leukemia, MESH:D015470	Arsenic Tr	21.26	18
CRY1	1407	Leukemia, MESH:D015470	Air Polluta	21.26	26
GPD2	2820	Leukemia, MESH:D015473	Arsenic|Ar	21.26	163
GTSE1	51512	Leukemia, MESH:D015473	Arsenic|Ca	21.26	159
HNRNPD	3184	Leukemia, MESH:D015473	Daunorub	21.26	161
RETN	56729	Leukemia, MESH:D015470	Alitretinoi	21.26	22
SAA1	6288	Leukemia, MESH:D015473	Arsenic|Ca	21.26	159
TUBA1C	84790	Leukemia, MESH:D015470	Dexameth	21.26	20
GPX2	2877	Leukemia, MESH:D015470	Alitretinoi	21.25	19
IGH	3492	Leukemia, MESH:D015473	Arsenic|Da	21.25	153
DSN1	79980	Leukemia, MESH:D015473	arsenite|D	21.23	156
HPRT1	3251	Leukemia, MESH:D015473	arsenite|C	21.23	157
IKZF2	22807	Leukemia, MESH:D015473	Antimony	21.23	154
LCP2	3937	Leukemia, MESH:D015473	Genistein|	21.23	157
SBSPON	157869	Leukemia, MESH:D015473	Cytarabine	21.23	154
SLC25A11	8402	Leukemia, MESH:D015473	arsenite|M	21.23	159
WNT2B	7482	Leukemia, MESH:D015473	Arsenic|ars	21.23	156
BTN3A3	10384	Leukemia, MESH:D015473	Arsenic|Ar	21.22	264
ISOC1	51015	Leukemia, MESH:D015473	Arsenic|De	21.22	158
LMOD1	25802	Leukemia, MESH:D015473	Arsenic Tr	21.22	260

PPP2R5A	5525	Leukemia, MESH:D015473	Arsenic Tr	21.22	262
SLC6A14	11254	Leukemia, MESH:D015473	Arsenic|De	21.22	12
TTF2	8458	Leukemia, MESH:D015473	Dexameth	21.22	155
CYP4F3	4051	Leukemia, MESH:D015470	Benzene|N	21.21	27
HIKESHI	51501	Leukemia, MESH:D015470	Bortezomi	21.21	15
IFI44	10561	Leukemia, MESH:D015473	arsenite|Cl	21.21	154
MYT1	4661	Leukemia, MESH:D015470	Dexameth	21.21	22
PACSIN1	29993	Leukemia, MESH:D015470	Bortezomi	21.21	13
POLE2	5427	Leukemia, MESH:D015473	arsenite|Ca	21.21	157
SORT1	6272	Leukemia, MESH:D015473	Cholestero	21.21	152
TRIM32	22954	Leukemia, MESH:D015470	Air Polluta	21.21	8
AATK	9625	Leukemia, MESH:D015470	Dexameth	21.2	16
ACOT11	26027	Leukemia, MESH:D015470	Calcitriol|D	21.2	15
ALYREF	10189	Leukemia, MESH:D015470	Cyclophos	21.2	11
CDKL5	6792	Leukemia, MESH:D015470	Doxorubic	21.2	15
CEP290	80184	Leukemia, MESH:D015470	Bortezomi	21.2	10
DCAF7	10238	Leukemia, MESH:D015470	Air Polluta	21.2	9
H2AC20	8338	Leukemia, MESH:D015470	Arsenic Tr	21.2	20
HPS3	84343	Leukemia, MESH:D015470	Air Polluta	21.2	19
ILDR2	387597	Leukemia, MESH:D015470	Doxorubic	21.2	19
MARK1	4139	Leukemia, MESH:D015470	Alitretinoi	21.2	19
MPHOSPH	10198	Leukemia, MESH:D015470	Air Polluta	21.2	15
MRPL24	79590	Leukemia, MESH:D015470	Arsenic Tr	21.2	16
MTA3	57504	Leukemia, MESH:D015470	Dronabinc	21.2	12
NINJ2	4815	Leukemia, MESH:D015470	Arsenic Tr	21.2	14
PAICS	10606	Leukemia, MESH:D015473	Arsenic|Ar	21.2	264
PAQR5	54852	Leukemia, MESH:D015470	Calcitriol|D	21.2	14
PIK3R3	8503	Leukemia, MESH:D015473	Arsenic|ars	21.2	160
PPM1E	22843	Leukemia, MESH:D015470	Dexameth	21.2	11
PWP2	5822	Leukemia, MESH:D015470	Dronabinc	21.2	11
RNF115	27246	Leukemia, MESH:D015470	Dexameth	21.2	14
SEPSECS	51091	Leukemia, MESH:D015470	Arsenic Tr	21.2	15
THEM4	117145	Leukemia, MESH:D015470	Doxorubic	21.2	14
TMEM177	80775	Leukemia, MESH:D015470	Arsenic Tr	21.2	18
TSHZ2	128553	Leukemia, MESH:D015470	Dexameth	21.2	16
WASF3	10810	Leukemia, MESH:D015470	Benzene|D	21.2	28
WBP1	23559	Leukemia, MESH:D015470	Dexameth	21.2	9
YTHDF2	51441	Leukemia, MESH:D015470	Arsenic Tr	21.2	17
ZFYVE1	53349	Leukemia, MESH:D015470	Dexameth	21.2	15
AGL	178	Leukemia, MESH:D015470	Air Polluta	21.19	20
ATR	545	Leukemia, MESH:D015470	Arsenic Tr	21.19	38
GLDC	2731	Leukemia, MESH:D015470	Decitabine	21.19	25
MAGI1	9223	Leukemia, MESH:D015470	Calcitriol|C	21.19	82
NOG	9241	Leukemia, MESH:D015470	Decitabine	21.19	18
PARK7	11315	Leukemia, MESH:D015470	15-deoxy-	21.19	28
PNISR	25957	Leukemia, MESH:D015470	Bortezomi	21.19	19
RBPJ	3516	Leukemia, MESH:D015470	Benzene|C	21.19	32
REL	5966	Leukemia, MESH:D015470	Arsenic Tr	21.19	25
SGO1	151648	Leukemia, MESH:D015470	Calcitriol|D	21.19	13
SLC1A2	6506	Leukemia, MESH:D015473	alpha-Toc	21.19	263
SMPDL3A	10924	Leukemia, MESH:D015470	Arsenic Tr	21.19	84
TTC3	7267	Leukemia, MESH:D015470	Decitabine	21.19	24
YPEL2	388403	Leukemia, MESH:D015470	Air Polluta	21.19	18
ZFHX3	463	Leukemia, MESH:D015470	Doxorubic	21.18	16
MAGEA6	4105	Leukemia, MESH:D015470	Decitabine	21.16	32
CSRP2	1466	Leukemia, MESH:D015473	Arsenic|Ar	21.15	265
IFITM3	10410	Leukemia, MESH:D015473	Arsenic Tr	21.15	261
KCNMA1	3778	Leukemia, MESH:D015473	Arsenic|Ar	21.15	263

TCF19	6941	Leukemia, MESH:D015473	Arsenic Tr	21.15	157
TUBB	203068	Leukemia, MESH:D015473	Arsenic|ars	21.15	14
CEBPZ	10153	Leukemia, MESH:D015473	Arsenic Tr	21.14	262
TM6SF1	53346	Leukemia, MESH:D015473	Arsenic|Ar	21.14	159
ART3	419	Leukemia, MESH:D015473	Arsenic|De	21.13	156
DMKN	93099	Leukemia, MESH:D015473	Arsenic|Ca	21.13	12
ID4	3400	Leukemia, MESH:D015470	Arsenic Tr	21.13	44
NUP37	79023	Leukemia, MESH:D015473	Arsenic Tr	21.13	263
OSBPL8	114882	Leukemia, MESH:D015473	Arsenic|Ar	21.13	263
RNASEH2I	79621	Leukemia, MESH:D015473	Arsenic|Ca	21.13	157
TACC3	10460	Leukemia, MESH:D015470	Calcitriol|D	21.13	14
TBC1D5	9779	Leukemia, MESH:D015473	arsenite|D	21.13	161
TCEA2	6919	Leukemia, MESH:D015473	Arsenic|Ca	21.13	11
ABCB1B	18669	Leukemia, MESH:D015473	Dexameth	21.12	156
OR52K3P	390035	Leukemia, MESH:D015470	Air Polluta	21.12	12
MZT2A	653784	Leukemia, MESH:D015470	Doxorubic	21.11	11
ALOX5AP	241	Leukemia, MESH:D015473	Arsenic Tr	21.1	266
C6ORF62	81688	Leukemia, MESH:D015470	Azacitidine	21.1	15
CAVIN1	284119	Leukemia, MESH:D015470	Benzene|C	21.1	95
CPD	1362	Leukemia, MESH:D015470	Calcitriol|D	21.1	16
DBT	1629	Leukemia, MESH:D015470	Arsenic Tr	21.1	22
MAP2K5	5607	Leukemia, MESH:D015470	Azacitidine	21.1	14
REEP5	7905	Leukemia, MESH:D015470	Dexameth	21.1	12
RRM2B	50484	Leukemia, MESH:D015473	Arsenic|ars	21.1	15
SEMA3B	7869	Leukemia, MESH:D015470	Calcitriol|D	21.1	14
SLC41A2	84102	Leukemia, MESH:D015470	Calcitriol|D	21.1	16
ST13	6767	Leukemia, MESH:D015470	Arsenic Tr	21.1	21
STK39	27347	Leukemia, MESH:D015470	Calcitriol|C	21.1	17
STMN2	11075	Leukemia, MESH:D015470	Decitabine	21.1	23
TBL1Y	90665	Leukemia, MESH:D015470	Dexameth	21.1	8
TNPO1	3842	Leukemia, MESH:D015470	Calcitriol|F	21.1	13
UAP1	6675	Leukemia, MESH:D015470	Arsenic Tr	21.1	35
ZNF32	7580	Leukemia, MESH:D015470	Dexameth	21.1	19
CD1D	912	Leukemia, MESH:D015470	Arsenic Tr	21.09	15
HDAC2	3066	Leukemia, MESH:D015473	Arsenic Tr	21.09	263
RNF166	115992	Leukemia, MESH:D015470	Doxorubic	21.09	12
TMEM132	114795	Leukemia, MESH:D015470	Decitabine	21.09	15
AGO1	26523	Leukemia, MESH:D015470	Benzene|D	21.08	24
ATP6V0A4	50617	Leukemia, MESH:D015470	Dronabinc	21.08	16
ATP6V1F	9296	Leukemia, MESH:D015470	Doxorubic	21.08	14
BRINP1	1620	Leukemia, MESH:D015470	Arsenic Tr	21.08	20
BTAF1	9044	Leukemia, MESH:D015470	Dexameth	21.08	13
CA2	760	Leukemia, MESH:D015473	Calcitriol|D	21.08	157
CD5	921	Leukemia, MESH:D015470	Benzene|B	21.08	23
CERS1	10715	Leukemia, MESH:D015470	Dexameth	21.08	10
CFAP97	57587	Leukemia, MESH:D015470	Doxorubic	21.08	13
FOXK2	3607	Leukemia, MESH:D015470	Doxorubic	21.08	18
GIPC1	10755	Leukemia, MESH:D015470	Arsenic Tr	21.08	16
GTPBP1	9567	Leukemia, MESH:D015470	Bortezomi	21.08	88
MMS22L	253714	Leukemia, MESH:D015470	Calcitriol|D	21.08	10
NTPCR	84284	Leukemia, MESH:D015470	Doxorubic	21.08	15
NUFIP2	57532	Leukemia, MESH:D015470	Doxorubic	21.08	15
PCDH19	57526	Leukemia, MESH:D015470	Arsenic Tr	21.08	23
PRRC2B	84726	Leukemia, MESH:D015470	Dexameth	21.08	10
PSAT1	29968	Leukemia, MESH:D015473	Arsenic Tr	21.08	261
PSKH1	5681	Leukemia, MESH:D015470	Dexameth	21.08	12
TEX30	93081	Leukemia, MESH:D015470	Benzene|C	21.08	24
TSPYL2	64061	Leukemia, MESH:D015470	Air Polluta	21.08	19

VDAC3	7419	Leukemia, MESH:D015470	Dexameth	21.08	16
WASF1	8936	Leukemia, MESH:D015470	Clioquinol	21.08	14
ZC3H4	23211	Leukemia, MESH:D015470	Decitabine	21.08	15
ZNF287	57336	Leukemia, MESH:D015470	Dexameth	21.08	7
ARF5	381	Leukemia, MESH:D015470	Dexameth	21.07	14
CNOT4	4850	Leukemia, MESH:D015470	Dexameth	21.07	14
EPX	8288	Leukemia, MESH:D015470	Arsenic Tr	21.07	16
MBOAT7	79143	Leukemia, MESH:D015470	Dexameth	21.07	11
PGGT1B	5229	Leukemia, MESH:D015470	Arsenic Tr	21.07	21
PPP1R1C	151242	Leukemia, MESH:D015470	Calcitriol|D	21.07	13
RIN3	79890	Leukemia, MESH:D015470	Calcitriol|D	21.07	11
RTKN2	219790	Leukemia, MESH:D015470	Calcitriol|I	21.07	11
SGTB	54557	Leukemia, MESH:D015470	Arsenic Tr	21.07	14
TBC1D9	23158	Leukemia, MESH:D015470	Bortezomi	21.07	15
YOD1	55432	Leukemia, MESH:D015470	Doxorubic	21.07	13
CCR7	1236	Leukemia, MESH:D015470	Alitretinoi	21.06	31
SCARNA2	677681	Leukemia, MESH:D015470	Doxorubic	21.06	10
SERPINC1	462	Leukemia, MESH:D015470	Bezafibrat	21.06	13
SERPING1	710	Leukemia, MESH:D015470	Air Polluta	21.06	17
TRP53	22059	Leukemia, MESH:D007948	2-(2-amin	21.06	4
FUT4	2526	Leukemia, MESH:D015473	Arsenic|Ar	21.05	268
TAF15	8148	Leukemia, MESH:D015473	Arsenic|Ar	21.05	267
TERF1	7013	Leukemia, MESH:D015473	Arsenic Tr	21.05	261
YWHAE	7531	Leukemia, MESH:D015473	Arsenic|ars	21.05	17
ATG4B	23192	Leukemia, MESH:D015473	Arsenic|Ar	21.04	161
CDK2	1017	Leukemia, MESH:D007948	2-(2-amin	21.04	3
CGA	1081	Leukemia, MESH:D015473	2-(2-chlor	21.04	157
CPE	1363	Leukemia, MESH:D015473	Arsenic|Ar	21.04	262
LRRK1	79705	Leukemia, MESH:D015473	Arsenic|Ar	21.04	266
MAX	4149	Leukemia, MESH:D015473	Arsenic Tr	21.04	260
NOVA1	4857	Leukemia, MESH:D015473	Arsenic Tr	21.04	164
RARB	5915	Leukemia, MESH:D015473	2-(2-chlor	21.04	267
SLC2A5	6518	Leukemia, MESH:D015473	Arsenic|Ar	21.04	268
ZNF383	163087	Leukemia, MESH:D015473	Arsenic Tr	21.04	263
MAPK9	5601	Leukemia, MESH:D007948	2-(2-amin	21.02	3
DYSF	8291	Leukemia, MESH:D015470	Calcitriol|D	21.01	22
EIF3B	8662	Leukemia, MESH:D015470	Benzene|C	21.01	32
GTPBP2	54676	Leukemia, MESH:D015470	Decitabine	21.01	23
ICAM2	3384	Leukemia, MESH:D015470	Air Polluta	21.01	17
MEG3	55384	Leukemia, MESH:D015470	Arsenic Tr	21.01	26
MIR34A	407040	Leukemia, MESH:D015470	Benzene|D	21.01	34
NCOA4	8031	Leukemia, MESH:D015470	Arsenic Tr	21.01	20
PDP1	54704	Leukemia, MESH:D015470	Calcitriol|D	21.01	10
TFE3	7030	Leukemia, MESH:D015470	Azacitidine	21.01	83
TNFRSF11	8792	Leukemia, MESH:D015470	Arsenic Tr	21.01	17
TRIM25	7706	Leukemia, MESH:D015470	Arsenic Tr	21.01	20
AGTR1	185	Leukemia, MESH:D015470	Arsenic Tr	21	30
CASP2	835	Leukemia, MESH:D015473	Arsenic|Ar	21	166
CFH	3075	Leukemia, MESH:D015470	Doxorubic	21	17
CKM	1158	Leukemia, MESH:D015470	Allopurinc	21	58
FGF1	2246	Leukemia, MESH:D015473	Arsenic|ars	21	265
JUND	3727	Leukemia, MESH:D015473	alpha-Toc	21	265
TNC	3371	Leukemia, MESH:D015473	Arsenic|ars	21	159
TST	7263	Leukemia, MESH:D015470	Cytarabine	21	82
BAMBI	25805	Leukemia, MESH:D015473	Arsenic Tr	20.99	263
EIF4G1	1981	Leukemia, MESH:D007948	2-(2-amin	20.99	3
MALAT1	378938	Leukemia, MESH:D015473	Arsenic|Ar	20.98	266
AURKB	9212	Leukemia, MESH:D015473	arsenite|Ca	20.97	158

FMO4	2329	Leukemia, MESH:D015473		Calcitriol|(	20.96	159
NEO1	4756	Leukemia, MESH:D015473		Arsenic Tr	20.96	261
POLR1H	30834	Leukemia, MESH:D015473		arsenite|Et	20.96	11
PPP1R13L	10848	Leukemia, MESH:D015473		Arsenic|Da	20.96	160
PSMD13	5719	Leukemia, MESH:D015473		Arsenic|Ar	20.96	265
RPS21	6227	Leukemia, MESH:D015473		Arsenic Tr	20.96	156
YKT6	10652	Leukemia, MESH:D015473		Arsenic|Ar	20.96	267
ARGLU1	55082	Leukemia, MESH:D015470		Dexameth	20.95	11
CCDC18	343099	Leukemia, MESH:D015470		Calcitriol|D	20.95	15
CDH23	64072	Leukemia, MESH:D015470		Arsenic Tr	20.95	20
CNOT6L	246175	Leukemia, MESH:D015470		Arsenic Tr	20.95	19
CREB3L4	148327	Leukemia, MESH:D015470		Azacitidine	20.95	15
CTNNBIP1	56998	Leukemia, MESH:D015470		Cyclophos	20.95	14
DISP1	84976	Leukemia, MESH:D015470		Calcitriol|D	20.95	13
EIF5A2	56648	Leukemia, MESH:D015473		Arsenic|Ar	20.95	264
FES	2242	Leukemia, MESH:D015470		Dexameth	20.95	11
GPBAR1	151306	Leukemia, MESH:D015470		Air Polluta	20.95	10
GPX1A	352926	Leukemia, MESH:D015470		Arsenic Tr	20.95	17
HDAC10	83933	Leukemia, MESH:D015470		Bortezomi	20.95	10
HLA-F	3134	Leukemia, MESH:D015470		Arsenic Tr	20.95	16
ITGBL1	9358	Leukemia, MESH:D015470		Arsenic Tr	20.95	21
KANSL2	54934	Leukemia, MESH:D015470		Dexameth	20.95	13
LDB2	9079	Leukemia, MESH:D015470		Arsenic Tr	20.95	20
MOSPD2	158747	Leukemia, MESH:D015470		Air Polluta	20.95	20
NDUFA4	4697	Leukemia, MESH:D015473		Arsenic Tr	20.95	263
NSMAF	8439	Leukemia, MESH:D015470		Benzene|D	20.95	27
PARP3	10039	Leukemia, MESH:D015470		Dexameth	20.95	9
PAX5	5079	Leukemia, MESH:D015470		Arsenic Tr	20.95	14
PAX7	5081	Leukemia, MESH:D015470		Dexameth	20.95	9
PKN2	5586	Leukemia, MESH:D015470		Dexameth	20.95	15
PLEKHG4	25894	Leukemia, MESH:D015470		Decitabine	20.95	16
PRRG1	5638	Leukemia, MESH:D015470		Arsenic Tr	20.95	20
PUM3	9933	Leukemia, MESH:D015470		Dexameth	20.95	14
RAVER2	55225	Leukemia, MESH:D015470		Dasatinib|I	20.95	14
RBFOX3	146713	Leukemia, MESH:D015473		Arsenic|Ge	20.95	157
SAP30	8819	Leukemia, MESH:D015470		Dexameth	20.95	10
SLC25A32	81034	Leukemia, MESH:D015470		Dexameth	20.95	14
SOX10	6663	Leukemia, MESH:D015470		Bortezomi	20.95	18
TM7SF3	51768	Leukemia, MESH:D015470		Dexameth	20.95	21
TMEM44	93109	Leukemia, MESH:D015470		Decitabine	20.95	15
TMEM63A	9725	Leukemia, MESH:D015470		Dexameth	20.95	14
TOR1AIP2	163590	Leukemia, MESH:D015470		Air Polluta	20.95	8
ZFP91	80829	Leukemia, MESH:D015470		Dexameth	20.95	14
ZMYM2	7750	Leukemia, MESH:D015473		Arsenic Tr	20.95	263
EZR	7430	Leukemia, MESH:D015470		Arsenic Tr	20.94	90
ADGRE1	2015	Leukemia, MESH:D015473		Arsenic|Ar	20.93	269
BAG1	573	Leukemia, MESH:D015470		alvocidib|A	20.93	35
DOCK8	81704	Leukemia, MESH:D015470		Air Polluta	20.93	19
HMGCS1	3157	Leukemia, MESH:D015473		Arsenic Tr	20.93	268
MYADM	91663	Leukemia, MESH:D015470		Air Polluta	20.93	78
NEDD4L	23327	Leukemia, MESH:D015470		Decitabine	20.93	23
NUF2	83540	Leukemia, MESH:D015473		arsenite|Ca	20.93	158
PSMB9	5698	Leukemia, MESH:D015473		arsenite|Ca	20.93	159
RUNX1T1	862	Leukemia, MESH:D01	marker/me	Calcitriol|D	20.92	16
ACSF2	80221	Leukemia, MESH:D015470		Decitabine	20.92	20
F8	2157	Leukemia, MESH:D015470		Arsenic Tr	20.92	21
GALK1	2584	Leukemia, MESH:D015470		Arsenic Tr	20.92	20
GLA	2717	Leukemia, MESH:D015470		Arsenic Tr	20.92	21

MAP1LC3A	84557	Leukemia, MESH:D007948	2-(2-amin	20.92	3
PHEX	5251	Leukemia, MESH:D015470	Calcitriol|D	20.92	10
RAI14	26064	Leukemia, MESH:D015470	Calcitriol|D	20.92	16
TACC2	10579	Leukemia, MESH:D015470	Dexameth	20.92	17
APOA1	335	Leukemia, MESH:D015470	Alitretinoi	20.91	21
ESR2	2100	Leukemia, MESH:D015473	alpha-Toc	20.91	268
MIR497	574456	Leukemia, MESH:D015473	Arsenic|M	20.9	12
TRIM68	55128	Leukemia, MESH:D015470	Decitabine	20.9	18
ATP1A2	477	Leukemia, MESH:D015473	Calcitriol|D	20.89	158
BMX	660	Leukemia, MESH:D015470	Dexameth	20.89	13
CRKL	1399	Leukemia, MESH:D015473	alvocidib|A	20.89	259
CTSL	1514	Leukemia, MESH:D015473	Arsenic Tr	20.89	262
CXCL2	2920	Leukemia, MESH:D007948	2-(2-amin	20.89	3
MSL2	55167	Leukemia, MESH:D015470	Arsenic Tr	20.89	34
NPIPA1	9284	Leukemia, MESH:D015470	Dexameth	20.89	14
PIGB	9488	Leukemia, MESH:D015470	Doxorubic	20.89	16
POLDIP3	84271	Leukemia, MESH:D015470	Dexameth	20.89	11
SOD3	6649	Leukemia, MESH:D015470	Arsenic Tr	20.89	26
COTL1	23406	Leukemia, MESH:D015473	Arsenic Tr	20.88	263
DCAF12	25853	Leukemia, MESH:D015470	Dexameth	20.88	13
H3-3A	3020	Leukemia, MESH:D015470	Dexameth	20.88	12
JUP	3728	Leukemia, MESH:D015473	Arsenic|Ca	20.88	159
KIFC1	3833	Leukemia, MESH:D015473	Arsenic|ars	20.88	157
LAMB1	3912	Leukemia, MESH:D015473	Arsenic|Da	20.88	158
MIAT	440823	Leukemia, MESH:D015470	Calcitriol|T	20.88	9
NCOA3	8202	Leukemia, MESH:D015473	arsenite|Ca	20.88	156
NPPA	4878	Leukemia, MESH:D015473	2-(2-chlor	20.88	164
PGF	5228	Leukemia, MESH:D015473	Arsenic|Ar	20.88	264
PPP2CA	5515	Leukemia, MESH:D015473	Arsenic|Ar	20.88	266
SDC2	6383	Leukemia, MESH:D015473	Arsenic Tr	20.88	263
TMEM251	26175	Leukemia, MESH:D015470	Doxorubic	20.88	13
CYP4B1	1580	Leukemia, MESH:D015470	Bezafibrat	20.87	13
DHX15	1665	Leukemia, MESH:D015473	Arsenic Tr	20.87	263
GINS3	64785	Leukemia, MESH:D015473	Arsenic|ars	20.87	160
HTATIP2	10553	Leukemia, MESH:D015470	Arsenic Tr	20.87	24
PARP2	10038	Leukemia, MESH:D015473	Arsenic Tr	20.87	157
RERG	85004	Leukemia, MESH:D015473	Arsenic Tr	20.87	262
AHCTF1	25909	Leukemia, MESH:D015473	Arsenic|ars	20.86	159
EVA1A	84141	Leukemia, MESH:D015473	Arsenic|ars	20.86	158
GRIN1	2902	Leukemia, MESH:D015473	arsenic dis	20.86	264
NDUFS3	4722	Leukemia, MESH:D015473	Arsenic|De	20.86	158
SPP1	6696	Leukemia, MESH:D007948	2-(2-amin	20.86	3
TSPAN12	23554	Leukemia, MESH:D015473	Arsenic|ars	20.86	13
CDKN1A	1026	Leukemia, MESH:D007948	2-(2-amin	20.85	4
ABHD5	51099	Leukemia, MESH:D015470	Arsenic Tr	20.84	28
CORIN	10699	Leukemia, MESH:D015470	Arsenic Tr	20.84	54
CSF2RB	1439	Leukemia, MESH:D015470	Arsenic Tr	20.84	40
EPAS1	2034	Leukemia, MESH:D015470	Dasatinib|I	20.84	20
GCH1	2643	Leukemia, MESH:D015470	Decitabine	20.84	31
JAK3	3718	Leukemia, MESH:D015470	Arsenic Tr	20.84	25
LAMA4	3910	Leukemia, MESH:D015470	Busulfan|D	20.84	18
MAP2K4	6416	Leukemia, MESH:D015473	alvocidib|a	20.84	157
MEST	4232	Leukemia, MESH:D015470	Dexameth	20.84	27
PDE7B	27115	Leukemia, MESH:D015470	Arsenic Tr	20.84	38
POLR2A	5430	Leukemia, MESH:D015470	alvocidib|A	20.84	18
PTPN3	5774	Leukemia, MESH:D015470	Calcitriol|D	20.84	18
SREBF2	6721	Leukemia, MESH:D015470	Allopurinc	20.84	20
TRPV2	51393	Leukemia, MESH:D015470	Cytarabine	20.84	82

TTPA	7274	Leukemia, MESH:D015470		Dexameth	20.84	15
WT1	7490	Leukemia, MESH:D01	marker/m	Arsenic Tr	20.83	263
CBLL1	79872	Leukemia, MESH:D015470		Calcitriol|D	20.83	15
CCDC136	64753	Leukemia, MESH:D015470		Decitabine	20.83	15
CEACAM6	4680	Leukemia, MESH:D015470		Calcitriol|D	20.83	19
CENPL	91687	Leukemia, MESH:D015470		Calcitriol|D	20.83	11
CPVL	54504	Leukemia, MESH:D015470		Doxorubic	20.83	22
CRTC2	200186	Leukemia, MESH:D015470		Dexameth	20.83	17
ENPP1	5167	Leukemia, MESH:D015473		Dasatinib|I	20.83	155
FKBP8	23770	Leukemia, MESH:D015470		Doxorubic	20.83	16
FNDC3A	22862	Leukemia, MESH:D015470		Decitabine	20.83	29
FREM1	158326	Leukemia, MESH:D015470		Calcitriol|D	20.83	14
GNL2	29889	Leukemia, MESH:D015470		Air Polluta	20.83	11
HOXA2	3199	Leukemia, MESH:D015470		Arsenic Tr	20.83	82
LAPTM4B	55353	Leukemia, MESH:D015470		Decitabine	20.83	21
LCOR	84458	Leukemia, MESH:D015470		(+)-JQ1 cc	20.83	14
LTV1	84946	Leukemia, MESH:D015470		Dexameth	20.83	13
NTM	50863	Leukemia, MESH:D015470		Cytarabine	20.83	77
OSBPL5	114879	Leukemia, MESH:D015470		Doxorubic	20.83	17
PAPSS2	9060	Leukemia, MESH:D015473		Arsenic Tr	20.83	262
RAB13	5872	Leukemia, MESH:D015470		Arsenic Tr	20.83	21
RAB8A	4218	Leukemia, MESH:D015470		Doxorubic	20.83	15
RP2	6102	Leukemia, MESH:D015470		Arsenic Tr	20.83	16
SENP1	29843	Leukemia, MESH:D015470		Arsenic Tr	20.83	22
SPAG5	10615	Leukemia, MESH:D015473		arsenite|Ca	20.83	157
TNFRSF21	27242	Leukemia, MESH:D015473		Arsenic Tr	20.83	262
XIST	7503	Leukemia, MESH:D015470		Air Polluta	20.83	14
XPO7	23039	Leukemia, MESH:D015470		Calcitriol|I	20.83	9
ZFPM1	161882	Leukemia, MESH:D015470		Bortezomi	20.83	15
ACLY	47	Leukemia, MESH:D015473		Arsenic|ars	20.82	15
PLPP5	84513	Leukemia, MESH:D015470		Dexameth	20.82	14
TTC9	23508	Leukemia, MESH:D015470		Calcitriol|D	20.82	15
EIF5	1983	Leukemia, MESH:D015470		Arsenic Tr	20.81	22
TBX3	6926	Leukemia, MESH:D015470		Decitabine	20.81	19
ALAD	210	Leukemia, MESH:D015473		alpha-Toc	20.79	163
CYP4F2	8529	Leukemia, MESH:D015473		alpha-Toc	20.79	156
KCNMB4	27345	Leukemia, MESH:D015473		Arsenic|Ca	20.79	156
NEFL	4747	Leukemia, MESH:D015470		Alitretinoi	20.79	18
PILRA	29992	Leukemia, MESH:D015473		Arsenic Tr	20.79	261
AFF1	4299	Leukemia, MESH:D015473		Arsenic|ars	20.78	158
CHAT	1103	Leukemia, MESH:D015473		alpha-Toc	20.78	161
CLEC2D	29121	Leukemia, MESH:D015473		Arsenic Tr	20.78	262
CSGALNA	55790	Leukemia, MESH:D015473		Arsenic Tr	20.78	261
DST	667	Leukemia, MESH:D015473		Antimony	20.78	264
MXRA7	439921	Leukemia, MESH:D015473		Arsenic|Ar	20.78	264
SPG7	6687	Leukemia, MESH:D015473		Arsenic|De	20.78	156
CLU	1191	Leukemia, MESH:D015473		Arsenic Tr	20.77	264
EHMT2	10919	Leukemia, MESH:D01	marker/m	Arsenic Tr	20.76	28
ATF3	467	Leukemia, MESH:D007948		2-(2-amin	20.76	4
CYP2S1	29785	Leukemia, MESH:D015470		Calcitriol|D	20.76	19
GULP1	51454	Leukemia, MESH:D015470		Arsenic Tr	20.76	20
RAB27B	5874	Leukemia, MESH:D015470		Arsenic Tr	20.76	23
SYT1	6857	Leukemia, MESH:D015470		Air Polluta	20.76	12
TENT5A	55603	Leukemia, MESH:D015470		Cytarabine	20.76	84
UBE2D3	7323	Leukemia, MESH:D015470		Dexameth	20.76	15
UTRN	7402	Leukemia, MESH:D015470		(+)-JQ1 cc	20.76	22
CASP4	837	Leukemia, MESH:D015473		Arsenic|Ar	20.75	160
CNR1	1268	Leukemia, MESH:D015470		Cytarabine	20.74	95

CYP7A1	1581	Leukemia, MESH:D015473	Arsenic|Ca	20.74	159
DECR1	1666	Leukemia, MESH:D015470	Air Polluta	20.74	23
HRC	3270	Leukemia, MESH:D015470	Daunorub	20.72	51
PDE4D	5144	Leukemia, MESH:D015473	Arsenic|Ar	20.72	266
SLC7A14	57709	Leukemia, MESH:D015473	Arsenic|Ar	20.72	261
ARHGAP8	23779	Leukemia, MESH:D015470	Dronabinc	20.71	9
ATP2A2	488	Leukemia, MESH:D015473	Arsenic Tr	20.71	261
CYTH1	9267	Leukemia, MESH:D015470	Azacitidin	20.71	96
DDX23	9416	Leukemia, MESH:D015470	Air Polluta	20.71	15
FBXO22	26263	Leukemia, MESH:D015470	Decitabine	20.71	23
MFNG	4242	Leukemia, MESH:D015470	Bortezomi	20.71	16
MYCT1	80177	Leukemia, MESH:D015470	Doxorubic	20.71	16
MYL12B	103910	Leukemia, MESH:D015470	Doxorubic	20.71	17
NDUFA12	55967	Leukemia, MESH:D015470	Arsenic Tr	20.71	20
NDUFS2	4720	Leukemia, MESH:D015470	Arsenic Tr	20.71	20
OGFR	11054	Leukemia, MESH:D015470	Bortezomi	20.71	18
PAPLN	89932	Leukemia, MESH:D015470	Air Polluta	20.71	15
PDCD2	5134	Leukemia, MESH:D015470	Arsenic Tr	20.71	17
PDS5A	23244	Leukemia, MESH:D015470	Doxorubic	20.71	15
PHF1	5252	Leukemia, MESH:D015470	Arsenic Tr	20.71	15
RAB5B	5869	Leukemia, MESH:D015470	Cytarabine	20.71	78
TCAF1	9747	Leukemia, MESH:D015470	Doxorubic	20.71	17
TFF1	7031	Leukemia, MESH:D015473	Arsenic|Ar	20.71	162
TMEM204	79652	Leukemia, MESH:D015470	Dexameth	20.71	18
UBAP1	51271	Leukemia, MESH:D015470	Dexameth	20.71	9
XPO6	23214	Leukemia, MESH:D015470	Air Polluta	20.71	10
ZSCAN32	54925	Leukemia, MESH:D015470	Benzene|D	20.71	20
DNASE2	1777	Leukemia, MESH:D015470	Decitabine	20.7	22
GEMIN5	25929	Leukemia, MESH:D015470	Air Polluta	20.7	14
GXYLT2	727936	Leukemia, MESH:D015470	Dexameth	20.7	14
ICOSLG	23308	Leukemia, MESH:D015470	Arsenic Tr	20.7	16
LHX6	26468	Leukemia, MESH:D015470	Arsenic Tr	20.7	21
LINC0112	643837	Leukemia, MESH:D015470	Doxorubic	20.7	13
NDUFB4	4710	Leukemia, MESH:D015470	Dexameth	20.7	18
NDUFS5	4725	Leukemia, MESH:D015473	arsenite|M	20.7	158
OSER1-DT	1.01E+08	Leukemia, MESH:D015470	Doxorubic	20.7	12
PRTFDC1	56952	Leukemia, MESH:D015470	Decitabine	20.7	21
REV3L	5980	Leukemia, MESH:D015473	arsenite|D	20.7	159
SMG1	23049	Leukemia, MESH:D015470	Dexameth	20.7	13
SRGAP1	57522	Leukemia, MESH:D015470	Calcitriol|D	20.7	14
TMEM229	161145	Leukemia, MESH:D015470	Air Polluta	20.7	14
ANOS1	3730	Leukemia, MESH:D015470	Calcitriol|D	20.69	14
CHCHD3	54927	Leukemia, MESH:D015473	Arsenic|ar	20.69	160
KLHDC7B	113730	Leukemia, MESH:D015470	Bortezomi	20.69	10
LYRM4	57128	Leukemia, MESH:D015470	(+)-JQ1 c	20.69	10
RPS25	6230	Leukemia, MESH:D015473	Arsenic Tr	20.69	262
UBAP2L	9898	Leukemia, MESH:D015473	Arsenic Tr	20.69	262
ZBTB25	7597	Leukemia, MESH:D015470	Air Polluta	20.69	12
BORCS5	118426	Leukemia, MESH:D015470	Dexameth	20.68	12
FGF7	2252	Leukemia, MESH:D015473	Arsenic|Ar	20.68	159
IL2RB	3560	Leukemia, MESH:D015470	Benzene|B	20.68	25
RPSA	3921	Leukemia, MESH:D015470	Arsenic Tr	20.68	25
CGA	1081	Leukemia, MESH:D007948	Arsenic Tr	20.67	3
CHPT1	56994	Leukemia, MESH:D015470	Dexameth	20.67	11
CRIM1	51232	Leukemia, MESH:D015470	Arsenic Tr	20.67	18
CYP2J2	1573	Leukemia, MESH:D015470	Arsenic Tr	20.67	22
H6PD	9563	Leukemia, MESH:D015470	Arsenic Tr	20.67	17
HSPG2	3339	Leukemia, MESH:D015470	Calcitriol|I	20.67	12

JMJD1C	221037	Leukemia, MESH:D015470	Air Polluta	20.67	18
NCAPG2	54892	Leukemia, MESH:D015470	Calcitriol|D	20.67	14
PDGFC	56034	Leukemia, MESH:D015470	Dexameth	20.67	23
ST8SIA4	7903	Leukemia, MESH:D015470	Air Polluta	20.67	21
TACC3	10460	Leukemia, MESH:D015473	Arsenic|Ca	20.67	13
TNFRSF19	55504	Leukemia, MESH:D015473	Arsenic|Ca	20.67	160
TRAF3	7187	Leukemia, MESH:D015470	Arsenic Tr	20.67	31
ESR2	2100	Leukemia, MESH:D015470	Alitretinoi	20.66	42
BDNF	627	Leukemia, MESH:D007948	2-(2-amin	20.65	3
TSC2	7249	Leukemia, MESH:D007948	2-(2-amin	20.64	3
EXO1	9156	Leukemia, MESH:D015473	Calcitriol|D	20.63	157
TBC1D8	11138	Leukemia, MESH:D015473	arsenite|Ca	20.63	12
CCK	885	Leukemia, MESH:D015470	Decitabine	20.62	20
CCNA1	8900	Leukemia, MESH:D015473	Arsenic|Ar	20.62	265
CENPH	64946	Leukemia, MESH:D015473	arsenite|Ca	20.62	157
COL11A2	1302	Leukemia, MESH:D015473	Arsenic|De	20.62	160
HMGB1	3146	Leukemia, MESH:D004915	Cytarabine	20.62	3
HOMER2	9455	Leukemia, MESH:D015473	Arsenic Tr	20.62	157
IFIT2	3433	Leukemia, MESH:D015473	Arsenic Tr	20.62	264
NDUFA13	51079	Leukemia, MESH:D015473	arsenite|Bu	20.62	157
NDUFS1	4719	Leukemia, MESH:D015473	Arsenic Tr	20.62	157
NPR1	4881	Leukemia, MESH:D015473	Dexameth	20.62	153
PHLDA2	7262	Leukemia, MESH:D015473	Arsenic Tr	20.62	261
PLA2G4C	8605	Leukemia, MESH:D015473	Arsenic|ars	20.62	12
PRKCZ	5590	Leukemia, MESH:D015470	Arsenic Tr	20.62	102
SRGAP3	9901	Leukemia, MESH:D015473	Arsenic|ars	20.62	23
ARMCX3	51566	Leukemia, MESH:D015473	Arsenic Tr	20.61	262
BGLAP	632	Leukemia, MESH:D015473	Arsenic Tr	20.61	262
CYB561	1534	Leukemia, MESH:D015473	Arsenic|De	20.61	156
DLL4	54567	Leukemia, MESH:D015473	Arsenic|Ar	20.61	161
PCDH9	5101	Leukemia, MESH:D015473	Arsenic|ars	20.61	158
POSTN	10631	Leukemia, MESH:D007948	2-(2-amin	20.61	3
BOC	91653	Leukemia, MESH:D015470	Arsenic Tr	20.59	19
CDK7	1022	Leukemia, MESH:D015470	Decitabine	20.59	15
CHST2	9435	Leukemia, MESH:D015470	Bortezomi	20.59	14
COCH	1690	Leukemia, MESH:D015470	Cyclophos	20.59	19
DIS3	22894	Leukemia, MESH:D015470	Dexameth	20.59	17
DYNC1I2	1781	Leukemia, MESH:D015470	Air Polluta	20.59	20
DYNC2H1	79659	Leukemia, MESH:D015470	Doxorubic	20.59	14
E2F4	1874	Leukemia, MESH:D015470	Arsenic Tr	20.59	26
EFS	10278	Leukemia, MESH:D015470	Dexameth	20.59	17
FN1	2335	Leukemia, MESH:D007948	2-(2-amin	20.59	3
IFT27	11020	Leukemia, MESH:D015470	Dexameth	20.59	14
MAP4K2	5871	Leukemia, MESH:D015470	Arsenic Tr	20.59	25
MMACHC	25974	Leukemia, MESH:D015470	Doxorubic	20.59	15
MRPL3	11222	Leukemia, MESH:D015470	Doxorubic	20.59	15
NPTX1	4884	Leukemia, MESH:D015470	Bortezomi	20.59	23
NXT2	55916	Leukemia, MESH:D015470	Azacitidine	20.59	16
P2RX4	5025	Leukemia, MESH:D015470	Benzene|B	20.59	26
PARP1	142	Leukemia, MESH:D007948	2-(2-amin	20.59	4
PCSK6	5046	Leukemia, MESH:D015470	Calcitriol|D	20.59	23
PDPN	10630	Leukemia, MESH:D015470	Calcitriol|C	20.59	77
PTPA	5524	Leukemia, MESH:D015470	Azacitidine	20.59	15
RLIM	51132	Leukemia, MESH:D015470	Indometha	20.59	9
SIPA1	6494	Leukemia, MESH:D015470	Benzene|D	20.59	27
SMAGP	57228	Leukemia, MESH:D015470	Calcitriol|D	20.59	15
SNRNP20(	23020	Leukemia, MESH:D015470	Dronabinc	20.59	15
SORBS2	8470	Leukemia, MESH:D015470	Cytarabine	20.59	84

SPTSSA	171546	Leukemia, MESH:D015470		Doxorubic	20.59	22
SUMO1	7341	Leukemia, MESH:D015470		Arsenic Tr	20.59	21
THSD4	79875	Leukemia, MESH:D015470		Calcitriol|D	20.59	15
TRPV6	55503	Leukemia, MESH:D015470		Calcitriol|D	20.59	12
TSEN15	116461	Leukemia, MESH:D015470		Bortezomi	20.59	19
WWTR1	25937	Leukemia, MESH:D015470		Arsenic Tr	20.59	17
ZDHHC21	340481	Leukemia, MESH:D015470		Dexameth	20.59	14
ZRANB3	84083	Leukemia, MESH:D015470		Dexameth	20.59	9
CKM	1158	Leukemia, MESH:D015473		Arsenic Tr	20.58	167
PRRC2C	23215	Leukemia, MESH:D004915		Cytarabine	20.58	3
AGTR1	185	Leukemia, MESH:D015473		Arsenic Tr	20.57	263
ATF5	22809	Leukemia, MESH:D015473		arsenite|Ca	20.57	156
CALD1	800	Leukemia, MESH:D015473		Arsenic|Ar	20.57	163
GPT	2875	Leukemia, MESH:D015473		alpha-Toc	20.57	163
HK2	3099	Leukemia, MESH:D015473		Arsenic|Ar	20.57	266
NDRG2	57447	Leukemia, MESH:D015473		Antimony	20.57	158
PPL	5493	Leukemia, MESH:D015473		arsenite|Ca	20.57	158
RB1	5925	Leukemia, MESH:D007948		2-(2-amin	20.57	3
TGIF1	7050	Leukemia, MESH:D015473		Arsenic Tr	20.57	267
ULK1	8408	Leukemia, MESH:D015473		Arsenic|Ar	20.57	266
APOC3	345	Leukemia, MESH:D015470		Allopurinc	20.56	41
NSDHL	50814	Leukemia, MESH:D015470		Arsenic Tr	20.56	21
UGT1A8	54576	Leukemia, MESH:D015470		Etoposide	20.56	22
APLP1	333	Leukemia, MESH:D015473		Arsenic|Et	20.54	155
DDX10	1662	Leukemia, MESH:D015473		Arsenic|M	20.54	158
ERMP1	79956	Leukemia, MESH:D015473		Arsenic|ars	20.54	158
RTN1	6252	Leukemia, MESH:D015473		Arsenic|ars	20.54	159
PAMR1	25891	Leukemia, MESH:D015473		Arsenic|Ca	20.53	159
SH3D19	152503	Leukemia, MESH:D015473		Arsenic Tr	20.53	262
SNRPB	6628	Leukemia, MESH:D015473		Arsenic Tr	20.53	263
WDFY3	23001	Leukemia, MESH:D015473		Arsenic|ars	20.53	157
ZNF682	91120	Leukemia, MESH:D015473		Arsenic Tr	20.53	262
CD44	960	Leukemia, MESH:D004915		Cytarabine	20.52	4
PTPRF	5792	Leukemia, MESH:D015473		arsenite|Ca	20.52	162
SERPINF1	5176	Leukemia, MESH:D015473		Arsenic|ca	20.52	159
NECTIN2	5819	Leukemia, MESH:D01	therapeuti	Doxorubic	20.51	26
ALDH4A1	8659	Leukemia, MESH:D015470		Arsenic Tr	20.51	21
MARCKSL	65108	Leukemia, MESH:D015470		Dexameth	20.51	16
MPZL1	9019	Leukemia, MESH:D004915		Cytarabine	20.51	3
ZBTB33	10009	Leukemia, MESH:D015470		Arsenic Tr	20.51	19
BBLN	79095	Leukemia, MESH:D015470		Bortezomi	20.5	13
C4ORF19	55286	Leukemia, MESH:D015470		Etoposide	20.5	16
CANX	821	Leukemia, MESH:D015470		Arsenic Tr	20.5	24
GCG	2641	Leukemia, MESH:D015470		Dexameth	20.5	12
GTF3C3	9330	Leukemia, MESH:D015470		Indometha	20.5	13
IFT43	112752	Leukemia, MESH:D015470		Dexameth	20.5	16
IGF2	3481	Leukemia, MESH:D015473		Arsenic Tr	20.5	263
MAF	4094	Leukemia, MESH:D015470		Decitabine	20.5	23
NR4A2	4929	Leukemia, MESH:D015473		Arsenic|Ar	20.5	265
PAX9	5083	Leukemia, MESH:D015470		Arsenic Tr	20.5	13
R3HDM4	91300	Leukemia, MESH:D015470		Bortezomi	20.5	13
RIPK1	8737	Leukemia, MESH:D015470		Arsenic Tr	20.5	32
SP140L	93349	Leukemia, MESH:D015470		Calcitriol|T	20.5	11
THRSP	7069	Leukemia, MESH:D015470		Benzene|B	20.5	35
TMEM234	56063	Leukemia, MESH:D015470		Air Polluta	20.5	13
UGT1A10	54575	Leukemia, MESH:D015470		Indometha	20.5	15
ZDHHC6	64429	Leukemia, MESH:D015470		Arsenic Tr	20.5	19
FAM89B	23625	Leukemia, MESH:D015470		(+)-JQ1 cc	20.49	10

RUBCN	9711	Leukemia, MESH:D015470	Dexameth	20.49	12
UBE3D	90025	Leukemia, MESH:D015470	Calcitriol|D	20.49	8
APEX2	27301	Leukemia, MESH:D015470	Doxorubic	20.47	17
ARPC5L	81873	Leukemia, MESH:D015470	Arsenic Tr	20.47	16
CDC5L	988	Leukemia, MESH:D015470	Doxorubic	20.47	15
CDX2	1045	Leukemia, MESH:D015470	Arsenic Tr	20.47	21
CYSTM1	84418	Leukemia, MESH:D015470	Calcitriol|D	20.47	16
DEF6	50619	Leukemia, MESH:D015470	Bortezomi	20.47	13
GALNT11	63917	Leukemia, MESH:D015470	Dexameth	20.47	13
IRF2BPL	64207	Leukemia, MESH:D015470	Doxorubic	20.47	15
ITGA9	3680	Leukemia, MESH:D015470	Arsenic Tr	20.47	21
LINGO1	84894	Leukemia, MESH:D015470	Calcitriol|D	20.47	14
MLXIP	22877	Leukemia, MESH:D015470	Dexameth	20.47	16
MRPL45	84311	Leukemia, MESH:D015470	Air Polluta	20.47	9
MTPN	136319	Leukemia, MESH:D015470	Benzene|D	20.47	29
NOD1	10392	Leukemia, MESH:D015470	Decitabine	20.47	23
PLSCR4	57088	Leukemia, MESH:D015470	Dasatinib|	20.47	10
PLXNA3	55558	Leukemia, MESH:D015470	Calcitriol|D	20.47	13
RAB17	64284	Leukemia, MESH:D015470	Arsenic Tr	20.47	42
SFRP1	6422	Leukemia, MESH:D015473	Arsenic|Ar	20.47	265
SNRNP25	79622	Leukemia, MESH:D015470	Doxorubic	20.47	14
SPATA20	64847	Leukemia, MESH:D015470	Arsenic Tr	20.47	28
ST8SIA2	8128	Leukemia, MESH:D015470	Azacitidine	20.47	16
TES	26136	Leukemia, MESH:D015473	Arsenic Tr	20.47	263
TGIF2	60436	Leukemia, MESH:D015470	Etoposide	20.47	21
TIMM44	10469	Leukemia, MESH:D015470	Dexameth	20.47	15
UGT8	7368	Leukemia, MESH:D015470	Dexameth	20.47	9
SNRPA	6626	Leukemia, MESH:D015473	Antimony	20.46	261
STIL	6491	Leukemia, MESH:D015473	arsenite|Ca	20.46	157
BNC2	54796	Leukemia, MESH:D015473	Arsenic Tr	20.45	264
CCL24	6369	Leukemia, MESH:D015473	Arsenic|Ar	20.45	263
CCL7	6354	Leukemia, MESH:D015470	Arsenic Tr	20.45	24
CDK1	983	Leukemia, MESH:D007948	2-(2-amin	20.45	3
CHTF18	63922	Leukemia, MESH:D015473	Arsenic Tr	20.45	264
LACTB2	51110	Leukemia, MESH:D015473	arsenite|Ca	20.45	158
RGS17	26575	Leukemia, MESH:D015473	Arsenic|Ca	20.45	156
WWC1	23286	Leukemia, MESH:D015473	Arsenic Tr	20.45	260
AKR1A1	10327	Leukemia, MESH:D015470	Arsenic Tr	20.44	48
C1QB	713	Leukemia, MESH:D015470	Arsenic Tr	20.44	26
DNAJC3	5611	Leukemia, MESH:D015470	Benzene|C	20.44	37
SHC1	6464	Leukemia, MESH:D007948	Arsenic Tr	20.44	3
TUBB4B	10383	Leukemia, MESH:D015470	Doxorubic	20.44	20
CEL	1056	Leukemia, MESH:D015470	Calcitriol|C	20.43	19
CLIP1	6249	Leukemia, MESH:D015470	Cyclophos	20.43	16
CMPK2	129607	Leukemia, MESH:D015470	Doxorubic	20.43	15
CYLD	1540	Leukemia, MESH:D015470	Arsenic Tr	20.43	25
DGKA	1606	Leukemia, MESH:D015470	Calcitriol|D	20.43	15
FNDC3B	64778	Leukemia, MESH:D015470	Cyclophos	20.43	16
HMGA1	3159	Leukemia, MESH:D015473	alvocidib|A	20.43	263
KIF18A	81930	Leukemia, MESH:D015470	Calcitriol|D	20.43	16
KIF1A	547	Leukemia, MESH:D004915	Daunorub	20.43	2
KRT15	3866	Leukemia, MESH:D015470	Alitretinoi	20.43	12
SLC23A2	9962	Leukemia, MESH:D015470	Dexameth	20.43	15
CREB1	1385	Leukemia, MESH:D007948	2-(2-amin	20.42	3
GADD45A	1647	Leukemia, MESH:D007948	2-(2-amin	20.42	4
MYB	4602	Leukemia, MESH:D015473	Calcitriol|D	20.42	158
NCOR2	9612	Leukemia, MESH:D015473	Arsenic|ars	20.42	18
XRCC2	7516	Leukemia, MESH:D004915	Doxorubic	20.4	3

ACER2	340485	Leukemia, MESH:D015473	Mercaptoן	20.39	154
ITGB3BP	23421	Leukemia, MESH:D015473	Antimony	20.39	259
TNFRSF10	8795	Leukemia, MESH:D004915	Cytarabine	20.39	2
HPX	3263	Leukemia, MESH:D015470	Doxorubic	20.38	21
PAX1	5075	Leukemia, MESH:D015473	Arsenic|ars	20.38	157
PDLIM3	27295	Leukemia, MESH:D004915	Cytarabine	20.38	3
POLD4	57804	Leukemia, MESH:D015473	Arsenic|Ar	20.38	267
RFC1	5981	Leukemia, MESH:D015473	Arsenic|ca	20.38	155
SDHA	6389	Leukemia, MESH:D015470	Arsenic Tr	20.38	24
TCF21	6943	Leukemia, MESH:D015473	arsenite|C	20.38	156
TMC6	11322	Leukemia, MESH:D015473	Calcitriol|D	20.38	155
USP2	9099	Leukemia, MESH:D015470	Chloroqui	20.38	20
VRK1	7443	Leukemia, MESH:D015473	arsenite|Ca	20.38	157
PTPN11	5781	Leukemia, MESH:D01 marker/m	Arsenic Tr	20.37	265
ARG1	383	Leukemia, MESH:D015470	Arsenic Tr	20.37	27
CIDEA	1149	Leukemia, MESH:D015473	Arsenic|Ar	20.37	266
EPHA2	1969	Leukemia, MESH:D015473	Antimony	20.37	156
HELLS	3070	Leukemia, MESH:D015473	Calcitriol|D	20.37	157
HSPA4L	22824	Leukemia, MESH:D015473	Arsenic Tr	20.37	263
MEF2C	4208	Leukemia, MESH:D015473	Arsenic|ars	20.37	160
RIPOR2	9750	Leukemia, MESH:D015473	Arsenic Tr	20.37	261
SAMD9	54809	Leukemia, MESH:D015473	Arsenic|ars	20.37	158
TMCC3	57458	Leukemia, MESH:D015473	arsenite|Ca	20.37	156
VASP	7408	Leukemia, MESH:D015473	Dexameth	20.37	160
AGTRAP	57085	Leukemia, MESH:D015470	Bortezomi	20.36	14
CETP	1071	Leukemia, MESH:D015470	Alitretinoi	20.36	26
CTSV	1515	Leukemia, MESH:D015470	Calcitriol|D	20.36	17
DYNC2I2	89891	Leukemia, MESH:D015470	Arsenic Tr	20.36	19
H4C9	8294	Leukemia, MESH:D015470	Dexameth	20.36	9
HS6ST2	90161	Leukemia, MESH:D015470	Calcitriol|(	20.36	11
KCNJ16	3773	Leukemia, MESH:D015470	Decitabine	20.36	21
LMO1	4004	Leukemia, MESH:D015470	Benzene|D	20.36	25
LNX2	222484	Leukemia, MESH:D015470	Doxorubic	20.36	14
NRM	11270	Leukemia, MESH:D015470	Calcitriol|D	20.36	12
NUDT21	11051	Leukemia, MESH:D015470	Dexameth	20.36	12
OS9	10956	Leukemia, MESH:D015470	Arsenic Tr	20.36	14
PTRH2	51651	Leukemia, MESH:D015470	Arsenic Tr	20.36	21
REXO2	25996	Leukemia, MESH:D015470	Air Polluta	20.36	14
RIN1	9610	Leukemia, MESH:D015470	Azacitidine	20.36	18
RTN3	10313	Leukemia, MESH:D015470	Decitabine	20.36	20
STX7	8417	Leukemia, MESH:D015470	Air Polluta	20.36	9
SYNPO2	171024	Leukemia, MESH:D015470	Dasatinib|	20.36	14
TIMM13	26517	Leukemia, MESH:D015470	Doxorubic	20.36	15
ZIC1	7545	Leukemia, MESH:D015470	Cyclophos	20.36	15
ANP32E	81611	Leukemia, MESH:D015470	Calcitriol|D	20.35	18
ARID1A	8289	Leukemia, MESH:D015470	Arsenic Tr	20.35	19
CALU	813	Leukemia, MESH:D015470	Dexameth	20.35	19
EPC1	80314	Leukemia, MESH:D015470	Dexameth	20.35	9
FLII	2314	Leukemia, MESH:D015470	Doxorubic	20.35	14
GSTT1A	563972	Leukemia, MESH:D015470	Alitretinoi	20.35	7
IFT57	55081	Leukemia, MESH:D015470	Doxorubic	20.35	14
ITGB8	3696	Leukemia, MESH:D015470	Calcitriol|D	20.35	18
LAMB2	3913	Leukemia, MESH:D015470	Azacitidine	20.35	17
LRRK2	120892	Leukemia, MESH:D015470	Air Polluta	20.35	15
LTBP2	4053	Leukemia, MESH:D015470	Calcitriol|D	20.35	21
PLP1	5354	Leukemia, MESH:D015470	Arsenic Tr	20.35	85
PPP2R1A	5518	Leukemia, MESH:D015470	Air Polluta	20.35	33
PRKAB1	5564	Leukemia, MESH:D015470	Dexameth	20.35	21

RND2	8153	Leukemia, MESH:D015470		Decitabine	20.35	20
ABCB1	5243	Leukemia, MESH:D004915		Cytarabine	20.34	3
H2AX	3014	Leukemia, MESH:D007948		Arsenic Tr	20.34	4
C7ORF31	136895	Leukemia, MESH:D015470		Dronabinc	20.33	7
CD96	10225	Leukemia, MESH:D015470		Arsenic Tr	20.33	15
IDH3A	3419	Leukemia, MESH:D015473		Arsenic|ca	20.33	17
IGFBP5B	403039	Leukemia, MESH:D015470		Dexameth	20.33	7
LINC0033!	29092	Leukemia, MESH:D015470		Dexameth	20.33	11
TMSB4Y	9087	Leukemia, MESH:D015473		arsenite|Re	20.33	158
WDFY3-A	404201	Leukemia, MESH:D015470		Dexameth	20.33	6
ZNF19	7567	Leukemia, MESH:D015470		Arsenic Tr	20.33	18
ZBTB16	7704	Leukemia, MESH:D01	marker/m	Arsenic|De	20.32	162
BCAS4	55653	Leukemia, MESH:D015470		Cytarabine	20.32	79
BNIP3L	665	Leukemia, MESH:D015473		Arsenic Tr	20.32	264
ETS2	2114	Leukemia, MESH:D015473		Arsenic Tr	20.32	263
H3C6	8353	Leukemia, MESH:D015470		Bortezomi	20.32	13
IGHG1	3500	Leukemia, MESH:D015470		Arsenic Tr	20.32	18
INHBB	3625	Leukemia, MESH:D015473		Arsenic|Ar	20.32	265
LRFN4	78999	Leukemia, MESH:D015470		Bortezomi	20.32	14
MFSD4B	91749	Leukemia, MESH:D015470		Arsenic Tr	20.32	15
PPP1R1B	84152	Leukemia, MESH:D015470		Doxorubic	20.32	21
SCYL3	57147	Leukemia, MESH:D015470		Air Polluta	20.32	8
VIM	7431	Leukemia, MESH:D007948		2-(2-amin	20.32	3
ARMT1	79624	Leukemia, MESH:D015470		Air Polluta	20.31	13
B3GNT9	84752	Leukemia, MESH:D015470		Dexameth	20.31	15
CASP7	840	Leukemia, MESH:D007948		2-(2-amin	20.31	3
PNMA8A	55228	Leukemia, MESH:D015470		Decitabine	20.31	18
SCGN	10590	Leukemia, MESH:D015470		Dexameth	20.31	9
SMIM11	54065	Leukemia, MESH:D015470		Bortezomi	20.31	9
YIPF2	78992	Leukemia, MESH:D015470		Doxorubic	20.31	13
ZDHHC11	79844	Leukemia, MESH:D015470		Doxorubic	20.31	21
ZNF706	51123	Leukemia, MESH:D015470		Dexameth	20.31	8
ITGB7	3695	Leukemia, MESH:D015473		Arsenic Tr	20.3	265
SLC17A6	57084	Leukemia, MESH:D015473		arsenite|Cy	20.3	17
TNFRSF14	8764	Leukemia, MESH:D015473		Arsenic|Ar	20.3	265
FGF10	2255	Leukemia, MESH:D015473		Arsenic|Ar	20.29	264
KRT13	3860	Leukemia, MESH:D015473		Arsenic Tr	20.29	262
MAST4	375449	Leukemia, MESH:D015473		Arsenic|ars	20.29	158
NLRP3	114548	Leukemia, MESH:D015470		Air Polluta	20.29	25
ACSL3	2181	Leukemia, MESH:D015473		Calcitriol|D	20.28	13
PRKCZ	5590	Leukemia, MESH:D015473		Arsenic|Ar	20.28	173
CEMIP	57214	Leukemia, MESH:D015470		Calcitriol|C	20.27	81
CENPK	64105	Leukemia, MESH:D015470		Calcitriol|D	20.27	17
GZMA	3001	Leukemia, MESH:D015470		Arsenic Tr	20.27	18
P2RX7	5027	Leukemia, MESH:D015473		Arsenic|Et	20.27	158
PRKAG2	51422	Leukemia, MESH:D015470		Dexameth	20.27	17
SLC51A	200931	Leukemia, MESH:D015470		Calcitriol|D	20.27	12
TNIP1	10318	Leukemia, MESH:D015470		Dexameth	20.27	18
PTK2B	2185	Leukemia, MESH:D015470		Arsenic Tr	20.26	25
TARDBP	23435	Leukemia, MESH:D015470		Chloroqui	20.26	17
ERG	2078	Leukemia, MESH:D01	marker/m	Androgen	20.25	15
ALMS1	7840	Leukemia, MESH:D015470		Calcitriol|D	20.25	12
CEP152	22995	Leukemia, MESH:D015470		Calcitriol|D	20.25	11
CRYAA	1409	Leukemia, MESH:D015470		Azacitidine	20.25	14
GSTA2	2939	Leukemia, MESH:D015470		Alitretinoi	20.25	29
JPH2	57158	Leukemia, MESH:D015470		Arsenic Tr	20.25	20
MIR210	406992	Leukemia, MESH:D015470		Air Polluta	20.25	19
NOS1	4842	Leukemia, MESH:D015470		Bortezomi	20.25	18

S100A7	6278	Leukemia, MESH:D015473	Antimony	20.25	155
SPRR3	6707	Leukemia, MESH:D015473	Antimony	20.25	263
VPS41	27072	Leukemia, MESH:D015470	Air Polluta	20.25	14
BBS2	583	Leukemia, MESH:D015470	Decitabine	20.24	22
BCL2L10	10017	Leukemia, MESH:D015470	Arsenic Tr	20.24	20
CLIP2	7461	Leukemia, MESH:D015470	Dexameth	20.24	17
CMSS1	84319	Leukemia, MESH:D015470	Calcitriol|D	20.24	11
CTDSP2	10106	Leukemia, MESH:D015470	Dexameth	20.24	14
DPAGT1	1798	Leukemia, MESH:D015470	Arsenic Tr	20.24	27
FAM222A	84915	Leukemia, MESH:D015470	Dexameth	20.24	14
GLMP	112770	Leukemia, MESH:D015470	Dexameth	20.24	11
L3MBTL3	84456	Leukemia, MESH:D015470	Calcitriol|D	20.24	8
LARP4B	23185	Leukemia, MESH:D015470	Air Polluta	20.24	14
MBD3	53615	Leukemia, MESH:D015470	Arsenic Tr	20.24	30
MORC3	23515	Leukemia, MESH:D015470	Decitabine	20.24	22
OPCML	4978	Leukemia, MESH:D015470	Arsenic Tr	20.24	20
PEX13	5194	Leukemia, MESH:D015470	Calcitriol|D	20.24	11
PRKD2	25865	Leukemia, MESH:D015470	Doxorubic	20.24	15
SLC22A15	55356	Leukemia, MESH:D015470	Arsenic Tr	20.24	19
STK26	51765	Leukemia, MESH:D015470	Calcitriol|D	20.24	14
TDRD7	23424	Leukemia, MESH:D015470	Arsenic Tr	20.24	20
UQCR11	10975	Leukemia, MESH:D015470	Arsenic Tr	20.24	21
XPNPEP1	7511	Leukemia, MESH:D015470	Arsenic Tr	20.24	15
MGST1	4257	Leukemia, MESH:D015473	Antimony	20.23	265
MIR148A	406940	Leukemia, MESH:D015473	Arsenic|Ar	20.23	159
MT3	4504	Leukemia, MESH:D015473	Arsenic|Ar	20.23	267
TFRC	7037	Leukemia, MESH:D015473	Arsenic|Ar	20.23	264
ADGRE5	976	Leukemia, MESH:D015473	Arsenic Tr	20.22	260
ADORA3	140	Leukemia, MESH:D015473	Calcitriol|D	20.22	158
ARHGAP4	23526	Leukemia, MESH:D015473	Arsenic|De	20.22	159
BAIAP2L1	55971	Leukemia, MESH:D015473	Arsenic|Ar	20.22	266
C5	727	Leukemia, MESH:D015473	Arsenic|Ar	20.22	264
CLBA1	122616	Leukemia, MESH:D015473	Arsenic|De	20.22	156
IGFBP6	3489	Leukemia, MESH:D015473	Arsenic|ars	20.22	158
TIGAR	57103	Leukemia, MESH:D015473	Arsenic Tr	20.22	158
TNFSF15	9966	Leukemia, MESH:D015473	Arsenic|Ar	20.22	264
VAV2	7410	Leukemia, MESH:D015473	Arsenic|Ar	20.22	161
CASP3	836	Leukemia, MESH:D004915	Cytarabine	20.21	4
CBFB	865	Leukemia, MESH:D015473	Arsenic|ars	20.21	159
RPL30	6156	Leukemia, MESH:D015473	Arsenic|Ar	20.21	265
ACOT2	10965	Leukemia, MESH:D015470	Alitretinoi	20.2	16
EFHD1	80303	Leukemia, MESH:D015470	Cytarabine	20.2	87
SP7	121340	Leukemia, MESH:D015470	Arsenic Tr	20.2	20
DDX39A	10212	Leukemia, MESH:D015470	Calcitriol|D	20.19	15
HPCAL1	3241	Leukemia, MESH:D015470	Arsenic Tr	20.19	27
RPS27	6232	Leukemia, MESH:D015470	Arsenic Tr	20.19	16
SYNPO	11346	Leukemia, MESH:D015470	Dexameth	20.19	14
ECT2	1894	Leukemia, MESH:D015473	arsenite|Ca	20.18	157
F2	2147	Leukemia, MESH:D015470	Cytarabine	20.18	90
GJA1	2697	Leukemia, MESH:D015473	Arsenic Tr	20.18	263
PCSK9	255738	Leukemia, MESH:D007948	2-(2-amin	20.18	3
PTHLH	5744	Leukemia, MESH:D015473	Arsenic|ars	20.18	158
TLR3	7098	Leukemia, MESH:D015473	Arsenic Tr	20.18	262
TUBB6	84617	Leukemia, MESH:D015473	Arsenic|Ca	20.18	158
BIRC5	332	Leukemia, MESH:D007948	2-(2-amin	20.17	3
TGM2	7052	Leukemia, MESH:D015473	Arsenic Tr	20.17	267
CDH5	1003	Leukemia, MESH:D007948	2-(2-amin	20.16	3
PPARGC1/	10891	Leukemia, MESH:D015473	alpha-Toc	20.16	266

EPB41L2	2037	Leukemia, MESH:D015473	Arsenic|Ar	20.15	264
GK	2710	Leukemia, MESH:D015470	Benzene|D	20.15	31
GPER1	2852	Leukemia, MESH:D007948	2-(2-amin	20.15	4
KCNAB2	8514	Leukemia, MESH:D015473	Arsenic|Et	20.15	160
PKD2L1	9033	Leukemia, MESH:D015470	Arsenic Tr	20.15	16
PRELP	5549	Leukemia, MESH:D015473	Arsenic Tr	20.15	156
RAD17	5884	Leukemia, MESH:D015473	Arsenic Tr	20.15	261
RASSF2	9770	Leukemia, MESH:D015473	arsenite|C	20.15	156
SVEP1	79987	Leukemia, MESH:D015473	arsenite|C	20.15	156
ABCA3	21	Leukemia, MESH:D015473	Arsenic|Ar	20.14	162
ATP9A	10079	Leukemia, MESH:D015470	Benzene|C	20.14	21
BDKRB2	624	Leukemia, MESH:D015470	Cyclophos	20.14	20
CBX7	23492	Leukemia, MESH:D015473	Arsenic Tr	20.14	261
CEP104	9731	Leukemia, MESH:D015470	Doxorubic	20.14	15
DCX	1641	Leukemia, MESH:D015473	Arsenic|Ar	20.14	262
DTX3L	151636	Leukemia, MESH:D015470	Air Polluta	20.14	14
EML3	256364	Leukemia, MESH:D015470	Air Polluta	20.14	19
FAM76A	199870	Leukemia, MESH:D015470	Bortezomi	20.14	13
FAM83A	84985	Leukemia, MESH:D015470	Decitabine	20.14	14
INPP1	3628	Leukemia, MESH:D015473	Arsenic Tr	20.14	261
INTS7	25896	Leukemia, MESH:D015470	Air Polluta	20.14	16
KCMF1	56888	Leukemia, MESH:D015470	Bortezomi	20.14	13
MAGED1	9500	Leukemia, MESH:D015470	Cyclophos	20.14	20
PLEKHA6	22874	Leukemia, MESH:D015473	Arsenic|Ar	20.14	266
PPP1R14C	81706	Leukemia, MESH:D015470	Calcitriol|D	20.14	19
RAB6A	5870	Leukemia, MESH:D015473	arsenite|D	20.14	161
SEPTIN11	55752	Leukemia, MESH:D015473	Arsenic|Ar	20.14	268
SFTPA1	653509	Leukemia, MESH:D015470	Calcitriol|D	20.14	10
VMO1	284013	Leukemia, MESH:D015470	Arsenic Tr	20.14	29
ZBTB14	7541	Leukemia, MESH:D015470	Indometha	20.14	8
ZNF467	168544	Leukemia, MESH:D015470	Cytarabine	20.14	85
ABHD11	83451	Leukemia, MESH:D015470	Dexameth	20.13	14
ARPC4	10093	Leukemia, MESH:D015470	Doxorubic	20.13	15
BTD	686	Leukemia, MESH:D015470	Dexameth	20.13	13
BTN3A1	11119	Leukemia, MESH:D015470	Dexameth	20.13	13
CFAP44	55779	Leukemia, MESH:D015470	Dronabino	20.13	10
COX18	285521	Leukemia, MESH:D015470	Doxorubic	20.13	13
CREB3L1	90993	Leukemia, MESH:D015470	Calcitriol|D	20.13	21
CXCL3	2921	Leukemia, MESH:D015473	Arsenic|Ca	20.13	14
EML6	400954	Leukemia, MESH:D015470	Dexameth	20.13	10
ENPP5	59084	Leukemia, MESH:D015470	Air Polluta	20.13	9
FBXL14	144699	Leukemia, MESH:D015470	Dexameth	20.13	8
FNIP2	57600	Leukemia, MESH:D015470	Arsenic Tr	20.13	19
GATA2	2624	Leukemia, MESH:D007948	2-(2-amin	20.13	2
HTATSF1	27336	Leukemia, MESH:D015470	Arsenic Tr	20.13	19
ITPRIPL2	162073	Leukemia, MESH:D015470	Dexameth	20.13	9
JPH1	56704	Leukemia, MESH:D015470	Dexameth	20.13	14
LRATD1	151354	Leukemia, MESH:D015470	Calcitriol|D	20.13	13
LRRC17	10234	Leukemia, MESH:D015470	Dexameth	20.13	11
LRRC20	55222	Leukemia, MESH:D015470	Dexameth	20.13	13
MAP1B	4131	Leukemia, MESH:D015473	Arsenic Tr	20.13	267
MAVS	57506	Leukemia, MESH:D015470	Decitabine	20.13	20
N4BP2	55728	Leukemia, MESH:D015470	Dexameth	20.13	15
PARP10	84875	Leukemia, MESH:D015470	Doxorubic	20.13	13
PPM1H	57460	Leukemia, MESH:D015470	Calcitriol|D	20.13	20
RAB11B	9230	Leukemia, MESH:D015470	Alitretinoi	20.13	15
RAB18	22931	Leukemia, MESH:D015470	Dexameth	20.13	14
RPRD1B	58490	Leukemia, MESH:D015470	Dexameth	20.13	11

SHROOM3	57619	Leukemia, MESH:D015470	Calcitriol|D	20.13	14
SLC1A5	6510	Leukemia, MESH:D015473	Arsenic|ca	20.13	14
TMEM135	65084	Leukemia, MESH:D015470	Dexameth	20.13	11
TMEM30B	161291	Leukemia, MESH:D015470	Calcitriol|D	20.13	9
TRIM22	10346	Leukemia, MESH:D015470	Decitabine	20.13	24
TRIM56	81844	Leukemia, MESH:D015470	Calcitriol|D	20.13	14
TTK	7272	Leukemia, MESH:D015473	Calcitriol|D	20.13	155
UNC13B	10497	Leukemia, MESH:D015470	Dexameth	20.13	14
USP13	8975	Leukemia, MESH:D015470	Dexameth	20.13	9
CRIP1	1396	Leukemia, MESH:D015470	Arsenic Tr	20.12	18
PHYH	5264	Leukemia, MESH:D015470	Decitabine	20.12	25
PIK3C3	5289	Leukemia, MESH:D015470	ABT-737|A	20.12	20
RBBP4	5928	Leukemia, MESH:D015470	Arsenic Tr	20.12	23
RPS6KA5	9252	Leukemia, MESH:D015470	Benzene|C	20.12	29
VSNL1	7447	Leukemia, MESH:D015470	Arsenic Tr	20.12	20
TAFA2	338811	Leukemia, MESH:D015473	arsenite|Cl	20.1	7
TAX1BP3	30851	Leukemia, MESH:D004915	Daunorub	20.1	3
CDH1	999	Leukemia, MESH:D007948	2-(2-amin	20.09	3
HAL	3034	Leukemia, MESH:D015473	alpha-Toc	20.09	157
NCOR1	9611	Leukemia, MESH:D015470	Calcitriol|D	20.09	18
SLC1A4	6509	Leukemia, MESH:D015470	Dasatinib|I	20.09	13
SPRR2A	6700	Leukemia, MESH:D015473	Arsenic|Ar	20.09	160
STAT6	6778	Leukemia, MESH:D015473	4'-methox	20.09	156
GLB1	2720	Leukemia, MESH:D015473	Arsenic Tr	20.08	261
HNRNPR	10236	Leukemia, MESH:D015473	Arsenic|Ar	20.08	160
KLF2	10365	Leukemia, MESH:D015473	Arsenic|Ar	20.08	162
THRB	7068	Leukemia, MESH:D015473	Arsenic|Ar	20.08	266
TPX2	22974	Leukemia, MESH:D015473	arsenite|Ca	20.08	158
UGT1A9	54600	Leukemia, MESH:D015470	Arsenic Tr	20.08	23
ANKRD36	57730	Leukemia, MESH:D015473	arsenite|D	20.07	155
GDI2	2665	Leukemia, MESH:D015473	Arsenic|Ar	20.07	263
HSD11B1	3290	Leukemia, MESH:D015473	Calcitriol|D	20.07	9
JCHAIN	3512	Leukemia, MESH:D015473	arsenite|D	20.07	8
KCNK2	3776	Leukemia, MESH:D015473	Arsenic Tr	20.07	155
TENM2	57451	Leukemia, MESH:D015473	Arsenic|Ar	20.07	160
WNT10B	7480	Leukemia, MESH:D015473	Dexameth	20.07	153
CASZ1	54897	Leukemia, MESH:D015473	Arsenic|Ca	20.06	156
CGNL1	84952	Leukemia, MESH:D015473	Arsenic Tr	20.06	263
ELF1	1997	Leukemia, MESH:D015473	Arsenic Tr	20.06	263
PHF19	26147	Leukemia, MESH:D015473	arsenite|Ca	20.06	158
SIX1	6495	Leukemia, MESH:D015473	arsenite|D	20.06	156
TPCN1	53373	Leukemia, MESH:D015473	Arsenic|ars	20.06	156
CD163	9332	Leukemia, MESH:D015470	Calcitriol|D	20.04	12
DLGAP5	9787	Leukemia, MESH:D015473	Calcitriol|D	20.04	155
ELL2	22936	Leukemia, MESH:D015470	Dronabino	20.04	10
IMPA2	3613	Leukemia, MESH:D015470	Arsenic Tr	20.04	19
MYBBP1A	10514	Leukemia, MESH:D015470	Arsenic Tr	20.04	23
NINJ1	4814	Leukemia, MESH:D015470	Air Polluta	20.04	18
OR8B8	26493	Leukemia, MESH:D015470	Azacitidine	20.04	10
PPP1R1B	84152	Leukemia, MESH:D015473	arsenite|G	20.04	157
SLC1A3	6507	Leukemia, MESH:D015473	Calcitriol|C	20.04	156
VEGFB	7423	Leukemia, MESH:D015470	Arsenic Tr	20.04	18
ACO2	50	Leukemia, MESH:D015470	Arsenic Tr	20.03	25
ARPP21	10777	Leukemia, MESH:D015470	Bezafibrat	20.03	12
AXIN2	8313	Leukemia, MESH:D015473	Arsenic Tr	20.03	263
FCGR2A	2212	Leukemia, MESH:D015470	Air Polluta	20.03	81
HAX1	10456	Leukemia, MESH:D015470	Carboplati	20.03	15
IGSF11	152404	Leukemia, MESH:D015470	Decitabine	20.03	26

INS	3630	Leukemia, MESH:D015473	arsenite|Ca	20.03	158
SAT2	112483	Leukemia, MESH:D015470	Arsenic Tr	20.03	21
TAGLN3	29114	Leukemia, MESH:D015470	Daunorub	20.03	41
TOX2	84969	Leukemia, MESH:D015470	Decitabine	20.03	15
TRIAP1	51499	Leukemia, MESH:D015470	Doxorubic	20.03	15
TUBA4A	7277	Leukemia, MESH:D015470	Cyclophos	20.03	28
USP34	9736	Leukemia, MESH:D015470	Air Polluta	20.03	16
ARC	23237	Leukemia, MESH:D007948	2-(2-amin	20.02	3
ARHGEF9	23229	Leukemia, MESH:D015470	Dexameth	20.02	15
ARID4A	5926	Leukemia, MESH:D015470	Dexameth	20.02	15
CTSF	8722	Leukemia, MESH:D015470	Dexameth	20.02	14
DNAJC19	131118	Leukemia, MESH:D015470	Dexameth	20.02	14
DNAJC6	9829	Leukemia, MESH:D015470	Dexameth	20.02	15
DNAJC7	7266	Leukemia, MESH:D015470	Arsenic Tr	20.02	18
FILIP1	27145	Leukemia, MESH:D015470	Arsenic Tr	20.02	25
GAK	2580	Leukemia, MESH:D015470	Arsenic Tr	20.02	19
GALNT12	79695	Leukemia, MESH:D015470	Arsenic Tr	20.02	14
HACD2	201562	Leukemia, MESH:D015470	Calcitriol|D	20.02	15
HLA-C	3107	Leukemia, MESH:D015470	Arsenic Tr	20.02	19
LRRC28	123355	Leukemia, MESH:D015470	Decitabine	20.02	22
LRRC8C	84230	Leukemia, MESH:D015470	Doxorubic	20.02	18
MOCS2	4338	Leukemia, MESH:D015470	Air Polluta	20.02	15
NOP16	51491	Leukemia, MESH:D015470	Dexameth	20.02	11
NSUN2	54888	Leukemia, MESH:D015470	Dexameth	20.02	14
PLXNB1	5364	Leukemia, MESH:D015470	Dexameth	20.02	10
RDH10B	378722	Leukemia, MESH:D015470	Alitretinoi	20.02	7
SDAD1	55153	Leukemia, MESH:D015470	Air Polluta	20.02	9
SERTAD4	56256	Leukemia, MESH:D015470	Decitabine	20.02	15
SETD7	80854	Leukemia, MESH:D015470	Arsenic Tr	20.02	19
SLC25A19	60386	Leukemia, MESH:D015470	Calcitriol|D	20.02	11
TBC1D31	93594	Leukemia, MESH:D015470	Doxorubic	20.02	15
ZDHHC14	79683	Leukemia, MESH:D015470	Benzene|D	20.02	28
CCDC192	728586	Leukemia, MESH:D015470	Dexameth	20.01	9
LINC0059	81698	Leukemia, MESH:D015470	Dexameth	20.01	6
SCAND2P	54581	Leukemia, MESH:D015470	Arsenic Tr	20.01	15
LDLR	3949	Leukemia, MESH:D015473	Arsenic|Ca	20	159
NOTCH1	4851	Leukemia, MESH:D015473	Arsenic|Ar	20	266
PLEK	5341	Leukemia, MESH:D015473	Arsenic|Re	20	162
SNAI3-AS	197187	Leukemia, MESH:D015470	Dexameth	20	9
YWHAQA	399487	Leukemia, MESH:D015470	Dexameth	20	5
ARL17A	51326	Leukemia, MESH:D015470	Dexameth	19.99	11
BACE1	23621	Leukemia, MESH:D015473	alpha-Toc	19.99	264
C6ORF89	221477	Leukemia, MESH:D015470	Dexameth	19.99	11
CASP8AP2	9994	Leukemia, MESH:D015473	Arsenic Tr	19.99	155
CD151	977	Leukemia, MESH:D015473	Arsenic|ca	19.99	155
CENPM	79019	Leukemia, MESH:D015473	Arsenic|ars	19.99	160
CST6	1474	Leukemia, MESH:D015473	Arsenic|Ca	19.99	156
HYMAI	57061	Leukemia, MESH:D015470	Methotrex	19.99	9
RERE	473	Leukemia, MESH:D015473	Arsenic|Ar	19.99	161
RHPN2	85415	Leukemia, MESH:D015473	Arsenic|Ca	19.99	158
RPF2	84154	Leukemia, MESH:D015473	Arsenic Tr	19.99	263
EGR1	1958	Leukemia, MESH:D007948	2-(2-amin	19.98	3
FOXJ2	55810	Leukemia, MESH:D015470	Doxorubic	19.98	22
XBP1	7494	Leukemia, MESH:D015473	alpha-Toc	19.98	268
BIN3	55909	Leukemia, MESH:D015470	Doxorubic	19.97	21
FAM214A	56204	Leukemia, MESH:D015470	Dexameth	19.97	11
FKBP11	51303	Leukemia, MESH:D015470	Arsenic Tr	19.97	18
LEFTY2	7044	Leukemia, MESH:D015470	Doxorubic	19.97	14

MORC2	22880	Leukemia, MESH:D015470	Bortezomi	19.97	15
NAT1	9	Leukemia, MESH:D015470	Calcitriol|H	19.97	14
NSUN7	79730	Leukemia, MESH:D015470	Decitabine	19.97	14
TMSB10	9168	Leukemia, MESH:D015470	Doxorubic	19.97	16
UCP3	7352	Leukemia, MESH:D015470	Bezafibrat	19.97	17
UGDH	7358	Leukemia, MESH:D015470	Alitretinoi	19.97	17
ANTKMT	65990	Leukemia, MESH:D015470	Indometha	19.96	10
CCNB1	891	Leukemia, MESH:D007948	2-(2-amin	19.96	3
CFAP36	112942	Leukemia, MESH:D015470	Arsenic Tr	19.96	19
IGF1	3479	Leukemia, MESH:D007948	2-(2-amin	19.96	3
IL11RA	3590	Leukemia, MESH:D015470	Benzene|D	19.96	28
MIR574	693159	Leukemia, MESH:D015470	Doxorubic	19.96	14
RBMXL1	494115	Leukemia, MESH:D015470	Calcitriol|D	19.96	12
SHISAL1	85352	Leukemia, MESH:D015470	Doxorubic	19.96	12
TGM4	7047	Leukemia, MESH:D015473	caffeic aci	19.96	153
TMEM8B	51754	Leukemia, MESH:D015470	Doxorubic	19.96	12
ZNF436	80818	Leukemia, MESH:D015470	Dexameth	19.96	12
IFNB1	3456	Leukemia, MESH:D015473	alvocidib|A	19.95	264
CSRP1	1465	Leukemia, MESH:D015473	Arsenic Tr	19.94	267
ENO3	2027	Leukemia, MESH:D015473	Arsenic|Cy	19.94	22
CAMK2A	815	Leukemia, MESH:D007948	Arsenic Tr	19.93	3
PIMREG	54478	Leukemia, MESH:D015473	arsenite|Ca	19.93	155
POLQ	10721	Leukemia, MESH:D015473	arsenite|Ca	19.93	10
ARF3	377	Leukemia, MESH:D015470	Dexameth	19.92	13
ARSA	410	Leukemia, MESH:D015470	Calcitriol|D	19.92	17
ASRGL1	80150	Leukemia, MESH:D015473	Arsenic Tr	19.92	156
C4A	720	Leukemia, MESH:D015470	Dexameth	19.92	13
CAMK2A	815	Leukemia, MESH:D015470	Arsenic Tr	19.92	19
CDC37	11140	Leukemia, MESH:D015470	Arsenic Tr	19.92	34
CDC42SE1	56882	Leukemia, MESH:D015470	Dexameth	19.92	12
CKAP2L	150468	Leukemia, MESH:D015473	arsenite|Ca	19.92	157
CUBN	8029	Leukemia, MESH:D015470	Dexameth	19.92	12
EDA2R	60401	Leukemia, MESH:D015470	Arsenic Tr	19.92	20
ERC1	23085	Leukemia, MESH:D015470	Arsenic Tr	19.92	22
G3BP2	9908	Leukemia, MESH:D015470	Air Polluta	19.92	14
GBA	2629	Leukemia, MESH:D015470	Alitretinoi	19.92	21
IKZF2	22807	Leukemia, MESH:D015470	(+)-JQ1 cc	19.92	13
KANK4	163782	Leukemia, MESH:D015473	Arsenic Tr	19.92	265
LAP3	51056	Leukemia, MESH:D015473	Arsenic|Ar	19.92	265
LMOD1	25802	Leukemia, MESH:D015470	Arsenic Tr	19.92	20
MITF	4286	Leukemia, MESH:D015470	Alitretinoi	19.92	33
MUC4	4585	Leukemia, MESH:D015470	Decitabine	19.92	15
N4BP2L2	10443	Leukemia, MESH:D015473	Arsenic|De	19.92	160
PARP8	79668	Leukemia, MESH:D015470	Calcitriol|D	19.92	9
PRKAG1	5571	Leukemia, MESH:D015470	Decitabine	19.92	23
RNASEL	6041	Leukemia, MESH:D015470	Air Polluta	19.92	14
SBSPON	157869	Leukemia, MESH:D015470	Cytarabine	19.92	80
SFRP4	6424	Leukemia, MESH:D015473	Arsenic|ars	19.92	15
SFXN4	119559	Leukemia, MESH:D015470	Doxorubic	19.92	13
SMPD2	6610	Leukemia, MESH:D015470	Bezafibrat	19.92	17
SSBP1	6742	Leukemia, MESH:D015473	Arsenic|Ar	19.92	266
STING1	340061	Leukemia, MESH:D015470	Dexameth	19.92	13
TP53	7157	Leukemia, MESH:D007948	2-(2-amin	19.92	4
TSEN2	80746	Leukemia, MESH:D015470	Indometha	19.92	9
TYRO3	7301	Leukemia, MESH:D015473	Dexameth	19.92	157
USPL1	10208	Leukemia, MESH:D015470	Dexameth	19.92	13
HSPA12A	259217	Leukemia, MESH:D015473	Arsenic|ars	19.91	158
MYO1D	4642	Leukemia, MESH:D015473	Arsenic|Ca	19.91	157

SLC43A1	8501	Leukemia, MESH:D015473		Arsenic|Ar	19.91	264
VANGL1	81839	Leukemia, MESH:D015470		Arsenic Tr	19.91	19
HSPA9	3313	Leukemia, MESH:D015473		Arsenic|Ar	19.9	266
MAFB	9935	Leukemia, MESH:D015473		Arsenic|ars	19.9	158
STC1	6781	Leukemia, MESH:D015473		Arsenic|Ar	19.9	160
CACNA1A	773	Leukemia, MESH:D015470		Arsenic Tr	19.89	17
FBXO30	84085	Leukemia, MESH:D015470		Arsenic Tr	19.89	22
VKORC1	79001	Leukemia, MESH:D015470		Dexameth	19.89	23
FGF2	2247	Leukemia, MESH:D015473		Arsenic|Ar	19.88	266
GSTT1	2952	Leukemia, MESH:D015470		Arsenic Tr	19.88	35
GRN	2896	Leukemia, MESH:D007948		2-(2-amin	19.87	3
MMP12	4321	Leukemia, MESH:D007948		2-(2-amin	19.87	2
CDK9	1025	Leukemia, MESH:D015473		alvocidib|a	19.86	155
GJB1	2705	Leukemia, MESH:D015473		caffeic aci	19.86	152
HSPB1	3315	Leukemia, MESH:D015473		Arsenic Tr	19.86	263
LIPG	9388	Leukemia, MESH:D015473		Arsenic|Ca	19.86	17
TKT	7086	Leukemia, MESH:D015470		Arsenic Tr	19.86	35
ALDH1A2	8854	Leukemia, MESH:D015473		arsenite|ca	19.85	157
BORA	79866	Leukemia, MESH:D015473		Arsenic|Cy	19.85	160
COBLL1	22837	Leukemia, MESH:D015473		Arsenic Tr	19.85	263
FANCG	2189	Leukemia, MESH:D015473		Calcitriol|D	19.85	11
IL10RB	3588	Leukemia, MESH:D015473		Arsenic|De	19.85	160
OAS3	4940	Leukemia, MESH:D015473		Arsenic|ars	19.85	157
SLC1A4	6509	Leukemia, MESH:D015473		arsenite|D	19.85	157
SQSTM1	8878	Leukemia, MESH:D007948		2-(2-amin	19.85	3
TIPIN	54962	Leukemia, MESH:D015473		arsenite|D	19.85	156
VCL	7414	Leukemia, MESH:D015473		Arsenic|De	19.85	158
COX7B	1349	Leukemia, MESH:D015473		Arsenic Tr	19.84	263
EIF4G2	1982	Leukemia, MESH:D015473		Arsenic Tr	19.84	262
GALM	130589	Leukemia, MESH:D015473		Arsenic Tr	19.84	261
SCNN1B	6338	Leukemia, MESH:D015473		Arsenic Tr	19.84	156
EN2	2020	Leukemia, MESH:D015473		Etoposide	19.83	154
ACSM3	6296	Leukemia, MESH:D015470		Dexameth	19.82	16
ARPP19	10776	Leukemia, MESH:D015470		Arsenic Tr	19.82	20
ATP7B	540	Leukemia, MESH:D015473		Buthionine	19.82	156
LTA	4049	Leukemia, MESH:D015470		Arsenic Tr	19.82	21
PSME1	5720	Leukemia, MESH:D015470		Decitabine	19.82	22
TPM2	7169	Leukemia, MESH:D015470		Azacitidine	19.82	24
SPI1	6688	Leukemia, MESH:D01	marker/me	Arsenic Tr	19.81	24
ACLY	47	Leukemia, MESH:D015470		Benzene|D	19.81	34
APP	351	Leukemia, MESH:D007948		2-(2-amin	19.81	3
ART3	419	Leukemia, MESH:D015470		Dexameth	19.81	18
ATAD5	79915	Leukemia, MESH:D015470		Calcitriol|D	19.81	9
CDK19	23097	Leukemia, MESH:D015470		Doxorubic	19.81	17
CIART	148523	Leukemia, MESH:D015470		Doxorubic	19.81	14
CYP20A1	57404	Leukemia, MESH:D015470		Decitabine	19.81	16
FAT3	120114	Leukemia, MESH:D015470		Decitabine	19.81	22
FCHSD2	9873	Leukemia, MESH:D015470		Benzene|C	19.81	28
GLIS3	169792	Leukemia, MESH:D015470		Doxorubic	19.81	14
GNL3L	54552	Leukemia, MESH:D015470		Dexameth	19.81	13
HAS1	3036	Leukemia, MESH:D015470		Benzene|D	19.81	30
HMGN5	79366	Leukemia, MESH:D015470		Decitabine	19.81	18
KCNG1	3755	Leukemia, MESH:D015470		Arsenic Tr	19.81	15
KRCC1	51315	Leukemia, MESH:D015470		Air Polluta	19.81	19
NCK2	8440	Leukemia, MESH:D015470		Daunorub	19.81	45
OBSCN	84033	Leukemia, MESH:D015470		Bortezomi	19.81	14
PCBD1	5092	Leukemia, MESH:D015470		Arsenic Tr	19.81	19
PLA2G2A	5320	Leukemia, MESH:D015473		caffeic aci	19.81	155

PLIN2	123	Leukemia, MESH:D015473	Arsenic|Ar	19.81	264
PREB	10113	Leukemia, MESH:D015470	Dexameth	19.81	14
RRP9	9136	Leukemia, MESH:D015470	Arsenic Tr	19.81	20
RUNX1	861	Leukemia, MESH:D015473	arsenite|Ca	19.81	159
RYR3	6263	Leukemia, MESH:D015470	Arsenic Tr	19.81	19
SMYD2	56950	Leukemia, MESH:D015470	Decitabine	19.81	20
SNX5	27131	Leukemia, MESH:D015470	Dexameth	19.81	14
ST6GALNA	10610	Leukemia, MESH:D015470	Calcitriol|D	19.81	22
STOM	2040	Leukemia, MESH:D004915	Cytarabine	19.81	3
TBC1D5	9779	Leukemia, MESH:D015470	Dexameth	19.81	11
TTYH2	94015	Leukemia, MESH:D015470	Doxorubic	19.81	17
DALRD3	55152	Leukemia, MESH:D015470	Cytarabine	19.8	76
HENMT1	113802	Leukemia, MESH:D015473	Arsenic|Ar	19.8	261
PODN	127435	Leukemia, MESH:D015470	Calcitriol|D	19.8	9
RECQL5	9400	Leukemia, MESH:D015470	Cytarabine	19.8	87
SNHG15	285958	Leukemia, MESH:D015470	Doxorubic	19.8	13
AP5S1	55317	Leukemia, MESH:D015470	Methotrex	19.79	11
DNAAF11	23639	Leukemia, MESH:D015470	Dexameth	19.79	7
DNAJC4	3338	Leukemia, MESH:D015470	Bortezomi	19.79	14
H3C7	8968	Leukemia, MESH:D015470	Dexameth	19.79	17
KIF24	347240	Leukemia, MESH:D015470	Calcitriol|(	19.79	9
POM121	9883	Leukemia, MESH:D015470	Dexameth	19.79	12
PRPF40B	25766	Leukemia, MESH:D015470	Dexameth	19.79	12
RNF185	91445	Leukemia, MESH:D015470	Calcitriol|D	19.79	13
RTL8A	26071	Leukemia, MESH:D015470	Dexameth	19.79	12
SLC27A6	28965	Leukemia, MESH:D015470	Dexameth	19.79	10
SLC35A2	7355	Leukemia, MESH:D015470	Dexameth	19.79	14
TGFBRAP1	9392	Leukemia, MESH:D015470	Dronabino	19.79	8
ZBTB34	403341	Leukemia, MESH:D015470	Decitabine	19.79	21
CAPN2	824	Leukemia, MESH:D007948	2-(2-amin	19.78	2
EIF3A	8661	Leukemia, MESH:D004915	Doxorubic	19.78	3
HJURP	55355	Leukemia, MESH:D015473	Arsenic|Ca	19.78	158
PRRC2C	23215	Leukemia, MESH:D015473	Arsenic Tr	19.78	267
CFL2	1073	Leukemia, MESH:D015473	arsenite|D	19.77	157
CYC1	1537	Leukemia, MESH:D015473	Arsenic|Ar	19.77	266
DUSP16	80824	Leukemia, MESH:D015473	Arsenic|Ar	19.77	266
EEPD1	80820	Leukemia, MESH:D015473	Arsenic|Ca	19.77	157
LIMCH1	22998	Leukemia, MESH:D004915	Cytarabine	19.77	3
RAD54B	25788	Leukemia, MESH:D015473	Arsenic Tr	19.77	261
RHBDF1	64285	Leukemia, MESH:D015473	Arsenic|De	19.77	158
PPP3CA	5530	Leukemia, MESH:D015473	Arsenic Tr	19.76	261
APOB	338	Leukemia, MESH:D015473	alpha-Toc	19.75	20
F5	2153	Leukemia, MESH:D015470	Arsenic Tr	19.75	30
FDXR	2232	Leukemia, MESH:D015470	Daunorub	19.75	46
HEXB	3074	Leukemia, MESH:D015470	Decitabine	19.75	24
HOXA1	3198	Leukemia, MESH:D015470	Azacitidine	19.75	14
HTR2B	3357	Leukemia, MESH:D015470	Bortezomi	19.75	13
SVIL	6840	Leukemia, MESH:D01 marker/me	Decitabine	19.74	23
FERMT2	10979	Leukemia, MESH:D015470	Arsenic Tr	19.74	23
PDLIM7	9260	Leukemia, MESH:D015470	Arsenic Tr	19.74	18
CYP1A2	1544	Leukemia, MESH:D015473	alpha-Toc	19.73	164
ACSL1	2180	Leukemia, MESH:D015473	Antimony	19.72	262
FDPS	2224	Leukemia, MESH:D015470	Androgen	19.72	38
GRIN2B	2904	Leukemia, MESH:D015470	Azacitidine	19.72	29
ADH1B	125	Leukemia, MESH:D015470	Arsenic Tr	19.71	17
BMP5	653	Leukemia, MESH:D015473	Arsenic|ars	19.71	22
CD28	940	Leukemia, MESH:D015473	Calcitriol|D	19.71	8
CD3E	916	Leukemia, MESH:D015470	Benzene|C	19.71	23

CDK5RAP3	80279	Leukemia, MESH:D015470	Dexameth	19.71	10
CPPED1	55313	Leukemia, MESH:D015470	Air Polluta	19.71	11
CREBZF	58487	Leukemia, MESH:D015470	Pentachlo	19.71	15
DKK2	27123	Leukemia, MESH:D015470	Decitabine	19.71	20
DNM3	26052	Leukemia, MESH:D015470	Arsenic Tr	19.71	14
GABPB1	2553	Leukemia, MESH:D015470	Decitabine	19.71	21
GPC4	2239	Leukemia, MESH:D015473	Arsenic|Ar	19.71	265
GPSM2	29899	Leukemia, MESH:D015473	arsenite|Ca	19.71	10
HLA-DMA	3108	Leukemia, MESH:D015470	Air Polluta	19.71	9
MAN1C1	57134	Leukemia, MESH:D015470	Arsenic Tr	19.71	19
MGARP	84709	Leukemia, MESH:D015470	Cytarabine	19.71	77
MIDN	90007	Leukemia, MESH:D015470	Doxorubic	19.71	15
MXRA8	54587	Leukemia, MESH:D015470	Calcitriol|D	19.71	14
NEIL1	79661	Leukemia, MESH:D015470	Decitabine	19.71	21
NLRP12	91662	Leukemia, MESH:D015470	Air Polluta	19.71	16
NRARP	441478	Leukemia, MESH:D015470	Calcitriol|(	19.71	10
SEMA3A	10371	Leukemia, MESH:D015473	Arsenic|Ar	19.71	159
SGPP1	81537	Leukemia, MESH:D015470	Bortezomi	19.71	15
SLC29A2	3177	Leukemia, MESH:D015470	Azacitidine	19.71	24
SLC9A3R2	9351	Leukemia, MESH:D015470	Doxorubic	19.71	17
SNRPE	6635	Leukemia, MESH:D015470	Arsenic Tr	19.71	21
TAF15	8148	Leukemia, MESH:D015470	Arsenic Tr	19.71	19
TMC5	79838	Leukemia, MESH:D015470	Arsenic Tr	19.71	14
TMED9	54732	Leukemia, MESH:D015470	Cytarabine	19.71	80
TRANK1	9881	Leukemia, MESH:D015470	Air Polluta	19.71	14
TSPAN3	10099	Leukemia, MESH:D015470	Arsenic Tr	19.71	21
TTLL7	79739	Leukemia, MESH:D015470	Arsenic Tr	19.71	19
ADGRF5	221395	Leukemia, MESH:D015470	Arsenic Tr	19.7	19
CSNK1D	1453	Leukemia, MESH:D015473	Arsenic|Ar	19.7	268
EFNB1	1947	Leukemia, MESH:D015473	Arsenic|ars	19.7	157
EIF4E	1977	Leukemia, MESH:D007948	2-(2-amin	19.7	3
HADHA	3030	Leukemia, MESH:D015470	Allopurino	19.7	20
MYOD1	4654	Leukemia, MESH:D015473	arsenite|D	19.7	157
PSMD8	5714	Leukemia, MESH:D015473	Arsenic|Ar	19.7	265
PTGER4	5734	Leukemia, MESH:D015470	Calcitriol|C	19.7	19
RAPH1	65059	Leukemia, MESH:D015473	Arsenic Tr	19.7	264
RBMS1	5937	Leukemia, MESH:D015473	Arsenic|Ge	19.7	157
SEC23B	10483	Leukemia, MESH:D015473	Arsenic|ars	19.7	12
DNM3OS	1.01E+08	Leukemia, MESH:D015470	Arsenic Tr	19.69	12
PRR3	80742	Leukemia, MESH:D015473	Arsenic Tr	19.69	260
ALB	213	Leukemia, MESH:D015473	alpha-Toc	19.68	265
BNIP3	664	Leukemia, MESH:D015473	Arsenic Tr	19.68	263
HMMR	3161	Leukemia, MESH:D015473	arsenite|Ca	19.68	157
LINC00173	1E+08	Leukemia, MESH:D015470	Decitabine	19.68	13
MIR16-1	406950	Leukemia, MESH:D015470	Arsenic Tr	19.68	14
OIP5-AS1	729082	Leukemia, MESH:D015470	Dexameth	19.68	13
PLG	5340	Leukemia, MESH:D015473	alpha-Toc	19.68	168
VCAN	1462	Leukemia, MESH:D015473	arsenite|Ca	19.68	157
ZNF638	27332	Leukemia, MESH:D015473	arsenite|D	19.68	7
ACP5	54	Leukemia, MESH:D015473	caffeic aci	19.67	156
AIM2	9447	Leukemia, MESH:D015473	Arsenic|M	19.67	155
BCL10	8915	Leukemia, MESH:D015470	Arsenic Tr	19.67	22
CDK5	1020	Leukemia, MESH:D015470	alvocidib|A	19.67	22
CRIP2	1397	Leukemia, MESH:D015470	Bortezomi	19.67	24
FOXQ1	94234	Leukemia, MESH:D015470	Calcitriol|D	19.67	10
GPATCH1	253635	Leukemia, MESH:D015473	arsenite|D	19.67	155
GPRC5A	9052	Leukemia, MESH:D015470	Arsenic Tr	19.67	26
MED1	5469	Leukemia, MESH:D015470	15-deoxy-	19.67	21

SERPINA3	12	Leukemia, MESH:D015470		Alitretinoi	19.67	11
SLC16A2	6567	Leukemia, MESH:D015470		Dasatinib|I	19.67	16
TNS3	64759	Leukemia, MESH:D015470		Dasatinib|I	19.67	10
ZNF267	10308	Leukemia, MESH:D015473		Arsenic|De	19.67	159
AIM2	9447	Leukemia, MESH:D015470		Air Polluta	19.65	14
KRT18	3875	Leukemia, MESH:D015473		Arsenic|Ar	19.65	264
CS	1431	Leukemia, MESH:D015473		Arsenic|ca	19.64	158
EVL	51466	Leukemia, MESH:D015473		arsenic dis	19.64	261
FOXO4	4303	Leukemia, MESH:D015473		Arsenic Tr	19.64	264
MRM2	29960	Leukemia, MESH:D015470		Dronabinc	19.64	15
MRPS5	64969	Leukemia, MESH:D015470		Bortezomi	19.64	8
PTPRN	5798	Leukemia, MESH:D015473		arsenite|Ca	19.64	159
PTPRZ1	5803	Leukemia, MESH:D015473		arsenite|Ca	19.64	158
SOX21	11166	Leukemia, MESH:D015470		Cytarabine	19.64	81
ULBP2	80328	Leukemia, MESH:D015470		Arsenic Tr	19.64	23
AHSA2P	130872	Leukemia, MESH:D015470		Arsenic Tr	19.63	17
BICRAL	23506	Leukemia, MESH:D015470		Dexameth	19.63	8
C6ORF141	135398	Leukemia, MESH:D015470		Decitabine	19.63	14
CDIN1	84529	Leukemia, MESH:D015470		Doxorubic	19.63	13
CPLANE1	65250	Leukemia, MESH:D015470		Doxorubic	19.63	13
DNAJC27	51277	Leukemia, MESH:D015470		Decitabine	19.63	19
EIF4ENIF1	56478	Leukemia, MESH:D015470		Benzene|D	19.63	22
FOXN2	3344	Leukemia, MESH:D015470		Dexameth	19.63	13
GDF10	2662	Leukemia, MESH:D015473		Arsenic|De	19.63	156
GPN1	11321	Leukemia, MESH:D015470		Arsenic Tr	19.63	18
GRN	2896	Leukemia, MESH:D015473		Arsenic Tr	19.63	265
H2BC21	8349	Leukemia, MESH:D015473		Dexameth	19.63	156
HSD17B7	51478	Leukemia, MESH:D015473		Antimony	19.63	160
ICA1L	130026	Leukemia, MESH:D015470		Doxorubic	19.63	13
KIAA0930	23313	Leukemia, MESH:D015470		Air Polluta	19.63	18
MIR100HC	399959	Leukemia, MESH:D015470		Calcitriol|D	19.63	12
MKS1	54903	Leukemia, MESH:D015470		Calcitriol|D	19.63	8
NOL12	79159	Leukemia, MESH:D015470		Doxorubic	19.63	16
RRM1	6240	Leukemia, MESH:D015473		Arsenic Tr	19.63	263
S100B	6285	Leukemia, MESH:D015473		Arsenic Tr	19.63	260
SACS	26278	Leukemia, MESH:D015473		Arsenic Tr	19.63	265
SH3BGRL	6451	Leukemia, MESH:D015473		Arsenic Tr	19.63	262
SLC35A4	113829	Leukemia, MESH:D015470		Dronabinc	19.63	10
SLC6A6	6533	Leukemia, MESH:D015473		Arsenic|Da	19.63	158
SNX21	90203	Leukemia, MESH:D015470		Doxorubic	19.63	14
TLL2	7093	Leukemia, MESH:D015470		Arsenic Tr	19.63	15
TRAPPC4	51399	Leukemia, MESH:D015470		Arsenic Tr	19.63	19
TSPAN11	441631	Leukemia, MESH:D015470		Dexameth	19.63	15
UBE3A	7337	Leukemia, MESH:D015473		Arsenic Tr	19.63	263
UPP1	7378	Leukemia, MESH:D015473		Arsenic|Ar	19.63	264
ZNF267	10308	Leukemia, MESH:D015470		Air Polluta	19.63	8
ZNF638	27332	Leukemia, MESH:D015470		Dexameth	19.63	11
BAALC	79870	Leukemia, MESH:D01	marker/me	Arsenic Tr	19.62	19
CHEK2	11200	Leukemia, MESH:D015473		Arsenic Tr	19.62	159
PKM	5315	Leukemia, MESH:D015473		Arsenic|Ar	19.62	266
ADIPOR1	51094	Leukemia, MESH:D015470		Alitretinoi	19.61	13
AFAP1L2	84632	Leukemia, MESH:D015470		Cytarabine	19.61	87
ALPP	250	Leukemia, MESH:D015470		Arsenic Tr	19.61	31
CACNA2D	9254	Leukemia, MESH:D015470		Arsenic Tr	19.61	52
CORO6	84940	Leukemia, MESH:D015470		Arsenic Tr	19.61	18
CPEB1	64506	Leukemia, MESH:D015470		Arsenic Tr	19.61	22
DUSP7	1849	Leukemia, MESH:D015470		Air Polluta	19.61	21
FBXL4	26235	Leukemia, MESH:D015470		Arsenic Tr	19.61	17

GALNT7	51809	Leukemia, MESH:D015470	Dexameth	19.61	10
GTF2B	2959	Leukemia, MESH:D015470	Cyclophos	19.61	14
IL20RB	53833	Leukemia, MESH:D015470	Arsenic Tr	19.61	21
NEO1	4756	Leukemia, MESH:D015470	Arsenic Tr	19.61	16
PDLIM4	8572	Leukemia, MESH:D015470	Cytarabine	19.61	87
POLR3K	51728	Leukemia, MESH:D015470	Dronabinc	19.61	10
RAPGEF5	9771	Leukemia, MESH:D015470	Arsenic Tr	19.61	20
SARAF	51669	Leukemia, MESH:D015470	Benzene|C	19.61	95
SELENOS	55829	Leukemia, MESH:D015470	Decitabine	19.61	14
SEPTIN6	23157	Leukemia, MESH:D015470	Arsenic Tr	19.61	23
SNCG	6623	Leukemia, MESH:D015470	Calcitriol|D	19.61	17
SOS2	6655	Leukemia, MESH:D015470	Benzene|C	19.61	28
WDR33	55339	Leukemia, MESH:D015470	Decitabine	19.61	23
ZSWIM6	57688	Leukemia, MESH:D015470	Air Polluta	19.61	14
BRD2	6046	Leukemia, MESH:D015470	Doxorubic	19.6	16
CFI	3426	Leukemia, MESH:D015470	Arsenic Tr	19.6	20
DNAJB5	25822	Leukemia, MESH:D015470	Dexameth	19.6	16
EIF4G3	8672	Leukemia, MESH:D015470	Dexameth	19.6	14
GYG1	2992	Leukemia, MESH:D015470	Dexameth	19.6	14
HDAC6	10013	Leukemia, MESH:D015470	Bortezomi	19.6	18
IDE	3416	Leukemia, MESH:D015470	Bortezomi	19.6	20
NOL8	55035	Leukemia, MESH:D015470	Dexameth	19.6	15
PCDH8	5100	Leukemia, MESH:D015470	Dexameth	19.6	14
PGAP2	27315	Leukemia, MESH:D015470	Doxorubic	19.6	14
PRICKLE2	166336	Leukemia, MESH:D015470	Calcitriol|D	19.6	9
RALB	5899	Leukemia, MESH:D015470	Decitabine	19.6	20
RAPGEF4	11069	Leukemia, MESH:D015470	Arsenic Tr	19.6	34
RELA	5970	Leukemia, MESH:D007948	2-(2-amin	19.6	4
TENT5B	115572	Leukemia, MESH:D015470	Calcitriol|D	19.6	13
ZCCHC24	219654	Leukemia, MESH:D015470	Dronabinc	19.6	9
RORC	6097	Leukemia, MESH:D015473	Arsenic|Bu	19.59	13
SLC3A2	6520	Leukemia, MESH:D015473	Arsenic|Ar	19.59	162
ALOX12	239	Leukemia, MESH:D015473	Calcitriol|C	19.58	8
SERPINE1	5054	Leukemia, MESH:D007948	2-(2-amin	19.58	3
ARL4C	10123	Leukemia, MESH:D015473	Arsenic|Ar	19.57	266
CENPI	2491	Leukemia, MESH:D015473	Calcitriol|D	19.57	153
RAB20	55647	Leukemia, MESH:D015473	Dexameth	19.57	154
SATB2	23314	Leukemia, MESH:D015473	Arsenic Tr	19.57	264
TMEM200	114801	Leukemia, MESH:D015473	Calcitriol|C	19.57	15
TNFSF14	8740	Leukemia, MESH:D015473	Arsenic Tr	19.57	263
XAF1	54739	Leukemia, MESH:D015473	Arsenic Tr	19.57	262
EFHD2	79180	Leukemia, MESH:D015473	arsenite|D	19.56	155
GPX8	493869	Leukemia, MESH:D015473	arsenite|Ca	19.56	157
KIF18B	146909	Leukemia, MESH:D015473	Arsenic|ars	19.56	157
MSI2	124540	Leukemia, MESH:D015473	Arsenic|ars	19.56	159
PMS1	5378	Leukemia, MESH:D015473	Dexameth	19.56	156
RPA3	6119	Leukemia, MESH:D015473	Arsenic|Ar	19.56	264
TNFRSF8	943	Leukemia, MESH:D015473	Arsenic|Ar	19.56	264
BCL3	602	Leukemia, MESH:D015473	Arsenic Tr	19.54	261
DKK1	22943	Leukemia, MESH:D015473	Arsenic Tr	19.54	259
RUSF1	64755	Leukemia, MESH:D015473	Arsenic|Ar	19.54	263
DDX3X	1654	Leukemia, MESH:D015470	Air Polluta	19.53	12
DTL	51514	Leukemia, MESH:D015470	Arsenic Tr	19.53	23
EGLN3	112399	Leukemia, MESH:D015470	Bortezomi	19.53	18
FOXO3	2309	Leukemia, MESH:D015473	Arsenic Tr	19.53	263
GUCY1B1	2983	Leukemia, MESH:D015470	Dexameth	19.53	26
IL16	3603	Leukemia, MESH:D015470	Benzene|C	19.53	26
PRDX4	10549	Leukemia, MESH:D015470	Arsenic Tr	19.53	22

PTPN6	5777	Leukemia, MESH:D015470	Arsenic Tr	19.53	22
SLC38A1	81539	Leukemia, MESH:D015470	Arsenic Tr	19.53	15
SLC5A3	6526	Leukemia, MESH:D015470	Allopurinc	19.53	15
CDH6	1004	Leukemia, MESH:D015470	Dexameth	19.51	14
CTNND2	1501	Leukemia, MESH:D015470	Cyclophos	19.51	14
DAGLB	221955	Leukemia, MESH:D015470	Dexameth	19.51	13
GINS3	64785	Leukemia, MESH:D015470	Calcitriol|I	19.51	11
LY6D	8581	Leukemia, MESH:D015470	Bezafibrat	19.51	14
MKNK1	8569	Leukemia, MESH:D015470	Arsenic Tr	19.51	27
NDUFS3	4722	Leukemia, MESH:D015470	Air Polluta	19.51	15
NFE2	4778	Leukemia, MESH:D015473	Aclarubici	19.51	159
NTHL1	4913	Leukemia, MESH:D015470	Bezafibrat	19.51	12
PARP2	10038	Leukemia, MESH:D015470	Arsenic Tr	19.51	21
POPDC2	64091	Leukemia, MESH:D015470	Arsenic Tr	19.51	19
RPL36A	6173	Leukemia, MESH:D015470	Arsenic Tr	19.51	29
SOCS5	9655	Leukemia, MESH:D015470	Bortezomi	19.51	10
TSPAN2	10100	Leukemia, MESH:D015470	Dasatinib|	19.51	9
UGT1A4	54657	Leukemia, MESH:D015470	Indometh	19.51	11
VPS13A	23230	Leukemia, MESH:D015470	Calcitriol|D	19.51	11
XCL1	6375	Leukemia, MESH:D015470	Bortezomi	19.51	9
ARHGEF28	64283	Leukemia, MESH:D015470	Calcitriol|D	19.5	14
DYRK2	8445	Leukemia, MESH:D015473	Arsenic Tr	19.5	262
FTO	79068	Leukemia, MESH:D015470	Dexameth	19.5	9
IFI44L	10964	Leukemia, MESH:D015473	Arsenic|M	19.5	155
KLHL29	114818	Leukemia, MESH:D015470	Dexameth	19.5	13
MAN1A2	10905	Leukemia, MESH:D015470	Arsenic Tr	19.5	15
MCOLN1	57192	Leukemia, MESH:D015470	Dexameth	19.5	9
NME3	4832	Leukemia, MESH:D015470	Dexameth	19.5	13
NRF1	4899	Leukemia, MESH:D015473	Arsenic|Ar	19.5	265
NTRK3	4916	Leukemia, MESH:D015473	Arsenic|Ar	19.5	265
PAPOLA	10914	Leukemia, MESH:D015473	Arsenic|De	19.5	159
PAX3	5077	Leukemia, MESH:D015473	alpha-Toc	19.5	158
PRPS1	5631	Leukemia, MESH:D015473	Arsenic|Ar	19.5	267
SLC48A1	55652	Leukemia, MESH:D015470	Arsenic Tr	19.5	14
TMEM117	84216	Leukemia, MESH:D015470	Calcitriol|D	19.5	11
TMEM50B	757	Leukemia, MESH:D015470	Dexameth	19.5	14
WDR77	79084	Leukemia, MESH:D015470	Arsenic Tr	19.5	19
XRCC2	7516	Leukemia, MESH:D015473	Calcitriol|D	19.5	155
ANO1	55107	Leukemia, MESH:D015473	Arsenic|De	19.49	158
B4GALT5	9334	Leukemia, MESH:D015473	Arsenic Tr	19.49	262
CDV3	55573	Leukemia, MESH:D015473	Arsenic Tr	19.49	263
FRAS1	80144	Leukemia, MESH:D015473	arsenite|C	19.49	156
HMGN2	3151	Leukemia, MESH:D015473	Arsenic Tr	19.49	156
MIR196B	442920	Leukemia, MESH:D015470	Air Polluta	19.49	28
MIR223	407008	Leukemia, MESH:D015470	Arsenic Tr	19.49	21
PDX1	3651	Leukemia, MESH:D015473	Arsenic|ar	19.49	160
PTK6	5753	Leukemia, MESH:D015470	Arsenic Tr	19.49	28
SUV39H1	6839	Leukemia, MESH:D015473	Arsenic|Ca	19.49	159
TJP3	27134	Leukemia, MESH:D015473	Arsenic|Ca	19.49	13
BIRC7	79444	Leukemia, MESH:D015470	Arsenic Tr	19.48	28
MTIF3	219402	Leukemia, MESH:D015470	Decitabine	19.48	21
PLEKHM2	23207	Leukemia, MESH:D015470	Arsenic Tr	19.48	14
SFPQ	6421	Leukemia, MESH:D015470	Arsenic Tr	19.48	22
ARNT	405	Leukemia, MESH:D015470	Deferoxan	19.47	28
CCDC14	64770	Leukemia, MESH:D015470	Calcitriol|D	19.47	14
CHCHD4	131474	Leukemia, MESH:D015470	Doxorubic	19.47	12
LMLN	89782	Leukemia, MESH:D015470	Doxorubic	19.47	12
MPV17L2	84769	Leukemia, MESH:D015470	Dexameth	19.47	14

PCNX2	80003	Leukemia, MESH:D015470	Dexameth	19.47	15
PLAC1	10761	Leukemia, MESH:D015470	Arsenic Tr	19.47	13
PLPPR4	9890	Leukemia, MESH:D015470	Doxorubic	19.47	15
RUSF1	64755	Leukemia, MESH:D015470	Arsenic Tr	19.47	20
SLC35C1	55343	Leukemia, MESH:D015470	Dexameth	19.47	12
TMEM18	129787	Leukemia, MESH:D015470	Arsenic Tr	19.47	19
ZNF394	84124	Leukemia, MESH:D015470	Doxorubic	19.47	13
ATG7	10533	Leukemia, MESH:D015473	Arsenic|ars	19.46	266
CD63	967	Leukemia, MESH:D015470	Air Polluta	19.46	27
ITGA5	3678	Leukemia, MESH:D015473	Arsenic|De	19.46	158
ITGA6	3655	Leukemia, MESH:D015473	Arsenic Tr	19.46	263
TPM4	7171	Leukemia, MESH:D015473	Arsenic Tr	19.46	264
VDAC2	7417	Leukemia, MESH:D015470	Arsenic Tr	19.46	20
YARS1	8565	Leukemia, MESH:D015470	Dexameth	19.46	12
ZNF473	25888	Leukemia, MESH:D015470	Dexameth	19.46	13
PCNA	5111	Leukemia, MESH:D007948	2-(2-amin	19.45	3
IL12A	3592	Leukemia, MESH:D015473	Arsenic|ars	19.44	158
PSD3	23362	Leukemia, MESH:D015473	Arsenic|Ar	19.44	267
DSG1	1828	Leukemia, MESH:D015473	Antimony	19.43	13
GAS6	2621	Leukemia, MESH:D015470	Decitabine	19.43	24
GSPT1	2935	Leukemia, MESH:D015473	Arsenic Tr	19.43	262
HPGD	3248	Leukemia, MESH:D007948	2-(2-amin	19.43	3
MTA1	9112	Leukemia, MESH:D015473	Arsenic|Ar	19.43	268
PIDD1	55367	Leukemia, MESH:D015473	Arsenic|Ca	19.43	158
POU2F1	5451	Leukemia, MESH:D015473	Arsenic Tr	19.43	263
PRPS2	5634	Leukemia, MESH:D015473	Arsenic Tr	19.43	263
SEL1L	6400	Leukemia, MESH:D015473	arsenite|D	19.43	9
SULT2B1	6820	Leukemia, MESH:D015473	Antimony	19.43	12
TNFSF10	8743	Leukemia, MESH:D004915	Cytarabine	19.43	3
TNFSF4	7292	Leukemia, MESH:D015473	Dexameth	19.43	154
ADA	100	Leukemia, MESH:D015473	caffeic aci	19.42	157
GSTM2	2946	Leukemia, MESH:D015470	Calcitriol|C	19.42	24
NFKBIB	4793	Leukemia, MESH:D015473	Arsenic Tr	19.42	263
PDIA4	9601	Leukemia, MESH:D015473	Antimony	19.42	160
RBP1	5947	Leukemia, MESH:D015473	Arsenic|Ar	19.42	264
SPARCL1	8404	Leukemia, MESH:D015473	Arsenic|Ca	19.42	157
TENM3	55714	Leukemia, MESH:D015473	arsenite|Ca	19.42	156
ZSCAN31	64288	Leukemia, MESH:D015473	Arsenic|Ar	19.42	266
AFF1	4299	Leukemia, MESH:D015470	Bortezomi	19.41	15
AMPD2	271	Leukemia, MESH:D015470	Benzene|D	19.41	23
ANXA13	312	Leukemia, MESH:D015470	Irinotecan	19.41	9
CFD	1675	Leukemia, MESH:D015473	Arsenic|Ar	19.41	265
CSGALNA	55790	Leukemia, MESH:D015470	Arsenic Tr	19.41	22
CYBRD1	79901	Leukemia, MESH:D015470	Air Polluta	19.41	20
EIF1AX	1964	Leukemia, MESH:D015470	Cyclophos	19.41	14
FAM89A	375061	Leukemia, MESH:D015470	Dronabinc	19.41	9
FNBP4	23360	Leukemia, MESH:D015470	Air Polluta	19.41	23
FOLR2	2350	Leukemia, MESH:D015470	Air Polluta	19.41	11
GAP43	2596	Leukemia, MESH:D007948	Arsenic Tr	19.41	4
KLF5	688	Leukemia, MESH:D007948	2-(2-amin	19.41	3
MAD2L1B	9587	Leukemia, MESH:D015473	arsenite|M	19.41	156
MCM8	84515	Leukemia, MESH:D015470	Calcitriol|D	19.41	19
NCKAP1	10787	Leukemia, MESH:D015470	Arsenic Tr	19.41	15
OSR1	130497	Leukemia, MESH:D015470	Cytarabine	19.41	82
PIGH	5283	Leukemia, MESH:D015473	Arsenic Tr	19.41	263
PLCB1	23236	Leukemia, MESH:D015473	Arsenic|Ar	19.41	264
POU3F1	5453	Leukemia, MESH:D015470	Calcitriol|D	19.41	9
PTGR2	145482	Leukemia, MESH:D015470	Doxorubic	19.41	17

SLC38A5	92745	Leukemia, MESH:D015470	Dexameth	19.41	12
SOSTDC1	25928	Leukemia, MESH:D015470	Arsenic Tr	19.41	17
STX11	8676	Leukemia, MESH:D015470	Benzene|D	19.41	24
TEAD4	7004	Leukemia, MESH:D015470	Calcitriol|D	19.41	14
TRMT61A	115708	Leukemia, MESH:D015470	Arsenic Tr	19.41	14
TSPAN33	340348	Leukemia, MESH:D015470	Benzene|D	19.41	23
WNT7B	7477	Leukemia, MESH:D015470	Calcitriol|D	19.41	15
CNKSR3	154043	Leukemia, MESH:D015470	Dexameth	19.4	14
DCLRE1B	64858	Leukemia, MESH:D015473	Arsenic|De	19.4	158
EBF3	253738	Leukemia, MESH:D015470	Dexameth	19.4	14
GATA4	2626	Leukemia, MESH:D007948	2-(2-amin	19.4	3
IL24	11009	Leukemia, MESH:D007948	2-(2-amin	19.4	3
MOB3B	79817	Leukemia, MESH:D015470	Dexameth	19.4	10
MTUS1	57509	Leukemia, MESH:D004915	Cytarabine	19.4	2
NAGLU	4669	Leukemia, MESH:D015470	Arsenic Tr	19.4	19
SLC39A11	201266	Leukemia, MESH:D015470	Calcitriol|D	19.4	11
AK1	203	Leukemia, MESH:D015470	Clioquinol	19.39	17
BASP1	10409	Leukemia, MESH:D015470	Arsenic Tr	19.39	23
KRT14	3861	Leukemia, MESH:D015470	Alitretinoi	19.39	16
PDK2	5164	Leukemia, MESH:D015470	Bezafibrat	19.39	18
PRKACA	5566	Leukemia, MESH:D015473	arsenite|ca	19.38	13
PSMG3-A	114796	Leukemia, MESH:D015470	Dexameth	19.38	11
RTL10	79680	Leukemia, MESH:D015470	Calcitriol|D	19.38	12
SP3	6670	Leukemia, MESH:D015473	Arsenic Tr	19.38	165
AZGP1	563	Leukemia, MESH:D015473	Antimony	19.37	159
CAPN2	824	Leukemia, MESH:D015473	Arsenic|Ar	19.37	266
CAVIN3	112464	Leukemia, MESH:D015473	Arsenic Tr	19.37	265
CYP2C19	1557	Leukemia, MESH:D015473	Calcitriol|D	19.37	156
GJB2	2706	Leukemia, MESH:D015473	arsenite|Ca	19.37	158
IL17RB	55540	Leukemia, MESH:D015473	Calcitriol|C	19.37	154
RNF175	285533	Leukemia, MESH:D015470	Pentachlo	19.37	7
SFPQ	6421	Leukemia, MESH:D015473	Arsenic|Ar	19.37	270
SMPDL3A	10924	Leukemia, MESH:D015473	Arsenic|Ar	19.37	264
ST3GAL5	8869	Leukemia, MESH:D004915	Cytarabine	19.37	2
WIPF1	7456	Leukemia, MESH:D015473	Arsenic|Ar	19.37	265
ZFHX4	79776	Leukemia, MESH:D015473	Arsenic|Ca	19.37	20
ZNF271P	10778	Leukemia, MESH:D015470	Dexameth	19.37	11
ZNF358	140467	Leukemia, MESH:D015470	Dexameth	19.37	6
ZNF439	90594	Leukemia, MESH:D015470	Dexameth	19.37	6
COL1A1	1277	Leukemia, MESH:D007948	2-(2-amin	19.36	4
HSPA5	3309	Leukemia, MESH:D007948	2-(2-amin	19.36	3
NOG	9241	Leukemia, MESH:D015473	Arsenic|ca	19.36	155
PRKAR2A	5576	Leukemia, MESH:D015473	Arsenic|ca	19.36	157
DSC2	1824	Leukemia, MESH:D004915	Cytarabine	19.35	3
GCLC	2729	Leukemia, MESH:D007948	2-(2-amin	19.35	3
SELE	6401	Leukemia, MESH:D015473	alpha-Toc	19.35	158
SLC38A2	54407	Leukemia, MESH:D015473	Arsenic|ars	19.34	162
ACAA2	10449	Leukemia, MESH:D015473	Arsenic Tr	19.33	261
CYB5D1	124637	Leukemia, MESH:D015470	Cytarabine	19.33	80
DLK2	65989	Leukemia, MESH:D015470	Calcitriol|D	19.33	10
NUSAP1	51203	Leukemia, MESH:D015473	Arsenic Tr	19.33	262
SCAMP4	113178	Leukemia, MESH:D015470	Arsenic Tr	19.33	16
SLC30A1	7779	Leukemia, MESH:D015473	Arsenic Tr	19.33	263
ZNRF2	223082	Leukemia, MESH:D015470	Bortezomi	19.33	15
ACTC1	70	Leukemia, MESH:D015470	Cytarabine	19.32	81
ALDH1L1	10840	Leukemia, MESH:D015470	Dexameth	19.32	10
CEBPG	1054	Leukemia, MESH:D015470	Dronabinc	19.32	13
CYP4F11	57834	Leukemia, MESH:D015470	Alitretinoi	19.32	13

EMSY	56946	Leukemia, MESH:D015470		Doxorubic	19.32	13
FAM219A	203259	Leukemia, MESH:D015470		Bortezomi	19.32	13
FMO2	2327	Leukemia, MESH:D015470		Calcitriol|[	19.32	17
GLIPR1	11010	Leukemia, MESH:D015470		Arsenic Tr	19.32	25
HESX1	8820	Leukemia, MESH:D015470		Decitabine	19.32	14
IRF9	10379	Leukemia, MESH:D015470		Dexameth	19.32	13
KLHL26	55295	Leukemia, MESH:D015470		Doxorubic	19.32	20
NCEH1	57552	Leukemia, MESH:D015470		Arsenic Tr	19.32	18
OST4	1E+08	Leukemia, MESH:D015470		Dexameth	19.32	11
PHOSPHC	493911	Leukemia, MESH:D015470		Arsenic Tr	19.32	14
PI3	5266	Leukemia, MESH:D015470		Doxorubic	19.32	17
PIGL	9487	Leukemia, MESH:D015470		Pentachlo	19.32	8
STOML1	9399	Leukemia, MESH:D015470		Calcitriol|[	19.32	7
ZUP1	221302	Leukemia, MESH:D015470		Benzene|[	19.32	21
KMT2C	58508	Leukemia, MESH:D01	marker/m	Arsenic Tr	19.31	20
ABHD3	171586	Leukemia, MESH:D015470		Arsenic Tr	19.31	15
AIF1L	83543	Leukemia, MESH:D015470		Cytarabine	19.31	79
AMIGO1	57463	Leukemia, MESH:D015470		Doxorubic	19.31	13
ATG2A	23130	Leukemia, MESH:D015470		Doxorubic	19.31	14
B4GALT6	9331	Leukemia, MESH:D015470		Bortezomi	19.31	10
CAMP	820	Leukemia, MESH:D015470		Arsenic Tr	19.31	16
CCDC121	79635	Leukemia, MESH:D015470		Calcitriol|[	19.31	8
CDH4	1002	Leukemia, MESH:D015470		Dronabinc	19.31	10
DDA1	79016	Leukemia, MESH:D015470		Dexameth	19.31	14
ELF4	2000	Leukemia, MESH:D015470		Decitabine	19.31	21
FA2H	79152	Leukemia, MESH:D015470		Arsenic Tr	19.31	16
FOXJ1	2302	Leukemia, MESH:D015470		Decitabine	19.31	21
HNRNPH3	3189	Leukemia, MESH:D015470		Dexameth	19.31	9
ITM2B	9445	Leukemia, MESH:D015470		Arsenic Tr	19.31	19
LDLRAD4	753	Leukemia, MESH:D015470		Dexameth	19.31	18
LIX1	167410	Leukemia, MESH:D015470		Doxorubic	19.31	15
MAD2L1B	9587	Leukemia, MESH:D015470		Dronabinc	19.31	12
MAP3K12	7786	Leukemia, MESH:D015470		Carboplati	19.31	14
MLYCD	23417	Leukemia, MESH:D015470		Doxorubic	19.31	15
NAA30	122830	Leukemia, MESH:D015470		Dexameth	19.31	13
NAB1	4664	Leukemia, MESH:D015470		Benzene|[	19.31	24
NANS	54187	Leukemia, MESH:D015470		Arsenic Tr	19.31	19
NIPAL2	79815	Leukemia, MESH:D015470		Calcitriol|[	19.31	13
NKTR	4820	Leukemia, MESH:D015470		Arsenic Tr	19.31	20
PGM5	5239	Leukemia, MESH:D015470		Cytarabine	19.31	83
PIEZO2	63895	Leukemia, MESH:D015470		Dexameth	19.31	14
RNF122	79845	Leukemia, MESH:D015470		Dexameth	19.31	14
RNF139	11236	Leukemia, MESH:D015470		Doxorubic	19.31	12
SH3RF1	57630	Leukemia, MESH:D015470		Doxorubic	19.31	15
SNRPN	6638	Leukemia, MESH:D015470		Dexameth	19.31	11
SOX5	6660	Leukemia, MESH:D015470		Dexameth	19.31	15
SRD5A3	79644	Leukemia, MESH:D015470		Doxorubic	19.31	15
TSC22D2	9819	Leukemia, MESH:D015470		Arsenic Tr	19.31	21
TTC39C	125488	Leukemia, MESH:D015470		Calcitriol|[	19.31	10
VTA1	51534	Leukemia, MESH:D015470		Dexameth	19.31	12
ADARB1	104	Leukemia, MESH:D015473		arsenite|D	19.3	157
CCR6	1235	Leukemia, MESH:D015473		Arsenic|De	19.3	158
FLRT3	23767	Leukemia, MESH:D015473		Arsenic Tr	19.3	263
LRPPRC	10128	Leukemia, MESH:D015473		Arsenic|ars	19.3	158
RASA1	5921	Leukemia, MESH:D015473		Arsenic|ars	19.3	159
RFTN1	23180	Leukemia, MESH:D015473		Arsenic|De	19.3	157
TNPO1	3842	Leukemia, MESH:D015473		arsenite|Ca	19.3	161
ALDH3A1	218	Leukemia, MESH:D015473		Arsenic|Ar	19.29	265

ALDH3A2	224	Leukemia,	MESH:D015473	Antimony	19.29	263
CAPN1	823	Leukemia,	MESH:D015473	Arsenic|Ar	19.29	266
HIVEP2	3097	Leukemia,	MESH:D015473	arsenite|Ca	19.29	156
KIF11	3832	Leukemia,	MESH:D015473	arsenite|Ca	19.29	157
MAP2K5	5607	Leukemia,	MESH:D015473	Arsenic|ars	19.29	158
MIR134	406924	Leukemia,	MESH:D015473	arsenite|Ca	19.29	9
SEMA3B	7869	Leukemia,	MESH:D015473	Calcitriol|D	19.29	156
STK39	27347	Leukemia,	MESH:D015473	Arsenic|Ca	19.29	158
UAP1	6675	Leukemia,	MESH:D015473	Arsenic|Ar	19.29	264
WNT5A	7474	Leukemia,	MESH:D015473	arsenite|Ca	19.29	157
YBX1	4904	Leukemia,	MESH:D015473	arsenite|D	19.29	157
GH1	2688	Leukemia,	MESH:D007948	2-(2-amin	19.28	3
NECAP2	55707	Leukemia,	MESH:D015473	arsenite|G	19.28	156
NTMT1	28989	Leukemia,	MESH:D015473	Arsenic|De	19.28	158
IL1RL1	9173	Leukemia,	MESH:D015470	Calcitriol|D	19.27	14
AGRN	375790	Leukemia,	MESH:D004915	Daunorub	19.26	3
DYNLL1	8655	Leukemia,	MESH:D015470	Decitabine	19.25	20
HNRNPDL	9987	Leukemia,	MESH:D015470	Doxorubic	19.25	20
MARCKS	4082	Leukemia,	MESH:D015473	Arsenic Tr	19.25	261
MDK	4192	Leukemia,	MESH:D004915	Cytarabine	19.25	3
RIPK3	11035	Leukemia,	MESH:D015470	Bortezomi	19.25	17
SLC37A4	2542	Leukemia,	MESH:D015470	Arsenic Tr	19.25	23
TPMT	7172	Leukemia,	MESH:D015473	Arsenic|Ar	19.25	159
EIF3B	8662	Leukemia,	MESH:D015473	Calcitriol|G	19.24	155
AKT3	10000	Leukemia,	MESH:D015473	Arsenic|ars	19.23	159
CD8A	925	Leukemia,	MESH:D015473	Arsenic|Cy	19.23	22
ENTPD1	953	Leukemia,	MESH:D015473	Arsenic|Cy	19.23	161
FGL1	2267	Leukemia,	MESH:D015473	Arsenic Tr	19.23	262
HDAC9	9734	Leukemia,	MESH:D015473	arsenite|G	19.23	157
MIR34A	407040	Leukemia,	MESH:D015473	arsenite|G	19.23	156
NAV1	89796	Leukemia,	MESH:D015473	Arsenic|ars	19.23	158
NCOA4	8031	Leukemia,	MESH:D015473	Arsenic Tr	19.23	157
RPS3A	6189	Leukemia,	MESH:D015473	arsenite|ca	19.23	9
CTSS	1520	Leukemia,	MESH:D015470	Arsenic Tr	19.22	24
EXOC4	60412	Leukemia,	MESH:D015470	Doxorubic	19.22	22
GAS2L3	283431	Leukemia,	MESH:D015470	Benzene|C	19.22	28
IDI1	3422	Leukemia,	MESH:D015470	Benzene|C	19.22	34
MPZ	4359	Leukemia,	MESH:D015470	Arsenic Tr	19.22	23
NPR1	4881	Leukemia,	MESH:D015470	Dexameth	19.22	14
SLCO3A1	28232	Leukemia,	MESH:D015473	Arsenic|Ar	19.22	265
ZFR	51663	Leukemia,	MESH:D015473	Arsenic|ars	19.22	160
ACVR2B	93	Leukemia,	MESH:D015470	Decitabine	19.21	17
ANXA10	11199	Leukemia,	MESH:D015470	Dexameth	19.21	14
ANXA4	307	Leukemia,	MESH:D015473	Arsenic|ars	19.21	163
AOPEP	84909	Leukemia,	MESH:D015470	Arsenic Tr	19.21	20
ARHGEF6	9459	Leukemia,	MESH:D015470	Calcitriol|D	19.21	19
CAMTA1	23261	Leukemia,	MESH:D015470	Decitabine	19.21	20
CENPH	64946	Leukemia,	MESH:D015470	Calcitriol|D	19.21	11
CENPJ	55835	Leukemia,	MESH:D015470	Calcitriol|D	19.21	9
CHGB	1114	Leukemia,	MESH:D015470	Arsenic Tr	19.21	18
COX15	1355	Leukemia,	MESH:D015470	Arsenic Tr	19.21	20
CSNK2B	1460	Leukemia,	MESH:D015470	Air Polluta	19.21	21
CYB5R1	51706	Leukemia,	MESH:D015470	Bortezomi	19.21	12
DLL4	54567	Leukemia,	MESH:D015470	Arsenic Tr	19.21	18
FAM126B	285172	Leukemia,	MESH:D015470	Dexameth	19.21	13
GMPR	2766	Leukemia,	MESH:D015470	Air Polluta	19.21	9
IL17RD	54756	Leukemia,	MESH:D015470	Doxorubic	19.21	13
JADE2	23338	Leukemia,	MESH:D015470	Doxorubic	19.21	17

NDUFA13	51079	Leukemia, MESH:D015470	Doxorubic	19.21	15
PAG1	55824	Leukemia, MESH:D015470	Dexameth	19.21	9
PFKP	5214	Leukemia, MESH:D015473	Arsenic Tr	19.21	262
PHKB	5257	Leukemia, MESH:D015470	Bortezomi	19.21	17
PITPNM1	9600	Leukemia, MESH:D015470	Dexameth	19.21	13
PTX3	5806	Leukemia, MESH:D015473	Arsenic|Ar	19.21	264
RAB6B	51560	Leukemia, MESH:D015470	Doxorubic	19.21	15
SPAG1	6674	Leukemia, MESH:D015470	Calcitriol|D	19.21	11
SRPRB	58477	Leukemia, MESH:D015470	Dexameth	19.21	15
ID2	3398	Leukemia, MESH:D015473	Arsenic|Ar	19.2	267
CNIH2	254263	Leukemia, MESH:D015473	Arsenic|Ca	19.19	18
RGS13	6003	Leukemia, MESH:D015473	Cytarabine	19.19	155
TSLP	85480	Leukemia, MESH:D015470	15-deoxy-	19.19	15
ASCL1B	30478	Leukemia, MESH:D015470	Dexameth	19.18	5
BAG3	9531	Leukemia, MESH:D007948	Arsenic Tr	19.18	4
CACNA2D	781	Leukemia, MESH:D015470	Arsenic Tr	19.18	32
GNAI3	2773	Leukemia, MESH:D015473	alpha-Toc	19.18	154
KLRK1	22914	Leukemia, MESH:D015473	Arsenic|Ar	19.18	158
MIR10B	406903	Leukemia, MESH:D015473	Arsenic Tr	19.18	261
MIR155	406947	Leukemia, MESH:D015473	Antineopl	19.18	263
MYSM1	114803	Leukemia, MESH:D015470	Doxorubic	19.18	16
OGA	10724	Leukemia, MESH:D015470	Dexameth	19.18	14
OGT	8473	Leukemia, MESH:D015470	Arsenic Tr	19.18	23
POLR2D	5433	Leukemia, MESH:D015473	arsenite|M	19.18	158
SLC39A10	57181	Leukemia, MESH:D015470	Arsenic Tr	19.18	22
SMARCA2	6595	Leukemia, MESH:D015470	Arsenic Tr	19.18	20
TLR2	7097	Leukemia, MESH:D015473	arsenic dis	19.18	260
ZSCAN12	9753	Leukemia, MESH:D015470	Decitabine	19.18	16
ANKRD36	645784	Leukemia, MESH:D015470	Dexameth	19.17	5
ASPRV1	151516	Leukemia, MESH:D015470	Dexameth	19.17	10
CTSC	1075	Leukemia, MESH:D015470	Arsenic Tr	19.17	17
CUZD1	50624	Leukemia, MESH:D015473	Arsenic|De	19.17	154
DOCK8	81704	Leukemia, MESH:D015473	arsenite|Ca	19.17	158
FKBP15	23307	Leukemia, MESH:D015470	Air Polluta	19.17	13
GLA	2717	Leukemia, MESH:D015473	Antimony	19.17	262
ILDR1	286676	Leukemia, MESH:D015470	Calcitriol|D	19.17	9
KLHDC4	54758	Leukemia, MESH:D015470	Benzene|D	19.17	27
LGR5	8549	Leukemia, MESH:D015473	Arsenic|Ar	19.17	160
NUP62CL	54830	Leukemia, MESH:D015470	Calcitriol|N	19.17	10
NYNRIN	57523	Leukemia, MESH:D015470	Decitabine	19.17	20
PLXDC2	84898	Leukemia, MESH:D015473	Arsenic|ars	19.17	13
RHOU	58480	Leukemia, MESH:D015473	Genistein|I	19.17	158
SIPA1L2	57568	Leukemia, MESH:D015473	Calcitriol|C	19.17	157
TBX21	30009	Leukemia, MESH:D015473	Arsenic|Bu	19.17	159
TNFRSF10	8794	Leukemia, MESH:D015473	Arsenic Tr	19.17	261
TSEN54	283989	Leukemia, MESH:D015470	Benzene|D	19.17	25
ZBED8	63920	Leukemia, MESH:D015470	Calcitriol|F	19.17	9
ZNF589	51385	Leukemia, MESH:D015470	Air Polluta	19.17	14
ZNF93	81931	Leukemia, MESH:D015470	Doxorubic	19.17	14
ARMH4	145407	Leukemia, MESH:D015470	Doxorubic	19.16	12
BMI1	648	Leukemia, MESH:D015473	Arsenic|Ar	19.16	263
CXCR6	10663	Leukemia, MESH:D015473	Arsenic|De	19.16	10
EXTL3	2137	Leukemia, MESH:D015473	Arsenic|ars	19.16	157
KPNA5	3841	Leukemia, MESH:D015470	Dexameth	19.16	12
LYRM9	201229	Leukemia, MESH:D015470	Doxorubic	19.16	13
NFKBIZ	64332	Leukemia, MESH:D015473	Arsenic|ars	19.16	160
NTMT1	28989	Leukemia, MESH:D015470	Dexameth	19.16	15
OIP5	11339	Leukemia, MESH:D015473	Calcitriol|D	19.16	156

PARS2	25973	Leukemia, MESH:D015470		Dexameth	19.16	12
RAI14	26064	Leukemia, MESH:D015473		Arsenic|ars	19.16	157
RASL11B	65997	Leukemia, MESH:D015473		Arsenic|ars	19.16	157
SHLD2	54537	Leukemia, MESH:D015470		Dexameth	19.16	13
TUBGCP5	114791	Leukemia, MESH:D015473		Dexameth	19.16	154
UBE2H	7328	Leukemia, MESH:D015473		Arsenic Tr	19.16	263
USP6NL	9712	Leukemia, MESH:D015470		Calcitriol|D	19.16	14
ZNF219	51222	Leukemia, MESH:D015470		Dexameth	19.16	13
ZBTB20	26137	Leukemia, MESH:D004915		Daunorub	19.14	3
FBN1	2200	Leukemia, MESH:D015473		arsenite|D	19.13	158
PPP1R3C	5507	Leukemia, MESH:D015473		arsenite|D	19.13	155
BACH2	60468	Leukemia, MESH:D01	marker/m	Arsenic Tr	19.12	22
ARAF	369	Leukemia, MESH:D015470		Dexameth	19.12	11
ARG1	383	Leukemia, MESH:D015473		Arsenic|Ar	19.12	265
BID	637	Leukemia, MESH:D004915		Cytarabine	19.12	2
CAPN6	827	Leukemia, MESH:D015470		Arsenic Tr	19.12	19
CDS2	8760	Leukemia, MESH:D015470		Dexameth	19.12	15
CELSR3	1951	Leukemia, MESH:D015470		Dexameth	19.12	14
CETN3	1070	Leukemia, MESH:D015470		Decitabine	19.12	14
DDHD1	80821	Leukemia, MESH:D015470		Doxorubic	19.12	14
EMILIN1	11117	Leukemia, MESH:D015470		Doxorubic	19.12	14
EPHA7	2045	Leukemia, MESH:D015470		Dexameth	19.12	14
FIGN	55137	Leukemia, MESH:D015470		Dronabinc	19.12	11
FLVCR2	55640	Leukemia, MESH:D015470		Doxorubic	19.12	14
FNIP1	96459	Leukemia, MESH:D015470		Arsenic Tr	19.12	21
HAGH	3029	Leukemia, MESH:D015470		Arsenic Tr	19.12	16
IER5	51278	Leukemia, MESH:D015470		Cyclophos	19.12	18
IFNA1	3439	Leukemia, MESH:D015470		Cyclophos	19.12	13
LYAR	55646	Leukemia, MESH:D015470		Calcitriol|D	19.12	11
MXD3	83463	Leukemia, MESH:D015470		Calcitriol|C	19.12	79
ND2	4536	Leukemia, MESH:D015470		Deferoxan	19.12	13
NDUFAF4	29078	Leukemia, MESH:D015470		Doxorubic	19.12	14
NIBAN1	116496	Leukemia, MESH:D004915		Doxorubic	19.12	3
OSR2	116039	Leukemia, MESH:D015470		Calcitriol|D	19.12	8
OVOS2	144203	Leukemia, MESH:D015470		Air Polluta	19.12	13
PDLIM5	10611	Leukemia, MESH:D015470		Arsenic Tr	19.12	21
PSMA2	5683	Leukemia, MESH:D015470		Dexameth	19.12	16
RAB1A	5861	Leukemia, MESH:D015470		Arsenic Tr	19.12	18
SASH1	23328	Leukemia, MESH:D015470		Arsenic Tr	19.12	15
SLC16A5	9121	Leukemia, MESH:D015470		Calcitriol|D	19.12	10
SLC25A36	55186	Leukemia, MESH:D015470		Dexameth	19.12	8
SLC29A3	55315	Leukemia, MESH:D015470		Decitabine	19.12	16
SNRPB	6628	Leukemia, MESH:D015470		Arsenic Tr	19.12	16
SPEG	10290	Leukemia, MESH:D015470		Arsenic Tr	19.12	21
STAB1	23166	Leukemia, MESH:D015470		Arsenic Tr	19.12	22
TFCP2L1	29842	Leukemia, MESH:D015470		Calcitriol|D	19.12	11
TIMM8B	26521	Leukemia, MESH:D015470		Dexameth	19.12	14
WASF2	10163	Leukemia, MESH:D015470		Dexameth	19.12	11
JAK3	3718	Leukemia, MESH:D015473		Arsenic|Ar	19.11	163
MEST	4232	Leukemia, MESH:D015473		arsenite|D	19.11	156
NAB2	4665	Leukemia, MESH:D015473		Arsenic Tr	19.11	260
PPIAL4A	653505	Leukemia, MESH:D015470		Dexameth	19.11	7
PTPN14	5784	Leukemia, MESH:D015470		Dexameth	19.11	14
SEPN1	1E+08	Leukemia, MESH:D015470		Dexameth	19.11	8
B4GALT1	2683	Leukemia, MESH:D015473		Calcitriol|D	19.1	154
C17ORF49	124944	Leukemia, MESH:D015470		Decitabine	19.1	14
GFPT2	9945	Leukemia, MESH:D015473		Arsenic|Ca	19.1	159
HHIP	64399	Leukemia, MESH:D015473		Calcitriol|D	19.1	156

PDE7B	27115	Leukemia, MESH:D015473	Arsenic|Ar	19.1	161
SREBF2	6721	Leukemia, MESH:D007948	2-(2-amir	19.1	2
TP53BP1	7158	Leukemia, MESH:D015473	Arsenic|De	19.1	158
C22ORF39	128977	Leukemia, MESH:D015470	Dexameth	19.09	11
ZNF414	84330	Leukemia, MESH:D015470	Dexameth	19.09	6
NR1I3	9970	Leukemia, MESH:D015470	Alitretinoi	19.08	33
LRRC15	131578	Leukemia, MESH:D015473	caffeic aci	19.07	153
PTGS2	5743	Leukemia, MESH:D007948	2-(2-amir	19.07	4
NLRP3	114548	Leukemia, MESH:D015473	Arsenic|Ar	19.06	264
TBCA	6902	Leukemia, MESH:D015473	arsenite|Bu	19.06	157
ABCB6	10058	Leukemia, MESH:D015470	Docetaxel|	19.05	12
AQP3	360	Leukemia, MESH:D007948	2-(2-amir	19.05	3
BAXA	58081	Leukemia, MESH:D015473	Dexameth	19.05	7
FLG	2312	Leukemia, MESH:D015473	Antimony	19.05	157
KCNN4	3783	Leukemia, MESH:D015470	Decitabine	19.05	20
MYLIP	29116	Leukemia, MESH:D015470	Calcitriol|C	19.05	82
MYLK	4638	Leukemia, MESH:D007948	2-(2-amir	19.05	3
PSMB1	5689	Leukemia, MESH:D015470	Arsenic Tr	19.05	21
SCO2	9997	Leukemia, MESH:D015473	Arsenic Tr	19.05	261
SRSF1	6426	Leukemia, MESH:D015470	Bortezomi	19.05	19
SRSF5	6430	Leukemia, MESH:D015470	Arsenic Tr	19.05	31
TENM1	10178	Leukemia, MESH:D015473	Arsenic|Ar	19.05	261
WIPI1	55062	Leukemia, MESH:D015470	Calcitriol|D	19.05	24
ETHE1	23474	Leukemia, MESH:D015473	Arsenic Tr	19.04	261
GCNT2	2651	Leukemia, MESH:D015473	Arsenic|ars	19.04	160
KMO	8564	Leukemia, MESH:D015473	Calcitriol|C	19.04	159
MSX1	4487	Leukemia, MESH:D015473	Arsenic|ars	19.04	160
OGN	4969	Leukemia, MESH:D015473	Arsenic Tr	19.04	260
PPP2CB	5516	Leukemia, MESH:D015473	Arsenic|Ar	19.04	160
PSME4	23198	Leukemia, MESH:D015473	Arsenic Tr	19.04	262
TACSTD2	4070	Leukemia, MESH:D015473	Arsenic Tr	19.04	261
ATP1B3	483	Leukemia, MESH:D015473	Arsenic|De	19.03	158
CXCR6	10663	Leukemia, MESH:D015470	Benzene|C	19.03	22
ECHDC1	55862	Leukemia, MESH:D015470	Air Polluta	19.03	14
MIP	4284	Leukemia, MESH:D015470	Alitretinoi	19.03	10
NUDT18	79873	Leukemia, MESH:D015470	Air Polluta	19.03	21
POLDIP2	26073	Leukemia, MESH:D015470	Arsenic Tr	19.03	36
PPP1R21	129285	Leukemia, MESH:D015470	Air Polluta	19.03	8
RNF4	6047	Leukemia, MESH:D015470	Arsenic Tr	19.03	21
SARS1	6301	Leukemia, MESH:D015470	Doxorubic	19.03	14
SORT1	6272	Leukemia, MESH:D004915	Doxorubic	19.03	3
SUSD2	56241	Leukemia, MESH:D015470	Dexameth	19.03	13
ANGEL2	90806	Leukemia, MESH:D015470	Dexameth	19.02	10
B3GALNT2	148789	Leukemia, MESH:D015470	Cyclophos	19.02	8
BEX1	55859	Leukemia, MESH:D015470	Dexameth	19.02	14
BMP2K	55589	Leukemia, MESH:D015470	Dexameth	19.02	13
BPTF	2186	Leukemia, MESH:D015470	Dexameth	19.02	15
CCDC102A	92922	Leukemia, MESH:D015470	Cytarabine	19.02	76
CD7	924	Leukemia, MESH:D015470	Benzene|C	19.02	21
CDC27	996	Leukemia, MESH:D015470	Arsenic Tr	19.02	20
CHST11	50515	Leukemia, MESH:D015470	Bortezomi	19.02	16
DCAKD	79877	Leukemia, MESH:D015470	Dexameth	19.02	15
DNAAF3	352909	Leukemia, MESH:D015470	Doxorubic	19.02	12
HCN1	348980	Leukemia, MESH:D015470	Doxorubic	19.02	12
HIP1R	9026	Leukemia, MESH:D015470	Arsenic Tr	19.02	21
LACTB	114294	Leukemia, MESH:D015470	Decitabine	19.02	20
LPCAT3	10162	Leukemia, MESH:D015470	Dexameth	19.02	11
MEX3A	92312	Leukemia, MESH:D015470	Calcitriol|D	19.02	7

NIPSNAP1	8508	Leukemia, MESH:D015470	Bortezomi	19.02	21
NPEPPS	9520	Leukemia, MESH:D015470	Dexameth	19.02	14
PDCD11	22984	Leukemia, MESH:D015470	Dexameth	19.02	18
PDE6B	5158	Leukemia, MESH:D015470	Dexameth	19.02	8
PHF12	57649	Leukemia, MESH:D015470	Arsenic Tr	19.02	22
PSMF1	9491	Leukemia, MESH:D015470	Bortezomi	19.02	14
RGS13	6003	Leukemia, MESH:D015470	Cytarabine	19.02	83
RIPOR1	79567	Leukemia, MESH:D015470	Calcitriol|D	19.02	20
RWDD3	25950	Leukemia, MESH:D015470	Dexameth	19.02	9
SCIN	85477	Leukemia, MESH:D015470	Arsenic Tr	19.02	20
TBCC	6903	Leukemia, MESH:D015470	Dexameth	19.02	13
TMTC4	84899	Leukemia, MESH:D015470	Dexameth	19.02	9
TRIP11	9321	Leukemia, MESH:D015470	Air Polluta	19.02	20
TRMT13	54482	Leukemia, MESH:D015470	Dronabinc	19.02	8
VAMP5	10791	Leukemia, MESH:D015470	Calcitriol|D	19.02	13
WSB2	55884	Leukemia, MESH:D015470	Arsenic Tr	19.02	14
ZNF146	7705	Leukemia, MESH:D015470	Air Polluta	19.02	13
ABRACL	58527	Leukemia, MESH:D015470	Dexameth	19.01	8
FAM91A1	157769	Leukemia, MESH:D015470	Dexameth	19.01	12
KMT2A	4297	Leukemia, MESH:D004915	Cytarabine	19.01	2
CHEK1	1111	Leukemia, MESH:D015473	Arsenic Tr	19	263
FABP3	2170	Leukemia, MESH:D015473	Arsenic|ars	19	17
PLD1	5337	Leukemia, MESH:D015473	Arsenic|ars	19	160
PERP	64065	Leukemia, MESH:D015473	alvocidib|a	18.99	155
RBBP8	5932	Leukemia, MESH:D015473	Calcitriol|C	18.99	156
CAMKK2	10645	Leukemia, MESH:D015473	Arsenic|ars	18.98	12
DRAM1	55332	Leukemia, MESH:D015473	arsenite|M	18.98	157
FAT1	2195	Leukemia, MESH:D015470	Arsenic Tr	18.98	20
HSPG2	3339	Leukemia, MESH:D015473	arsenite|Ca	18.98	155
ILF3	3609	Leukemia, MESH:D015473	Dexameth	18.98	159
MBNL2	10150	Leukemia, MESH:D015470	Air Polluta	18.98	21
NFKB1	4790	Leukemia, MESH:D007948	2-(2-amin	18.98	3
RAF1	5894	Leukemia, MESH:D015473	2-(2-chlor	18.98	265
SLC22A18	5002	Leukemia, MESH:D015473	Arsenic|Ar	18.98	264
TAX1BP3	30851	Leukemia, MESH:D015473	Daunorub	18.98	160
CFL1	1072	Leukemia, MESH:D015473	Arsenic|Ar	18.97	265
NAV3	89795	Leukemia, MESH:D015473	Arsenic Tr	18.97	261
ST8SIA4	7903	Leukemia, MESH:D015473	Arsenic Tr	18.97	263
BCHE	590	Leukemia, MESH:D015470	Arsenic Tr	18.96	51
EGFR	1956	Leukemia, MESH:D007948	2-(2-amin	18.96	3
ABCC1	4363	Leukemia, MESH:D004915	Cytarabine	18.94	3
COQ2	27235	Leukemia, MESH:D015473	Arsenic|Ar	18.94	159
CYP2A4	13086	Leukemia, MESH:D015470	Benzene|C	18.94	35
CYP2C18	1562	Leukemia, MESH:D015470	Cyclophos	18.94	17
POLK	51426	Leukemia, MESH:D015470	Benzene|D	18.94	26
PRRG4	79056	Leukemia, MESH:D015470	Hydroxyur	18.94	11
ARNTL	406	Leukemia, MESH:D015473	Arsenic|De	18.93	155
B3GALNT1	8706	Leukemia, MESH:D015470	Decitabine	18.93	21
CACNB3	784	Leukemia, MESH:D015470	Decitabine	18.93	19
DCUN1D4	23142	Leukemia, MESH:D015470	Dexameth	18.93	12
DOCK11	139818	Leukemia, MESH:D015470	Calcitriol|D	18.93	9
FAM81A	145773	Leukemia, MESH:D015473	Calcitriol|D	18.93	154
HADH	3033	Leukemia, MESH:D015473	Arsenic Tr	18.93	261
HCAR2	338442	Leukemia, MESH:D015473	Cholesterc	18.93	153
JAG2	3714	Leukemia, MESH:D015470	Arsenic Tr	18.93	20
KRT2	3849	Leukemia, MESH:D015473	Arsenic|Ca	18.93	156
LAT	27040	Leukemia, MESH:D015470	Arsenic Tr	18.93	17
LYPD1	116372	Leukemia, MESH:D015470	Decitabine	18.93	20

MGA	23269	Leukemia, MESH:D015473	Arsenic|Re	18.93	158
PGRMC2	10424	Leukemia, MESH:D015470	Dexameth	18.93	15
PIK3IP1	113791	Leukemia, MESH:D015470	Calcitriol|D	18.93	9
RAB27A	5873	Leukemia, MESH:D015470	Alitretinoi	18.93	9
RAI1	10743	Leukemia, MESH:D015470	Dexameth	18.93	14
RFC1	5981	Leukemia, MESH:D015470	Dexameth	18.93	24
RIMBP2	23504	Leukemia, MESH:D015473	Arsenic|Ar	18.93	263
SAR1B	51128	Leukemia, MESH:D015470	Dexameth	18.93	9
SCN8A	6334	Leukemia, MESH:D015470	Dexameth	18.93	9
SLC2A12	154091	Leukemia, MESH:D015470	Dexameth	18.93	14
SMAD9	4093	Leukemia, MESH:D015470	Decitabine	18.93	21
SPA17	53340	Leukemia, MESH:D015470	Doxorubic	18.93	13
SYT7	9066	Leukemia, MESH:D015470	Decitabine	18.93	21
TBX6	6911	Leukemia, MESH:D015473	Arsenic Tr	18.93	259
TCF21	6943	Leukemia, MESH:D015470	Cytarabine	18.93	83
TRAM1	23471	Leukemia, MESH:D015470	Dexameth	18.93	14
ADAMTS9	56999	Leukemia, MESH:D015473	Calcitriol|C	18.92	157
DDR1	780	Leukemia, MESH:D015473	arsenite|Ca	18.92	155
DDX21	9188	Leukemia, MESH:D015470	Arsenic Tr	18.92	19
ELN	2006	Leukemia, MESH:D015470	Calcitriol|D	18.92	16
ERP29	10961	Leukemia, MESH:D015470	Deferoxan	18.92	17
MR1	3140	Leukemia, MESH:D015473	Arsenic|Ar	18.92	263
PELI1	57162	Leukemia, MESH:D015473	Arsenic|Ar	18.92	267
TIMP2	7077	Leukemia, MESH:D015473	Arsenic|Ar	18.92	265
TMPRSS2	7113	Leukemia, MESH:D015473	Calcitriol|D	18.92	156
TRPV6	55503	Leukemia, MESH:D015473	arsenite|Ca	18.92	7
VEGFD	2277	Leukemia, MESH:D015470	Arsenic Tr	18.92	17
YDJC	150223	Leukemia, MESH:D015473	arsenite|D	18.92	154
AFF4	27125	Leukemia, MESH:D015473	Arsenic|Ar	18.91	161
RPL7	6129	Leukemia, MESH:D015473	Arsenic|Ar	18.91	266
SCPEP1	59342	Leukemia, MESH:D015473	Arsenic Tr	18.91	262
NOS2A	404036	Leukemia, MESH:D015470	Arsenic Tr	18.9	18
CYP7B1	9420	Leukemia, MESH:D015473	Arsenic|Ar	18.89	160
HLA-DQA	3117	Leukemia, MESH:D015470	Benzene|C	18.89	23
MUC16	94025	Leukemia, MESH:D015470	Air Polluta	18.89	9
NEMP1	23306	Leukemia, MESH:D015470	Calcitriol|D	18.89	15
RBBP5	5929	Leukemia, MESH:D015470	Daunorub	18.89	38
CAMTA2	23125	Leukemia, MESH:D015470	Decitabine	18.88	20
CCDC12	151903	Leukemia, MESH:D015470	Gasoline|I	18.88	12
CPT1B	1375	Leukemia, MESH:D015473	Arsenic|Ar	18.88	264
DCTN5	84516	Leukemia, MESH:D015470	Doxorubic	18.88	13
FAM110A	83541	Leukemia, MESH:D015470	Calcitriol|C	18.88	7
FYB1	2533	Leukemia, MESH:D015470	Arsenic Tr	18.88	16
GGA3	23163	Leukemia, MESH:D015470	Arsenic Tr	18.88	22
NSUN6	221078	Leukemia, MESH:D015470	Calcitriol|D	18.88	13
PIGZ	80235	Leukemia, MESH:D015470	(+)-JQ1 cc	18.88	10
RPP25L	138716	Leukemia, MESH:D015470	Decitabine	18.88	19
SCD5	79966	Leukemia, MESH:D015470	Pentachlo	18.88	11
SETD1A	9739	Leukemia, MESH:D015470	Decitabine	18.88	15
SGSH	6448	Leukemia, MESH:D015470	Bortezomi	18.88	13
SMG7	9887	Leukemia, MESH:D015470	Doxorubic	18.88	13
TAF12	6883	Leukemia, MESH:D015470	Dexameth	18.88	9
TENM1	10178	Leukemia, MESH:D015470	Arsenic Tr	18.88	15
TRMT2A	27037	Leukemia, MESH:D015470	Dexameth	18.88	12
ZBTB8A	653121	Leukemia, MESH:D015470	Indometha	18.88	9
ADAL	161823	Leukemia, MESH:D015470	Doxorubic	18.87	13
C15ORF39	56905	Leukemia, MESH:D015470	Dexameth	18.87	15
CRISPLD1	83690	Leukemia, MESH:D015470	Calcitriol|D	18.87	13

EDC3	80153	Leukemia, MESH:D015470	Dexameth	18.87	12
GAD1	2571	Leukemia, MESH:D015470	Decitabine	18.87	24
GTF3C6	112495	Leukemia, MESH:D015470	Doxorubic	18.87	12
HIPK2	28996	Leukemia, MESH:D004915	Doxorubic	18.87	3
INTS10	55174	Leukemia, MESH:D015470	Dexameth	18.87	13
LRRC42	115353	Leukemia, MESH:D015470	Decitabine	18.87	15
PIGK	10026	Leukemia, MESH:D015470	Dexameth	18.87	14
PTPRT	11122	Leukemia, MESH:D015470	Dronabinc	18.87	8
SMARCD1	6602	Leukemia, MESH:D015470	Dexameth	18.87	13
SRD5A1	6715	Leukemia, MESH:D015470	Androgen	18.87	31
TMEM147	10430	Leukemia, MESH:D015470	Doxorubic	18.87	13
TOM1L1	10040	Leukemia, MESH:D015470	Dexameth	18.87	13
ZW10	9183	Leukemia, MESH:D015470	Arsenic Tr	18.87	18
CTSB	1508	Leukemia, MESH:D015473	Arsenic|Ar	18.86	264
FOXA2	3170	Leukemia, MESH:D015470	Dexameth	18.86	12
MMP14	4323	Leukemia, MESH:D007948	2-(2-amin	18.86	2
RPA2	6118	Leukemia, MESH:D015473	arsenite|Ca	18.86	10
C11ORF80	79703	Leukemia, MESH:D015470	Calcitriol|I	18.85	6
CBX3	11335	Leukemia, MESH:D015470	Alitretinoi	18.85	18
DLK1	8788	Leukemia, MESH:D015473	Arsenic|Ar	18.85	263
FOSL2	2355	Leukemia, MESH:D015473	alpha-Toc	18.85	157
GAP43	2596	Leukemia, MESH:D015473	Arsenic|Ar	18.85	268
GNG11	2791	Leukemia, MESH:D015473	arsenite|D	18.85	155
GRIA4	2893	Leukemia, MESH:D015470	Benzoates	18.85	15
HSPE1	3336	Leukemia, MESH:D015473	Arsenic|Ca	18.85	159
KIAA1522	57648	Leukemia, MESH:D015470	Arsenic Tr	18.85	14
PID1	55022	Leukemia, MESH:D015470	Dexameth	18.85	16
RPS6KA3	6197	Leukemia, MESH:D015470	Arsenic Tr	18.85	22
S100A14	57402	Leukemia, MESH:D015470	Arsenic Tr	18.85	16
SET	6418	Leukemia, MESH:D015473	Arsenic|Ar	18.85	264
SIRPA	140885	Leukemia, MESH:D015473	Arsenic Tr	18.85	263
WNT3A	89780	Leukemia, MESH:D015473	Arsenic|De	18.85	158
ARID1B	57492	Leukemia, MESH:D015470	Arsenic Tr	18.84	22
CCDC88A	55704	Leukemia, MESH:D015470	Doxorubic	18.84	15
COX6A1	1337	Leukemia, MESH:D015470	Dexameth	18.84	16
CTPS2	56474	Leukemia, MESH:D015470	Arsenic Tr	18.84	15
DMGDH	29958	Leukemia, MESH:D015470	Calcitriol|D	18.84	12
FGF10	2255	Leukemia, MESH:D015470	Arsenic Tr	18.84	17
GCA	25801	Leukemia, MESH:D015470	Air Polluta	18.84	79
INPPL1	3636	Leukemia, MESH:D015470	Arsenic Tr	18.84	20
KRT13	3860	Leukemia, MESH:D015470	Arsenic Tr	18.84	16
MGAT2	4247	Leukemia, MESH:D015470	Dexameth	18.84	10
MTAP	4507	Leukemia, MESH:D015470	Arsenic Tr	18.84	22
NOC3L	64318	Leukemia, MESH:D015470	Dexameth	18.84	11
NONO	4841	Leukemia, MESH:D015470	Dexameth	18.84	14
PACSIN2	11252	Leukemia, MESH:D015470	Arsenic Tr	18.84	21
PCSK1	5122	Leukemia, MESH:D015470	Arsenic Tr	18.84	27
PPID	5481	Leukemia, MESH:D015470	Dexameth	18.84	12
PPP1R14A	94274	Leukemia, MESH:D015470	Decitabine	18.84	16
PTPRB	5787	Leukemia, MESH:D015470	Doxorubic	18.84	15
PTPRN2	5799	Leukemia, MESH:D015470	Arsenic Tr	18.84	16
RBM33	155435	Leukemia, MESH:D015470	Doxorubic	18.84	15
RBM8A	9939	Leukemia, MESH:D015470	Doxorubic	18.84	18
RNF13	11342	Leukemia, MESH:D015470	Arsenic Tr	18.84	19
SCN4B	6330	Leukemia, MESH:D015470	Arsenic Tr	18.84	27
SYBU	55638	Leukemia, MESH:D015470	Calcitriol|D	18.84	11
TGOLN2	10618	Leukemia, MESH:D015470	Arsenic Tr	18.84	33
TMEM106	54664	Leukemia, MESH:D015470	Calcitriol|D	18.84	14

TMOD1	7111	Leukemia, MESH:D015470	Calcitriol|D	18.84	15
TRAF5	7188	Leukemia, MESH:D015470	Arsenic Tr	18.84	14
UQCRH	7388	Leukemia, MESH:D015470	Arsenic Tr	18.84	29
ADIPOQ	9370	Leukemia, MESH:D015473	Arsenic|Ar	18.83	170
FBP1B	282672	Leukemia, MESH:D015470	Dexameth	18.83	7
HOXB8	3218	Leukemia, MESH:D015473	arsenite|Cy	18.83	155
MAP2K2	5605	Leukemia, MESH:D007948	2-(2-amin	18.83	3
NAT16	375607	Leukemia, MESH:D015470	Dexameth	18.83	7
PCDHGA3	56112	Leukemia, MESH:D015470	Dexameth	18.83	13
PCED1B	91523	Leukemia, MESH:D015473	Arsenic|Cy	18.83	158
SGK1	6446	Leukemia, MESH:D015473	Arsenic|Ar	18.83	265
STAT3	6774	Leukemia, MESH:D007948	2-(2-amin	18.83	3
SYTL3	94120	Leukemia, MESH:D015473	Calcitriol|N	18.83	153
ZBED10P	113763	Leukemia, MESH:D015470	Calcitriol|(	18.83	7
C3ORF33	285315	Leukemia, MESH:D015470	(+)-JQ1 cc	18.82	7
HECTD4	283450	Leukemia, MESH:D015473	arsenite|D	18.82	155
IQCN	80726	Leukemia, MESH:D015470	Dronabinc	18.82	7
PRDX6	9588	Leukemia, MESH:D015470	Calcitriol|D	18.82	23
SLC22A2	6582	Leukemia, MESH:D015470	Aclarubici	18.82	16
ST20-AS1	283687	Leukemia, MESH:D015470	Dexameth	18.82	7
ZNF513	130557	Leukemia, MESH:D015470	Doxorubic	18.82	12
ATP1B1	481	Leukemia, MESH:D015473	Arsenic Tr	18.81	263
BAX	581	Leukemia, MESH:D007948	2-(2-amin	18.81	4
GATA4	2626	Leukemia, MESH:D015473	Arsenic|Ar	18.81	266
MYF5	4617	Leukemia, MESH:D015473	Dexameth	18.81	155
SLC1A2	6506	Leukemia, MESH:D007948	2-(2-amin	18.81	3
SLCO2B1	11309	Leukemia, MESH:D015473	Arsenic|Ar	18.81	162
SOD2	6648	Leukemia, MESH:D004915	Daunorub	18.81	4
TRUB1	142940	Leukemia, MESH:D015473	Arsenic Tr	18.81	262
UCP1	7350	Leukemia, MESH:D015473	Arsenic|Ar	18.81	264
ARHGAP1	93663	Leukemia, MESH:D015473	Arsenic Tr	18.8	261
CASP6	839	Leukemia, MESH:D007948	Arsenic Tr	18.8	3
CYLD	1540	Leukemia, MESH:D015473	Arsenic Tr	18.8	261
MMD	23531	Leukemia, MESH:D015473	Arsenic Tr	18.8	157
SMAD6	4091	Leukemia, MESH:D015473	Arsenic Tr	18.8	264
COL14A1	7373	Leukemia, MESH:D015473	Arsenic Tr	18.79	261
ERCC2	2068	Leukemia, MESH:D015473	Arsenic|Ar	18.79	265
FGF9	2254	Leukemia, MESH:D015470	Dasatinib|I	18.79	15
LOXL1	4016	Leukemia, MESH:D015470	Calcitriol|D	18.79	15
MAP4	4134	Leukemia, MESH:D015473	Arsenic|De	18.79	158
MT1H	4496	Leukemia, MESH:D015473	Arsenic|Ar	18.79	161
PNP	4860	Leukemia, MESH:D015470	Arsenic Tr	18.79	21
PXDN	7837	Leukemia, MESH:D015473	Arsenic|Ar	18.79	159
SLCO2B1	11309	Leukemia, MESH:D015470	Arsenic Tr	18.77	27
ADORA3	140	Leukemia, MESH:D015470	Calcitriol|D	18.76	15
FGFBP1	9982	Leukemia, MESH:D015470	Calcitriol|(	18.76	10
HEXIM1	10614	Leukemia, MESH:D015470	Doxorubic	18.76	15
RBM5	10181	Leukemia, MESH:D015470	Air Polluta	18.76	17
SLC2A9	56606	Leukemia, MESH:D015470	Allopurinc	18.76	12
CBFB	865	Leukemia, MESH:D01 marker/me	Doxorubic	18.75	17
ANKRD10	55608	Leukemia, MESH:D015470	Decitabine	18.75	31
C2CD2	25966	Leukemia, MESH:D015470	Cytarabine	18.75	76
CLMP	79827	Leukemia, MESH:D015470	Dexameth	18.75	13
COBL	23242	Leukemia, MESH:D015470	Dexameth	18.75	22
CSF2RA	1438	Leukemia, MESH:D015470	Benzene|D	18.75	21
DOCK10	55619	Leukemia, MESH:D015470	Dexameth	18.75	14
EEF1E1	9521	Leukemia, MESH:D015470	Arsenic Tr	18.75	16
ELK4	2005	Leukemia, MESH:D015470	Arsenic Tr	18.75	20

ERN1	2081	Leukemia, MESH:D015473	Arsenic Tr	18.75	264
FNBP1L	54874	Leukemia, MESH:D015470	Arsenic Tr	18.75	14
GALNT10	55568	Leukemia, MESH:D015470	Cytarabine	18.75	81
HOXA11	3207	Leukemia, MESH:D015470	Bortezomi	18.75	19
HSPB7	27129	Leukemia, MESH:D015470	Dexameth	18.75	14
IER5L	389792	Leukemia, MESH:D015470	Arsenic Tr	18.75	15
LIMD1	8994	Leukemia, MESH:D015470	Arsenic Tr	18.75	16
MANBA	4126	Leukemia, MESH:D015470	Decitabine	18.75	16
MIR15A	406948	Leukemia, MESH:D015470	Arsenic Tr	18.75	20
MYH14	79784	Leukemia, MESH:D015470	Calcitriol|D	18.75	15
PIAS1	8554	Leukemia, MESH:D015470	Arsenic Tr	18.75	16
PXYLP1	92370	Leukemia, MESH:D015470	Dexameth	18.75	14
RAP1B	5908	Leukemia, MESH:D015470	Arsenic Tr	18.75	22
SH2D2A	9047	Leukemia, MESH:D015470	Benzene|D	18.75	23
SORCS2	57537	Leukemia, MESH:D015470	Doxorubic	18.75	17
SRP19	6728	Leukemia, MESH:D015470	Gasoline|I	18.75	9
TNFSF15	9966	Leukemia, MESH:D015470	Arsenic Tr	18.75	16
TNRC6B	23112	Leukemia, MESH:D015470	Dexameth	18.75	11
VAV2	7410	Leukemia, MESH:D015470	Arsenic Tr	18.75	22
ZMYM3	9203	Leukemia, MESH:D015470	Dexameth	18.75	16
BFSP1	631	Leukemia, MESH:D015470	(+)-JQ1 cc	18.74	8
CEP95	90799	Leukemia, MESH:D015470	Doxorubic	18.74	13
DEAF1	10522	Leukemia, MESH:D015470	Bortezomi	18.74	14
DMBT1	1755	Leukemia, MESH:D015473	Arsenic|Ge	18.74	161
FAM120B	84498	Leukemia, MESH:D015470	Decitabine	18.74	13
GINS1	9837	Leukemia, MESH:D015473	Calcitriol|D	18.74	155
H4C14	8370	Leukemia, MESH:D015470	Decitabine	18.74	18
KDM1B	221656	Leukemia, MESH:D015470	Air Polluta	18.74	12
KHNYN	23351	Leukemia, MESH:D015470	Benzene|P	18.74	23
MGA	23269	Leukemia, MESH:D015470	Doxorubic	18.74	18
MOAP1	64112	Leukemia, MESH:D015470	Air Polluta	18.74	13
NICN1	84276	Leukemia, MESH:D015470	Dexameth	18.74	12
NIPAL4	348938	Leukemia, MESH:D015470	Air Polluta	18.74	14
PDCD7	10081	Leukemia, MESH:D015470	Dexameth	18.74	12
PSMC2	5701	Leukemia, MESH:D015473	Arsenic|ars	18.74	266
SULT1C2	6819	Leukemia, MESH:D015473	Arsenic Tr	18.74	260
TTC38	55020	Leukemia, MESH:D015470	Arsenic Tr	18.74	20
VSTM4	196740	Leukemia, MESH:D015470	Calcitriol|D	18.74	14
ACOT7	11332	Leukemia, MESH:D015473	Arsenic|ars	18.73	160
ADAMTSL	92949	Leukemia, MESH:D015470	Dexameth	18.73	12
ANKS6	203286	Leukemia, MESH:D015470	Doxorubic	18.73	13
CDH11	1009	Leukemia, MESH:D015473	Arsenic|ars	18.73	160
CKAP4	10970	Leukemia, MESH:D015473	Arsenic|Ar	18.73	262
FAN1	22909	Leukemia, MESH:D015470	Dexameth	18.73	13
GNAO1	2775	Leukemia, MESH:D015473	Arsenic|ars	18.73	158
GNE	10020	Leukemia, MESH:D015473	Arsenic|Ar	18.73	265
LAMA1	284217	Leukemia, MESH:D015473	Arsenic|ars	18.73	158
LPCAT1	79888	Leukemia, MESH:D015473	Arsenic|Ar	18.73	265
LTBP2	4053	Leukemia, MESH:D015473	Arsenic|ars	18.73	12
MAP2K3	5606	Leukemia, MESH:D004915	Daunorub	18.73	2
MAP3K9	4293	Leukemia, MESH:D015470	Doxorubic	18.73	14
MMP8	4317	Leukemia, MESH:D015473	Arsenic|ars	18.73	158
PEX11A	8800	Leukemia, MESH:D015470	Dexameth	18.73	12
PF4	5196	Leukemia, MESH:D015470	Benzene|D	18.73	32
PGD	5226	Leukemia, MESH:D015473	Antimony	18.73	267
PLEKHF1	79156	Leukemia, MESH:D015473	Arsenic|ars	18.73	13
PRDM15	63977	Leukemia, MESH:D015470	Doxorubic	18.73	13
SEMA6D	80031	Leukemia, MESH:D015473	Arsenic Tr	18.73	263

TALDO1	6888	Leukemia, MESH:D015470	Arsenic Tr	18.73	22
TMEM245	23731	Leukemia, MESH:D015470	Arsenic Tr	18.73	18
TPR	7175	Leukemia, MESH:D015473	Dexameth	18.73	159
TRABD	80305	Leukemia, MESH:D015470	Dexameth	18.73	9
UNC5D	137970	Leukemia, MESH:D015470	Arsenic Tr	18.73	18
CASP2	835	Leukemia, MESH:D007948	Arsenic Tr	18.72	3
CKM	1158	Leukemia, MESH:D004915	Daunorub	18.72	3
JUND	3727	Leukemia, MESH:D007948	Arsenic Tr	18.72	3
MRPS16	51021	Leukemia, MESH:D015473	Arsenic Tr	18.72	158
NR1H3	10062	Leukemia, MESH:D007948	Arsenic Tr	18.72	3
PPM1M	132160	Leukemia, MESH:D015473	Calcitriol|C	18.72	156
S100A6	6277	Leukemia, MESH:D015470	Arsenic Tr	18.72	28
SCARF2	91179	Leukemia, MESH:D015473	Arsenic|Ar	18.72	262
CEP63	80254	Leukemia, MESH:D015473	Arsenic|Ca	18.71	10
CHML	1122	Leukemia, MESH:D015473	Arsenic|Ar	18.71	264
HNRNPUL	221092	Leukemia, MESH:D015473	Dexameth	18.71	7
IPO13	9670	Leukemia, MESH:D015473	Calcitriol|D	18.71	154
MRPL48	51642	Leukemia, MESH:D015473	arsenite|D	18.71	155
PROM2	150696	Leukemia, MESH:D015473	Arsenic|Ca	18.71	158
SDR16C5	195814	Leukemia, MESH:D015473	Arsenic|ars	18.71	157
SOX1	6656	Leukemia, MESH:D015473	Arsenic|Ar	18.71	261
TRAPPC1C	7109	Leukemia, MESH:D015473	Arsenic|ars	18.71	12
SCML2	10389	Leukemia, MESH:D015473	Calcitriol|D	18.7	156
SLC35F3	148641	Leukemia, MESH:D015473	arsenite|D	18.7	155
STAT5B	6777	Leukemia, MESH:D015473	Arsenic Tr	18.7	265
BCL6	604	Leukemia, MESH:D015473	Arsenic|Ar	18.69	264
HP	3240	Leukemia, MESH:D015473	alpha-Toc	18.69	157
CEMIP	57214	Leukemia, MESH:D015473	Arsenic|Ca	18.68	20
DACT1	51339	Leukemia, MESH:D015473	Arsenic Tr	18.68	261
GGH	8836	Leukemia, MESH:D015473	Arsenic Tr	18.68	156
GZMA	3001	Leukemia, MESH:D015473	Arsenic Tr	18.68	154
TIMELESS	8914	Leukemia, MESH:D015473	Arsenic Tr	18.68	263
TRIO	7204	Leukemia, MESH:D015473	arsenite|D	18.68	160
ACOX1	51	Leukemia, MESH:D015473	Arsenic Tr	18.67	261
ATP5F1B	506	Leukemia, MESH:D015470	Decitabine	18.67	21
HAT1	8520	Leukemia, MESH:D015473	Arsenic|Ar	18.67	265
LIPC	3990	Leukemia, MESH:D015470	Bezafibrat	18.67	15
MLLT11	10962	Leukemia, MESH:D015473	Arsenic Tr	18.67	262
POLR3G	10622	Leukemia, MESH:D015470	Calcitriol|D	18.67	9
PRKCQ	5588	Leukemia, MESH:D015473	Arsenic|ars	18.67	159
QSOX1	5768	Leukemia, MESH:D015473	Arsenic Tr	18.67	263
RASL11A	387496	Leukemia, MESH:D015470	Alitretinoi	18.67	10
SAA1	6288	Leukemia, MESH:D015470	Bortezomi	18.67	14
SCN1B	6324	Leukemia, MESH:D015470	Calcitriol|D	18.67	18
SLC12A6	9990	Leukemia, MESH:D015470	Cytarabine	18.67	82
SPRED1	161742	Leukemia, MESH:D015470	Doxorubic	18.67	17
SPRY1	10252	Leukemia, MESH:D015473	arsenite|D	18.67	158
SSX2IP	117178	Leukemia, MESH:D015470	Azacitidine	18.67	16
TINF2	26277	Leukemia, MESH:D015470	Benzene|D	18.67	35
TMEM176	55365	Leukemia, MESH:D015470	Calcitriol|F	18.67	10
TNIP1	10318	Leukemia, MESH:D015473	Arsenic|De	18.67	158
CITED2	10370	Leukemia, MESH:D015473	Arsenic|Ar	18.66	266
COL10A1	1300	Leukemia, MESH:D015470	Dexameth	18.66	12
EMX2	2018	Leukemia, MESH:D015470	Dexameth	18.66	9
EPPK1	83481	Leukemia, MESH:D015470	Calcitriol|D	18.66	13
GCNT1	2650	Leukemia, MESH:D015470	Calcitriol|D	18.66	11
GNAZ	2781	Leukemia, MESH:D015470	Benzene|D	18.66	23
JARID2	3720	Leukemia, MESH:D015470	Arsenic Tr	18.66	21

NBL1	4681	Leukemia, MESH:D015470	Calcitriol|D	18.66	16
NDUFA1	4694	Leukemia, MESH:D015470	Doxorubic	18.66	15
NIN	51199	Leukemia, MESH:D015470	Dexameth	18.66	13
PDGFD	80310	Leukemia, MESH:D015470	Dexameth	18.66	13
PLXND1	23129	Leukemia, MESH:D015470	Dexameth	18.66	15
PREX1	57580	Leukemia, MESH:D015470	Dexameth	18.66	14
PTPN21	11099	Leukemia, MESH:D015470	Arsenic Tr	18.66	23
RAB6A	5870	Leukemia, MESH:D015470	Dexameth	18.66	15
RBMS3	27303	Leukemia, MESH:D015470	Dexameth	18.66	15
SSPN	8082	Leukemia, MESH:D015470	Calcitriol|D	18.66	9
TBC1D1	23216	Leukemia, MESH:D015470	Calcitriol|D	18.66	9
YPEL3	83719	Leukemia, MESH:D015470	Dexameth	18.66	10
GRIN1	2902	Leukemia, MESH:D007948	2-(2-amin	18.65	3
COMT	1312	Leukemia, MESH:D015473	arsenite|Ca	18.64	158
NR1I3	9970	Leukemia, MESH:D015473	arsenite|Cl	18.64	158
PLA2G4A	5321	Leukemia, MESH:D007948	2-(2-amin	18.63	3
DEPDC1B	55789	Leukemia, MESH:D015473	Arsenic|Ca	18.62	156
MAPK8	5599	Leukemia, MESH:D007948	2-(2-amin	18.62	3
MIR23A	407010	Leukemia, MESH:D015473	Arsenic Tr	18.62	261
NAV2	89797	Leukemia, MESH:D015473	Arsenic|ars	18.62	160
PPFIBP1	8496	Leukemia, MESH:D015473	arsenite|Ca	18.62	154
TP53TG1	11257	Leukemia, MESH:D015470	Daunorub	18.62	38
ATP5C1	11949	Leukemia, MESH:D015470	Dexameth	18.61	22
CCDC15	80071	Leukemia, MESH:D015470	Calcitriol|C	18.61	74
CLMN	79789	Leukemia, MESH:D015473	Arsenic|Ca	18.61	157
CNGB1	1258	Leukemia, MESH:D015470	Calcitriol|D	18.61	13
CYP4X1	260293	Leukemia, MESH:D015470	Calcitriol|C	18.61	75
EDNRB	1910	Leukemia, MESH:D007948	2-(2-amin	18.61	2
HNRNPAE	3182	Leukemia, MESH:D015473	Dexameth	18.61	155
HOXB8	3218	Leukemia, MESH:D015470	Cytarabine	18.61	78
HPCAL1	3241	Leukemia, MESH:D015473	Arsenic|Ar	18.61	263
KIR2DS4	3809	Leukemia, MESH:D015470	Azacitidine	18.61	7
MRPL57	78988	Leukemia, MESH:D015470	Arsenic Tr	18.61	19
MYF5	4617	Leukemia, MESH:D015470	Azacitidine	18.61	11
NLRP1	22861	Leukemia, MESH:D015473	Arsenic Tr	18.61	157
PCED1B	91523	Leukemia, MESH:D015470	Cytarabine	18.61	78
POGZ	23126	Leukemia, MESH:D015470	Dexameth	18.61	15
TM2D3	80213	Leukemia, MESH:D015470	Bortezomi	18.61	14
VBP1	7411	Leukemia, MESH:D015470	Bezafibrat	18.61	13
ZZEF1	23140	Leukemia, MESH:D015470	Cyclophos	18.61	9
ADSS1	122622	Leukemia, MESH:D015470	Dexameth	18.6	12
APOBEC3I	200316	Leukemia, MESH:D015470	Azacitidine	18.6	21
ATP23	91419	Leukemia, MESH:D015470	Calcitriol|N	18.6	10
CCDC167	154467	Leukemia, MESH:D015470	Decitabine	18.6	14
CCDC88B	283234	Leukemia, MESH:D015473	Arsenic|Ge	18.6	157
CDH15	1013	Leukemia, MESH:D015470	Dexameth	18.6	7
CNPY4	245812	Leukemia, MESH:D015470	Dexameth	18.6	12
CPSF1	29894	Leukemia, MESH:D015473	arsenite|D	18.6	9
CXXC1	30827	Leukemia, MESH:D015470	Dexameth	18.6	13
CYB5R4	51167	Leukemia, MESH:D015470	Calcitriol|D	18.6	13
FAP	2191	Leukemia, MESH:D015470	Arsenic Tr	18.6	14
FOXN4	121643	Leukemia, MESH:D015470	Indometha	18.6	8
GAREM1	64762	Leukemia, MESH:D015470	Arsenic Tr	18.6	18
GBE1	2632	Leukemia, MESH:D015470	Deferoxan	18.6	16
GREB1L	80000	Leukemia, MESH:D015473	arsenite|G	18.6	154
H3C10	8357	Leukemia, MESH:D015470	Arsenic Tr	18.6	20
KCNQ4	9132	Leukemia, MESH:D015470	Cyclophos	18.6	8
LRRC32	2615	Leukemia, MESH:D015470	Cytarabine	18.6	80

LSM7	51690	Leukemia, MESH:D015470	Dexameth	18.6	12
MAMLD1	10046	Leukemia, MESH:D015470	Calcitriol|D	18.6	12
MIR143	406935	Leukemia, MESH:D015473	Arsenic|Ar	18.6	264
MIR20A	406982	Leukemia, MESH:D015473	Calcitriol|D	18.6	7
MMP2	4313	Leukemia, MESH:D007948	2-(2-amir	18.6	3
MORF4L1	10933	Leukemia, MESH:D015470	Dexameth	18.6	13
NDUFAF2	91942	Leukemia, MESH:D015470	Dexameth	18.6	13
PCID2	55795	Leukemia, MESH:D015470	Dronabinc	18.6	8
PPT2	9374	Leukemia, MESH:D015470	Cytarabine	18.6	79
PSMG4	389362	Leukemia, MESH:D015470	Gasoline|(	18.6	11
RBM3	5935	Leukemia, MESH:D015470	Dexameth	18.6	16
SENP5	205564	Leukemia, MESH:D015470	Doxorubic	18.6	15
SGCE	8910	Leukemia, MESH:D015470	Cyclophos	18.6	15
SLAIN2	57606	Leukemia, MESH:D015470	Dexameth	18.6	10
SNX27	81609	Leukemia, MESH:D015470	Dexameth	18.6	9
SYNE3	161176	Leukemia, MESH:D015473	Arsenic|De	18.6	156
VPS28	51160	Leukemia, MESH:D015470	Doxorubic	18.6	13
ZCCHC8	55596	Leukemia, MESH:D015470	Dronabinc	18.6	10
ACBD7	414149	Leukemia, MESH:D015473	Arsenic|Ca	18.59	156
APOL6	80830	Leukemia, MESH:D015473	arsenite|D	18.59	155
IFT172	26160	Leukemia, MESH:D015473	Arsenic Tr	18.59	262
L3MBTL1	26013	Leukemia, MESH:D015470	Dexameth	18.59	12
PACC1	55248	Leukemia, MESH:D015473	arsenite|D	18.59	154
ABTB2	25841	Leukemia, MESH:D015470	Doxorubic	18.58	17
ADRB1	153	Leukemia, MESH:D015473	arsenite|D	18.58	10
CDS1	1040	Leukemia, MESH:D015470	Arsenic Tr	18.58	21
DDX1	1653	Leukemia, MESH:D015470	Arsenic Tr	18.58	17
FLOT2	2319	Leukemia, MESH:D015470	Dexameth	18.58	16
GH1	2688	Leukemia, MESH:D015473	Arsenic Tr	18.58	262
HELZ2	85441	Leukemia, MESH:D015470	Decitabine	18.58	20
HINT1	3094	Leukemia, MESH:D015470	Benzene|D	18.58	31
IFRD2	7866	Leukemia, MESH:D015470	Bortezomi	18.58	13
KDM7A	80853	Leukemia, MESH:D015470	Arsenic Tr	18.58	22
KHDRBS1	10657	Leukemia, MESH:D015470	Irinotecan	18.58	12
KPNA7	402569	Leukemia, MESH:D015470	Indometha	18.58	6
LIMK1	3984	Leukemia, MESH:D015470	Benzene|D	18.58	29
LRP6	4040	Leukemia, MESH:D015470	(+)-JQ1 cc	18.58	12
NDUFB2	4708	Leukemia, MESH:D015470	Arsenic Tr	18.58	20
NEFH	4744	Leukemia, MESH:D015473	alpha-Toc	18.58	160
PCDH18	54510	Leukemia, MESH:D015470	Azacitidine	18.58	16
PIK3R5	23533	Leukemia, MESH:D015470	Air Polluta	18.58	22
PLOD3	8985	Leukemia, MESH:D015470	Cyclophos	18.58	14
PTBP3	9991	Leukemia, MESH:D015470	Arsenic Tr	18.58	20
SEPHS2	22928	Leukemia, MESH:D015470	Arsenic Tr	18.58	24
SUZ12	23512	Leukemia, MESH:D015470	Dexameth	18.58	13
TRPC3	7222	Leukemia, MESH:D015470	Arsenic Tr	18.58	20
TUBA8	51807	Leukemia, MESH:D015470	Arsenic Tr	18.58	19
CGNL1	84952	Leukemia, MESH:D015470	Arsenic Tr	18.57	19
CLIC3	9022	Leukemia, MESH:D015470	Arsenic Tr	18.57	14
DAD1	1603	Leukemia, MESH:D015470	Arsenic Tr	18.57	19
DSCC1	79075	Leukemia, MESH:D015470	Calcitriol|D	18.57	15
HNRNPC	3183	Leukemia, MESH:D015473	arsenic dis	18.57	155
KPNB1	3837	Leukemia, MESH:D015473	Buthionine	18.57	156
MIR4435-	541471	Leukemia, MESH:D015470	Doxorubic	18.57	12
PHF20L1	51105	Leukemia, MESH:D015470	Dexameth	18.57	9
RSL1D1	26156	Leukemia, MESH:D015470	Arsenic Tr	18.57	19
SLC6A15	55117	Leukemia, MESH:D015470	Dronabinc	18.57	9
TFF1	7031	Leukemia, MESH:D007948	2-(2-amir	18.57	3

TNRC18	84629	Leukemia, MESH:D015470	Doxorubic	18.57	15
CREBRF	153222	Leukemia, MESH:D015473	Arsenic|ar:	18.56	158
CRIP1	1396	Leukemia, MESH:D015473	Arsenic|Ar	18.56	159
DKK3	27122	Leukemia, MESH:D015473	Arsenic|Ar	18.56	263
EIF3A	8661	Leukemia, MESH:D015473	arsenite|D	18.56	158
RBBP4	5928	Leukemia, MESH:D015473	Arsenic Tr	18.56	265
TBL1XR1	79718	Leukemia, MESH:D015473	Arsenic|ar:	18.56	14
DLAT	1737	Leukemia, MESH:D015473	arsenite|D	18.55	157
CCR1	1230	Leukemia, MESH:D015470	Arsenic Tr	18.54	21
CSRNP1	64651	Leukemia, MESH:D015470	Calcitriol|[	18.54	16
CSRP2	1466	Leukemia, MESH:D015470	Arsenic Tr	18.54	20
EEF2	1938	Leukemia, MESH:D015473	Arsenic Tr	18.54	164
EPCAM	4072	Leukemia, MESH:D015473	Arsenic|Ar	18.54	264
GNAS	2778	Leukemia, MESH:D015473	arsenite|Cl	18.54	157
IER2	9592	Leukemia, MESH:D015470	Arsenic Tr	18.54	33
SLC2A4	6517	Leukemia, MESH:D015473	Arsenic|ar:	18.54	161
HACL1	26061	Leukemia, MESH:D015473	Arsenic Tr	18.52	260
CD163	9332	Leukemia, MESH:D015473	arsenite|Bı	18.51	10
HNF4A	3172	Leukemia, MESH:D015473	Calcitriol|(	18.51	156
KLHL14	57565	Leukemia, MESH:D015473	Arsenic Tr	18.51	260
MAPK11	5600	Leukemia, MESH:D015473	Arsenic Tr	18.51	155
MIR146B	574447	Leukemia, MESH:D015473	Arsenic Tr	18.51	261
NR1D1	9572	Leukemia, MESH:D015473	arsenite|D	18.51	155
OTPA	560759	Leukemia, MESH:D015470	Dronabinc	18.51	6
PRKAA1	5562	Leukemia, MESH:D015473	Arsenic Tr	18.51	158
VIT	5212	Leukemia, MESH:D015473	Arsenic Tr	18.51	153
ELAPOR1	57535	Leukemia, MESH:D015473	Calcitriol|(	18.5	155
ETV4	2118	Leukemia, MESH:D015473	Arsenic Tr	18.5	262
IL10RA	3587	Leukemia, MESH:D015473	Arsenic|De	18.5	156
INCENP	3619	Leukemia, MESH:D015473	Arsenic|ar:	18.5	160
KLHL41B	321064	Leukemia, MESH:D015470	Dexameth	18.5	6
LGALS9	3965	Leukemia, MESH:D015470	Allopurinc	18.5	16
MYBBP1A	10514	Leukemia, MESH:D015473	Arsenic Tr	18.5	261
NFATC2	4773	Leukemia, MESH:D015473	Arsenic|ar:	18.5	158
SNORA72	26775	Leukemia, MESH:D015470	Doxorubic	18.5	11
TLR1	7096	Leukemia, MESH:D015473	Arsenic|ar:	18.5	157
TPT1	7178	Leukemia, MESH:D015473	Arsenic Tr	18.5	263
ATL2	64225	Leukemia, MESH:D015470	Doxorubic	18.49	18
CASP8AP2	9994	Leukemia, MESH:D015470	Arsenic Tr	18.49	23
GLRX5	51218	Leukemia, MESH:D015473	arsenite|G	18.49	156
HDDC2	51020	Leukemia, MESH:D015473	Arsenic|ar:	18.49	157
HDGF	3068	Leukemia, MESH:D015470	Doxorubic	18.49	15
IRF4	3662	Leukemia, MESH:D015470	Arsenic Tr	18.49	17
LAT2	7462	Leukemia, MESH:D015470	Arsenic Tr	18.49	14
LTC4S	4056	Leukemia, MESH:D015470	Air Polluta	18.49	18
MED13L	23389	Leukemia, MESH:D015470	Cyclophos	18.49	16
NUP50	10762	Leukemia, MESH:D015470	Dexameth	18.49	15
PCOLCE2	26577	Leukemia, MESH:D015470	Arsenic Tr	18.49	15
PRPF4B	8899	Leukemia, MESH:D015470	Indometha	18.49	14
RHPN2	85415	Leukemia, MESH:D015470	Calcitriol|(	18.49	83
SLC26A2	1836	Leukemia, MESH:D015470	Dronabinc	18.49	13
SPTLC1	10558	Leukemia, MESH:D015473	Arsenic Tr	18.49	155
STARD10	10809	Leukemia, MESH:D015470	Doxorubic	18.49	14
TECR	9524	Leukemia, MESH:D015470	Air Polluta	18.49	34
ZCCHC7	84186	Leukemia, MESH:D015470	Dexameth	18.49	15
ACAT2	39	Leukemia, MESH:D015470	Arsenic Tr	18.48	23
NEFH	4744	Leukemia, MESH:D015470	Alitretinoi	18.48	28
RAB2B	84932	Leukemia, MESH:D015470	Arsenic Tr	18.48	34

TCP1	6950	Leukemia, MESH:D015470	Air Polluta	18.48	26
TUBB3	10381	Leukemia, MESH:D015473	2-(2-chlor	18.48	161
ANKRD13A	88455	Leukemia, MESH:D015470	Dexameth	18.47	8
ARHGEF5	7984	Leukemia, MESH:D015470	Gasoline|(	18.47	8
ARSI	340075	Leukemia, MESH:D015470	Calcitriol|D	18.47	8
CARF	79800	Leukemia, MESH:D015470	Indometha	18.47	9
CEP63	80254	Leukemia, MESH:D015470	Calcitriol|D	18.47	12
CRYBA4	1413	Leukemia, MESH:D015470	Dronabinc	18.47	8
CYP27A1	1593	Leukemia, MESH:D015473	Arsenic|Ca	18.47	21
EVA1C	59271	Leukemia, MESH:D015470	Dexameth	18.47	12
GPR153	387509	Leukemia, MESH:D015470	Calcitriol|E	18.47	8
KIF26A	26153	Leukemia, MESH:D015470	Calcitriol|D	18.47	13
L3HYPDH	112849	Leukemia, MESH:D015470	Calcitriol|D	18.47	8
MAF1	84232	Leukemia, MESH:D015470	Bortezomi	18.47	13
MCTP2	55784	Leukemia, MESH:D015470	Air Polluta	18.47	15
MED12	9968	Leukemia, MESH:D015470	Dexameth	18.47	13
MRPL48	51642	Leukemia, MESH:D015470	Dexameth	18.47	12
MYL6B	140465	Leukemia, MESH:D015470	Decitabine	18.47	15
NGRN	51335	Leukemia, MESH:D015470	Air Polluta	18.47	14
NKAIN4	128414	Leukemia, MESH:D015470	Dexameth	18.47	9
PAIP2	51247	Leukemia, MESH:D015470	Dexameth	18.47	8
PLD6	201164	Leukemia, MESH:D015470	(+)-JQ1 cc	18.47	8
PROM2	150696	Leukemia, MESH:D015470	Calcitriol|(	18.47	10
PUS7L	83448	Leukemia, MESH:D015470	Doxorubic	18.47	13
RBM28	55131	Leukemia, MESH:D015470	Dexameth	18.47	10
SCARF2	91179	Leukemia, MESH:D015470	Arsenic Tr	18.47	15
SMYD1	150572	Leukemia, MESH:D015470	Dexameth	18.47	12
SSH2	85464	Leukemia, MESH:D015470	Dexameth	18.47	15
TMEM243	79161	Leukemia, MESH:D015470	Arsenic Tr	18.47	18
TMEM40	55287	Leukemia, MESH:D015470	Benzene|C	18.47	23
TRMT12	55039	Leukemia, MESH:D015470	Dexameth	18.47	12
ZBTB8OS	339487	Leukemia, MESH:D015470	Dexameth	18.47	13
COX20	116228	Leukemia, MESH:D015470	Dexameth	18.46	13
DCLK2	166614	Leukemia, MESH:D015470	Dronabinc	18.46	8
DIRAS1	148252	Leukemia, MESH:D015470	Dexameth	18.46	12
HGH1	51236	Leukemia, MESH:D015470	Dexameth	18.46	12
IL5	3567	Leukemia, MESH:D015473	Dasatinib|I	18.46	155
NHS	4810	Leukemia, MESH:D015470	Calcitriol|D	18.46	13
NT5DC1	221294	Leukemia, MESH:D015470	Doxorubic	18.46	13
QTRT2	79691	Leukemia, MESH:D015470	Dexameth	18.46	13
SCML2	10389	Leukemia, MESH:D015470	Calcitriol|D	18.46	10
SETD3	84193	Leukemia, MESH:D015470	Dexameth	18.46	12
TMEM120	144404	Leukemia, MESH:D015470	Doxorubic	18.46	15
TPD52	7163	Leukemia, MESH:D015473	Arsenic Tr	18.46	264
TTLL1	25809	Leukemia, MESH:D015470	Doxorubic	18.46	13
ZBTB46	140685	Leukemia, MESH:D015470	Decitabine	18.46	19
CDH3	1001	Leukemia, MESH:D015473	Arsenic|ars	18.45	160
EIF2S2	8894	Leukemia, MESH:D015473	arsenite|Ca	18.45	155
AHSG	197	Leukemia, MESH:D015473	Dexameth	18.44	155
AQP7	364	Leukemia, MESH:D015473	arsenite|D	18.44	159
BIRC2	329	Leukemia, MESH:D007948	Arsenic Tr	18.44	3
CLCF1	23529	Leukemia, MESH:D015473	Arsenic|Ca	18.44	13
ELAVL1	1994	Leukemia, MESH:D015473	Arsenic Tr	18.44	261
ETFB	2109	Leukemia, MESH:D015473	Arsenic|Ar	18.44	161
TFAP2A	7020	Leukemia, MESH:D004915	Cytarabine	18.44	2
TSHB	7252	Leukemia, MESH:D015470	15-deoxy-	18.44	19
CYBA	1535	Leukemia, MESH:D007948	Arsenic Tr	18.43	4
COG3	83548	Leukemia, MESH:D015473	arsenic dis	18.42	7

DLD	1738	Leukemia, MESH:D015470		Dexameth	18.42	16
DMRT1	1761	Leukemia, MESH:D015473		caffeic aci	18.42	151
IFITM1	8519	Leukemia, MESH:D004915		Doxorubic	18.42	3
KRT10	3858	Leukemia, MESH:D015470		Alitretinoi	18.42	15
MIR10A	406902	Leukemia, MESH:D015473		Arsenic|ars	18.42	263
RTN4	57142	Leukemia, MESH:D015470		Arsenic Tr	18.42	36
SHMT2	6472	Leukemia, MESH:D015470		Dexameth	18.42	12
TNS1	7145	Leukemia, MESH:D015470		Dexameth	18.42	15
ATP6V0C	527	Leukemia, MESH:D015470		Azacitidine	18.41	16
EXT1	2131	Leukemia, MESH:D015470		Doxorubic	18.41	17
F12	2161	Leukemia, MESH:D015470		Arsenic Tr	18.41	24
LAP3	51056	Leukemia, MESH:D015470		Alitretinoi	18.41	22
LRBA	987	Leukemia, MESH:D015470		Cytarabine	18.41	83
MAPRE1	22919	Leukemia, MESH:D015470		Arsenic Tr	18.41	23
MEOX2	4223	Leukemia, MESH:D015470		Decitabine	18.41	20
MTDH	92140	Leukemia, MESH:D015470		Dronabinc	18.41	9
MYC	4609	Leukemia, MESH:D007948		2-(2-amin	18.41	3
ND6	4541	Leukemia, MESH:D015470		Deferoxan	18.41	16
PIP4K2A	5305	Leukemia, MESH:D015470		Air Polluta	18.41	13
PUS1	80324	Leukemia, MESH:D015470		Benzoates	18.41	10
PXMP2	5827	Leukemia, MESH:D015470		Arsenic Tr	18.41	15
ADAMTS1	9510	Leukemia, MESH:D015473		Arsenic|Ar	18.4	268
ATOH8	84913	Leukemia, MESH:D015470		Dexameth	18.4	15
ATP1A1	476	Leukemia, MESH:D015473		Calcitriol|E	18.4	155
DUSP4	1846	Leukemia, MESH:D004915		Cytarabine	18.4	2
FAR2	55711	Leukemia, MESH:D015473		arsenite|Ca	18.4	154
GLRX	2745	Leukemia, MESH:D015473		Antimony	18.4	159
GSTO2	119391	Leukemia, MESH:D015470		Arsenic Tr	18.4	21
GZMB	3002	Leukemia, MESH:D015473		Cholestero	18.4	154
KCNK5	8645	Leukemia, MESH:D015470		Calcitriol|D	18.4	9
MCMBP	79892	Leukemia, MESH:D015473		Calcitriol|D	18.4	154
MYO1D	4642	Leukemia, MESH:D015470		Calcitriol|D	18.4	9
N4BP2L2	10443	Leukemia, MESH:D015470		Dexameth	18.4	17
RAMP1	10267	Leukemia, MESH:D015473		Arsenic|ca	18.4	19
SASH3	54440	Leukemia, MESH:D015473		Calcitriol|D	18.4	154
SIAH2	6478	Leukemia, MESH:D015473		Arsenic|Bu	18.4	157
TAOK1	57551	Leukemia, MESH:D015470		Dexameth	18.4	9
TLE3	7090	Leukemia, MESH:D015470		Decitabine	18.4	21
TPBG	7162	Leukemia, MESH:D015470		Calcitriol|D	18.4	15
UBA5	79876	Leukemia, MESH:D015470		Arsenic Tr	18.4	19
AKT2	208	Leukemia, MESH:D015470		Arsenic Tr	18.39	25
CACNA1A	773	Leukemia, MESH:D015473		Arsenic Tr	18.39	262
MAPK13	5603	Leukemia, MESH:D015473		Arsenic Tr	18.39	155
MRPL16	54948	Leukemia, MESH:D015473		Arsenic|ars	18.39	13
MRPL22	29093	Leukemia, MESH:D015473		arsenite|D	18.39	9
MRPS12	6183	Leukemia, MESH:D015473		Arsenic Tr	18.39	262
PDE2A	5138	Leukemia, MESH:D015473		Arsenic Tr	18.39	262
PIK3CG	5294	Leukemia, MESH:D015473		Arsenic Tr	18.39	267
RMI1	80010	Leukemia, MESH:D015473		Calcitriol|D	18.39	156
BCAN	63827	Leukemia, MESH:D015473		arsenite|D	18.38	155
SLC41A3	54946	Leukemia, MESH:D015473		Arsenic Tr	18.38	261
C1ORF162	128346	Leukemia, MESH:D015470		Calcitriol|(	18.37	9
TSC2	7249	Leukemia, MESH:D01	marker/m	Arsenic Tr	18.36	41
CAMK2D	817	Leukemia, MESH:D015470		Arsenic Tr	18.36	24
GLUD1	2746	Leukemia, MESH:D015470		Arsenic Tr	18.36	15
NRCAM	4897	Leukemia, MESH:D015470		Decitabine	18.36	16
RPS27L	51065	Leukemia, MESH:D015470		Decitabine	18.36	31
SCAND2P	54581	Leukemia, MESH:D015473		Arsenic Tr	18.36	259

CRNN	49860	Leukemia, MESH:D015473	Arsenic|M	18.35	154
MIR23A	407010	Leukemia, MESH:D015470	Arsenic Tr	18.35	37
MIR708	1E+08	Leukemia, MESH:D015470	Air Polluta	18.35	14
TAGLN	6876	Leukemia, MESH:D007948	Arsenic Tr	18.35	4
ABTB1	80325	Leukemia, MESH:D015470	Calcitriol|D	18.34	13
ANKRD24	170961	Leukemia, MESH:D015470	Decitabine	18.34	14
BCL2	596	Leukemia, MESH:D007948	2-(2-amin	18.34	4
CA12	771	Leukemia, MESH:D015473	Arsenic|Ca	18.34	20
CAPN15	6650	Leukemia, MESH:D015470	Bortezomi	18.34	13
CDC23	8697	Leukemia, MESH:D015470	Arsenic Tr	18.34	18
CDIPT	10423	Leukemia, MESH:D015470	Dexameth	18.34	7
COQ5	84274	Leukemia, MESH:D015470	Dexameth	18.34	12
CPSF1	29894	Leukemia, MESH:D015470	Dexameth	18.34	12
DOLK	22845	Leukemia, MESH:D015470	Benzene|D	18.34	26
GAL3ST4	79690	Leukemia, MESH:D015470	Calcitriol|D	18.34	11
HNRNPAC	10949	Leukemia, MESH:D015470	Doxorubic	18.34	13
IFT172	26160	Leukemia, MESH:D015470	Arsenic Tr	18.34	19
MPHOSPH	10199	Leukemia, MESH:D015470	Dexameth	18.34	15
MRPL52	122704	Leukemia, MESH:D015470	Dexameth	18.34	8
NIPAL1	152519	Leukemia, MESH:D015470	Dronabinc	18.34	8
NPY2R	4887	Leukemia, MESH:D015470	Ethylnitros	18.34	10
NUDT3	11165	Leukemia, MESH:D015470	Dexameth	18.34	9
OXGR1	27199	Leukemia, MESH:D015470	(+)-JQ1 cc	18.34	9
PDIA3	2923	Leukemia, MESH:D015470	Arsenic Tr	18.34	31
PFN1	5216	Leukemia, MESH:D015473	arsenite|D	18.34	156
POLR2J2	246721	Leukemia, MESH:D015470	Arsenic Tr	18.34	13
RFXANK	8625	Leukemia, MESH:D015470	Doxorubic	18.34	12
RPE65	6121	Leukemia, MESH:D015473	Arsenic|ca	18.34	155
RPL3	6122	Leukemia, MESH:D015473	Arsenic|Ar	18.34	160
SNAPC5	10302	Leukemia, MESH:D015470	Arsenic Tr	18.34	14
SREK1IP1	285672	Leukemia, MESH:D015470	Calcitriol|C	18.34	8
TCF20	6942	Leukemia, MESH:D015470	Dexameth	18.34	9
TTC12	54970	Leukemia, MESH:D015470	Arsenic Tr	18.34	18
ZNF792	126375	Leukemia, MESH:D015470	Decitabine	18.34	18
AP1G2	8906	Leukemia, MESH:D015470	Dexameth	18.33	8
APOL6	80830	Leukemia, MESH:D015470	Dexameth	18.33	8
BBS7	55212	Leukemia, MESH:D015470	Dexameth	18.33	8
BEND3	57673	Leukemia, MESH:D015470	Dexameth	18.33	13
CEP55	55165	Leukemia, MESH:D015473	Arsenic Tr	18.33	264
CHST6	4166	Leukemia, MESH:D015470	Arsenic Tr	18.33	14
CXCL15	20309	Leukemia, MESH:D015470	Benzene|D	18.33	28
DCP1A	55802	Leukemia, MESH:D015470	Dexameth	18.33	12
ELMOD3	84173	Leukemia, MESH:D015470	Dexameth	18.33	13
EPHA3	2042	Leukemia, MESH:D015470	Doxorubic	18.33	15
GPHN	10243	Leukemia, MESH:D015470	Bortezomi	18.33	23
GREB1L	80000	Leukemia, MESH:D015470	Doxorubic	18.33	15
ICAM1	3383	Leukemia, MESH:D007948	2-(2-amin	18.33	3
LRRC49	54839	Leukemia, MESH:D015470	Doxorubic	18.33	13
MACC1	346389	Leukemia, MESH:D015470	Dexameth	18.33	8
MIR22	407004	Leukemia, MESH:D015470	Calcitriol|E	18.33	14
MZT2B	80097	Leukemia, MESH:D015470	Indometha	18.33	7
NFYA	4800	Leukemia, MESH:D015470	Benzene|D	18.33	25
PACC1	55248	Leukemia, MESH:D015470	Dexameth	18.33	15
PLK3	1263	Leukemia, MESH:D004915	Daunorub	18.33	2
RNF182	221687	Leukemia, MESH:D015470	Doxorubic	18.33	13
ROBO1	6091	Leukemia, MESH:D015473	Arsenic|Ar	18.33	263
SLCO4A1	28231	Leukemia, MESH:D015473	arsenite|D	18.33	157
SPATA2	9825	Leukemia, MESH:D015470	Dexameth	18.33	13

TXLNG	55787	Leukemia, MESH:D015470	Dexameth	18.33	13
ZNF324	25799	Leukemia, MESH:D015470	Dexameth	18.33	6
ZNRF3	84133	Leukemia, MESH:D015470	Arsenic Tr	18.33	14
ABI3BP	25890	Leukemia, MESH:D015470	Calcitriol|D	18.32	13
ARSB	411	Leukemia, MESH:D015470	Doxorubic	18.32	14
COBLL1	22837	Leukemia, MESH:D015470	Arsenic Tr	18.32	85
COX7B	1349	Leukemia, MESH:D015470	Arsenic Tr	18.32	20
DLX2	1746	Leukemia, MESH:D015470	Decitabine	18.32	15
GRIK2	2898	Leukemia, MESH:D015470	Benzoates	18.32	14
KRT16	3868	Leukemia, MESH:D015470	Calcitriol|D	18.32	9
KTN1	3895	Leukemia, MESH:D015470	Arsenic Tr	18.32	21
MAN2A1	4124	Leukemia, MESH:D015470	Dexameth	18.32	17
MPRIP	23164	Leukemia, MESH:D015470	Calcitriol|D	18.32	15
MTHFR	4524	Leukemia, MESH:D015473	Arsenic|Ca	18.32	159
PALMD	54873	Leukemia, MESH:D015470	Dexameth	18.32	13
PGM2L1	283209	Leukemia, MESH:D015470	Dexameth	18.32	11
SLC12A8	84561	Leukemia, MESH:D015470	Doxorubic	18.32	14
SP5	389058	Leukemia, MESH:D015470	Dexameth	18.32	9
THOP1	7064	Leukemia, MESH:D015470	Arsenic Tr	18.32	14
TMBIM1	64114	Leukemia, MESH:D015470	Arsenic Tr	18.32	19
CDKN1B	1027	Leukemia, MESH:D004915	Daunorub	18.31	3
ADGRE1	2015	Leukemia, MESH:D015470	Air Polluta	18.3	18
CKAP2	26586	Leukemia, MESH:D015470	Arsenic Tr	18.3	21
NCAM1	4684	Leukemia, MESH:D015470	Azacitidine	18.3	16
NRG1	3084	Leukemia, MESH:D007948	2-(2-amin	18.3	3
NUF2	83540	Leukemia, MESH:D015470	Calcitriol|D	18.3	22
UBD	10537	Leukemia, MESH:D015470	Cyclophos	18.3	13
CDC25C	995	Leukemia, MESH:D007948	2-(2-amin	18.29	3
CYP2E1	1571	Leukemia, MESH:D007948	2-(2-amin	18.29	3
EYA3	2140	Leukemia, MESH:D015473	Arsenic|De	18.29	12
FDXR	2232	Leukemia, MESH:D015473	Arsenic|Da	18.29	25
GSTM1	2944	Leukemia, MESH:D004915	Cytarabine	18.29	2
RPS6KA4	8986	Leukemia, MESH:D015473	Arsenic Tr	18.29	260
STC1	6781	Leukemia, MESH:D004915	Doxorubic	18.29	3
TFAM	7019	Leukemia, MESH:D015473	Arsenic|Ca	18.29	159
ABCB1	5243	Leukemia, MESH:D007948	Arsenic Tr	18.28	4
EIF3L	51386	Leukemia, MESH:D015473	arsenite|D	18.28	157
MAPT	4137	Leukemia, MESH:D015473	Arsenic|Ca	18.28	163
MTHFD1	4522	Leukemia, MESH:D015473	Arsenic Tr	18.28	261
NEDD8	4738	Leukemia, MESH:D015473	Arsenic Tr	18.28	262
NFKBIE	4794	Leukemia, MESH:D015473	Arsenic|Ar	18.28	263
RBPMS	11030	Leukemia, MESH:D015473	Antimony	18.28	263
BMPR2	659	Leukemia, MESH:D015473	Arsenic|Ar	18.27	266
LFNG	3955	Leukemia, MESH:D015473	Arsenic Tr	18.27	261
PDLIM7	9260	Leukemia, MESH:D015473	Arsenic Tr	18.27	260
RRBP1	6238	Leukemia, MESH:D015473	Arsenic Tr	18.27	263
EZR	7430	Leukemia, MESH:D015473	Arsenic|Ar	18.25	266
AKR1B1	231	Leukemia, MESH:D007948	Arsenic Tr	18.24	3
APBB2	323	Leukemia, MESH:D015470	Arsenic Tr	18.24	20
APLNR	187	Leukemia, MESH:D015470	Cytarabine	18.24	88
APOA2	336	Leukemia, MESH:D015470	Alitretinoi	18.24	10
ARHGAP2	58504	Leukemia, MESH:D015470	Dasatinib|I	18.24	9
ARHGEF7	8874	Leukemia, MESH:D015470	Doxorubic	18.24	17
ASCL1	429	Leukemia, MESH:D015470	Dronabinc	18.24	11
CYC1	1537	Leukemia, MESH:D015470	Air Polluta	18.24	21
CYP4A1	50549	Leukemia, MESH:D015470	Benzene|B	18.24	32
EEA1	8411	Leukemia, MESH:D015470	Air Polluta	18.24	13
FRMD6	122786	Leukemia, MESH:D015470	Doxorubic	18.24	16

MEX3B	84206	Leukemia, MESH:D015470	Doxorubic	18.24	16
MIR29A	407021	Leukemia, MESH:D015470	Arsenic Tr	18.24	22
PEMT	10400	Leukemia, MESH:D015470	Doxorubic	18.24	15
PROX1	5629	Leukemia, MESH:D015470	Decitabine	18.24	16
PSRC1	84722	Leukemia, MESH:D015473	arsenite|Ca	18.24	18
RIN2	54453	Leukemia, MESH:D015470	Calcitriol|D	18.24	14
RUVBL2	10856	Leukemia, MESH:D015470	Air Polluta	18.24	14
SCARA5	286133	Leukemia, MESH:D015470	Decitabine	18.24	21
SDF2L1	23753	Leukemia, MESH:D015470	Arsenic Tr	18.24	18
SEMA3E	9723	Leukemia, MESH:D015470	Doxorubic	18.24	14
SH2B3	10019	Leukemia, MESH:D015470	Arsenic Tr	18.24	19
SLC12A4	6560	Leukemia, MESH:D015470	Decitabine	18.24	24
SLC29A1	2030	Leukemia, MESH:D004915	Cytarabine	18.24	3
TJP2	9414	Leukemia, MESH:D015470	Arsenic Tr	18.24	21
CTNNAL1	8727	Leukemia, MESH:D015473	Arsenic|Ar	18.23	267
FOXQ1	94234	Leukemia, MESH:D015473	Antimony	18.23	155
GSC	145258	Leukemia, MESH:D015470	Antifungal	18.23	13
LEPR	3953	Leukemia, MESH:D015473	Arsenic|Ch	18.23	21
MIR146B	574447	Leukemia, MESH:D015470	Arsenic Tr	18.23	20
RPL10A	4736	Leukemia, MESH:D015473	Arsenic Tr	18.23	262
TRAP1	10131	Leukemia, MESH:D015473	Arsenic Tr	18.23	261
TSNAX	7257	Leukemia, MESH:D015470	Air Polluta	18.23	6
ANKRD37	353322	Leukemia, MESH:D015473	Arsenic|ars	18.22	13
BCL10	8915	Leukemia, MESH:D015473	Arsenic|Ar	18.22	158
CDA	978	Leukemia, MESH:D015473	arsenite|Ca	18.22	156
CRIP2	1397	Leukemia, MESH:D015473	arsenite|D	18.22	10
CXCL5	6374	Leukemia, MESH:D015473	Arsenic|Ar	18.22	161
CYP2E1	1571	Leukemia, MESH:D015473	alpha-Toc	18.22	265
FABP5	2171	Leukemia, MESH:D015473	Antimony	18.22	265
FSCN1	6624	Leukemia, MESH:D015473	Arsenic Tr	18.22	261
GPRC5A	9052	Leukemia, MESH:D015473	Arsenic Tr	18.22	261
MAP3K5	4217	Leukemia, MESH:D015473	Calcitriol|D	18.22	161
MCM5	4174	Leukemia, MESH:D015473	Arsenic|Ar	18.22	265
MED1	5469	Leukemia, MESH:D015473	Arsenic|Ar	18.22	264
MICA	1.01E+08	Leukemia, MESH:D015470	Arsenic Tr	18.22	27
MIPOL1	145282	Leukemia, MESH:D015470	Hydroxyur	18.22	9
OLFM1	10439	Leukemia, MESH:D015473	Arsenic|Ge	18.22	157
PPP1R10	5514	Leukemia, MESH:D015473	Arsenic|Ge	18.22	158
SLAMF1	6504	Leukemia, MESH:D015470	Air Polluta	18.22	10
ADCK2	90956	Leukemia, MESH:D015470	Decitabine	18.21	18
BMERB1	89927	Leukemia, MESH:D015470	Calcitriol|D	18.21	13
CLEC3B	7123	Leukemia, MESH:D015470	Calcitriol|D	18.21	12
DDIT3	1649	Leukemia, MESH:D007948	2-(2-amin	18.21	3
DGKE	8526	Leukemia, MESH:D015470	Bortezomi	18.21	9
GDF1	2657	Leukemia, MESH:D015470	Doxorubic	18.21	14
MAK16	84549	Leukemia, MESH:D015470	Dexameth	18.21	8
MAP3K7C	56911	Leukemia, MESH:D015470	Arsenic Tr	18.21	19
MCPH1	79648	Leukemia, MESH:D015470	Doxorubic	18.21	14
MRI1	84245	Leukemia, MESH:D015470	Decitabine	18.21	19
NCAPH2	29781	Leukemia, MESH:D015470	Dexameth	18.21	8
PKLR	5313	Leukemia, MESH:D015470	Alitretinoi	18.21	16
PPP2R5B	5526	Leukemia, MESH:D015470	Calcitriol|D	18.21	13
PRELID3B	51012	Leukemia, MESH:D015470	Dexameth	18.21	23
PRPF18	8559	Leukemia, MESH:D015470	Arsenic Tr	18.21	13
RNLS	55328	Leukemia, MESH:D015470	Arsenic Tr	18.21	14
TMEM71	137835	Leukemia, MESH:D015470	Doxorubic	18.21	15
TRIM9	114088	Leukemia, MESH:D015470	Doxorubic	18.21	13
UTP25	27042	Leukemia, MESH:D015470	Dexameth	18.21	13

ZDBF2	57683	Leukemia, MESH:D015470	Air Polluta	18.21	12
ZDHHC9	51114	Leukemia, MESH:D015470	Dexameth	18.21	9
NIPA1	123606	Leukemia, MESH:D015470	Doxorubic	18.2	13
PDE1B	5153	Leukemia, MESH:D015473	arsenite|C	18.2	156
PLCH1	23007	Leukemia, MESH:D015473	arsenite|C	18.2	157
ANGPT2	285	Leukemia, MESH:D015473	Arsenic Tr	18.19	263
GSPT2	23708	Leukemia, MESH:D015473	Arsenic Tr	18.19	262
HUS1	3364	Leukemia, MESH:D015473	Arsenic|Ar	18.19	161
QKI	9444	Leukemia, MESH:D015473	Arsenic|Ar	18.19	265
STOML2	30968	Leukemia, MESH:D015473	Arsenic Tr	18.19	263
ANKRD1	27063	Leukemia, MESH:D015470	Daunorub	18.18	42
B3GAT2	135152	Leukemia, MESH:D015473	Arsenic|De	18.18	155
HOXA9	3205	Leukemia, MESH:D015473	Arsenic Tr	18.18	260
MDH2	4191	Leukemia, MESH:D015473	Arsenic|ars	18.18	16
PPP1R12A	4659	Leukemia, MESH:D015470	Doxorubic	18.18	16
SLC40A1	30061	Leukemia, MESH:D015473	Arsenic Tr	18.18	264
TGFBR1	7046	Leukemia, MESH:D015473	Arsenic|ars	18.18	161
TIMP1	7076	Leukemia, MESH:D004915	Cytarabine	18.18	4
VPS52	6293	Leukemia, MESH:D015473	Arsenic|De	18.18	156
CDCA7	83879	Leukemia, MESH:D015473	Calcitriol|D	18.17	157
HSPA9	3313	Leukemia, MESH:D007948	Arsenic Tr	18.17	3
ORC1	4998	Leukemia, MESH:D015473	Calcitriol|D	18.17	156
PPIF	10105	Leukemia, MESH:D015473	Arsenic Tr	18.17	261
ATOX1	475	Leukemia, MESH:D015470	Clioquinol	18.16	14
CRYL1	51084	Leukemia, MESH:D015473	Arsenic|ars	18.16	157
CYB5A	1528	Leukemia, MESH:D015470	Androgen	18.16	83
IDE	3416	Leukemia, MESH:D015473	Arsenic|ars	18.16	160
MCCC2	64087	Leukemia, MESH:D015470	Dexameth	18.16	18
MTHFR	4524	Leukemia, MESH:D015470	Calcitriol|C	18.16	82
NUAK2	81788	Leukemia, MESH:D015470	Arsenic Tr	18.16	18
PSMD8	5714	Leukemia, MESH:D015470	Air Polluta	18.16	14
RAPH1	65059	Leukemia, MESH:D015470	Arsenic Tr	18.16	16
SEC23B	10483	Leukemia, MESH:D015470	Air Polluta	18.16	15
SEL1L3	23231	Leukemia, MESH:D015470	Air Polluta	18.16	19
STRBP	55342	Leukemia, MESH:D015470	Arsenic Tr	18.16	35
TSC22D1	8848	Leukemia, MESH:D015473	Arsenic Tr	18.15	158
ZNF552	79818	Leukemia, MESH:D015470	Arsenic Tr	18.14	18
ACPP	56318	Leukemia, MESH:D015470	Air Polluta	18.12	8
C17ORF80	55028	Leukemia, MESH:D015470	Air Polluta	18.12	14
C1R	715	Leukemia, MESH:D015473	Arsenic Tr	18.12	262
GADD45A	1647	Leukemia, MESH:D004915	Cytarabine	18.12	3
GSTM4	2948	Leukemia, MESH:D015470	Arsenic Tr	18.12	39
HMOX2	3163	Leukemia, MESH:D015473	Arsenic|Ar	18.12	263
PC	5091	Leukemia, MESH:D015470	Bezafibrat	18.12	18
SPART	23111	Leukemia, MESH:D015470	Doxorubic	18.12	15
TIA1	7072	Leukemia, MESH:D015473	arsenite|D	18.12	157
TRAF2	7186	Leukemia, MESH:D015473	Arsenic|Ar	18.12	264
ABHD2	11057	Leukemia, MESH:D015473	arsenite|D	18.11	155
CSRNP2	81566	Leukemia, MESH:D015473	Calcitriol|N	18.11	157
DES	1674	Leukemia, MESH:D004915	Daunorub	18.11	3
DNM3OS	1.01E+08	Leukemia, MESH:D015473	Arsenic Tr	18.11	261
DUSP8	1850	Leukemia, MESH:D015473	Arsenic|Ar	18.11	161
EGFLAM	133584	Leukemia, MESH:D015473	Arsenic|ars	18.11	158
ELOVL2	54898	Leukemia, MESH:D015473	Arsenic Tr	18.11	156
IL16	3603	Leukemia, MESH:D015473	Arsenic|Ca	18.11	156
IRS2	8660	Leukemia, MESH:D015473	Arsenic|De	18.11	158
OPTN	10133	Leukemia, MESH:D015473	Arsenic|Ar	18.11	265
SLC66A1L	152078	Leukemia, MESH:D015470	Dexameth	18.11	7

STEAP4	79689	Leukemia, MESH:D015473	Arsenic|Ca	18.11	158
XPA	7507	Leukemia, MESH:D015473	Arsenic|Ar	18.11	265
C1ORF159	54991	Leukemia, MESH:D015470	Dexameth	18.1	11
CYTH3	9265	Leukemia, MESH:D015473	arsenite|D	18.1	153
DOK5	55816	Leukemia, MESH:D015473	Arsenic Tr	18.1	156
ERH	2079	Leukemia, MESH:D015473	Arsenic Tr	18.1	156
FNDC10	643988	Leukemia, MESH:D015470	Doxorubic	18.1	14
ITK	3702	Leukemia, MESH:D015470	Air Polluta	18.1	9
SLITRK5	26050	Leukemia, MESH:D015473	arsenite|Cy	18.1	157
SPATA5	166378	Leukemia, MESH:D015473	arsenite|Ca	18.1	154
STMN4	81551	Leukemia, MESH:D015470	Cytarabine	18.1	83
TMEM200	645369	Leukemia, MESH:D015470	Dexameth	18.1	11
TRIM29	23650	Leukemia, MESH:D015473	Arsenic|Ca	18.1	156
TUSC1	286319	Leukemia, MESH:D015470	Dexameth	18.1	7
YIF1A	10897	Leukemia, MESH:D015473	Arsenic|Cy	18.1	21
ARL2BP	23568	Leukemia, MESH:D015470	Calcitriol|D	18.09	7
CARS2	79587	Leukemia, MESH:D015473	Arsenic|De	18.09	156
CCDC47	57003	Leukemia, MESH:D015470	Arsenic Tr	18.09	14
EDA	1896	Leukemia, MESH:D015470	Dexameth	18.09	13
FAR2	55711	Leukemia, MESH:D015470	Calcitriol|D	18.09	8
FBXO8	26269	Leukemia, MESH:D015470	Dexameth	18.09	8
FOXK1	221937	Leukemia, MESH:D015473	Arsenic|ars	18.09	157
HAND1	9421	Leukemia, MESH:D015470	Cytarabine	18.09	80
HPS6	79803	Leukemia, MESH:D015470	Benzene|D	18.09	20
HSP90B1	7184	Leukemia, MESH:D007948	Arsenic Tr	18.09	3
LETMD1	25875	Leukemia, MESH:D015470	Dexameth	18.09	12
MIR10A	406902	Leukemia, MESH:D015470	Air Polluta	18.09	15
MRPL22	29093	Leukemia, MESH:D015470	Dexameth	18.09	11
NSMCE4A	54780	Leukemia, MESH:D015470	Indometha	18.09	11
PGGHG	80162	Leukemia, MESH:D015470	Arsenic Tr	18.09	15
POLR3B	55703	Leukemia, MESH:D015470	Decitabine	18.09	16
POU4F1	5457	Leukemia, MESH:D015473	Arsenic|Ar	18.09	263
PPP1R13B	23368	Leukemia, MESH:D015473	Arsenic|Ar	18.09	265
RRAGA	10670	Leukemia, MESH:D015470	Cyclophos	18.09	14
SHE	126669	Leukemia, MESH:D015470	Calcitriol|D	18.09	14
SLC2A14	144195	Leukemia, MESH:D015473	arsenite|Ca	18.09	155
SLC49A4	84925	Leukemia, MESH:D015470	Benzene|D	18.09	27
SNX12	29934	Leukemia, MESH:D015470	Arsenic Tr	18.09	18
SP1	6667	Leukemia, MESH:D007948	Arsenic Tr	18.09	3
TAF5	6877	Leukemia, MESH:D015470	Benzene|M	18.09	27
TMEM256	254863	Leukemia, MESH:D015470	Doxorubic	18.09	13
UNC119	9094	Leukemia, MESH:D015470	Methotrex	18.09	12
UROS	7390	Leukemia, MESH:D015470	Gasoline|P	18.09	10
ZBTB5	9925	Leukemia, MESH:D015470	Air Polluta	18.09	13
ZNF207	7756	Leukemia, MESH:D015470	Doxorubic	18.09	16
ADH1A	124	Leukemia, MESH:D015470	Arsenic Tr	18.08	13
BMPER	168667	Leukemia, MESH:D015470	Decitabine	18.08	17
BTBD7	55727	Leukemia, MESH:D015470	Dexameth	18.08	8
CCDC127	133957	Leukemia, MESH:D015470	Dexameth	18.08	13
COX6A2	1339	Leukemia, MESH:D015470	Arsenic Tr	18.08	30
DDN	23109	Leukemia, MESH:D015470	Dexameth	18.08	7
DPEP1	1800	Leukemia, MESH:D015470	Arsenic Tr	18.08	20
EIF6	3692	Leukemia, MESH:D015470	Dexameth	18.08	10
GNG7	2788	Leukemia, MESH:D015470	Azacitidine	18.08	21
HIRA	7290	Leukemia, MESH:D015470	Dexameth	18.08	9
INO80D	54891	Leukemia, MESH:D015470	Doxorubic	18.08	13
KCNE4	23704	Leukemia, MESH:D015470	Doxorubic	18.08	14
KLHDC8A	55220	Leukemia, MESH:D015470	Arsenic Tr	18.08	13

KLHDC8B	200942	Leukemia, MESH:D015470		Decitabine	18.08	18
LEO1	123169	Leukemia, MESH:D015470		Doxorubic	18.08	13
MPPED2	744	Leukemia, MESH:D015470		Arsenic Tr	18.08	19
MTSS2	92154	Leukemia, MESH:D015470		Dexameth	18.08	12
NDUFA2	4695	Leukemia, MESH:D015470		Dexameth	18.08	14
PTPRN	5798	Leukemia, MESH:D015470		Calcitriol|D	18.08	14
PTPRZ1	5803	Leukemia, MESH:D015470		Calcitriol|C	18.08	77
RHOB	388	Leukemia, MESH:D015473		Arsenic|Ar	18.08	265
SLC2A10	81031	Leukemia, MESH:D015470		Doxorubic	18.08	13
SNRPB2	6629	Leukemia, MESH:D015470		Dexameth	18.08	10
SYT13	57586	Leukemia, MESH:D015470		Decitabine	18.08	14
TSN	7247	Leukemia, MESH:D015470		Dexameth	18.08	10
UBIAD1	29914	Leukemia, MESH:D015470		Calcitriol|D	18.08	8
UBLCP1	134510	Leukemia, MESH:D015470		Dexameth	18.08	12
VASH2	79805	Leukemia, MESH:D015470		Dexameth	18.08	7
VWA1	64856	Leukemia, MESH:D015470		Calcitriol|D	18.08	12
WDR90	197335	Leukemia, MESH:D015470		Dexameth	18.08	15
ZNF644	84146	Leukemia, MESH:D015470		Dexameth	18.08	8
AQP9	366	Leukemia, MESH:D01	therapeuti	Arsenic Tr	18.07	20
IL6	3569	Leukemia, MESH:D004915		Cytarabine	18.07	4
ITPR2	3709	Leukemia, MESH:D015473		Arsenic|Ar	18.07	267
LMAN1	3998	Leukemia, MESH:D015470		Dexameth	18.07	14
SACS	26278	Leukemia, MESH:D015470		Arsenic Tr	18.07	16
SPSB1	80176	Leukemia, MESH:D015473		Arsenic|ars	18.07	157
ADD3	120	Leukemia, MESH:D015473		arsenite|Bu	18.06	158
BDKRB1	623	Leukemia, MESH:D015473		arsenite|Ca	18.06	156
ECI1	1632	Leukemia, MESH:D015470		Arsenic Tr	18.06	22
ERCC6	2074	Leukemia, MESH:D015473		Arsenic|Ar	18.06	161
H2AX	3014	Leukemia, MESH:D004915		Cytarabine	18.06	2
KL	9365	Leukemia, MESH:D015473		Arsenic Tr	18.06	156
NOCT	25819	Leukemia, MESH:D015470		Air Polluta	18.06	17
SELL	6402	Leukemia, MESH:D015470		Decitabine	18.06	21
TNFSF11	8600	Leukemia, MESH:D007948		2-(2-amin	18.06	3
CASP7	840	Leukemia, MESH:D004915		Doxorubic	18.05	3
DHCR24	1718	Leukemia, MESH:D015473		Calcitriol|C	18.05	157
HBEGF	1839	Leukemia, MESH:D007948		2-(2-amin	18.05	2
IFRD1	3475	Leukemia, MESH:D015473		Antimony	18.05	163
GPX1	2876	Leukemia, MESH:D004915		Daunorub	18.04	3
CHEK2	11200	Leukemia, MESH:D007948		2-(2-amin	18.03	3
PRL	5617	Leukemia, MESH:D015470		Azacitidine	18.03	90
SELE	6401	Leukemia, MESH:D015470		Bezafibrat	18.03	24
HAMP	57817	Leukemia, MESH:D015473		caffeic aci	18.02	10
MSR1	4481	Leukemia, MESH:D015473		Arsenic Tr	18.02	159
PGM1	5236	Leukemia, MESH:D015473		Antimony	18.02	261
ALOX15	246	Leukemia, MESH:D015470		Benzene|(	18.01	29
BASP1	10409	Leukemia, MESH:D015473		Arsenic Tr	18.01	261
FUS	2521	Leukemia, MESH:D015470		Calcitriol|D	18.01	13
MED14	9282	Leukemia, MESH:D015473		Arsenic|ars	18.01	156
NCAPD2	9918	Leukemia, MESH:D015473		arsenite|Ca	18.01	155
NR5A2	2494	Leukemia, MESH:D015470		Alitretinoi	18.01	12
PPM1D	8493	Leukemia, MESH:D015473		Arsenic|Ar	18.01	262
PSMB10	5699	Leukemia, MESH:D015473		arsenite|Ca	18.01	157
RPS19	6223	Leukemia, MESH:D015473		arsenite|Ca	18.01	9
SMC2	10592	Leukemia, MESH:D015473		Calcitriol|D	18.01	155
WNK2	65268	Leukemia, MESH:D015473		Arsenic Tr	18.01	155
ARHGAP2	9411	Leukemia, MESH:D015470		Decitabine	18	20
ARL5B	221079	Leukemia, MESH:D015470		Air Polluta	18	23
CD2	914	Leukemia, MESH:D015473		Arsenic|De	18	10

CYCS	54205	Leukemia, MESH:D004915	Daunorub	18	3
EPHA8	2046	Leukemia, MESH:D015473	Arsenic|ars	18	156
HGD	3081	Leukemia, MESH:D015470	Azacitidine	18	23
HSPA14	51182	Leukemia, MESH:D015473	arsenite|D	18	155
KIF18B	146909	Leukemia, MESH:D015470	Calcitriol|D	18	11
KIF7	374654	Leukemia, MESH:D015473	Arsenic|Ca	18	12
KRT14	3861	Leukemia, MESH:D015473	Arsenic Tr	18	262
MYO1B	4430	Leukemia, MESH:D015470	Arsenic Tr	18	21
OSBPL10	114884	Leukemia, MESH:D015473	arsenite|Cy	18	157
PRMT5	10419	Leukemia, MESH:D015470	Air Polluta	18	19
PSME3	10197	Leukemia, MESH:D015470	Arsenic Tr	18	17
PSPC1	55269	Leukemia, MESH:D015470	Arsenic Tr	18	23
RPL22L1	200916	Leukemia, MESH:D015470	Dexameth	18	8
SCP2	6342	Leukemia, MESH:D015470	Dexameth	18	15
SH3D21	79729	Leukemia, MESH:D015473	Arsenic|ars	18	157
STIM1	6786	Leukemia, MESH:D015470	Arsenic Tr	18	20
TFAP4	7023	Leukemia, MESH:D015473	Calcitriol|D	18	156
BOLA3	388962	Leukemia, MESH:D015473	arsenite|D	17.99	155
FOXO3	2309	Leukemia, MESH:D007948	2-(2-amin	17.99	3
IL12A	3592	Leukemia, MESH:D007948	2-(2-amin	17.99	2
MSI2	124540	Leukemia, MESH:D015470	Doxorubic	17.99	16
NFE2L2	4780	Leukemia, MESH:D004915	butenolide	17.99	5
NTN1	9423	Leukemia, MESH:D015470	Dexameth	17.99	14
WDR19	57728	Leukemia, MESH:D015473	Arsenic|Ar	17.99	263
BARD1	580	Leukemia, MESH:D015473	Calcitriol|D	17.98	159
CIP2A	57650	Leukemia, MESH:D015470	Calcitriol|C	17.98	9
H1F8	171506	Leukemia, MESH:D015470	Decitabine	17.98	16
SPIB	6689	Leukemia, MESH:D015470	Arsenic Tr	17.98	14
ALDH3B2	222	Leukemia, MESH:D015470	Benzene|C	17.97	23
CHKB	1120	Leukemia, MESH:D015470	Benzene|D	17.97	23
COL18A1	80781	Leukemia, MESH:D004915	Cytarabine	17.97	3
CYP27B1	1594	Leukemia, MESH:D015473	Arsenic|Ca	17.97	11
DDX27	55661	Leukemia, MESH:D015470	Doxorubic	17.97	16
EIF3L	51386	Leukemia, MESH:D015470	Benzene|D	17.97	24
FBXO33	254170	Leukemia, MESH:D015470	Dronabino	17.97	10
FMO2	2327	Leukemia, MESH:D015473	Arsenic|ca	17.97	11
GET1	7485	Leukemia, MESH:D015470	Air Polluta	17.97	19
KLK10	5655	Leukemia, MESH:D015470	Alitretinoi	17.97	8
KRT6B	3854	Leukemia, MESH:D015470	Calcitriol|C	17.97	8
MDGA1	266727	Leukemia, MESH:D015470	Dasatinib|I	17.97	12
MRPL34	64981	Leukemia, MESH:D015470	Dexameth	17.97	9
NCEH1	57552	Leukemia, MESH:D015473	Arsenic Tr	17.97	261
NSUN5	55695	Leukemia, MESH:D015470	Dexameth	17.97	14
PI4K2A	55361	Leukemia, MESH:D015470	Arsenic Tr	17.97	27
PYGO2	90780	Leukemia, MESH:D015470	Decitabine	17.97	15
RPAIN	84268	Leukemia, MESH:D015470	(+)-JQ1 co	17.97	11
SNAPC3	6619	Leukemia, MESH:D015470	Air Polluta	17.97	15
ACER3	55331	Leukemia, MESH:D015470	Doxorubic	17.96	13
ARHGAP3	257106	Leukemia, MESH:D015470	Calcitriol|D	17.96	8
CALHM2	51063	Leukemia, MESH:D015470	Air Polluta	17.96	13
CEBPG	1054	Leukemia, MESH:D015473	Arsenic|ars	17.96	163
CEP135	9662	Leukemia, MESH:D015470	Dexameth	17.96	11
CRTAC1	55118	Leukemia, MESH:D015470	Doxorubic	17.96	13
DZIP3	9666	Leukemia, MESH:D015470	Dexameth	17.96	8
FYTTD1	84248	Leukemia, MESH:D015470	Dexameth	17.96	9
FZD2	2535	Leukemia, MESH:D015473	Dexameth	17.96	157
HMBOX1	79618	Leukemia, MESH:D015470	Dexameth	17.96	12
KAT7	11143	Leukemia, MESH:D015470	Doxorubic	17.96	14

NEK1	4750	Leukemia, MESH:D015470	Arsenic Tr	17.96	18
NINL	22981	Leukemia, MESH:D015470	Dexameth	17.96	11
PLAGL1	5325	Leukemia, MESH:D015473	Arsenic|De	17.96	157
PLAUR	5329	Leukemia, MESH:D007948	2-(2-amin	17.96	3
PLEKHA4	57664	Leukemia, MESH:D015470	Doxorubic	17.96	13
POFUT2	23275	Leukemia, MESH:D015470	Dexameth	17.96	13
RBFOX1	54715	Leukemia, MESH:D015470	Dexameth	17.96	13
RBM43	375287	Leukemia, MESH:D015470	Doxorubic	17.96	13
RPL7L1	285855	Leukemia, MESH:D015470	Gasoline|I	17.96	8
SMYD5	10322	Leukemia, MESH:D015470	Dexameth	17.96	7
SPTBN4	57731	Leukemia, MESH:D015470	Arsenic Tr	17.96	14
TTLL5	23093	Leukemia, MESH:D015470	Dexameth	17.96	13
XRCC1	7515	Leukemia, MESH:D004915	Cytarabine	17.96	2
ZNHIT6	54680	Leukemia, MESH:D015470	Dexameth	17.96	13
DRL	30167	Leukemia, MESH:D015470	Pentachlo	17.95	6
HPSE	10855	Leukemia, MESH:D015473	Arsenic|De	17.95	156
HSPA7	3311	Leukemia, MESH:D015470	Bortezomi	17.95	7
LINC0065	29075	Leukemia, MESH:D015470	Arsenic Tr	17.95	11
MAP3K6	9064	Leukemia, MESH:D015473	Arsenic|De	17.95	156
NTS	4922	Leukemia, MESH:D015470	Arsenic Tr	17.95	20
PDYN	5173	Leukemia, MESH:D015470	Decitabine	17.95	22
SDHB	6390	Leukemia, MESH:D015473	Arsenic|Ar	17.95	264
SYNE1	23345	Leukemia, MESH:D015473	Arsenic|ars	17.95	160
DHRS4L2	317749	Leukemia, MESH:D015470	Dexameth	17.94	7
EPHX5	322331	Leukemia, MESH:D015470	Dexameth	17.94	5
MGLL	11343	Leukemia, MESH:D015473	arsenite|Ca	17.94	158
NFIL3	4783	Leukemia, MESH:D015473	Arsenic|ars	17.94	158
PER2	8864	Leukemia, MESH:D015473	Arsenic|Ar	17.94	262
THRSP	7069	Leukemia, MESH:D015473	Arsenic|Bu	17.94	13
FASL	14103	Leukemia, MESH:D015473	Arsenic Tr	17.93	155
PDX1	3651	Leukemia, MESH:D015470	Alitretinoi	17.93	27
C3AR1	719	Leukemia, MESH:D015470	Azacitidin	17.92	78
CLTB	1212	Leukemia, MESH:D015470	Arsenic Tr	17.92	21
CORO2A	7464	Leukemia, MESH:D015470	Arsenic Tr	17.92	16
DLL3	10683	Leukemia, MESH:D015473	Arsenic|Ge	17.92	155
FLRT2	23768	Leukemia, MESH:D015470	Cytarabine	17.92	78
GOLGA8A	23015	Leukemia, MESH:D015473	Dexameth	17.92	157
HLA-DRB	3126	Leukemia, MESH:D015470	Doxorubic	17.92	14
HMGN2	3151	Leukemia, MESH:D015470	Arsenic Tr	17.92	28
HYLS1	219844	Leukemia, MESH:D015473	arsenite|D	17.92	156
KCTD15	79047	Leukemia, MESH:D015470	Doxorubic	17.92	14
MPP1	4354	Leukemia, MESH:D015470	Doxorubic	17.92	16
MXD4	10608	Leukemia, MESH:D015470	Dexameth	17.92	9
NXF1	10482	Leukemia, MESH:D015470	Dexameth	17.92	9
PATJ	10207	Leukemia, MESH:D015470	Benzene|C	17.92	23
PAX3	5077	Leukemia, MESH:D015470	Dexameth	17.92	11
PPP1R9A	55607	Leukemia, MESH:D015470	Arsenic Tr	17.92	20
PSMD11	5717	Leukemia, MESH:D015470	Arsenic Tr	17.92	28
SGMS2	166929	Leukemia, MESH:D015470	Dexameth	17.92	9
TET1	80312	Leukemia, MESH:D015470	Arsenic Tr	17.92	21
TMC4	147798	Leukemia, MESH:D015473	Calcitriol|C	17.92	7
ZNF702P	79986	Leukemia, MESH:D015470	Cytarabine	17.92	82
AP3S1	1176	Leukemia, MESH:D015473	Arsenic Tr	17.91	260
AZU1	566	Leukemia, MESH:D015473	Arsenic Tr	17.91	264
BCS1L	617	Leukemia, MESH:D015473	arsenite|M	17.91	156
CCL7	6354	Leukemia, MESH:D015473	Arsenic|Ar	17.91	264
CKB	1152	Leukemia, MESH:D015473	Arsenic Tr	17.91	265
CTNNA1	1495	Leukemia, MESH:D015473	Arsenic|Ar	17.91	263

EIF3M	10480	Leukemia, MESH:D015473		Arsenic Tr	17.91	263
ITGA1	3672	Leukemia, MESH:D015473		Genistein|I	17.91	155
PABPC1L	80336	Leukemia, MESH:D015473		Arsenic|De	17.91	156
PKP3	11187	Leukemia, MESH:D015473		Arsenic Tr	17.91	157
SPATA18	132671	Leukemia, MESH:D015473		Arsenic|De	17.91	155
TXNL1	9352	Leukemia, MESH:D015473		arsenite|ca	17.91	11
H3-4	8290	Leukemia, MESH:D015473		Arsenic|Ar	17.9	263
NDUFB1	4707	Leukemia, MESH:D015473		arsenite|D	17.9	155
PNPLA3	80339	Leukemia, MESH:D015473		arsenite|D	17.9	155
SPATC1L	84221	Leukemia, MESH:D015470		Doxorubic	17.9	11
TNFRSF6B	8771	Leukemia, MESH:D015470		Arsenic Tr	17.9	13
WDR5	11091	Leukemia, MESH:D015473		Arsenic|ars	17.9	159
ZNF608	57507	Leukemia, MESH:D015473		Arsenic|ars	17.9	157
CDKN1A	1026	Leukemia, MESH:D004915		Cytarabine	17.89	3
DCXR	51181	Leukemia, MESH:D015470		Decitabine	17.89	22
GATA6	2627	Leukemia, MESH:D015470		Alitretinoi	17.89	29
NDRG2	57447	Leukemia, MESH:D015470		Cytarabine	17.89	82
NOTCH2N	388677	Leukemia, MESH:D015470		Dexameth	17.89	11
ZNF263	10127	Leukemia, MESH:D015470		Dexameth	17.89	8
AP5B1	91056	Leukemia, MESH:D015470		Dexameth	17.88	7
CCNG2	901	Leukemia, MESH:D015473		Arsenic Tr	17.88	166
ERFE	151176	Leukemia, MESH:D015470		Doxorubic	17.88	12
OGT	8473	Leukemia, MESH:D015473		arsenic dis	17.87	266
ZFP36	7538	Leukemia, MESH:D015473		Arsenic Tr	17.87	264
CEBPE	1053	Leukemia, MESH:D01	therapeuti	Arsenic Tr	17.86	262
ALAS2	212	Leukemia, MESH:D015473		Arsenic Tr	17.86	155
LY75	4065	Leukemia, MESH:D015470		Cytarabine	17.86	79
POLA1	5422	Leukemia, MESH:D015473		arsenite|D	17.86	156
SCN2B	6327	Leukemia, MESH:D015470		Daunorub	17.86	44
SERPINB9	5272	Leukemia, MESH:D015473		Calcitriol|C	17.86	156
ABLIM1	3983	Leukemia, MESH:D015473		Arsenic Tr	17.85	264
B4GALT7	11285	Leukemia, MESH:D015470		Cytarabine	17.85	79
CD300A	11314	Leukemia, MESH:D015470		Calcitriol|(	17.85	8
COQ9	57017	Leukemia, MESH:D015470		Dexameth	17.85	12
FGB	2244	Leukemia, MESH:D015470		Alitretinoi	17.85	11
FIP1L1	81608	Leukemia, MESH:D015470		Dasatinib|I	17.85	15
GLI3	2737	Leukemia, MESH:D015470		Androgen	17.85	15
GPR132	29933	Leukemia, MESH:D015470		Air Polluta	17.85	24
GPR87	53836	Leukemia, MESH:D015470		Arsenic Tr	17.85	13
IL10	3586	Leukemia, MESH:D007948		2-(2-amin	17.85	3
LMBR1	64327	Leukemia, MESH:D015470		Dexameth	17.85	12
LRRC58	116064	Leukemia, MESH:D015470		Calcitriol|D	17.85	14
MTBP	27085	Leukemia, MESH:D015470		Calcitriol|D	17.85	8
MYO19	80179	Leukemia, MESH:D015470		Calcitriol|D	17.85	7
NUDT16	131870	Leukemia, MESH:D015470		Dexameth	17.85	17
PIP4K2C	79837	Leukemia, MESH:D015470		Arsenic Tr	17.85	16
PLCH1	23007	Leukemia, MESH:D015470		Calcitriol|C	17.85	76
POLR2L	5441	Leukemia, MESH:D015470		Decitabine	17.85	19
PRDM8	56978	Leukemia, MESH:D015470		Arsenic Tr	17.85	84
PSAP	5660	Leukemia, MESH:D015473		arsenite|Ca	17.85	155
RBM6	10180	Leukemia, MESH:D015470		Air Polluta	17.85	11
RNF38	152006	Leukemia, MESH:D015470		Doxorubic	17.85	12
SCLT1	132320	Leukemia, MESH:D015470		Calcitriol|I	17.85	10
SLC26A3	1811	Leukemia, MESH:D015470		Calcitriol|D	17.85	18
SLC34A2	10568	Leukemia, MESH:D015473		Arsenic|Ca	17.85	156
SUN1	23353	Leukemia, MESH:D015470		Cyclophos	17.85	15
USP28	57646	Leukemia, MESH:D015470		Air Polluta	17.85	7
PICALM	8301	Leukemia, MESH:D01	marker/m	Dexameth	17.84	15

ACTN4	81	Leukemia, MESH:D015470	Doxorubic	17.84	14
AGPAT3	56894	Leukemia, MESH:D015470	Dexameth	17.84	17
ATP6	4508	Leukemia, MESH:D015470	Arsenic Tr	17.84	20
B3GAT2	135152	Leukemia, MESH:D015470	Dexameth	17.84	10
BZW1	9689	Leukemia, MESH:D015470	Arsenic Tr	17.84	21
C1RL	51279	Leukemia, MESH:D015470	Decitabine	17.84	14
C9ORF72	203228	Leukemia, MESH:D015470	Calcitriol|D	17.84	14
CCPG1	9236	Leukemia, MESH:D015470	Arsenic Tr	17.84	14
CCR5	1234	Leukemia, MESH:D015470	Benzene|C	17.84	25
CD53	963	Leukemia, MESH:D015470	Arsenic Tr	17.84	31
COL16A1	1307	Leukemia, MESH:D015470	Arsenic Tr	17.84	14
DNAJA4	55466	Leukemia, MESH:D015470	Arsenic Tr	17.84	27
ENPP3	5169	Leukemia, MESH:D015470	Dasatinib|	17.84	9
FAM120A	23196	Leukemia, MESH:D015470	Dexameth	17.84	12
IFT81	28981	Leukemia, MESH:D015470	Dexameth	17.84	14
IQCG	84223	Leukemia, MESH:D015470	Dexameth	17.84	12
MARCHF5	54708	Leukemia, MESH:D015470	Dexameth	17.84	10
MSN	4478	Leukemia, MESH:D015470	Arsenic Tr	17.84	23
MTA1	9112	Leukemia, MESH:D015470	Arsenic Tr	17.84	16
MYBPH	4608	Leukemia, MESH:D015470	Arsenic Tr	17.84	15
OGFOD1	55239	Leukemia, MESH:D015470	Arsenic Tr	17.84	20
PALB2	79728	Leukemia, MESH:D015470	Dexameth	17.84	21
PARD3	56288	Leukemia, MESH:D015470	Cyclophos	17.84	15
POU2F1	5451	Leukemia, MESH:D015470	Arsenic Tr	17.84	17
PROCA1	147011	Leukemia, MESH:D015470	Dexameth	17.84	8
PRRT2	112476	Leukemia, MESH:D015470	Dronabino	17.84	8
PSD3	23362	Leukemia, MESH:D015470	Arsenic Tr	17.84	83
PUM2	23369	Leukemia, MESH:D015470	Dexameth	17.84	13
QKI	9444	Leukemia, MESH:D015470	Arsenic Tr	17.84	19
RASGRP3	25780	Leukemia, MESH:D015470	Dexameth	17.84	17
RCN1	5954	Leukemia, MESH:D015470	Azacitidine	17.84	15
RPS27A	6233	Leukemia, MESH:D015470	Arsenic Tr	17.84	28
SLC17A7	57030	Leukemia, MESH:D015470	Dexameth	17.84	9
SMYD4	114826	Leukemia, MESH:D015470	Dexameth	17.84	13
SNIP1	79753	Leukemia, MESH:D015470	Dexameth	17.84	12
STAP2	55620	Leukemia, MESH:D015470	Calcitriol|D	17.84	12
SULT1A2	6799	Leukemia, MESH:D015470	Arsenic Tr	17.84	19
TIAM1	7074	Leukemia, MESH:D015470	Benzoates	17.84	13
UBE2V1	7335	Leukemia, MESH:D015470	Dexameth	17.84	9
WWC3	55841	Leukemia, MESH:D015470	Dexameth	17.84	13
BNC1	646	Leukemia, MESH:D015473	arsenite|Cy	17.83	156
ARFGEF3	57221	Leukemia, MESH:D015473	Arsenic|Ca	17.82	156
CEP350	9857	Leukemia, MESH:D015473	Arsenic|ars	17.82	11
ILDR2	387597	Leukemia, MESH:D015473	Methotrex	17.82	155
MYT1	4661	Leukemia, MESH:D015473	arsenite|D	17.82	154
NUTF2	10204	Leukemia, MESH:D015473	Arsenic|De	17.82	159
TMX4	56255	Leukemia, MESH:D015473	arsenite|Re	17.82	157
TYMS	7298	Leukemia, MESH:D007948	Arsenic Tr	17.82	4
ABI2	10152	Leukemia, MESH:D015473	Arsenic|Ar	17.81	263
APEX1	328	Leukemia, MESH:D015473	Arsenic|ars	17.81	27
NUDT6	11162	Leukemia, MESH:D015473	Arsenic|De	17.81	156
PDGFA	5154	Leukemia, MESH:D015473	Arsenic Tr	17.81	264
PGAM5	192111	Leukemia, MESH:D015473	Arsenic Tr	17.81	260
POU4F2	5458	Leukemia, MESH:D015473	arsenite|D	17.81	155
RPS2	6187	Leukemia, MESH:D015473	arsenite|D	17.81	158
TRIM2	23321	Leukemia, MESH:D015473	Calcitriol|D	17.81	155
ZBTB7A	51341	Leukemia, MESH:D015473	Arsenic|De	17.81	156
ACTA1	58	Leukemia, MESH:D004915	Cytarabine	17.8	2

TLR2	7097	Leukemia, MESH:D007948		2-(2-amir	17.8	3
GABRB2	2561	Leukemia, MESH:D015470		Dexameth	17.78	15
ROCK2	9475	Leukemia, MESH:D015470		Dexameth	17.78	11
VEGFC	7424	Leukemia, MESH:D004915		Cytarabine	17.78	2
CDC25A	993	Leukemia, MESH:D004915		Daunorub	17.77	2
HAMP	57817	Leukemia, MESH:D015470		Deferoxan	17.77	27
MMP3	4314	Leukemia, MESH:D007948		2-(2-amir	17.77	2
MYCBP2	23077	Leukemia, MESH:D015470		Arsenic Tr	17.77	24
PRKAB2	5565	Leukemia, MESH:D015470		Doxorubic	17.77	16
SFTPB	6439	Leukemia, MESH:D015470		Calcitriol|[	17.77	14
SYNGR1	9145	Leukemia, MESH:D01	marker/m	Arsenic Tr	17.76	27
ARHGDIA	396	Leukemia, MESH:D015473		Genistein|I	17.76	155
CCNH	902	Leukemia, MESH:D015470		Arsenic Tr	17.76	25
CFP	5199	Leukemia, MESH:D015470		Benzene|(	17.76	26
DUSP3	1845	Leukemia, MESH:D015470		Decitabine	17.76	20
HIBADH	11112	Leukemia, MESH:D015470		Arsenic Tr	17.76	20
KYAT1	883	Leukemia, MESH:D015470		Decitabine	17.76	14
LNPEP	4012	Leukemia, MESH:D015470		Dronabinc	17.76	13
MDK	4192	Leukemia, MESH:D015473		arsenite|C	17.76	160
MYL6	4637	Leukemia, MESH:D015470		Dexameth	17.76	14
OSBPL3	26031	Leukemia, MESH:D015470		Dexameth	17.76	15
PLA2G6	8398	Leukemia, MESH:D015470		Arsenic Tr	17.76	36
PSMB1	5689	Leukemia, MESH:D015473		Arsenic Tr	17.76	156
SLC46A3	283537	Leukemia, MESH:D015470		Dexameth	17.76	13
SPTBN2	6712	Leukemia, MESH:D015470		Benzene|C	17.76	24
SRSF1	6426	Leukemia, MESH:D015473		Antimony	17.76	156
ANXA6	309	Leukemia, MESH:D015473		arsenite|C	17.75	158
CACYBP	27101	Leukemia, MESH:D015473		Arsenic|ar	17.75	159
CCL22	6367	Leukemia, MESH:D015473		Arsenic|Ar	17.75	265
DLG1	1739	Leukemia, MESH:D015473		Arsenic Tr	17.75	263
ESPL1	9700	Leukemia, MESH:D015473		arsenite|C	17.75	9
HLA-G	3135	Leukemia, MESH:D015473		Arsenic|Ar	17.75	265
SLC27A1	376497	Leukemia, MESH:D015473		arsenite|C	17.75	154
CD79A	973	Leukemia, MESH:D015473		Genistein|I	17.74	156
CREB3	10488	Leukemia, MESH:D015473		Calcitriol|N	17.74	156
EPX	8288	Leukemia, MESH:D015473		Arsenic Tr	17.74	262
KLHL41	10324	Leukemia, MESH:D015470		Cytarabine	17.74	78
MMS22L	253714	Leukemia, MESH:D015473		Calcitriol|[	17.74	153
PRPF6	24148	Leukemia, MESH:D015470		Bortezomi	17.74	13
TP53I11	9537	Leukemia, MESH:D015473		Arsenic|Ar	17.74	264
UBXN1	51035	Leukemia, MESH:D015470		Doxorubic	17.74	14
ZBTB24	9841	Leukemia, MESH:D015470		Etoposide	17.74	18
AAMDC	28971	Leukemia, MESH:D015473		Arsenic|ar	17.73	156
ATAD2B	54454	Leukemia, MESH:D015470		Dexameth	17.73	13
ATP5PF	522	Leukemia, MESH:D015470		Doxorubic	17.73	13
BCAS2	10286	Leukemia, MESH:D015470		Dexameth	17.73	10
CD247	919	Leukemia, MESH:D015473		Arsenic|C	17.73	155
CFAP20	29105	Leukemia, MESH:D015470		Air Polluta	17.73	13
DCAF13	25879	Leukemia, MESH:D015470		Bortezomi	17.73	9
DCLRE1C	64421	Leukemia, MESH:D015470		Arsenic Tr	17.73	14
EMC9	51016	Leukemia, MESH:D015470		Dexameth	17.73	10
ENPP4	22875	Leukemia, MESH:D015470		Doxorubic	17.73	13
EXOC8	149371	Leukemia, MESH:D015470		Dexameth	17.73	12
EXOSC6	118460	Leukemia, MESH:D015470		Doxorubic	17.73	14
FAM78A	286336	Leukemia, MESH:D015470		Air Polluta	17.73	8
FBXL7	23194	Leukemia, MESH:D015470		Arsenic Tr	17.73	15
GADD45B	4616	Leukemia, MESH:D007948		Arsenic Tr	17.73	3
GATAD1	57798	Leukemia, MESH:D015470		Doxorubic	17.73	13

HMGA1	3159	Leukemia, MESH:D015470	alvocidib|/	17.73	16
KLRG1	10219	Leukemia, MESH:D015470	Dexameth	17.73	9
LEPROTL1	23484	Leukemia, MESH:D015470	Benzene|D	17.73	22
LETM1	3954	Leukemia, MESH:D015470	Decitabine	17.73	19
LRRN4	164312	Leukemia, MESH:D015470	Cytarabine	17.73	80
MAML1	9794	Leukemia, MESH:D015470	Arsenic Tr	17.73	15
METTL3	56339	Leukemia, MESH:D015473	Arsenic|Ar	17.73	264
NFE2L2A	360149	Leukemia, MESH:D015470	Arsenic Tr	17.73	16
NTNG2	84628	Leukemia, MESH:D015470	Calcitriol|D	17.73	8
PIP4P2	55529	Leukemia, MESH:D015470	Doxorubic	17.73	14
PLCH2	9651	Leukemia, MESH:D015470	Dexameth	17.73	7
PLEKHG1	57480	Leukemia, MESH:D015473	Arsenic|Ar	17.73	264
PPP1R13B	23368	Leukemia, MESH:D015470	Arsenic Tr	17.73	18
RELT	84957	Leukemia, MESH:D015470	Doxorubic	17.73	16
RSBN1	54665	Leukemia, MESH:D015470	Doxorubic	17.73	14
ST6GALN/	81849	Leukemia, MESH:D015470	Benzene|N	17.73	24
YIF1A	10897	Leukemia, MESH:D015470	Cytarabine	17.73	80
YLPM1	56252	Leukemia, MESH:D015470	Arsenic Tr	17.73	19
ZBED3	84327	Leukemia, MESH:D015470	Dexameth	17.73	7
ZNFX1	57169	Leukemia, MESH:D015470	Dexameth	17.73	16
ARSJ	79642	Leukemia, MESH:D015470	Doxorubic	17.72	13
ASB9	140462	Leukemia, MESH:D015473	Arsenic Tr	17.72	262
ATXN7L1	222255	Leukemia, MESH:D015470	Dexameth	17.72	12
BROX	148362	Leukemia, MESH:D015470	Dexameth	17.72	13
CCDC3	83643	Leukemia, MESH:D015470	Dexameth	17.72	13
CHFR	55743	Leukemia, MESH:D015473	Arsenic|ars	17.72	11
CSRNP3	80034	Leukemia, MESH:D015470	Doxorubic	17.72	13
DDX11	1663	Leukemia, MESH:D015473	Arsenic|Ca	17.72	156
EIF3F	8665	Leukemia, MESH:D015473	Arsenic Tr	17.72	262
ERLIN2	11160	Leukemia, MESH:D015473	Arsenic|De	17.72	11
GHRL	51738	Leukemia, MESH:D015473	Arsenic|De	17.72	155
LILRB1	10859	Leukemia, MESH:D015470	Air Polluta	17.72	17
MGST3	4259	Leukemia, MESH:D015470	Dexameth	17.72	17
MIR200B	406984	Leukemia, MESH:D015473	Arsenic|De	17.72	158
SNORD10	692227	Leukemia, MESH:D015470	Gasoline|T	17.72	10
UBE3C	9690	Leukemia, MESH:D015473	Arsenic|ars	17.72	11
ZCCHC14	23174	Leukemia, MESH:D015473	Arsenic|ars	17.72	157
ZNF232	7775	Leukemia, MESH:D015470	Dexameth	17.72	14
CRYBA2	1412	Leukemia, MESH:D015470	Alitretinoi	17.71	7
FAT1	2195	Leukemia, MESH:D015473	Arsenic Tr	17.71	264
RAB31	11031	Leukemia, MESH:D015473	Arsenic|Ar	17.71	268
SLC4A1	6521	Leukemia, MESH:D015473	Cholestero	17.71	155
CGREF1	10669	Leukemia, MESH:D015470	Bortezomi	17.7	22
ANO8	57719	Leukemia, MESH:D015470	Cytarabine	17.69	78
C16ORF87	388272	Leukemia, MESH:D015470	Irinotecan	17.69	6
CAMK2G	818	Leukemia, MESH:D015470	Alitretinoi	17.69	21
CYP2R1	120227	Leukemia, MESH:D015470	Dexameth	17.69	9
DDAH2	23564	Leukemia, MESH:D015470	Doxorubic	17.69	15
EI24	9538	Leukemia, MESH:D015470	Daunorub	17.69	45
EPD	30199	Leukemia, MESH:D015470	Dronabinc	17.69	7
FZD8	8325	Leukemia, MESH:D015470	Decitabine	17.69	16
H3F4	382523	Leukemia, MESH:D015470	Arsenic Tr	17.69	18
HIVEP2	3097	Leukemia, MESH:D015470	Benzene|C	17.69	28
LRP4	4038	Leukemia, MESH:D015470	Arsenic Tr	17.69	14
METAP2	10988	Leukemia, MESH:D015470	Doxorubic	17.69	16
RHOBTB1	9886	Leukemia, MESH:D015470	Arsenic Tr	17.69	16
SCARNA1	677769	Leukemia, MESH:D015470	Bortezomi	17.69	7
SF1	7536	Leukemia, MESH:D015470	Dexameth	17.69	15

TANK	10010	Leukemia, MESH:D015470		Arsenic Tr	17.69	22
TLN2	83660	Leukemia, MESH:D015470		Cytarabine	17.69	83
YBX1	4904	Leukemia, MESH:D015470		Air Polluta	17.69	16
ZMIZ1	57178	Leukemia, MESH:D015470		Dexameth	17.69	9
ITGB2	3689	Leukemia, MESH:D01	marker/m	Arsenic Tr	17.68	266
C12ORF75	387882	Leukemia, MESH:D015470		(+)-JQ1 co	17.68	7
DDX60L	91351	Leukemia, MESH:D015470		Air Polluta	17.68	12
LRPPRC	10128	Leukemia, MESH:D015470		Dexameth	17.68	14
NFIL3	4783	Leukemia, MESH:D015470		Benzoates	17.68	26
TIMP2	7077	Leukemia, MESH:D007948		Arsenic Tr	17.68	3
ZNF101	94039	Leukemia, MESH:D015470		Dexameth	17.68	6
ZNF572	137209	Leukemia, MESH:D015470		Dexameth	17.68	6
PTPN11	5781	Leukemia, MESH:D01	marker/m	15-deoxy-	17.67	21
ADGB	79747	Leukemia, MESH:D015473		Arsenic|M	17.67	11
DYRK4	8798	Leukemia, MESH:D015470		Dexameth	17.67	11
ELN	2006	Leukemia, MESH:D015473		Calcitriol|D	17.67	154
LCAT	3931	Leukemia, MESH:D015470		Decitabine	17.67	22
MGP	4256	Leukemia, MESH:D004915		Cytarabine	17.67	3
MVK	4598	Leukemia, MESH:D015470		Dexameth	17.67	12
PGR	5241	Leukemia, MESH:D015473		Arsenic|ars	17.67	160
RRAD	6236	Leukemia, MESH:D015470		Calcitriol|D	17.67	26
TDRD3	81550	Leukemia, MESH:D015473		arsenite|Et	17.67	155
AGRN	375790	Leukemia, MESH:D015473		Arsenic|Da	17.66	160
BAK1	578	Leukemia, MESH:D007948		Arsenic Tr	17.66	3
CADM1	23705	Leukemia, MESH:D015473		Arsenic|Ar	17.66	267
IRS1	3667	Leukemia, MESH:D007948		Arsenic Tr	17.66	3
KRR1	11103	Leukemia, MESH:D015473		Arsenic|M	17.66	15
PLSCR1	5359	Leukemia, MESH:D015473		Arsenic Tr	17.66	263
FSTL1	11167	Leukemia, MESH:D015473		Dexameth	17.65	155
ITGBL1	9358	Leukemia, MESH:D015473		Arsenic Tr	17.65	153
MICAL3	57553	Leukemia, MESH:D015473		Calcitriol|M	17.65	153
MT1F	4494	Leukemia, MESH:D015473		Arsenic|Ar	17.65	266
NOLC1	9221	Leukemia, MESH:D015473		Arsenic Tr	17.65	261
ORC4	5000	Leukemia, MESH:D015473		Arsenic|Ar	17.65	160
PRDX5	25824	Leukemia, MESH:D015473		Arsenic|Ar	17.65	265
RAVER2	55225	Leukemia, MESH:D015473		Dasatinib|	17.65	153
RCHY1	25898	Leukemia, MESH:D015473		Arsenic|Ar	17.65	265
WDR72	256764	Leukemia, MESH:D015473		Arsenic|Ca	17.65	155
ZNF343	79175	Leukemia, MESH:D015473		Arsenic|ars	17.65	156
ALG14	199857	Leukemia, MESH:D015473		Arsenic|De	17.64	156
APPBP2	10513	Leukemia, MESH:D015473		Calcitriol|D	17.64	7
CNST	163882	Leukemia, MESH:D015473		Arsenic|ars	17.64	13
EDNRA	1909	Leukemia, MESH:D015470		Daunorub	17.64	42
GTF2A1	2957	Leukemia, MESH:D015473		Arsenic|Ar	17.64	263
PRRG1	5638	Leukemia, MESH:D015473		Arsenic|Ar	17.64	158
SAP30	8819	Leukemia, MESH:D015473		arsenite|D	17.64	158
SGCD	6444	Leukemia, MESH:D015473		Arsenic Tr	17.64	261
DCN	1634	Leukemia, MESH:D004915		Cytarabine	17.63	3
MSTN	2660	Leukemia, MESH:D015470		Androgen	17.63	13
PAX5	5079	Leukemia, MESH:D015473		Arsenic Tr	17.63	262
ATP5ME	521	Leukemia, MESH:D015470		Arsenic Tr	17.62	41
ATP6V0D2	245972	Leukemia, MESH:D015470		Deferoxan	17.62	10
CASP14	23581	Leukemia, MESH:D015470		Calcitriol|D	17.62	13
CHAF1A	10036	Leukemia, MESH:D015470		Bortezomi	17.62	22
CLTC	1213	Leukemia, MESH:D015470		Arsenic Tr	17.62	41
FTSJ3	117246	Leukemia, MESH:D015470		Air Polluta	17.62	10
GTF2H4	2968	Leukemia, MESH:D015470		Dexameth	17.62	11
H1-4	3008	Leukemia, MESH:D015470		Dasatinib|	17.62	12

HSD11B2	3291	Leukemia, MESH:D015473	Calcitriol|D	17.62	8
HYAL2	8692	Leukemia, MESH:D015470	Benzene|D	17.62	27
IFT20	90410	Leukemia, MESH:D015470	Dexameth	17.62	15
KCNQ2	3785	Leukemia, MESH:D015470	Dronabinc	17.62	12
MINPP1	9562	Leukemia, MESH:D015470	Air Polluta	17.62	13
MRPS31	10240	Leukemia, MESH:D015470	Air Polluta	17.62	13
MYL3	4634	Leukemia, MESH:D015470	Dexameth	17.62	21
OPN3	23596	Leukemia, MESH:D015470	Arsenic Tr	17.62	19
OSBPL7	114881	Leukemia, MESH:D015470	Alitretinoi	17.62	13
PLA2G7	7941	Leukemia, MESH:D015470	Benzene|C	17.62	24
PORCN	64840	Leukemia, MESH:D015470	Decitabine	17.62	21
RAB37	326624	Leukemia, MESH:D015470	Cytarabine	17.62	76
RAD9A	5883	Leukemia, MESH:D015470	Arsenic Tr	17.62	29
TCEAL1	9338	Leukemia, MESH:D015470	Bortezomi	17.62	13
USH1C	10083	Leukemia, MESH:D015470	Dronabinc	17.62	10
WNK2	65268	Leukemia, MESH:D015470	Arsenic Tr	17.62	16
AKT3	10000	Leukemia, MESH:D015470	Doxorubic	17.61	15
ATP6V1E1	529	Leukemia, MESH:D015470	Arsenic Tr	17.61	18
BAIAP2	10458	Leukemia, MESH:D015470	Calcitriol|C	17.61	9
BOLA3	388962	Leukemia, MESH:D015470	Dexameth	17.61	13
CBX4	8535	Leukemia, MESH:D015470	Arsenic Tr	17.61	20
CCDC126	90693	Leukemia, MESH:D015470	Doxorubic	17.61	13
CORO1C	23603	Leukemia, MESH:D015470	Dexameth	17.61	11
CUL5	8065	Leukemia, MESH:D015470	Arsenic Tr	17.61	19
DERL2	51009	Leukemia, MESH:D015470	Dexameth	17.61	13
DIDO1	11083	Leukemia, MESH:D015470	Benzene|D	17.61	26
DUSP5	1847	Leukemia, MESH:D015473	Arsenic Tr	17.61	263
ERC2	26059	Leukemia, MESH:D015470	Arsenic Tr	17.61	16
ERCC6L	54821	Leukemia, MESH:D015470	Calcitriol|D	17.61	14
ETFDH	2110	Leukemia, MESH:D015470	Dexameth	17.61	24
FGL1	2267	Leukemia, MESH:D015470	Arsenic Tr	17.61	16
INHBB	3625	Leukemia, MESH:D015470	Arsenic Tr	17.61	15
KIF13B	23303	Leukemia, MESH:D015470	Arsenic Tr	17.61	20
KIF7	374654	Leukemia, MESH:D015470	Calcitriol|D	17.61	9
LAPTM5	7805	Leukemia, MESH:D015470	Arsenic Tr	17.61	20
MEPCE	56257	Leukemia, MESH:D015470	Dexameth	17.61	12
MIER1	57708	Leukemia, MESH:D015470	Dexameth	17.61	8
NDUFS6	4726	Leukemia, MESH:D015470	Dexameth	17.61	13
PACSIN3	29763	Leukemia, MESH:D015470	Doxorubic	17.61	12
PDXDC1	23042	Leukemia, MESH:D015470	Arsenic Tr	17.61	18
POMP	51371	Leukemia, MESH:D015470	Doxorubic	17.61	13
QTRT1	81890	Leukemia, MESH:D015470	Arsenic Tr	17.61	18
RAB24	53917	Leukemia, MESH:D015470	Arsenic Tr	17.61	13
RCSD1	92241	Leukemia, MESH:D015470	Doxorubic	17.61	13
SCN2A	6326	Leukemia, MESH:D015470	Arsenic Tr	17.61	14
SHC3	53358	Leukemia, MESH:D015470	Dexameth	17.61	12
SLC9A8	23315	Leukemia, MESH:D015470	Doxorubic	17.61	15
SLCO3A1	28232	Leukemia, MESH:D015470	Arsenic Tr	17.61	20
SPECC1	92521	Leukemia, MESH:D015470	Calcitriol|D	17.61	8
UPB1	51733	Leukemia, MESH:D015470	Dexameth	17.61	14
WAC	51322	Leukemia, MESH:D015470	Doxorubic	17.61	14
ERBB3	2065	Leukemia, MESH:D004915	Daunorub	17.6	2
FOXA1	3169	Leukemia, MESH:D015473	Arsenic|Ar	17.6	266
FOXA2	3170	Leukemia, MESH:D015473	arsenite|D	17.6	157
NABP1	64859	Leukemia, MESH:D015473	Arsenic Tr	17.6	262
CXCR4	7852	Leukemia, MESH:D007948	Arsenic Tr	17.59	4
GSK3B	2932	Leukemia, MESH:D004915	Doxorubic	17.59	4
ALOX5	240	Leukemia, MESH:D007948	2-(2-amir	17.58	2

CBLL1	79872	Leukemia, MESH:D015473	Arsenic|ars	17.58	10
DNAJB9	4189	Leukemia, MESH:D015473	Arsenic Tr	17.58	261
SNCA	6622	Leukemia, MESH:D007948	Arsenic Tr	17.58	4
SP110	3431	Leukemia, MESH:D015473	Arsenic|Ar	17.58	265
VRK2	7444	Leukemia, MESH:D015473	Cytarabine	17.58	159
DNMT1	1786	Leukemia, MESH:D007948	2-(2-amin	17.57	3
MIR141	406933	Leukemia, MESH:D015473	Arsenic|Bu	17.57	11
RXRA	6256	Leukemia, MESH:D007948	Arsenic Tr	17.57	3
ADIPOR2	79602	Leukemia, MESH:D015473	Arsenic|De	17.56	156
CDH2	1000	Leukemia, MESH:D007948	2-(2-amin	17.56	3
EBP	10682	Leukemia, MESH:D015470	Doxorubic	17.56	16
PROCR	10544	Leukemia, MESH:D015473	Arsenic|ars	17.56	155
RASSF1	11186	Leukemia, MESH:D015473	Arsenic|Ar	17.56	162
TRIM5	85363	Leukemia, MESH:D015473	arsenite|Cy	17.56	157
ZBTB20	26137	Leukemia, MESH:D015473	Arsenic|Da	17.56	161
CUL2	8453	Leukemia, MESH:D015473	Arsenic|Ar	17.55	160
CYGB	114757	Leukemia, MESH:D015473	Arsenic Tr	17.55	261
DPYSL3	1809	Leukemia, MESH:D015473	Arsenic Tr	17.55	262
ERGIC1	57222	Leukemia, MESH:D015473	Arsenic|ars	17.55	156
HMGCS2	3158	Leukemia, MESH:D015473	caffeic aci	17.55	18
IL21R	50615	Leukemia, MESH:D015473	Dexameth	17.55	153
KRT17	3872	Leukemia, MESH:D015473	arsenite|Ca	17.55	156
NTM	50863	Leukemia, MESH:D015473	Arsenic|Cy	17.55	158
PMPCB	9512	Leukemia, MESH:D015473	Arsenic Tr	17.55	156
PNP	4860	Leukemia, MESH:D015473	Arsenic|Ar	17.55	266
AGR2	10551	Leukemia, MESH:D015470	Arsenic Tr	17.54	17
AKR1C1	1645	Leukemia, MESH:D015470	Arsenic Tr	17.54	19
BAZ1A	11177	Leukemia, MESH:D015470	Doxorubic	17.54	16
BRIP1	83990	Leukemia, MESH:D015470	Calcitriol|D	17.54	18
COX11	1353	Leukemia, MESH:D015473	Arsenic Tr	17.54	261
DERL1	79139	Leukemia, MESH:D015473	Arsenic|De	17.54	158
HFE	3077	Leukemia, MESH:D015470	Arsenic Tr	17.54	20
HOXB5	3215	Leukemia, MESH:D015473	Arsenic|ars	17.54	157
LGR5	8549	Leukemia, MESH:D015470	Arsenic Tr	17.54	19
OAS2	4939	Leukemia, MESH:D015470	Air Polluta	17.54	35
PLIN1	5346	Leukemia, MESH:D015470	15-deoxy-	17.54	10
PLPP5	84513	Leukemia, MESH:D015473	arsenite|D	17.54	155
PPP1R14B	26472	Leukemia, MESH:D015470	Dexameth	17.54	12
RHOBTB3	22836	Leukemia, MESH:D015470	Dexameth	17.54	15
RIF1	55183	Leukemia, MESH:D015470	Pentachlo	17.54	13
TSPAN8	7103	Leukemia, MESH:D015470	Dexameth	17.54	11
YBX3	8531	Leukemia, MESH:D015470	Dexameth	17.54	18
ZNF106A	437005	Leukemia, MESH:D015473	Dexameth	17.54	4
ECHS1	1892	Leukemia, MESH:D015473	Arsenic|Ar	17.52	162
FLOT1A	561345	Leukemia, MESH:D015473	Arsenic|De	17.52	8
MUCL1	118430	Leukemia, MESH:D015470	Cyclophos	17.52	8
MYH7	4625	Leukemia, MESH:D004915	Daunorub	17.52	3
SLC24A5	283652	Leukemia, MESH:D015470	Doxorubic	17.52	12
ANXA9	8416	Leukemia, MESH:D015470	Arsenic Tr	17.51	17
ATF6B	1388	Leukemia, MESH:D015470	Arsenic Tr	17.51	16
CHRD	8646	Leukemia, MESH:D015470	Decitabine	17.51	17
FMN2	56776	Leukemia, MESH:D015470	Arsenic Tr	17.51	19
FOSL1	8061	Leukemia, MESH:D007948	2-(2-amin	17.51	2
G6PC3	92579	Leukemia, MESH:D015470	Arsenic Tr	17.51	15
IL15RA	3601	Leukemia, MESH:D015470	Cyclophos	17.51	13
L1TD1	54596	Leukemia, MESH:D015470	Decitabine	17.51	16
MT3	4504	Leukemia, MESH:D015470	Arsenic Tr	17.51	30
OSBP	5007	Leukemia, MESH:D015470	Benzene|D	17.51	25

PF4	5196	Leukemia, MESH:D015473		Arsenic|De	17.51	12
RBM4B	83759	Leukemia, MESH:D015470		Dexameth	17.51	10
REPIN1	29803	Leukemia, MESH:D015470		Arsenic Tr	17.51	20
RFX8	731220	Leukemia, MESH:D015473		Calcitriol|D	17.51	151
SCRIB	23513	Leukemia, MESH:D015470		Dexameth	17.51	8
SNAPC1	6617	Leukemia, MESH:D015470		Calcitriol|D	17.51	13
SRL	6345	Leukemia, MESH:D015470		Dexameth	17.51	12
THBS2	7058	Leukemia, MESH:D015473		arsenite|Ca	17.51	155
TMC4	147798	Leukemia, MESH:D015470		Calcitriol|N	17.51	12
TRIM23	373	Leukemia, MESH:D015470		Air Polluta	17.51	18
UQCC2	84300	Leukemia, MESH:D015470		Air Polluta	17.51	19
ZFP36L1	677	Leukemia, MESH:D015473		Arsenic Tr	17.51	260
CAPZA2	830	Leukemia, MESH:D015470		Arsenic Tr	17.5	19
CCNJ	54619	Leukemia, MESH:D015470		Dexameth	17.5	8
DENND2C	163259	Leukemia, MESH:D015470		Dronabinc	17.5	8
DGLUCY	80017	Leukemia, MESH:D015470		Doxorubic	17.5	13
FAM172A	83989	Leukemia, MESH:D015470		Air Polluta	17.5	12
GSN	2934	Leukemia, MESH:D004915		Doxorubic	17.5	3
HPCA	3208	Leukemia, MESH:D015470		Arsenic Tr	17.5	13
ISYNA1	51477	Leukemia, MESH:D015473		arsenite|D	17.5	157
KIAA1522	57648	Leukemia, MESH:D015473		Arsenic|Ar	17.5	157
LSM11	134353	Leukemia, MESH:D015470		Arsenic Tr	17.5	19
MAGEH1	28986	Leukemia, MESH:D015470		Dexameth	17.5	13
MAGT1	84061	Leukemia, MESH:D015470		Dexameth	17.5	15
MAK	4117	Leukemia, MESH:D015470		Arsenic Tr	17.5	16
NR4A1	3164	Leukemia, MESH:D007948		2-(2-amin	17.5	3
NUP58	9818	Leukemia, MESH:D015470		Indometha	17.5	9
PLA2R1	22925	Leukemia, MESH:D015470		Arsenic Tr	17.5	25
PPIH	10465	Leukemia, MESH:D015473		Antimony	17.5	155
RAB21	23011	Leukemia, MESH:D015470		Arsenic Tr	17.5	23
RILPL2	196383	Leukemia, MESH:D015470		Doxorubic	17.5	13
RNF170	81790	Leukemia, MESH:D015470		Dexameth	17.5	8
SBF2	81846	Leukemia, MESH:D015470		Doxorubic	17.5	17
SCEL	8796	Leukemia, MESH:D015470		Calcitriol|D	17.5	8
SEZ6L2	26470	Leukemia, MESH:D015470		Calcitriol|D	17.5	13
SLC18B1	116843	Leukemia, MESH:D015470		Calcitriol|D	17.5	13
SLC7A10	56301	Leukemia, MESH:D015470		Decitabine	17.5	14
SYNC	81493	Leukemia, MESH:D015470		Calcitriol|D	17.5	7
TBCEL	219899	Leukemia, MESH:D015470		Dexameth	17.5	12
TMEM156	80008	Leukemia, MESH:D015470		Calcitriol|D	17.5	13
ZNF608	57507	Leukemia, MESH:D015470		Dexameth	17.5	13
AAR2	25980	Leukemia, MESH:D015470		Benzene|D	17.49	27
CER1	9350	Leukemia, MESH:D015470		Dexameth	17.49	7
DUOX1	53905	Leukemia, MESH:D015473		Antimony	17.49	153
EVI5	7813	Leukemia, MESH:D015473		Arsenic|Ca	17.49	13
GALK2	2585	Leukemia, MESH:D015473		Arsenic|Ar	17.49	267
PHOX2B	8929	Leukemia, MESH:D015470		Decitabine	17.49	14
SMC5	23137	Leukemia, MESH:D015473		Arsenic Tr	17.49	264
SSUH2	51066	Leukemia, MESH:D015470		Cytarabine	17.49	74
DHCR7	1717	Leukemia, MESH:D01	marker/m	Arsenic|Ca	17.48	159
ARHGAP8	23779	Leukemia, MESH:D015473		Arsenic|Ge	17.48	159
EHBP1L1	254102	Leukemia, MESH:D015473		Arsenic|Ca	17.48	157
EIF4EBP1	1978	Leukemia, MESH:D007948		2-(2-amin	17.48	3
FRK	2444	Leukemia, MESH:D015473		Arsenic Tr	17.48	260
KSR1	8844	Leukemia, MESH:D015473		arsenite|Ca	17.48	153
NCOA2	10499	Leukemia, MESH:D004915		Daunorub	17.48	3
ZNF10	7556	Leukemia, MESH:D015470		Doxorubic	17.48	13
ZNF248	57209	Leukemia, MESH:D015470		Dexameth	17.48	6

ZNF689	115509	Leukemia, MESH:D015470	Dexameth	17.48	11
ALG10	84920	Leukemia, MESH:D015470	Dexameth	17.47	7
C6ORF47	57827	Leukemia, MESH:D015470	Dexameth	17.47	7
CCDC198	55195	Leukemia, MESH:D015473	Arsenic Tr	17.47	261
COLEC12	81035	Leukemia, MESH:D015470	Calcitriol|C	17.47	83
CUX1	1523	Leukemia, MESH:D015470	(+)-JQ1 cc	17.47	11
FER	2241	Leukemia, MESH:D015473	Arsenic|Ar	17.47	159
GFRA2	2675	Leukemia, MESH:D015470	Dexameth	17.47	11
HIBCH	26275	Leukemia, MESH:D015470	Calcitriol|N	17.47	12
KIAA0232	9778	Leukemia, MESH:D015470	Dexameth	17.47	7
MMP14	4323	Leukemia, MESH:D015470	Arsenic Tr	17.47	27
OGFR	11054	Leukemia, MESH:D015473	Genistein|I	17.47	154
PDE1A	5136	Leukemia, MESH:D015470	Arsenic Tr	17.47	83
SLC22A23	63027	Leukemia, MESH:D015470	Calcitriol|D	17.47	13
TMEM204	79652	Leukemia, MESH:D015473	Dexameth	17.47	153
ZNF157	7712	Leukemia, MESH:D015470	Decitabine	17.47	11
ZNF77	58492	Leukemia, MESH:D015470	Doxorubic	17.47	12
BCL2L11	10018	Leukemia, MESH:D007948	Arsenic Tr	17.46	3
GNA12	2768	Leukemia, MESH:D015470	Dexameth	17.46	15
LTBP1	4052	Leukemia, MESH:D015473	Arsenic|ars	17.46	158
MMP9	4318	Leukemia, MESH:D007948	2-(2-amin	17.46	3
PMAIP1	5366	Leukemia, MESH:D007948	2-(2-amin	17.46	3
PRSS1	5644	Leukemia, MESH:D015473	Arsenic Tr	17.46	261
RBM47	54502	Leukemia, MESH:D015470	Arsenic Tr	17.46	28
RNF144B	255488	Leukemia, MESH:D015470	Doxorubic	17.46	15
SMARCA4	6597	Leukemia, MESH:D015473	Arsenic|Ar	17.46	264
ZMYND11	10771	Leukemia, MESH:D015473	Dexameth	17.46	153
MIF	4282	Leukemia, MESH:D015473	Arsenic|Ar	17.45	266
SMAD3	4088	Leukemia, MESH:D007948	Arsenic Tr	17.45	3
USP41	373856	Leukemia, MESH:D015470	Dexameth	17.45	12
ATM	472	Leukemia, MESH:D007948	2-(2-amin	17.44	3
ZNF584	201514	Leukemia, MESH:D015470	Doxorubic	17.44	12
ALPL	249	Leukemia, MESH:D007948	2-(2-amin	17.43	3
DEFA4	1669	Leukemia, MESH:D015470	Arsenic Tr	17.43	11
DPY19L1P	1E+08	Leukemia, MESH:D015470	Dexameth	17.43	6
ISLR	3671	Leukemia, MESH:D015473	Dexameth	17.43	151
THAP7-AS	439931	Leukemia, MESH:D015470	Dexameth	17.43	5
NTRK2	4915	Leukemia, MESH:D015473	Arsenic|Ar	17.42	267
GFRA1	2674	Leukemia, MESH:D015473	Arsenic|De	17.41	158
H19	283120	Leukemia, MESH:D015473	Arsenic|ars	17.41	160
NUCB2	4925	Leukemia, MESH:D015473	Arsenic Tr	17.41	262
TRAF6	7189	Leukemia, MESH:D015473	Arsenic|Ar	17.41	264
ARHGAP1	79658	Leukemia, MESH:D015470	Calcitriol|D	17.4	13
ARMC9	80210	Leukemia, MESH:D015473	Arsenic Tr	17.4	258
BBS4	585	Leukemia, MESH:D015470	Cyclophos	17.4	12
CLDND1	56650	Leukemia, MESH:D015470	Doxorubic	17.4	11
GOLGA2	2801	Leukemia, MESH:D015470	Arsenic Tr	17.4	14
GPER1	2852	Leukemia, MESH:D015470	Arsenic Tr	17.4	91
MLF2	8079	Leukemia, MESH:D015470	Dexameth	17.4	7
MRPS18C	51023	Leukemia, MESH:D015470	Gasoline|Ir	17.4	10
MYNN	55892	Leukemia, MESH:D015470	Benzene|C	17.4	26
PBDC1	51260	Leukemia, MESH:D015470	Benzene|D	17.4	22
PELI2	57161	Leukemia, MESH:D015473	Arsenic|Ge	17.4	155
PPP2CB	5516	Leukemia, MESH:D015470	Air Polluta	17.4	25
RNF138	51444	Leukemia, MESH:D015470	Bortezomi	17.4	8
SCYL1	57410	Leukemia, MESH:D015470	Cytarabine	17.4	79
SNRNP200	23020	Leukemia, MESH:D015473	Resveratrc	17.4	159
SYT4	6860	Leukemia, MESH:D015470	Arsenic Tr	17.4	13

TRIM21	6737	Leukemia, MESH:D015470	Arsenic Tr	17.4	19
TRIM33	51592	Leukemia, MESH:D015470	Gasoline|(	17.4	9
VPS53	55275	Leukemia, MESH:D015473	Arsenic Tr	17.4	157
VTI1A	143187	Leukemia, MESH:D015470	Dexameth	17.4	15
WDR47	22911	Leukemia, MESH:D015470	Azacitidin	17.4	13
ACOT4	122970	Leukemia, MESH:D015470	Dronabinc	17.39	12
ATP6V1B2	526	Leukemia, MESH:D015470	Air Polluta	17.39	22
B3GNT7	93010	Leukemia, MESH:D015470	Doxorubic	17.39	13
BRD1	23774	Leukemia, MESH:D015470	Dronabinc	17.39	9
CABYR	26256	Leukemia, MESH:D015470	Doxorubic	17.39	15
CPED1	79974	Leukemia, MESH:D015470	Dexameth	17.39	13
CPNE1	8904	Leukemia, MESH:D015470	Arsenic Tr	17.39	25
DONSON	29980	Leukemia, MESH:D015473	Calcitriol|D	17.39	9
GARNL3	84253	Leukemia, MESH:D015470	Arsenic Tr	17.39	18
GNG4	2786	Leukemia, MESH:D015473	arsenite|D	17.39	158
HEATR3	55027	Leukemia, MESH:D015470	Dexameth	17.39	8
IL6	3569	Leukemia, MESH:D007948	2-(2-amin	17.39	4
INPP4A	3631	Leukemia, MESH:D015470	Doxorubic	17.39	16
ITGA7	3679	Leukemia, MESH:D015470	Calcitriol|D	17.39	13
KCNIP3	30818	Leukemia, MESH:D015470	Dronabinc	17.39	10
KCNK6	9424	Leukemia, MESH:D015470	Arsenic Tr	17.39	19
KIRREL3	84623	Leukemia, MESH:D015470	Dexameth	17.39	7
KLF12	11278	Leukemia, MESH:D015470	Doxorubic	17.39	18
KLHL21	9903	Leukemia, MESH:D015470	Arsenic Tr	17.39	18
MATN3	4148	Leukemia, MESH:D015473	Arsenic|Ca	17.39	11
NCKAP5	344148	Leukemia, MESH:D015470	Calcitriol|D	17.39	12
NDUFB11	54539	Leukemia, MESH:D015470	Dexameth	17.39	12
NISCH	11188	Leukemia, MESH:D015470	Dexameth	17.39	17
NUDT6	11162	Leukemia, MESH:D015470	Dexameth	17.39	8
NUTF2	10204	Leukemia, MESH:D015470	Dexameth	17.39	13
ORMDL2	29095	Leukemia, MESH:D015470	Air Polluta	17.39	14
PDIA5	10954	Leukemia, MESH:D015470	Doxorubic	17.39	14
PEAR1	375033	Leukemia, MESH:D015470	Dexameth	17.39	13
PFDN5	5204	Leukemia, MESH:D015470	Calcitriol|D	17.39	12
PIGQ	9091	Leukemia, MESH:D015470	Dexameth	17.39	12
PITX1	5307	Leukemia, MESH:D015470	Cytarabine	17.39	80
POU2AF1	5450	Leukemia, MESH:D015473	Calcitriol|C	17.39	154
POU4F2	5458	Leukemia, MESH:D015470	Dexameth	17.39	8
PPP1R9B	84687	Leukemia, MESH:D015470	Dexameth	17.39	8
PVT1	5820	Leukemia, MESH:D015470	Benzene|D	17.39	27
RAB3B	5865	Leukemia, MESH:D015470	Dexameth	17.39	14
REEP4	80346	Leukemia, MESH:D015470	Doxorubic	17.39	14
RGS11	8786	Leukemia, MESH:D015470	Arsenic Tr	17.39	19
RHBDF2	79651	Leukemia, MESH:D015470	Dexameth	17.39	14
SLC10A7	84068	Leukemia, MESH:D015470	Bortezomi	17.39	14
SMAGP	57228	Leukemia, MESH:D015473	Arsenic|ar	17.39	156
THSD4	79875	Leukemia, MESH:D015473	Arsenic|Ca	17.39	156
TMX4	56255	Leukemia, MESH:D015470	Doxorubic	17.39	17
UBR1	197131	Leukemia, MESH:D015470	Dexameth	17.39	12
VPS37A	137492	Leukemia, MESH:D015470	Dexameth	17.39	8
FAM110B	90362	Leukemia, MESH:D015473	Arsenic Tr	17.38	261
IGFBP3	3486	Leukemia, MESH:D007948	Arsenic Tr	17.38	4
PPP1R16B	26051	Leukemia, MESH:D015473	Arsenic|ar	17.38	157
CAMSAP2	23271	Leukemia, MESH:D015473	Arsenic|ar	17.37	157
CCR1	1230	Leukemia, MESH:D015473	Arsenic|Ar	17.37	268
CXCL11	6373	Leukemia, MESH:D015473	Calcitriol|D	17.37	154
FLNB	2317	Leukemia, MESH:D015473	Arsenic|Ar	17.37	263
RPL13	6137	Leukemia, MESH:D015473	arsenite|D	17.37	156

TRH	7200	Leukemia, MESH:D015473	Dexameth	17.37	155
CD68	968	Leukemia, MESH:D015470	Calcitriol|D	17.35	21
NFKBIA	4792	Leukemia, MESH:D007948	2-(2-amin	17.35	3
PLOD2	5352	Leukemia, MESH:D015470	Dexameth	17.35	17
UBL3	5412	Leukemia, MESH:D015473	Cytarabine	17.35	155
GSTA1	2938	Leukemia, MESH:D015470	Allopurinc	17.34	29
GSTA4	2941	Leukemia, MESH:D015470	Decitabine	17.34	31
HRAS	3265	Leukemia, MESH:D007948	2-(2-amin	17.34	3
NPC1	4864	Leukemia, MESH:D015473	Arsenic Tr	17.34	162
CAMKK2	10645	Leukemia, MESH:D015470	Calcitriol|C	17.33	16
IRAG1	10335	Leukemia, MESH:D015473	Arsenic|Ca	17.33	9
NGF	4803	Leukemia, MESH:D007948	2-(2-amin	17.33	2
UBE2C	11065	Leukemia, MESH:D004915	Cytarabine	17.33	2
ADHFE1	137872	Leukemia, MESH:D015470	Dexameth	17.32	15
CHGA	1113	Leukemia, MESH:D015470	Azacitidine	17.32	12
CLDN3	1365	Leukemia, MESH:D015473	arsenite|Ca	17.32	156
CPEB4	80315	Leukemia, MESH:D015470	Dronabinc	17.32	9
ERO1A	30001	Leukemia, MESH:D015473	Arsenic|Ca	17.32	159
FRY	10129	Leukemia, MESH:D015470	Bortezomi	17.32	18
IFI27L2	83982	Leukemia, MESH:D015470	Decitabine	17.32	17
KRT7	3855	Leukemia, MESH:D015473	Arsenic Tr	17.32	264
NUCKS1	64710	Leukemia, MESH:D015470	Bortezomi	17.32	15
PLSCR4	57088	Leukemia, MESH:D015473	arsenite|D	17.32	7
PSMB6	5694	Leukemia, MESH:D015470	Air Polluta	17.32	20
SLC15A2	6565	Leukemia, MESH:D015470	Calcitriol|D	17.32	9
TGIF2	60436	Leukemia, MESH:D015473	arsenite|Et	17.32	154
UBA1	7317	Leukemia, MESH:D015470	Arsenic Tr	17.32	22
CD27	939	Leukemia, MESH:D015473	Arsenic|Ar	17.31	157
MAP2K1	5604	Leukemia, MESH:D007948	2-(2-amin	17.31	3
MED13	9969	Leukemia, MESH:D015473	arsenite|Ca	17.31	155
STRIP1	85369	Leukemia, MESH:D015470	Doxorubic	17.31	13
AHR	196	Leukemia, MESH:D007948	2-(2-amin	17.3	3
CCNO	10309	Leukemia, MESH:D015473	Arsenic|Ar	17.3	263
CENPC	1060	Leukemia, MESH:D015470	Arsenic Tr	17.3	18
CYSTM1	84418	Leukemia, MESH:D015473	arsenite|Ca	17.3	155
GFM1	85476	Leukemia, MESH:D015473	arsenite|D	17.3	155
H3C1	8350	Leukemia, MESH:D015470	Benzene|C	17.3	30
HLA-G	3135	Leukemia, MESH:D015470	Arsenic Tr	17.3	22
MIR200C	406985	Leukemia, MESH:D015473	Arsenic|Ar	17.3	159
MYRIP	25924	Leukemia, MESH:D015473	Arsenic|ars	17.3	11
PNPO	55163	Leukemia, MESH:D015473	Arsenic Tr	17.3	154
SLC10A6	345274	Leukemia, MESH:D015473	Arsenic|ars	17.3	11
TDRD5	163589	Leukemia, MESH:D015470	Decitabine	17.3	12
TECRL	253017	Leukemia, MESH:D015470	Cytarabine	17.3	78
TICAM2	353376	Leukemia, MESH:D015470	Arsenic Tr	17.3	13
VPS35L	57020	Leukemia, MESH:D015470	Calcitriol|D	17.3	6
AKAP7	9465	Leukemia, MESH:D015470	Decitabine	17.29	16
CCNDBP1	23582	Leukemia, MESH:D015470	Air Polluta	17.29	15
CD247	919	Leukemia, MESH:D015470	Benzene|C	17.29	24
CD79A	973	Leukemia, MESH:D015470	Bortezomi	17.29	11
CDK16	5127	Leukemia, MESH:D015470	Dronabinc	17.29	8
CDKN2AIF	91368	Leukemia, MESH:D015470	Dexameth	17.29	8
CLIP3	25999	Leukemia, MESH:D015470	Decitabine	17.29	13
CREB3	10488	Leukemia, MESH:D015470	Calcitriol|D	17.29	15
DIAPH1	1729	Leukemia, MESH:D015473	Arsenic Tr	17.29	262
DUSP11	8446	Leukemia, MESH:D015470	Alitretinoi	17.29	13
ELP2	55250	Leukemia, MESH:D015470	Benzene|D	17.29	27
FABP7A	58128	Leukemia, MESH:D015470	Dexameth	17.29	7

FGD3	89846	Leukemia, MESH:D015470	Calcitriol|(	17.29	8
GOLGA8B	440270	Leukemia, MESH:D015470	Doxorubic	17.29	14
ING2	3622	Leukemia, MESH:D015470	Dexameth	17.29	11
ITGB1BP1	9270	Leukemia, MESH:D015470	Dronabinc	17.29	9
LMF1	64788	Leukemia, MESH:D015470	Arsenic Tr	17.29	19
MFSD6	54842	Leukemia, MESH:D015470	Calcitriol|D	17.29	16
MRGBP	55257	Leukemia, MESH:D015470	Doxorubic	17.29	11
MT1E	4493	Leukemia, MESH:D015473	Antimony	17.29	167
NDUFA11	126328	Leukemia, MESH:D015470	Doxorubic	17.29	12
NFS1	9054	Leukemia, MESH:D015473	Dexameth	17.29	155
NPTXR	23467	Leukemia, MESH:D015470	(+)-JQ1 cc	17.29	11
PHC1	1911	Leukemia, MESH:D015470	Air Polluta	17.29	21
PHF21A	51317	Leukemia, MESH:D015470	Arsenic Tr	17.29	20
PLEKHG1	57480	Leukemia, MESH:D015470	Arsenic Tr	17.29	16
RSU1	6251	Leukemia, MESH:D015473	Arsenic Tr	17.29	262
SAMSN1	64092	Leukemia, MESH:D015470	Air Polluta	17.29	11
STAM2	10254	Leukemia, MESH:D015470	Air Polluta	17.29	19
TIMM44	10469	Leukemia, MESH:D015473	Arsenic|De	17.29	158
UBL4A	8266	Leukemia, MESH:D015470	Doxorubic	17.29	12
UCP2	7351	Leukemia, MESH:D007948	2-(2-amin	17.29	3
WDR83OS	51398	Leukemia, MESH:D015470	Dexameth	17.29	10
ZCCHC12	170261	Leukemia, MESH:D015470	Decitabine	17.29	20
ZNF124	7678	Leukemia, MESH:D015470	Dexameth	17.29	7
ZNF469	84627	Leukemia, MESH:D015470	Calcitriol|D	17.29	12
ABHD15	116236	Leukemia, MESH:D015470	Doxorubic	17.28	14
ARPC3	10094	Leukemia, MESH:D015470	Arsenic Tr	17.28	19
BCAR3	8412	Leukemia, MESH:D015473	Arsenic|Cy	17.28	160
BCAS1	8537	Leukemia, MESH:D015470	Calcitriol|D	17.28	7
C1QTNF1	114897	Leukemia, MESH:D015470	Dexameth	17.28	12
COASY	80347	Leukemia, MESH:D015470	Arsenic Tr	17.28	14
DLD	1738	Leukemia, MESH:D015473	Buthionine	17.28	156
EIF3F	8665	Leukemia, MESH:D015470	Arsenic Tr	17.28	13
ESRP2	80004	Leukemia, MESH:D015470	(+)-JQ1 cc	17.28	9
F8A1	8263	Leukemia, MESH:D015470	Dexameth	17.28	11
FABP7	2173	Leukemia, MESH:D015473	Arsenic Tr	17.28	264
FAS	355	Leukemia, MESH:D004915	Cytarabine	17.28	2
FIG4	9896	Leukemia, MESH:D015470	Air Polluta	17.28	13
HSPB2	3316	Leukemia, MESH:D015470	Dexameth	17.28	16
KRT10	3858	Leukemia, MESH:D015473	Antimony	17.28	157
MCAM	4162	Leukemia, MESH:D015473	arsenite|D	17.28	11
MIR200B	406984	Leukemia, MESH:D015470	Dexameth	17.28	14
NFAT5	10725	Leukemia, MESH:D015473	Dexameth	17.28	154
PDLIM1	9124	Leukemia, MESH:D015473	Arsenic|De	17.28	158
PPARGC1I	133522	Leukemia, MESH:D015473	alpha-Toc	17.28	156
SGPP2	130367	Leukemia, MESH:D015470	Dexameth	17.28	12
ST6GALN/	27090	Leukemia, MESH:D015470	Arsenic Tr	17.28	14
TMEM237	65062	Leukemia, MESH:D015470	Doxorubic	17.28	15
TOMM40L	84134	Leukemia, MESH:D015470	Dexameth	17.28	7
TSR1	55720	Leukemia, MESH:D015470	Dexameth	17.28	13
TTC23	64927	Leukemia, MESH:D015470	Decitabine	17.28	19
XYLT1	64131	Leukemia, MESH:D015470	Dexameth	17.28	13
AGO2	27161	Leukemia, MESH:D015473	Arsenic|Ar	17.27	264
CLIC4	25932	Leukemia, MESH:D015473	Arsenic Tr	17.27	262
COL3A1	1281	Leukemia, MESH:D007948	2-(2-amin	17.27	3
HAMP	57817	Leukemia, MESH:D004915	Doxorubic	17.27	4
LAMA5	3911	Leukemia, MESH:D015473	Arsenic|ars	17.27	158
MAPK10	5602	Leukemia, MESH:D015473	arsenite|D	17.27	155
PTGR1	22949	Leukemia, MESH:D015470	Air Polluta	17.27	22

STAT5	1E+08	Leukemia, MESH:D015473	Arsenic Tr	17.27	151
TUBB2B	347733	Leukemia, MESH:D015473	Arsenic|De	17.27	160
HLA-E	3133	Leukemia, MESH:D015473	Arsenic Tr	17.26	263
C2	717	Leukemia, MESH:D015470	Air Polluta	17.25	21
CLOCK	9575	Leukemia, MESH:D015470	Bezafibrat	17.25	16
CXCL16	58191	Leukemia, MESH:D015470	Arsenic Tr	17.25	27
DDIT4L	115265	Leukemia, MESH:D015470	Dronabinc	17.25	9
F2R	2149	Leukemia, MESH:D004915	Doxorubic	17.25	3
GRB14	2888	Leukemia, MESH:D015470	Dexameth	17.25	9
HCAR2	338442	Leukemia, MESH:D015470	Dexameth	17.25	11
KCNK1	3775	Leukemia, MESH:D015470	Decitabine	17.25	17
MDM4	4194	Leukemia, MESH:D015470	Arsenic Tr	17.25	41
MID1IP1	58526	Leukemia, MESH:D015470	Gasoline|N	17.25	13
PANK1	53354	Leukemia, MESH:D015470	Ifosfamide	17.25	10
PTMS	5763	Leukemia, MESH:D015470	Benzene|D	17.25	27
RANGAP1	5905	Leukemia, MESH:D015470	Benzene|D	17.25	36
RIOK3	8780	Leukemia, MESH:D015470	Dexameth	17.25	15
SAA3	20210	Leukemia, MESH:D015470	Cyclophos	17.25	13
SOX18	54345	Leukemia, MESH:D015470	Arsenic Tr	17.25	35
TBK1	29110	Leukemia, MESH:D015470	Dexameth	17.25	12
TBXT	6862	Leukemia, MESH:D015473	Arsenic|ars	17.25	156
BACE1	23621	Leukemia, MESH:D015470	Arsenic Tr	17.24	16
FBXO5	26271	Leukemia, MESH:D015473	Calcitriol|D	17.24	154
GLUD1	2746	Leukemia, MESH:D015473	arsenic dis	17.24	262
TSC2	7249	Leukemia, MESH:D015473	Arsenic|Ar	17.24	160
CD86	942	Leukemia, MESH:D007948	2-(2-amin	17.23	3
COL4A2	1284	Leukemia, MESH:D015473	Arsenic|Ca	17.23	156
DUT	1854	Leukemia, MESH:D015473	arsenite|Ca	17.23	158
ESRP1	54845	Leukemia, MESH:D015473	Arsenic Tr	17.23	261
HNRNPH1	3187	Leukemia, MESH:D015473	Arsenic Tr	17.23	260
ADGRD1	283383	Leukemia, MESH:D015473	Arsenic|De	17.22	11
CD44	960	Leukemia, MESH:D007948	Arsenic Tr	17.22	4
DNMT3L	29947	Leukemia, MESH:D015473	Arsenic|Ar	17.22	160
DYNC2I2	89891	Leukemia, MESH:D015473	Arsenic|Ar	17.22	264
HOXA10	3206	Leukemia, MESH:D015473	arsenite|Ca	17.22	155
NDUFA10	4705	Leukemia, MESH:D015473	Arsenic Tr	17.22	262
NFASC	23114	Leukemia, MESH:D015473	Arsenic|Ca	17.22	156
PXK	54899	Leukemia, MESH:D015473	Arsenic Tr	17.22	155
RGS7	6000	Leukemia, MESH:D015473	Arsenic|Ge	17.22	155
UBR2	23304	Leukemia, MESH:D015473	Arsenic|ars	17.22	157
MT1	17748	Leukemia, MESH:D015470	Dexameth	17.21	19
PLAGL2	5326	Leukemia, MESH:D015473	Arsenic Tr	17.21	262
ZIC1	7545	Leukemia, MESH:D015473	Arsenic|ars	17.21	157
GATA5	140628	Leukemia, MESH:D015470	Cytarabine	17.2	87
INSR	3643	Leukemia, MESH:D015473	Arsenic|ars	17.2	160
LDB3	11155	Leukemia, MESH:D015473	Daunorub	17.2	18
MOGAT2	80168	Leukemia, MESH:D015470	Calcitriol|D	17.2	7
PAWR	5074	Leukemia, MESH:D015473	alpha-Toc	17.2	262
TRPA1	8989	Leukemia, MESH:D015470	Benzene|C	17.2	23
UBD	10537	Leukemia, MESH:D015473	Arsenic|Ch	17.2	154
ACACA	31	Leukemia, MESH:D015473	Arsenic|Ar	17.19	266
ARHGAP1	392	Leukemia, MESH:D015470	Air Polluta	17.19	21
COL1A2	1278	Leukemia, MESH:D007948	2-(2-amin	17.19	4
CPSF2	53981	Leukemia, MESH:D015470	Air Polluta	17.19	12
CSRP1	1465	Leukemia, MESH:D015470	Arsenic Tr	17.19	21
JUNB	3726	Leukemia, MESH:D007948	2-(2-amin	17.19	3
PHLPP1	23239	Leukemia, MESH:D015470	Arsenic Tr	17.19	20
PLCXD2	257068	Leukemia, MESH:D015470	Calcitriol|I	17.19	9

RGMB	285704	Leukemia, MESH:D015470	Calcitriol|C	17.19	78
S1PR1	1901	Leukemia, MESH:D015470	Benzene|D	17.19	29
SRSF7	6432	Leukemia, MESH:D015473	Arsenic Tr	17.19	158
TUG1	55000	Leukemia, MESH:D015470	Cyclophos	17.19	12
ADAM10	102	Leukemia, MESH:D015470	Bortezomi	17.18	17
AMMECR1	9949	Leukemia, MESH:D015470	Dexameth	17.18	13
APPBP2	10513	Leukemia, MESH:D015470	Calcitriol|D	17.18	13
ARHGAP4	393	Leukemia, MESH:D015470	Calcitriol|D	17.18	13
ASCL2	430	Leukemia, MESH:D015470	Bezafibrat	17.18	9
B3GAT1	27087	Leukemia, MESH:D015470	Decitabine	17.18	14
BDH1	622	Leukemia, MESH:D015473	Arsenic Tr	17.18	261
CACHD1	57685	Leukemia, MESH:D015470	Doxorubic	17.18	12
CCDC50	152137	Leukemia, MESH:D015470	Doxorubic	17.18	13
CHCHD7	79145	Leukemia, MESH:D015470	Arsenic Tr	17.18	19
CLDN6	9074	Leukemia, MESH:D015470	Azacitidine	17.18	9
CNTN1	1272	Leukemia, MESH:D015470	Arsenic Tr	17.18	20
CPS1	1373	Leukemia, MESH:D015473	arsenite|D	17.18	154
CX3CR1	1524	Leukemia, MESH:D015470	Air Polluta	17.18	14
CYP39A1	51302	Leukemia, MESH:D015470	Arsenic Tr	17.18	14
DOCK6	57572	Leukemia, MESH:D015470	Doxorubic	17.18	15
EIF2AK2	5610	Leukemia, MESH:D015473	Arsenic Tr	17.18	263
EPG5	57724	Leukemia, MESH:D015470	Dronabino	17.18	11
FOXP4	116113	Leukemia, MESH:D015470	Cyclophos	17.18	13
GNA13	10672	Leukemia, MESH:D015470	Irinotecan	17.18	10
GPR19	2842	Leukemia, MESH:D015470	Calcitriol|C	17.18	76
HECTD2	143279	Leukemia, MESH:D015470	Bortezomi	17.18	14
HOXB2	3212	Leukemia, MESH:D015470	Dexameth	17.18	8
ISCU	23479	Leukemia, MESH:D015470	Dexameth	17.18	13
KIF5C	3800	Leukemia, MESH:D015470	Calcitriol|D	17.18	14
LIAS	11019	Leukemia, MESH:D015470	Dexameth	17.18	10
MPST	4357	Leukemia, MESH:D015470	Air Polluta	17.18	15
MRPS28	28957	Leukemia, MESH:D015470	Decitabine	17.18	16
NKX2-1	7080	Leukemia, MESH:D015470	Decitabine	17.18	17
NMNAT2	23057	Leukemia, MESH:D015470	Calcitriol|D	17.18	12
NMRK1	54981	Leukemia, MESH:D015470	Decitabine	17.18	16
OTX1	5013	Leukemia, MESH:D015470	Air Polluta	17.18	9
PIK3CB	5291	Leukemia, MESH:D015470	Arsenic Tr	17.18	20
PPM1B	5495	Leukemia, MESH:D015470	Dexameth	17.18	9
QSER1	79832	Leukemia, MESH:D015470	Dexameth	17.18	13
RCHY1	25898	Leukemia, MESH:D015470	Arsenic Tr	17.18	27
SAE1	10055	Leukemia, MESH:D015470	Arsenic Tr	17.18	19
SAP30BP	29115	Leukemia, MESH:D015470	Dexameth	17.18	12
SEC61A1	29927	Leukemia, MESH:D015470	Air Polluta	17.18	14
SERPINA3	20716	Leukemia, MESH:D015470	Dexameth	17.18	14
SERTAD1	29950	Leukemia, MESH:D015473	arsenite|C	17.18	157
SGTA	6449	Leukemia, MESH:D015470	Dexameth	17.18	13
SLC45A4	57210	Leukemia, MESH:D015470	Arsenic Tr	17.18	14
SLC46A1	113235	Leukemia, MESH:D015470	Calcitriol|D	17.18	22
STEAP1	26872	Leukemia, MESH:D015470	Dasatinib|	17.18	14
STK40	83931	Leukemia, MESH:D015470	Benzene|D	17.18	26
STON1	11037	Leukemia, MESH:D015470	Calcitriol|C	17.18	76
SUGT1	10910	Leukemia, MESH:D015470	Arsenic Tr	17.18	18
SUMF1	285362	Leukemia, MESH:D015470	Dexameth	17.18	11
TP53BP2	7159	Leukemia, MESH:D015470	Dexameth	17.18	9
TRIP6	7205	Leukemia, MESH:D015470	Doxorubic	17.18	13
TTN	7273	Leukemia, MESH:D015470	Air Polluta	17.18	14
WDR72	256764	Leukemia, MESH:D015470	Calcitriol|D	17.18	16
CLPTM1	1209	Leukemia, MESH:D015473	Antimony	17.17	6

FAM53B	9679	Leukemia, MESH:D015470	Doxorubic	17.17	13
LMO3	55885	Leukemia, MESH:D015470	Dexameth	17.17	8
MANSC1	54682	Leukemia, MESH:D015470	Doxorubic	17.17	14
SNHG12	85028	Leukemia, MESH:D015473	Arsenic|ar:	17.17	10
AGAP2	116986	Leukemia, MESH:D015473	Arsenic|ca	17.15	155
APEX1	328	Leukemia, MESH:D004915	Daunorub	17.15	3
CCL13	6357	Leukemia, MESH:D015470	Dexameth	17.15	7
CSNK1G1	53944	Leukemia, MESH:D015473	Dexameth	17.15	8
MIR210	406992	Leukemia, MESH:D015473	Arsenic Tr	17.15	260
ZFPM2	23414	Leukemia, MESH:D015473	Arsenic Tr	17.15	263
ACADVL	37	Leukemia, MESH:D015473	Arsenic|De	17.14	157
ARC	23237	Leukemia, MESH:D015470	Arsenic Tr	17.14	17
CAMK2B	816	Leukemia, MESH:D015470	Doxorubic	17.14	15
CLIP2	7461	Leukemia, MESH:D015473	arsenite|D	17.14	153
DNAH1	25981	Leukemia, MESH:D015473	Arsenic|ar:	17.14	11
IGH	3492	Leukemia, MESH:D015470	Dasatinib|I	17.14	15
ILF2	3608	Leukemia, MESH:D015473	arsenite|G	17.14	156
MRPL33	9553	Leukemia, MESH:D015473	arsenite|D	17.14	8
PSMB8	5696	Leukemia, MESH:D015473	Arsenic Tr	17.14	261
PUF60	22827	Leukemia, MESH:D015473	Arsenic|Ar	17.14	161
STX12	23673	Leukemia, MESH:D015473	Arsenic|De	17.14	156
TMEM91	641649	Leukemia, MESH:D015470	Calcitriol|D	17.14	15
USP53	54532	Leukemia, MESH:D015473	arsenite|Ca	17.14	155
COQ7	10229	Leukemia, MESH:D015473	Dexameth	17.13	155
RBFOX2	23543	Leukemia, MESH:D015473	arsenite|D	17.13	155
RPS26L	393715	Leukemia, MESH:D015470	Dexameth	17.13	10
SDF2L1	23753	Leukemia, MESH:D015473	Arsenic Tr	17.13	263
SLC32A1	140679	Leukemia, MESH:D015473	Arsenic|ar:	17.13	157
TJP2	9414	Leukemia, MESH:D015473	Arsenic Tr	17.13	263
VPS41	27072	Leukemia, MESH:D015473	Arsenic Tr	17.13	262
ZC3H11A	9877	Leukemia, MESH:D015473	Arsenic Tr	17.13	262
DICER1	23405	Leukemia, MESH:D015470	Clioquinol	17.12	13
FUT3	2525	Leukemia, MESH:D015470	Arsenic Tr	17.12	20
GPX4	2879	Leukemia, MESH:D015473	alpha-Toc	17.12	264
MIR137	406928	Leukemia, MESH:D015470	Air Polluta	17.12	8
MIR339	442907	Leukemia, MESH:D015473	Buthionine	17.12	155
TCF24	1E+08	Leukemia, MESH:D015470	Arsenic Tr	17.12	14
TUBGCP4	27229	Leukemia, MESH:D015470	Doxorubic	17.12	12
ALDH3B1	221	Leukemia, MESH:D015470	Arsenic Tr	17.11	20
C4ORF46	201725	Leukemia, MESH:D015470	Calcitriol|D	17.11	13
CLTCL1	8218	Leukemia, MESH:D015470	Calcitriol|D	17.11	8
CRISP2	7180	Leukemia, MESH:D015470	Decitabine	17.11	17
HBD	3045	Leukemia, MESH:D015470	(+)-JQ1 co	17.11	10
KARS1	3735	Leukemia, MESH:D015470	Doxorubic	17.11	11
PDCD6IP	10015	Leukemia, MESH:D015470	Calcitriol|D	17.11	11
PFN2	5217	Leukemia, MESH:D015470	Arsenic Tr	17.11	15
PGM3	5238	Leukemia, MESH:D015470	Arsenic Tr	17.11	14
PNPLA2	57104	Leukemia, MESH:D015473	Dexameth	17.11	9
SAMD10	140700	Leukemia, MESH:D015470	Bortezomi	17.11	12
SLC22A8	9376	Leukemia, MESH:D015470	Allopurino	17.11	16
SNX22	79856	Leukemia, MESH:D015470	Decitabine	17.11	14
STX3	6809	Leukemia, MESH:D015470	Benzene|D	17.11	35
ASAH1	427	Leukemia, MESH:D015473	Arsenic Tr	17.1	261
BABAM1	29086	Leukemia, MESH:D015470	Arsenic Tr	17.1	18
IFI27	3429	Leukemia, MESH:D015473	Calcitriol|D	17.1	153
KIRREL1	55243	Leukemia, MESH:D015470	Doxorubic	17.1	13
ZNF286A	57335	Leukemia, MESH:D015470	Doxorubic	17.1	14
ZNF710	374655	Leukemia, MESH:D015470	Doxorubic	17.1	12

ATP5G2	67942	Leukemia, MESH:D015473	Arsenic Tr	17.09	261
CPLX1	10815	Leukemia, MESH:D015470	Azacitidine	17.09	9
CRADD	8738	Leukemia, MESH:D015470	Benzene|D	17.09	20
CRYBG2	55057	Leukemia, MESH:D015470	Decitabine	17.09	16
CYP7A1	1581	Leukemia, MESH:D007948	2-(2-amin	17.09	2
IBTK	25998	Leukemia, MESH:D015470	Bortezomi	17.09	15
KRT19	3880	Leukemia, MESH:D004915	Cytarabine	17.09	2
NELFA	7469	Leukemia, MESH:D015470	alvocidib|D	17.09	9
PHB1	5245	Leukemia, MESH:D015473	Arsenic Tr	17.09	263
SH3TC1	54436	Leukemia, MESH:D015470	Calcitriol|C	17.09	7
SLAMF7	57823	Leukemia, MESH:D015470	Air Polluta	17.09	13
SLC47A1	55244	Leukemia, MESH:D015470	Chloramph	17.09	82
USP18	11274	Leukemia, MESH:D015473	Arsenic|De	17.09	158
ABCB10	23456	Leukemia, MESH:D015470	Calcitriol|D	17.08	14
AGPAT5	55326	Leukemia, MESH:D015473	Arsenic Tr	17.08	262
ARFIP1	27236	Leukemia, MESH:D015470	Air Polluta	17.08	16
ARHGAP1	84986	Leukemia, MESH:D015470	Arsenic Tr	17.08	28
CERS4	79603	Leukemia, MESH:D015473	Arsenic|Ar	17.08	160
CNMD	11061	Leukemia, MESH:D015470	Arsenic Tr	17.08	16
COL4A4	1286	Leukemia, MESH:D015470	Doxorubic	17.08	14
CSGALNA	55454	Leukemia, MESH:D015470	Dexameth	17.08	8
CTDSPL2	51496	Leukemia, MESH:D015470	Bortezomi	17.08	15
DERL1	79139	Leukemia, MESH:D015470	Dexameth	17.08	15
EPB41L4A	64097	Leukemia, MESH:D015470	Dronabinc	17.08	9
FMN1	342184	Leukemia, MESH:D015470	Chloroqui	17.08	7
HOXB5	3215	Leukemia, MESH:D015470	Alitretinoi	17.08	8
LGALS7	3963	Leukemia, MESH:D015470	Cyclophos	17.08	15
S100A2	6273	Leukemia, MESH:D015470	Arsenic Tr	17.08	21
SAMM50	25813	Leukemia, MESH:D015470	Decitabine	17.08	15
SELENOK	58515	Leukemia, MESH:D015470	Air Polluta	17.08	13
SLC4A11	83959	Leukemia, MESH:D015473	arsenite|Ca	17.08	154
SRPRA	6734	Leukemia, MESH:D015470	Methotrex	17.08	9
TPK1	27010	Leukemia, MESH:D015470	Cytarabine	17.08	80
TUB	7275	Leukemia, MESH:D015470	Calcitriol|D	17.08	16
UBE2G2	7327	Leukemia, MESH:D015470	Ifosfamide	17.08	11
USP12	219333	Leukemia, MESH:D015470	Doxorubic	17.08	17
WAPL	23063	Leukemia, MESH:D015470	Arsenic Tr	17.08	16
YIPF5	81555	Leukemia, MESH:D015470	Doxorubic	17.08	13
ZBED1	9189	Leukemia, MESH:D004915	Doxorubic	17.08	3
ACTR2	10097	Leukemia, MESH:D015473	Dexameth	17.07	153
AMOT	154796	Leukemia, MESH:D015470	Arsenic Tr	17.07	18
CIB2	10518	Leukemia, MESH:D015470	Dexameth	17.07	9
CORO2B	10391	Leukemia, MESH:D015470	Dexameth	17.07	8
ETV3	2117	Leukemia, MESH:D015470	Arsenic Tr	17.07	18
GNA11	2767	Leukemia, MESH:D015473	Arsenic|Ar	17.07	161
MAML2	84441	Leukemia, MESH:D015470	Doxorubic	17.07	12
MLLT10	8028	Leukemia, MESH:D015473	Arsenic|Ar	17.07	261
NECTIN4	81607	Leukemia, MESH:D015473	Arsenic|ars	17.07	11
OGDHL	55753	Leukemia, MESH:D015470	Doxorubic	17.07	14
PES1	23481	Leukemia, MESH:D015470	Dexameth	17.07	14
PMPCB	9512	Leukemia, MESH:D015470	Arsenic Tr	17.07	20
RASA2	5922	Leukemia, MESH:D015470	Doxorubic	17.07	14
RCOR1	23186	Leukemia, MESH:D015470	Doxorubic	17.07	14
RGS20	8601	Leukemia, MESH:D015473	arsenite|Cy	17.07	157
SEC22B	9554	Leukemia, MESH:D015470	Dexameth	17.07	14
SH3RF3	344558	Leukemia, MESH:D015470	Doxorubic	17.07	13
STN1	79991	Leukemia, MESH:D015470	Dexameth	17.07	13
TRAPPC9	83696	Leukemia, MESH:D015470	Dexameth	17.07	12

TTC30B	150737	Leukemia, MESH:D015470	Dexameth	17.07	8
VANGL2	57216	Leukemia, MESH:D015470	Doxorubic	17.07	14
VEGFA	7422	Leukemia, MESH:D007948	2-(2-amin	17.07	3
VRK2	7444	Leukemia, MESH:D015470	Cytarabine	17.07	80
ZNF292	23036	Leukemia, MESH:D015470	Dexameth	17.07	10
ADCY4	196883	Leukemia, MESH:D015473	arsenite|D	17.06	155
CCDC88C	440193	Leukemia, MESH:D015473	Arsenic|Ca	17.06	156
CCL26	10344	Leukemia, MESH:D015473	Arsenic|ars	17.06	13
CREB3L1	90993	Leukemia, MESH:D015473	Arsenic|Ca	17.06	156
ELP4	26610	Leukemia, MESH:D015473	Calcitriol|D	17.06	153
GCLC	2729	Leukemia, MESH:D004915	Doxorubic	17.06	4
LOX	4015	Leukemia, MESH:D015473	Arsenic|ars	17.06	161
LRRC17	10234	Leukemia, MESH:D015473	arsenite|D	17.06	154
PDGFB	5155	Leukemia, MESH:D015473	arsenite|G	17.06	156
PDSS1	23590	Leukemia, MESH:D015473	arsenite|Ca	17.06	157
TMEM64	169200	Leukemia, MESH:D015473	Dexameth	17.06	155
ULBP1	80329	Leukemia, MESH:D015473	Arsenic Tr	17.06	263
AMPD3	272	Leukemia, MESH:D015473	Calcitriol|D	17.05	154
CAMK2N1	55450	Leukemia, MESH:D015470	Cytarabine	17.05	85
CCKBR	887	Leukemia, MESH:D015470	Cytarabine	17.05	78
CTTN	2017	Leukemia, MESH:D015473	Arsenic|Ar	17.05	266
FCGR2C	9103	Leukemia, MESH:D015470	Cytarabine	17.05	74
GABARAP	11337	Leukemia, MESH:D015473	arsenite|D	17.05	157
GSTM4	2948	Leukemia, MESH:D015473	Arsenic|Ar	17.05	263
HTATSF1	27336	Leukemia, MESH:D015473	Arsenic|Ar	17.05	263
MYBL1	4603	Leukemia, MESH:D015473	Arsenic Tr	17.05	260
RAC2	5880	Leukemia, MESH:D015473	Arsenic|Ar	17.05	265
TUBA1C	84790	Leukemia, MESH:D015473	arsenite|D	17.05	158
ATP7B	540	Leukemia, MESH:D015470	Carboplati	17.04	82
CA3	761	Leukemia, MESH:D015470	Arsenic Tr	17.04	14
GAL	51083	Leukemia, MESH:D015470	Calcitriol|C	17.04	15
GINS1	9837	Leukemia, MESH:D015470	Calcitriol|D	17.04	11
GNE	10020	Leukemia, MESH:D015470	Arsenic Tr	17.04	22
MMP8	4317	Leukemia, MESH:D015470	Calcitriol|C	17.04	16
POU5F1P4	645682	Leukemia, MESH:D015470	Cytarabine	17.04	74
PPM1G	5496	Leukemia, MESH:D015473	Arsenic|Bu	17.04	156
PSMC2	5701	Leukemia, MESH:D015470	Arsenic Tr	17.04	19
RAB6C	84084	Leukemia, MESH:D015470	Decitabine	17.04	18
VGF	7425	Leukemia, MESH:D015470	Indometha	17.04	12
SOD2	6648	Leukemia, MESH:D007948	2-(2-amin	17.03	3
GIMAP2	26157	Leukemia, MESH:D015470	Calcitriol|N	17.02	7
IFITM4P	340198	Leukemia, MESH:D015470	Dexameth	17.02	4
NQO1	1728	Leukemia, MESH:D007948	2-(2-amin	17.02	3
SOX19A	30038	Leukemia, MESH:D015470	Dexameth	17.02	5
TMSB	402820	Leukemia, MESH:D015470	Pentachlo	17.02	6
TYMP	1890	Leukemia, MESH:D015473	Arsenic Tr	17.02	157
AK5	26289	Leukemia, MESH:D015473	Calcitriol|D	17.01	152
ECI1	1632	Leukemia, MESH:D015473	Arsenic|Ar	17.01	268
H3-4	8290	Leukemia, MESH:D007948	2-(2-amin	17.01	2
NEFM	4741	Leukemia, MESH:D015473	Arsenic|ars	17.01	157
P2RX5	5026	Leukemia, MESH:D015473	Genistein|s	17.01	157
PCOTH	542767	Leukemia, MESH:D015470	(+)-JQ1 cc	17.01	7
RHOC	389	Leukemia, MESH:D015473	Arsenic Tr	17.01	264
SELENOW	378438	Leukemia, MESH:D015470	Dexameth	17.01	5
SELL	6402	Leukemia, MESH:D015473	Genistein|I	17.01	158
SMAD2	4087	Leukemia, MESH:D004915	Doxorubic	17.01	3
DCAF4L1	285429	Leukemia, MESH:D015470	Dronabinc	17	5
ELK1	2002	Leukemia, MESH:D015473	Arsenic Tr	17	156

EPHB1	2047	Leukemia, MESH:D015470		Decitabine	17	18
GRIK3	2899	Leukemia, MESH:D015473		Arsenic|Ar	17	160
NCL	4691	Leukemia, MESH:D015473		Arsenic|Ar	17	262
ZNF28	7576	Leukemia, MESH:D015470		Arsenic Tr	17	16
ACTG1	71	Leukemia, MESH:D015470		Arsenic Tr	16.99	23
ANKRD33	651746	Leukemia, MESH:D015473		Arsenic|De	16.99	156
BTBD3	22903	Leukemia, MESH:D015470		Arsenic Tr	16.99	25
C16ORF54	283897	Leukemia, MESH:D015470		Dronabino	16.99	6
CDH8	1006	Leukemia, MESH:D015473		Arsenic Tr	16.99	156
ELANE	1991	Leukemia, MESH:D015470		Deferoxan	16.99	9
GDF9	2661	Leukemia, MESH:D015470		Azacitidine	16.99	11
HACD2	201562	Leukemia, MESH:D015473		Arsenic|Ca	16.99	157
PROS1	5627	Leukemia, MESH:D015470		Decitabine	16.99	24
SCUBE2	57758	Leukemia, MESH:D015473		Arsenic|Ar	16.99	159
SH3GL3	6457	Leukemia, MESH:D015473		Arsenic|ars	16.99	156
UGGT2	55757	Leukemia, MESH:D015473		Arsenic|De	16.99	156
VPS13B	157680	Leukemia, MESH:D015473		Arsenic|De	16.99	156
DLEU2	8847	Leukemia, MESH:D01	marker/me	Arsenic Tr	16.98	20
ALOX15	246	Leukemia, MESH:D015473		alpha-Toc	16.98	155
CABIN1	23523	Leukemia, MESH:D015470		Arsenic Tr	16.98	19
DNAJC6	9829	Leukemia, MESH:D015473		Arsenic|ars	16.98	11
DPM3	54344	Leukemia, MESH:D015470		Dexameth	16.98	13
DROSHA	29102	Leukemia, MESH:D015473		Arsenic|ars	16.98	157
ERCC3	2071	Leukemia, MESH:D015473		Arsenic|Ge	16.98	157
EVI5	7813	Leukemia, MESH:D015470		Calcitriol|(	16.98	18
FASTKD2	22868	Leukemia, MESH:D015470		Dexameth	16.98	10
FER	2241	Leukemia, MESH:D015470		Arsenic Tr	16.98	17
FGFR1OP2	26127	Leukemia, MESH:D015470		Arsenic Tr	16.98	14
GAK	2580	Leukemia, MESH:D015473		Arsenic|Ar	16.98	263
GEMIN6	79833	Leukemia, MESH:D015473		arsenite|D	16.98	155
GP1BB	2812	Leukemia, MESH:D015470		Benzene|(	16.98	24
HGS	9146	Leukemia, MESH:D015470		Dexameth	16.98	14
LUZP1	7798	Leukemia, MESH:D015470		Doxorubic	16.98	13
MPC2	25874	Leukemia, MESH:D015470		Cyclophos	16.98	21
OTUD7B	56957	Leukemia, MESH:D015470		Calcitriol|D	16.98	15
PFDN2	5202	Leukemia, MESH:D015470		Benzene|D	16.98	21
PHC3	80012	Leukemia, MESH:D015470		Bortezomi	16.98	14
PI4KA	5297	Leukemia, MESH:D015473		Arsenic|Ar	16.98	160
PRSS1	5644	Leukemia, MESH:D015470		Arsenic Tr	16.98	16
SEM1	7979	Leukemia, MESH:D015470		Arsenic Tr	16.98	14
SF3A2	8175	Leukemia, MESH:D015470		(+)-JQ1 co	16.98	9
SLC8B1	80024	Leukemia, MESH:D015470		Ethylnitros	16.98	11
SNRPD3	6634	Leukemia, MESH:D015473		Arsenic Tr	16.98	156
SRSF4	6429	Leukemia, MESH:D015470		Air Polluta	16.98	14
SSBP3	23648	Leukemia, MESH:D015473		Arsenic Tr	16.98	262
TIMELESS	8914	Leukemia, MESH:D015470		Arsenic Tr	16.98	17
IDH2	3418	Leukemia, MESH:D01	marker/me	Arsenic|Ar	16.97	267
ABCD3	5825	Leukemia, MESH:D015473		alpha-Toc	16.97	154
ARHGEF1	9138	Leukemia, MESH:D015470		Dexameth	16.97	13
CBR4	84869	Leukemia, MESH:D015470		Arsenic Tr	16.97	25
CHCHD2	51142	Leukemia, MESH:D015470		Doxorubic	16.97	16
CLDN12	9069	Leukemia, MESH:D015470		Calcitriol|D	16.97	13
COMMD3	23412	Leukemia, MESH:D015470		Decitabine	16.97	17
CYP2C29	13095	Leukemia, MESH:D015473		alpha-Toc	16.97	155
FSHB	2488	Leukemia, MESH:D007948		2-(2-amin	16.97	2
GJA3	2700	Leukemia, MESH:D015470		Doxorubic	16.97	14
GMPR2	51292	Leukemia, MESH:D015470		Dexameth	16.97	11
HNRNPA2	3181	Leukemia, MESH:D015473		Arsenic Tr	16.97	262

KIF3C	3797	Leukemia, MESH:D015470	Calcitriol|[illegible]	16.97	13
LIN7C	55327	Leukemia, MESH:D015470	Arsenic Tr	16.97	18
METTL1	4234	Leukemia, MESH:D015470	Arsenic Tr	16.97	19
MTA2	9219	Leukemia, MESH:D015470	Dexameth	16.97	7
NXPE3	91775	Leukemia, MESH:D015470	Bortezomi	16.97	14
PPP1CA	5499	Leukemia, MESH:D015470	Arsenic Tr	16.97	21
PPP3R1	5534	Leukemia, MESH:D015470	Bortezomi	16.97	14
PPTC7	160760	Leukemia, MESH:D015470	Doxorubic	16.97	13
PRMT6	55170	Leukemia, MESH:D015470	Pentachlo	16.97	10
RAB3C	115827	Leukemia, MESH:D015470	Dexameth	16.97	8
SH3BP5	9467	Leukemia, MESH:D015470	Dexameth	16.97	10
STRN	6801	Leukemia, MESH:D015470	Doxorubic	16.97	13
TEX2	55852	Leukemia, MESH:D015470	Dexameth	16.97	8
TFAP2B	7021	Leukemia, MESH:D015470	Dexameth	16.97	8
TP53	7157	Leukemia, MESH:D004915	Cytarabine	16.97	3
TUBE1	51175	Leukemia, MESH:D015470	Doxorubic	16.97	13
UTP4	84916	Leukemia, MESH:D015470	Dexameth	16.97	10
VPS29	51699	Leukemia, MESH:D015470	Doxorubic	16.97	13
ABCA9	10350	Leukemia, MESH:D015470	Arsenic Tr	16.96	18
FUS	2521	Leukemia, MESH:D015473	Arsenic|Ca	16.96	156
LTB	4050	Leukemia, MESH:D015473	Arsenic|Ar	16.96	264
BCL2A1A	12044	Leukemia, MESH:D015473	Arsenic Tr	16.95	262
RAD9B	144715	Leukemia, MESH:D015470	Dronabino	16.95	10
XDH	7498	Leukemia, MESH:D015473	Arsenic|ars	16.95	160
ABAT	18	Leukemia, MESH:D015470	Calcitriol|[illegible]	16.94	16
CDKN2A	1029	Leukemia, MESH:D007948	Arsenic Tr	16.94	3
CMIP	80790	Leukemia, MESH:D015473	Arsenic Tr	16.94	263
CRYGS	1427	Leukemia, MESH:D015470	Alitretinoi	16.94	7
HHLA3	11147	Leukemia, MESH:D015470	Arsenic Tr	16.94	20
ATPAF2	91647	Leukemia, MESH:D015470	Doxorubic	16.93	13
CIPC	85457	Leukemia, MESH:D015470	Doxorubic	16.93	12
EXD3	54932	Leukemia, MESH:D015470	Dexameth	16.93	11
IRX1	79192	Leukemia, MESH:D015473	arsenite|G	16.93	156
KIAA0513	9764	Leukemia, MESH:D015470	Calcitriol|(	16.93	7
LCE1B	353132	Leukemia, MESH:D015473	Arsenic|ars	16.93	155
METTL13	51603	Leukemia, MESH:D015470	Air Polluta	16.93	11
PITPNB	23760	Leukemia, MESH:D015473	Dexameth	16.93	157
PLEKHG3	26030	Leukemia, MESH:D015473	Arsenic|Ar	16.93	165
PVRL1	1E+08	Leukemia, MESH:D015470	Arsenic Tr	16.93	19
S100A16	140576	Leukemia, MESH:D015473	Genistein|[illegible]	16.93	154
TMEM198	440104	Leukemia, MESH:D015470	Dexameth	16.93	6
TRRAP	8295	Leukemia, MESH:D015473	Arsenic|Ar	16.93	267
VGLL4	9686	Leukemia, MESH:D015473	Arsenic|Ar	16.93	166
C2ORF68	388969	Leukemia, MESH:D015470	Dexameth	16.92	6
CA11	770	Leukemia, MESH:D015470	Doxorubic	16.92	12
CCDC177	56936	Leukemia, MESH:D015470	Dexameth	16.92	7
CHMP3	51652	Leukemia, MESH:D015470	Doxorubic	16.92	11
DENND11	57189	Leukemia, MESH:D015470	Doxorubic	16.92	12
LIF	3976	Leukemia, MESH:D004915	Doxorubic	16.92	3
MCOLN3	55283	Leukemia, MESH:D015473	Arsenic Tr	16.92	263
NCAPD3	23310	Leukemia, MESH:D015473	arsenite|D	16.92	154
OTOGL	283310	Leukemia, MESH:D015470	Dexameth	16.92	11
PSTPIP2	9050	Leukemia, MESH:D015473	Cytarabine	16.92	155
RAB4A	5867	Leukemia, MESH:D015473	arsenite|G	16.92	158
SERHL	94009	Leukemia, MESH:D015473	Calcitriol|C	16.92	6
SNURF	8926	Leukemia, MESH:D015470	Dexameth	16.92	13
UMPS	7372	Leukemia, MESH:D015470	Benzoates	16.92	11
CCN5	8839	Leukemia, MESH:D015470	Arsenic Tr	16.91	19

GPI	2821	Leukemia, MESH:D015470	Arsenic Tr	16.91	16
LIN7A	8825	Leukemia, MESH:D015470	Calcitriol|D	16.91	17
NLRC5	84166	Leukemia, MESH:D015473	Arsenic|Ca	16.91	10
PABPC1	26986	Leukemia, MESH:D015470	Dexameth	16.91	21
PLAC8	51316	Leukemia, MESH:D015470	Doxorubic	16.91	16
PRKAG1	5571	Leukemia, MESH:D015473	Arsenic|ars	16.91	158
RRS1	23212	Leukemia, MESH:D015470	Dexameth	16.91	8
SAC3D1	29901	Leukemia, MESH:D015473	arsenite|D	16.91	8
STING1	340061	Leukemia, MESH:D015473	Arsenic|ars	16.91	11
STRN3	29966	Leukemia, MESH:D015473	Arsenic Tr	16.91	156
UBE2D2	7322	Leukemia, MESH:D015473	Arsenic|Ar	16.91	265
GBA	2629	Leukemia, MESH:D015473	Arsenic Tr	16.9	261
CIDEC	63924	Leukemia, MESH:D015470	15-deoxy-	16.89	10
CTPS	51797	Leukemia, MESH:D015470	Air Polluta	16.89	9
HSP90AA1	30591	Leukemia, MESH:D015473	Arsenic|Ar	16.89	158
PDS5B	23047	Leukemia, MESH:D015470	Androgen	16.89	11
TNFAIP2	7127	Leukemia, MESH:D015473	Calcitriol|N	16.89	156
ANLN	54443	Leukemia, MESH:D015473	arsenite|Ca	16.88	10
BET1	10282	Leukemia, MESH:D015470	Benzene|P	16.88	24
BLMH	642	Leukemia, MESH:D015470	Carboplati	16.88	14
CDCA4	55038	Leukemia, MESH:D015470	Calcitriol|D	16.88	9
CHRND	1144	Leukemia, MESH:D015470	Dexameth	16.88	7
CYFIP1	23191	Leukemia, MESH:D015470	Benzoates	16.88	13
DTNB	1838	Leukemia, MESH:D015470	Bezafibrat	16.88	9
GRHL3	57822	Leukemia, MESH:D015470	Calcitriol|D	16.88	15
MCCC1	56922	Leukemia, MESH:D015470	Dexameth	16.88	13
MIR181D	574457	Leukemia, MESH:D015473	Arsenic Tr	16.88	260
MIR19A	406979	Leukemia, MESH:D015470	Arsenic Tr	16.88	30
OPA1	4976	Leukemia, MESH:D015473	Arsenic Tr	16.88	157
OSBPL2	9885	Leukemia, MESH:D015470	Bortezomi	16.88	16
PELI2	57161	Leukemia, MESH:D015470	Doxorubic	16.88	16
POU2AF1	5450	Leukemia, MESH:D015470	Calcitriol|(	16.88	11
PRTN3	5657	Leukemia, MESH:D015470	Arsenic Tr	16.88	15
RAC3	5881	Leukemia, MESH:D015470	Doxorubic	16.88	14
SHROOM1	357	Leukemia, MESH:D015470	Calcitriol|D	16.88	8
SLC8A1	6546	Leukemia, MESH:D015473	Arsenic|Ar	16.88	160
TNFAIP1	7126	Leukemia, MESH:D015470	Air Polluta	16.88	8
VPS53	55275	Leukemia, MESH:D015470	Arsenic Tr	16.88	18
ADCY8	114	Leukemia, MESH:D015470	Dexameth	16.87	12
CUTC	51076	Leukemia, MESH:D015470	Dexameth	16.87	7
DGKI	9162	Leukemia, MESH:D015470	Arsenic Tr	16.87	18
DISC1	27185	Leukemia, MESH:D015470	Decitabine	16.87	19
EFNB3	1949	Leukemia, MESH:D015470	Decitabine	16.87	20
FAM110B	90362	Leukemia, MESH:D015470	Arsenic Tr	16.87	15
FOS	2353	Leukemia, MESH:D007948	2-(2-amin	16.87	3
IFNG	3458	Leukemia, MESH:D007948	2-(2-amin	16.87	3
IGSF10	285313	Leukemia, MESH:D015470	Dexameth	16.87	7
KDSR	2531	Leukemia, MESH:D015470	Doxorubic	16.87	16
LRP12	29967	Leukemia, MESH:D015470	Air Polluta	16.87	12
MAP4K5	11183	Leukemia, MESH:D015470	Doxorubic	16.87	14
MIR145	406937	Leukemia, MESH:D015470	Decitabine	16.87	16
NAPG	8774	Leukemia, MESH:D015470	Arsenic Tr	16.87	19
POLR3H	171568	Leukemia, MESH:D015470	Bortezomi	16.87	13
RAB25	57111	Leukemia, MESH:D015470	Arsenic Tr	16.87	13
RAB43	339122	Leukemia, MESH:D015470	Methotrex	16.87	13
RASSF8	11228	Leukemia, MESH:D015470	Arsenic Tr	16.87	14
SCFD2	152579	Leukemia, MESH:D015470	Dexameth	16.87	12
SPNS2	124976	Leukemia, MESH:D015470	Dronabinc	16.87	10

SYNGR3	9143	Leukemia, MESH:D015470	Calcitriol|D	16.87	14
TGFB1	7040	Leukemia, MESH:D007948	2-(2-amin	16.87	3
UIMC1	51720	Leukemia, MESH:D015470	Arsenic Tr	16.87	18
PDE8A	5151	Leukemia, MESH:D015473	Arsenic|Ar	16.86	265
CXCR5	643	Leukemia, MESH:D015473	Arsenic|Ge	16.85	159
CYP1A1	1543	Leukemia, MESH:D004915	Daunorub	16.85	4
DEGS1	8560	Leukemia, MESH:D015473	Arsenic|ca	16.85	155
HSD17B7	51478	Leukemia, MESH:D015470	Androgen	16.85	12
MUC5AC	4586	Leukemia, MESH:D015470	Air Polluta	16.85	13
NGEF	25791	Leukemia, MESH:D015470	Daunorub	16.85	36
BBOX1	8424	Leukemia, MESH:D015470	Dexameth	16.84	16
CCL6	20305	Leukemia, MESH:D015473	arsenite|D	16.84	13
COX5A	9377	Leukemia, MESH:D015470	Arsenic Tr	16.84	28
CRK	1398	Leukemia, MESH:D015470	Benzene|D	16.84	22
GSK3A	2931	Leukemia, MESH:D015470	Bortezomi	16.84	20
GSTA1	2938	Leukemia, MESH:D015473	Arsenic Tr	16.84	157
HNRNPC	3183	Leukemia, MESH:D015470	Doxorubic	16.84	16
HRK	8739	Leukemia, MESH:D015470	Arsenic Tr	16.84	26
KLKB1	3818	Leukemia, MESH:D015470	Air Polluta	16.84	18
LRATD2	157638	Leukemia, MESH:D015473	arsenite|Ca	16.84	154
NOL6	65083	Leukemia, MESH:D015473	Arsenic Tr	16.84	154
NT5DC2	64943	Leukemia, MESH:D015470	Air Polluta	16.84	15
PEBP1	5037	Leukemia, MESH:D015473	arsenite|D	16.84	157
PFKM	5213	Leukemia, MESH:D015470	Daunorub	16.84	40
PPP2R3A	5523	Leukemia, MESH:D015473	Arsenic|ars	16.84	11
SCNN1A	6337	Leukemia, MESH:D015470	Calcitriol|D	16.84	16
SNX5	27131	Leukemia, MESH:D015473	Dexameth	16.84	156
SRRM2	23524	Leukemia, MESH:D015470	Dexameth	16.84	11
SUCLG2	8801	Leukemia, MESH:D015470	Arsenic Tr	16.84	23
HAS1	3036	Leukemia, MESH:D015473	arsenite|D	16.83	8
IL15	3600	Leukemia, MESH:D015473	Arsenic Tr	16.83	261
KLF6	1316	Leukemia, MESH:D004915	Doxorubic	16.83	3
MATK	4145	Leukemia, MESH:D015473	Arsenic|Ar	16.83	263
RYR3	6263	Leukemia, MESH:D015473	Arsenic|Ar	16.83	265
SHH	6469	Leukemia, MESH:D015473	Arsenic Tr	16.83	261
SOX13	9580	Leukemia, MESH:D015473	Arsenic Tr	16.83	261
ZFYVE16	9765	Leukemia, MESH:D015473	arsenic dis	16.81	156
HTATIP2	10553	Leukemia, MESH:D015473	Antimony	16.8	262
RHOA	387	Leukemia, MESH:D015473	Arsenic Tr	16.8	261
BPNT1	10380	Leukemia, MESH:D015470	Air Polluta	16.79	7
C4ORF3	401152	Leukemia, MESH:D015470	Bortezomi	16.79	13
COL12A1	1303	Leukemia, MESH:D015473	arsenite|D	16.79	157
GJA1	2697	Leukemia, MESH:D007948	2-(2-amin	16.79	3
GSTK1	373156	Leukemia, MESH:D015473	Arsenic|ca	16.79	12
IRAG1	10335	Leukemia, MESH:D015470	Calcitriol|D	16.79	8
MIR125A	406910	Leukemia, MESH:D015470	Air Polluta	16.79	30
MIR149	406941	Leukemia, MESH:D015473	Arsenic|Ar	16.79	158
NTRK1	4914	Leukemia, MESH:D015473	Arsenic|Ar	16.79	263
ROCK2	9475	Leukemia, MESH:D015473	Arsenic|ars	16.79	13
SOHLH1	402381	Leukemia, MESH:D015473	Arsenic|Et	16.79	152
APLN	8862	Leukemia, MESH:D015470	Dexameth	16.78	15
ASXL1	171023	Leukemia, MESH:D015470	Benzene|D	16.78	30
CAB39L	81617	Leukemia, MESH:D015473	Arsenic Tr	16.78	165
CCND1	595	Leukemia, MESH:D007948	2-(2-amin	16.78	3
CCNO	10309	Leukemia, MESH:D015470	Arsenic Tr	16.78	20
CLASP2	23122	Leukemia, MESH:D015470	Doxorubic	16.78	17
DYNC1LI2	1783	Leukemia, MESH:D015473	arsenite|M	16.78	157
GREM2	64388	Leukemia, MESH:D015473	Dasatinib|	16.78	151

INCENP	3619	Leukemia, MESH:D015470	Calcitriol|D	16.78	16
MAB21L2	10586	Leukemia, MESH:D015470	Cytarabine	16.78	81
MIA	8190	Leukemia, MESH:D015470	Decitabine	16.78	15
MTFP1	51537	Leukemia, MESH:D015470	Doxorubic	16.78	13
NDUFB10	4716	Leukemia, MESH:D015470	Doxorubic	16.78	12
OLIG2	10215	Leukemia, MESH:D015470	Arsenic Tr	16.78	19
PPP4R3B	57223	Leukemia, MESH:D015470	Doxorubic	16.78	15
SNX25	83891	Leukemia, MESH:D015470	Calcitriol|D	16.78	16
STK17A	9263	Leukemia, MESH:D015473	Arsenic Tr	16.78	259
THSD7A	221981	Leukemia, MESH:D015470	Decitabine	16.78	14
TRPM4	54795	Leukemia, MESH:D015470	Arsenic Tr	16.78	26
TWIST2	117581	Leukemia, MESH:D015470	Benzene|D	16.78	27
ZNRF1	84937	Leukemia, MESH:D015470	Dexameth	16.78	8
APOM	55937	Leukemia, MESH:D015470	Dexameth	16.77	11
ATP6V1G1	9550	Leukemia, MESH:D015470	Arsenic Tr	16.77	18
CAPRIN1	4076	Leukemia, MESH:D015470	Doxorubic	16.77	15
CCDC69	26112	Leukemia, MESH:D015470	Dexameth	16.77	12
CCDC77	84318	Leukemia, MESH:D015470	Dexameth	16.77	8
CMTM7	112616	Leukemia, MESH:D015470	Doxorubic	16.77	13
DIAPH1	1729	Leukemia, MESH:D015470	Arsenic Tr	16.77	13
EID3	493861	Leukemia, MESH:D015470	Dexameth	16.77	8
FYB2	199920	Leukemia, MESH:D015470	Cytarabine	16.77	76
GFI1	2672	Leukemia, MESH:D015473	Arsenic|De	16.77	154
GSAP	54103	Leukemia, MESH:D015470	Doxorubic	16.77	13
HLA-DMA	3108	Leukemia, MESH:D015473	Arsenic|De	16.77	159
KCNIP4	80333	Leukemia, MESH:D015470	Arsenic Tr	16.77	25
MAPK11	5600	Leukemia, MESH:D015470	Arsenic Tr	16.77	18
MICAL2	9645	Leukemia, MESH:D015470	Doxorubic	16.77	14
MIR449A	554213	Leukemia, MESH:D015470	Dexameth	16.77	6
MYOCD	93649	Leukemia, MESH:D015473	Arsenic|Ar	16.77	159
NLRP12	91662	Leukemia, MESH:D015473	Arsenic|Ar	16.77	262
RUFY3	22902	Leukemia, MESH:D015470	Doxorubic	16.77	13
SLC16A7	9194	Leukemia, MESH:D015470	Doxorubic	16.77	15
TFB2M	64216	Leukemia, MESH:D015470	Doxorubic	16.77	13
TRIL	9865	Leukemia, MESH:D015470	Arsenic Tr	16.77	13
TULP4	56995	Leukemia, MESH:D015470	Dexameth	16.77	11
VCPIP1	80124	Leukemia, MESH:D015470	Arsenic Tr	16.77	13
WDR62	284403	Leukemia, MESH:D015470	Calcitriol|D	16.77	12
ZC3H12C	85463	Leukemia, MESH:D015470	Calcitriol|D	16.77	13
ADH1B	125	Leukemia, MESH:D015473	Arsenic Tr	16.76	261
ATP8B2	57198	Leukemia, MESH:D015473	arsenite|G	16.76	156
BPI	671	Leukemia, MESH:D015470	Arsenic Tr	16.76	12
CENATAC	338657	Leukemia, MESH:D015470	Decitabine	16.76	13
CPPED1	55313	Leukemia, MESH:D015473	arsenite|D	16.76	154
DOCK5	80005	Leukemia, MESH:D015473	Arsenic|ars	16.76	157
ECPAS	23392	Leukemia, MESH:D015473	Arsenic|Ar	16.76	265
LDLR	3949	Leukemia, MESH:D007948	2-(2-amin	16.76	2
MTMR11	10903	Leukemia, MESH:D015473	Arsenic|Ar	16.76	263
NRARP	441478	Leukemia, MESH:D015473	arsenite|Ca	16.76	155
NUPL1	71844	Leukemia, MESH:D015470	(+)-JQ1 cc	16.76	8
PIN1	5300	Leukemia, MESH:D015473	Arsenic Tr	16.76	262
PROSER1	80209	Leukemia, MESH:D015470	Doxorubic	16.76	12
PWWP3A	84939	Leukemia, MESH:D015473	Arsenic|Ar	16.76	263
SNRPE	6635	Leukemia, MESH:D015473	Arsenic Tr	16.76	156
TRANK1	9881	Leukemia, MESH:D015473	Arsenic|ars	16.76	157
FAM50B	26240	Leukemia, MESH:D015470	Dexameth	16.75	6
HSPA4	3308	Leukemia, MESH:D015470	Arsenic Tr	16.75	23
INSIG2	51141	Leukemia, MESH:D015473	Arsenic|Ca	16.75	156

IQCH	64799	Leukemia, MESH:D015470	Arsenic Tr	16.75	12
KCNS1	3787	Leukemia, MESH:D015470	Dexameth	16.75	8
PRDM11	56981	Leukemia, MESH:D015470	Dexameth	16.75	12
TDRD9	122402	Leukemia, MESH:D015473	Arsenic|De	16.75	10
TSPYL5	85453	Leukemia, MESH:D015470	Doxorubic	16.75	12
ZNF385D	79750	Leukemia, MESH:D015470	Doxorubic	16.75	12
ADRA1B	147	Leukemia, MESH:D015473	Arsenic|ars	16.74	158
CABP5B	553766	Leukemia, MESH:D015470	Dexameth	16.74	4
KRT27	342574	Leukemia, MESH:D015473	Arsenic|Ar	16.74	158
MGST3	4259	Leukemia, MESH:D015473	arsenite|D	16.74	157
ACP3	55	Leukemia, MESH:D015473	Arsenic|Ge	16.73	153
ATP7A	538	Leukemia, MESH:D015473	Buthionine	16.73	155
ZNF516	9658	Leukemia, MESH:D015473	Arsenic|Ar	16.73	158
ABHD4	63874	Leukemia, MESH:D015470	Dasatinib|I	16.72	15
ANKH	56172	Leukemia, MESH:D015473	Arsenic|Ca	16.72	153
EHF	26298	Leukemia, MESH:D015473	Antimony	16.72	7
RAD51	5888	Leukemia, MESH:D004915	Cytarabine	16.72	2
ADAM8	101	Leukemia, MESH:D015470	Calcitriol|D	16.71	16
ALPP	250	Leukemia, MESH:D015473	Arsenic Tr	16.71	258
AQP7	364	Leukemia, MESH:D015470	Dexameth	16.71	15
ARHGAP1	9824	Leukemia, MESH:D015470	Calcitriol|D	16.71	14
CPA4	51200	Leukemia, MESH:D015473	arsenite|Ca	16.71	154
EIF2S2	8894	Leukemia, MESH:D015470	Calcitriol|D	16.71	18
ELAVL1	1994	Leukemia, MESH:D015470	Arsenic Tr	16.71	22
ETFB	2109	Leukemia, MESH:D015470	Arsenic Tr	16.71	21
GPC1	2817	Leukemia, MESH:D015470	Arsenic Tr	16.71	21
GRP	2922	Leukemia, MESH:D015473	Arsenic Tr	16.71	156
HSD3B1	3283	Leukemia, MESH:D015473	Calcitriol|D	16.71	156
PARP14	54625	Leukemia, MESH:D015470	(+)-JQ1 cc	16.71	10
RNH1	6050	Leukemia, MESH:D015473	Antimony	16.71	154
RRAD	6236	Leukemia, MESH:D015473	Calcitriol|D	16.71	154
SLC35G1	159371	Leukemia, MESH:D015473	Arsenic Tr	16.71	260
TCIRG1	10312	Leukemia, MESH:D015473	Arsenic|so	16.71	159
GPIB	246095	Leukemia, MESH:D015470	Alitretinoi	16.7	7
LHCGR	3973	Leukemia, MESH:D015473	arsenite|D	16.7	157
MBD4	8930	Leukemia, MESH:D015473	Arsenic Tr	16.7	262
MCOLN2	255231	Leukemia, MESH:D015473	arsenite|D	16.7	8
PDLIM4	8572	Leukemia, MESH:D015473	arsenite|Cy	16.7	157
SCN3B	55800	Leukemia, MESH:D015473	Arsenic|Eto	16.7	155
SLC16A14	151473	Leukemia, MESH:D015473	Arsenic|Ca	16.7	156
SP100	6672	Leukemia, MESH:D015473	Arsenic|De	16.7	157
UBE2S	27338	Leukemia, MESH:D015473	Arsenic|ars	16.7	156
ANKRD28	23243	Leukemia, MESH:D015470	Doxorubic	16.69	21
BICD1	636	Leukemia, MESH:D015470	Azacitidine	16.69	10
CAVIN4	347273	Leukemia, MESH:D015473	arsenite|D	16.69	157
CCNG1	900	Leukemia, MESH:D004915	Cytarabine	16.69	2
DNAJB5	25822	Leukemia, MESH:D015473	Dexameth	16.69	153
FBXL4	26235	Leukemia, MESH:D015473	Arsenic Tr	16.69	261
GABRD	2563	Leukemia, MESH:D015473	arsenite|D	16.69	153
LSS	4047	Leukemia, MESH:D015470	Calcitriol|D	16.69	16
MARK2	2011	Leukemia, MESH:D015470	Air Polluta	16.69	13
SRD5A2A	550398	Leukemia, MESH:D015470	Alitretinoi	16.69	7
SRPK1	6732	Leukemia, MESH:D015473	arsenite|Ca	16.69	157
TMCC2	9911	Leukemia, MESH:D015470	Doxorubic	16.69	14
ARHGAP9	64333	Leukemia, MESH:D015470	Arsenic Tr	16.68	23
ARHGEF39	84904	Leukemia, MESH:D015470	Calcitriol|I	16.68	7
ARL3	403	Leukemia, MESH:D015470	(+)-JQ1 cc	16.68	10
BLCAP	10904	Leukemia, MESH:D015470	Benzene|D	16.68	33

CBX2	84733	Leukemia, MESH:D015470	Calcitriol|N	16.68	10
DENND2D	79961	Leukemia, MESH:D015470	Air Polluta	16.68	10
DNMT3L	29947	Leukemia, MESH:D015470	Arsenic Tr	16.68	15
ESRP1	54845	Leukemia, MESH:D015470	Arsenic Tr	16.68	21
ESYT2	57488	Leukemia, MESH:D015470	Bortezomi	16.68	13
EXOC2	55770	Leukemia, MESH:D015470	Dexameth	16.68	12
FCGBP	8857	Leukemia, MESH:D015470	Decitabine	16.68	15
FRMD5	84978	Leukemia, MESH:D015470	Dexameth	16.68	14
LCORL	254251	Leukemia, MESH:D015470	Arsenic Tr	16.68	22
LLGL2	3993	Leukemia, MESH:D015473	Arsenic Tr	16.68	262
LSM4	25804	Leukemia, MESH:D015470	Bortezomi	16.68	14
NDUFA10	4705	Leukemia, MESH:D015470	Arsenic Tr	16.68	20
PHTF1	10745	Leukemia, MESH:D015470	Bortezomi	16.68	10
PRKX	5613	Leukemia, MESH:D015473	Daunorub	16.68	20
PURB	5814	Leukemia, MESH:D015470	Benzene|D	16.68	27
PXK	54899	Leukemia, MESH:D015470	Arsenic Tr	16.68	19
RDH5	5959	Leukemia, MESH:D015470	Dexameth	16.68	8
RHOD	29984	Leukemia, MESH:D015470	Arsenic Tr	16.68	19
SCOC	60592	Leukemia, MESH:D015470	Calcitriol|D	16.68	7
SERPINB6	5269	Leukemia, MESH:D015470	Calcitriol|C	16.68	81
SETD4	54093	Leukemia, MESH:D015470	Dexameth	16.68	8
SF3B3	23450	Leukemia, MESH:D015470	Air Polluta	16.68	15
SPSB4	92369	Leukemia, MESH:D015470	Doxorubic	16.68	14
SPTB	6710	Leukemia, MESH:D015473	Arsenic Tr	16.68	260
STYXL1	51657	Leukemia, MESH:D015470	Doxorubic	16.68	15
TMEM144	55314	Leukemia, MESH:D015470	Decitabine	16.68	19
TMEM39A	55254	Leukemia, MESH:D015470	Air Polluta	16.68	25
TRDMT1	1787	Leukemia, MESH:D015470	Decitabine	16.68	18
VWA5A	4013	Leukemia, MESH:D015470	Arsenic Tr	16.68	16
ZNF740B	767693	Leukemia, MESH:D015473	Dexameth	16.68	4
AGO4	192670	Leukemia, MESH:D015470	Doxorubic	16.67	15
CENPT	80152	Leukemia, MESH:D015470	Dexameth	16.67	8
DDX18	8886	Leukemia, MESH:D015473	Resveratrc	16.67	159
KDM4C	23081	Leukemia, MESH:D015470	Doxorubic	16.67	13
NFASC	23114	Leukemia, MESH:D015470	Calcitriol|D	16.67	13
SRC	6714	Leukemia, MESH:D007948	2-(2-amin	16.67	3
UGCG	7357	Leukemia, MESH:D015473	Dexameth	16.67	158
ANKRD36	400986	Leukemia, MESH:D015470	Cytarabine	16.66	72
F2RL1	2150	Leukemia, MESH:D015473	Arsenic Tr	16.66	260
FKBP4	2288	Leukemia, MESH:D015473	Dexameth	16.66	155
HSPA1A	3303	Leukemia, MESH:D007948	Arsenic Tr	16.66	3
RELA	5970	Leukemia, MESH:D004915	Cytarabine	16.66	3
WNT4	54361	Leukemia, MESH:D015473	Arsenic|ars	16.66	158
MIR337	442905	Leukemia, MESH:D015473	Arsenic|Bu	16.65	9
NPY1R	4886	Leukemia, MESH:D015470	Dexameth	16.65	12
PCBP1	5093	Leukemia, MESH:D015473	Arsenic Tr	16.65	156
PDGFRB	5159	Leukemia, MESH:D004915	Cytarabine	16.65	3
PIK3CG	5294	Leukemia, MESH:D015470	Arsenic Tr	16.65	23
PLCB4	5332	Leukemia, MESH:D015470	Arsenic Tr	16.65	29
PSMA4	5685	Leukemia, MESH:D015470	Air Polluta	16.65	26
RAB30	27314	Leukemia, MESH:D015470	Air Polluta	16.65	16
RPA1	6117	Leukemia, MESH:D015470	Cyclophos	16.65	19
TSHR	7253	Leukemia, MESH:D015470	Azacitidine	16.65	12
CHAF1B	8208	Leukemia, MESH:D015470	Calcitriol|D	16.64	10
ENPEP	2028	Leukemia, MESH:D015470	Dronabinc	16.64	11
IQGAP2	10788	Leukemia, MESH:D015470	Indometha	16.64	14
JMY	133746	Leukemia, MESH:D015473	arsenite|Ca	16.64	154
PDLIM5B	393165	Leukemia, MESH:D015473	Dexameth	16.64	152

PLLP	51090	Leukemia, MESH:D015473	Arsenic Tr	16.64	165
RFLNB	359845	Leukemia, MESH:D015473	Calcitriol|D	16.64	153
RPL26	6154	Leukemia, MESH:D015473	Arsenic Tr	16.64	157
SLC38A4	55089	Leukemia, MESH:D015470	(+)-JQ1 cc	16.64	11
TAGLN	6876	Leukemia, MESH:D004915	Cytarabine	16.64	3
TRIM13	10206	Leukemia, MESH:D015473	Calcitriol|D	16.64	7
YWHAZ	7534	Leukemia, MESH:D015473	Arsenic Tr	16.64	266
CELSR1	9620	Leukemia, MESH:D015473	Arsenic Tr	16.63	261
CPZ	8532	Leukemia, MESH:D015473	Calcitriol|C	16.63	156
CYP11A1	1583	Leukemia, MESH:D007948	Arsenic Tr	16.63	3
EXOC3	11336	Leukemia, MESH:D015473	Arsenic|De	16.63	159
LILRB1	10859	Leukemia, MESH:D015473	Arsenic|Ar	16.63	158
ND4	4538	Leukemia, MESH:D015473	Arsenic|Ar	16.63	160
NOD2	64127	Leukemia, MESH:D015473	Arsenic|Ca	16.63	9
PHACTR2	9749	Leukemia, MESH:D015473	arsenite|Cy	16.63	156
POM121L8	29797	Leukemia, MESH:D015470	Dexameth	16.63	7
VPS13A	23230	Leukemia, MESH:D015473	arsenite|Ca	16.63	8
ATP5MC1	516	Leukemia, MESH:D015473	Arsenic|ars	16.62	12
B3GALT5-	114041	Leukemia, MESH:D015470	Dexameth	16.62	5
KRBOX5	124411	Leukemia, MESH:D015470	Arsenic Tr	16.62	12
NIFK	84365	Leukemia, MESH:D015473	Arsenic Tr	16.62	262
PDCD2	5134	Leukemia, MESH:D007948	Arsenic Tr	16.62	3
SIT1	27240	Leukemia, MESH:D015470	Air Polluta	16.62	6
SLC7A14	57709	Leukemia, MESH:D015470	Arsenic Tr	16.62	22
TMEM117	84216	Leukemia, MESH:D015473	Calcitriol|C	16.62	155
TMEM45A	55076	Leukemia, MESH:D015473	Arsenic|Cy	16.62	158
USP1	7398	Leukemia, MESH:D015473	arsenite|Ca	16.62	157
ZNF282	8427	Leukemia, MESH:D015473	Cytarabine	16.62	153
ACCSL	390110	Leukemia, MESH:D015470	Dexameth	16.61	6
ATMIN	23300	Leukemia, MESH:D004915	Doxorubic	16.61	2
DMWD	1762	Leukemia, MESH:D015470	Dronabino	16.61	7
ELMO1	9844	Leukemia, MESH:D015473	Arsenic Tr	16.61	262
KRT8	3856	Leukemia, MESH:D004915	Cytarabine	16.61	2
MAP1LC3I	81631	Leukemia, MESH:D007948	2-(2-amin	16.61	3
RPL36A	6173	Leukemia, MESH:D015473	Arsenic Tr	16.61	262
SLC10A1	6554	Leukemia, MESH:D015470	Allopurino	16.61	15
SLC18A2	6571	Leukemia, MESH:D015470	Air Polluta	16.61	11
SLC2A1	6513	Leukemia, MESH:D007948	2-(2-amin	16.61	4
SMPDL3B	27293	Leukemia, MESH:D015473	Arsenic|ars	16.61	157
BNIPL	149428	Leukemia, MESH:D015470	Dronabino	16.6	6
BORCS7	119032	Leukemia, MESH:D015470	Doxorubic	16.6	15
FGF19	9965	Leukemia, MESH:D015470	Dasatinib|	16.6	10
MIR455	619556	Leukemia, MESH:D015470	Benzene|D	16.6	26
RFWD3	55159	Leukemia, MESH:D015470	Calcitriol|D	16.6	16
ARL8B	55207	Leukemia, MESH:D015470	Arsenic Tr	16.59	20
BCAP29	55973	Leukemia, MESH:D015470	Calcitriol|D	16.59	13
CALCOCC	57658	Leukemia, MESH:D015470	Dasatinib|	16.59	9
CCDC66	285331	Leukemia, MESH:D015470	Doxorubic	16.59	11
CLPTM1	1209	Leukemia, MESH:D015470	Bortezomi	16.59	14
COMMD2	51122	Leukemia, MESH:D015470	Dexameth	16.59	12
CRBN	51185	Leukemia, MESH:D015470	Arsenic Tr	16.59	19
CSMD2	114784	Leukemia, MESH:D015470	Calcitriol|D	16.59	11
CSNK1G1	53944	Leukemia, MESH:D015470	Decitabine	16.59	18
DENND4A	10260	Leukemia, MESH:D015470	Bortezomi	16.59	13
DMTF1	9988	Leukemia, MESH:D015470	Benzene|D	16.59	22
EMC10	284361	Leukemia, MESH:D015470	Doxorubic	16.59	13
KERA	11081	Leukemia, MESH:D015470	Dexameth	16.59	6
LHX1	3975	Leukemia, MESH:D015470	Calcitriol|E	16.59	18

MFF	56947	Leukemia, MESH:D015470	Deferoxan	16.59	12
NEK2	4751	Leukemia, MESH:D015473	arsenite|Ca	16.59	157
PADI4	23569	Leukemia, MESH:D015470	Alitretinoi	16.59	13
PHF6	84295	Leukemia, MESH:D015470	Methotrex	16.59	12
PUF60	22827	Leukemia, MESH:D015470	Air Polluta	16.59	20
RPL23A	6147	Leukemia, MESH:D015470	Dexameth	16.59	9
S100A3	6274	Leukemia, MESH:D015470	Calcitriol|D	16.59	13
SEC14L1	6397	Leukemia, MESH:D015470	Calcitriol|C	16.59	80
SGCB	6443	Leukemia, MESH:D015470	Dronabinc	16.59	10
SH3YL1	26751	Leukemia, MESH:D015470	Calcitriol|D	16.59	16
SOAT1	6646	Leukemia, MESH:D015470	Air Polluta	16.59	11
UPF2	26019	Leukemia, MESH:D015470	Arsenic Tr	16.59	19
USP24	23358	Leukemia, MESH:D015470	Dexameth	16.59	8
UVSSA	57654	Leukemia, MESH:D015470	Dexameth	16.59	8
ZBED6	1E+08	Leukemia, MESH:D015470	Dronabinc	16.59	6
ZNF33B	7582	Leukemia, MESH:D015470	Arsenic Tr	16.59	15
ACOX2	8309	Leukemia, MESH:D015470	Calcitriol|D	16.58	20
ALDH9A1	223	Leukemia, MESH:D015470	Doxorubic	16.58	15
C1QBP	708	Leukemia, MESH:D015470	Dexameth	16.58	24
CASQ2	845	Leukemia, MESH:D015470	Dexameth	16.58	21
CCT3	7203	Leukemia, MESH:D015470	Arsenic Tr	16.58	19
DKC1	1736	Leukemia, MESH:D015470	Dexameth	16.58	10
DNAH1	25981	Leukemia, MESH:D015470	Dexameth	16.58	7
DUSP9	1852	Leukemia, MESH:D015470	Dexameth	16.58	8
ENY2	56943	Leukemia, MESH:D015470	Dexameth	16.58	13
ERBIN	55914	Leukemia, MESH:D015470	Doxorubic	16.58	13
ESCO2	157570	Leukemia, MESH:D015470	Calcitriol|D	16.58	17
FBXO44	93611	Leukemia, MESH:D015470	Doxorubic	16.58	11
HECA	51696	Leukemia, MESH:D015470	Arsenic Tr	16.58	14
HNF1A	6927	Leukemia, MESH:D015470	Azacitidin	16.58	18
IFT122	55764	Leukemia, MESH:D015470	Doxorubic	16.58	14
INPP5D	3635	Leukemia, MESH:D015470	Decitabine	16.58	15
LRRC8B	23507	Leukemia, MESH:D015470	Arsenic Tr	16.58	18
MIS18A	54069	Leukemia, MESH:D015473	arsenite|Ca	16.58	7
NSL1	25936	Leukemia, MESH:D015470	Arsenic Tr	16.58	19
PAQR8	85315	Leukemia, MESH:D015470	Air Polluta	16.58	8
PDZRN4	29951	Leukemia, MESH:D015470	Arsenic Tr	16.58	12
PFN1	5216	Leukemia, MESH:D015470	Azacitidin	16.58	15
RALBP1	10928	Leukemia, MESH:D015470	Arsenic Tr	16.58	19
RBM20	282996	Leukemia, MESH:D015470	Doxorubic	16.58	14
RPL3	6122	Leukemia, MESH:D015470	Arsenic Tr	16.58	23
SCG3	29106	Leukemia, MESH:D015470	Cyclophos	16.58	8
SLC32A1	140679	Leukemia, MESH:D015470	Dexameth	16.58	8
SLCO4A1	28231	Leukemia, MESH:D015470	Dexameth	16.58	19
SOCS1	8651	Leukemia, MESH:D015473	Arsenic Tr	16.58	263
TRARG1	286753	Leukemia, MESH:D015470	Dexameth	16.58	7
UGT1A6	54578	Leukemia, MESH:D015473	Antimony	16.58	161
VASH1	22846	Leukemia, MESH:D015470	Arsenic Tr	16.58	20
VGLL3	389136	Leukemia, MESH:D015470	Dexameth	16.58	8
ZNF654	55279	Leukemia, MESH:D015470	Doxorubic	16.58	12
FGF5	2250	Leukemia, MESH:D015473	Arsenic|ars	16.57	11
NPHS2	7827	Leukemia, MESH:D015473	Arsenic Tr	16.57	259
SMS	6611	Leukemia, MESH:D015473	Arsenic Tr	16.57	155
SOSTDC1	25928	Leukemia, MESH:D015473	Arsenic Tr	16.57	260
H3F4	382523	Leukemia, MESH:D015473	Arsenic Tr	16.56	262
KCNE3	10008	Leukemia, MESH:D015473	Arsenic|Et	16.56	155
OSR1	130497	Leukemia, MESH:D015473	Arsenic|Cy	16.56	19
PRKACA	5566	Leukemia, MESH:D015470	Dexameth	16.56	15

RFFL	117584	Leukemia, MESH:D015473	Calcitriol|D	16.56	6
SLC38A5	92745	Leukemia, MESH:D015473	arsenite|D	16.56	154
TCF7L1	83439	Leukemia, MESH:D015473	Arsenic Tr	16.56	155
ZNF605	1E+08	Leukemia, MESH:D015473	Arsenic|ars	16.56	156
GNAI2	2771	Leukemia, MESH:D015473	alpha-Toc	16.55	155
KCNS3	3790	Leukemia, MESH:D015473	Arsenic Tr	16.55	261
MLF1	4291	Leukemia, MESH:D015473	Calcitriol|D	16.55	154
MSRB3	253827	Leukemia, MESH:D015473	Arsenic|Ar	16.55	264
MYO5C	55930	Leukemia, MESH:D015473	Arsenic|Ar	16.55	263
POU3F1	5453	Leukemia, MESH:D015473	arsenite|Ca	16.55	155
H1-0	3005	Leukemia, MESH:D015473	Arsenic Tr	16.54	260
PAX6	5080	Leukemia, MESH:D015473	Arsenic|ars	16.54	160
PCSK9	255738	Leukemia, MESH:D015473	Arsenic Tr	16.54	261
PIGH	5283	Leukemia, MESH:D004915	Cytarabine	16.54	3
SMG6	23293	Leukemia, MESH:D004915	Doxorubic	16.54	2
ARRDC4	91947	Leukemia, MESH:D015470	Calcitriol|D	16.52	13
ATP2A1	487	Leukemia, MESH:D015470	Bortezomi	16.52	15
BMPR2	659	Leukemia, MESH:D015470	Arsenic Tr	16.52	17
CFLAR	8837	Leukemia, MESH:D004915	Doxorubic	16.52	3
CREG1	8804	Leukemia, MESH:D015470	Doxorubic	16.52	14
EPHX2	2053	Leukemia, MESH:D015473	Arsenic Tr	16.52	263
GNAQ	2776	Leukemia, MESH:D015470	Air Polluta	16.52	10
LIPA	3988	Leukemia, MESH:D015470	Dexameth	16.52	25
PLA2G1B	5319	Leukemia, MESH:D007948	2-(2-amin	16.52	2
PTPRK	5796	Leukemia, MESH:D015470	Decitabine	16.52	19
UQCRC2	7385	Leukemia, MESH:D015470	Arsenic Tr	16.52	21
YWHAG	7532	Leukemia, MESH:D015470	Arsenic Tr	16.52	17
CCNF	899	Leukemia, MESH:D015473	Arsenic Tr	16.51	266
INS2	16334	Leukemia, MESH:D015470	Arsenic Tr	16.51	17
MARCHF3	115123	Leukemia, MESH:D015470	Arsenic Tr	16.51	80
MAT2A	4144	Leukemia, MESH:D015473	Arsenic|De	16.51	160
MYL7	58498	Leukemia, MESH:D015473	Calcitriol|C	16.51	155
PFKFB3	5209	Leukemia, MESH:D015470	Air Polluta	16.51	19
SLC12A1	6557	Leukemia, MESH:D015470	Chloroqui	16.51	9
ACSS2	55902	Leukemia, MESH:D015473	Arsenic|Ar	16.5	263
CD84	8832	Leukemia, MESH:D015470	Arsenic Tr	16.5	15
CEP128	145508	Leukemia, MESH:D015470	Calcitriol|D	16.5	10
COL6A1	1291	Leukemia, MESH:D015473	Dexameth	16.5	155
CYP2B10	13088	Leukemia, MESH:D015473	alpha-Toc	16.5	159
FCMR	9214	Leukemia, MESH:D015470	Doxorubic	16.5	12
MIR30A	407029	Leukemia, MESH:D015470	Air Polluta	16.5	16
MRPL4	51073	Leukemia, MESH:D015470	Gasoline|Ir	16.5	13
NCS1	23413	Leukemia, MESH:D015470	Benzoates	16.5	20
REC8	9985	Leukemia, MESH:D015470	Decitabine	16.5	18
TUBB4B	10383	Leukemia, MESH:D015473	Arsenic|ars	16.5	160
AIF1L	83543	Leukemia, MESH:D015473	Cytarabine	16.49	158
ARHGAP1	55843	Leukemia, MESH:D015470	(+)-JQ1 cc	16.49	11
BPNT2	54928	Leukemia, MESH:D015470	Cytarabine	16.49	80
CCDC88C	440193	Leukemia, MESH:D015470	Calcitriol|D	16.49	8
CCL26	10344	Leukemia, MESH:D015470	Dexameth	16.49	9
CENPQ	55166	Leukemia, MESH:D015470	Calcitriol|(	16.49	10
CERS4	79603	Leukemia, MESH:D015470	Arsenic Tr	16.49	20
CLEC4D	338339	Leukemia, MESH:D015470	Doxorubic	16.49	16
DVL1	1855	Leukemia, MESH:D015470	Decitabine	16.49	19
ETNK2	55224	Leukemia, MESH:D015470	Calcitriol|D	16.49	14
FAM189A2	9413	Leukemia, MESH:D015470	Calcitriol|D	16.49	12
HOOK2	29911	Leukemia, MESH:D015470	Arsenic Tr	16.49	19
IGDCC4	57722	Leukemia, MESH:D015470	Dexameth	16.49	16

ING1	3621	Leukemia, MESH:D015470	Decitabine	16.49	14
MUCL1	118430	Leukemia, MESH:D015473	Arsenic|De	16.49	8
NIT2	56954	Leukemia, MESH:D015470	Dexameth	16.49	20
NLGN1	22871	Leukemia, MESH:D015470	Arsenic Tr	16.49	18
PANX2	56666	Leukemia, MESH:D015470	Arsenic Tr	16.49	14
PCLO	27445	Leukemia, MESH:D015470	Alitretinoi	16.49	12
PDSS1	23590	Leukemia, MESH:D015470	Calcitriol|D	16.49	14
PLEKHH2	130271	Leukemia, MESH:D015470	Dasatinib|	16.49	13
PLEKHO1	51177	Leukemia, MESH:D015470	Arsenic Tr	16.49	18
RDM1	201299	Leukemia, MESH:D015470	Calcitriol|D	16.49	14
RNF6	6049	Leukemia, MESH:D015470	Arsenic Tr	16.49	21
SCAPER	49855	Leukemia, MESH:D015470	Calcitriol|D	16.49	13
SURF1	6834	Leukemia, MESH:D015470	Dexameth	16.49	14
TMEM163	81615	Leukemia, MESH:D015470	Arsenic Tr	16.49	16
USP25	29761	Leukemia, MESH:D015473	arsenite|G	16.49	155
ADD2	119	Leukemia, MESH:D015470	Dexameth	16.48	12
ATG10	83734	Leukemia, MESH:D015473	Arsenic|ars	16.48	11
B4GALT6	9331	Leukemia, MESH:D015473	Calcitriol|D	16.48	154
EPHB6	2051	Leukemia, MESH:D015473	Arsenic|ars	16.48	156
GDF15	9518	Leukemia, MESH:D007948	2-(2-amin	16.48	3
H4C8	8365	Leukemia, MESH:D015473	Arsenic Tr	16.48	261
PCGF5	84333	Leukemia, MESH:D015470	Doxorubic	16.48	13
PDZRN3	23024	Leukemia, MESH:D015473	arsenite|Ca	16.48	155
TNFRSF17	608	Leukemia, MESH:D015470	Air Polluta	16.48	16
ACOT1	641371	Leukemia, MESH:D015470	Benzene|D	16.47	26
AGPAT4	56895	Leukemia, MESH:D015473	Arsenic Tr	16.47	261
CSPP1	79848	Leukemia, MESH:D015473	Arsenic Tr	16.47	260
EMG1	10436	Leukemia, MESH:D015473	arsenite|D	16.47	155
EMSY	56946	Leukemia, MESH:D004915	Doxorubic	16.47	2
MLPH	79083	Leukemia, MESH:D015473	arsenite|D	16.47	155
MPO	4353	Leukemia, MESH:D004915	Doxorubic	16.47	4
NAT2	10	Leukemia, MESH:D015470	Benzene|C	16.47	26
RAB38	23682	Leukemia, MESH:D015473	Dexameth	16.47	155
SMYD3	64754	Leukemia, MESH:D015473	Arsenic|Ar	16.47	263
SNRPF	6636	Leukemia, MESH:D015473	Arsenic Tr	16.47	262
SOX5	6660	Leukemia, MESH:D015473	arsenite|D	16.47	155
ALDH18A1	5832	Leukemia, MESH:D015470	Bortezomi	16.46	12
CCT5	22948	Leukemia, MESH:D015470	Air Polluta	16.46	14
CTNNAL1	8727	Leukemia, MESH:D015470	Arsenic Tr	16.46	19
CYB5R2	51700	Leukemia, MESH:D007948	Arsenic Tr	16.46	4
H4C4	8360	Leukemia, MESH:D015470	Doxorubic	16.46	11
HMCN2	256158	Leukemia, MESH:D015470	Doxorubic	16.46	12
HMOX1A	791518	Leukemia, MESH:D015470	Arsenic Tr	16.46	16
PPP1R10	5514	Leukemia, MESH:D015470	Air Polluta	16.46	32
SCAMP5	192683	Leukemia, MESH:D007948	Arsenic Tr	16.46	4
SGMS1	259230	Leukemia, MESH:D015470	Bortezomi	16.46	15
USP2	9099	Leukemia, MESH:D015473	arsenite|D	16.46	157
LMO7	4008	Leukemia, MESH:D015470	Doxorubic	16.45	14
OLFM1	10439	Leukemia, MESH:D015470	Doxorubic	16.45	17
RPL10A	4736	Leukemia, MESH:D015470	Arsenic Tr	16.45	14
ANKRD49	54851	Leukemia, MESH:D015470	Dexameth	16.44	11
C14ORF13	56967	Leukemia, MESH:D015470	Bortezomi	16.44	12
CDNF	441549	Leukemia, MESH:D015470	Arsenic Tr	16.44	12
COL2A1	1280	Leukemia, MESH:D015473	arsenite|D	16.44	157
GMEB2	26205	Leukemia, MESH:D015470	Benzene|D	16.44	25
H2-DMA	14998	Leukemia, MESH:D015470	Dexameth	16.44	12
POLR3GL	84265	Leukemia, MESH:D015470	Decitabine	16.44	12
RPS9	6203	Leukemia, MESH:D015473	Arsenic Tr	16.44	156

WDR82	80335	Leukemia, MESH:D015470	Arsenic Tr	16.44	12
AOPEP	84909	Leukemia, MESH:D015473	Arsenic|Ar	16.43	263
ASB6	140459	Leukemia, MESH:D015470	Doxorubic	16.43	13
CEACAM5	1048	Leukemia, MESH:D015470	(+)-JQ1 co	16.43	9
DHRSX	207063	Leukemia, MESH:D015470	Arsenic Tr	16.43	18
KLHL24	54800	Leukemia, MESH:D015473	Arsenic Tr	16.43	262
KREMEN2	79412	Leukemia, MESH:D015470	Dronabino	16.43	7
LIMS1	3987	Leukemia, MESH:D015473	arsenite|Ca	16.43	154
MAO	404730	Leukemia, MESH:D015470	Dexameth	16.43	7
NEPRO	25871	Leukemia, MESH:D015470	Dexameth	16.43	13
NMB	4828	Leukemia, MESH:D015473	arsenite|D	16.43	152
ODAD2	55130	Leukemia, MESH:D015470	(+)-JQ1 co	16.43	7
PPP1R3E	90673	Leukemia, MESH:D015470	Doxorubic	16.43	14
THAP3	90326	Leukemia, MESH:D015470	Dexameth	16.43	6
TRIM66	9866	Leukemia, MESH:D015470	Dexameth	16.43	6
USP30	84749	Leukemia, MESH:D015470	Doxorubic	16.43	11
ZNF44	51710	Leukemia, MESH:D015470	Methotrex	16.43	9
ZNF562	54811	Leukemia, MESH:D015470	Dexameth	16.43	10
ZNF682	91120	Leukemia, MESH:D015470	Arsenic Tr	16.43	13
ARHGEF6	9459	Leukemia, MESH:D015473	arsenite|Ca	16.42	155
C9ORF40	55071	Leukemia, MESH:D015473	Calcitriol|F	16.42	155
MIEN1	84299	Leukemia, MESH:D015470	Dexameth	16.42	11
NPPC	4880	Leukemia, MESH:D015473	arsenite|Ca	16.42	7
RPS19BP1	91582	Leukemia, MESH:D015470	Dexameth	16.42	12
SIGMAR1	10280	Leukemia, MESH:D015473	arsenite|D	16.42	157
TMEM176	28959	Leukemia, MESH:D015473	Calcitriol|G	16.42	153
ZNF507	22847	Leukemia, MESH:D015470	Doxorubic	16.42	13
ABCF2	10061	Leukemia, MESH:D015473	Arsenic|Me	16.41	157
ADAM22	53616	Leukemia, MESH:D015473	Calcitriol|D	16.41	153
ANKRA2	57763	Leukemia, MESH:D015470	Ifosfamide	16.41	12
AP2A1	160	Leukemia, MESH:D015470	Bortezomi	16.41	9
CENPJ	55835	Leukemia, MESH:D015473	Calcitriol|D	16.41	154
CPEB3	22849	Leukemia, MESH:D015473	Arsenic|ars	16.41	11
DGCR8	54487	Leukemia, MESH:D015470	Arsenic Tr	16.41	17
DROSHA	29102	Leukemia, MESH:D015470	Clioquinol	16.41	14
ERCC3	2071	Leukemia, MESH:D015470	Benzene|B	16.41	30
GPX3	2878	Leukemia, MESH:D004915	Doxorubic	16.41	3
KIAA0232	9778	Leukemia, MESH:D015473	Arsenic|ars	16.41	155
PCP4	5121	Leukemia, MESH:D015473	arsenite|D	16.41	8
PIM2	11040	Leukemia, MESH:D015473	Dexameth	16.41	154
SCLY	51540	Leukemia, MESH:D015473	Arsenic Tr	16.41	261
SPAG1	6674	Leukemia, MESH:D015473	arsenite|Ca	16.41	157
SRPRB	58477	Leukemia, MESH:D015473	Dexameth	16.41	155
STAG1	10274	Leukemia, MESH:D015470	Decitabine	16.41	16
VPS37B	79720	Leukemia, MESH:D015470	Arsenic Tr	16.41	14
VSTM2A	222008	Leukemia, MESH:D015473	Arsenic|De	16.41	154
ANKRD33	651746	Leukemia, MESH:D015470	Dexameth	16.4	17
ATP5B	11947	Leukemia, MESH:D015470	Dexameth	16.4	22
BLZF1	8548	Leukemia, MESH:D015470	Arsenic Tr	16.4	13
CD37	951	Leukemia, MESH:D015470	Alitretinoi	16.4	10
DTWD1	56986	Leukemia, MESH:D015470	Calcitriol|D	16.4	8
EIF3D	8664	Leukemia, MESH:D015470	Doxorubic	16.4	13
GABRE	2564	Leukemia, MESH:D015470	Arsenic Tr	16.4	13
GBP6	163351	Leukemia, MESH:D015470	Dexameth	16.4	14
GMPR	2766	Leukemia, MESH:D015473	Arsenic|De	16.4	156
GRIK3	2899	Leukemia, MESH:D015470	Arsenic Tr	16.4	16
HSF2	3298	Leukemia, MESH:D015470	Arsenic Tr	16.4	20
KAT5	10524	Leukemia, MESH:D015470	Arsenic Tr	16.4	21

KCNA2	3737	Leukemia, MESH:D015470	Dexameth	16.4	8
KITL	17311	Leukemia, MESH:D015473	Arsenic|De	16.4	158
LAMTOR2	28956	Leukemia, MESH:D015470	Decitabine	16.4	20
LIMD2	80774	Leukemia, MESH:D015470	Arsenic Tr	16.4	19
MRTO4	51154	Leukemia, MESH:D015470	Dexameth	16.4	10
NAAA	27163	Leukemia, MESH:D015470	Arsenic Tr	16.4	27
NKX2-5	1482	Leukemia, MESH:D015470	Azacitidine	16.4	14
NUDT5	11164	Leukemia, MESH:D015470	Air Polluta	16.4	14
P2RX5	5026	Leukemia, MESH:D015470	Doxorubic	16.4	16
PAG1	55824	Leukemia, MESH:D015473	Arsenic|De	16.4	156
PI4KA	5297	Leukemia, MESH:D015470	Arsenic Tr	16.4	14
R3HDM1	23518	Leukemia, MESH:D004915	Doxorubic	16.4	2
RBCK1	10616	Leukemia, MESH:D015470	Dexameth	16.4	8
RENBP	5973	Leukemia, MESH:D015473	Arsenic|ars	16.4	157
RPRD2	23248	Leukemia, MESH:D004915	Doxorubic	16.4	2
SBNO1	55206	Leukemia, MESH:D015470	Calcitriol|T	16.4	11
SYNJ1	8867	Leukemia, MESH:D015470	Doxorubic	16.4	13
UNC13D	201294	Leukemia, MESH:D015470	Doxorubic	16.4	13
UNC5C	8633	Leukemia, MESH:D015470	Doxorubic	16.4	20
WDR61	80349	Leukemia, MESH:D015470	Dexameth	16.4	12
WNT2	7472	Leukemia, MESH:D015470	Decitabine	16.4	21
ADAMTS1	170689	Leukemia, MESH:D015470	Arsenic Tr	16.39	19
C1QTNF4	114900	Leukemia, MESH:D015470	Arsenic Tr	16.39	13
CLCN7	1186	Leukemia, MESH:D015470	Dexameth	16.39	13
DCK	1633	Leukemia, MESH:D015470	Cytarabine	16.39	82
HIPK3	10114	Leukemia, MESH:D015470	Doxorubic	16.39	14
MGAT1	4245	Leukemia, MESH:D015470	Dexameth	16.39	13
PLCG1	5335	Leukemia, MESH:D015473	Arsenic|ca	16.39	12
PNRC2	55629	Leukemia, MESH:D015470	Dexameth	16.39	14
SH3GL3	6457	Leukemia, MESH:D015470	Calcitriol|D	16.39	8
SORL1	6653	Leukemia, MESH:D015473	Arsenic|ars	16.39	158
ZNF660	285349	Leukemia, MESH:D015473	Arsenic|De	16.39	155
APOB	338	Leukemia, MESH:D004915	Daunorub	16.38	3
HK1	3098	Leukemia, MESH:D015473	Arsenic|Ar	16.38	266
OSGIN1	29948	Leukemia, MESH:D015470	Bortezomi	16.38	11
SLCO1A1	28248	Leukemia, MESH:D015470	Androgen	16.38	26
MASTL	84930	Leukemia, MESH:D015473	Arsenic|Da	16.37	10
MIS18BP1	55320	Leukemia, MESH:D015473	arsenite|Ca	16.37	9
NOX4	50507	Leukemia, MESH:D004915	Cytarabine	16.37	2
RAB1A	5861	Leukemia, MESH:D015473	Arsenic Tr	16.37	263
TNFSF10	8743	Leukemia, MESH:D007948	Arsenic Tr	16.37	4
GSTM3	2947	Leukemia, MESH:D015473	Arsenic|Ar	16.36	266
HIC1	3090	Leukemia, MESH:D015473	Dexameth	16.36	153
MXD3	83463	Leukemia, MESH:D015473	Arsenic|Ca	16.36	158
CHAC2	494143	Leukemia, MESH:D015473	Calcitriol|G	16.35	155
CTTNBP2N	55917	Leukemia, MESH:D015473	Arsenic|Ar	16.35	159
FIGN	55137	Leukemia, MESH:D015473	Arsenic|ars	16.35	155
LHB	3972	Leukemia, MESH:D007948	2-(2-amin	16.35	3
MDM2	4193	Leukemia, MESH:D007948	2-(2-amin	16.35	3
NPNT	255743	Leukemia, MESH:D015473	Arsenic|Et	16.35	155
PNOC	5368	Leukemia, MESH:D015473	arsenite|G	16.35	155
RNF128	79589	Leukemia, MESH:D015473	Arsenic Tr	16.35	262
TAT	6898	Leukemia, MESH:D015473	Arsenic Tr	16.35	262
CCL2	6347	Leukemia, MESH:D007948	2-(2-amin	16.34	3
EPHA7	2045	Leukemia, MESH:D015473	arsenite|D	16.34	155
GC	2638	Leukemia, MESH:D015470	Calcitriol|D	16.34	15
LYAR	55646	Leukemia, MESH:D015473	Calcitriol|D	16.34	156
NDUFA3	4696	Leukemia, MESH:D015473	arsenite|D	16.34	157

PTPN14	5784	Leukemia, MESH:D015473	arsenite|D	16.34	155
RPL19	6143	Leukemia, MESH:D015473	arsenite|D	16.34	8
SRRT	51593	Leukemia, MESH:D015473	Arsenic Tr	16.34	263
SUV39H2	79723	Leukemia, MESH:D015473	Calcitriol|D	16.34	156
TIMM8B	26521	Leukemia, MESH:D015473	Arsenic|ars	16.34	13
USP10	9100	Leukemia, MESH:D015473	Arsenic|De	16.34	158
WASF2	10163	Leukemia, MESH:D015473	arsenite|D	16.34	157
BRD4	23476	Leukemia, MESH:D015470	Doxorubic	16.33	14
CCT2	10576	Leukemia, MESH:D015470	Arsenic Tr	16.33	20
JMJD6	23210	Leukemia, MESH:D015470	Dexameth	16.33	14
NFE2L2	4780	Leukemia, MESH:D007948	2-(2-amin	16.33	3
SLC22A3	6581	Leukemia, MESH:D015470	Cyclophos	16.33	25
SORD	6652	Leukemia, MESH:D015470	Cyclophos	16.33	10
SYNE2	23224	Leukemia, MESH:D015470	Doxorubic	16.33	17
ZFP52	22710	Leukemia, MESH:D015470	Hydroxyur	16.33	9
CCNE1	898	Leukemia, MESH:D007948	2-(2-amin	16.32	3
CDK4	1019	Leukemia, MESH:D007948	2-(2-amin	16.32	3
CTLA4	1493	Leukemia, MESH:D015470	Benzene|D	16.32	29
ESPN	83715	Leukemia, MESH:D015473	Genistein|I	16.32	7
LCP2	3937	Leukemia, MESH:D015470	Dronabinc	16.32	12
LHB	3972	Leukemia, MESH:D015473	2-(2-chlor	16.32	262
MAP3K20	51776	Leukemia, MESH:D015470	Calcitriol|D	16.32	13
NPHP1	4867	Leukemia, MESH:D015470	Azacitidine	16.32	11
PKD1	5310	Leukemia, MESH:D015470	Dexameth	16.32	7
PLK3	1263	Leukemia, MESH:D015473	Daunorub	16.32	19
PRR13	54458	Leukemia, MESH:D015470	Decitabine	16.32	15
RAB10	10890	Leukemia, MESH:D015470	Air Polluta	16.32	14
WNT2B	7482	Leukemia, MESH:D015470	(+)-JQ1 cc	16.32	11
ALDOAB	406496	Leukemia, MESH:D015470	Alitretinoi	16.31	6
ATL3	25923	Leukemia, MESH:D015470	Dexameth	16.31	9
ATP5PB	515	Leukemia, MESH:D015470	Calcitriol|D	16.31	13
FAF1	11124	Leukemia, MESH:D015470	Bortezomi	16.31	14
FGD6	55785	Leukemia, MESH:D015470	Doxorubic	16.31	15
FNTA	2339	Leukemia, MESH:D015470	Decitabine	16.31	19
HNRNPH2	3188	Leukemia, MESH:D015470	Arsenic Tr	16.31	28
IRX1	79192	Leukemia, MESH:D015470	Decitabine	16.31	23
ISOC1	51015	Leukemia, MESH:D015470	Dexameth	16.31	15
NDUFA8	4702	Leukemia, MESH:D015470	Air Polluta	16.31	12
NLRC5	84166	Leukemia, MESH:D015470	Calcitriol|D	16.31	13
PARD6B	84612	Leukemia, MESH:D015470	Dronabinc	16.31	8
RNF145	153830	Leukemia, MESH:D015470	Calcitriol|D	16.31	13
SLC26A6	65010	Leukemia, MESH:D015470	Dexameth	16.31	11
SPDEF	25803	Leukemia, MESH:D015470	Dexameth	16.31	9
STRN3	29966	Leukemia, MESH:D015470	Arsenic Tr	16.31	16
TANC2	26115	Leukemia, MESH:D015470	Arsenic Tr	16.31	14
TRAK1	22906	Leukemia, MESH:D015470	Doxorubic	16.31	14
ZNF45	7596	Leukemia, MESH:D015470	Doxorubic	16.31	20
AHCYL2	23382	Leukemia, MESH:D015470	Arsenic Tr	16.3	18
COX7A2	1347	Leukemia, MESH:D015473	arsenite|ca	16.3	156
FAIM	55179	Leukemia, MESH:D015470	Dexameth	16.3	10
GPD1	2819	Leukemia, MESH:D015473	Arsenic Tr	16.3	261
H2BC10	8346	Leukemia, MESH:D015470	Air Polluta	16.3	14
HUNK	30811	Leukemia, MESH:D015470	Doxorubic	16.3	13
ITGB4	3691	Leukemia, MESH:D015473	Arsenic Tr	16.3	261
MIR135B	442891	Leukemia, MESH:D015470	Decitabine	16.3	25
PITPNB	23760	Leukemia, MESH:D015470	Dexameth	16.3	16
RCOR2	283248	Leukemia, MESH:D015470	Dexameth	16.3	9
AP1G1	164	Leukemia, MESH:D015473	arsenite|Ca	16.29	153

C9ORF64	84267	Leukemia, MESH:D015470	Air Polluta	16.29	7
EN1	2019	Leukemia, MESH:D015470	Decitabine	16.29	17
FAAP100	80233	Leukemia, MESH:D015470	Air Polluta	16.29	7
IBSP	3381	Leukemia, MESH:D007948	2-(2-amin	16.29	1
IFT52	51098	Leukemia, MESH:D015470	Air Polluta	16.29	13
LINC0095	255031	Leukemia, MESH:D015470	Gasoline|(	16.29	5
MIR22HG	84981	Leukemia, MESH:D015473	Arsenic|Ca	16.29	158
MYO15B	80022	Leukemia, MESH:D015470	Air Polluta	16.29	6
PCNX1	22990	Leukemia, MESH:D015473	Arsenic|ar	16.29	11
TDP1	55775	Leukemia, MESH:D015473	Arsenic Tr	16.29	261
TMED8	283578	Leukemia, MESH:D015470	Doxorubic	16.29	11
ZNF280C	55609	Leukemia, MESH:D015470	Doxorubic	16.29	12
ZNF362	149076	Leukemia, MESH:D015470	Calcitriol|D	16.29	7
ADAMTS2	9509	Leukemia, MESH:D015473	Arsenic|Ar	16.28	263
ADAT3	113179	Leukemia, MESH:D015470	Arsenic Tr	16.28	15
AGR3	155465	Leukemia, MESH:D015470	(+)-JQ1 co	16.28	8
AUTS2	26053	Leukemia, MESH:D015473	Arsenic|Ar	16.28	264
BMP2K	55589	Leukemia, MESH:D015473	Dexameth	16.28	153
CCM2	83605	Leukemia, MESH:D015470	Air Polluta	16.28	12
DCAKD	79877	Leukemia, MESH:D015473	Arsenic|ar	16.28	158
EGLN1	54583	Leukemia, MESH:D015470	Calcitriol|C	16.28	11
FHIP1B	84067	Leukemia, MESH:D015470	Dexameth	16.28	13
GPR50	9248	Leukemia, MESH:D015470	Calcitriol|D	16.28	12
KNSTRN	90417	Leukemia, MESH:D015470	Calcitriol|D	16.28	11
LHFPL2	10184	Leukemia, MESH:D015473	Calcitriol|D	16.28	154
MIR342	442909	Leukemia, MESH:D015470	Arsenic Tr	16.28	14
MYL2	4633	Leukemia, MESH:D015473	Dexameth	16.28	155
PDCD11	22984	Leukemia, MESH:D015473	Dexameth	16.28	153
RNF135	84282	Leukemia, MESH:D015470	Doxorubic	16.28	12
SNUPN	10073	Leukemia, MESH:D015470	Arsenic Tr	16.28	17
TBC1D10E	26000	Leukemia, MESH:D015470	Doxorubic	16.28	13
TMEFF2	23671	Leukemia, MESH:D015473	Arsenic|Ar	16.28	265
UGT1A1	54658	Leukemia, MESH:D007948	2-(2-amin	16.28	2
VPS35L	57020	Leukemia, MESH:D015473	arsenite|Ca	16.28	6
APCDD1	147495	Leukemia, MESH:D015473	Arsenic|Ca	16.27	156
CCDC152	1E+08	Leukemia, MESH:D015470	Doxorubic	16.27	14
DIS3L	115752	Leukemia, MESH:D015470	Doxorubic	16.27	14
ERCC6	2074	Leukemia, MESH:D015470	Arsenic Tr	16.27	28
ESRRG	2104	Leukemia, MESH:D015470	Arsenic Tr	16.27	19
GNB2	2783	Leukemia, MESH:D015473	arsenite|D	16.27	155
IHH	3549	Leukemia, MESH:D015470	Arsenic Tr	16.27	18
MAP3K4	4216	Leukemia, MESH:D015473	Arsenic|ar	16.27	159
MKNK1	8569	Leukemia, MESH:D007948	2-(2-amin	16.27	2
PXN	5829	Leukemia, MESH:D015470	Dasatinib|I	16.27	14
SLC47A1	55244	Leukemia, MESH:D015473	Arsenic|Cy	16.27	20
SPTAN1	6709	Leukemia, MESH:D015473	Arsenic Tr	16.27	261
ST3GAL4	6484	Leukemia, MESH:D015473	arsenite|Ca	16.27	155
TLE2	7089	Leukemia, MESH:D015473	Arsenic|Ar	16.27	263
WDCP	80304	Leukemia, MESH:D015470	Dexameth	16.27	12
AQP8A.1	447923	Leukemia, MESH:D015470	Dronabinc	16.26	6
C8ORF44	56260	Leukemia, MESH:D015470	Dexameth	16.26	5
CARD16	114769	Leukemia, MESH:D015473	Arsenic Tr	16.26	261
KLHL24B	393412	Leukemia, MESH:D015470	Dexameth	16.26	5
LRRC37A2	474170	Leukemia, MESH:D015470	Dexameth	16.26	9
NCOR1	9611	Leukemia, MESH:D015473	arsenite|Ca	16.26	158
NT5DC4	284958	Leukemia, MESH:D015470	Dexameth	16.26	7
TANGO6	79613	Leukemia, MESH:D015470	Dexameth	16.26	12
RGPD5	84220	Leukemia, MESH:D015470	Arsenic Tr	16.25	17

CYP8B1	1582	Leukemia, MESH:D015470	Allopurinc	16.24	27
ENO1	2023	Leukemia, MESH:D004915	Doxorubic	16.24	3
MMP10	4319	Leukemia, MESH:D015473	Genistein|I	16.24	152
NRTN	4902	Leukemia, MESH:D015473	Arsenic|Ar	16.24	261
PHTF2	57157	Leukemia, MESH:D015473	arsenite|Ca	16.24	8
AKAP8	10270	Leukemia, MESH:D015470	Air Polluta	16.23	13
CD79B	974	Leukemia, MESH:D015470	Cyclophos	16.23	11
E2F5	1875	Leukemia, MESH:D015473	Arsenic|Ar	16.23	267
GRIA2	2891	Leukemia, MESH:D015473	Arsenic Tr	16.23	265
NACA	4666	Leukemia, MESH:D015473	Arsenic Tr	16.23	158
PDE8A	5151	Leukemia, MESH:D015470	Arsenic Tr	16.23	22
SAFB	6294	Leukemia, MESH:D015470	Bortezomi	16.23	8
SLC27A3	11000	Leukemia, MESH:D015473	arsenite|D	16.23	10
SLC6A9	6536	Leukemia, MESH:D015473	Arsenic|Ar	16.23	160
SSRP1	6749	Leukemia, MESH:D015470	Bortezomi	16.23	16
TIMM17A	10440	Leukemia, MESH:D015473	arsenite|D	16.23	156
ADH1C	126	Leukemia, MESH:D015470	Dexameth	16.22	9
ANXA8	653145	Leukemia, MESH:D015473	Cytarabine	16.22	156
BICC1	80114	Leukemia, MESH:D015470	Dexameth	16.22	13
CA4	762	Leukemia, MESH:D015470	Decitabine	16.22	15
CCNT2	905	Leukemia, MESH:D015470	Benzene|R	16.22	24
CGN	57530	Leukemia, MESH:D015473	Arsenic|Ca	16.22	155
CITED1	4435	Leukemia, MESH:D015470	Decitabine	16.22	21
COPB2	9276	Leukemia, MESH:D015470	Air Polluta	16.22	14
CREB3L2	64764	Leukemia, MESH:D015473	Arsenic Tr	16.22	261
CYP3A5	1577	Leukemia, MESH:D004915	Etoposide	16.22	4
DEGS1	8560	Leukemia, MESH:D015470	Dexameth	16.22	8
HR	55806	Leukemia, MESH:D015473	Calcitriol|C	16.22	155
IGF2BP2	10644	Leukemia, MESH:D015473	Arsenic|Ar	16.22	262
IGSF9	57549	Leukemia, MESH:D015470	Calcitriol|D	16.22	12
IL12RB2	3595	Leukemia, MESH:D015470	Arsenic Tr	16.22	14
JAG2	3714	Leukemia, MESH:D015473	Arsenic|Ar	16.22	161
KAT14	57325	Leukemia, MESH:D015470	Dexameth	16.22	12
KCNE1	3753	Leukemia, MESH:D015470	Arsenic Tr	16.22	13
MANEA	79694	Leukemia, MESH:D015470	Doxorubic	16.22	17
METRN	79006	Leukemia, MESH:D015470	Cyclophos	16.22	13
MRPL50	54534	Leukemia, MESH:D015470	Doxorubic	16.22	16
NHSL1	57224	Leukemia, MESH:D015470	Air Polluta	16.22	11
NUP37	79023	Leukemia, MESH:D015470	Arsenic Tr	16.22	14
NUP88	4927	Leukemia, MESH:D015470	Doxorubic	16.22	15
PCBP4	57060	Leukemia, MESH:D015470	Etoposide	16.22	17
PGPEP1	54858	Leukemia, MESH:D015473	Calcitriol|C	16.22	155
PRR5L	79899	Leukemia, MESH:D015473	Arsenic|Ca	16.22	10
PTH2R	5746	Leukemia, MESH:D015473	Arsenic|M	16.22	9
PTP4A3	11156	Leukemia, MESH:D015470	Bortezomi	16.22	14
PXDC1	221749	Leukemia, MESH:D015470	Decitabine	16.22	14
RAB5C	5878	Leukemia, MESH:D015470	Decitabine	16.22	17
SAR1A	56681	Leukemia, MESH:D015470	Arsenic Tr	16.22	16
SNRNP70	6625	Leukemia, MESH:D015470	Decitabine	16.22	16
SPEN	23013	Leukemia, MESH:D015470	Doxorubic	16.22	14
STMN3	50861	Leukemia, MESH:D015470	Cyclophos	16.22	13
THRAP3	9967	Leukemia, MESH:D015470	Irinotecan	16.22	11
TK2	7084	Leukemia, MESH:D015470	Air Polluta	16.22	79
ALPK1	80216	Leukemia, MESH:D015470	Dexameth	16.21	13
B3GNT2	10678	Leukemia, MESH:D015473	arsenite|Ca	16.21	155
FZD7	8324	Leukemia, MESH:D015470	Decitabine	16.21	17
GABBR1	2550	Leukemia, MESH:D015470	Arsenic Tr	16.21	19
NPC2	10577	Leukemia, MESH:D015470	Decitabine	16.21	18

PDCD6	10016	Leukemia, MESH:D015470	Dexameth	16.21	12
PIK3IP1	113791	Leukemia, MESH:D015473	Arsenic|Ca	16.21	156
PTPRM	5797	Leukemia, MESH:D015473	arsenite|Ca	16.21	155
RPS19	6223	Leukemia, MESH:D015470	Bortezomi	16.21	17
SCN8A	6334	Leukemia, MESH:D015473	arsenite|D	16.21	155
SMAD9	4093	Leukemia, MESH:D015473	Arsenic|De	16.21	158
SMARCA5	8467	Leukemia, MESH:D015473	arsenite|G	16.21	156
SRPK2	6733	Leukemia, MESH:D015473	Arsenic|ars	16.21	156
TCEAL8	90843	Leukemia, MESH:D015470	Dexameth	16.21	12
TM4SF1	4071	Leukemia, MESH:D015470	Arsenic Tr	16.21	15
WDR75	84128	Leukemia, MESH:D015470	Dexameth	16.21	13
GPATCH8	23131	Leukemia, MESH:D004915	Doxorubic	16.2	2
ITGB3	3690	Leukemia, MESH:D015470	Arsenic Tr	16.2	27
ACTG1	71	Leukemia, MESH:D015473	Arsenic Tr	16.19	262
FOXO1	2308	Leukemia, MESH:D004915	Doxorubic	16.19	3
BCL2L1	598	Leukemia, MESH:D004915	Cytarabine	16.18	3
CCL13	6357	Leukemia, MESH:D015473	Dexameth	16.18	4
RPTN	126638	Leukemia, MESH:D015473	Antimony	16.18	154
ADGRF4	221393	Leukemia, MESH:D015470	Calcitriol|E	16.17	8
ADIPOQ	9370	Leukemia, MESH:D007948	Arsenic Tr	16.17	4
MIR181C	406957	Leukemia, MESH:D015470	Arsenic Tr	16.17	14
PPP1R3A	5506	Leukemia, MESH:D015470	Daunorub	16.17	44
CCR3	1232	Leukemia, MESH:D015473	Arsenic|De	16.16	155
CDC42BPA	8476	Leukemia, MESH:D015473	Arsenic|Ar	16.16	264
CXCL10	3627	Leukemia, MESH:D007948	2-(2-amin	16.16	3
GUSB	2990	Leukemia, MESH:D015473	alpha-Toc	16.16	156
HOXC8	3224	Leukemia, MESH:D015473	arsenite|Cy	16.16	155
IL18	3606	Leukemia, MESH:D007948	2-(2-amin	16.16	3
MLXIPL	51085	Leukemia, MESH:D015470	Decitabine	16.16	15
NUMB	8650	Leukemia, MESH:D015473	Arsenic|Ar	16.16	264
PSME2	5721	Leukemia, MESH:D015473	Arsenic|Ar	16.16	267
RNASE2	6036	Leukemia, MESH:D015470	Arsenic Tr	16.16	79
SELPLG	6404	Leukemia, MESH:D015473	Arsenic Tr	16.16	261
SIRT1	23411	Leukemia, MESH:D007948	Arsenic Tr	16.16	3
THPO	7066	Leukemia, MESH:D007948	pyrazolant	16.16	2
UGT3A1	133688	Leukemia, MESH:D015470	Ethylnitros	16.16	7
BCAR1	9564	Leukemia, MESH:D015470	Arsenic Tr	16.15	20
CAMK2A	815	Leukemia, MESH:D015473	Arsenic|Ar	16.15	161
COX6A1	1337	Leukemia, MESH:D015473	arsenite|D	16.15	9
CREB5	9586	Leukemia, MESH:D015470	Arsenic Tr	16.15	26
DCPS	28960	Leukemia, MESH:D015470	Benzoates	16.15	14
DENND6B	414918	Leukemia, MESH:D015470	Calcitriol|D	16.15	11
DMGDH	29958	Leukemia, MESH:D015473	Calcitriol|D	16.15	153
DPP9	91039	Leukemia, MESH:D015473	Arsenic|ars	16.15	11
EEF2K	29904	Leukemia, MESH:D015470	Doxorubic	16.15	14
ETFA	2108	Leukemia, MESH:D015470	Arsenic Tr	16.15	21
GNAI1	2770	Leukemia, MESH:D015470	Doxorubic	16.15	14
GPR63	81491	Leukemia, MESH:D015473	Arsenic|ars	16.15	11
GRIA1	2890	Leukemia, MESH:D015473	Arsenic Tr	16.15	263
HOXC5	3222	Leukemia, MESH:D015470	Alitretinoi	16.15	7
HSD3B1	3283	Leukemia, MESH:D015470	Azacitidine	16.15	17
IDO1	3620	Leukemia, MESH:D015470	Decitabine	16.15	17
MNS1	55329	Leukemia, MESH:D015473	Calcitriol|D	16.15	153
PLCB3	5331	Leukemia, MESH:D015473	arsenite|Ca	16.15	157
PPID	5481	Leukemia, MESH:D015473	Arsenic|De	16.15	12
PPP2R2B	5521	Leukemia, MESH:D015470	Decitabine	16.15	20
REEP6	92840	Leukemia, MESH:D015470	Decitabine	16.15	19
RPLP2	6181	Leukemia, MESH:D015473	arsenite|Ca	16.15	9

SLC31A2	1318	Leukemia, MESH:D015473	Calcitriol|[illegible]	16.15	153
SYBU	55638	Leukemia, MESH:D015473	Arsenic|Ca	16.15	158
TGFB1I1	7041	Leukemia, MESH:D015470	Alitretinoi	16.15	20
ARNTL2	56938	Leukemia, MESH:D015470	Dasatinib|[illegible]	16.14	13
ASPN	54829	Leukemia, MESH:D015470	Calcitriol|[illegible]	16.14	12
C21ORF58	54058	Leukemia, MESH:D015470	Calcitriol|[illegible]	16.14	7
EPHB3	2049	Leukemia, MESH:D015470	Doxorubic	16.14	13
FUT3	2525	Leukemia, MESH:D015473	Arsenic Tr	16.14	154
GANAB	23193	Leukemia, MESH:D015470	Arsenic Tr	16.14	22
INTS5	80789	Leukemia, MESH:D015470	Doxorubic	16.14	14
KIAA1671	85379	Leukemia, MESH:D015470	Calcitriol|[illegible]	16.14	74
MADD	8567	Leukemia, MESH:D015473	Dexameth	16.14	155
MSS51	118490	Leukemia, MESH:D015470	Cyclophos	16.14	11
MTRF1L	54516	Leukemia, MESH:D015470	Decitabine	16.14	20
NOVA1	4857	Leukemia, MESH:D015470	Arsenic Tr	16.14	82
OSBP2	23762	Leukemia, MESH:D015470	Decitabine	16.14	18
PRIMPOL	201973	Leukemia, MESH:D015473	Calcitriol|[illegible]	16.14	152
PRXL2C	195827	Leukemia, MESH:D015470	Air Polluta	16.14	6
PTGS2	5743	Leukemia, MESH:D004915	Cytarabine	16.14	3
RAB40C	57799	Leukemia, MESH:D015470	Dexameth	16.14	8
RLF	6018	Leukemia, MESH:D015470	Air Polluta	16.14	13
RNF13	11342	Leukemia, MESH:D015473	Arsenic|Ar	16.14	263
SAPCD2	89958	Leukemia, MESH:D015470	Arsenic Tr	16.14	20
TGS1	96764	Leukemia, MESH:D015470	Indometha	16.14	11
TICRR	90381	Leukemia, MESH:D015470	Calcitriol|[illegible]	16.14	8
TMEM100	55273	Leukemia, MESH:D015470	Calcitriol|(	16.14	11
TRIM17	51127	Leukemia, MESH:D015470	Dronabinc	16.14	7
TRIOBP	11078	Leukemia, MESH:D015470	Dexameth	16.14	7
ZBTB43	23099	Leukemia, MESH:D015470	Air Polluta	16.14	11
ADSL	158	Leukemia, MESH:D015470	Arsenic Tr	16.13	18
AGPAT9	1.03E+08	Leukemia, MESH:D015470	Arsenic Tr	16.13	13
ASTN2	23245	Leukemia, MESH:D015470	Decitabine	16.13	19
ATG4B	23192	Leukemia, MESH:D015470	Arsenic Tr	16.13	21
ATP8B2	57198	Leukemia, MESH:D015470	Dronabinc	16.13	10
C4ORF46	201725	Leukemia, MESH:D015473	Calcitriol|F	16.13	155
CTDSP1	58190	Leukemia, MESH:D015470	Arsenic Tr	16.13	15
CXCL6	6372	Leukemia, MESH:D015470	Dexameth	16.13	13
DYNC1I1	1780	Leukemia, MESH:D015470	Bortezomi	16.13	14
ECPAS	23392	Leukemia, MESH:D015470	Arsenic Tr	16.13	13
FAM131B	9715	Leukemia, MESH:D015470	Calcitriol|T	16.13	7
GRAMD1B	57476	Leukemia, MESH:D015470	(+)-JQ1 cc	16.13	9
GRWD1	83743	Leukemia, MESH:D015470	Doxorubic	16.13	15
H2AC17	8336	Leukemia, MESH:D015470	Arsenic Tr	16.13	17
KDM5A	5927	Leukemia, MESH:D015470	(+)-JQ1 cc	16.13	11
KIF21A	55605	Leukemia, MESH:D015470	Dexameth	16.13	11
LRRK1	79705	Leukemia, MESH:D015470	Arsenic Tr	16.13	13
MOGS	7841	Leukemia, MESH:D015470	Dexameth	16.13	13
MYOCD	93649	Leukemia, MESH:D015470	Arsenic Tr	16.13	20
NCR3LG1	374383	Leukemia, MESH:D015470	Air Polluta	16.13	12
PALS2	51678	Leukemia, MESH:D015470	Cytarabine	16.13	82
PITPNA	5306	Leukemia, MESH:D015470	Doxorubic	16.13	14
PWWP3A	84939	Leukemia, MESH:D015470	Arsenic Tr	16.13	14
RFK	55312	Leukemia, MESH:D015470	Dexameth	16.13	13
SH3PXD2B	285590	Leukemia, MESH:D015470	Calcitriol|[illegible]	16.13	12
SLC25A18	83733	Leukemia, MESH:D015470	Dexameth	16.13	11
SPIN1	10927	Leukemia, MESH:D015470	Dexameth	16.13	8
TBC1D16	125058	Leukemia, MESH:D015470	Doxorubic	16.13	12
TMEM132	92293	Leukemia, MESH:D015470	Dexameth	16.13	6

UACA	55075	Leukemia, MESH:D015470	Doxorubic	16.13	14
UBTF	7343	Leukemia, MESH:D015470	Dexameth	16.13	12
WRAP53	55135	Leukemia, MESH:D015470	Calcitriol|[	16.13	6
CEP97	79598	Leukemia, MESH:D015470	Doxorubic	16.12	12
CLBA1	122616	Leukemia, MESH:D015470	Dexameth	16.12	7
FBXO42	54455	Leukemia, MESH:D015470	Dexameth	16.12	12
FUZ	80199	Leukemia, MESH:D015470	Dexameth	16.12	7
MON1A	84315	Leukemia, MESH:D015470	Doxorubic	16.12	12
OLFM4	10562	Leukemia, MESH:D015470	Dexameth	16.12	7
RERG	85004	Leukemia, MESH:D007948	Arsenic Tr	16.12	4
ACSL4	2182	Leukemia, MESH:D015473	arsenite|D	16.11	157
ADCY2	108	Leukemia, MESH:D015473	Arsenic|ars	16.11	11
MDN1	23195	Leukemia, MESH:D015473	Dexameth	16.11	156
TNF	7124	Leukemia, MESH:D004915	Cytarabine	16.11	4
CFL1	1072	Leukemia, MESH:D015470	Arsenic Tr	16.1	21
CLYBL	171425	Leukemia, MESH:D015473	Daunorub	16.1	160
EPSTI1	94240	Leukemia, MESH:D015473	Arsenic Tr	16.1	262
PIAS1	8554	Leukemia, MESH:D015473	Arsenic|Ar	16.1	159
SPRY2	10253	Leukemia, MESH:D015470	Calcitriol|[	16.1	9
TDO2	6999	Leukemia, MESH:D015470	Allopurinc	16.1	12
APOBEC3(	27350	Leukemia, MESH:D004915	Doxorubic	16.09	3
B3GNT5	84002	Leukemia, MESH:D015473	Dexameth	16.09	156
CASP3	836	Leukemia, MESH:D007948	2-(2-amin	16.09	4
CLSTN1	22883	Leukemia, MESH:D015473	Arsenic Tr	16.09	258
FGFBP1	9982	Leukemia, MESH:D015473	Arsenic|ars	16.09	157
FNBP1L	54874	Leukemia, MESH:D015473	Arsenic Tr	16.09	263
GJA5	2702	Leukemia, MESH:D015473	Arsenic Tr	16.09	260
PXMP4	11264	Leukemia, MESH:D015473	arsenite|D	16.09	9
SLC2A9	56606	Leukemia, MESH:D015473	Arsenic|ars	16.09	13
TCOF1	6949	Leukemia, MESH:D015473	Arsenic|Ca	16.09	158
TXNL1	9352	Leukemia, MESH:D015470	Dexameth	16.09	19
ZMYM3	9203	Leukemia, MESH:D015473	Dexameth	16.09	7
ARPC2	10109	Leukemia, MESH:D015473	Arsenic|De	16.08	156
CLCC1	23155	Leukemia, MESH:D004915	Doxorubic	16.08	2
DLGAP4	22839	Leukemia, MESH:D015473	Arsenic|Ar	16.08	263
IFNAR2	3455	Leukemia, MESH:D015473	Arsenic|De	16.08	155
MFAP5	8076	Leukemia, MESH:D015473	Calcitriol|[	16.08	156
MYOG	4656	Leukemia, MESH:D015473	Arsenic|De	16.08	158
PAFAH1B3	5050	Leukemia, MESH:D015473	Arsenic Tr	16.08	260
RAB3D	9545	Leukemia, MESH:D015473	arsenite|D	16.08	157
SLC12A5	57468	Leukemia, MESH:D015473	arsenite|D	16.08	155
TP53AIP1	63970	Leukemia, MESH:D015470	2-(2-chlor	16.08	18
ZNF148	7707	Leukemia, MESH:D004915	Doxorubic	16.08	2
CLCN1	1180	Leukemia, MESH:D015473	caffeic aci	16.07	4
MAPK7	5598	Leukemia, MESH:D007948	Arsenic Tr	16.07	3
MIR146A	406938	Leukemia, MESH:D007948	2-(2-amin	16.07	3
PDK4	5166	Leukemia, MESH:D015473	arsenite|D	16.07	157
ACVRL1	94	Leukemia, MESH:D015473	alpha-Toc	16.06	263
SLN	6588	Leukemia, MESH:D015470	Cytarabine	16.06	77
AARS1	16	Leukemia, MESH:D015470	Air Polluta	16.05	21
ACIN1	22985	Leukemia, MESH:D015473	Buthionine	16.05	155
ALPI	248	Leukemia, MESH:D015473	Genistein|l	16.05	6
APMAP	57136	Leukemia, MESH:D015470	Alitretinoi	16.05	13
BRD8	10902	Leukemia, MESH:D015470	Air Polluta	16.05	9
CNTRL	11064	Leukemia, MESH:D015470	Cytarabine	16.05	85
CTPS1	1503	Leukemia, MESH:D015470	Arsenic Tr	16.05	27
EHF	26298	Leukemia, MESH:D015470	Calcitriol|(	16.05	8
F5	2153	Leukemia, MESH:D015473	Antimony	16.05	264

GRP	2922	Leukemia, MESH:D015470		Arsenic Tr	16.05	18
IL18R1	8809	Leukemia, MESH:D015470		Bortezomi	16.05	9
SNTG2	54221	Leukemia, MESH:D015470		Methotrex	16.05	10
TESC	54997	Leukemia, MESH:D015470		Doxorubic	16.05	13
TNNT3	7140	Leukemia, MESH:D015470		Dexameth	16.05	9
USP47	55031	Leukemia, MESH:D015470		Doxorubic	16.05	13
WNT1	7471	Leukemia, MESH:D015470		Benzene|D	16.05	30
YKT6	10652	Leukemia, MESH:D015470		Arsenic Tr	16.05	14
ATXN2	6311	Leukemia, MESH:D015470		Dexameth	16.04	14
CETN2	1069	Leukemia, MESH:D015470		Dexameth	16.04	12
FOXG1	2290	Leukemia, MESH:D015470		Dexameth	16.04	10
GLRX2	51022	Leukemia, MESH:D015470		Indometha	16.04	10
GSE1	23199	Leukemia, MESH:D015470		Doxorubic	16.04	13
LRRC25	126364	Leukemia, MESH:D015470		Air Polluta	16.04	10
MAMDC2	256691	Leukemia, MESH:D015473		Arsenic Tr	16.04	154
MKLN1	4289	Leukemia, MESH:D015473		Arsenic|Ca	16.04	10
PGRMC1	10857	Leukemia, MESH:D015470		Dexameth	16.04	15
PLCD1	5333	Leukemia, MESH:D015473		Arsenic|Ar	16.04	159
RPRM	56475	Leukemia, MESH:D015470		Decitabine	16.04	16
S100A10	6281	Leukemia, MESH:D015473		Arsenic Tr	16.04	266
SLC1A6	6511	Leukemia, MESH:D015473		arsenite|Ca	16.04	158
TG	7038	Leukemia, MESH:D015470		15-deoxy-	16.04	18
TMEM86A	144110	Leukemia, MESH:D015470		Dexameth	16.04	9
ZBTB4	57659	Leukemia, MESH:D015470		Dexameth	16.04	8
NUMA1	4926	Leukemia, MESH:D01	marker/m	Arsenic Tr	16.03	156
MEP1A.2	565535	Leukemia, MESH:D015470		Dronabinc	16.03	6
NBL1	4681	Leukemia, MESH:D015473		Arsenic|Ca	16.03	154
RGS5	8490	Leukemia, MESH:D015470		Doxorubic	16.03	15
RNF213	57674	Leukemia, MESH:D015473		Arsenic|ars	16.03	157
SLC34A2	10568	Leukemia, MESH:D015470		Calcitriol|D	16.03	12
XRCC4	7518	Leukemia, MESH:D015473		Arsenic|Ar	16.03	160
ATPAF2	91647	Leukemia, MESH:D015473		Resveratrc	16.02	158
BMPR1B	658	Leukemia, MESH:D015473		Arsenic|Ca	16.02	158
CXCL8	3576	Leukemia, MESH:D007948		2-(2-amin	16.02	3
GCNT1	2650	Leukemia, MESH:D015473		Calcitriol|D	16.02	153
PLCB3	5331	Leukemia, MESH:D007948		2-(2-amin	16.02	2
PROM1	8842	Leukemia, MESH:D015473		Arsenic|Ar	16.02	169
QRICH1	54870	Leukemia, MESH:D015470		Gasoline|P	16.02	9
TYK2	7297	Leukemia, MESH:D015473		Arsenic Tr	16.02	260
ANKS1A	23294	Leukemia, MESH:D015470		Arsenic Tr	16.01	79
CD70	970	Leukemia, MESH:D007948		2-(2-amin	16.01	2
MAGEA12	4111	Leukemia, MESH:D015470		Decitabine	16.01	22
MMP12	4321	Leukemia, MESH:D015473		Arsenic Tr	16.01	154
RELN	5649	Leukemia, MESH:D015473		arsenite|Cy	16.01	159
ZMAT5	55954	Leukemia, MESH:D015470		Dexameth	16.01	10
FAM131A	131408	Leukemia, MESH:D015470		Benzene|D	16	28
HOXB-AS:	404266	Leukemia, MESH:D015470		Cytarabine	16	75
INO80	54617	Leukemia, MESH:D015470		Arsenic Tr	16	12
SOX15	6665	Leukemia, MESH:D015470		Decitabine	16	13
ABHD13	84945	Leukemia, MESH:D015470		Doxorubic	15.99	11
ALKBH1	8846	Leukemia, MESH:D015470		Doxorubic	15.99	15
COPRS	55352	Leukemia, MESH:D015473		arsenite|Ca	15.99	154
GTF2IRD2	389524	Leukemia, MESH:D015470		Air Polluta	15.99	11
LGALS3B	325599	Leukemia, MESH:D015473		Dexameth	15.99	152
NAT9	26151	Leukemia, MESH:D015470		Dexameth	15.99	10
NRSN2	80023	Leukemia, MESH:D015470		Dexameth	15.99	13
NUDT22	84304	Leukemia, MESH:D015470		Doxorubic	15.99	11
TBC1D32	221322	Leukemia, MESH:D015470		Gasoline|Ir	15.99	6

TMEM26	219623	Leukemia, MESH:D015473	arsenite|G	15.99	7
ADAMTS1	171019	Leukemia, MESH:D015470	Doxorubic	15.98	12
ARHGAP1	89839	Leukemia, MESH:D015470	Calcitriol|D	15.98	7
ASXL3	80816	Leukemia, MESH:D015470	Doxorubic	15.98	11
ATG4D	84971	Leukemia, MESH:D015470	Methotrex	15.98	9
B4GALT3	8703	Leukemia, MESH:D015470	Doxorubic	15.98	14
C5ORF34	375444	Leukemia, MESH:D015470	Calcitriol|(	15.98	7
DBNDD1	79007	Leukemia, MESH:D015470	Calcitriol|(	15.98	7
GK5	256356	Leukemia, MESH:D015470	Calcitriol|D	15.98	11
HSDL1	83693	Leukemia, MESH:D015470	Arsenic Tr	15.98	17
IL6RA	16194	Leukemia, MESH:D015473	Arsenic|De	15.98	13
LAMTOR1	55004	Leukemia, MESH:D015470	Doxorubic	15.98	12
LEMD3	23592	Leukemia, MESH:D015470	Dexameth	15.98	12
LRRC47	57470	Leukemia, MESH:D004915	Doxorubic	15.98	3
MBIP	51562	Leukemia, MESH:D015470	Doxorubic	15.98	11
MRPL28	10573	Leukemia, MESH:D015470	Dronabinc	15.98	7
NMT2	9397	Leukemia, MESH:D015473	Arsenic|Ar	15.98	261
PSMB5	5693	Leukemia, MESH:D015470	Bortezomi	15.98	15
RUSC1	23623	Leukemia, MESH:D015470	Dexameth	15.98	6
SNU13	4809	Leukemia, MESH:D015470	Arsenic Tr	15.98	18
SRRM3	222183	Leukemia, MESH:D015470	Calcitriol|D	15.98	6
TMEM178	130733	Leukemia, MESH:D015470	Arsenic Tr	15.98	13
TMEM242	729515	Leukemia, MESH:D015470	Dexameth	15.98	12
TPRN	286262	Leukemia, MESH:D015470	Dexameth	15.98	6
TRIM34	53840	Leukemia, MESH:D015470	Dexameth	15.98	12
ZNF135	7694	Leukemia, MESH:D015470	Medroxyp	15.98	8
ZNF641	121274	Leukemia, MESH:D015470	Arsenic Tr	15.98	12
ABCC1	4363	Leukemia, MESH:D007948	Arsenic Tr	15.97	4
ATP5D	66043	Leukemia, MESH:D015470	Dexameth	15.97	11
DPCD	25911	Leukemia, MESH:D015470	Dexameth	15.97	11
DVL2	1856	Leukemia, MESH:D015470	Decitabine	15.97	13
IARS1	3376	Leukemia, MESH:D015470	Arsenic Tr	15.97	14
PPAN	56342	Leukemia, MESH:D015473	Arsenic|Ge	15.97	11
RBM12	10137	Leukemia, MESH:D015470	Arsenic Tr	15.97	22
RBM45	129831	Leukemia, MESH:D015470	Dexameth	15.97	7
RPS2	6187	Leukemia, MESH:D015470	Dexameth	15.97	11
SMURF2	64750	Leukemia, MESH:D015470	Bortezomi	15.97	19
SOX8	30812	Leukemia, MESH:D015470	Azacitidine	15.97	10
SPACA9	11092	Leukemia, MESH:D015470	Arsenic Tr	15.97	17
THOC5	8563	Leukemia, MESH:D015470	Dexameth	15.97	7
UCHL5	51377	Leukemia, MESH:D015470	Arsenic Tr	15.97	13
VRK3	51231	Leukemia, MESH:D015470	Dexameth	15.97	11
ZNF598	90850	Leukemia, MESH:D015473	Arsenic|ars	15.97	10
AAAS	8086	Leukemia, MESH:D015470	Doxorubic	15.96	13
ADAR	103	Leukemia, MESH:D015470	Arsenic Tr	15.96	20
APOBB.1	321166	Leukemia, MESH:D015473	Dexameth	15.96	153
BTBD11	121551	Leukemia, MESH:D015470	Benzene|D	15.96	22
CD164	8763	Leukemia, MESH:D015470	Arsenic Tr	15.96	18
CHRNE	1145	Leukemia, MESH:D015470	Calcitriol|N	15.96	10
CRLF1	9244	Leukemia, MESH:D015470	Calcitriol|D	15.96	13
DAD1	1603	Leukemia, MESH:D015473	Arsenic Tr	15.96	260
DDX1	1653	Leukemia, MESH:D015473	Arsenic Tr	15.96	263
EIF4E3	317649	Leukemia, MESH:D015470	Arsenic Tr	15.96	14
EXOSC2	23404	Leukemia, MESH:D015473	Arsenic Tr	15.96	262
HAUS4	54930	Leukemia, MESH:D015470	Calcitriol|D	15.96	14
HINT1	3094	Leukemia, MESH:D015473	arsenite|D	15.96	155
HIVEP3	59269	Leukemia, MESH:D015473	Arsenic|De	15.96	156
HLTF	6596	Leukemia, MESH:D015470	Air Polluta	15.96	15

IDH3G	3421	Leukemia, MESH:D015470	Dexameth	15.96	14
IGSF1	3547	Leukemia, MESH:D015470	Dexameth	15.96	13
KDM7A	80853	Leukemia, MESH:D015473	Arsenic Tr	15.96	262
KPNA1	3836	Leukemia, MESH:D015470	Benzene|C	15.96	22
LHPP	64077	Leukemia, MESH:D015470	Dexameth	15.96	13
NEGR1	257194	Leukemia, MESH:D015470	Calcitriol|D	15.96	13
NMT1	4836	Leukemia, MESH:D015470	Arsenic Tr	15.96	19
NOL1	1E+08	Leukemia, MESH:D015470	Calcitriol|M	15.96	8
PCBP1	5093	Leukemia, MESH:D015470	Arsenic Tr	15.96	16
RBM24	221662	Leukemia, MESH:D015473	arsenite|Ca	15.96	157
RGPD6	729540	Leukemia, MESH:D015470	Dexameth	15.96	9
SHB	6461	Leukemia, MESH:D015470	Methotrex	15.96	11
TSPAN12	23554	Leukemia, MESH:D015470	Dexameth	15.96	9
TTC39A	22996	Leukemia, MESH:D015470	Bortezomi	15.96	14
TUBA8	51807	Leukemia, MESH:D015473	Arsenic|Ar	15.96	263
USP1	7398	Leukemia, MESH:D015470	Calcitriol|(	15.96	10
UTP25	27042	Leukemia, MESH:D004915	Doxorubic	15.96	2
VAPA	9218	Leukemia, MESH:D015470	Benzene|D	15.96	23
CDC27	996	Leukemia, MESH:D007948	Arsenic Tr	15.95	3
DNAJC1	64215	Leukemia, MESH:D015470	Doxorubic	15.95	13
FAM210B	116151	Leukemia, MESH:D015473	arsenite|D	15.95	155
GPIA	246094	Leukemia, MESH:D015470	Dexameth	15.95	5
PGAM1A	323107	Leukemia, MESH:D015470	Dexameth	15.95	5
PIPOX	51268	Leukemia, MESH:D015473	Arsenic|De	15.95	156
PTPRU	10076	Leukemia, MESH:D015473	Arsenic|De	15.95	156
HBB	3043	Leukemia, MESH:D015473	Arsenic Tr	15.94	156
ZNF718	255403	Leukemia, MESH:D015470	Calcitriol|T	15.94	5
DYRK1A	1859	Leukemia, MESH:D007948	Arsenic Tr	15.93	3
FUT8-AS1	645431	Leukemia, MESH:D015470	Dexameth	15.93	5
GJA1B	30236	Leukemia, MESH:D015470	Indometha	15.93	6
LGALS9	3965	Leukemia, MESH:D015473	Arsenic|Ar	15.93	266
PBX1A	58138	Leukemia, MESH:D015470	Dronabinc	15.93	6
PRKCQ-AS	439949	Leukemia, MESH:D015470	(+)-JQ1 cc	15.93	6
APOC1	341	Leukemia, MESH:D015470	Decitabine	15.92	21
CLEC18B	497190	Leukemia, MESH:D015470	Dexameth	15.92	5
EPB41	2035	Leukemia, MESH:D015473	Arsenic|Ch	15.92	155
FIS1	51024	Leukemia, MESH:D015470	Deferoxan	15.92	14
GOT2	2806	Leukemia, MESH:D015470	Arsenic Tr	15.92	19
MFN1	55669	Leukemia, MESH:D015470	Arsenic Tr	15.92	21
MTMR9LP	339483	Leukemia, MESH:D015470	Dexameth	15.92	5
OSMR	9180	Leukemia, MESH:D015470	Decitabine	15.92	21
RRAS2	22800	Leukemia, MESH:D015470	Arsenic Tr	15.92	22
SAA2	6289	Leukemia, MESH:D015473	Calcitriol|C	15.92	155
SIRT3	23410	Leukemia, MESH:D015470	Arsenic Tr	15.92	23
SLC26A2	1836	Leukemia, MESH:D015473	Methotrex	15.92	158
SYN1	6853	Leukemia, MESH:D015470	Air Polluta	15.92	15
ZNF71	58491	Leukemia, MESH:D015470	Doxorubic	15.92	10
CXCR4	7852	Leukemia, MESH:D004915	Cytarabine	15.91	2
LORICRIN	4014	Leukemia, MESH:D015473	Antimony	15.91	153
RBM25	58517	Leukemia, MESH:D015473	Arsenic|De	15.91	13
SCNN1G	6340	Leukemia, MESH:D015473	Arsenic|ca	15.91	155
ATXN7	6314	Leukemia, MESH:D004915	Doxorubic	15.9	2
CDCP1	64866	Leukemia, MESH:D015473	Arsenic|Ca	15.9	156
DNER	92737	Leukemia, MESH:D015473	Arsenic|Ca	15.9	155
ELK3	2004	Leukemia, MESH:D015473	arsenite|D	15.9	155
LMNB2	84823	Leukemia, MESH:D015473	arsenite|D	15.9	8
MFAP2	4237	Leukemia, MESH:D015473	Arsenic|ars	15.9	156
NR1I2	8856	Leukemia, MESH:D004915	Doxorubic	15.9	4

POLR1B	84172	Leukemia, MESH:D015473	Dexameth	15.9	153
RNF125	54941	Leukemia, MESH:D015473	Arsenic|Ge	15.9	155
RPL21	6144	Leukemia, MESH:D015473	Arsenic Tr	15.9	260
SDC4	6385	Leukemia, MESH:D015473	Arsenic Tr	15.9	263
SEMA4D	10507	Leukemia, MESH:D015473	Arsenic|ars	15.9	156
SGK3	23678	Leukemia, MESH:D015473	Arsenic|Ge	15.9	157
SH3BGRL3	83442	Leukemia, MESH:D015473	Arsenic Tr	15.9	262
ADM2	79924	Leukemia, MESH:D015470	Decitabine	15.89	15
BPI	671	Leukemia, MESH:D015473	Arsenic Tr	15.89	264
CBS	875	Leukemia, MESH:D015470	Gasoline|I	15.89	11
CCT4	10575	Leukemia, MESH:D015473	arsenite|D	15.89	157
CYP51	13121	Leukemia, MESH:D015470	Bezafibrat	15.89	13
FADD	8772	Leukemia, MESH:D015470	Decitabine	15.89	25
IL2RA	3559	Leukemia, MESH:D015473	Arsenic|Ca	15.89	13
NDUFV1	4723	Leukemia, MESH:D015470	Air Polluta	15.89	13
NEUROD1	4760	Leukemia, MESH:D015473	Arsenic|ars	15.89	157
PGP	283871	Leukemia, MESH:D015470	Air Polluta	15.89	16
SLC18A2	6571	Leukemia, MESH:D015473	arsenite|Ca	15.89	9
ZCCHC7	84186	Leukemia, MESH:D015473	arsenite|D	15.89	157
ADAM15	8751	Leukemia, MESH:D015470	Dexameth	15.88	12
ADPGK	83440	Leukemia, MESH:D015470	Dexameth	15.88	12
AGAP1	116987	Leukemia, MESH:D015470	Decitabine	15.88	15
BRD3	8019	Leukemia, MESH:D015470	Dexameth	15.88	9
CYP3A23-	25642	Leukemia, MESH:D015473	arsenite|Ca	15.88	9
FNDC1	84624	Leukemia, MESH:D015470	Arsenic Tr	15.88	20
HLA-DMB	3109	Leukemia, MESH:D015470	Benzene|B	15.88	22
IL17RA	23765	Leukemia, MESH:D015470	Air Polluta	15.88	26
KCNMB4	27345	Leukemia, MESH:D015470	Calcitriol|D	15.88	16
KPNA3	3839	Leukemia, MESH:D015470	Calcitriol|D	15.88	16
LRBA	987	Leukemia, MESH:D015473	arsenite|Cy	15.88	159
LYST	1130	Leukemia, MESH:D015470	Air Polluta	15.88	13
MICALL1	85377	Leukemia, MESH:D015470	Doxorubic	15.88	12
NDRG1	10397	Leukemia, MESH:D015473	Arsenic Tr	15.88	266
RFX5	5993	Leukemia, MESH:D015470	Dexameth	15.88	12
SH3BP2	6452	Leukemia, MESH:D015470	Doxorubic	15.88	13
SMARCC1	6599	Leukemia, MESH:D015470	Arsenic Tr	15.88	14
USO1	8615	Leukemia, MESH:D015470	Air Polluta	15.88	8
ARL6IP5	10550	Leukemia, MESH:D015473	Arsenic Tr	15.87	263
C1ORF112	55732	Leukemia, MESH:D015470	Calcitriol|D	15.87	9
CAPZB	832	Leukemia, MESH:D015470	Arsenic Tr	15.87	19
CASP9	842	Leukemia, MESH:D007948	2-(2-amin	15.87	3
CDR2	1039	Leukemia, MESH:D015470	Dexameth	15.87	8
FOXC2	2303	Leukemia, MESH:D015473	arsenite|ca	15.87	154
H4C12	8362	Leukemia, MESH:D015473	Arsenic|Ar	15.87	158
IFNA2	3440	Leukemia, MESH:D015470	Arsenic Tr	15.87	22
MAP7	9053	Leukemia, MESH:D015473	Arsenic Tr	15.87	259
MSRB3	253827	Leukemia, MESH:D015470	Arsenic Tr	15.87	13
NXN	64359	Leukemia, MESH:D015470	(+)-JQ1 co	15.87	10
OSER1	51526	Leukemia, MESH:D015470	Dexameth	15.87	12
PLA2G10	8399	Leukemia, MESH:D015470	Calcitriol|D	15.87	11
SPG7	6687	Leukemia, MESH:D015470	Dexameth	15.87	15
STIP1	10963	Leukemia, MESH:D015473	Arsenic Tr	15.87	158
TLK2	11011	Leukemia, MESH:D015470	Doxorubic	15.87	14
WDR4	10785	Leukemia, MESH:D015470	Dexameth	15.87	8
ATP8A1	10396	Leukemia, MESH:D015473	Arsenic|Ar	15.86	161
BAHD1	22893	Leukemia, MESH:D015470	Doxorubic	15.86	11
CD3D	915	Leukemia, MESH:D015473	Calcitriol|D	15.86	155
CELF1	10658	Leukemia, MESH:D015473	Arsenic|De	15.86	158

DEK	7913	Leukemia, MESH:D015470	Calcitriol|D	15.86	15
FAM174B	400451	Leukemia, MESH:D015470	Doxorubic	15.86	11
GABRB3	2562	Leukemia, MESH:D015470	Arsenic Tr	15.86	19
GRHL1	29841	Leukemia, MESH:D015473	Antimony	15.86	154
HDGFL2	84717	Leukemia, MESH:D015470	Decitabine	15.86	14
NSUN3	63899	Leukemia, MESH:D015470	Doxorubic	15.86	13
S100A11	6282	Leukemia, MESH:D015473	Arsenic|Ar	15.86	263
BTNL9	153579	Leukemia, MESH:D015470	Doxorubic	15.85	12
CD1D	912	Leukemia, MESH:D015473	Arsenic Tr	15.85	260
CTHRC1	115908	Leukemia, MESH:D015473	arsenite|Ca	15.85	155
DTX4	23220	Leukemia, MESH:D015473	Calcitriol|D	15.85	6
FLACC1	130540	Leukemia, MESH:D015470	Doxorubic	15.85	11
HROB	78995	Leukemia, MESH:D015470	Calcitriol|D	15.85	13
KLHL32	114792	Leukemia, MESH:D015473	Arsenic|Ar	15.85	158
LHX2	9355	Leukemia, MESH:D015473	arsenite|D	15.85	155
LIM2	3982	Leukemia, MESH:D015470	Alitretinoi	15.85	7
MDC1	9656	Leukemia, MESH:D015473	Arsenic|ars	15.85	156
MEIS1	4211	Leukemia, MESH:D015473	Arsenic|ars	15.85	158
MYL5	4636	Leukemia, MESH:D015470	Benzene|D	15.85	21
NCR1	9437	Leukemia, MESH:D015470	Bortezomi	15.85	8
OTUD5	55593	Leukemia, MESH:D015470	Doxorubic	15.85	15
PGD	5226	Leukemia, MESH:D015470	Arsenic Tr	15.85	17
PPP1R17	10842	Leukemia, MESH:D015470	Benzene|D	15.85	21
SLA	6503	Leukemia, MESH:D007948	Arsenic Tr	15.85	4
SLFN13	146857	Leukemia, MESH:D015470	Methotrex	15.85	10
TEKT2	27285	Leukemia, MESH:D015470	Dexameth	15.85	6
TGM4	7047	Leukemia, MESH:D015470	Calcitriol|D	15.85	7
TMTC2	160335	Leukemia, MESH:D015473	Arsenic|Ar	15.85	160
TMUB1	83590	Leukemia, MESH:D015470	(+)-JQ1 cc	15.85	6
TUBG1	7283	Leukemia, MESH:D015473	Arsenic Tr	15.85	262
UNK	85451	Leukemia, MESH:D015470	Decitabine	15.85	15
ZKSCAN7	55888	Leukemia, MESH:D015470	Calcitriol|D	15.85	7
ZSCAN2	54993	Leukemia, MESH:D015470	Arsenic Tr	15.85	24
ATG3	64422	Leukemia, MESH:D015473	Arsenic Tr	15.84	263
ATN1	1822	Leukemia, MESH:D004915	Cytarabine	15.84	2
BOLA2	552900	Leukemia, MESH:D015470	Doxorubic	15.84	12
CHD9	80205	Leukemia, MESH:D015473	arsenite|Ca	15.84	155
CLCA1	1179	Leukemia, MESH:D015473	Arsenic|De	15.84	11
FSTL5	56884	Leukemia, MESH:D015470	Arsenic Tr	15.84	20
KREMEN1	83999	Leukemia, MESH:D015470	Calcitriol|D	15.84	12
LRRTM1	347730	Leukemia, MESH:D015470	Indometha	15.84	7
PITPNC1	26207	Leukemia, MESH:D015473	Arsenic|Ar	15.84	263
PLIN4	729359	Leukemia, MESH:D015473	arsenite|D	15.84	8
RAD51B	5890	Leukemia, MESH:D015473	Arsenic|ars	15.84	157
SOX11	6664	Leukemia, MESH:D015473	Arsenic|ars	15.84	156
SPICE1	152185	Leukemia, MESH:D015470	Arsenic Tr	15.84	17
SPPL3	121665	Leukemia, MESH:D015470	Dexameth	15.84	11
TMEM59L	25789	Leukemia, MESH:D015470	Arsenic Tr	15.84	12
ZNF143	7702	Leukemia, MESH:D015470	Doxorubic	15.84	13
ZNF281	23528	Leukemia, MESH:D015470	Arsenic Tr	15.84	13
ACSF3	197322	Leukemia, MESH:D015470	Doxorubic	15.83	12
DNMT1	1786	Leukemia, MESH:D004915	Doxorubic	15.83	3
GAS6	2621	Leukemia, MESH:D015473	caffeic aci	15.83	155
GOLGA7	51125	Leukemia, MESH:D015470	Dexameth	15.83	11
GSTO2	119391	Leukemia, MESH:D015473	Arsenic|Ar	15.83	263
PPFIA4	8497	Leukemia, MESH:D015473	Arsenic|ars	15.83	157
SSBP2	23635	Leukemia, MESH:D015473	Arsenic Tr	15.83	260
SULT1E1	6783	Leukemia, MESH:D015473	Antimony	15.83	8

TOPBP1	11073	Leukemia, MESH:D015473	Calcitriol|I	15.83	29
PFKFB3	5209	Leukemia, MESH:D015473	Arsenic|De	15.82	158
ANG	283	Leukemia, MESH:D015470	Decitabine	15.81	16
AP1S2	8905	Leukemia, MESH:D015473	Dexameth	15.81	156
GLIPR2	152007	Leukemia, MESH:D015473	arsenite|Ca	15.81	155
PTPN21	11099	Leukemia, MESH:D007948	Arsenic Tr	15.81	3
SKP1	6500	Leukemia, MESH:D015473	Arsenic|ars	15.81	11
ALS2	57679	Leukemia, MESH:D015470	Arsenic Tr	15.8	20
ATG10	83734	Leukemia, MESH:D015470	Dexameth	15.8	9
CSTB	1476	Leukemia, MESH:D015470	Arsenic Tr	15.8	19
FABP6	2172	Leukemia, MESH:D015470	Dronabinc	15.8	10
NAPEPLD	222236	Leukemia, MESH:D015470	Air Polluta	15.8	8
PCMTD1	115294	Leukemia, MESH:D015470	Dasatinib|	15.8	10
PCYT1A	5130	Leukemia, MESH:D015470	Bezafibrat	15.8	16
PLOD1	5351	Leukemia, MESH:D015470	Cyclophos	15.8	79
POLR1C	9533	Leukemia, MESH:D015470	Dexameth	15.8	16
PROSER2	254427	Leukemia, MESH:D015470	Calcitriol|C	15.8	9
BST1	683	Leukemia, MESH:D015470	Dexameth	15.79	10
CHST3	9469	Leukemia, MESH:D015470	Calcitriol|D	15.79	12
CPSF6	11052	Leukemia, MESH:D015470	Doxorubic	15.79	16
CTDSPL	10217	Leukemia, MESH:D015470	Dexameth	15.79	7
FBP1	2203	Leukemia, MESH:D015473	Arsenic Tr	15.79	266
IFT81	28981	Leukemia, MESH:D004915	Doxorubic	15.79	2
LRIG3	121227	Leukemia, MESH:D015470	Calcitriol|D	15.79	13
MAN2A1	4124	Leukemia, MESH:D015473	Arsenic|ars	15.79	156
MIR22	407004	Leukemia, MESH:D015473	Calcitriol|M	15.79	155
PDZRN3	23024	Leukemia, MESH:D015470	Calcitriol|D	15.79	8
PPIC	5480	Leukemia, MESH:D015473	Calcitriol|C	15.79	156
RAB38	23682	Leukemia, MESH:D015470	Dexameth	15.79	10
REV3L	5980	Leukemia, MESH:D015470	Dexameth	15.79	13
RPS25	6230	Leukemia, MESH:D015470	Arsenic Tr	15.79	18
SERINC5	256987	Leukemia, MESH:D015470	(+)-JQ1 cc	15.79	11
SLC25A29	123096	Leukemia, MESH:D015470	Indometha	15.79	8
TSPAN4	7106	Leukemia, MESH:D015473	Arsenic Tr	15.79	263
AFAP1	60312	Leukemia, MESH:D015473	Arsenic|Ar	15.78	265
CD9	928	Leukemia, MESH:D015473	Arsenic|Ar	15.78	263
DSTN	11034	Leukemia, MESH:D015473	arsenite|G	15.78	156
FTH1	2495	Leukemia, MESH:D004915	Doxorubic	15.78	3
LSR	51599	Leukemia, MESH:D015473	Arsenic Tr	15.78	261
MMP15	4324	Leukemia, MESH:D015473	Arsenic|Ge	15.78	155
MYO5B	4645	Leukemia, MESH:D015473	Arsenic Tr	15.78	261
SEMA7A	8482	Leukemia, MESH:D015473	Arsenic Tr	15.78	260
SMIM3	85027	Leukemia, MESH:D015473	Calcitriol|D	15.78	154
TICAM1	148022	Leukemia, MESH:D015473	Dexameth	15.78	7
ADCY1	107	Leukemia, MESH:D015473	Arsenic Tr	15.77	262
NPIPA1	9284	Leukemia, MESH:D015473	Dexameth	15.77	155
RUVBL1	8607	Leukemia, MESH:D015473	Arsenic Tr	15.77	262
UBA7	7318	Leukemia, MESH:D015473	arsenic dis	15.77	158
ATP5ME	521	Leukemia, MESH:D004915	Doxorubic	15.76	3
BRAF	673	Leukemia, MESH:D015473	Arsenic|Ar	15.76	264
DLEU2L	79469	Leukemia, MESH:D015473	arsenic dis	15.76	6
OPN1MW	728458	Leukemia, MESH:D015470	Alitretinoi	15.76	7
PIK3R2	5296	Leukemia, MESH:D015473	Antimony	15.76	155
STK10	6793	Leukemia, MESH:D015473	arsenite|D	15.76	153
ADAM17	6868	Leukemia, MESH:D015470	Dexameth	15.75	11
ATM	472	Leukemia, MESH:D004915	Daunorub	15.75	2
HES6	55502	Leukemia, MESH:D015470	Dexameth	15.75	13
RDH2	107460	Leukemia, MESH:D015470	Dexameth	15.75	7

RUVBL2	10856	Leukemia, MESH:D015473	Arsenic Tr	15.75	263
IL23R	149233	Leukemia, MESH:D015470	(+)-JQ1 cc	15.74	11
MIR185	406961	Leukemia, MESH:D015470	Dronabinc	15.74	8
NRXN3	9369	Leukemia, MESH:D015473	Arsenic Tr	15.74	260
PRKCI	5584	Leukemia, MESH:D015473	arsenite|D	15.74	159
THSD7B	80731	Leukemia, MESH:D015473	Arsenic|Ar	15.74	261
YLPM1	56252	Leukemia, MESH:D004915	Doxorubic	15.74	2
ARHGAP2	58504	Leukemia, MESH:D015473	Dasatinib|	15.73	153
ARHGEF7	8874	Leukemia, MESH:D015473	Arsenic|ars	15.73	157
CD22	933	Leukemia, MESH:D015473	Arsenic Tr	15.73	264
EN2	2020	Leukemia, MESH:D015470	Etoposide	15.73	24
MOSMO	730094	Leukemia, MESH:D015473	Arsenic|Ca	15.73	154
SCG2	7857	Leukemia, MESH:D015473	Arsenic Tr	15.73	260
TFEC	22797	Leukemia, MESH:D015470	Air Polluta	15.73	17
TLR5	7100	Leukemia, MESH:D007948	2-(2-amin	15.73	2
XXYLT1	152002	Leukemia, MESH:D015470	Bortezomi	15.73	8
ZNF559	84527	Leukemia, MESH:D015470	Air Polluta	15.73	13
ABCF2	10061	Leukemia, MESH:D015470	Air Polluta	15.72	12
CARS1	833	Leukemia, MESH:D015473	Arsenic Tr	15.72	154
CCNC	892	Leukemia, MESH:D015473	Arsenic|Ca	15.72	156
CMC2	56942	Leukemia, MESH:D015470	Daunorub	15.72	35
CYP19A1	1588	Leukemia, MESH:D007948	2-(2-amin	15.72	2
GCOM1	145781	Leukemia, MESH:D015470	Air Polluta	15.72	13
GPR146	115330	Leukemia, MESH:D015470	(+)-JQ1 cc	15.72	9
HOMER2	9455	Leukemia, MESH:D015470	Arsenic Tr	15.72	20
HSH2D	84941	Leukemia, MESH:D015470	Calcitriol|(	15.72	10
IL1RL1	9173	Leukemia, MESH:D015473	Arsenic|Ca	15.72	156
IL6ST	3572	Leukemia, MESH:D015473	Arsenic Tr	15.72	262
NDUFA7	4701	Leukemia, MESH:D015470	Decitabine	15.72	16
NPPC	4880	Leukemia, MESH:D015470	Calcitriol|C	15.72	9
PCM1	5108	Leukemia, MESH:D015473	Arsenic Tr	15.72	262
PIGB	9488	Leukemia, MESH:D015473	Arsenic|M	15.72	154
PNKD	25953	Leukemia, MESH:D015470	Calcitriol|D	15.72	15
PPP3CC	5533	Leukemia, MESH:D015470	Air Polluta	15.72	21
RAB4B	53916	Leukemia, MESH:D015470	Calcitriol|D	15.72	11
RDH13	112724	Leukemia, MESH:D015470	Bortezomi	15.72	9
SLC12A4	6560	Leukemia, MESH:D015473	Genistein|	15.72	154
SPHKAP	80309	Leukemia, MESH:D015470	Decitabine	15.72	19
TENT2	167153	Leukemia, MESH:D015470	Arsenic Tr	15.72	18
TMOD2	29767	Leukemia, MESH:D015470	Dexameth	15.72	7
TNFAIP8L1	126282	Leukemia, MESH:D015470	Bortezomi	15.72	12
U2AF1L4	199746	Leukemia, MESH:D015470	Dexameth	15.72	11
VIPR1	7433	Leukemia, MESH:D015473	Arsenic|ars	15.72	155
ZFAND5	7763	Leukemia, MESH:D015473	Arsenic Tr	15.72	261
ARF6	382	Leukemia, MESH:D015470	Bortezomi	15.71	14
ARMCX3	51566	Leukemia, MESH:D015470	Arsenic Tr	15.71	15
ARSD	414	Leukemia, MESH:D015470	Doxorubic	15.71	14
CCN4	8840	Leukemia, MESH:D007948	Arsenic Tr	15.71	3
CLEC2B	9976	Leukemia, MESH:D015470	Decitabine	15.71	14
CPEB3	22849	Leukemia, MESH:D015470	Calcitriol|D	15.71	13
CSNK1D	1453	Leukemia, MESH:D007948	Arsenic Tr	15.71	3
CTU2	348180	Leukemia, MESH:D015470	Calcitriol|D	15.71	11
DGAT1A	325875	Leukemia, MESH:D015470	Alitretinoi	15.71	7
FEZ2	9637	Leukemia, MESH:D015470	Air Polluta	15.71	13
FSTL3	10272	Leukemia, MESH:D015470	Doxorubic	15.71	12
HENMT1	113802	Leukemia, MESH:D015470	Arsenic Tr	15.71	12
IFT80	57560	Leukemia, MESH:D015470	Doxorubic	15.71	13
INVS	27130	Leukemia, MESH:D015470	Dexameth	15.71	13

KITL	17311	Leukemia, MESH:D015470	Cyclophos	15.71	10
LCLAT1	253558	Leukemia, MESH:D015470	Dronabinc	15.71	10
LIN9	286826	Leukemia, MESH:D015470	Calcitriol|(	15.71	7
MIPEP	4285	Leukemia, MESH:D015470	Dexameth	15.71	8
NBPF10	1E+08	Leukemia, MESH:D015473	Calcitriol|D	15.71	153
NPC1L1	29881	Leukemia, MESH:D015470	Dexameth	15.71	10
NXPH1	30010	Leukemia, MESH:D015470	Dexameth	15.71	7
ODF2	4957	Leukemia, MESH:D015470	Dexameth	15.71	13
PCDH9	5101	Leukemia, MESH:D015470	Dexameth	15.71	13
RCBTB2	1102	Leukemia, MESH:D015470	Air Polluta	15.71	8
RENBP	5973	Leukemia, MESH:D015470	Decitabine	15.71	15
SEC23A	10484	Leukemia, MESH:D015470	Cyclophos	15.71	13
SENP8	123228	Leukemia, MESH:D015470	Doxorubic	15.71	11
SFT2D1	113402	Leukemia, MESH:D015470	Air Polluta	15.71	7
SMAD5	4090	Leukemia, MESH:D015473	Arsenic|De	15.71	158
SNX15	29907	Leukemia, MESH:D015470	Dexameth	15.71	6
SYPL2	284612	Leukemia, MESH:D015470	Dexameth	15.71	14
TENT4B	64282	Leukemia, MESH:D015470	(+)-JQ1 cc	15.71	6
TMEM176	28959	Leukemia, MESH:D015470	Calcitriol|D	15.71	15
ABHD17C	58489	Leukemia, MESH:D015470	Dexameth	15.7	6
AKT1	207	Leukemia, MESH:D007948	2-(2-amin	15.7	3
AMBP	259	Leukemia, MESH:D015470	Air Polluta	15.7	19
CHSY3	337876	Leukemia, MESH:D015470	Doxorubic	15.7	11
CTIF	9811	Leukemia, MESH:D015470	Doxorubic	15.7	12
FLRT1	23769	Leukemia, MESH:D015470	Dronabinc	15.7	7
FOCAD	54914	Leukemia, MESH:D015470	Calcitriol|D	15.7	12
GSTM5	2949	Leukemia, MESH:D015470	Arsenic Tr	15.7	22
LMBR1L	55716	Leukemia, MESH:D015470	Dexameth	15.7	7
MTMR12	54545	Leukemia, MESH:D015470	Dexameth	15.7	11
NOL3	8996	Leukemia, MESH:D015470	Arsenic Tr	15.7	21
PDZRN4	29951	Leukemia, MESH:D015473	Arsenic Tr	15.7	260
PSMG3	84262	Leukemia, MESH:D015470	Doxorubic	15.7	13
STK33	65975	Leukemia, MESH:D015470	Doxorubic	15.7	14
TUBGCP6	85378	Leukemia, MESH:D015470	Dexameth	15.7	7
UFC1	51506	Leukemia, MESH:D015470	Dexameth	15.7	11
ULK3	25989	Leukemia, MESH:D015470	Dexameth	15.7	7
ACAT1	38	Leukemia, MESH:D015473	alpha-Toc	15.69	157
CORO1A	11151	Leukemia, MESH:D015470	Arsenic Tr	15.69	18
HECTD3	79654	Leukemia, MESH:D015470	Doxorubic	15.69	12
PNN	5411	Leukemia, MESH:D015473	arsenite|G	15.69	158
SORD	6652	Leukemia, MESH:D015473	arsenite|Bu	15.69	156
TNF	7124	Leukemia, MESH:D007948	2-(2-amin	15.69	4
TRO	7216	Leukemia, MESH:D015470	Dexameth	15.69	12
ZNF189	7743	Leukemia, MESH:D015473	Dexameth	15.69	5
ACO1	48	Leukemia, MESH:D015473	Arsenic|Ar	15.68	263
DEFA1	1667	Leukemia, MESH:D015470	Arsenic Tr	15.68	11
DPP7	29952	Leukemia, MESH:D015473	Arsenic|ars	15.68	14
GALNT3	2591	Leukemia, MESH:D007948	2-(2-amin	15.68	2
GASK1B	51313	Leukemia, MESH:D015473	Calcitriol|D	15.68	153
KMT2B	9757	Leukemia, MESH:D004915	Doxorubic	15.68	2
NR3C2	4306	Leukemia, MESH:D015470	Dexameth	15.68	17
NUP153	9972	Leukemia, MESH:D007948	2-(2-amin	15.68	3
PSMC3IP	29893	Leukemia, MESH:D015473	Calcitriol|D	15.68	155
ROR1	4919	Leukemia, MESH:D015473	Arsenic|Ca	15.68	154
COL13A1	1305	Leukemia, MESH:D015473	Calcitriol|C	15.67	156
FLOT1	10211	Leukemia, MESH:D015473	Dexameth	15.67	159
ITPK1-AS1	319085	Leukemia, MESH:D015470	Gasoline|N	15.67	7
LARP1B	55132	Leukemia, MESH:D015473	Calcitriol|D	15.67	154

MCCC2	64087	Leukemia, MESH:D015473	Dexameth	15.67	154
NUAK2	81788	Leukemia, MESH:D015473	Arsenic|Ar	15.67	159
C1ORF74	148304	Leukemia, MESH:D015470	Dronabinc	15.66	8
CAPNS1	826	Leukemia, MESH:D015473	Arsenic Tr	15.66	156
CFTR	1080	Leukemia, MESH:D015473	alpha-Toc	15.66	160
GSTT1	2952	Leukemia, MESH:D015473	alpha-Toc	15.66	161
PEA15	8682	Leukemia, MESH:D015473	Arsenic|De	15.66	156
SEL1L3	23231	Leukemia, MESH:D015473	Arsenic Tr	15.66	262
WEE1	7465	Leukemia, MESH:D015473	Arsenic Tr	15.66	266
ZNF2	7549	Leukemia, MESH:D015470	Dexameth	15.66	9
ATRN	8455	Leukemia, MESH:D015470	Benzene|C	15.65	21
GUSBP14	11039	Leukemia, MESH:D015470	Dexameth	15.65	7
ING3	54556	Leukemia, MESH:D015470	Doxorubic	15.65	12
KRT80	144501	Leukemia, MESH:D015470	Calcitriol|C	15.65	15
PITX1	5307	Leukemia, MESH:D004915	Cytarabine	15.65	3
APLP1	333	Leukemia, MESH:D015470	Doxorubic	15.64	23
CES2	8824	Leukemia, MESH:D015470	Dexameth	15.64	25
FCGRT	2217	Leukemia, MESH:D015470	Cyclophos	15.64	8
GSS	2937	Leukemia, MESH:D015470	Dronabinc	15.64	13
HECTD1	25831	Leukemia, MESH:D015470	Dexameth	15.64	8
IGTP	16145	Leukemia, MESH:D015470	Benzene|D	15.64	20
KAT6A	7994	Leukemia, MESH:D015470	Benzene|(	15.64	22
KRT1-19D	664718	Leukemia, MESH:D015470	Dexameth	15.64	5
MAPT	4137	Leukemia, MESH:D004915	Daunorub	15.64	3
MIR362	574030	Leukemia, MESH:D015473	Buthionine	15.64	155
MIS18BP1	55320	Leukemia, MESH:D015470	Calcitriol|D	15.64	9
MPZL1	9019	Leukemia, MESH:D015473	Cytarabine	15.64	153
NAGA	4668	Leukemia, MESH:D015470	Air Polluta	15.64	8
NDUFB3	4709	Leukemia, MESH:D015470	Doxorubic	15.64	13
NLN	57486	Leukemia, MESH:D015470	Air Polluta	15.64	13
NPM3	10360	Leukemia, MESH:D015470	Gasoline|(	15.64	8
PKD2	5311	Leukemia, MESH:D015470	Decitabine	15.64	20
PMS2P5	5383	Leukemia, MESH:D015470	Arsenic Tr	15.64	12
RCL1	10171	Leukemia, MESH:D015470	Air Polluta	15.64	18
SRRT	51593	Leukemia, MESH:D015470	Arsenic Tr	15.64	15
TFB1M	51106	Leukemia, MESH:D015470	Doxorubic	15.64	13
TNRC6A	27327	Leukemia, MESH:D015470	Cytarabine	15.64	78
ACOT13	55856	Leukemia, MESH:D015470	Decitabine	15.63	20
ATP6V1C1	528	Leukemia, MESH:D015470	Air Polluta	15.63	12
BMPR1A	657	Leukemia, MESH:D015470	Dexameth	15.63	8
CHAC2	494143	Leukemia, MESH:D015470	Calcitriol|D	15.63	14
EDN2	1907	Leukemia, MESH:D015470	Dexameth	15.63	10
GPX1B	447895	Leukemia, MESH:D015470	Dronabinc	15.63	6
ISL1	3670	Leukemia, MESH:D015470	Dexameth	15.63	9
KRTAP2-3	730755	Leukemia, MESH:D015470	(+)-JQ1 cc	15.63	6
MLLT3	4300	Leukemia, MESH:D015470	Doxorubic	15.63	16
NPNT	255743	Leukemia, MESH:D015470	Doxorubic	15.63	21
PER1	5187	Leukemia, MESH:D015473	Arsenic|De	15.63	153
PKMYT1	9088	Leukemia, MESH:D015473	Arsenic Tr	15.63	261
SFXN3	81855	Leukemia, MESH:D015470	Calcitriol|D	15.63	16
SPRR1A	6698	Leukemia, MESH:D015473	Antimony	15.63	153
ST3GAL2	6483	Leukemia, MESH:D015470	Arsenic Tr	15.63	18
ST3GAL3	6487	Leukemia, MESH:D015470	Doxorubic	15.63	14
SUV39H2	79723	Leukemia, MESH:D015470	Calcitriol|D	15.63	10
TRIM14	9830	Leukemia, MESH:D015470	Doxorubic	15.63	13
WDR12	55759	Leukemia, MESH:D015470	Arsenic Tr	15.63	13
XRCC3	7517	Leukemia, MESH:D015473	Arsenic|Ar	15.63	161
ABCB8	11194	Leukemia, MESH:D015470	Daunorub	15.62	32

FOXN3-AS	400236	Leukemia, MESH:D015470	Dexameth	15.62	6
GLIPR1L2	144321	Leukemia, MESH:D015470	Dexameth	15.62	5
LRCH3	84859	Leukemia, MESH:D015473	Arsenic|ars	15.62	11
N6AMT2	1E+08	Leukemia, MESH:D015470	Dexameth	15.62	6
NOS2	4843	Leukemia, MESH:D007948	2-(2-amin	15.62	3
PDXDC2P	1.1E+08	Leukemia, MESH:D015470	Dexameth	15.62	5
PRSS12	8492	Leukemia, MESH:D015473	Arsenic|ca	15.62	155
RAD51-AS	1.01E+08	Leukemia, MESH:D015470	Dexameth	15.62	5
RPS14	6208	Leukemia, MESH:D015473	arsenite|Bu	15.62	155
RPTOR	57521	Leukemia, MESH:D015473	Arsenic|Ch	15.62	12
SIVA1	10572	Leukemia, MESH:D015473	Arsenic|ars	15.62	12
TTTY14	83869	Leukemia, MESH:D015470	Dexameth	15.62	5
ZSWIM9	374920	Leukemia, MESH:D015470	Dexameth	15.62	5
CHRNA5	1138	Leukemia, MESH:D015473	Dexameth	15.61	153
COA8	84334	Leukemia, MESH:D015470	Doxorubic	15.61	11
DSG2	1829	Leukemia, MESH:D015473	Arsenic Tr	15.61	262
DUSP14	11072	Leukemia, MESH:D015473	Arsenic|ars	15.61	156
FAM107A	11170	Leukemia, MESH:D015473	Arsenic|ars	15.61	11
KLHDC7B	113730	Leukemia, MESH:D015473	Calcitriol|D	15.61	5
LINC0100	1E+08	Leukemia, MESH:D015470	Tobacco S	15.61	6
MALT1	10892	Leukemia, MESH:D015473	Arsenic|Ca	15.61	155
MECOM	2122	Leukemia, MESH:D015473	Arsenic|Ar	15.61	264
OSM	5008	Leukemia, MESH:D007948	2-(2-amin	15.61	1
SPRR2G	6706	Leukemia, MESH:D015473	Arsenic|Ar	15.61	261
BMPER	168667	Leukemia, MESH:D015473	Arsenic|De	15.6	155
CABLES1	91768	Leukemia, MESH:D015473	arsenite|D	15.6	155
CEP72	55722	Leukemia, MESH:D015473	Arsenic|Ca	15.6	155
GNG7	2788	Leukemia, MESH:D015473	Arsenic|ars	15.6	157
HES5	388585	Leukemia, MESH:D015473	2-(2-chlor	15.6	264
HYPK	25764	Leukemia, MESH:D015470	Dexameth	15.6	6
KLF3	51274	Leukemia, MESH:D015473	Arsenic Tr	15.6	262
PCDHGA8	9708	Leukemia, MESH:D015470	Doxorubic	15.6	11
RPL32	6161	Leukemia, MESH:D015473	Arsenic|ars	15.6	13
SRXN1	140809	Leukemia, MESH:D004915	Cytarabine	15.6	2
TRAF4	9618	Leukemia, MESH:D015473	Arsenic Tr	15.6	261
UCA1	652995	Leukemia, MESH:D015473	Arsenic|Ca	15.6	155
DNAI3	126820	Leukemia, MESH:D015470	Arsenic Tr	15.59	18
FBXO38	81545	Leukemia, MESH:D015470	Air Polluta	15.59	13
FUNDC1	139341	Leukemia, MESH:D015470	Chloroqui	15.59	11
GGA1	26088	Leukemia, MESH:D015470	Arsenic Tr	15.59	31
HYOU1	10525	Leukemia, MESH:D015470	Cyclophos	15.59	14
ILKAP	80895	Leukemia, MESH:D015470	Arsenic Tr	15.59	38
LEMD2	221496	Leukemia, MESH:D015470	Dexameth	15.59	20
SETDB2	83852	Leukemia, MESH:D015470	Decitabine	15.59	21
TTC13	79573	Leukemia, MESH:D015470	Doxorubic	15.59	11
WDR82	80335	Leukemia, MESH:D015473	Arsenic|Ar	15.59	263
XAB2	56949	Leukemia, MESH:D015470	Decitabine	15.59	23
ZDHHC16	84287	Leukemia, MESH:D015470	Air Polluta	15.59	18
CSNK1G3	1456	Leukemia, MESH:D004915	Doxorubic	15.58	2
DIRAS3	9077	Leukemia, MESH:D015470	Arsenic Tr	15.58	18
ESPN	83715	Leukemia, MESH:D015470	Androgen	15.58	14
GLB1L2	89944	Leukemia, MESH:D015470	Benzene|T	15.58	22
GPATCH1	253635	Leukemia, MESH:D015470	Dexameth	15.58	11
MSANTD4	84437	Leukemia, MESH:D015470	Dexameth	15.58	6
PBX4	80714	Leukemia, MESH:D015470	Indometha	15.58	7
SAMD13	148418	Leukemia, MESH:D015470	Calcitriol|(	15.58	6
SDS	10993	Leukemia, MESH:D015473	caffeic aci	15.58	153
THBS4	7060	Leukemia, MESH:D015473	arsenite|Et	15.58	153

TMEM192	201931	Leukemia, MESH:D015470	Doxorubic	15.58	11
TUBG2	27175	Leukemia, MESH:D015470	Arsenic Tr	15.58	25
UNC5B	219699	Leukemia, MESH:D015470	Dexameth	15.58	10
ATG101	60673	Leukemia, MESH:D015470	Dronabinc	15.57	8
ATMIN	23300	Leukemia, MESH:D015470	Doxorubic	15.57	13
CCDC148	130940	Leukemia, MESH:D015470	Doxorubic	15.57	11
CINP	51550	Leukemia, MESH:D015470	Dexameth	15.57	11
CNPY1	285888	Leukemia, MESH:D015470	Dexameth	15.57	6
FBXO43	286151	Leukemia, MESH:D015470	Pentachlo	15.57	7
GTF2IRD2	84163	Leukemia, MESH:D015470	Dexameth	15.57	6
KIRREL2	84063	Leukemia, MESH:D015470	Dronabinc	15.57	8
KLHL31	401265	Leukemia, MESH:D015470	Doxorubic	15.57	11
LRWD1	222229	Leukemia, MESH:D015470	Dexameth	15.57	7
MYL2	4633	Leukemia, MESH:D015470	Azacitidine	15.57	24
NR2C2AP	126382	Leukemia, MESH:D015470	Calcitriol|(	15.57	7
SYS1	90196	Leukemia, MESH:D015470	Doxorubic	15.57	13
TMCO3	55002	Leukemia, MESH:D015470	Calcitriol|D	15.57	7
TTLL11	158135	Leukemia, MESH:D015470	Dexameth	15.57	11
TYW3	127253	Leukemia, MESH:D015470	Arsenic Tr	15.57	17
USE1	55850	Leukemia, MESH:D015470	Doxorubic	15.57	13
VAV1	7409	Leukemia, MESH:D015470	Air Polluta	15.57	19
ZNF618	114991	Leukemia, MESH:D015470	(+)-JQ1 cc	15.57	7
ACAD9	28976	Leukemia, MESH:D015473	Arsenic|ars	15.56	12
ALOX5	240	Leukemia, MESH:D015473	Arsenic|Ca	15.56	159
APPL2	55198	Leukemia, MESH:D015470	Air Polluta	15.56	13
ATP11A	23250	Leukemia, MESH:D015470	Doxorubic	15.56	14
BIRC3	330	Leukemia, MESH:D004915	Doxorubic	15.56	3
COX8A	1351	Leukemia, MESH:D015470	Bortezomi	15.56	15
CTSA	5476	Leukemia, MESH:D015473	Arsenic Tr	15.56	264
DERL3	91319	Leukemia, MESH:D015470	Calcitriol|E	15.56	9
DYRK3	8444	Leukemia, MESH:D015470	Dronabinc	15.56	11
IRF2	3660	Leukemia, MESH:D015470	Air Polluta	15.56	13
PRMT3	10196	Leukemia, MESH:D015470	Dexameth	15.56	14
PSPC1	55269	Leukemia, MESH:D015473	Arsenic Tr	15.56	155
TBC1D2	55357	Leukemia, MESH:D015470	Calcitriol|C	15.56	9
UCP2	7351	Leukemia, MESH:D004915	Doxorubic	15.56	3
ZNF430	80264	Leukemia, MESH:D015470	Dexameth	15.56	7
ZNF622	90441	Leukemia, MESH:D015470	Dexameth	15.56	7
AGER	177	Leukemia, MESH:D015473	Arsenic|Ca	15.55	12
CD4	920	Leukemia, MESH:D015473	Dexameth	15.55	155
CFB	629	Leukemia, MESH:D015473	Antimony	15.55	263
COX7A1	1346	Leukemia, MESH:D015470	Dexameth	15.55	13
GALNT6	11226	Leukemia, MESH:D015470	Calcitriol|D	15.55	13
HACD1	9200	Leukemia, MESH:D015470	Dexameth	15.55	13
IGF1R	3480	Leukemia, MESH:D004915	Doxorubic	15.55	3
MBOAT1	154141	Leukemia, MESH:D015470	Dronabinc	15.55	8
MTCH2	23788	Leukemia, MESH:D015470	Dexameth	15.55	14
NDUFB8	4714	Leukemia, MESH:D015473	Arsenic|ars	15.55	13
NEIL3	55247	Leukemia, MESH:D015473	Calcitriol|N	15.55	155
NUDT4	11163	Leukemia, MESH:D015473	Arsenic|Ar	15.55	261
PCCA	5095	Leukemia, MESH:D015473	Arsenic Tr	15.55	262
PMM2	5373	Leukemia, MESH:D015470	Arsenic Tr	15.55	19
PRAG1	157285	Leukemia, MESH:D015470	Dexameth	15.55	12
PRKN	5071	Leukemia, MESH:D015473	Arsenic|Ar	15.55	264
PSME3	10197	Leukemia, MESH:D015473	Arsenic Tr	15.55	156
RMDN2	151393	Leukemia, MESH:D015470	Arsenic Tr	15.55	13
SERPINB1/	66222	Leukemia, MESH:D015470	Dexameth	15.55	9
SRR	63826	Leukemia, MESH:D015473	Arsenic Tr	15.55	261

SUCLG1	8802	Leukemia, MESH:D015473	Arsenic|ars	15.55	13
TBC1D15	64786	Leukemia, MESH:D015470	Arsenic Tr	15.55	13
VAPB	9217	Leukemia, MESH:D015470	Doxorubic	15.55	14
ADRB2	154	Leukemia, MESH:D015473	arsenite|Ca	15.54	156
ATP6V0D1	9114	Leukemia, MESH:D015473	Arsenic|Ar	15.54	263
FXN	2395	Leukemia, MESH:D015473	arsenite|D	15.54	157
MIR576	693161	Leukemia, MESH:D015473	Arsenic|Re	15.54	10
SPINK2	6691	Leukemia, MESH:D015473	Arsenic Tr	15.54	262
SRR	63826	Leukemia, MESH:D007948	Arsenic Tr	15.54	3
STRA6	64220	Leukemia, MESH:D015473	Arsenic Tr	15.54	262
MNT	4335	Leukemia, MESH:D004915	Doxorubic	15.53	2
SP110	3431	Leukemia, MESH:D004915	Daunorub	15.53	2
ST6GAL1	6480	Leukemia, MESH:D015470	Decitabine	15.53	16
TLR4	7099	Leukemia, MESH:D007948	2-(2-amin	15.53	3
CLTB	1212	Leukemia, MESH:D015473	Arsenic Tr	15.52	264
DAAM1	23002	Leukemia, MESH:D015473	Arsenic|ars	15.52	159
IFT43	112752	Leukemia, MESH:D015473	arsenite|D	15.52	8
LEP	3952	Leukemia, MESH:D007948	2-(2-amin	15.52	3
MAT2AL	678634	Leukemia, MESH:D015473	Dexameth	15.52	4
SMOC2	64094	Leukemia, MESH:D015473	Cytarabine	15.52	155
CDC34	997	Leukemia, MESH:D007948	Arsenic Tr	15.51	3
CIITA	4261	Leukemia, MESH:D015473	Arsenic|Ar	15.51	267
CX3CL1	6376	Leukemia, MESH:D015473	Arsenic Tr	15.51	263
FLRT2	23768	Leukemia, MESH:D015473	arsenite|Cy	15.51	16
GTF3C3	9330	Leukemia, MESH:D015473	arsenite|Re	15.51	155
NUAK1	9891	Leukemia, MESH:D015473	Arsenic|Ar	15.51	263
PPARGC1A	10891	Leukemia, MESH:D007948	Arsenic Tr	15.51	3
YES1	7525	Leukemia, MESH:D015470	Arsenic Tr	15.51	13
AUH	549	Leukemia, MESH:D015473	Dexameth	15.5	7
CDC34	997	Leukemia, MESH:D015473	Arsenic Tr	15.5	155
CDKN1B	1027	Leukemia, MESH:D007948	2-(2-amin	15.5	3
HEPH	9843	Leukemia, MESH:D015473	Dasatinib|I	15.5	7
INS	3630	Leukemia, MESH:D007948	2-(2-amin	15.5	2
MINDY4	84182	Leukemia, MESH:D015473	Arsenic|Cy	15.5	157
MIR494	574452	Leukemia, MESH:D015470	Benzene|(	15.5	21
STEAP3	55240	Leukemia, MESH:D015473	Arsenic|Ca	15.5	155
TFAP2C	7022	Leukemia, MESH:D015473	Arsenic Tr	15.5	156
TMEM158	25907	Leukemia, MESH:D015473	Arsenic|Ar	15.5	262
UBL3	5412	Leukemia, MESH:D004915	Cytarabine	15.5	2
COMMD1	150684	Leukemia, MESH:D015470	Clioquinol	15.49	12
EPB41L5	57669	Leukemia, MESH:D015473	Arsenic|ars	15.49	159
GJC1	10052	Leukemia, MESH:D015470	Chloroqui	15.49	17
KCNB1	3745	Leukemia, MESH:D015473	Arsenic|De	15.49	156
LPGAT1	9926	Leukemia, MESH:D015473	Calcitriol|D	15.49	156
MUC2	4583	Leukemia, MESH:D015473	Arsenic|Ge	15.49	155
RRP12	23223	Leukemia, MESH:D015473	Dexameth	15.49	153
SRF	6722	Leukemia, MESH:D015470	Arsenic Tr	15.49	17
CDK13	8621	Leukemia, MESH:D004915	Doxorubic	15.48	2
COX1	4512	Leukemia, MESH:D015473	Arsenic Tr	15.48	155
EN1	2019	Leukemia, MESH:D015473	arsenite|D	15.48	155
IL36A	27179	Leukemia, MESH:D015473	Arsenic|Ar	15.48	160
NACA	4666	Leukemia, MESH:D015470	Arsenic Tr	15.48	23
NRTN	4902	Leukemia, MESH:D015470	Arsenic Tr	15.48	19
NTF3	4908	Leukemia, MESH:D015470	Benzene|C	15.48	32
PAX9	5083	Leukemia, MESH:D015473	Arsenic Tr	15.48	261
PCBP2	5094	Leukemia, MESH:D015470	Arsenic Tr	15.48	22
PPP1R3A	5506	Leukemia, MESH:D015473	arsenite|D	15.48	157
RASSF5	83593	Leukemia, MESH:D015470	Doxorubic	15.48	14

RXRG	6258	Leukemia, MESH:D015470	Alitretinoi	15.48	17
SELENOM	140606	Leukemia, MESH:D015470	Cytarabine	15.48	79
SELP	6403	Leukemia, MESH:D015470	Arsenic Tr	15.48	17
SREBF1	6720	Leukemia, MESH:D007948	Arsenic Tr	15.48	3
TPK1	27010	Leukemia, MESH:D004915	Cytarabine	15.48	2
TRIP6	7205	Leukemia, MESH:D004915	Doxorubic	15.48	2
TUG1	55000	Leukemia, MESH:D004915	Doxorubic	15.48	2
YY1	7528	Leukemia, MESH:D015473	alpha-Toc	15.48	261
CHRNA6	8973	Leukemia, MESH:D015470	Arsenic Tr	15.47	14
CHSY1	22856	Leukemia, MESH:D015470	Dexameth	15.47	13
CLSTN3	9746	Leukemia, MESH:D015470	Dexameth	15.47	9
COL1A2	1278	Leukemia, MESH:D004915	Cytarabine	15.47	3
COL4A3	1285	Leukemia, MESH:D015470	Doxorubic	15.47	14
EMILIN2	84034	Leukemia, MESH:D015470	Dexameth	15.47	13
FHOD3	80206	Leukemia, MESH:D015470	Calcitriol|D	15.47	13
GNAL	2774	Leukemia, MESH:D015470	Arsenic Tr	15.47	13
KEAP1A	321837	Leukemia, MESH:D015473	Arsenic|Ar	15.47	158
MIRLET7C	406885	Leukemia, MESH:D015473	arsenite|Re	15.47	156
PJA1	64219	Leukemia, MESH:D015470	Azacitidine	15.47	20
PTPRM	5797	Leukemia, MESH:D015470	Calcitriol|D	15.47	13
PTPRS	5802	Leukemia, MESH:D015470	Doxorubic	15.47	13
TIMM17A	10440	Leukemia, MESH:D015470	Dexameth	15.47	10
TLCD4B	449652	Leukemia, MESH:D015473	Dexameth	15.47	152
TSPAN5	10098	Leukemia, MESH:D015470	Calcitriol|D	15.47	13
UBE3D	90025	Leukemia, MESH:D015473	Calcitriol|D	15.47	153
ANPEPB.1	322533	Leukemia, MESH:D015470	Alitretinoi	15.46	7
FAAP20	199990	Leukemia, MESH:D015470	Arsenic Tr	15.46	13
FLVCR1	28982	Leukemia, MESH:D015470	Air Polluta	15.46	11
GSTM1	2944	Leukemia, MESH:D007948	Arsenic Tr	15.46	4
MYL4	4635	Leukemia, MESH:D015473	Arsenic|Ar	15.46	167
MZT1	440145	Leukemia, MESH:D015470	Calcitriol|G	15.46	9
PAN3	255967	Leukemia, MESH:D015470	Dronabino	15.46	7
PARD3	56288	Leukemia, MESH:D015473	Arsenic|ars	15.46	155
PDLIM3	27295	Leukemia, MESH:D015473	Arsenic|Cy	15.46	19
PRAME	23532	Leukemia, MESH:D015470	Alitretinoi	15.46	12
RPUSD2	27079	Leukemia, MESH:D015470	Doxorubic	15.46	12
ABL2	27	Leukemia, MESH:D015473	Arsenic|ars	15.45	11
AHI1	54806	Leukemia, MESH:D004915	Cytarabine	15.45	2
BAIAP3	8938	Leukemia, MESH:D015470	(+)-JQ1 cc	15.45	6
CAND2	23066	Leukemia, MESH:D015470	Decitabine	15.45	18
CHAMP1	283489	Leukemia, MESH:D015470	Cyclophos	15.45	11
DUS1L	64118	Leukemia, MESH:D015470	Gasoline|P	15.45	7
ENTPD6	955	Leukemia, MESH:D015473	Arsenic|De	15.45	155
FEM1A	55527	Leukemia, MESH:D015470	Dexameth	15.45	8
GNGT1	2792	Leukemia, MESH:D015470	Dronabino	15.45	7
HSD17B4	3295	Leukemia, MESH:D015473	Arsenic|Ar	15.45	160
L3MBTL2	83746	Leukemia, MESH:D015470	Dexameth	15.45	6
MPP3	4356	Leukemia, MESH:D015470	Paclitaxel|I	15.45	7
PEAK1	79834	Leukemia, MESH:D015470	Benzene|D	15.45	25
PIR	8544	Leukemia, MESH:D015473	Antimony	15.45	262
TDP2	51567	Leukemia, MESH:D015470	Benzene|D	15.45	26
WRNIP1	56897	Leukemia, MESH:D015470	Dexameth	15.45	10
ZC3H14	79882	Leukemia, MESH:D015470	Air Polluta	15.45	13
ADORA1	134	Leukemia, MESH:D015470	Calcitriol|D	15.44	15
ALDOAA	336425	Leukemia, MESH:D015470	Alitretinoi	15.44	7
APBA3	9546	Leukemia, MESH:D015470	Decitabine	15.44	19
APRT	353	Leukemia, MESH:D015473	arsenite|D	15.44	8
ATP5PD	10476	Leukemia, MESH:D015470	Doxorubic	15.44	11

BZW1	9689	Leukemia, MESH:D015473	Arsenic Tr	15.44	261
CNN2	1265	Leukemia, MESH:D015473	Calcitriol|D	15.44	154
ELAC1	55520	Leukemia, MESH:D015470	Dexameth	15.44	6
FHIP2A	57700	Leukemia, MESH:D015470	Doxorubic	15.44	13
IFT46	56912	Leukemia, MESH:D015470	Dexameth	15.44	12
IGHG1	3500	Leukemia, MESH:D015473	Arsenic Tr	15.44	263
MSTO1	55154	Leukemia, MESH:D015470	Dexameth	15.44	8
MYT1L	23040	Leukemia, MESH:D015470	Arsenic Tr	15.44	13
NFATC3	4775	Leukemia, MESH:D015473	Arsenic|Ar	15.44	159
PSEN2	5664	Leukemia, MESH:D015473	Arsenic|De	15.44	11
RNFT1	51136	Leukemia, MESH:D015470	Calcitriol|D	15.44	11
SAV1	60485	Leukemia, MESH:D015470	Arsenic Tr	15.44	12
SF3B6	51639	Leukemia, MESH:D015470	Doxorubic	15.44	13
SHISA2	387914	Leukemia, MESH:D015473	Arsenic|Ar	15.44	265
TNRC6C	57690	Leukemia, MESH:D015470	Doxorubic	15.44	12
ZDHHC3	51304	Leukemia, MESH:D015470	Dexameth	15.44	12
ZNF521	25925	Leukemia, MESH:D015470	Dexameth	15.44	12
ASAP1	50807	Leukemia, MESH:D015473	Arsenic|ars	15.43	159
BCL2L11	10018	Leukemia, MESH:D015473	Arsenic Tr	15.43	262
KRT32	3882	Leukemia, MESH:D015470	Azacitidine	15.43	8
LYRM7	90624	Leukemia, MESH:D015470	Dexameth	15.43	7
MBNL1	4154	Leukemia, MESH:D015473	arsenite|D	15.43	155
PHF3	23469	Leukemia, MESH:D004915	Doxorubic	15.43	2
PSMD14	10213	Leukemia, MESH:D015473	Arsenic|De	15.43	158
RASGRP3	25780	Leukemia, MESH:D015473	Arsenic|De	15.43	155
SNAPC4	6621	Leukemia, MESH:D015470	Dexameth	15.43	11
TRIB3	57761	Leukemia, MESH:D004915	Doxorubic	15.43	3
YIPF6	286451	Leukemia, MESH:D015470	Dexameth	15.43	11
AIF1	199	Leukemia, MESH:D015473	Arsenic Tr	15.42	266
IRX4	50805	Leukemia, MESH:D004915	Daunorub	15.42	2
JAG1	182	Leukemia, MESH:D015473	arsenite|D	15.42	153
MYOM2	9172	Leukemia, MESH:D004915	Daunorub	15.42	2
PRKACB	5567	Leukemia, MESH:D015470	Dexameth	15.42	15
CCR3	1232	Leukemia, MESH:D015470	Alitretinoi	15.41	15
CD83	9308	Leukemia, MESH:D015473	Arsenic|De	15.41	156
CEBPA	1050	Leukemia, MESH:D004915	Doxorubic	15.41	3
COL15A1	1306	Leukemia, MESH:D015473	Dexameth	15.41	7
CTRB1	1504	Leukemia, MESH:D015470	Chloroqui	15.41	7
GCK	2645	Leukemia, MESH:D015473	Arsenic|Ar	15.41	264
HTRA3	94031	Leukemia, MESH:D015470	Arsenic Tr	15.41	22
ITM2C	81618	Leukemia, MESH:D004915	Cytarabine	15.41	3
KIAA1671	85379	Leukemia, MESH:D015473	arsenite|Ca	15.41	15
LPAR3	23566	Leukemia, MESH:D015470	Ethylnitros	15.41	9
PRDX6	9588	Leukemia, MESH:D015473	Arsenic|Ca	15.41	159
RPL37	6167	Leukemia, MESH:D015470	Arsenic Tr	15.41	30
SCN1A	6323	Leukemia, MESH:D015470	Doxorubic	15.41	13
SFTPC	6440	Leukemia, MESH:D015470	Dexameth	15.41	11
SLC31A2	1318	Leukemia, MESH:D015470	Calcitriol|C	15.41	11
CD99L2	83692	Leukemia, MESH:D015470	Dexameth	15.4	12
DPP9	91039	Leukemia, MESH:D015470	Dexameth	15.4	13
F3	2152	Leukemia, MESH:D004915	Daunorub	15.4	3
FAM149A	25854	Leukemia, MESH:D015470	Dexameth	15.4	8
GMPS	8833	Leukemia, MESH:D015470	Air Polluta	15.4	14
GPM6A	2823	Leukemia, MESH:D015473	Arsenic Tr	15.4	164
HAO1	54363	Leukemia, MESH:D015470	Dexameth	15.4	8
HLCS	3141	Leukemia, MESH:D015470	Decitabine	15.4	15
HS3ST3B1	9953	Leukemia, MESH:D015470	Doxorubic	15.4	13
IKZF1	10320	Leukemia, MESH:D015473	Calcitriol|D	15.4	157

INTS6	26512	Leukemia,	MESH:D015470	Arsenic Tr	15.4	16
IPO7	10527	Leukemia,	MESH:D015470	Calcitriol|D	15.4	7
ITGB7	3695	Leukemia,	MESH:D015470	Arsenic Tr	15.4	17
KPNA4	3840	Leukemia,	MESH:D015470	Calcitriol|D	15.4	13
MAST4	375449	Leukemia,	MESH:D015470	Calcitriol|D	15.4	8
MYCL	4610	Leukemia,	MESH:D015470	Calcitriol|N	15.4	9
OTX2	5015	Leukemia,	MESH:D015470	Dexameth	15.4	8
SATB1	6304	Leukemia,	MESH:D015473	Arsenic Tr	15.4	260
SEMA3F	6405	Leukemia,	MESH:D015470	Calcitriol|D	15.4	7
SGO1	151648	Leukemia,	MESH:D015473	Calcitriol|D	15.4	155
SIRPB1	10326	Leukemia,	MESH:D015473	arsenite|M	15.4	152
SLC5A6	8884	Leukemia,	MESH:D015470	Arsenic Tr	15.4	16
STEAP2	261729	Leukemia,	MESH:D015470	Air Polluta	15.4	12
TIMM9	26520	Leukemia,	MESH:D015470	Dexameth	15.4	13
ZNF875	284459	Leukemia,	MESH:D015470	Bortezomi	15.4	6
ACTN2	88	Leukemia,	MESH:D015473	arsenite|D	15.39	155
AGL	178	Leukemia,	MESH:D015473	Arsenic|Ar	15.39	264
ANXA11	311	Leukemia,	MESH:D015473	Arsenic Tr	15.39	259
CD48	962	Leukemia,	MESH:D015473	Genistein|I	15.39	153
CYP1A1	1543	Leukemia,	MESH:D007948	2-(2-amin	15.39	3
DAXX	1616	Leukemia,	MESH:D007948	2-(2-amin	15.39	3
FAM114A1	92689	Leukemia,	MESH:D015470	Dexameth	15.39	12
MIR99AHC	388815	Leukemia,	MESH:D015473	arsenite|Cy	15.39	156
OMD	4958	Leukemia,	MESH:D015470	Dexameth	15.39	13
RBM4	5936	Leukemia,	MESH:D004915	Doxorubic	15.39	2
SERP1	27230	Leukemia,	MESH:D015470	Dexameth	15.39	13
SFTPB	6439	Leukemia,	MESH:D015473	Calcitriol|D	15.39	154
SLC43A2	124935	Leukemia,	MESH:D015470	Dexameth	15.39	13
TENM4	26011	Leukemia,	MESH:D015470	Dexameth	15.39	12
VAMP1	6843	Leukemia,	MESH:D015470	Arsenic Tr	15.39	13
CP	1356	Leukemia,	MESH:D015473	Arsenic Tr	15.38	262
DDT	1652	Leukemia,	MESH:D015473	Arsenic Tr	15.38	262
DUSP3	1845	Leukemia,	MESH:D015473	Arsenic|M	15.38	157
HIBADH	11112	Leukemia,	MESH:D015473	Arsenic Tr	15.38	261
IL24	11009	Leukemia,	MESH:D015473	Arsenic|Ar	15.38	265
PSMA3	5684	Leukemia,	MESH:D015473	Arsenic|ars	15.38	11
SQOR	58472	Leukemia,	MESH:D015473	Arsenic|ars	15.38	13
SWSAP1	126074	Leukemia,	MESH:D015470	Thalidomi	15.38	6
THRA	7067	Leukemia,	MESH:D015473	Dexameth	15.38	157
YPEL2	388403	Leukemia,	MESH:D015473	Arsenic|ars	15.38	158
ZDHHC11	79844	Leukemia,	MESH:D015473	Arsenic|Et	15.38	154
CASP3A	140621	Leukemia,	MESH:D015473	Arsenic|Ar	15.37	160
CGREF1	10669	Leukemia,	MESH:D015473	Etoposide	15.37	151
LDB3	11155	Leukemia,	MESH:D004915	Daunorub	15.37	2
NEUROD4	58158	Leukemia,	MESH:D015470	Hydroxyur	15.37	6
NRAP	4892	Leukemia,	MESH:D004915	Daunorub	15.37	2
PIK3CD	5293	Leukemia,	MESH:D015470	Benzene|D	15.37	29
PPARGC1B	133522	Leukemia,	MESH:D015470	Etoposide	15.37	17
RBL2	5934	Leukemia,	MESH:D015470	Calcitriol|D	15.37	16
TGM1	7051	Leukemia,	MESH:D015470	Alitretinoi	15.37	11
TUBB2B	347733	Leukemia,	MESH:D015470	Cyclophos	15.37	16
WASHC1	1E+08	Leukemia,	MESH:D015470	Doxorubic	15.37	9
YAP1	10413	Leukemia,	MESH:D015470	Dexameth	15.37	14
ATP1A1A	64615	Leukemia,	MESH:D015470	Dexameth	15.36	5
C11ORF87	399947	Leukemia,	MESH:D015470	Arsenic Tr	15.36	12
C8ORF33	65265	Leukemia,	MESH:D015470	Doxorubic	15.36	13
EI24	9538	Leukemia,	MESH:D015473	Daunorub	15.36	20
FADD	8772	Leukemia,	MESH:D015473	Cholestero	15.36	154

KLK7	5650	Leukemia, MESH:D015473		Arsenic|Ca	15.36	155
MIR181C	406957	Leukemia, MESH:D015473		Arsenic Tr	15.36	261
TFF3	7033	Leukemia, MESH:D007948		2-(2-amin	15.36	3
UBE3B	89910	Leukemia, MESH:D015473		arsenite|D	15.36	6
ZNF554	115196	Leukemia, MESH:D015470		Arsenic Tr	15.36	12
ZNF706	51123	Leukemia, MESH:D015473		Arsenic|De	15.36	154
ZNF767P	79970	Leukemia, MESH:D015470		Dexameth	15.36	9
CLU	1191	Leukemia, MESH:D004915		Doxorubic	15.35	3
FBXO42	54455	Leukemia, MESH:D015473		arsenite|D	15.35	6
GPR83	10888	Leukemia, MESH:D015470		Cytarabine	15.35	75
H2AZ1	3015	Leukemia, MESH:D015473		arsenite|D	15.35	8
INA	9118	Leukemia, MESH:D015473		arsenite|C	15.35	156
PSTK	118672	Leukemia, MESH:D015473		Arsenic Tr	15.35	262
RUBCNL	80183	Leukemia, MESH:D015473		Calcitriol|D	15.35	153
TIPARP	25976	Leukemia, MESH:D015473		Arsenic Tr	15.35	262
ZNF626	199777	Leukemia, MESH:D015470		Doxorubic	15.35	11
ZNF721	170960	Leukemia, MESH:D015473		Arsenic|ar	15.35	10
IDH1	3417	Leukemia, MESH:D01	marker/m	Arsenic|Ar	15.34	266
C16ORF91	283951	Leukemia, MESH:D015470		Doxorubic	15.34	10
CYP51	13121	Leukemia, MESH:D015473		Arsenic|De	15.34	158
DENND1B	163486	Leukemia, MESH:D004915		Doxorubic	15.34	2
FAM120A	158293	Leukemia, MESH:D015470		Doxorubic	15.34	10
GAPDH	2597	Leukemia, MESH:D004915		Cytarabine	15.34	3
GJA5	2702	Leukemia, MESH:D015470		Arsenic Tr	15.34	27
GUSBP11	91316	Leukemia, MESH:D015470		Dexameth	15.34	5
HOXB4	3214	Leukemia, MESH:D015470		Cytarabine	15.34	81
IFNAR2	3455	Leukemia, MESH:D015470		Dexameth	15.34	10
ISLR	3671	Leukemia, MESH:D004915		Doxorubic	15.34	2
LRP4	4038	Leukemia, MESH:D015473		Arsenic|Ar	15.34	262
MAB21L2	10586	Leukemia, MESH:D004915		Cytarabine	15.34	2
MAP4K5	11183	Leukemia, MESH:D004915		Doxorubic	15.34	2
MZB1	51237	Leukemia, MESH:D015470		Air Polluta	15.34	9
RHOBTB1	9886	Leukemia, MESH:D015473		Arsenic|Ar	15.34	262
SERTAD4-	574036	Leukemia, MESH:D015470		(+)-JQ1 co	15.34	6
SYT15	83849	Leukemia, MESH:D015470		Dasatinib|	15.34	7
TANK	10010	Leukemia, MESH:D015473		Arsenic|Ar	15.34	264
ZC2HC1A	51101	Leukemia, MESH:D004915		Doxorubic	15.34	2
ZNF713	349075	Leukemia, MESH:D015470		Dexameth	15.34	5
ABI1	10006	Leukemia, MESH:D015470		Bortezomi	15.33	14
ACAN	176	Leukemia, MESH:D015470		Dasatinib|	15.33	10
AFF2	2334	Leukemia, MESH:D015470		Dronabinc	15.33	6
AK2	204	Leukemia, MESH:D015473		arsenite|D	15.33	8
ARHGAP2	23092	Leukemia, MESH:D015473		Arsenic|Ar	15.33	265
ARHGAP4	23526	Leukemia, MESH:D015470		Decitabine	15.33	15
ARID5A	10865	Leukemia, MESH:D015470		Dexameth	15.33	8
C11ORF54	28970	Leukemia, MESH:D015470		Arsenic Tr	15.33	13
CAMK2G	818	Leukemia, MESH:D015473		Arsenic Tr	15.33	262
CASK	8573	Leukemia, MESH:D015473		Arsenic Tr	15.33	263
CLIP4	79745	Leukemia, MESH:D015470		Bortezomi	15.33	9
CYTOR	112597	Leukemia, MESH:D015470		Decitabine	15.33	17
DLGAP4	22839	Leukemia, MESH:D015470		Arsenic Tr	15.33	19
DNAJC9	23234	Leukemia, MESH:D015470		Air Polluta	15.33	20
FOXN3	1112	Leukemia, MESH:D015473		Arsenic|M	15.33	157
MEP1A	4224	Leukemia, MESH:D015470		Arsenic Tr	15.33	15
MVB12A	93343	Leukemia, MESH:D015470		Cytarabine	15.33	78
MYOG	4656	Leukemia, MESH:D015470		Azacitidine	15.33	11
NCF4	4689	Leukemia, MESH:D015470		Bortezomi	15.33	11
PDK3	5165	Leukemia, MESH:D015470		Calcitriol|D	15.33	16

PHF23	79142	Leukemia, MESH:D015470		Air Polluta	15.33	8
PHLDB2	90102	Leukemia, MESH:D015473		Arsenic Tr	15.33	155
RER1	11079	Leukemia, MESH:D015470		Dexameth	15.33	11
RNASET2	8635	Leukemia, MESH:D015470		Dronabinc	15.33	10
SAMD9L	219285	Leukemia, MESH:D015470		Air Polluta	15.33	14
SCGN	10590	Leukemia, MESH:D015473		Arsenic|De	15.33	155
SERP2	387923	Leukemia, MESH:D015470		Air Polluta	15.33	8
SMIM10L2	644596	Leukemia, MESH:D015470		Doxorubic	15.33	11
SYT11	23208	Leukemia, MESH:D015470		Benzoates	15.33	8
TCERG1L	256536	Leukemia, MESH:D015470		Decitabine	15.33	13
TLR6	10333	Leukemia, MESH:D007948		2-(2-amin	15.33	1
TLR7	51284	Leukemia, MESH:D007948		2-(2-amin	15.33	1
TTC19	54902	Leukemia, MESH:D015470		Benzene|D	15.33	27
UFSP2	55325	Leukemia, MESH:D015470		Air Polluta	15.33	7
WASL	8976	Leukemia, MESH:D015470		Doxorubic	15.33	13
ZMIZ1	57178	Leukemia, MESH:D015473		Arsenic|ars	15.33	156
NUP98	4928	Leukemia, MESH:D01	marker/me	Dexameth	15.32	14
ANAPC10	10393	Leukemia, MESH:D015470		Dexameth	15.32	7
AP1AR	55435	Leukemia, MESH:D015470		Dronabinc	15.32	7
APOLD1	81575	Leukemia, MESH:D015470		Dronabinc	15.32	11
ARPC2	10109	Leukemia, MESH:D015470		Dexameth	15.32	13
AVPR1A	552	Leukemia, MESH:D015470		Doxorubic	15.32	24
BBS1	582	Leukemia, MESH:D015470		Dexameth	15.32	11
BZW2	28969	Leukemia, MESH:D015470		Arsenic Tr	15.32	18
CBLN2	147381	Leukemia, MESH:D015470		Calcitriol|D	15.32	7
DMAP1	55929	Leukemia, MESH:D015470		Dexameth	15.32	11
DUSP18	150290	Leukemia, MESH:D015470		Benzene|D	15.32	25
EMC3	55831	Leukemia, MESH:D015470		Indometha	15.32	7
ERO1B	56605	Leukemia, MESH:D015470		Doxorubic	15.32	13
F7	2155	Leukemia, MESH:D015470		Bezafibrat	15.32	13
FBP2	8789	Leukemia, MESH:D015473		Arsenic|ars	15.32	157
GLG1	2734	Leukemia, MESH:D015470		Doxorubic	15.32	13
GRK6	2870	Leukemia, MESH:D015470		Dexameth	15.32	13
HELB	92797	Leukemia, MESH:D015470		Decitabine	15.32	14
HLA-E	3133	Leukemia, MESH:D004915		Doxorubic	15.32	3
HOXC10	3226	Leukemia, MESH:D015470		Dexameth	15.32	9
HOXD4	3233	Leukemia, MESH:D015470		Arsenic Tr	15.32	12
LAMP1	3916	Leukemia, MESH:D015470		Arsenic Tr	15.32	20
LPIN1	23175	Leukemia, MESH:D015473		Dexameth	15.32	159
MB21D2	151963	Leukemia, MESH:D015470		Dexameth	15.32	7
MYH7B	57644	Leukemia, MESH:D015470		Doxorubic	15.32	11
NAA16	79612	Leukemia, MESH:D015470		Doxorubic	15.32	12
PAFAH1B3	5050	Leukemia, MESH:D015470		Arsenic Tr	15.32	18
PNMT	5409	Leukemia, MESH:D015470		Bortezomi	15.32	8
PXMP4	11264	Leukemia, MESH:D015470		Dexameth	15.32	11
RNF126	55658	Leukemia, MESH:D015470		Gasoline|(	15.32	7
RPL30	6156	Leukemia, MESH:D015470		Arsenic Tr	15.32	13
SDK1	221935	Leukemia, MESH:D015470		Calcitriol|D	15.32	7
SFSWAP	6433	Leukemia, MESH:D015470		Dexameth	15.32	13
SLC6A1	6529	Leukemia, MESH:D015470		Dexameth	15.32	13
SPEF2	79925	Leukemia, MESH:D015470		Indometha	15.32	6
THYN1	29087	Leukemia, MESH:D015470		Arsenic Tr	15.32	19
TRPS1	7227	Leukemia, MESH:D015470		Dexameth	15.32	13
TXNRD2	10587	Leukemia, MESH:D015470		Bortezomi	15.32	12
USF1	7391	Leukemia, MESH:D015470		Arsenic Tr	15.32	17
ATF7IP2	80063	Leukemia, MESH:D015470		Doxorubic	15.31	11
CEP120	153241	Leukemia, MESH:D015470		Doxorubic	15.31	13
COLEC11	78989	Leukemia, MESH:D015470		Dexameth	15.31	12

ENOPH1	58478	Leukemia, MESH:D015470	Dexameth	15.31	7
FAF2	23197	Leukemia, MESH:D015470	Dexameth	15.31	13
HABP4	22927	Leukemia, MESH:D015470	Doxorubic	15.31	13
KCTD11	147040	Leukemia, MESH:D015470	(+)-JQ1 cc	15.31	7
KIAA1217	56243	Leukemia, MESH:D015470	Doxorubic	15.31	12
KLHDC9	126823	Leukemia, MESH:D015470	Arsenic Tr	15.31	13
KLHL15	80311	Leukemia, MESH:D015470	Doxorubic	15.31	11
METTL23	124512	Leukemia, MESH:D015470	Dexameth	15.31	6
MIR184	406960	Leukemia, MESH:D015473	Arsenic|Ar	15.31	158
MIR18A	406953	Leukemia, MESH:D015470	Arsenic Tr	15.31	12
PLAT	5327	Leukemia, MESH:D004915	Cytarabine	15.31	3
RNF168	165918	Leukemia, MESH:D015470	Dexameth	15.31	11
TEX9	374618	Leukemia, MESH:D015470	Doxorubic	15.31	14
TM9SF4	9777	Leukemia, MESH:D015470	Dexameth	15.31	12
TRIM62	55223	Leukemia, MESH:D015470	Doxorubic	15.31	11
ZNF750	79755	Leukemia, MESH:D015470	Dexameth	15.31	6
ANKS1A	23294	Leukemia, MESH:D015473	Arsenic Tr	15.3	162
MIR4423	1.01E+08	Leukemia, MESH:D015470	Doxorubic	15.3	16
ASXL1	171023	Leukemia, MESH:D004915	Doxorubic	15.29	2
CAB39	51719	Leukemia, MESH:D004915	Daunorub	15.29	2
CCT8	10694	Leukemia, MESH:D015473	Arsenic Tr	15.29	157
ETFDH	2110	Leukemia, MESH:D015473	Arsenic|De	15.29	157
FUT8	2530	Leukemia, MESH:D015473	Arsenic|Ca	15.29	154
PRKD1	5587	Leukemia, MESH:D015473	Arsenic|ars	15.29	158
ZNF467	168544	Leukemia, MESH:D015473	Arsenic|Cy	15.29	158
DYSF	8291	Leukemia, MESH:D015473	arsenite|Ca	15.28	155
MYL1	4632	Leukemia, MESH:D015473	Arsenic|De	15.28	156
TAF1D	79101	Leukemia, MESH:D015473	Arsenic|Ar	15.28	264
TRIM16	10626	Leukemia, MESH:D015473	Arsenic Tr	15.28	261
UPB1	51733	Leukemia, MESH:D015473	Dexameth	15.28	153
WNT16	51384	Leukemia, MESH:D015473	Arsenic|Ar	15.28	159
ACSS1	84532	Leukemia, MESH:D015473	Arsenic|ars	15.27	11
CORO1C	23603	Leukemia, MESH:D015473	Arsenic|De	15.27	11
DAXX	1616	Leukemia, MESH:D015473	Arsenic|Ar	15.27	264
GTPBP2	54676	Leukemia, MESH:D015473	Dexameth	15.27	153
GYS1	2997	Leukemia, MESH:D015473	Arsenic Tr	15.27	261
HAT1	8520	Leukemia, MESH:D007948	2-(2-amin	15.27	2
LAPTM5	7805	Leukemia, MESH:D015473	Arsenic|Ar	15.27	264
SCN2A	6326	Leukemia, MESH:D015473	Arsenic Tr	15.27	260
SMAD6	4091	Leukemia, MESH:D007948	Arsenic Tr	15.27	3
TUBB5	22154	Leukemia, MESH:D015470	Dexameth	15.27	17
ATF1	466	Leukemia, MESH:D015470	Deferoxan	15.26	10
BAG1	573	Leukemia, MESH:D015473	alvocidib|A	15.26	157
BOP1	23246	Leukemia, MESH:D015470	Arsenic Tr	15.26	18
CUX2	23316	Leukemia, MESH:D015470	Doxorubic	15.26	12
CYCS	54205	Leukemia, MESH:D007948	Arsenic Tr	15.26	3
CYTIP	9595	Leukemia, MESH:D015470	Air Polluta	15.26	10
FASLG	356	Leukemia, MESH:D004915	Daunorub	15.26	2
FGD5-AS1	1.01E+08	Leukemia, MESH:D015470	Thalidomi	15.26	4
JPT1	51155	Leukemia, MESH:D015470	Arsenic Tr	15.26	22
KHSRP	8570	Leukemia, MESH:D015470	Doxorubic	15.26	14
MPEG1	219972	Leukemia, MESH:D015470	Bezafibrat	15.26	10
OLFML2B	25903	Leukemia, MESH:D015470	Calcitriol|D	15.26	10
PCCB	5096	Leukemia, MESH:D015470	Doxorubic	15.26	14
SALL1	6299	Leukemia, MESH:D015470	Cyclophos	15.26	13
TRPV1	7442	Leukemia, MESH:D015470	Cyclophos	15.26	84
VIL1	7429	Leukemia, MESH:D015470	Arsenic Tr	15.26	17
ADRA2C	152	Leukemia, MESH:D015470	Dexameth	15.25	9

APCDD1L	164284	Leukemia, MESH:D015473	Arsenic|Ca	15.25	9
CFAP44	55779	Leukemia, MESH:D015473	Genistein|I	15.25	152
CHD3	1107	Leukemia, MESH:D015470	Decitabine	15.25	16
DLST	1743	Leukemia, MESH:D015470	Doxorubic	15.25	14
GTF2IRD1	9569	Leukemia, MESH:D015470	Doxorubic	15.25	14
HDLBP	3069	Leukemia, MESH:D015470	Dexameth	15.25	14
HYAL1	3373	Leukemia, MESH:D015470	Arsenic Tr	15.25	25
ITIH5	80760	Leukemia, MESH:D015470	Doxorubic	15.25	14
PLCD1	5333	Leukemia, MESH:D015470	Arsenic Tr	15.25	20
PRF1	5551	Leukemia, MESH:D015473	Arsenic|Ar	15.25	160
PURA	5813	Leukemia, MESH:D015470	Cyclophos	15.25	13
RPL13P5	283345	Leukemia, MESH:D015470	Bortezomi	15.25	5
SF3B1	23451	Leukemia, MESH:D015470	Methotrex	15.25	12
SLC1A6	6511	Leukemia, MESH:D015470	Calcitriol|I	15.25	8
SLC25A22	79751	Leukemia, MESH:D015470	Dexameth	15.25	13
SOCS2	8835	Leukemia, MESH:D015473	Calcitriol|D	15.25	156
TMEM45B	120224	Leukemia, MESH:D015470	Dexameth	15.25	9
TOX3	27324	Leukemia, MESH:D015470	Calcitriol|D	15.25	14
TSPAN7	7102	Leukemia, MESH:D015470	Decitabine	15.25	15
CDC42EP2	10435	Leukemia, MESH:D015470	Dexameth	15.24	12
DIP2A	23181	Leukemia, MESH:D015470	Ethylnitros	15.24	10
HSDL1	83693	Leukemia, MESH:D015473	Arsenic|Ar	15.24	263
INO80	54617	Leukemia, MESH:D015473	Arsenic Tr	15.24	155
NFE2L3	9603	Leukemia, MESH:D015473	Arsenic|Ar	15.24	263
NR3C1	2908	Leukemia, MESH:D007948	2-(2-amin	15.24	3
OSM	5008	Leukemia, MESH:D015473	Antimony	15.24	156
PKD2L1	9033	Leukemia, MESH:D015473	Arsenic Tr	15.24	153
RBM45	129831	Leukemia, MESH:D015473	Arsenic|ars	15.24	155
SUMO1	7341	Leukemia, MESH:D007948	2-(2-amin	15.24	3
DNAJB4	11080	Leukemia, MESH:D015473	Antimony	15.23	156
GM2A	2760	Leukemia, MESH:D015473	Arsenic|De	15.23	155
IGFBP1B	793907	Leukemia, MESH:D015470	Dronabinc	15.23	8
PLXNB2	23654	Leukemia, MESH:D015473	Arsenic|Ar	15.23	159
PPBP	5473	Leukemia, MESH:D015473	Arsenic|Ar	15.23	159
RPRD1B	58490	Leukemia, MESH:D015473	Dexameth	15.23	155
STS	412	Leukemia, MESH:D015473	arsenite|G	15.23	153
TET2	54790	Leukemia, MESH:D01 marker/m	Arsenic|Ar	15.22	266
BHLHE22	27319	Leukemia, MESH:D015470	Cytarabine	15.22	74
CGB3	1082	Leukemia, MESH:D007948	2-(2-amin	15.22	2
CMAH	12763	Leukemia, MESH:D015470	Dexameth	15.22	10
F8	2157	Leukemia, MESH:D015473	Arsenic Tr	15.22	261
HRAS	3265	Leukemia, MESH:D015473	Arsenic|Ar	15.22	265
INIP	58493	Leukemia, MESH:D015470	Air Polluta	15.22	10
LIPE	3991	Leukemia, MESH:D015470	Deferoxan	15.22	15
NOP10	55505	Leukemia, MESH:D015473	arsenite|D	15.22	154
PADI2	11240	Leukemia, MESH:D015473	arsenite|D	15.22	155
PAPPA	5069	Leukemia, MESH:D007948	Arsenic Tr	15.22	3
PHEX	5251	Leukemia, MESH:D015473	Arsenic|Ca	15.22	156
ANKRD27	84079	Leukemia, MESH:D015470	Etoposide	15.21	15
C1ORF112	55732	Leukemia, MESH:D015473	Calcitriol|D	15.21	153
GDF2	2658	Leukemia, MESH:D015470	Alitretinoi	15.21	12
GPS2	2874	Leukemia, MESH:D015470	Dexameth	15.21	6
H2AC19	723790	Leukemia, MESH:D015470	Benzene|D	15.21	25
HARS2	23438	Leukemia, MESH:D015470	Arsenic Tr	15.21	27
IRGM1	15944	Leukemia, MESH:D015470	Dexameth	15.21	8
KCNJ2-AS	400617	Leukemia, MESH:D015470	Doxorubic	15.21	8
LATS1	9113	Leukemia, MESH:D015470	Decitabine	15.21	18
LRRC39	127495	Leukemia, MESH:D015470	Dexameth	15.21	12

PKNOX1	5316	Leukemia, MESH:D015470		Arsenic Tr	15.21	14
POLR2A	5430	Leukemia, MESH:D015473		alvocidib|/	15.21	157
PRL	5617	Leukemia, MESH:D004915		Cytarabine	15.21	4
SLC6A10P	653562	Leukemia, MESH:D015470		(+)-JQ1 co	15.21	4
SNRNP48	154007	Leukemia, MESH:D015470		Bortezomi	15.21	10
SPNS1	83985	Leukemia, MESH:D015470		Alitretinoi	15.21	7
SZT2	23334	Leukemia, MESH:D015470		Dexameth	15.21	11
TIMMDC1	51300	Leukemia, MESH:D015470		Air Polluta	15.21	11
ADAMTS2	80070	Leukemia, MESH:D015470		Calcitriol|D	15.2	6
ALKBH5	54890	Leukemia, MESH:D015470		Dronabinc	15.2	6
APOBR	55911	Leukemia, MESH:D015470		Bortezomi	15.2	8
ATP5G1	11951	Leukemia, MESH:D015470		Arsenic Tr	15.2	19
BPNT2	54928	Leukemia, MESH:D004915		Cytarabine	15.2	2
DNAJC22	79962	Leukemia, MESH:D015470		Dronabinc	15.2	7
FAM181B	220382	Leukemia, MESH:D015470		Decitabine	15.2	16
FBRSL1	57666	Leukemia, MESH:D015470		Indometha	15.2	8
FEZF2	55079	Leukemia, MESH:D015470		Dexameth	15.2	7
GEMIN8	54960	Leukemia, MESH:D015470		Decitabine	15.2	18
GLUD2	2747	Leukemia, MESH:D015470		Calcitriol|D	15.2	7
IL36RN	26525	Leukemia, MESH:D015470		Benzene|D	15.2	25
LARP4B	23185	Leukemia, MESH:D004915		Doxorubic	15.2	2
MGRN1	23295	Leukemia, MESH:D015470		Dexameth	15.2	12
MIR134	406924	Leukemia, MESH:D015470		Calcitriol|D	15.2	8
MIR212	406994	Leukemia, MESH:D015470		Arsenic Tr	15.2	32
MPP4	58538	Leukemia, MESH:D015470		Arsenic Tr	15.2	13
N4BP1	9683	Leukemia, MESH:D004915		Doxorubic	15.2	2
NCK2	8440	Leukemia, MESH:D004915		Daunorub	15.2	2
NXPH4	11247	Leukemia, MESH:D015470		Decitabine	15.2	12
PLA2G10	8399	Leukemia, MESH:D015473		Calcitriol|D	15.2	151
RADX	55086	Leukemia, MESH:D015470		Calcitriol|N	15.2	8
SETDB1	9869	Leukemia, MESH:D015470		Arsenic Tr	15.2	18
SLC39A3	29985	Leukemia, MESH:D015470		Calcitriol|D	15.2	7
SLC41A1	254428	Leukemia, MESH:D015470		Calcitriol|D	15.2	12
SLC4A11	83959	Leukemia, MESH:D004915		Doxorubic	15.2	2
TRIM69	140691	Leukemia, MESH:D015470		Doxorubic	15.2	12
ZKSCAN3	80317	Leukemia, MESH:D015470		Methotrex	15.2	10
ADO	84890	Leukemia, MESH:D015470		Dexameth	15.19	11
ARL6	84100	Leukemia, MESH:D015470		Dexameth	15.19	8
CAMSAP3	57662	Leukemia, MESH:D015470		Calcitriol|D	15.19	6
CAPS2	84698	Leukemia, MESH:D015470		Dexameth	15.19	6
CES3	23491	Leukemia, MESH:D015473		Dexameth	15.19	7
GTPBP8	29083	Leukemia, MESH:D015470		Dexameth	15.19	7
IFI35	3430	Leukemia, MESH:D015473		Calcitriol|D	15.19	155
KLHL18	23276	Leukemia, MESH:D015470		Dexameth	15.19	11
KLHL22	84861	Leukemia, MESH:D015470		Dexameth	15.19	11
LGR4	55366	Leukemia, MESH:D015473		Arsenic Tr	15.19	263
MTX3	345778	Leukemia, MESH:D015470		(+)-JQ1 co	15.19	8
NAA40	79829	Leukemia, MESH:D015470		Dexameth	15.19	11
PEF1	553115	Leukemia, MESH:D015470		Decitabine	15.19	17
POLA2	23649	Leukemia, MESH:D015473		Calcitriol|D	15.19	155
RFXAP	5994	Leukemia, MESH:D015470		(+)-JQ1 co	15.19	7
STAU1	6780	Leukemia, MESH:D015470		Dexameth	15.19	11
TARBP2	6895	Leukemia, MESH:D015470		Dexameth	15.19	11
TMEM41A	90407	Leukemia, MESH:D015470		Dexameth	15.19	13
ZDHHC17	23390	Leukemia, MESH:D015470		Dexameth	15.19	6
ZGRF1	55345	Leukemia, MESH:D015470		Calcitriol|D	15.19	12
ZSWIM4	65249	Leukemia, MESH:D015470		Doxorubic	15.19	13
FXYD6	53826	Leukemia, MESH:D01	marker/me	Decitabine	15.18	20

BTF3	689	Leukemia, MESH:D015470	Arsenic Tr	15.18	20
CELSR2	1952	Leukemia, MESH:D015470	Calcitriol|F	15.18	10
DLX5	1749	Leukemia, MESH:D015470	Azacitidine	15.18	15
ERMAP	114625	Leukemia, MESH:D015470	Doxorubic	15.18	12
INTS9	55756	Leukemia, MESH:D015470	Doxorubic	15.18	12
KCNK2	3776	Leukemia, MESH:D015470	Arsenic Tr	15.18	14
KLHL5	51088	Leukemia, MESH:D015470	Calcitriol|D	15.18	20
LTBP4	8425	Leukemia, MESH:D015470	Decitabine	15.18	19
LUC7L	55692	Leukemia, MESH:D015470	Bortezomi	15.18	8
PDE1A	5136	Leukemia, MESH:D015473	Arsenic Tr	15.18	164
PPAN	56342	Leukemia, MESH:D015470	Dronabinc	15.18	11
PTPRO	5800	Leukemia, MESH:D015473	Arsenic Tr	15.18	261
RBP7	116362	Leukemia, MESH:D015470	Calcitriol|D	15.18	14
RIPK4	54101	Leukemia, MESH:D015470	Calcitriol|D	15.18	9
SKIL	6498	Leukemia, MESH:D015473	Arsenic|Ge	15.18	156
SLC22A23	63027	Leukemia, MESH:D015473	Arsenic|Ca	15.18	155
STXBP6	29091	Leukemia, MESH:D015470	Arsenic Tr	15.18	25
TPCN1	53373	Leukemia, MESH:D015470	Dexameth	15.18	12
ABCA11P	79963	Leukemia, MESH:D015470	Dexameth	15.17	4
CMIP	80790	Leukemia, MESH:D004915	Daunorub	15.17	2
CSF2RB	1439	Leukemia, MESH:D015473	Arsenic Tr	15.17	260
GSS	2937	Leukemia, MESH:D015473	Arsenic|Bu	15.17	156
INF2	64423	Leukemia, MESH:D015470	Dexameth	15.17	8
OTUB2	78990	Leukemia, MESH:D015470	Dexameth	15.17	7
PDGFRL	5157	Leukemia, MESH:D015473	Calcitriol|D	15.17	154
PSMB2	5690	Leukemia, MESH:D015473	arsenite|D	15.17	154
PTPRU	10076	Leukemia, MESH:D015470	Dexameth	15.17	8
TCFL5	10732	Leukemia, MESH:D015473	Etoposide	15.17	151
TUBB4A	10382	Leukemia, MESH:D015470	Doxorubic	15.17	13
ZIC6	415097	Leukemia, MESH:D015470	Dexameth	15.17	4
HDAC3	8841	Leukemia, MESH:D015473	Dexameth	15.16	155
IGHA1	3493	Leukemia, MESH:D015470	Air Polluta	15.16	11
JUN	3725	Leukemia, MESH:D004915	Cytarabine	15.16	3
KCNK9	51305	Leukemia, MESH:D015473	Calcitriol|C	15.16	5
NCK2A	393972	Leukemia, MESH:D015470	Tretinoin|t	15.16	5
PHB1	5245	Leukemia, MESH:D015470	Arsenic Tr	15.16	19
PSMD7	5713	Leukemia, MESH:D015473	Arsenic Tr	15.16	262
SCAPER	49855	Leukemia, MESH:D004915	Doxorubic	15.16	2
SUN2	25777	Leukemia, MESH:D004915	Doxorubic	15.16	2
ZNF423	23090	Leukemia, MESH:D015473	Cytarabine	15.16	155
HMOX1	3162	Leukemia, MESH:D007948	2-(2-amin	15.15	3
CRP	1401	Leukemia, MESH:D015473	alpha-Toc	15.14	161
TENT5A	55603	Leukemia, MESH:D015473	arsenite|Cy	15.14	156
TTR	7276	Leukemia, MESH:D015470	Alitretinoi	15.14	17
VGLL4	9686	Leukemia, MESH:D004915	Cytarabine	15.14	3
ADAM19	8728	Leukemia, MESH:D015473	Calcitriol|C	15.13	156
CFAP36	112942	Leukemia, MESH:D015473	Arsenic Tr	15.13	261
GRIN2A	2903	Leukemia, MESH:D015470	Allopurinc	15.13	30
HN1	1E+08	Leukemia, MESH:D015470	Tobacco S	15.13	7
ILK	3611	Leukemia, MESH:D015473	Dexameth	15.13	157
POLR1H	30834	Leukemia, MESH:D004915	Doxorubic	15.13	3
RPS6KB1	6198	Leukemia, MESH:D004915	Daunorub	15.13	3
SERBP1	26135	Leukemia, MESH:D015473	Arsenic Tr	15.13	266
SESN3	143686	Leukemia, MESH:D015473	Cytarabine	15.13	155
SPICE1	152185	Leukemia, MESH:D015473	Arsenic|Ar	15.13	263
ZBTB10	65986	Leukemia, MESH:D015473	Arsenic|De	15.13	13
AVPI1	60370	Leukemia, MESH:D015470	Benzene|E	15.12	29
CHAT	1103	Leukemia, MESH:D015470	Azacitidine	15.12	84

CHD1	1105	Leukemia, MESH:D004915	Doxorubic	15.12	2
CUL4A	8451	Leukemia, MESH:D015470	Benzene|Ir	15.12	25
EHMT2	10919	Leukemia, MESH:D015473	Arsenic|Ar	15.12	265
KLF12	11278	Leukemia, MESH:D015473	Arsenic|ars	15.12	157
NOVA1	4857	Leukemia, MESH:D004915	Cytarabine	15.12	3
PLEKHG3	26030	Leukemia, MESH:D004915	Cytarabine	15.12	2
PPP1R13L	10848	Leukemia, MESH:D004915	Daunorub	15.12	2
PRKG1	5592	Leukemia, MESH:D015470	Dexameth	15.12	14
SEC24D	9871	Leukemia, MESH:D015473	arsenite|G	15.12	154
SUCLA2	8803	Leukemia, MESH:D015470	Doxorubic	15.12	22
SYT1	6857	Leukemia, MESH:D015473	arsenite|Ca	15.12	154
TRAM2	9697	Leukemia, MESH:D004915	Doxorubic	15.12	2
UBE2D3	7323	Leukemia, MESH:D015473	Arsenic|De	15.12	11
ZNF560	147741	Leukemia, MESH:D015473	Arsenic|ars	15.12	154
BDH2	56898	Leukemia, MESH:D015473	Arsenic|Ar	15.11	263
BLVRA	644	Leukemia, MESH:D015470	Calcitriol|D	15.11	9
CH25H	9023	Leukemia, MESH:D015473	arsenite|D	15.11	155
CXCL12A	352944	Leukemia, MESH:D015470	Dexameth	15.11	5
DDIAS	220042	Leukemia, MESH:D015470	Calcitriol|D	15.11	9
EPB41	2035	Leukemia, MESH:D015470	Decitabine	15.11	15
EPOR	2057	Leukemia, MESH:D007948	Arsenic Tr	15.11	3
FETUB	26998	Leukemia, MESH:D015473	Arsenic|De	15.11	156
GFAP	2670	Leukemia, MESH:D004915	Doxorubic	15.11	3
GPCPD1	56261	Leukemia, MESH:D015473	Arsenic|De	15.11	156
GULP1	51454	Leukemia, MESH:D015473	Arsenic Tr	15.11	262
IGKC	3514	Leukemia, MESH:D015470	Arsenic Tr	15.11	16
KLK2	3817	Leukemia, MESH:D015473	Calcitriol|E	15.11	6
KLRK1	22914	Leukemia, MESH:D015470	Arsenic Tr	15.11	16
MIR25	407014	Leukemia, MESH:D015470	Air Polluta	15.11	13
NDUFA5	4698	Leukemia, MESH:D015470	Benzene|D	15.11	27
NEUROD1	4760	Leukemia, MESH:D015470	Dexameth	15.11	9
PRKCSH	5589	Leukemia, MESH:D015470	Doxorubic	15.11	12
RAB14	51552	Leukemia, MESH:D015470	Benzene|E	15.11	24
RALA	5898	Leukemia, MESH:D015473	Arsenic Tr	15.11	262
RTP4	64108	Leukemia, MESH:D015470	Irinotecan	15.11	8
SERINC3	10955	Leukemia, MESH:D015470	Dexameth	15.11	14
UROD	7389	Leukemia, MESH:D015470	Arsenic Tr	15.11	17
ABCG5	64240	Leukemia, MESH:D015473	Arsenic|Ch	15.1	155
C11ORF1	64776	Leukemia, MESH:D015470	Doxorubic	15.1	13
CD151	977	Leukemia, MESH:D015470	Cyclophos	15.1	10
FADS3	3995	Leukemia, MESH:D015470	Dexameth	15.1	16
FCF1	51077	Leukemia, MESH:D015470	Doxorubic	15.1	11
GOSR2	9570	Leukemia, MESH:D015470	Bortezomi	15.1	13
INS1	16333	Leukemia, MESH:D007948	2-(2-amin	15.1	3
KANK2	25959	Leukemia, MESH:D015470	Doxorubic	15.1	12
LMNB2	84823	Leukemia, MESH:D015470	Dexameth	15.1	9
MIR17	406952	Leukemia, MESH:D015470	Arsenic Tr	15.1	14
MTX2	10651	Leukemia, MESH:D015470	Benzene|D	15.1	24
POTEE	445582	Leukemia, MESH:D015470	Calcitriol|D	15.1	11
RNPEP	6051	Leukemia, MESH:D015470	Arsenic Tr	15.1	13
ROBO1	6091	Leukemia, MESH:D007948	Arsenic Tr	15.1	2
RSPO2	340419	Leukemia, MESH:D015470	Arsenic Tr	15.1	80
SCNN1G	6340	Leukemia, MESH:D015470	Dexameth	15.1	13
SEMA4D	10507	Leukemia, MESH:D015470	Doxorubic	15.1	15
SH3BGRL3	83442	Leukemia, MESH:D015470	Arsenic Tr	15.1	15
TATDN2	9797	Leukemia, MESH:D015470	Dexameth	15.1	5
ADSS2	159	Leukemia, MESH:D015470	Arsenic Tr	15.09	13
CNIH2	254263	Leukemia, MESH:D015470	Calcitriol|C	15.09	74

CRIM1	51232	Leukemia, MESH:D015473	Arsenic|Ar	15.09	262
CRNN	49860	Leukemia, MESH:D015470	Methotrex	15.09	8
CYP2B9	13094	Leukemia, MESH:D015473	alpha-Toc	15.09	12
EXT2	2132	Leukemia, MESH:D015470	Cytarabine	15.09	78
GALNT4	8693	Leukemia, MESH:D015470	Bortezomi	15.09	11
HINFP	25988	Leukemia, MESH:D015470	Arsenic Tr	15.09	13
MCRS1	10445	Leukemia, MESH:D015470	Dexameth	15.09	6
MIR20B	574032	Leukemia, MESH:D015470	Arsenic Tr	15.09	15
PGAM4	441531	Leukemia, MESH:D015470	Doxorubic	15.09	11
POLR2D	5433	Leukemia, MESH:D015470	Mitoxantro	15.09	18
PRORSD1I	344405	Leukemia, MESH:D015470	(+)-JQ1 co	15.09	5
PSMC6	5706	Leukemia, MESH:D015473	alpha-Toc	15.09	12
STX5	6811	Leukemia, MESH:D015470	Doxorubic	15.09	11
TIGD1	200765	Leukemia, MESH:D015470	(+)-JQ1 co	15.09	5
TRAPPC2L	51693	Leukemia, MESH:D015470	Dexameth	15.09	11
VEZT	55591	Leukemia, MESH:D015470	Bortezomi	15.09	12
ZNF747	65988	Leukemia, MESH:D015470	Doxorubic	15.09	12
ZNF771	51333	Leukemia, MESH:D015470	Arsenic Tr	15.09	17
ACTR3C	653857	Leukemia, MESH:D015470	Arsenic Tr	15.08	11
ADAMTS8	11095	Leukemia, MESH:D015470	Decitabine	15.08	18
BTBD2	55643	Leukemia, MESH:D015470	Arsenic Tr	15.08	13
CDR2L	30850	Leukemia, MESH:D015470	Calcitriol|D	15.08	11
CMC2	56942	Leukemia, MESH:D015473	Daunorub	15.08	159
CUZD1	50624	Leukemia, MESH:D015470	Dexameth	15.08	9
DCTN6	10671	Leukemia, MESH:D015470	Doxorubic	15.08	12
FER1L4	80307	Leukemia, MESH:D015470	Dexameth	15.08	8
FOXC1	2296	Leukemia, MESH:D015473	Calcitriol|D	15.08	152
HOXA13	3209	Leukemia, MESH:D015473	Genistein|I	15.08	152
HS2ST1	9653	Leukemia, MESH:D015470	Doxorubic	15.08	15
ICAM4	3386	Leukemia, MESH:D015470	Doxorubic	15.08	15
IL23R	149233	Leukemia, MESH:D015473	Mercaptop	15.08	151
JMJD7-PL	8681	Leukemia, MESH:D015470	Dexameth	15.08	5
KDELR3	11015	Leukemia, MESH:D015473	Arsenic|Ca	15.08	158
KIAA0895	23366	Leukemia, MESH:D015470	Doxorubic	15.08	10
LGI3	203190	Leukemia, MESH:D015470	Arsenic Tr	15.08	15
LPXN	9404	Leukemia, MESH:D015473	arsenite|D	15.08	154
LRRC23	10233	Leukemia, MESH:D015470	Decitabine	15.08	18
LSM8	51691	Leukemia, MESH:D015470	Pentachlo	15.08	9
N4BP3	23138	Leukemia, MESH:D015470	Calcitriol|D	15.08	11
NAP1L3	4675	Leukemia, MESH:D015470	Dexameth	15.08	11
NCKIPSD	51517	Leukemia, MESH:D015470	Arsenic Tr	15.08	18
NEUROD2	4761	Leukemia, MESH:D015470	Dexameth	15.08	8
PDGFC	56034	Leukemia, MESH:D015473	Arsenic|ars	15.08	155
PRKX	5613	Leukemia, MESH:D004915	Daunorub	15.08	2
RAB33B	83452	Leukemia, MESH:D015470	Dronabinc	15.08	9
RAD51D	5892	Leukemia, MESH:D015470	Arsenic Tr	15.08	14
RETREG3	162427	Leukemia, MESH:D015470	Dronabinc	15.08	6
TOE1	114034	Leukemia, MESH:D015470	Dronabinc	15.08	7
TRA2A	29896	Leukemia, MESH:D015473	Arsenic|Ar	15.08	161
ZC3H10	84872	Leukemia, MESH:D015470	Doxorubic	15.08	11
ZNF398	57541	Leukemia, MESH:D015470	Doxorubic	15.08	10
AMIGO3	386724	Leukemia, MESH:D015470	Dexameth	15.07	6
C15ORF61	145853	Leukemia, MESH:D015470	Tobacco S	15.07	6
CEP131	22994	Leukemia, MESH:D015470	Dexameth	15.07	6
CREM	1390	Leukemia, MESH:D015473	Arsenic|Ar	15.07	162
GALNT5	11227	Leukemia, MESH:D015470	Dexameth	15.07	11
GRIA3	2892	Leukemia, MESH:D015470	Benzoates	15.07	17
H6PD	9563	Leukemia, MESH:D015473	Arsenic|Ar	15.07	160

JADE1	79960	Leukemia, MESH:D015473	Arsenic|ar:	15.07	158
KCNMB3	27094	Leukemia, MESH:D015470	Tobacco S	15.07	6
MAB21L1	4081	Leukemia, MESH:D015470	Dexameth	15.07	7
MBTPS2	51360	Leukemia, MESH:D015470	Dexameth	15.07	11
MSRA	4482	Leukemia, MESH:D015473	Arsenic|Ar	15.07	161
NOVA2	4858	Leukemia, MESH:D015470	Doxorubic	15.07	11
PSD4	23550	Leukemia, MESH:D015470	Doxorubic	15.07	12
RMST	196475	Leukemia, MESH:D015470	Tobacco S	15.07	6
RSAD1	55316	Leukemia, MESH:D015470	Doxorubic	15.07	13
SMPD4	55627	Leukemia, MESH:D015470	Doxorubic	15.07	12
STARD5	80765	Leukemia, MESH:D004915	Doxorubic	15.07	2
STOX1	219736	Leukemia, MESH:D015470	Pentachlo	15.07	7
STRADB	55437	Leukemia, MESH:D015470	Doxorubic	15.07	12
TAF1A	9015	Leukemia, MESH:D015470	Dexameth	15.07	7
TENT5C	54855	Leukemia, MESH:D007948	Arsenic Tr	15.07	4
TMEM87B	84910	Leukemia, MESH:D015470	Calcitriol|D	15.07	12
TOX4	9878	Leukemia, MESH:D015470	Arsenic Tr	15.07	18
TRAF3	7187	Leukemia, MESH:D015473	Arsenic|Ar	15.07	263
UBA1	7317	Leukemia, MESH:D015473	Arsenic|Ar	15.07	159
UCK2	7371	Leukemia, MESH:D015473	Calcitriol|D	15.07	8
HMGCR	3156	Leukemia, MESH:D015473	alpha-Toc	15.06	156
ODF2L	57489	Leukemia, MESH:D015470	Doxorubic	15.06	12
PWWP3B	139221	Leukemia, MESH:D015470	Doxorubic	15.06	12
SPTLC3	55304	Leukemia, MESH:D004915	Cytarabine	15.06	3
TENT2	167153	Leukemia, MESH:D015473	Arsenic Tr	15.06	156
DPH1	1801	Leukemia, MESH:D015473	Dexameth	15.05	156
EDN3	1908	Leukemia, MESH:D015470	Dronabinc	15.05	8
IL2	3558	Leukemia, MESH:D007948	2-(2-amin	15.05	3
RNF19B	127544	Leukemia, MESH:D015473	arsenite|M	15.05	152
ACOT9	23597	Leukemia, MESH:D015470	Arsenic Tr	15.04	34
ATG3	64422	Leukemia, MESH:D015470	Arsenic Tr	15.04	15
COX3	4514	Leukemia, MESH:D015470	Deferoxan	15.04	13
CPN1	1369	Leukemia, MESH:D015470	Dexameth	15.04	10
GRB7	2886	Leukemia, MESH:D015470	Alitretinoi	15.04	13
HEG1	57493	Leukemia, MESH:D015470	Benzene|D	15.04	26
IFT22	64792	Leukemia, MESH:D015473	Arsenic|ar:	15.04	11
LYPLA1	10434	Leukemia, MESH:D015470	Alitretinoi	15.04	19
MTF1	4520	Leukemia, MESH:D015473	Arsenic Tr	15.04	261
PCSK6	5046	Leukemia, MESH:D015473	Arsenic|ca	15.04	12
PHACTR2	9749	Leukemia, MESH:D004915	Cytarabine	15.04	3
PLIN4	729359	Leukemia, MESH:D015470	Bezafibrat	15.04	14
RAD23B	5887	Leukemia, MESH:D007948	Arsenic Tr	15.04	3
SAA3	20210	Leukemia, MESH:D015473	Arsenic|Ch	15.04	12
SLC12A7	10723	Leukemia, MESH:D015470	Air Polluta	15.04	11
SLC27A6	28965	Leukemia, MESH:D015473	Dexameth	15.04	151
SORBS2	8470	Leukemia, MESH:D015473	Cytarabine	15.04	155
SYAP1	94056	Leukemia, MESH:D015473	arsenite|G	15.04	7
ACP1	52	Leukemia, MESH:D015470	Air Polluta	15.03	14
ARSD	414	Leukemia, MESH:D015473	Genistein|I	15.03	152
CDK14	5218	Leukemia, MESH:D015470	Dexameth	15.03	8
CHD4	1108	Leukemia, MESH:D015470	Dexameth	15.03	14
CREG2	200407	Leukemia, MESH:D015473	Calcitriol|G	15.03	153
CYP51A1	1595	Leukemia, MESH:D015473	Arsenic|De	15.03	153
DHODH	1723	Leukemia, MESH:D015470	Doxorubic	15.03	14
DTNA	1837	Leukemia, MESH:D015470	Dexameth	15.03	13
EHD1A	405810	Leukemia, MESH:D015473	Dexameth	15.03	152
GNPDA1	10007	Leukemia, MESH:D015470	Air Polluta	15.03	7
GRHL1	29841	Leukemia, MESH:D015470	Calcitriol|T	15.03	8

KLF11	8462	Leukemia, MESH:D015473	Arsenic Tr	15.03	155
MRPL12	6182	Leukemia, MESH:D015470	Doxorubic	15.03	13
NTD5	386628	Leukemia, MESH:D015473	Dexameth	15.03	152
NXPH1	30010	Leukemia, MESH:D015473	arsenite|D	15.03	153
PHLDB1	23187	Leukemia, MESH:D015470	Dexameth	15.03	8
PPFIA4	8497	Leukemia, MESH:D015470	Dexameth	15.03	9
PPFIBP2	8495	Leukemia, MESH:D015470	Calcitriol|D	15.03	14
RAD23A	5886	Leukemia, MESH:D015473	Arsenic|Ar	15.03	167
SIX6	4990	Leukemia, MESH:D015473	arsenite|D	15.03	153
SOX11	6664	Leukemia, MESH:D015470	Decitabine	15.03	14
SPEN	23013	Leukemia, MESH:D004915	Doxorubic	15.03	2
SRP72	6731	Leukemia, MESH:D015470	Dexameth	15.03	10
SRSF11	9295	Leukemia, MESH:D015470	Dexameth	15.03	14
UBE2J2	118424	Leukemia, MESH:D015473	Arsenic|De	15.03	9
WDR59	79726	Leukemia, MESH:D015473	Arsenic|De	15.03	153
ZDHHC7	55625	Leukemia, MESH:D015473	Arsenic|De	15.03	156
ELAVL1	1994	Leukemia, MESH:D007948	Arsenic Tr	15.02	3
ENDOG	2021	Leukemia, MESH:D007948	pyrazolant	15.02	2
EPB41L1	2036	Leukemia, MESH:D015473	Arsenic Tr	15.02	260
MYO5A	4644	Leukemia, MESH:D015473	Arsenic|ars	15.02	158
NRXN2	9379	Leukemia, MESH:D015470	Dexameth	15.02	13
PDLIM4	8572	Leukemia, MESH:D004915	Cytarabine	15.02	3
SPDL1	54908	Leukemia, MESH:D015473	Arsenic|Ca	15.02	158
XXYLT1	152002	Leukemia, MESH:D015473	Arsenic|Ge	15.02	155
ADORA1	134	Leukemia, MESH:D015473	Arsenic|ars	15.01	14
AOX1	316	Leukemia, MESH:D015473	Dasatinib|I	15.01	9
CYP19A1	1588	Leukemia, MESH:D015473	arsenite|Ca	15.01	158
GRB14	2888	Leukemia, MESH:D015473	Arsenic|ars	15.01	157
PCOLCE	5118	Leukemia, MESH:D015473	Arsenic Tr	15.01	260
PCSK5	5125	Leukemia, MESH:D007948	Arsenic Tr	15.01	4
PPT1	5538	Leukemia, MESH:D015473	Dexameth	15.01	155
REG3B	18489	Leukemia, MESH:D015470	Cyclophos	15.01	11
RIOK3	8780	Leukemia, MESH:D015473	Arsenic|De	15.01	158
TBK1	29110	Leukemia, MESH:D015473	Dexameth	15.01	155
TRO	7216	Leukemia, MESH:D015473	arsenite|D	15.01	154
TUBA1A	7846	Leukemia, MESH:D015473	Calcitriol|D	15.01	9
KLF9	687	Leukemia, MESH:D015473	Arsenic Tr	15	265
SULT1C3	442038	Leukemia, MESH:D015473	Antimony	15	6
TSPO	706	Leukemia, MESH:D015473	arsenite|Cy	15	159
VSIG4	11326	Leukemia, MESH:D01 marker/me	Arsenic Tr	14.99	15
ANP32B	10541	Leukemia, MESH:D004915	Doxorubic	14.99	2
DBN1	1627	Leukemia, MESH:D007948	Arsenic Tr	14.99	4
ILKAP	80895	Leukemia, MESH:D015473	Arsenic Tr	14.99	262
MAPK13	5603	Leukemia, MESH:D007948	Arsenic Tr	14.99	3
RGL1	23179	Leukemia, MESH:D015473	Arsenic Tr	14.99	166
SRSF3	6428	Leukemia, MESH:D015473	arsenite|so	14.99	158
TOB1	10140	Leukemia, MESH:D015473	Antimony	14.99	152
USP22	23326	Leukemia, MESH:D015470	Methotrex	14.99	9
WNT9B	7484	Leukemia, MESH:D015473	arsenic dis	14.99	5
APIP	51074	Leukemia, MESH:D015470	Gasoline|I	14.98	6
ATP5J	11957	Leukemia, MESH:D015470	Doxorubic	14.98	11
CDC7	8317	Leukemia, MESH:D015473	Arsenic Tr	14.98	261
COL20A1	57642	Leukemia, MESH:D015470	Dronabino	14.98	6
PLLP	51090	Leukemia, MESH:D004915	Cytarabine	14.98	3
SLC39A2	29986	Leukemia, MESH:D015473	Arsenic|Ca	14.98	18
TBL1X	6907	Leukemia, MESH:D004915	Doxorubic	14.98	3
TESK2	10420	Leukemia, MESH:D015470	Dronabino	14.98	6
XKRX	402415	Leukemia, MESH:D015473	Arsenic|ars	14.98	10

ANKZF1	55139	Leukemia, MESH:D015470	Dexameth	14.97	14
ATP6V0A1	535	Leukemia, MESH:D015470	Dexameth	14.97	10
ATP8B4	79895	Leukemia, MESH:D015470	Cytarabine	14.97	75
CUTA	51596	Leukemia, MESH:D015470	Doxorubic	14.97	13
EXTL2	2135	Leukemia, MESH:D015470	Doxorubic	14.97	21
FAM217B	63939	Leukemia, MESH:D015470	Dronabinc	14.97	7
FILIP1L	11259	Leukemia, MESH:D015473	Arsenic|ars	14.97	157
FMO3	2328	Leukemia, MESH:D015470	Dexameth	14.97	10
GMNN	51053	Leukemia, MESH:D015470	Calcitriol|D	14.97	11
ITIH2	3698	Leukemia, MESH:D015470	Dexameth	14.97	7
LAMP3	27074	Leukemia, MESH:D015473	Dexameth	14.97	154
LBR	3930	Leukemia, MESH:D015473	Calcitriol|D	14.97	156
MARCO	8685	Leukemia, MESH:D015470	Decitabine	14.97	14
MOBP	4336	Leukemia, MESH:D015470	Benzene|D	14.97	22
NUDCD2	134492	Leukemia, MESH:D015470	Dexameth	14.97	8
PIGM	93183	Leukemia, MESH:D015470	Air Polluta	14.97	12
PRICKLE1	144165	Leukemia, MESH:D015473	arsenite|Cy	14.97	157
RABGAP1l	9910	Leukemia, MESH:D015473	Arsenic|Ca	14.97	158
RABIF	5877	Leukemia, MESH:D015470	Dexameth	14.97	14
RPE	6120	Leukemia, MESH:D015470	Air Polluta	14.97	12
RPS24	6229	Leukemia, MESH:D015470	Benzene|Ir	14.97	24
SNRPD1	6632	Leukemia, MESH:D015470	Dexameth	14.97	9
SOCS7	30837	Leukemia, MESH:D015470	Cyclophos	14.97	13
SPC24	147841	Leukemia, MESH:D015473	Calcitriol|M	14.97	155
TMCO6	55374	Leukemia, MESH:D015470	Arsenic Tr	14.97	21
TMEM259	91304	Leukemia, MESH:D015470	Doxorubic	14.97	13
TUFM	7284	Leukemia, MESH:D015470	Doxorubic	14.97	22
ZCCHC17	51538	Leukemia, MESH:D015470	Dexameth	14.97	6
ZNHIT3	9326	Leukemia, MESH:D015470	Dexameth	14.97	7
ABCC2	1244	Leukemia, MESH:D007948	2-(2-amin	14.96	3
ABHD17A	81926	Leukemia, MESH:D015470	Dronabinc	14.96	7
ADAM10	102	Leukemia, MESH:D015473	Arsenic|De	14.96	158
ADGRG2	10149	Leukemia, MESH:D015470	Arsenic Tr	14.96	26
ADGRG6	57211	Leukemia, MESH:D015470	Doxorubic	14.96	13
CERS2	29956	Leukemia, MESH:D015470	Dronabinc	14.96	9
CLUAP1	23059	Leukemia, MESH:D015470	Doxorubic	14.96	12
CNTN1	1272	Leukemia, MESH:D015473	Arsenic Tr	14.96	262
EDRF1	26098	Leukemia, MESH:D015470	Arsenic Tr	14.96	14
FKBP14	55033	Leukemia, MESH:D015470	Doxorubic	14.96	13
GPR155	151556	Leukemia, MESH:D015470	Dexameth	14.96	8
HS3ST3A1	9955	Leukemia, MESH:D015470	Calcitriol|D	14.96	7
MRNIP	51149	Leukemia, MESH:D015470	Calcitriol|T	14.96	7
PQBP1	10084	Leukemia, MESH:D015470	Doxorubic	14.96	14
PRAM1	84106	Leukemia, MESH:D015470	Arsenic Tr	14.96	12
PTGFR	5737	Leukemia, MESH:D015470	Arsenic Tr	14.96	14
PURG	29942	Leukemia, MESH:D015470	Arsenic Tr	14.96	12
RCC1	1104	Leukemia, MESH:D015470	Doxorubic	14.96	14
RPUSD3	285367	Leukemia, MESH:D015470	Decitabine	14.96	19
SKP1	6500	Leukemia, MESH:D015470	Decitabine	14.96	14
SLC66A3	130814	Leukemia, MESH:D015470	Dexameth	14.96	7
STARD3NI	83930	Leukemia, MESH:D015470	Dexameth	14.96	11
TBCA	6902	Leukemia, MESH:D015470	Doxorubic	14.96	14
TGFBR3	7049	Leukemia, MESH:D007948	Arsenic Tr	14.96	4
ZBTB41	360023	Leukemia, MESH:D015470	Cyclophos	14.96	7
AHSA2P	130872	Leukemia, MESH:D015473	Arsenic|Ar	14.95	160
AOAH	313	Leukemia, MESH:D015470	(+)-JQ1 cc	14.95	7
ATP9B	374868	Leukemia, MESH:D015473	Arsenic|ars	14.95	10
B3GALT2	8707	Leukemia, MESH:D015470	Doxorubic	14.95	13

CCNYL1	151195	Leukemia, MESH:D015470	(+)-JQ1 cc	14.95	7
CEP112	201134	Leukemia, MESH:D015470	Dexameth	14.95	6
DGUOK	1716	Leukemia, MESH:D015470	Doxorubic	14.95	13
FAM161A	84140	Leukemia, MESH:D015470	Dronabinc	14.95	7
GUCD1	83606	Leukemia, MESH:D015470	Dexameth	14.95	11
HOXA3	3200	Leukemia, MESH:D015470	Arsenic Tr	14.95	17
JCAD	57608	Leukemia, MESH:D015470	Doxorubic	14.95	12
KLHL3	26249	Leukemia, MESH:D015470	Dexameth	14.95	11
MAN2B1	4125	Leukemia, MESH:D004915	Doxorubic	14.95	2
NOL7	51406	Leukemia, MESH:D015470	Dexameth	14.95	11
NOL8	55035	Leukemia, MESH:D004915	Doxorubic	14.95	2
NR6A1	2649	Leukemia, MESH:D015470	Dexameth	14.95	7
SEPTIN6	23157	Leukemia, MESH:D004915	Doxorubic	14.95	2
SLX4	84464	Leukemia, MESH:D015470	Dexameth	14.95	11
TLL2	7093	Leukemia, MESH:D015473	Arsenic Tr	14.95	258
TOP2A	7153	Leukemia, MESH:D004915	Daunorub	14.95	2
TRMT9B	57604	Leukemia, MESH:D015470	Doxorubic	14.95	12
AHSP	51327	Leukemia, MESH:D015473	Arsenic|Ge	14.94	9
BAALC	79870	Leukemia, MESH:D015473	Arsenic|Ar	14.94	261
BAIAP2L2	80115	Leukemia, MESH:D015473	Calcitriol|D	14.94	152
BCL2L2	599	Leukemia, MESH:D015473	Arsenic Tr	14.94	156
PGM3	5238	Leukemia, MESH:D015473	Antimony	14.94	261
FGF2	2247	Leukemia, MESH:D004915	Doxorubic	14.93	3
WDR86	349136	Leukemia, MESH:D015473	Calcitriol|G	14.93	153
AMACR	23600	Leukemia, MESH:D015473	Arsenic Tr	14.92	261
CXCR2	3579	Leukemia, MESH:D015470	Air Polluta	14.92	18
KIF18A	81930	Leukemia, MESH:D015473	arsenite|Ca	14.92	157
LIN52	91750	Leukemia, MESH:D015473	arsenite|M	14.92	153
MIR196B	442920	Leukemia, MESH:D015473	Arsenic|Et	14.92	10
MRPL27	51264	Leukemia, MESH:D015473	arsenite|D	14.92	8
OPA1	4976	Leukemia, MESH:D015470	Arsenic Tr	14.92	21
OSCAR	126014	Leukemia, MESH:D015473	Arsenic Tr	14.92	260
PDCD6IP	10015	Leukemia, MESH:D015473	Calcitriol|D	14.92	154
PRKAR2B	5577	Leukemia, MESH:D015473	Arsenic|ars	14.92	13
SLC13A1	6561	Leukemia, MESH:D015473	arsenite|D	14.92	6
C1ORF21	81563	Leukemia, MESH:D015473	Calcitriol|D	14.91	153
CCSER1	401145	Leukemia, MESH:D015473	Arsenic|Ge	14.91	155
DDC	1644	Leukemia, MESH:D015473	arsenite|D	14.91	157
ECI2	10455	Leukemia, MESH:D015473	arsenite|D	14.91	157
GREM1	26585	Leukemia, MESH:D007948	2-(2-amin	14.91	2
GSK3B	2932	Leukemia, MESH:D007948	2-(2-amin	14.91	3
IDH3G	3421	Leukemia, MESH:D004915	Doxorubic	14.91	2
MIR491	574444	Leukemia, MESH:D015470	Arsenic Tr	14.91	28
MKS1	54903	Leukemia, MESH:D015473	Calcitriol|D	14.91	153
MRPS5	64969	Leukemia, MESH:D015473	Arsenic|ars	14.91	10
PFKMA	447836	Leukemia, MESH:D015470	Alitretinoi	14.91	6
PHB2	11331	Leukemia, MESH:D015470	Doxorubic	14.91	20
PLPP4	196051	Leukemia, MESH:D015473	Calcitriol|D	14.91	153
TRIM27	5987	Leukemia, MESH:D004915	Doxorubic	14.91	2
ARIH1	25820	Leukemia, MESH:D004915	Doxorubic	14.9	3
CXADR	1525	Leukemia, MESH:D015473	Arsenic|Ca	14.9	12
DAPK3	1613	Leukemia, MESH:D015470	Decitabine	14.9	15
EIF4ENIF1	56478	Leukemia, MESH:D015473	Arsenic|De	14.9	155
IPO5	3843	Leukemia, MESH:D015473	arsenite|Ca	14.9	157
MYL2	4633	Leukemia, MESH:D004915	Doxorubic	14.9	3
PIK3R2	5296	Leukemia, MESH:D015470	Arsenic Tr	14.9	17
WRNIP1	56897	Leukemia, MESH:D015473	caffeic aci	14.9	6
ZNF430	80264	Leukemia, MESH:D015473	arsenite|D	14.9	154

ADAM12	8038	Leukemia, MESH:D015470	Dexameth	14.89	13
ADCY3	109	Leukemia, MESH:D015470	Dexameth	14.89	9
ADGRL2	23266	Leukemia, MESH:D015470	Dexameth	14.89	14
CCNC	892	Leukemia, MESH:D015470	Calcitriol|[illegible]	14.89	8
CSH1	1442	Leukemia, MESH:D015470	Arsenic Tr	14.89	18
DUSP16	80824	Leukemia, MESH:D015470	Arsenic Tr	14.89	17
IRAK3	11213	Leukemia, MESH:D015470	Doxorubic	14.89	13
NTN4	59277	Leukemia, MESH:D015470	Doxorubic	14.89	14
PCM1	5108	Leukemia, MESH:D015470	Arsenic Tr	14.89	13
RPS6KA1	6195	Leukemia, MESH:D007948	pyrazolant	14.89	2
SH3GLB1	51100	Leukemia, MESH:D015470	Calcitriol|[illegible]	14.89	14
SLC39A6	25800	Leukemia, MESH:D015470	Calcitriol|[illegible]	14.89	12
STX1A	6804	Leukemia, MESH:D015470	Doxorubic	14.89	15
WDR43	23160	Leukemia, MESH:D015470	Dexameth	14.89	9
CBX5	23468	Leukemia, MESH:D015473	Genistein|[illegible]	14.88	155
FNBP1	23048	Leukemia, MESH:D015473	arsenite|D	14.88	7
SLC16A9B	445158	Leukemia, MESH:D015470	Dronabinc	14.88	5
SLC28A3	64078	Leukemia, MESH:D015473	Arsenic Tr	14.88	153
UGT2B	24862	Leukemia, MESH:D015470	Alitretinoi	14.88	8
ZNF333	84449	Leukemia, MESH:D015470	Arsenic Tr	14.88	18
ZNF700	90592	Leukemia, MESH:D015470	Benzene|T	14.88	19
CRB2	286204	Leukemia, MESH:D015470	Dronabinc	14.87	6
EIF2S3	1968	Leukemia, MESH:D015473	Calcitriol|s	14.87	156
FHL5	9457	Leukemia, MESH:D015470	Dronabinc	14.87	8
GCM1	8521	Leukemia, MESH:D015470	Dronabinc	14.87	9
MCF2L	23263	Leukemia, MESH:D015473	Arsenic|Ar	14.87	159
METRNL	284207	Leukemia, MESH:D015473	Arsenic|Ca	14.87	157
NDUFS4	4724	Leukemia, MESH:D015473	arsenite|D	14.87	8
NET1	10276	Leukemia, MESH:D015473	Calcitriol|[illegible]	14.87	153
OPTN	10133	Leukemia, MESH:D007948	Arsenic Tr	14.87	3
ZNF69	7620	Leukemia, MESH:D015470	Doxorubic	14.87	10
ABI3	51225	Leukemia, MESH:D015470	Air Polluta	14.86	5
DDR2	4921	Leukemia, MESH:D007948	Arsenic Tr	14.86	4
DYNLT5	200132	Leukemia, MESH:D015470	Decitabine	14.86	11
EDEM1	9695	Leukemia, MESH:D015473	Arsenic|Ar	14.86	263
EIF4B	1975	Leukemia, MESH:D007948	Arsenic Tr	14.86	3
FAM161B	145483	Leukemia, MESH:D015470	Dronabinc	14.86	10
FAM81A	145773	Leukemia, MESH:D015470	Calcitriol|[illegible]	14.86	11
KIF9	64147	Leukemia, MESH:D015470	Bortezomi	14.86	10
MIR205HC	642587	Leukemia, MESH:D015470	Methotrex	14.86	7
NDRG4	65009	Leukemia, MESH:D015473	Arsenic Tr	14.86	262
NEK3	4752	Leukemia, MESH:D015470	Doxorubic	14.86	11
PODXL2	50512	Leukemia, MESH:D015470	Cytarabine	14.86	74
RAB22A	57403	Leukemia, MESH:D015470	Benzene|[illegible]	14.86	26
ATP13A1	57130	Leukemia, MESH:D015470	Doxorubic	14.85	13
C12ORF54	121273	Leukemia, MESH:D015473	Arsenic|ars	14.85	9
CDK20	23552	Leukemia, MESH:D015470	Doxorubic	14.85	11
CDKL3	51265	Leukemia, MESH:D015470	Dexameth	14.85	6
CISD2	493856	Leukemia, MESH:D015470	Dexameth	14.85	14
CNIH3	149111	Leukemia, MESH:D015470	Dexameth	14.85	7
DNAJB14	79982	Leukemia, MESH:D015470	Calcitriol|[illegible]	14.85	8
DNHD1	144132	Leukemia, MESH:D015470	Dexameth	14.85	6
FIGNL1	63979	Leukemia, MESH:D015473	arsenite|Ca	14.85	155
KCTD6	200845	Leukemia, MESH:D015470	Arsenic Tr	14.85	18
LSM12	124801	Leukemia, MESH:D015470	Air Polluta	14.85	18
MEIS3	56917	Leukemia, MESH:D015470	Dexameth	14.85	13
PDCD2L	84306	Leukemia, MESH:D015470	Doxorubic	14.85	13
PKN3	29941	Leukemia, MESH:D015470	Dexameth	14.85	7

POLL	27343	Leukemia, MESH:D015470		Decitabine	14.85	18
PPIP5K1	9677	Leukemia, MESH:D015470		Calcitriol|D	14.85	11
PRAF2	11230	Leukemia, MESH:D015470		Doxorubic	14.85	12
RWDD1	51389	Leukemia, MESH:D015473		Arsenic|Cy	14.85	18
SLC35F1	222553	Leukemia, MESH:D015470		Doxorubic	14.85	12
SLC51A	200931	Leukemia, MESH:D015473		caffeic aci	14.85	151
SYNPO2L	79933	Leukemia, MESH:D015473		Arsenic|ars	14.85	9
TIAM2	26230	Leukemia, MESH:D015470		Doxorubic	14.85	11
TOMM22	56993	Leukemia, MESH:D015470		Indometha	14.85	9
TTC8	123016	Leukemia, MESH:D015470		Doxorubic	14.85	12
ZNF175	7728	Leukemia, MESH:D015470		Doxorubic	14.85	12
ZNF25	219749	Leukemia, MESH:D015470		Doxorubic	14.85	11
ACTA2	59	Leukemia, MESH:D007948		Arsenic Tr	14.84	3
AJAP1	55966	Leukemia, MESH:D015470		Methotrex	14.84	9
CADM2	253559	Leukemia, MESH:D015470		Dexameth	14.84	7
CCDC59	29080	Leukemia, MESH:D015470		Dexameth	14.84	6
CENPK	64105	Leukemia, MESH:D015473		Calcitriol|D	14.84	152
CHI3L1	1116	Leukemia, MESH:D007948		2-(2-amin	14.84	3
COA3	28958	Leukemia, MESH:D015470		Dexameth	14.84	11
DCUN1D5	84259	Leukemia, MESH:D015470		Doxorubic	14.84	13
EXOC4	60412	Leukemia, MESH:D004915		Doxorubic	14.84	2
GMEB1	10691	Leukemia, MESH:D015470		Doxorubic	14.84	12
GUSBP2	387036	Leukemia, MESH:D015470		Dexameth	14.84	5
IKBKE	9641	Leukemia, MESH:D015473		Arsenic|Ca	14.84	21
KANSL1L	151050	Leukemia, MESH:D015470		Doxorubic	14.84	12
KLC2	64837	Leukemia, MESH:D015470		Dexameth	14.84	11
LINC0017	285908	Leukemia, MESH:D015470		Dexameth	14.84	5
LMAN2	10960	Leukemia, MESH:D015470		Dexameth	14.84	8
MED18	54797	Leukemia, MESH:D015470		Dexameth	14.84	7
NBEA	26960	Leukemia, MESH:D015470		Doxorubic	14.84	12
PCDH12	51294	Leukemia, MESH:D015470		Dexameth	14.84	12
PDE3A	5139	Leukemia, MESH:D015473		Arsenic|Ca	14.84	155
PGBD5	79605	Leukemia, MESH:D015470		Calcitriol|D	14.84	12
RIMBP2	23504	Leukemia, MESH:D015470		Arsenic Tr	14.84	12
SLC10A2	6555	Leukemia, MESH:D015473		Calcitriol|D	14.84	7
TCHP	84260	Leukemia, MESH:D015470		Indometha	14.84	9
TDH	157739	Leukemia, MESH:D015470		Dexameth	14.84	7
TIMM50	92609	Leukemia, MESH:D015470		Doxorubic	14.84	13
TIMM8A	1678	Leukemia, MESH:D015470		Resveratro	14.84	9
TMEM131	23240	Leukemia, MESH:D015470		Dronabino	14.84	7
TMEM170	1E+08	Leukemia, MESH:D015470		Dexameth	14.84	7
UMAD1	729852	Leukemia, MESH:D015470		Dexameth	14.84	10
ZNF224	7767	Leukemia, MESH:D015470		Arsenic Tr	14.84	12
ITGAX	3687	Leukemia, MESH:D01	marker/m	Arsenic Tr	14.83	262
ACE2	59272	Leukemia, MESH:D015470		Chloramp	14.83	24
ASGR1	432	Leukemia, MESH:D015470		Decitabine	14.83	13
CDX1	1044	Leukemia, MESH:D015473		Arsenic Tr	14.83	259
EFEMP2	30008	Leukemia, MESH:D004915		Cytarabine	14.83	3
EPN2	22905	Leukemia, MESH:D004915		Doxorubic	14.83	2
FAM184A	79632	Leukemia, MESH:D015470		Dexameth	14.83	7
FCGR3	14131	Leukemia, MESH:D015470		Cyclophos	14.83	13
HCFC1	3054	Leukemia, MESH:D004915		Doxorubic	14.83	2
MIR223	407008	Leukemia, MESH:D015473		Arsenic Tr	14.83	260
MLST8	64223	Leukemia, MESH:D015470		Doxorubic	14.83	12
NARS1	4677	Leukemia, MESH:D015470		Air Polluta	14.83	15
NFATC4	4776	Leukemia, MESH:D015470		Carboplati	14.83	9
PLAC1	10761	Leukemia, MESH:D015473		Arsenic Tr	14.83	261
RPS7	6201	Leukemia, MESH:D015473		arsenite|Bu	14.83	155

SPRED2	200734	Leukemia, MESH:D015470	Benzoates	14.83	13
TOB2	10766	Leukemia, MESH:D004915	Doxorubic	14.83	2
UBAP2L	9898	Leukemia, MESH:D004915	Doxorubic	14.83	2
VAMP2	6844	Leukemia, MESH:D015470	Dexameth	14.83	15
ZNF354C	30832	Leukemia, MESH:D015470	Doxorubic	14.83	10
ZNF814	730051	Leukemia, MESH:D015470	Doxorubic	14.83	11
ALDH7A1	501	Leukemia, MESH:D015470	Arsenic Tr	14.82	20
ATG12	9140	Leukemia, MESH:D007948	2-(2-amin	14.82	3
CHD2	1106	Leukemia, MESH:D015473	Arsenic|Ar	14.82	160
CPLX2	10814	Leukemia, MESH:D015470	Dexameth	14.82	7
CSNK1D	1453	Leukemia, MESH:D015470	Arsenic Tr	14.82	80
DOCK9	23348	Leukemia, MESH:D015470	Air Polluta	14.82	13
EBAG9	9166	Leukemia, MESH:D015473	arsenite|D	14.82	6
FLOT1	10211	Leukemia, MESH:D015470	Dexameth	14.82	14
GCAT	23464	Leukemia, MESH:D015470	Dexameth	14.82	14
GSTK1	373156	Leukemia, MESH:D015470	Decitabine	14.82	19
HSD17B12	51144	Leukemia, MESH:D015470	Calcitriol|D	14.82	13
LRRC59	55379	Leukemia, MESH:D015470	Arsenic Tr	14.82	19
MID1	4281	Leukemia, MESH:D015470	Doxorubic	14.82	13
MYOD1	4654	Leukemia, MESH:D015470	Azacitidine	14.82	11
NEFL	4747	Leukemia, MESH:D015473	Arsenic|ars	14.82	158
NFIB	4781	Leukemia, MESH:D015470	Benzene|C	14.82	96
NPR2	4882	Leukemia, MESH:D015470	Dexameth	14.82	12
RALGDS	5900	Leukemia, MESH:D015470	Decitabine	14.82	19
RETREG1	54463	Leukemia, MESH:D015470	Dexameth	14.82	12
TOR1A	1861	Leukemia, MESH:D004915	Doxorubic	14.82	3
UBE2B	7320	Leukemia, MESH:D015470	Arsenic Tr	14.82	19
WNT11	7481	Leukemia, MESH:D015470	Dexameth	14.82	12
HSP70.3	30671	Leukemia, MESH:D015473	Arsenic|Ar	14.81	263
METTL14	57721	Leukemia, MESH:D015473	arsenite|G	14.81	154
MIRLET7D	406886	Leukemia, MESH:D015473	Arsenic|Ar	14.81	263
RNF220	55182	Leukemia, MESH:D015473	Arsenic|ars	14.81	10
RPS15A	6210	Leukemia, MESH:D015473	Arsenic Tr	14.81	262
TERC	7012	Leukemia, MESH:D015473	Arsenic|Ar	14.81	263
TXLNB	167838	Leukemia, MESH:D015473	Arsenic|ars	14.81	156
WT1-AS	51352	Leukemia, MESH:D015473	sodium ar	14.81	155
DBF4	10926	Leukemia, MESH:D015473	arsenite|Ca	14.8	155
ETFA	2108	Leukemia, MESH:D007948	Arsenic Tr	14.8	3
EXOG	9941	Leukemia, MESH:D015473	Arsenic Tr	14.8	262
PAMR1	25891	Leukemia, MESH:D004915	Cytarabine	14.8	2
PARM1	25849	Leukemia, MESH:D004915	Cytarabine	14.8	2
PSPH	5723	Leukemia, MESH:D015473	Antimony	14.8	260
PTEN	5728	Leukemia, MESH:D004915	Cytarabine	14.8	2
RAX	30062	Leukemia, MESH:D015473	arsenite|D	14.8	154
VEGFA	7422	Leukemia, MESH:D004915	Daunorub	14.8	3
DDX39A	10212	Leukemia, MESH:D015473	Calcitriol|D	14.79	153
EPOR	2057	Leukemia, MESH:D015473	Arsenic Tr	14.79	260
FAM180A	389558	Leukemia, MESH:D015473	arsenite|D	14.79	5
GDF15	9518	Leukemia, MESH:D004915	Daunorub	14.79	2
GPC3	2719	Leukemia, MESH:D015473	Arsenic Tr	14.79	259
LIN7A	8825	Leukemia, MESH:D015473	Calcitriol|C	14.79	153
TFDP1	7027	Leukemia, MESH:D007948	Arsenic Tr	14.79	3
OLFML3	56944	Leukemia, MESH:D007948	Arsenic Tr	14.78	4
TMSB4X	7114	Leukemia, MESH:D015473	Dexameth	14.78	155
ZYX	7791	Leukemia, MESH:D015473	arsenite|Ca	14.78	154
CLIC1	1192	Leukemia, MESH:D015473	Arsenic|ars	14.77	159
MAPK6	5597	Leukemia, MESH:D015470	Air Polluta	14.77	9
MLKL	197259	Leukemia, MESH:D015473	Dexameth	14.77	155

MPG	4350	Leukemia, MESH:D015470	Bezafibrat	14.77	14
RPL23	9349	Leukemia, MESH:D015473	Arsenic Tr	14.77	262
SCD2	20250	Leukemia, MESH:D015473	Arsenic|De	14.77	11
SCG5	6447	Leukemia, MESH:D015470	Decitabine	14.77	17
TRA2B	6434	Leukemia, MESH:D015473	Arsenic|Ar	14.77	264
UMPS	7372	Leukemia, MESH:D015473	Arsenic|ars	14.77	156
YWHAQ	10971	Leukemia, MESH:D015473	Arsenic Tr	14.77	155
COG5	10466	Leukemia, MESH:D015470	Arsenic Tr	14.76	18
CSNK1E	1454	Leukemia, MESH:D015470	Benzene|C	14.76	22
FXR1	8087	Leukemia, MESH:D015470	Doxorubic	14.76	13
HSPB11	51668	Leukemia, MESH:D015470	Decitabine	14.76	18
KDELR1	10945	Leukemia, MESH:D015473	Arsenic|ars	14.76	9
MTUS1	57509	Leukemia, MESH:D007948	Arsenic Tr	14.76	4
RASA3	22821	Leukemia, MESH:D015470	Azacitidine	14.76	8
SLC13A5	284111	Leukemia, MESH:D015470	Decitabine	14.76	20
SYDE2	84144	Leukemia, MESH:D015470	Doxorubic	14.76	11
SYT15	83849	Leukemia, MESH:D015473	Dasatinib|	14.76	152
SYT8	90019	Leukemia, MESH:D015470	Calcitriol|D	14.76	5
TRIM28	10155	Leukemia, MESH:D015470	Doxorubic	14.76	16
WASHC4	23325	Leukemia, MESH:D015470	Air Polluta	14.76	9
ADAM17	6868	Leukemia, MESH:D007948	pyrazolant	14.75	2
ADAM9	8754	Leukemia, MESH:D015470	Dexameth	14.75	11
ATP5MF	9551	Leukemia, MESH:D015470	Doxorubic	14.75	10
COX16	51241	Leukemia, MESH:D015470	Dexameth	14.75	6
DSG3	1830	Leukemia, MESH:D015473	Calcitriol|C	14.75	5
GRINA	2907	Leukemia, MESH:D015470	Dexameth	14.75	10
H4C11	8363	Leukemia, MESH:D015473	Arsenic|Ar	14.75	157
HOXB4	3214	Leukemia, MESH:D015473	Arsenic|Cy	14.75	157
IPO4	79711	Leukemia, MESH:D015470	Dexameth	14.75	13
MAGI2	9863	Leukemia, MESH:D015470	Decitabine	14.75	19
MALT1	10892	Leukemia, MESH:D015470	Calcitriol|D	14.75	15
MED24	9862	Leukemia, MESH:D015470	Doxorubic	14.75	12
NETO1	81832	Leukemia, MESH:D015470	Calcitriol|D	14.75	13
PPP2R1B	5519	Leukemia, MESH:D015470	Dexameth	14.75	8
PSMA7	5688	Leukemia, MESH:D015470	Arsenic Tr	14.75	18
SHANK3	85358	Leukemia, MESH:D015470	Decitabine	14.75	19
SLC6A13	6540	Leukemia, MESH:D015470	Dronabinc	14.75	9
SNAP23	8773	Leukemia, MESH:D015470	Resveratrc	14.75	11
SPAG9	9043	Leukemia, MESH:D015470	Benzene|D	14.75	22
SPEG	10290	Leukemia, MESH:D004915	Doxorubic	14.75	2
TAF5L	27097	Leukemia, MESH:D015470	Doxorubic	14.75	14
TCF21	6943	Leukemia, MESH:D004915	Cytarabine	14.75	3
TPCN2	219931	Leukemia, MESH:D015470	Benzene|T	14.75	23
TRAC	28755	Leukemia, MESH:D015470	Dronabinc	14.75	7
TRIM59	286827	Leukemia, MESH:D015470	Calcitriol|F	14.75	10
ZNF229	7772	Leukemia, MESH:D015473	Arsenic|M	14.75	8
ABCC8	6833	Leukemia, MESH:D015473	Dexameth	14.74	7
ACVR1B	91	Leukemia, MESH:D015473	Arsenic|ars	14.74	154
ALPK3	57538	Leukemia, MESH:D015470	Calcitriol|D	14.74	12
C12ORF73	728568	Leukemia, MESH:D015473	Arsenic|ars	14.74	154
CCDC71L	168455	Leukemia, MESH:D015470	Dexameth	14.74	8
CNIH1	10175	Leukemia, MESH:D015470	Air Polluta	14.74	12
DDX28	55794	Leukemia, MESH:D015470	Decitabine	14.74	15
DGCR6	8214	Leukemia, MESH:D015470	Doxorubic	14.74	13
GABRR1	2569	Leukemia, MESH:D015470	Arsenic Tr	14.74	12
HPS4	89781	Leukemia, MESH:D015470	Doxorubic	14.74	13
KCNK12	56660	Leukemia, MESH:D015470	Arsenic Tr	14.74	14
LAMC1	3915	Leukemia, MESH:D007948	Arsenic Tr	14.74	3

LRRC3B	116135	Leukemia, MESH:D015470		Arsenic Tr	14.74	18
LSM14B	149986	Leukemia, MESH:D015470		Methotrex	14.74	9
MEX3D	399664	Leukemia, MESH:D015470		Arsenic Tr	14.74	18
NBN	4683	Leukemia, MESH:D015473		Arsenic Tr	14.74	260
PDIA2	64714	Leukemia, MESH:D015470		Dexameth	14.74	6
PHKG2	5261	Leukemia, MESH:D015470		Arsenic Tr	14.74	17
PLEKHM3	389072	Leukemia, MESH:D015470		Doxorubic	14.74	11
PRKRIP1	79706	Leukemia, MESH:D015470		Doxorubic	14.74	12
SCRG1	11341	Leukemia, MESH:D015470		Indometh	14.74	8
SMARCC2	6601	Leukemia, MESH:D015470		Doxorubic	14.74	12
SNHG32	50854	Leukemia, MESH:D015470		Arsenic Tr	14.74	19
SUCLG2	8801	Leukemia, MESH:D015473		Arsenic Tr	14.74	155
SYDE1	85360	Leukemia, MESH:D015470		Calcitriol|D	14.74	11
TAGAP	117289	Leukemia, MESH:D015470		Arsenic Tr	14.74	12
TMEM131	23505	Leukemia, MESH:D015470		Doxorubic	14.74	15
TRUB1	142940	Leukemia, MESH:D015470		Arsenic Tr	14.74	13
TXN2	25828	Leukemia, MESH:D015473		Arsenic|Ar	14.74	161
ZKSCAN5	23660	Leukemia, MESH:D015470		Doxorubic	14.74	11
ZSWIM5	57643	Leukemia, MESH:D015470		Doxorubic	14.74	10
AARD	441376	Leukemia, MESH:D015473		Calcitriol|C	14.73	153
ATP2C2	9914	Leukemia, MESH:D015470		Methotrex	14.73	9
BEGAIN	57596	Leukemia, MESH:D015470		Dexameth	14.73	7
BEND7	222389	Leukemia, MESH:D015470		Doxorubic	14.73	13
CDC40	51362	Leukemia, MESH:D015470		Dexameth	14.73	11
CHST4	10164	Leukemia, MESH:D015470		(+)-JQ1 cc	14.73	7
COX5A	9377	Leukemia, MESH:D015473		Arsenic|Ar	14.73	161
CYP4A14	13119	Leukemia, MESH:D015470		Allopurinc	14.73	13
CYP4F11	57834	Leukemia, MESH:D015473		Arsenic Tr	14.73	261
DGAT1	8694	Leukemia, MESH:D015470		Air Polluta	14.73	16
DSC2	1824	Leukemia, MESH:D007948		Arsenic Tr	14.73	4
EBF1	1879	Leukemia, MESH:D015473		arsenite|D	14.73	155
EID2	163126	Leukemia, MESH:D015470		Dexameth	14.73	12
EXOSC10	5394	Leukemia, MESH:D015470		Dexameth	14.73	9
FAM53C	51307	Leukemia, MESH:D015470		Dronabinc	14.73	6
GRAMD4	23151	Leukemia, MESH:D015470		Calcitriol|D	14.73	11
HOXC10	3226	Leukemia, MESH:D015473		arsenite|D	14.73	152
HSD17B11	51170	Leukemia, MESH:D015473		arsenite|Ca	14.73	155
INTS6L	203522	Leukemia, MESH:D015473		Arsenic|Ca	14.73	9
KLRD1	3824	Leukemia, MESH:D015473		arsenite|D	14.73	6
OXNAD1	92106	Leukemia, MESH:D015470		Doxorubic	14.73	12
PIK3C3	5289	Leukemia, MESH:D015473		Arsenic Tr	14.73	155
RHNO1	83695	Leukemia, MESH:D015470		Doxorubic	14.73	13
RNF130	55819	Leukemia, MESH:D015470		Dexameth	14.73	7
SHPRH	257218	Leukemia, MESH:D015470		Dexameth	14.73	7
SSTR5	6755	Leukemia, MESH:D015470		Decitabine	14.73	12
SUSD6	9766	Leukemia, MESH:D015470		Doxorubic	14.73	12
UTP11	51118	Leukemia, MESH:D015470		Dexameth	14.73	8
YIPF3	25844	Leukemia, MESH:D015470		Dexameth	14.73	11
RAP1GAP	5909	Leukemia, MESH:D01	marker/m	Arsenic|ars	14.72	158
ARHGEF2	9181	Leukemia, MESH:D015473		Arsenic|De	14.72	158
BPTF	2186	Leukemia, MESH:D004915		Doxorubic	14.72	2
BSG	682	Leukemia, MESH:D007948		2-(2-amir	14.72	3
CEP44	80817	Leukemia, MESH:D015473		arsenite|G	14.72	154
COL24A1	255631	Leukemia, MESH:D015470		Dexameth	14.72	12
ECHDC2	55268	Leukemia, MESH:D004915		Doxorubic	14.72	2
EEFSEC	60678	Leukemia, MESH:D015473		Arsenic|Ar	14.72	263
FAF2	23197	Leukemia, MESH:D015473		Arsenic|De	14.72	9
FUBP3	8939	Leukemia, MESH:D015470		Dexameth	14.72	12

GAD2	2572	Leukemia, MESH:D015473	Arsenic|ars	14.72	157
MTCL1	23255	Leukemia, MESH:D015470	Dexameth	14.72	12
PCYT1B	9468	Leukemia, MESH:D015473	Genistein|I	14.72	152
PDE4A	5141	Leukemia, MESH:D015473	Arsenic Tr	14.72	262
PHYH	5264	Leukemia, MESH:D015473	Arsenic|Me	14.72	157
RFC1	5981	Leukemia, MESH:D004915	Doxorubic	14.72	2
RPS6KA5	9252	Leukemia, MESH:D015473	arsenite|Ca	14.72	155
SIM1	6492	Leukemia, MESH:D015473	arsenite|M	14.72	153
TEX9	374618	Leukemia, MESH:D015473	arsenite|M	14.72	153
TPSAB1	7177	Leukemia, MESH:D015473	Genistein|I	14.72	152
UBE4B	10277	Leukemia, MESH:D004915	Doxorubic	14.72	2
WWOX	51741	Leukemia, MESH:D015473	Arsenic|Ar	14.72	263
YWHAH	7533	Leukemia, MESH:D015473	Arsenic Tr	14.72	261
CBLN2	147381	Leukemia, MESH:D015473	Calcitriol|D	14.71	153
CXCR1	3577	Leukemia, MESH:D015470	Air Polluta	14.71	14
HOXD4	3233	Leukemia, MESH:D015473	Arsenic Tr	14.71	260
IL1A	3552	Leukemia, MESH:D007948	2-(2-amin	14.71	3
NOS3	4846	Leukemia, MESH:D007948	2-(2-amin	14.71	3
USP20	10868	Leukemia, MESH:D015473	Arsenic|Ge	14.71	155
ZNF750	79755	Leukemia, MESH:D015473	Arsenic|ars	14.71	10
APOA1	335	Leukemia, MESH:D015473	alpha-Toc	14.7	156
EPB41L4A	114915	Leukemia, MESH:D015473	arsenite|D	14.7	154
KRT1	3848	Leukemia, MESH:D015473	Antimony	14.7	156
NINJ1	4814	Leukemia, MESH:D015473	Arsenic|Ca	14.7	155
PCSK5	5125	Leukemia, MESH:D015473	Arsenic Tr	14.7	262
PNPLA8	50640	Leukemia, MESH:D015470	Deferoxan	14.7	8
RRAGD	58528	Leukemia, MESH:D015473	Arsenic Tr	14.7	159
TYW5	129450	Leukemia, MESH:D015473	Arsenic|De	14.7	155
ACAD9	28976	Leukemia, MESH:D015470	Dexameth	14.69	15
APLN	8862	Leukemia, MESH:D015473	Dexameth	14.69	8
C1ORF198	84886	Leukemia, MESH:D015470	Dasatinib|I	14.69	11
CALB2	794	Leukemia, MESH:D015473	Arsenic|Ar	14.69	167
CAPRIN1	4076	Leukemia, MESH:D015473	arsenite|Re	14.69	160
GALNT3	2591	Leukemia, MESH:D015470	Arsenic Tr	14.69	13
MLEC	9761	Leukemia, MESH:D015470	Dexameth	14.69	7
NCALD	83988	Leukemia, MESH:D015470	Cytarabine	14.69	76
NDUFAB1	4706	Leukemia, MESH:D015470	Arsenic Tr	14.69	19
NECTIN3	25945	Leukemia, MESH:D015470	Arsenic Tr	14.69	20
PCCA	5095	Leukemia, MESH:D015470	Arsenic Tr	14.69	18
PLEKHB1	58473	Leukemia, MESH:D015470	Decitabine	14.69	15
SERPINF2	5345	Leukemia, MESH:D015470	Arsenic Tr	14.69	14
SLBP	7884	Leukemia, MESH:D004915	Doxorubic	14.69	2
SLC4A2	6522	Leukemia, MESH:D015470	Arsenic Tr	14.69	19
SUCLG1	8802	Leukemia, MESH:D015470	Bezafibrat	14.69	14
TNFRSF8	943	Leukemia, MESH:D015470	Arsenic Tr	14.69	13
F11R	50848	Leukemia, MESH:D015473	arsenite|D	14.68	154
GALNT10	55568	Leukemia, MESH:D004915	Cytarabine	14.68	3
GLUD2	2747	Leukemia, MESH:D015473	Calcitriol|D	14.68	155
IMPA2	3613	Leukemia, MESH:D015473	Arsenic|Ar	14.68	264
ITIH1	3697	Leukemia, MESH:D015470	Arsenic Tr	14.68	14
LINC01588	283551	Leukemia, MESH:D015470	(+)-JQ1 co	14.68	9
MAP6	4135	Leukemia, MESH:D015470	Doxorubic	14.68	13
NR0B1	190	Leukemia, MESH:D015473	arsenite|D	14.68	154
SLC25A5	292	Leukemia, MESH:D015473	Arsenic Tr	14.68	156
APOM	55937	Leukemia, MESH:D015473	Arsenic|De	14.67	158
ASPRV1	151516	Leukemia, MESH:D015473	Arsenic|De	14.67	9
DEFA3	1668	Leukemia, MESH:D015470	Arsenic Tr	14.67	11
MAPKBP1	23005	Leukemia, MESH:D015473	arsenite|D	14.67	6

NEU1	4758	Leukemia, MESH:D015473	Arsenic|ar:	14.67	157
RAD23B	5887	Leukemia, MESH:D015473	Arsenic Tr	14.67	261
SHISA3	152573	Leukemia, MESH:D015473	Arsenic|M	14.67	154
SLC9A1	6548	Leukemia, MESH:D007948	2-(2-amin	14.67	3
APOBEC3/	200315	Leukemia, MESH:D015470	Air Polluta	14.66	5
ARHGAP1	9824	Leukemia, MESH:D015473	arsenite|Ca	14.66	7
EBLN2	55096	Leukemia, MESH:D015470	Dexameth	14.66	8
FADS2	9415	Leukemia, MESH:D015473	Arsenic|De	14.66	154
NLGN4X	57502	Leukemia, MESH:D015470	Doxorubic	14.66	11
NUP62CL	54830	Leukemia, MESH:D015473	Arsenic|Ca	14.66	153
RHBDD3	25807	Leukemia, MESH:D015473	Arsenic|so	14.66	158
SCINLB	406363	Leukemia, MESH:D015473	Dexameth	14.66	152
STAT1	6772	Leukemia, MESH:D004915	Doxorubic	14.66	3
TOP3A	7156	Leukemia, MESH:D015470	Air Polluta	14.66	14
ZNF691	51058	Leukemia, MESH:D015470	Doxorubic	14.66	13
C8ORF88	1E+08	Leukemia, MESH:D015470	Gasoline|T	14.65	6
EVPL	2125	Leukemia, MESH:D015473	Arsenic Tr	14.65	156
GIMAP6	474344	Leukemia, MESH:D015470	Air Polluta	14.65	18
RADX	55086	Leukemia, MESH:D015473	Arsenic|Ca	14.65	9
RMND1	55005	Leukemia, MESH:D015470	Arsenic Tr	14.65	13
RRP7A	27341	Leukemia, MESH:D015470	Decitabine	14.65	19
SECTM1	6398	Leukemia, MESH:D015473	Arsenic Tr	14.65	154
TMSB10	9168	Leukemia, MESH:D015473	arsenite|Bu	14.65	157
TTF1	7270	Leukemia, MESH:D015470	Pentachlo	14.65	7
ZNF587B	1E+08	Leukemia, MESH:D015470	Dronabinc	14.65	7
BST2	684	Leukemia, MESH:D007948	Arsenic Tr	14.64	4
CLCC1	23155	Leukemia, MESH:D015470	Arsenic Tr	14.64	19
DNAH2	146754	Leukemia, MESH:D015470	Cytarabine	14.64	74
EFCAB7	84455	Leukemia, MESH:D015470	Calcitriol|D	14.64	11
KATNAL1	84056	Leukemia, MESH:D015470	Bortezomi	14.64	10
MIR126	406913	Leukemia, MESH:D015473	Arsenic|Ar	14.64	159
MKX	283078	Leukemia, MESH:D015470	Irinotecan	14.64	6
NVL	4931	Leukemia, MESH:D015473	Arsenic|M	14.64	11
PIP4P1	90809	Leukemia, MESH:D015470	Arsenic Tr	14.64	18
POGLUT3	143888	Leukemia, MESH:D015470	Dexameth	14.64	8
SOX12	6666	Leukemia, MESH:D015470	Doxorubic	14.64	12
SPATA2L	124044	Leukemia, MESH:D015470	Dronabinc	14.64	7
TAL1	6886	Leukemia, MESH:D015473	Arsenic|Ar	14.64	263
VPS4A	27183	Leukemia, MESH:D015470	Doxorubic	14.64	11
VXN	254778	Leukemia, MESH:D015470	Dronabinc	14.64	8
WDSUB1	151525	Leukemia, MESH:D015470	Calcitriol|D	14.64	14
ADAM33	80332	Leukemia, MESH:D015470	Calcitriol|D	14.63	13
AGAP3	116988	Leukemia, MESH:D015470	Calcitriol|D	14.63	7
ANKRD29	147463	Leukemia, MESH:D015470	Arsenic Tr	14.63	13
ATP5G1	11951	Leukemia, MESH:D015473	Arsenic Tr	14.63	259
COX7B2	170712	Leukemia, MESH:D015470	Calcitriol|D	14.63	10
FBXL16	146330	Leukemia, MESH:D015470	Calcitriol|(	14.63	6
FEZF2	55079	Leukemia, MESH:D015473	Arsenic|ar:	14.63	155
GPC1	2817	Leukemia, MESH:D015473	Arsenic Tr	14.63	156
GUSBP4	375513	Leukemia, MESH:D015470	Dexameth	14.63	5
IMMP1L	196294	Leukemia, MESH:D015470	Dronabinc	14.63	9
IMP4	92856	Leukemia, MESH:D015470	Indometha	14.63	7
LAYN	143903	Leukemia, MESH:D015470	Calcitriol|D	14.63	7
LINC0067:	1E+08	Leukemia, MESH:D015470	Methotrex	14.63	7
MBD5	55777	Leukemia, MESH:D015470	Decitabine	14.63	17
MYO1E	4643	Leukemia, MESH:D015470	Doxorubic	14.63	14
NKX2-2	4821	Leukemia, MESH:D015473	arsenite|D	14.63	155
OSGEP	55644	Leukemia, MESH:D015470	Dexameth	14.63	6

PLK2	10769	Leukemia, MESH:D015473	Dexameth	14.63	153
PRKY	5616	Leukemia, MESH:D015470	Dexameth	14.63	9
PWP1	11137	Leukemia, MESH:D015470	Dexameth	14.63	9
SLC9A6	10479	Leukemia, MESH:D015470	Doxorubic	14.63	13
SSH1	54434	Leukemia, MESH:D015470	Calcitriol|D	14.63	12
ST6GALNA	55808	Leukemia, MESH:D015470	Calcitriol|D	14.63	8
ST8SIA3	51046	Leukemia, MESH:D015470	Dronabinc	14.63	7
SULT1A1	6817	Leukemia, MESH:D015473	Arsenic Tr	14.63	261
TSSC4	10078	Leukemia, MESH:D015470	Benzene|D	14.63	25
ULK4	54986	Leukemia, MESH:D015470	Doxorubic	14.63	12
USP21	27005	Leukemia, MESH:D015470	Doxorubic	14.63	12
AQP10	89872	Leukemia, MESH:D015470	Arsenic Tr	14.62	11
AZU1	566	Leukemia, MESH:D015470	Arsenic Tr	14.62	11
CACNA2D	93589	Leukemia, MESH:D015470	Dexameth	14.62	6
CCNE1	898	Leukemia, MESH:D004915	Daunorub	14.62	3
DAAM1	23002	Leukemia, MESH:D015470	(+)-JQ1 cc	14.62	12
DCAF5	8816	Leukemia, MESH:D015470	Doxorubic	14.62	13
DIPK2A	205428	Leukemia, MESH:D015470	Doxorubic	14.62	13
FBRSL1	57666	Leukemia, MESH:D015473	Arsenic|ars	14.62	156
FUT6	2528	Leukemia, MESH:D015470	Arsenic Tr	14.62	12
GAB1	2549	Leukemia, MESH:D015470	Calcitriol|D	14.62	16
GOLM2	113201	Leukemia, MESH:D015470	Doxorubic	14.62	12
HDGFL3	50810	Leukemia, MESH:D015470	Methotrex	14.62	9
KCTD17	79734	Leukemia, MESH:D015470	Dexameth	14.62	11
KIAA2026	158358	Leukemia, MESH:D015470	Dexameth	14.62	10
KNOP1	400506	Leukemia, MESH:D015470	Dexameth	14.62	6
LRRC34	151827	Leukemia, MESH:D015470	(+)-JQ1 cc	14.62	7
PCDHGA2	56113	Leukemia, MESH:D015470	Dexameth	14.62	10
PRPS1	5631	Leukemia, MESH:D015470	Arsenic Tr	14.62	18
SHTN1	57698	Leukemia, MESH:D015470	Arsenic Tr	14.62	18
SKAP2	8935	Leukemia, MESH:D015470	Doxorubic	14.62	14
SLC9B2	133308	Leukemia, MESH:D015470	Doxorubic	14.62	12
SVEP1	79987	Leukemia, MESH:D004915	Cytarabine	14.62	2
TCF3	6929	Leukemia, MESH:D015470	Decitabine	14.62	19
TOR4A	54863	Leukemia, MESH:D015470	Dexameth	14.62	6
VAT1	10493	Leukemia, MESH:D015473	Arsenic|De	14.62	156
ZC3H7A	29066	Leukemia, MESH:D015470	Doxorubic	14.62	11
ZNF704	619279	Leukemia, MESH:D015470	Doxorubic	14.62	11
ADAMTS3	9508	Leukemia, MESH:D015470	Doxorubic	14.61	12
ANKRD45	339416	Leukemia, MESH:D015470	Indometha	14.61	6
CYGB	114757	Leukemia, MESH:D007948	Arsenic Tr	14.61	3
EPM2A	7957	Leukemia, MESH:D015473	Dexameth	14.61	153
EPS8L2	64787	Leukemia, MESH:D015470	Doxorubic	14.61	13
FOS	2353	Leukemia, MESH:D004915	Doxorubic	14.61	4
GLG1	2734	Leukemia, MESH:D004915	Doxorubic	14.61	2
PLCB4	5332	Leukemia, MESH:D015473	Arsenic Tr	14.61	160
RASGRP2	10235	Leukemia, MESH:D004915	Doxorubic	14.61	2
TGFB1	7040	Leukemia, MESH:D004915	Daunorub	14.61	4
TRAPPC14	55262	Leukemia, MESH:D015470	Doxorubic	14.61	10
TRPV4	59341	Leukemia, MESH:D015473	Buthionine	14.61	154
ZNF219	51222	Leukemia, MESH:D015473	arsenite|D	14.61	154
CELF2	10659	Leukemia, MESH:D015473	arsenite|D	14.6	156
CHMP5	51510	Leukemia, MESH:D015473	arsenic dis	14.6	154
FGL2	10875	Leukemia, MESH:D015473	Arsenic|Ar	14.6	261
KLHDC4	54758	Leukemia, MESH:D015473	Arsenic|De	14.6	155
ATP5F1C	509	Leukemia, MESH:D015473	Arsenic Tr	14.59	156
CLDN5	7122	Leukemia, MESH:D007948	2-(2-amin	14.59	2
ECM1	1893	Leukemia, MESH:D015473	Arsenic Tr	14.59	262

IQGAP2	10788	Leukemia, MESH:D015473	Genistein|I	14.59	155
NPY1R	4886	Leukemia, MESH:D015473	arsenite|D	14.59	155
TALDO1	6888	Leukemia, MESH:D007948	Arsenic Tr	14.59	3
PRKAR1A	5573	Leukemia, MESH:D01 marker/m	Arsenic Tr	14.58	261
ARL6IP5	10550	Leukemia, MESH:D004915	Cytarabine	14.58	2
ASL	435	Leukemia, MESH:D015473	Arsenic Tr	14.58	260
CES1	1066	Leukemia, MESH:D015470	Benzene|D	14.58	31
CSK	1445	Leukemia, MESH:D015473	caffeic aci	14.58	151
GPT	2875	Leukemia, MESH:D007948	2-(2-amin	14.58	3
HEPH	9843	Leukemia, MESH:D015470	Dasatinib|I	14.58	24
NME2	4831	Leukemia, MESH:D015473	arsenite|C	14.58	155
PHACTR3	116154	Leukemia, MESH:D015473	Dexameth	14.58	5
PRPH	5630	Leukemia, MESH:D004915	Cytarabine	14.58	2
CASR	846	Leukemia, MESH:D015470	Calcitriol|C	14.57	9
GTF2IRD1	9569	Leukemia, MESH:D004915	Doxorubic	14.57	2
PRELP	5549	Leukemia, MESH:D004915	Doxorubic	14.57	2
SMPD4	55627	Leukemia, MESH:D015473	Arsenic|ca	14.57	154
TWIST1	7291	Leukemia, MESH:D007948	pyrazolant	14.57	2
ZAP70	7535	Leukemia, MESH:D015470	Cyclophos	14.57	7
DECR2	26063	Leukemia, MESH:D015470	Dexameth	14.56	8
FNDC5	252995	Leukemia, MESH:D015470	Air Polluta	14.56	19
MYL4	4635	Leukemia, MESH:D015470	Arsenic Tr	14.56	85
NEBL	10529	Leukemia, MESH:D015470	Doxorubic	14.56	14
PCP4L1	654790	Leukemia, MESH:D015470	Dronabinc	14.56	7
PRPS2	5634	Leukemia, MESH:D015470	Arsenic Tr	14.56	17
ST3GAL1	6482	Leukemia, MESH:D015473	Arsenic|Cy	14.56	158
TNFSF4	7292	Leukemia, MESH:D015470	Dexameth	14.56	12
APRT	353	Leukemia, MESH:D015470	Dexameth	14.55	13
BCORL1	63035	Leukemia, MESH:D015470	Dronabinc	14.55	10
C1QBP	708	Leukemia, MESH:D015473	Dexameth	14.55	152
CHRNA4	1137	Leukemia, MESH:D015473	antimony	14.55	157
COG8	84342	Leukemia, MESH:D015470	Decitabine	14.55	19
CYP1A2	1544	Leukemia, MESH:D004915	Daunorub	14.55	3
ESCO2	157570	Leukemia, MESH:D015473	arsenite|C	14.55	155
ETV6	2120	Leukemia, MESH:D004915	Doxorubic	14.55	2
LTA	4049	Leukemia, MESH:D015473	Arsenic|Ar	14.55	161
NKAIN1	79570	Leukemia, MESH:D015470	Cyclophos	14.55	12
PPARG	5468	Leukemia, MESH:D004915	Cytarabine	14.55	3
PSMD14	10213	Leukemia, MESH:D015470	Dexameth	14.55	10
SOAT1	6646	Leukemia, MESH:D015473	Arsenic|ca	14.55	155
STARD13	90627	Leukemia, MESH:D015470	Dexameth	14.55	12
TENM3	55714	Leukemia, MESH:D015470	Calcitriol|D	14.55	13
TNNC2	7125	Leukemia, MESH:D015470	Dexameth	14.55	7
UAP1L1	91373	Leukemia, MESH:D015470	Arsenic Tr	14.55	14
YY1	7528	Leukemia, MESH:D015470	Arsenic Tr	14.55	14
ABCC3	8714	Leukemia, MESH:D004915	Doxorubic	14.54	3
ADCYAP1	116	Leukemia, MESH:D015473	Arsenic|ars	14.54	11
BRF2	55290	Leukemia, MESH:D015470	Bortezomi	14.54	14
CASQ1	844	Leukemia, MESH:D015470	Doxorubic	14.54	11
CNOT3	4849	Leukemia, MESH:D015470	Methotrex	14.54	11
DHRS4	10901	Leukemia, MESH:D015473	Arsenic|Ge	14.54	157
EIF4EBP2	1979	Leukemia, MESH:D004915	Doxorubic	14.54	2
FER1L4	80307	Leukemia, MESH:D015473	arsenite|D	14.54	153
GPRIN1	114787	Leukemia, MESH:D015473	Arsenic|Ge	14.54	10
LMX1A	4009	Leukemia, MESH:D015473	Arsenic Tr	14.54	155
LRP5	4041	Leukemia, MESH:D015473	Arsenic|Ca	14.54	158
LTBP4	8425	Leukemia, MESH:D004915	Doxorubic	14.54	2
MEAF6	64769	Leukemia, MESH:D015473	arsenite|D	14.54	155

MIR20A	406982	Leukemia, MESH:D015470	Calcitriol|[	14.54	7
MT1L	4500	Leukemia, MESH:D015470	Arsenic Tr	14.54	18
TEF	7008	Leukemia, MESH:D015473	arsenite|D	14.54	154
TRAF6	7189	Leukemia, MESH:D007948	Arsenic Tr	14.54	3
ZFP459	328274	Leukemia, MESH:D015470	Cyclophos	14.54	7
LYL1	4066	Leukemia, MESH:D01 marker/m	Cytarabine	14.53	76
ACSM3	6296	Leukemia, MESH:D015473	Dexameth	14.53	155
AICDA	57379	Leukemia, MESH:D015473	Arsenic|Ar	14.53	263
ARL6IP4	51329	Leukemia, MESH:D015470	Dexameth	14.53	8
COPS3	8533	Leukemia, MESH:D015470	Doxorubic	14.53	13
CTR9	9646	Leukemia, MESH:D015470	Air Polluta	14.53	8
DZIP1	22873	Leukemia, MESH:D015470	Cyclophos	14.53	11
ENHO	375704	Leukemia, MESH:D015470	Dronabinc	14.53	7
FAM149B1	317662	Leukemia, MESH:D015473	Arsenic|De	14.53	11
FOXJ3	22887	Leukemia, MESH:D015470	Doxorubic	14.53	15
GALNT5	11227	Leukemia, MESH:D015473	Arsenic|De	14.53	9
GIMAP8	155038	Leukemia, MESH:D015470	Air Polluta	14.53	11
HCST	10870	Leukemia, MESH:D015470	Arsenic Tr	14.53	12
HOXD3	3232	Leukemia, MESH:D015470	Decitabine	14.53	14
HSPB3	8988	Leukemia, MESH:D015470	Calcitriol|[	14.53	12
IFITM2	10581	Leukemia, MESH:D015473	Arsenic|De	14.53	155
LIN28A	79727	Leukemia, MESH:D015470	Decitabine	14.53	14
LOXL3	84695	Leukemia, MESH:D015470	Dexameth	14.53	7
NUCB2	4925	Leukemia, MESH:D007948	2-(2-amin	14.53	3
POLDIP2	26073	Leukemia, MESH:D015473	Arsenic Tr	14.53	261
SLC10A4	201780	Leukemia, MESH:D015470	(+)-JQ1 cc	14.53	7
SLITRK6	84189	Leukemia, MESH:D015470	Air Polluta	14.53	7
USP45	85015	Leukemia, MESH:D015470	Bortezomi	14.53	13
WBP11	51729	Leukemia, MESH:D015470	Dexameth	14.53	8
WDR35	57539	Leukemia, MESH:D015470	(+)-JQ1 cc	14.53	7
ZFP42	132625	Leukemia, MESH:D015473	Arsenic Tr	14.53	262
ZNF277	11179	Leukemia, MESH:D015470	Indometha	14.53	7
ADPRM	56985	Leukemia, MESH:D015473	Arsenic|ars	14.52	10
CAPN7	23473	Leukemia, MESH:D015470	Calcitriol|[	14.52	11
CCDC88B	283234	Leukemia, MESH:D015470	(+)-JQ1 cc	14.52	9
CD248	57124	Leukemia, MESH:D015470	Doxorubic	14.52	13
DIPK1A	388650	Leukemia, MESH:D015470	Doxorubic	14.52	12
FAHD2A	51011	Leukemia, MESH:D015470	Arsenic Tr	14.52	12
FSHB	2488	Leukemia, MESH:D015473	2-(2-chlor	14.52	156
HLA-DQA	3117	Leukemia, MESH:D015473	Arsenic|Ca	14.52	8
LAMTOR5	10542	Leukemia, MESH:D015470	Arsenic Tr	14.52	13
LYPD6B	130576	Leukemia, MESH:D015473	Arsenic|Ge	14.52	155
MRPL15	29088	Leukemia, MESH:D015470	(+)-JQ1 cc	14.52	9
MRPS2	51116	Leukemia, MESH:D015470	Pentachlo	14.52	9
NMRK2	27231	Leukemia, MESH:D015470	Dexameth	14.52	11
NOSIP	51070	Leukemia, MESH:D015470	Doxorubic	14.52	11
PSMF1	9491	Leukemia, MESH:D015473	Calcitriol|[	14.52	153
RAD54L2	23132	Leukemia, MESH:D015470	Dexameth	14.52	8
RBM18	92400	Leukemia, MESH:D015470	Doxorubic	14.52	14
RHOT1	55288	Leukemia, MESH:D015470	Arsenic Tr	14.52	19
SERGEF	26297	Leukemia, MESH:D015470	Arsenic Tr	14.52	12
SMUG1	23583	Leukemia, MESH:D015470	Decitabine	14.52	19
TBL2	26608	Leukemia, MESH:D015470	Dronabinc	14.52	7
UBL7	84993	Leukemia, MESH:D015470	Dexameth	14.52	7
UPK1B	7348	Leukemia, MESH:D015470	Cyclophos	14.52	11
UPRT	139596	Leukemia, MESH:D015470	Calcitriol|[	14.52	12
ABRACL	58527	Leukemia, MESH:D015473	arsenite|D	14.51	154
FKRP	79147	Leukemia, MESH:D015470	Doxorubic	14.51	12

GABARAP	23766	Leukemia, MESH:D015473	Arsenic|Cy	14.51	155
HLA-B	3106	Leukemia, MESH:D015473	Arsenic Tr	14.51	261
HOMEZ	57594	Leukemia, MESH:D015470	Dexameth	14.51	6
KIF20B	9585	Leukemia, MESH:D015473	arsenite|Ca	14.51	154
MAB21L1	4081	Leukemia, MESH:D015473	arsenite|D	14.51	154
MX2	4600	Leukemia, MESH:D015473	Dexameth	14.51	156
PDE6B	5158	Leukemia, MESH:D015473	Arsenic|De	14.51	155
POLQ	10721	Leukemia, MESH:D004915	Doxorubic	14.51	2
PPIC	5480	Leukemia, MESH:D004915	Cytarabine	14.51	3
TLNRD1	59274	Leukemia, MESH:D015470	Dexameth	14.51	11
TNNI1	7135	Leukemia, MESH:D015470	Dexameth	14.51	11
TULP3	7289	Leukemia, MESH:D015470	Dexameth	14.51	7
AKT1S1	84335	Leukemia, MESH:D015470	Chloroqui	14.5	9
CLEC7A	64581	Leukemia, MESH:D015470	Air Polluta	14.5	16
COL6A3	1293	Leukemia, MESH:D007948	Arsenic Tr	14.5	4
DNASE1L3	1776	Leukemia, MESH:D015470	Dexameth	14.5	11
E2F3	1871	Leukemia, MESH:D015473	Arsenic|Ar	14.5	160
EIF3C	8663	Leukemia, MESH:D015470	Arsenic Tr	14.5	20
FERMT2	10979	Leukemia, MESH:D015473	Arsenic Tr	14.5	261
HIGD1A	25994	Leukemia, MESH:D015473	Arsenic|Ar	14.5	159
IKZF1	10320	Leukemia, MESH:D015470	Bortezomi	14.5	9
MIR501	574503	Leukemia, MESH:D015470	Air Polluta	14.5	20
NCOA7	135112	Leukemia, MESH:D015473	Arsenic Tr	14.5	262
RECK	8434	Leukemia, MESH:D015473	Dasatinib|	14.5	155
SERINC3	10955	Leukemia, MESH:D004915	Doxorubic	14.5	2
ADCY9	115	Leukemia, MESH:D015470	Arsenic Tr	14.49	17
ALOX5AP	241	Leukemia, MESH:D007948	Arsenic Tr	14.49	3
CAVIN2	8436	Leukemia, MESH:D015473	Arsenic Tr	14.49	156
CDH5	1003	Leukemia, MESH:D015470	Arsenic Tr	14.49	20
EHD4	30844	Leukemia, MESH:D015470	Calcitriol|D	14.49	10
NFYB	4801	Leukemia, MESH:D015470	Dexameth	14.49	8
PFKFB1	5207	Leukemia, MESH:D015470	Benzene|D	14.49	28
PLXNA1	5361	Leukemia, MESH:D015473	arsenite|D	14.49	156
QPCT	25797	Leukemia, MESH:D015470	Decitabine	14.49	21
RPL5	6125	Leukemia, MESH:D015470	Dronabinc	14.49	11
ATP5MEA	559659	Leukemia, MESH:D015470	Dronabinc	14.48	3
HLA-J	3137	Leukemia, MESH:D015473	sodium ar	14.48	155
MUC16	94025	Leukemia, MESH:D015473	Genistein|	14.48	152
PRL2C3	18812	Leukemia, MESH:D015473	alpha-Toc	14.48	153
GOT1	2805	Leukemia, MESH:D015473	Arsenic Tr	14.47	156
GRIN1	2902	Leukemia, MESH:D015470	Arsenic Tr	14.47	31
AGTR1A	11607	Leukemia, MESH:D015470	Daunorub	14.46	36
B3GNT3	10331	Leukemia, MESH:D015473	Arsenic Tr	14.46	154
CDC42SE2	56990	Leukemia, MESH:D015470	Benzoates	14.46	8
DGUOK	1716	Leukemia, MESH:D015473	arsenite|G	14.46	154
GAS1	2619	Leukemia, MESH:D015473	Arsenic|Ar	14.46	263
HNRNPK	3190	Leukemia, MESH:D007948	Arsenic Tr	14.46	3
HOXC6	3223	Leukemia, MESH:D015470	Cytarabine	14.46	77
HS3ST3A1	9955	Leukemia, MESH:D015473	Calcitriol|D	14.46	152
PFKMB	568001	Leukemia, MESH:D015470	Alitretinoi	14.46	6
PLEKHG6	55200	Leukemia, MESH:D015473	Arsenic|Ar	14.46	159
PRL	5617	Leukemia, MESH:D015473	Arsenic|Cy	14.46	22
RARG	5916	Leukemia, MESH:D015473	Anthracyc	14.46	264
SLC16A2	6567	Leukemia, MESH:D015473	arsenite|D	14.46	154
SSX8P	280659	Leukemia, MESH:D015470	Arsenic Tr	14.46	18
VPS25	84313	Leukemia, MESH:D015473	Dexameth	14.46	8
WDFY2	115825	Leukemia, MESH:D015473	Arsenic|Ar	14.46	159
ABCB11A	797269	Leukemia, MESH:D015470	Dexameth	14.45	3

ADIPOQ	9370	Leukemia, MESH:D004915	Cytarabine	14.45	3
ALDH6A1	4329	Leukemia, MESH:D015470	Arsenic Tr	14.45	26
APLNR	187	Leukemia, MESH:D004915	Cytarabine	14.45	2
C1ORF109	54955	Leukemia, MESH:D015470	Bortezomi	14.45	6
CAMK4	814	Leukemia, MESH:D015473	Arsenic Tr	14.45	156
CLDN9	9080	Leukemia, MESH:D015473	Arsenic Tr	14.45	154
CRISPLD1	83690	Leukemia, MESH:D015473	Calcitriol|G	14.45	153
DOK1	1796	Leukemia, MESH:D015470	Decitabine	14.45	19
GALE	2582	Leukemia, MESH:D015473	Arsenic Tr	14.45	260
HLA-DPB1	3115	Leukemia, MESH:D015470	Benzene|B	14.45	23
MRPL30	51263	Leukemia, MESH:D015470	Arsenic Tr	14.45	16
NOXA1	10811	Leukemia, MESH:D015470	Dexameth	14.45	6
PSMD10	5716	Leukemia, MESH:D015473	arsenite|G	14.45	7
VCP	7415	Leukemia, MESH:D015470	Arsenic Tr	14.45	15
VSIG4	11326	Leukemia, MESH:D015473	Arsenic Tr	14.45	261
ANKRD12	23253	Leukemia, MESH:D015473	Arsenic Tr	14.44	262
BIN1	274	Leukemia, MESH:D004915	Doxorubic	14.44	3
ELAVL4	1996	Leukemia, MESH:D015470	Dronabino	14.44	16
EXOSC7	23016	Leukemia, MESH:D015473	Genistein|I	14.44	154
GUSBP1	728411	Leukemia, MESH:D015470	Cytarabine	14.44	73
IL4	3565	Leukemia, MESH:D007948	2-(2-amin	14.44	3
INO80C	125476	Leukemia, MESH:D015470	Dexameth	14.44	6
MCTS1	28985	Leukemia, MESH:D015470	Arsenic Tr	14.44	33
PEX11B	8799	Leukemia, MESH:D015470	Air Polluta	14.44	13
POMT1	10585	Leukemia, MESH:D015470	Arsenic Tr	14.44	13
SLC39A4	55630	Leukemia, MESH:D015470	Calcitriol|D	14.44	8
STAP1	26228	Leukemia, MESH:D015473	Arsenic Tr	14.44	260
SWT1	54823	Leukemia, MESH:D015470	Decitabine	14.44	16
TINAG	27283	Leukemia, MESH:D015470	Calcitriol|D	14.44	9
TM2D1	83941	Leukemia, MESH:D015470	Decitabine	14.44	16
TOMM20A	406309	Leukemia, MESH:D015470	Dexameth	14.44	3
VSX1	30813	Leukemia, MESH:D015470	Alitretinoi	14.44	7
ZNF543	125919	Leukemia, MESH:D015470	Dexameth	14.44	6
AADAT	51166	Leukemia, MESH:D015470	Dexameth	14.43	12
ALKBH6	84964	Leukemia, MESH:D015470	Doxorubic	14.43	10
C8B	732	Leukemia, MESH:D015470	Dexameth	14.43	10
CASK	8573	Leukemia, MESH:D015470	Arsenic Tr	14.43	14
CDK5RAP1	51654	Leukemia, MESH:D015470	Doxorubic	14.43	12
CUTA	51596	Leukemia, MESH:D015473	Arsenic|Ge	14.43	155
DAZAP1	26528	Leukemia, MESH:D015470	Air Polluta	14.43	18
DEFA4	1669	Leukemia, MESH:D015473	Arsenic Tr	14.43	258
EIF2AK2	5610	Leukemia, MESH:D007948	Arsenic Tr	14.43	3
ELP6	54859	Leukemia, MESH:D015470	Arsenic Tr	14.43	13
FABP7	2173	Leukemia, MESH:D007948	Arsenic Tr	14.43	4
FBXO4	26272	Leukemia, MESH:D015470	Calcitriol|D	14.43	5
HAS2	3037	Leukemia, MESH:D007948	Arsenic Tr	14.43	3
IGFBP2A	794176	Leukemia, MESH:D015470	Dexameth	14.43	5
KTN1	3895	Leukemia, MESH:D004915	Doxorubic	14.43	2
LCMT2	9836	Leukemia, MESH:D015470	Decitabine	14.43	18
LTK	4058	Leukemia, MESH:D015473	Arsenic|ars	14.43	156
MAP1S	55201	Leukemia, MESH:D015470	Benzene|T	14.43	21
MIR15B	406949	Leukemia, MESH:D015470	Arsenic Tr	14.43	25
MRPL40	64976	Leukemia, MESH:D015470	Dexameth	14.43	12
MZF1	7593	Leukemia, MESH:D015470	Calcitriol|D	14.43	6
NPAS1	4861	Leukemia, MESH:D015470	Arsenic Tr	14.43	20
PTPRT	11122	Leukemia, MESH:D015473	arsenite|G	14.43	154
SEC22A	26984	Leukemia, MESH:D015470	Decitabine	14.43	12
SPTLC1	10558	Leukemia, MESH:D015470	Arsenic Tr	14.43	13

TCF15	6939	Leukemia, MESH:D015470	Dexameth	14.43	7
TMEM79	84283	Leukemia, MESH:D015470	Calcitriol|C	14.43	6
ZC3H12A	80149	Leukemia, MESH:D015470	Air Polluta	14.43	16
ZNF415	55786	Leukemia, MESH:D015470	Decitabine	14.43	16
ALDH8A1	64577	Leukemia, MESH:D015470	Arsenic Tr	14.42	13
APOOL	139322	Leukemia, MESH:D015470	Bortezomi	14.42	12
CASKIN2	57513	Leukemia, MESH:D015470	Arsenic Tr	14.42	17
CBARP	255057	Leukemia, MESH:D015470	Dexameth	14.42	6
COG1	9382	Leukemia, MESH:D015470	Doxorubic	14.42	11
COMMD6	170622	Leukemia, MESH:D015473	arsenite|D	14.42	154
DCK	1633	Leukemia, MESH:D015473	arsenite|Cy	14.42	159
DDR2	4921	Leukemia, MESH:D015473	Arsenic Tr	14.42	264
ELAPOR1	57535	Leukemia, MESH:D015470	Calcitriol|F	14.42	9
ERVMER34	1E+08	Leukemia, MESH:D015470	Arsenic Tr	14.42	12
FGFR2	2263	Leukemia, MESH:D015473	Arsenic Tr	14.42	261
IPO11	51194	Leukemia, MESH:D015470	Calcitriol|N	14.42	8
KATNBL1	79768	Leukemia, MESH:D015470	Dronabinc	14.42	6
LYZ	4069	Leukemia, MESH:D015473	Calcitriol|C	14.42	158
MINDY3	80013	Leukemia, MESH:D015470	Dexameth	14.42	7
MIR106A	406899	Leukemia, MESH:D015470	Dexameth	14.42	9
NOMO1	23420	Leukemia, MESH:D015470	Dexameth	14.42	5
NSG2	51617	Leukemia, MESH:D015470	Doxorubic	14.42	12
NUBP1	4682	Leukemia, MESH:D015470	Arsenic Tr	14.42	13
PDE7A	5150	Leukemia, MESH:D015470	Bortezomi	14.42	14
PDYN	5173	Leukemia, MESH:D007948	2-(2-amin	14.42	2
PGBD3	267004	Leukemia, MESH:D015470	Dexameth	14.42	5
PHYHIPL	84457	Leukemia, MESH:D015473	Arsenic|Ar	14.42	165
PHYKPL	85007	Leukemia, MESH:D015470	Calcitriol|D	14.42	12
PKD1L2	114780	Leukemia, MESH:D015470	Decitabine	14.42	13
PRKD3	23683	Leukemia, MESH:D015470	Arsenic Tr	14.42	19
PYCARD	29108	Leukemia, MESH:D015473	Arsenic Tr	14.42	261
SUSD1	64420	Leukemia, MESH:D015470	Dexameth	14.42	12
TBKBP1	9755	Leukemia, MESH:D015470	(+)-JQ1 cc	14.42	9
VPS8	23355	Leukemia, MESH:D015470	Dexameth	14.42	6
YTHDF3	253943	Leukemia, MESH:D015470	Indometha	14.42	10
ZW10	9183	Leukemia, MESH:D015473	Arsenic Tr	14.42	260
ABHD17B	51104	Leukemia, MESH:D015470	Dexameth	14.41	11
ANKRD18A	253650	Leukemia, MESH:D015470	Resveratrc	14.41	7
APOO	79135	Leukemia, MESH:D015470	Dexameth	14.41	7
ATP6AP1L	92270	Leukemia, MESH:D015470	Dexameth	14.41	6
BACE2	25825	Leukemia, MESH:D004915	Doxorubic	14.41	2
C2ORF76	130355	Leukemia, MESH:D015470	Dexameth	14.41	10
C3ORF62	375341	Leukemia, MESH:D015470	Decitabine	14.41	17
CFI	3426	Leukemia, MESH:D015473	Arsenic Tr	14.41	261
CNTN3	5067	Leukemia, MESH:D015470	Arsenic Tr	14.41	13
CTNND1	1500	Leukemia, MESH:D015473	Arsenic Tr	14.41	261
DPM1	8813	Leukemia, MESH:D015470	Doxorubic	14.41	12
ENDOG	2021	Leukemia, MESH:D015473	Arsenic|De	14.41	12
GPR161	23432	Leukemia, MESH:D015470	Doxorubic	14.41	11
ISLR2	57611	Leukemia, MESH:D015470	Doxorubic	14.41	12
KLHDC7A	127707	Leukemia, MESH:D015470	Dronabinc	14.41	7
LSM14A	26065	Leukemia, MESH:D015470	Dexameth	14.41	12
MIGA1	374986	Leukemia, MESH:D015470	Dexameth	14.41	6
MPDU1	9526	Leukemia, MESH:D015470	Arsenic Tr	14.41	17
MRAP2	112609	Leukemia, MESH:D015470	Arsenic Tr	14.41	12
MRPS14	63931	Leukemia, MESH:D015470	Dexameth	14.41	6
ORC2	4999	Leukemia, MESH:D015470	Dexameth	14.41	12
PALLD	23022	Leukemia, MESH:D015473	Calcitriol|D	14.41	153

PCDHGA9	56107	Leukemia, MESH:D015470	Doxorubic	14.41	12
POPDC3	64208	Leukemia, MESH:D015470	Doxorubic	14.41	12
PWWP2B	170394	Leukemia, MESH:D015470	Doxorubic	14.41	12
RBAK	57786	Leukemia, MESH:D015470	Doxorubic	14.41	13
RRP1	8568	Leukemia, MESH:D015470	Resveratrc	14.41	9
SLC25A37	51312	Leukemia, MESH:D015473	Arsenic Tr	14.41	262
SNRNP35	11066	Leukemia, MESH:D015470	Doxorubic	14.41	11
STK32A	202374	Leukemia, MESH:D015470	Dexameth	14.41	7
SZRD1	26099	Leukemia, MESH:D015470	Dexameth	14.41	7
TCAIM	285343	Leukemia, MESH:D015470	Doxorubic	14.41	12
TRAPPC1	58485	Leukemia, MESH:D015470	Dexameth	14.41	11
USP8	9101	Leukemia, MESH:D015470	Dexameth	14.41	11
ZDHHC8P	150244	Leukemia, MESH:D015470	(+)-JQ1 cc	14.41	6
APBB2	323	Leukemia, MESH:D004915	Doxorubic	14.4	2
COL1A1B	325675	Leukemia, MESH:D015470	Dexameth	14.4	6
EWSR1	2130	Leukemia, MESH:D004915	Doxorubic	14.4	2
FAM104B	90736	Leukemia, MESH:D015470	Arsenic Tr	14.4	17
MIRLET7B	406884	Leukemia, MESH:D015473	arsenite|M	14.4	153
PEMT	10400	Leukemia, MESH:D004915	Doxorubic	14.4	2
PPRC1	23082	Leukemia, MESH:D015473	Arsenic Tr	14.4	260
SNHG9	735301	Leukemia, MESH:D015470	Doxorubic	14.4	10
TAP2	6891	Leukemia, MESH:D004915	Daunorub	14.4	2
UGT2B15	7366	Leukemia, MESH:D015470	Calcitriol|D	14.4	11
YY1AP1	55249	Leukemia, MESH:D015470	Doxorubic	14.4	10
ZFAND1	79752	Leukemia, MESH:D015473	arsenite|M	14.4	155
ADGRE3	84658	Leukemia, MESH:D015473	Calcitriol|s	14.39	152
AIP-1	179898	Leukemia, MESH:D015473	Arsenic|ars	14.39	9
ARMH3	79591	Leukemia, MESH:D015470	Doxorubic	14.39	10
HTR2A	3356	Leukemia, MESH:D015473	Arsenic|ars	14.39	13
MST1L	11223	Leukemia, MESH:D015470	Tobacco S	14.39	6
TM4SF20	79853	Leukemia, MESH:D015473	Daunorub	14.39	15
VSTM4	196740	Leukemia, MESH:D015473	Arsenic|Ca	14.39	8
ZNF665	79788	Leukemia, MESH:D015473	Arsenic|ars	14.39	9
ANGPTL2	23452	Leukemia, MESH:D004915	Cytarabine	14.38	2
DEAF1	10522	Leukemia, MESH:D015473	caffeic aci	14.38	152
EIF4B	1975	Leukemia, MESH:D015473	Arsenic Tr	14.38	265
EPHB2	2048	Leukemia, MESH:D015470	Bortezomi	14.38	9
INSM1B	323882	Leukemia, MESH:D015470	Pentachlo	14.38	4
NELL2	4753	Leukemia, MESH:D004915	Cytarabine	14.38	2
PDCD7	10081	Leukemia, MESH:D015473	Dexameth	14.38	5
PGAM1B	327165	Leukemia, MESH:D015470	Pentachlo	14.38	4
RUNDC3A	393118	Leukemia, MESH:D015470	Pentachlo	14.38	4
SIRT2	22933	Leukemia, MESH:D015470	Methotrex	14.38	11
XRCC3	7517	Leukemia, MESH:D004915	Doxorubic	14.38	2
ZNF253	56242	Leukemia, MESH:D015470	Doxorubic	14.38	11
ZNF649	65251	Leukemia, MESH:D015470	Doxorubic	14.38	11
ANKDD1A	348094	Leukemia, MESH:D015473	arsenite|sc	14.37	154
CCT8	10694	Leukemia, MESH:D015470	Arsenic Tr	14.37	26
CD8A	925	Leukemia, MESH:D015470	Cyclophos	14.37	78
CLDN2	9075	Leukemia, MESH:D015473	alpha-Toc	14.37	6
EGLN2	112398	Leukemia, MESH:D015470	Benzene|C	14.37	20
FBL	2091	Leukemia, MESH:D015470	Doxorubic	14.37	21
KCTD6	200845	Leukemia, MESH:D015473	Arsenic|Ar	14.37	263
LCT	3938	Leukemia, MESH:D015470	Decitabine	14.37	13
LYZ2	17105	Leukemia, MESH:D015470	Benzene|D	14.37	26
PDLIM2	64236	Leukemia, MESH:D015470	Calcitriol|D	14.37	11
PEA15	8682	Leukemia, MESH:D004915	Doxorubic	14.37	2
PI16	221476	Leukemia, MESH:D015470	Dexameth	14.37	14

RBMS1	5937	Leukemia, MESH:D004915	Doxorubic	14.37	2
SNHG7	84973	Leukemia, MESH:D015473	Dexameth	14.37	8
ZNF157	7712	Leukemia, MESH:D015473	arsenite|D	14.37	5
SMO	6608	Leukemia, MESH:D01 marker/m	Arsenic Tr	14.36	155
ANKRD19	138649	Leukemia, MESH:D015473	arsenite|sc	14.36	153
ASPA	443	Leukemia, MESH:D015470	Dexameth	14.36	8
BFAR	51283	Leukemia, MESH:D015473	Arsenic|Ar	14.36	160
CCT2	10576	Leukemia, MESH:D015473	Arsenic Tr	14.36	263
CD200	4345	Leukemia, MESH:D015470	Doxorubic	14.36	16
CHCHD10	400916	Leukemia, MESH:D015470	Dexameth	14.36	9
GFPT1	2673	Leukemia, MESH:D015470	Doxorubic	14.36	13
HCAR3	8843	Leukemia, MESH:D015470	Calcitriol|I	14.36	8
HEATR6	63897	Leukemia, MESH:D015473	Arsenic Tr	14.36	155
JKAMP	51528	Leukemia, MESH:D015473	Arsenic Tr	14.36	260
KCNN2	3781	Leukemia, MESH:D004915	Cytarabine	14.36	2
LRRC47	57470	Leukemia, MESH:D015470	Doxorubic	14.36	21
LYRM1	57149	Leukemia, MESH:D015473	Arsenic|ars	14.36	10
MGST3B	567275	Leukemia, MESH:D015473	Arsenic|De	14.36	8
NEDD4	4734	Leukemia, MESH:D015473	Arsenic|ars	14.36	159
NUP133	55746	Leukemia, MESH:D015470	Air Polluta	14.36	13
PDXK	8566	Leukemia, MESH:D015473	Arsenic Tr	14.36	260
PNRC1	10957	Leukemia, MESH:D015473	Arsenic Tr	14.36	261
PRKCBB	393953	Leukemia, MESH:D015473	Genistein|	14.36	152
PRKD1	5587	Leukemia, MESH:D015470	Calcitriol|D	14.36	14
PRSS8	5652	Leukemia, MESH:D015473	Arsenic|Ge	14.36	157
RASSF4	83937	Leukemia, MESH:D015473	Dexameth	14.36	156
RETN	56729	Leukemia, MESH:D007948	Arsenic Tr	14.36	3
SMCR8	140775	Leukemia, MESH:D015473	Arsenic|De	14.36	153
SORL1	6653	Leukemia, MESH:D015470	Benzene|D	14.36	28
TAF1D	79101	Leukemia, MESH:D015470	Arsenic Tr	14.36	16
TMEM245	23731	Leukemia, MESH:D015473	Arsenic Tr	14.36	258
TMEM69	51249	Leukemia, MESH:D015470	Air Polluta	14.36	12
TSPYL1	7259	Leukemia, MESH:D015473	Dexameth	14.36	153
UNC5D	137970	Leukemia, MESH:D015473	Arsenic Tr	14.36	260
YEATS2	55689	Leukemia, MESH:D015470	Dronabinc	14.36	9
MIR15A	406948	Leukemia, MESH:D01 therapeuti	Arsenic Tr	14.35	154
ATP5L	27425	Leukemia, MESH:D015473	arsenite|D	14.35	8
CCDC82	79780	Leukemia, MESH:D015470	Bortezomi	14.35	11
CTDSPLB	767676	Leukemia, MESH:D015470	Indometha	14.35	4
EI24	9538	Leukemia, MESH:D004915	Daunorub	14.35	2
FOXG1A	30274	Leukemia, MESH:D015470	Indometha	14.35	4
LHX4	89884	Leukemia, MESH:D015470	Calcitriol|D	14.35	6
MRPS11	64963	Leukemia, MESH:D015473	arsenite|D	14.35	8
QSOX2	169714	Leukemia, MESH:D015473	Genistein|I	14.35	152
RALGAPB	57148	Leukemia, MESH:D015473	arsenite|D	14.35	6
SPTSSB	165679	Leukemia, MESH:D015473	Arsenic|Ca	14.35	155
TMEM86B	255043	Leukemia, MESH:D015473	Arsenic|De	14.35	9
ZNF165	7718	Leukemia, MESH:D015473	arsenite|Ca	14.35	154
ATR	545	Leukemia, MESH:D007948	Arsenic Tr	14.34	3
CLDN8	9073	Leukemia, MESH:D015470	Azacitidine	14.34	9
DCUN1D5	84259	Leukemia, MESH:D015473	arsenite|Re	14.34	156
DMRT1	1761	Leukemia, MESH:D015470	Dronabinc	14.34	17
FAM161B	145483	Leukemia, MESH:D015473	Arsenic|Re	14.34	157
FMNL1	752	Leukemia, MESH:D015470	Benzene|C	14.34	22
IL18RAP	8807	Leukemia, MESH:D015470	Indometha	14.34	11
METTL5	29081	Leukemia, MESH:D015470	Gasoline|I	14.34	7
NCOR2	9612	Leukemia, MESH:D007948	2-(2-amin	14.34	2
PLN	5350	Leukemia, MESH:D015473	Dasatinib|	14.34	153

RCBTB1	55213	Leukemia, MESH:D015470		Mitoxantro	14.34	18
SEMA6B	10501	Leukemia, MESH:D015470		Alitretinoi	14.34	7
ST6GALNA	30815	Leukemia, MESH:D015470		Cytarabine	14.34	79
TMA16	55319	Leukemia, MESH:D015473		arsenite|G	14.34	154
TOMM22	56993	Leukemia, MESH:D015473		arsenite|Re	14.34	156
ASMTL	8623	Leukemia, MESH:D01	marker/me	Benzene|D	14.33	28
ARHGEF37	389337	Leukemia, MESH:D015470		Calcitriol|T	14.33	6
CLNS1A	1207	Leukemia, MESH:D015473		arsenite|G	14.33	154
CTC1	80169	Leukemia, MESH:D015470		Doxorubic	14.33	13
CYHR1	50626	Leukemia, MESH:D015470		Air Polluta	14.33	7
FXR1	8087	Leukemia, MESH:D004915		Doxorubic	14.33	2
H2AW	92815	Leukemia, MESH:D015470		(+)-JQ1 cc	14.33	7
INSC	387755	Leukemia, MESH:D015470		Dexameth	14.33	7
KPNA6	23633	Leukemia, MESH:D015470		Dexameth	14.33	7
MED18	54797	Leukemia, MESH:D015473		Arsenic|De	14.33	155
NMNAT3	349565	Leukemia, MESH:D015470		Decitabine	14.33	21
PI4KB	5298	Leukemia, MESH:D015470		Bortezomi	14.33	14
RUNDC3A	10900	Leukemia, MESH:D015470		Dexameth	14.33	8
TDH	157739	Leukemia, MESH:D015473		Dexameth	14.33	153
UBE3A	7337	Leukemia, MESH:D004915		Doxorubic	14.33	2
ANKRD13	81573	Leukemia, MESH:D015470		Indometha	14.32	9
ATP13A4	84239	Leukemia, MESH:D015470		Dexameth	14.32	14
AVEN	57099	Leukemia, MESH:D015470		Doxorubic	14.32	12
CNTLN	54875	Leukemia, MESH:D015470		Doxorubic	14.32	14
CYP2C8	1558	Leukemia, MESH:D015473		Dexameth	14.32	156
ESRRG	2104	Leukemia, MESH:D015473		Arsenic|Ar	14.32	263
FSTA	1E+08	Leukemia, MESH:D015470		Dexameth	14.32	4
GBA2	57704	Leukemia, MESH:D015470		Air Polluta	14.32	6
HBBE1.1	81538	Leukemia, MESH:D015470		Dexameth	14.32	4
IFNAR1	3454	Leukemia, MESH:D015470		Air Polluta	14.32	12
IRF3	3661	Leukemia, MESH:D015473		Arsenic Tr	14.32	261
JAML	120425	Leukemia, MESH:D015470		Air Polluta	14.32	7
LHCGR	3973	Leukemia, MESH:D007948		2-(2-amin	14.32	2
MAL	4118	Leukemia, MESH:D015473		Arsenic|ars	14.32	156
MANF	7873	Leukemia, MESH:D015473		Arsenic Tr	14.32	155
MB	4151	Leukemia, MESH:D015470		Dexameth	14.32	14
METTL18	92342	Leukemia, MESH:D015470		Dexameth	14.32	8
PARVG	64098	Leukemia, MESH:D015470		Calcitriol|(	14.32	7
RAPGEF1	2889	Leukemia, MESH:D015470		Bortezomi	14.32	13
SNX2	6643	Leukemia, MESH:D015470		Dexameth	14.32	7
TMEM70	54968	Leukemia, MESH:D015470		Arsenic Tr	14.32	18
TNIP2	79155	Leukemia, MESH:D015470		Dexameth	14.32	14
UQCRC1	7384	Leukemia, MESH:D015473		Arsenic|ars	14.32	13
USP39	10713	Leukemia, MESH:D015470		Dexameth	14.32	7
ZFP62	643836	Leukemia, MESH:D015470		Dexameth	14.32	12
AKIP1	56672	Leukemia, MESH:D015470		Doxorubic	14.31	11
AMMECR1	83607	Leukemia, MESH:D015470		Doxorubic	14.31	13
ARHGEF40	55701	Leukemia, MESH:D015470		Dronabino	14.31	7
ARL4A	10124	Leukemia, MESH:D015470		Air Polluta	14.31	9
ATXN7	6314	Leukemia, MESH:D015470		Doxorubic	14.31	12
BCAN	63827	Leukemia, MESH:D015470		Decitabine	14.31	14
CAD	790	Leukemia, MESH:D015473		Arsenic Tr	14.31	261
CHPF	79586	Leukemia, MESH:D015470		Calcitriol|D	14.31	11
DENND2B	6764	Leukemia, MESH:D015470		Arsenic Tr	14.31	18
EAF2	55840	Leukemia, MESH:D015470		Calcitriol|D	14.31	8
HEATR5A	25938	Leukemia, MESH:D015470		Doxorubic	14.31	11
HER8A	323656	Leukemia, MESH:D015470		Tretinoin|t	14.31	5
ID4	3400	Leukemia, MESH:D007948		Arsenic Tr	14.31	3

MACROH2	55506	Leukemia, MESH:D015470	Arsenic Tr	14.31	18
MT1IP	644314	Leukemia, MESH:D015470	Cytarabine	14.31	80
NKG7	4818	Leukemia, MESH:D015470	Dronabinc	14.31	7
PAQR3	152559	Leukemia, MESH:D015470	Decitabine	14.31	19
PHKG2	5261	Leukemia, MESH:D015473	Arsenic Tr	14.31	260
PON1	5444	Leukemia, MESH:D015473	Arsenic|Ch	14.31	13
RFX2	5990	Leukemia, MESH:D015470	Indometha	14.31	8
RHOC	389	Leukemia, MESH:D007948	Arsenic Tr	14.31	4
RPH3AL	9501	Leukemia, MESH:D015470	Dexameth	14.31	8
SCMH1	22955	Leukemia, MESH:D015470	Dexameth	14.31	7
SIPA1L3	23094	Leukemia, MESH:D015470	Doxorubic	14.31	12
SLC19A2	10560	Leukemia, MESH:D015473	arsenite|D	14.31	7
SLC38A7	55238	Leukemia, MESH:D015470	Dexameth	14.31	11
SNX33	257364	Leukemia, MESH:D015470	Calcitriol|D	14.31	11
TPP1	1200	Leukemia, MESH:D015473	Arsenic|Ar	14.31	263
VDAC2	7417	Leukemia, MESH:D015473	Arsenic Tr	14.31	262
WWP2	11060	Leukemia, MESH:D015470	Dronabinc	14.31	9
ANXA5	308	Leukemia, MESH:D015473	arsenite|G	14.3	157
CCT7	10574	Leukemia, MESH:D015470	Arsenic Tr	14.3	18
CD52	1043	Leukemia, MESH:D015470	Arsenic Tr	14.3	20
COX5B	1329	Leukemia, MESH:D015470	Decitabine	14.3	16
CYP3A2	266682	Leukemia, MESH:D015473	alpha-Toc	14.3	7
FBLIM1	54751	Leukemia, MESH:D015470	Calcitriol|(	14.3	9
FFAR2	2867	Leukemia, MESH:D015473	arsenite|G	14.3	153
GM2A	2760	Leukemia, MESH:D015470	Dexameth	14.3	9
GSTA2	2939	Leukemia, MESH:D015473	Arsenic Tr	14.3	157
GSTP1	2950	Leukemia, MESH:D007948	2-(2-amin	14.3	3
MAP2K3	5606	Leukemia, MESH:D007948	2-(2-amin	14.3	3
NDUFS1	4719	Leukemia, MESH:D007948	2-(2-amin	14.3	3
OSM	5008	Leukemia, MESH:D015470	Cyclophos	14.3	8
PYCR1	5831	Leukemia, MESH:D015470	Dexameth	14.3	8
RAB3GAP1	22930	Leukemia, MESH:D015473	arsenite|sc	14.3	157
ROR2	4920	Leukemia, MESH:D015470	Doxorubic	14.3	12
RPA3	6119	Leukemia, MESH:D004915	Doxorubic	14.3	2
TCEA3	6920	Leukemia, MESH:D015470	Arsenic Tr	14.3	13
TMEM51	55092	Leukemia, MESH:D015470	Doxorubic	14.3	12
TRNP1	388610	Leukemia, MESH:D015470	Dexameth	14.3	7
WDR1	9948	Leukemia, MESH:D015470	Dronabinc	14.3	10
ZDHHC2	51201	Leukemia, MESH:D015470	Arsenic Tr	14.3	20
AGTR1	185	Leukemia, MESH:D007948	Arsenic Tr	14.29	3
ESD	2098	Leukemia, MESH:D015473	Arsenic Tr	14.29	261
LSM14B	149986	Leukemia, MESH:D015473	arsenite|G	14.29	152
NTS	4922	Leukemia, MESH:D007948	Arsenic Tr	14.29	3
PFKFB4	5210	Leukemia, MESH:D015473	Arsenic|Ar	14.29	158
PTOV1	53635	Leukemia, MESH:D015473	arsenite|G	14.29	152
RASIP1	54922	Leukemia, MESH:D015473	Arsenic|De	14.29	10
ATP23	91419	Leukemia, MESH:D015473	Calcitriol|N	14.28	152
CAVIN3	112464	Leukemia, MESH:D004915	Cytarabine	14.28	3
CBLB	868	Leukemia, MESH:D015473	arsenite|Ca	14.28	7
GRPR	2925	Leukemia, MESH:D015470	Androgen	14.28	74
LRRC32	2615	Leukemia, MESH:D015473	Cytarabine	14.28	155
LRRC3B	116135	Leukemia, MESH:D015473	Arsenic|Ar	14.28	261
MLIP	90523	Leukemia, MESH:D015473	Arsenic Tr	14.28	154
OSGEPL1	64172	Leukemia, MESH:D015473	Arsenic|Ar	14.28	263
SH3KBP1	30011	Leukemia, MESH:D015473	Arsenic|ars	14.28	11
ACOD1	730249	Leukemia, MESH:D015470	Cyclophos	14.27	8
CCDC198	55195	Leukemia, MESH:D015470	Arsenic Tr	14.27	15
CCDC71L	168455	Leukemia, MESH:D015473	arsenite|D	14.27	6

DYNC1H1	1778	Leukemia, MESH:D015473	Arsenic|Ar	14.27	160
FAP	2191	Leukemia, MESH:D015473	Arsenic Tr	14.27	154
GABBR1	2550	Leukemia, MESH:D015473	Arsenic|Ar	14.27	265
HOXC9	3225	Leukemia, MESH:D015473	arsenite|D	14.27	153
NID1	4811	Leukemia, MESH:D015473	Arsenic|De	14.27	155
PSMA1	5682	Leukemia, MESH:D015473	Arsenic Tr	14.27	262
TRIM74	378108	Leukemia, MESH:D015470	Doxorubic	14.27	9
ZNF385B	151126	Leukemia, MESH:D015473	Arsenic|De	14.27	156
ABL1	25	Leukemia, MESH:D007948	2-(2-amin	14.26	3
ACTC1	70	Leukemia, MESH:D015473	Cytarabine	14.26	155
GABRR1	2569	Leukemia, MESH:D015473	Arsenic Tr	14.26	260
GALC	2581	Leukemia, MESH:D015473	arsenite|G	14.26	154
GARIN1B	84691	Leukemia, MESH:D015470	Decitabine	14.26	14
GLT8D2	83468	Leukemia, MESH:D015473	Calcitriol|D	14.26	153
LYPD3	27076	Leukemia, MESH:D015473	Arsenic|Ca	14.26	155
MATN2	4147	Leukemia, MESH:D004915	Cytarabine	14.26	2
MIR98	407054	Leukemia, MESH:D015473	Arsenic Tr	14.26	154
MRPL11	65003	Leukemia, MESH:D015473	arsenite|Re	14.26	156
PITPNM2	57605	Leukemia, MESH:D015473	Arsenic|M	14.26	154
PLB1	151056	Leukemia, MESH:D015473	arsenite|G	14.26	154
RBP2	5948	Leukemia, MESH:D015470	Alitretinoi	14.26	13
RXRB	6257	Leukemia, MESH:D015473	Arsenic|Ch	14.26	157
SGCE	8910	Leukemia, MESH:D015473	Arsenic|M	14.26	154
TEX261	113419	Leukemia, MESH:D015473	Arsenic Tr	14.26	153
TIGD2	166815	Leukemia, MESH:D015473	Arsenic Tr	14.26	155
ZNF549	256051	Leukemia, MESH:D015470	Dexameth	14.26	9
ZNF703	80139	Leukemia, MESH:D015473	Genistein|I	14.26	154
CAVIN1	284119	Leukemia, MESH:D004915	Cytarabine	14.25	3
CCDC190	339512	Leukemia, MESH:D015470	Dronabinc	14.25	6
CCDC25	55246	Leukemia, MESH:D015473	Arsenic|Ge	14.25	155
CES3	23491	Leukemia, MESH:D015470	Dexameth	14.25	11
COL15A1	1306	Leukemia, MESH:D004915	Doxorubic	14.25	2
GOLGA2P	55592	Leukemia, MESH:D015470	Dexameth	14.25	7
H4C3	8364	Leukemia, MESH:D004915	Cytarabine	14.25	2
HOXD8	3234	Leukemia, MESH:D015473	Dexameth	14.25	153
MAGI1	9223	Leukemia, MESH:D004915	Cytarabine	14.25	2
MAP3K1	4214	Leukemia, MESH:D007948	pyrazolant	14.25	2
MTCL1	23255	Leukemia, MESH:D015473	Dexameth	14.25	153
PMEL	6490	Leukemia, MESH:D015473	Cytarabine	14.25	153
PPARA	5465	Leukemia, MESH:D007948	Arsenic Tr	14.25	3
PRKCZ	5590	Leukemia, MESH:D007948	2-(2-amin	14.25	3
SHH	6469	Leukemia, MESH:D007948	Arsenic Tr	14.25	3
TNFA	405785	Leukemia, MESH:D015470	Arsenic Tr	14.25	15
TPM1	7168	Leukemia, MESH:D015473	Dexameth	14.25	156
WARS2	10352	Leukemia, MESH:D015473	Arsenic|ars	14.25	156
ZFHX4	79776	Leukemia, MESH:D004915	Cytarabine	14.25	2
COL24A1	255631	Leukemia, MESH:D015473	Arsenic|De	14.24	155
DEFB1	1672	Leukemia, MESH:D015470	Calcitriol|D	14.24	8
EHBP1	23301	Leukemia, MESH:D004915	Doxorubic	14.24	2
FUBP3	8939	Leukemia, MESH:D015473	arsenite|D	14.24	154
GLIPR1	11010	Leukemia, MESH:D015473	Arsenic|Ar	14.24	264
INHA	3623	Leukemia, MESH:D015470	Dexameth	14.24	16
MRPS6	64968	Leukemia, MESH:D015470	Calcitriol|D	14.24	9
NACC1	112939	Leukemia, MESH:D015470	Chloroqui	14.24	10
NDUFAF2	91942	Leukemia, MESH:D015473	arsenite|D	14.24	154
NFATC3	4775	Leukemia, MESH:D004915	Doxorubic	14.24	2
PARD3	56288	Leukemia, MESH:D004915	Doxorubic	14.24	2
POLE	5426	Leukemia, MESH:D015470	Doxorubic	14.24	14

PSENEN	55851	Leukemia, MESH:D015473	arsenite|D	14.24	154
RBBP6	5930	Leukemia, MESH:D015470	Daunorub	14.24	38
RNF130	55819	Leukemia, MESH:D015473	Arsenic|De	14.24	155
RSRC2	65117	Leukemia, MESH:D015470	Dexameth	14.24	8
SCAMP2	10066	Leukemia, MESH:D015470	Doxorubic	14.24	14
SKIL	6498	Leukemia, MESH:D015470	Decitabine	14.24	17
SLC25A30	253512	Leukemia, MESH:D015470	Dexameth	14.24	8
SMCO4	56935	Leukemia, MESH:D015473	Arsenic|De	14.24	155
TM2D2	83877	Leukemia, MESH:D015470	Benzene|B	14.24	21
XPO1	7514	Leukemia, MESH:D015473	Arsenic Tr	14.24	262
ZAK	559247	Leukemia, MESH:D015470	Dexameth	14.24	5
ARX	170302	Leukemia, MESH:D015470	Dronabinc	14.23	6
CDIP1	29965	Leukemia, MESH:D015470	Arsenic Tr	14.23	18
CREB5	9586	Leukemia, MESH:D015473	Arsenic Tr	14.23	262
DAZAP2	9802	Leukemia, MESH:D015470	Arsenic Tr	14.23	13
DDX54	79039	Leukemia, MESH:D015470	Doxorubic	14.23	12
DSP	1832	Leukemia, MESH:D015473	Arsenic Tr	14.23	261
DYM	54808	Leukemia, MESH:D015470	Doxorubic	14.23	12
ETFA	2108	Leukemia, MESH:D015473	Arsenic Tr	14.23	156
FMR1	2332	Leukemia, MESH:D015473	arsenite|D	14.23	157
GPR182	11318	Leukemia, MESH:D015470	Chloroqui	14.23	8
LGALSL	29094	Leukemia, MESH:D015470	Arsenic Tr	14.23	18
LURAP1L	286343	Leukemia, MESH:D015470	Dexameth	14.23	7
NRROS	375387	Leukemia, MESH:D015470	Decitabine	14.23	14
RAP1A	5906	Leukemia, MESH:D015473	Arsenic|ar:	14.23	159
SERPINB3	6317	Leukemia, MESH:D015470	Calcitriol|C	14.23	7
TCN2	6948	Leukemia, MESH:D015470	Dexameth	14.23	15
TRPM7	54822	Leukemia, MESH:D015470	Calcitriol|D	14.23	12
ZNF148	7707	Leukemia, MESH:D015473	Dexameth	14.23	157
ADGRA3	166647	Leukemia, MESH:D015470	Dexameth	14.22	11
ADGRF1	266977	Leukemia, MESH:D015470	(+)-JQ1 cc	14.22	7
BLOC1S6	26258	Leukemia, MESH:D015470	Dronabinc	14.22	7
CADM3	57863	Leukemia, MESH:D015470	Calcitriol|D	14.22	6
CSPG5	10675	Leukemia, MESH:D015470	Arsenic Tr	14.22	12
CTSH	1512	Leukemia, MESH:D007948	Arsenic Tr	14.22	4
CWC22	57703	Leukemia, MESH:D015470	(+)-JQ1 cc	14.22	7
ELOVL4	6785	Leukemia, MESH:D015470	(+)-JQ1 cc	14.22	8
FTSJ1	24140	Leukemia, MESH:D015470	Doxorubic	14.22	12
GET3	439	Leukemia, MESH:D015470	Arsenic Tr	14.22	17
MEAK7	57707	Leukemia, MESH:D015470	Arsenic Tr	14.22	14
NCBP2	22916	Leukemia, MESH:D015470	Dexameth	14.22	8
NEDD8	4738	Leukemia, MESH:D015470	Arsenic Tr	14.22	13
OLR1	4973	Leukemia, MESH:D007948	Arsenic Tr	14.22	4
POLR1F	221830	Leukemia, MESH:D015470	Dexameth	14.22	11
ROMO1	140823	Leukemia, MESH:D015470	Arsenic Tr	14.22	18
SSH1	54434	Leukemia, MESH:D015473	Arsenic|ar:	14.22	11
TCEAL2	140597	Leukemia, MESH:D015470	Decitabine	14.22	12
TMEM88	92162	Leukemia, MESH:D015470	Cytarabine	14.22	78
UNC13A	23025	Leukemia, MESH:D015470	Dexameth	14.22	10
ZNF658	26149	Leukemia, MESH:D015470	Dronabinc	14.22	7
ADAM28	10863	Leukemia, MESH:D015470	Calcitriol|T	14.21	7
AHDC1	27245	Leukemia, MESH:D015470	Dexameth	14.21	13
CSPRS	114564	Leukemia, MESH:D015470	Dronabinc	14.21	7
DCUN1D3	123879	Leukemia, MESH:D015470	Arsenic Tr	14.21	13
DNAH2	146754	Leukemia, MESH:D015473	Cytarabine	14.21	14
ELL	8178	Leukemia, MESH:D015470	Doxorubic	14.21	14
FBXL3	26224	Leukemia, MESH:D015470	Dexameth	14.21	11
GALNT14	79623	Leukemia, MESH:D015470	Doxorubic	14.21	12

IRX2	153572	Leukemia, MESH:D015470		Dronabinc	14.21	7
KCTD16	57528	Leukemia, MESH:D015470		Doxorubic	14.21	10
KIF16B	55614	Leukemia, MESH:D015470		Doxorubic	14.21	11
LDAH	60526	Leukemia, MESH:D015470		Dexameth	14.21	8
MAFA	389692	Leukemia, MESH:D015470		Dexameth	14.21	6
MPPE1	65258	Leukemia, MESH:D015470		Dexameth	14.21	11
PNISR	25957	Leukemia, MESH:D004915		Doxorubic	14.21	2
RBBP6	5930	Leukemia, MESH:D004915		Daunorub	14.21	3
RDH12L	494176	Leukemia, MESH:D015470		Dexameth	14.21	5
SYT9	143425	Leukemia, MESH:D015470		Doxorubic	14.21	12
TMEM268	203197	Leukemia, MESH:D015470		Doxorubic	14.21	11
TRMT10C	54931	Leukemia, MESH:D015470		Doxorubic	14.21	12
TSPAN14	81619	Leukemia, MESH:D015470		Arsenic Tr	14.21	17
XIRP1	165904	Leukemia, MESH:D015470		Doxorubic	14.21	13
DSCAML1	57453	Leukemia, MESH:D015470		Dexameth	14.2	12
H2AC14	8331	Leukemia, MESH:D015470		Dexameth	14.2	12
KIAA0825	285600	Leukemia, MESH:D015470		Doxorubic	14.2	10
KIAA1143	57456	Leukemia, MESH:D015470		Dexameth	14.2	6
MIR503HC	84848	Leukemia, MESH:D015470		Arsenic Tr	14.2	12
MYOZ2	51778	Leukemia, MESH:D015470		Doxorubic	14.2	12
SFRP1	6422	Leukemia, MESH:D007948		Arsenic Tr	14.2	4
STXBP5	134957	Leukemia, MESH:D015470		Doxorubic	14.2	12
TMEM40	55287	Leukemia, MESH:D015473		Calcitriol|C	14.2	6
ZNF506	440515	Leukemia, MESH:D015470		Doxorubic	14.2	10
ITGAL	3683	Leukemia, MESH:D01	marker/m	arsenite|M	14.19	157
ATP1B3	483	Leukemia, MESH:D015470		Benzene|C	14.19	24
C4ORF48	401115	Leukemia, MESH:D015470		Dexameth	14.19	5
CAR2	12349	Leukemia, MESH:D015470		Arsenic Tr	14.19	14
FDPS	2224	Leukemia, MESH:D015473		Antimony	14.19	156
GRIA2	2891	Leukemia, MESH:D015470		Arsenic Tr	14.19	81
NBPF3	84224	Leukemia, MESH:D015470		Dexameth	14.19	5
PLCG2	5336	Leukemia, MESH:D015473		Arsenic Tr	14.19	263
RAD54L	8438	Leukemia, MESH:D004915		Doxorubic	14.19	2
RIPK3	11035	Leukemia, MESH:D015473		Arsenic|Ge	14.19	157
RPL22	6146	Leukemia, MESH:D015470		Air Polluta	14.19	21
RPS6KA1	6195	Leukemia, MESH:D015473		arsenite|Ca	14.19	157
SLC24A2	25769	Leukemia, MESH:D015473		Calcitriol|D	14.19	5
SLC37A4	2542	Leukemia, MESH:D015473		Arsenic Tr	14.19	260
SORBS1	10580	Leukemia, MESH:D015473		Arsenic|De	14.19	13
ZNF204P	7754	Leukemia, MESH:D015470		Tobacco S	14.19	6
APOBEC3C	27350	Leukemia, MESH:D015473		Etoposide	14.18	153
CD38	952	Leukemia, MESH:D007948		2-(2-amin	14.18	3
ESM1	11082	Leukemia, MESH:D015470		Dexameth	14.18	17
MCTP2	55784	Leukemia, MESH:D015473		Arsenic Tr	14.18	260
METAP2	10988	Leukemia, MESH:D004915		Doxorubic	14.18	2
NKAIN4	128414	Leukemia, MESH:D015473		Dexameth	14.18	5
PRDM2	7799	Leukemia, MESH:D004915		Doxorubic	14.18	2
RFTN1	23180	Leukemia, MESH:D004915		Doxorubic	14.18	2
RMND1	55005	Leukemia, MESH:D015473		Arsenic Tr	14.18	260
SERPINI1	5274	Leukemia, MESH:D015470		Calcitriol|D	14.18	13
SF1	7536	Leukemia, MESH:D004915		Doxorubic	14.18	2
SLC12A2	6558	Leukemia, MESH:D015470		Dexameth	14.18	14
TAPBPL	55080	Leukemia, MESH:D015473		Arsenic|ar:	14.18	155
ANKMY2	57037	Leukemia, MESH:D015473		Calcitriol|D	14.17	153
APOL1	8542	Leukemia, MESH:D015473		Calcitriol|D	14.17	153
ARSI	340075	Leukemia, MESH:D015473		Calcitriol|D	14.17	153
BCLAF1	9774	Leukemia, MESH:D015473		arsenite|D	14.17	157
CNDP2	55748	Leukemia, MESH:D015470		Dexameth	14.17	8

DAP	1611	Leukemia, MESH:D015470		Dexameth	14.17	13
GPCPD1	56261	Leukemia, MESH:D015470		Dexameth	14.17	13
ICAM3	3385	Leukemia, MESH:D015473		Arsenic Tr	14.17	263
L3HYPDH	112849	Leukemia, MESH:D015473		Calcitriol|D	14.17	153
OSGEP	55644	Leukemia, MESH:D015473		Arsenic|ars	14.17	10
PSMC3	5702	Leukemia, MESH:D015470		Arsenic Tr	14.17	19
SEC24D	9871	Leukemia, MESH:D015470		Doxorubic	14.17	15
TENT5A	55603	Leukemia, MESH:D004915		Cytarabine	14.17	2
TOP1	7150	Leukemia, MESH:D007948		Arsenic Tr	14.17	3
ABCG2	9429	Leukemia, MESH:D004915		Daunorub	14.16	2
CNN1	1264	Leukemia, MESH:D015473		Dasatinib|	14.16	153
DAAM2	23500	Leukemia, MESH:D015473		Arsenic|De	14.16	155
LASP1	3927	Leukemia, MESH:D015473		Arsenic Tr	14.16	264
MIR142	406934	Leukemia, MESH:D015473		Arsenic|M	14.16	11
MYL6B	140465	Leukemia, MESH:D015473		arsenite|D	14.16	154
OGA	10724	Leukemia, MESH:D015473		Dexameth	14.16	157
PIP5K1A	8394	Leukemia, MESH:D015470		Azacitidine	14.16	13
PWP1	11137	Leukemia, MESH:D015473		Dexameth	14.16	155
SSH2	85464	Leukemia, MESH:D015473		Dexameth	14.16	155
DSEL	92126	Leukemia, MESH:D015470		Calcitriol|r	14.15	7
NOX1	27035	Leukemia, MESH:D015470		Arsenic Tr	14.15	20
NRXN1	9378	Leukemia, MESH:D015473		Arsenic|Ge	14.15	155
PGRMC1	10857	Leukemia, MESH:D015473		arsenite|D	14.15	8
PRDM1	639	Leukemia, MESH:D015473		Arsenic|ars	14.15	156
RAB1B	81876	Leukemia, MESH:D015470		Azacitidine	14.15	13
ROBO3	64221	Leukemia, MESH:D015470		Cytarabine	14.15	77
SLC34A1	6569	Leukemia, MESH:D015470		Cyclophos	14.15	6
HOXA9	3205	Leukemia, MESH:D01	marker/m	Arsenic Tr	14.14	20
BGN	633	Leukemia, MESH:D015473		caffeic aci	14.14	152
C5	727	Leukemia, MESH:D007948		2-(2-amin	14.14	3
GON4L	54856	Leukemia, MESH:D015470		Doxorubic	14.14	14
IPO8	10526	Leukemia, MESH:D015470		Air Polluta	14.14	6
MT2	17750	Leukemia, MESH:D015470		Deferoxan	14.14	14
NDUFB7	4713	Leukemia, MESH:D015470		Doxorubic	14.14	12
PIK3C2A	5286	Leukemia, MESH:D015470		Decitabine	14.14	17
RGN	9104	Leukemia, MESH:D015470		Dexameth	14.14	11
SF3B2	10992	Leukemia, MESH:D015470		Doxorubic	14.14	12
SLC30A6	55676	Leukemia, MESH:D015473		Arsenic|ars	14.14	11
SMARCA2	6595	Leukemia, MESH:D015473		Arsenic Tr	14.14	260
TFF3	7033	Leukemia, MESH:D004915		Cytarabine	14.14	2
TULP3	7289	Leukemia, MESH:D015473		Dexameth	14.14	155
ACTL6A	86	Leukemia, MESH:D015470		Air Polluta	14.13	12
ACTR6	64431	Leukemia, MESH:D015470		Dexameth	14.13	7
BAZ2A	11176	Leukemia, MESH:D015470		Calcitriol|D	14.13	8
CD24A	12484	Leukemia, MESH:D015470		Dexameth	14.13	12
COL22A1	169044	Leukemia, MESH:D015470		Calcitriol|D	14.13	19
FCGR1A	2209	Leukemia, MESH:D015470		Air Polluta	14.13	12
GYS2	2998	Leukemia, MESH:D015470		Decitabine	14.13	16
ISL2	64843	Leukemia, MESH:D015470		Arsenic Tr	14.13	12
LUM	4060	Leukemia, MESH:D007948		Cytarabine	14.13	3
MIR122	406906	Leukemia, MESH:D015470		Arsenic Tr	14.13	40
MIR132	406921	Leukemia, MESH:D015470		Arsenic Tr	14.13	21
MRPL51	51258	Leukemia, MESH:D015470		Air Polluta	14.13	11
MSL1	339287	Leukemia, MESH:D015470		Doxorubic	14.13	12
MYH6	4624	Leukemia, MESH:D007948		Arsenic Tr	14.13	3
NEIL2	252969	Leukemia, MESH:D015473		Arsenic|Ge	14.13	9
NXT1	29107	Leukemia, MESH:D015470		Dronabinc	14.13	9
ORC3	23595	Leukemia, MESH:D015470		Doxorubic	14.13	12

PIKFYVE	200576	Leukemia, MESH:D015473	arsenite|sc	14.13	157
PTPN9	5780	Leukemia, MESH:D015470	Decitabine	14.13	18
PTTG2	10744	Leukemia, MESH:D015473	Arsenic|Ca	14.13	8
RPS6KA2	6196	Leukemia, MESH:D015470	Cyclophos	14.13	14
SARM1	23098	Leukemia, MESH:D015470	Arsenic Tr	14.13	15
SCAF8	22828	Leukemia, MESH:D015470	Air Polluta	14.13	7
TBC1D4	9882	Leukemia, MESH:D004915	Cytarabine	14.13	3
URB1	9875	Leukemia, MESH:D015470	Dexameth	14.13	13
ZFAND2B	130617	Leukemia, MESH:D015470	Benzene|D	14.13	28
ALKBH8	91801	Leukemia, MESH:D015470	(+)-JQ1 cc	14.12	8
ALOX12B	242	Leukemia, MESH:D015470	Decitabine	14.12	18
ARHGEF18	23370	Leukemia, MESH:D015470	Calcitriol|D	14.12	12
CANT1	124583	Leukemia, MESH:D015470	Dexameth	14.12	6
CAST	831	Leukemia, MESH:D015473	Arsenic|ars	14.12	156
CSNK1A1	1452	Leukemia, MESH:D015470	Doxorubic	14.12	15
EFCAB2	84288	Leukemia, MESH:D015470	Doxorubic	14.12	12
EFR3B	22979	Leukemia, MESH:D015470	Decitabine	14.12	12
GNG10	2790	Leukemia, MESH:D015470	Doxorubic	14.12	11
HELQ	113510	Leukemia, MESH:D015470	Dexameth	14.12	11
HM13	81502	Leukemia, MESH:D015470	Arsenic Tr	14.12	13
IQGAP3	128239	Leukemia, MESH:D015470	Calcitriol|D	14.12	13
KBTBD2	25948	Leukemia, MESH:D015470	Air Polluta	14.12	11
KCNG3	170850	Leukemia, MESH:D015470	(+)-JQ1 cc	14.12	7
LONP1	9361	Leukemia, MESH:D015470	Dexameth	14.12	10
MIR760	1E+08	Leukemia, MESH:D015470	Doxorubic	14.12	10
MLH3	27030	Leukemia, MESH:D015470	Decitabine	14.12	16
MSRA	4482	Leukemia, MESH:D015470	Arsenic Tr	14.12	23
MVP	9961	Leukemia, MESH:D007948	Arsenic Tr	14.12	3
PALS1	64398	Leukemia, MESH:D015470	Arsenic Tr	14.12	13
PEX26	55670	Leukemia, MESH:D015470	Doxorubic	14.12	11
RAI14	26064	Leukemia, MESH:D004915	Doxorubic	14.12	2
SAMD5	389432	Leukemia, MESH:D015470	Decitabine	14.12	16
SMNDC1	10285	Leukemia, MESH:D015470	Doxorubic	14.12	13
SNAP91	9892	Leukemia, MESH:D015473	arsenite|Cy	14.12	156
SNX18	112574	Leukemia, MESH:D015473	Calcitriol|N	14.12	7
SORBS2	8470	Leukemia, MESH:D004915	Cytarabine	14.12	2
USF2	7392	Leukemia, MESH:D015470	Arsenic Tr	14.12	13
VPS52	6293	Leukemia, MESH:D015470	Dexameth	14.12	7
ANKRD2	26287	Leukemia, MESH:D015470	Arsenic Tr	14.11	17
BMP1	649	Leukemia, MESH:D015470	Decitabine	14.11	22
BSG	682	Leukemia, MESH:D015473	Arsenic Tr	14.11	155
CDC123	8872	Leukemia, MESH:D015470	Doxorubic	14.11	12
CHMP2A	27243	Leukemia, MESH:D015470	Doxorubic	14.11	12
CPNE2	221184	Leukemia, MESH:D015470	Doxorubic	14.11	12
DCP2	167227	Leukemia, MESH:D015473	Arsenic Tr	14.11	156
DHPS	1725	Leukemia, MESH:D015470	Doxorubic	14.11	12
GNAT2	2780	Leukemia, MESH:D015470	Dexameth	14.11	7
GRIN3A	116443	Leukemia, MESH:D015473	arsenite|G	14.11	6
HAUS7	55559	Leukemia, MESH:D015473	Arsenic|ars	14.11	10
LYPD6	130574	Leukemia, MESH:D015470	Resveratro	14.11	9
MRPL39	54148	Leukemia, MESH:D015473	arsenite|sc	14.11	154
MRPS34	65993	Leukemia, MESH:D015470	Doxorubic	14.11	12
NCOR1	9611	Leukemia, MESH:D007948	2-(2-amin	14.11	2
PSMA2	5683	Leukemia, MESH:D015473	arsenite|D	14.11	7
PSMB7	5695	Leukemia, MESH:D015470	Dexameth	14.11	13
RTCA	8634	Leukemia, MESH:D015470	Doxorubic	14.11	13
SLCO1A2	6579	Leukemia, MESH:D015473	Calcitriol|C	14.11	153
SSU72	29101	Leukemia, MESH:D015470	Dexameth	14.11	11

TSKU	25987	Leukemia,	MESH:D015473	Arsenic Tr	14.11	259
VARS2	57176	Leukemia,	MESH:D015470	Dexameth	14.11	7
VMP1	81671	Leukemia,	MESH:D015470	Doxorubic	14.11	13
CRHBP	1393	Leukemia,	MESH:D015473	arsenite|D	14.1	153
EMP3	2014	Leukemia,	MESH:D004915	Doxorubic	14.1	3
FLNA	2316	Leukemia,	MESH:D015470	Benzene|D	14.1	34
GABPB2	126626	Leukemia,	MESH:D015473	Arsenic|Ar	14.1	158
HSPB3	8988	Leukemia,	MESH:D015473	Calcitriol|C	14.1	153
MPHOSPH	10199	Leukemia,	MESH:D015473	Arsenic|De	14.1	156
MSX1	4487	Leukemia,	MESH:D004915	Cytarabine	14.1	2
NPY2R	4887	Leukemia,	MESH:D015473	Genistein|I	14.1	152
PML	5371	Leukemia,	MESH:D007948	Arsenic Tr	14.1	3
SESN3	143686	Leukemia,	MESH:D004915	Cytarabine	14.1	2
SRSF5	6430	Leukemia,	MESH:D015473	Antimony	14.1	157
VNN1	8876	Leukemia,	MESH:D015470	Arsenic Tr	14.1	20
APOC1	341	Leukemia,	MESH:D015473	Dexameth	14.09	7
FOXG1B	405850	Leukemia,	MESH:D015473	arsenite|D	14.09	152
GLB1	2720	Leukemia,	MESH:D007948	Arsenic Tr	14.09	3
MAPK3	5595	Leukemia,	MESH:D007948	2-(2-amin	14.09	3
MRPS2	51116	Leukemia,	MESH:D015473	arsenite|Re	14.09	156
MYLIP	29116	Leukemia,	MESH:D015473	Arsenic|Ca	14.09	157
PLA2G2A	5320	Leukemia,	MESH:D007948	2-(2-amin	14.09	2
PLP1	5354	Leukemia,	MESH:D004915	Cytarabine	14.09	2
RARS1	5917	Leukemia,	MESH:D015473	Arsenic Tr	14.09	262
SERGEF	26297	Leukemia,	MESH:D015473	Arsenic|Ar	14.09	263
TP53I3	9540	Leukemia,	MESH:D004915	Daunorub	14.09	2
TTPA	7274	Leukemia,	MESH:D004915	Doxorubic	14.09	2
CSRP3	8048	Leukemia,	MESH:D004915	Doxorubic	14.08	2
E2F6	1876	Leukemia,	MESH:D015473	Arsenic|Ar	14.08	261
FAHD2A	51011	Leukemia,	MESH:D015473	Arsenic Tr	14.08	260
GPT2	84706	Leukemia,	MESH:D015473	Arsenic Tr	14.08	261
LAMTOR5	10542	Leukemia,	MESH:D015473	Arsenic Tr	14.08	260
NOL11	25926	Leukemia,	MESH:D015473	Dexameth	14.08	155
NUDT19	390916	Leukemia,	MESH:D015473	Dexameth	14.08	153
SDHA	6389	Leukemia,	MESH:D007948	Arsenic Tr	14.08	3
BEND3	57673	Leukemia,	MESH:D015473	Arsenic|De	14.07	155
CALCR	799	Leukemia,	MESH:D015470	Cytarabine	14.07	76
CST1	1469	Leukemia,	MESH:D015470	Etoposide	14.07	16
HOXC6	3223	Leukemia,	MESH:D015473	arsenite|Cy	14.07	157
KCNN4	3783	Leukemia,	MESH:D015473	arsenite|D	14.07	155
MAPK1	5594	Leukemia,	MESH:D007948	2-(2-amin	14.07	3
MGME1	92667	Leukemia,	MESH:D015470	Air Polluta	14.07	8
MR1	3140	Leukemia,	MESH:D004915	Doxorubic	14.07	3
MRPL52	122704	Leukemia,	MESH:D015473	arsenite|D	14.07	154
PABPC3	5042	Leukemia,	MESH:D015470	Arsenic Tr	14.07	18
PELI1	57162	Leukemia,	MESH:D004915	Cytarabine	14.07	3
SMC1A	8243	Leukemia,	MESH:D015473	arsenite|M	14.07	156
ZNF511	118472	Leukemia,	MESH:D015470	Gasoline|P	14.07	7
ABCE1	6059	Leukemia,	MESH:D015470	Docetaxel|	14.06	13
ADCY6	112	Leukemia,	MESH:D015470	Dexameth	14.06	7
CDC14B	8555	Leukemia,	MESH:D004915	Doxorubic	14.06	2
CMBL	134147	Leukemia,	MESH:D015473	Arsenic|De	14.06	158
EVC	2121	Leukemia,	MESH:D015473	arsenite|Ca	14.06	153
GLP2R	9340	Leukemia,	MESH:D015470	Dexameth	14.06	6
LSM14A	26065	Leukemia,	MESH:D015473	arsenite|D	14.06	156
MIR34C	407042	Leukemia,	MESH:D015473	Arsenic Tr	14.06	156
RGS3	5998	Leukemia,	MESH:D015470	Benzene|C	14.06	22
RGS6	9628	Leukemia,	MESH:D015473	caffeic aci	14.06	5

SCIMP	388325	Leukemia, MESH:D015470	Air Polluta	14.06	7
TBPL1	9519	Leukemia, MESH:D015470	Etoposide	14.06	15
ABCA4	24	Leukemia, MESH:D015473	arsenite|G	14.05	7
ANKRD50	57182	Leukemia, MESH:D015470	Air Polluta	14.05	7
CYTH3	9265	Leukemia, MESH:D015470	Dexameth	14.05	10
IL7	3574	Leukemia, MESH:D015473	Arsenic|De	14.05	14
KYNU	8942	Leukemia, MESH:D007948	Arsenic Tr	14.05	4
MARVELD	153562	Leukemia, MESH:D015470	Dronabinc	14.05	11
MITF	4286	Leukemia, MESH:D007948	2-(2-amir	14.05	2
MRPL30	51263	Leukemia, MESH:D015473	Arsenic Tr	14.05	157
MTF1	4520	Leukemia, MESH:D015470	Arsenic Tr	14.05	15
NDC80	10403	Leukemia, MESH:D007948	Arsenic Tr	14.05	4
NUP210	23225	Leukemia, MESH:D015470	Dexameth	14.05	9
PCTP	58488	Leukemia, MESH:D015470	Calcitriol|D	14.05	13
PLCE1	51196	Leukemia, MESH:D015470	Doxorubic	14.05	13
RNLS	55328	Leukemia, MESH:D015473	Arsenic Tr	14.05	154
SPDL1	54908	Leukemia, MESH:D015470	Calcitriol|D	14.05	14
ARHGAP5	84837	Leukemia, MESH:D015470	Calcitriol|D	14.04	5
ASB7	140460	Leukemia, MESH:D015473	Arsenic|De	14.04	8
BMERB1	89927	Leukemia, MESH:D015473	Calcitriol|C	14.04	6
CHD6	84181	Leukemia, MESH:D015470	Decitabine	14.04	14
DCT	1638	Leukemia, MESH:D004915	Doxorubic	14.04	2
MAZ	4150	Leukemia, MESH:D015470	Bortezomi	14.04	8
MRPS30	10884	Leukemia, MESH:D015470	Arsenic Tr	14.04	13
NDP	4693	Leukemia, MESH:D015470	Arsenic Tr	14.04	15
NEDD1	121441	Leukemia, MESH:D015470	Arsenic Tr	14.04	17
NPAS1	4861	Leukemia, MESH:D015473	Arsenic Tr	14.04	260
NT5C	30833	Leukemia, MESH:D015470	Arsenic Tr	14.04	13
ORC2	4999	Leukemia, MESH:D015473	Dexameth	14.04	156
RPS6KB2	6199	Leukemia, MESH:D015470	Arsenic Tr	14.04	18
SDE2	163859	Leukemia, MESH:D015470	Air Polluta	14.04	12
SLAMF1	6504	Leukemia, MESH:D015473	Dexameth	14.04	5
TEK	7010	Leukemia, MESH:D015473	Arsenic Tr	14.04	163
TIMM21	29090	Leukemia, MESH:D015470	Calcitriol|D	14.04	13
TINAG	27283	Leukemia, MESH:D015473	Calcitriol|D	14.04	6
TRIM29	23650	Leukemia, MESH:D015470	Calcitriol|T	14.04	11
ANAPC11	51529	Leukemia, MESH:D015470	Bortezomi	14.03	13
BHMT2	23743	Leukemia, MESH:D015470	Decitabine	14.03	13
CZIB	54987	Leukemia, MESH:D015470	Arsenic Tr	14.03	11
DDB2	1643	Leukemia, MESH:D015473	Arsenic|Ar	14.03	160
EMD	2010	Leukemia, MESH:D015470	Arsenic Tr	14.03	13
EXOSC3	51010	Leukemia, MESH:D015470	Dexameth	14.03	7
GJB6	10804	Leukemia, MESH:D015470	(+)-JQ1 cc	14.03	7
GLT1D1	144423	Leukemia, MESH:D015470	Air Polluta	14.03	7
GSC	145258	Leukemia, MESH:D015473	Arsenic|Ar	14.03	263
IL18BP	10068	Leukemia, MESH:D015470	Doxorubic	14.03	12
ILF3	3609	Leukemia, MESH:D004915	Doxorubic	14.03	2
ING4	51147	Leukemia, MESH:D015470	Doxorubic	14.03	11
JPT2	90861	Leukemia, MESH:D015470	Doxorubic	14.03	13
KCNC1	3746	Leukemia, MESH:D015473	Arsenic|ars	14.03	155
KLHL23	151230	Leukemia, MESH:D015470	Doxorubic	14.03	15
KPNA2	3838	Leukemia, MESH:D007948	Arsenic Tr	14.03	4
MGAT3	4248	Leukemia, MESH:D015470	Decitabine	14.03	18
MIR15B	406949	Leukemia, MESH:D015473	Arsenic|Ar	14.03	263
MT1G	4495	Leukemia, MESH:D015473	Arsenic|Ar	14.03	160
MUC5AC	4586	Leukemia, MESH:D007948	2-(2-amir	14.03	2
PLSCR2	57047	Leukemia, MESH:D015470	Dexameth	14.03	8
PPP1R12B	4660	Leukemia, MESH:D015470	Doxorubic	14.03	11

RABL3	285282	Leukemia, MESH:D015470	Arsenic Tr	14.03	18
SARNP	84324	Leukemia, MESH:D015470	Benzene|D	14.03	27
SCAF4	57466	Leukemia, MESH:D015470	Doxorubic	14.03	11
SDHAF3	57001	Leukemia, MESH:D015470	Dexameth	14.03	11
SEPTIN7	989	Leukemia, MESH:D015470	Arsenic Tr	14.03	12
SH3TC2	79628	Leukemia, MESH:D015470	Dexameth	14.03	10
SINHCAF	58516	Leukemia, MESH:D015470	Doxorubic	14.03	11
STRADA	92335	Leukemia, MESH:D015470	(+)-JQ1 cc	14.03	8
SYNGR2	9144	Leukemia, MESH:D015470	Bortezomi	14.03	13
TNS4	84951	Leukemia, MESH:D015470	Arsenic Tr	14.03	12
TTBK2	146057	Leukemia, MESH:D015470	Decitabine	14.03	12
ZFYVE28	57732	Leukemia, MESH:D015470	Decitabine	14.03	13
ZNF107	51427	Leukemia, MESH:D015470	Arsenic Tr	14.03	12
ACP6	51205	Leukemia, MESH:D015470	Doxorubic	14.02	12
ADAMTS1	81792	Leukemia, MESH:D015470	Doxorubic	14.02	12
AMN	81693	Leukemia, MESH:D015470	Dronabinc	14.02	9
CBLN1	869	Leukemia, MESH:D015470	Dexameth	14.02	7
CLTRN	57393	Leukemia, MESH:D015473	arsenite|Ca	14.02	154
CYTL1	54360	Leukemia, MESH:D015470	Arsenic Tr	14.02	12
ENTPD7	57089	Leukemia, MESH:D015470	Doxorubic	14.02	14
FBXO4	26272	Leukemia, MESH:D015473	Arsenic|Ca	14.02	9
FKBP7	51661	Leukemia, MESH:D015470	Dexameth	14.02	11
H2-AB1	14961	Leukemia, MESH:D015470	Dexameth	14.02	11
HDAC11	79885	Leukemia, MESH:D015470	Calcitriol|T	14.02	7
HSPA6	3310	Leukemia, MESH:D007948	Arsenic Tr	14.02	3
KMT5A	387893	Leukemia, MESH:D015470	Doxorubic	14.02	12
LGALS2B	393486	Leukemia, MESH:D015470	Dexameth	14.02	5
MED30	90390	Leukemia, MESH:D015470	Pentachlo	14.02	7
MESP1	55897	Leukemia, MESH:D015470	Dexameth	14.02	7
MICA	1.01E+08	Leukemia, MESH:D015473	Arsenic|Ar	14.02	159
NR1D2	9975	Leukemia, MESH:D015470	Air Polluta	14.02	21
P4HTM	54681	Leukemia, MESH:D015470	Arsenic Tr	14.02	18
PALM	5064	Leukemia, MESH:D015470	Dronabinc	14.02	7
PARN	5073	Leukemia, MESH:D015473	Arsenic Tr	14.02	259
PHYKPL	85007	Leukemia, MESH:D015473	Calcitriol|C	14.02	153
PSMD9	5715	Leukemia, MESH:D015470	Doxorubic	14.02	14
RAE1	8480	Leukemia, MESH:D015470	Dexameth	14.02	13
SLC9A1	6548	Leukemia, MESH:D015473	Arsenic|Ar	14.02	264
SV2C	22987	Leukemia, MESH:D015473	Arsenic|ars	14.02	10
TMEM164	84187	Leukemia, MESH:D015470	Doxorubic	14.02	13
TMEM201	199953	Leukemia, MESH:D015470	Calcitriol|D	14.02	12
UBE2D1	7321	Leukemia, MESH:D015470	Arsenic Tr	14.02	17
UBE2Z	65264	Leukemia, MESH:D015470	Arsenic Tr	14.02	17
ZNF761	388561	Leukemia, MESH:D015470	Dexameth	14.02	9
CD72	971	Leukemia, MESH:D015473	Arsenic|De	14.01	11
CPNE7	27132	Leukemia, MESH:D015473	Arsenic|De	14.01	9
CXCL14	9547	Leukemia, MESH:D007948	2-(2-amin	14.01	3
DDX21	9188	Leukemia, MESH:D015473	Arsenic Tr	14.01	263
HAUS6	54801	Leukemia, MESH:D015473	Arsenic Tr	14.01	262
INO80C	125476	Leukemia, MESH:D015473	arsenite|D	14.01	6
KIAA0586	9786	Leukemia, MESH:D015470	Doxorubic	14.01	11
MT2	17750	Leukemia, MESH:D015473	Arsenic|ars	14.01	12
NUBP1	4682	Leukemia, MESH:D015473	Arsenic|Ar	14.01	158
PLG	5340	Leukemia, MESH:D007948	2-(2-amin	14.01	3
SMIM30	401397	Leukemia, MESH:D015470	Dexameth	14.01	10
SYT5	6861	Leukemia, MESH:D015473	Arsenic|M	14.01	154
TIMM22	29928	Leukemia, MESH:D015473	arsenite|D	14.01	8
USP6	9098	Leukemia, MESH:D015470	Dexameth	14.01	10

VPS8	23355	Leukemia, MESH:D015473	Arsenic|ars	14.01	10
ZNF621	285268	Leukemia, MESH:D015470	Dexameth	14.01	10
AARSD1	80755	Leukemia, MESH:D015470	Dronabinc	14	6
C12ORF76	400073	Leukemia, MESH:D015470	Doxorubic	14	10
C1ORF216	127703	Leukemia, MESH:D015470	Doxorubic	14	10
CDK5R1	8851	Leukemia, MESH:D015473	Arsenic Tr	14	264
DHX33	56919	Leukemia, MESH:D015473	Dexameth	14	152
DPY19L2	283417	Leukemia, MESH:D015470	Doxorubic	14	10
EIF3G	8666	Leukemia, MESH:D015473	Arsenic Tr	14	155
ERGIC3	51614	Leukemia, MESH:D015473	Arsenic|ars	14	156
GDF3	9573	Leukemia, MESH:D015473	Arsenic|Ge	14	155
HBA1	3039	Leukemia, MESH:D015470	Doxorubic	14	15
ITIH4	3700	Leukemia, MESH:D015470	Dexameth	14	9
PCOLCE	5118	Leukemia, MESH:D004915	Doxorubic	14	2
PLAAT3	11145	Leukemia, MESH:D015470	Decitabine	14	17
POPDC3	64208	Leukemia, MESH:D015473	Arsenic|ars	14	156
PRPF18	8559	Leukemia, MESH:D015473	Arsenic Tr	14	260
RCBTB1	55213	Leukemia, MESH:D015473	Arsenic|Mi	14	157
SEC14L5	9717	Leukemia, MESH:D015470	Doxorubic	14	10
SNORA21	619505	Leukemia, MESH:D015470	Doxorubic	14	10
SRSF3	6428	Leukemia, MESH:D015470	Dronabinc	14	11
STAG3L4	64940	Leukemia, MESH:D015470	Doxorubic	14	11
TLN1	7094	Leukemia, MESH:D004915	Doxorubic	14	2
TRIM9	114088	Leukemia, MESH:D015473	Arsenic|ars	14	156
ZNF140	7699	Leukemia, MESH:D015470	Doxorubic	14	10
ZNF555	148254	Leukemia, MESH:D015470	Doxorubic	14	10
ZNF708	7562	Leukemia, MESH:D015470	Doxorubic	14	10
APOO	79135	Leukemia, MESH:D015473	Arsenic|De	14	155
CACNA1G	8913	Leukemia, MESH:D015473	Arsenic|Da	14	22
CYP51A1	1595	Leukemia, MESH:D015470	Dexameth	14	8
GRIK1	2897	Leukemia, MESH:D015470	Benzoates	14	10
