# Supplementary material for: High Expression of ACOT2 Predicts Worse Overall Survival and Abnormal Lipid Metabolism: A Potential Target for Acute Myeloid Leukemia
Source: J Healthc Eng. 2022 Sep 23;2022:2669114. doi: 10.1155/2022/2669114 (PMC9525752; doi:10.1155/2022/2669114)
Supplement: Supplementary Materials — Supplement Table 1. The 13299 AML-related genes from CTD. Supplement Table 2. The 53 ACOT2-related metabolites are based on Human Metabolome Database. [file 2669114.f1.zip › Supplement Table2.pdf]

Oleic acid
Palmitic acid
Linoleic acid
Stearic acid
2-Methylbutyryl-CoA
Arachidonic acid
Linoleoyl-CoA
Octanoyl-CoA
Butyryl-CoA
Isovaleryl-CoA
Stearoyl-CoA
Acetyl-CoA
Isobutyryl-CoA
Propionyl-CoA
Glutaconyl-CoA
Oleoyl-CoA
Palmityl-CoA
alpha-Linolenic acid
Coenzyme A
Eicosapentaenoic acid
Crotonoyl-CoA
Pristanoyl-CoA
Water
Docosahexaenoic acid
Arachidic acid
Hexanoyl-CoA
Dihomo-gamma-linolenic acid
gamma-Linolenic acid
Lauroyl-CoA
(2E)-Dodecenoyl-CoA
trans-2-Hexenoyl-CoA
(2E)-Hexadecenoyl-CoA
(2E)-Tetradecenoyl-CoA
8Z,11Z,14Z-eicosatrienoyl-CoA
(2E)-Decenoyl-CoA
(2E)-Octenoyl-CoA
3Z-dodecenoyl-CoA
Eicosanoyl-CoA
Alpha-Linolenoyl-CoA
Gamma-linolenoyl-CoA
Decanoyl-CoA (n-C10:0CoA)
Heptadecanoyl CoA
Arachidonyl-CoA
Tetracosanoyl-CoA
trans-Octadec-2-enoyl-CoA
2-Methylhexanoyl-CoA
Heptanoyl-CoA
Nonanoyl-CoA
Pentanoyl-CoA
CAS Number
Undecanoyl-CoA
Cervonyl coenzyme A
(5Z,8Z,11Z,14Z,17Z)-Icosapentaenoyl-CoA
